# 38th International Symposium on Intensive Care and Emergency Medicine: Brussels, Belgium. 20-23 March 2018

**DOI:** 10.1186/s13054-018-1973-5

**Published:** 2018-03-29

## P001 Reduced cellular respiration and ATP production in an in vitro model of sepsis

### V Herwanto^1^, Y Wang^1^, M Shojaei^1^, B Tang^2^, AS McLean^2^

#### ^1^University of Sydney, Westmead, Australia; ^2^Nepean Hospital University of Sydney, Nepean Clinical School, Kingswood, Australia

**Introduction:** Leukocyte dysfunction may play a role in sepsis pathogenesis. Established evidence showed that leukocyte dysfunction leads to reduced immune response and consequently an increased sepsis-related mortality. Impaired metabolism has been recently proposed as one possible mechanisms underpinning leukocyte dysfunction in sepsis. In this study, we investigated the global changes in leukocyte metabolism in sepsis, using an established in vitro model of lipopolysaccharide (LPS) stimulation.

**Methods:** Peripheral blood mononuclear cells (PBMC) were isolated from healthy volunteers (n=4) and incubated with 62.5 ng/mL LPS. Mitochondrial respiration was measured using Agilent Seahorse XF Analyzer (Cell Mito Stress Test Kit). Total cellular oxidative stress was measured using DCFDA Cellular Reactive Oxygen Species (ROS) Detection Assay Kit (Abcam) and mitochondrial superoxide was measured using MitoSOXTM (Life Technology). Apoptosis was measured by Annexin V-FITC Apoptosis Detection Kit (Abcam). Evaluation of oxidative stress and apoptosis were performed using BD FACSCanto flow cytometer and flow cytometry data was analyzed using FlowJo Software V10.

**Results:** LPS stimulation of PBMC from healthy volunteers showed a trend of decrease in both oxidative phosphorylation and cellular respiration (Fig. 1). This decrease in cellular metabolism was accompanied by a trend towards an increase in cell death in the stimulated leukocytes (Fig. 2). The increase in cell death was associated with an increase in oxidative stress (total and mitochondria) (Fig. 2), suggesting that the adverse effect of LPS on cellular metabolism may be mediated by an imbalance in redox potential.

**Conclusions:** The LPS stimulation model could provide a useful approach to study the effect of sepsis on leukocyte metabolism. Further study is required to better understand the mechanism of reduced leukocyte metabolism, including the possible role of oxidative stress in reducing cellular respiration and causing leukocyte cell death.

**Fig. 1 (abstract P001).**
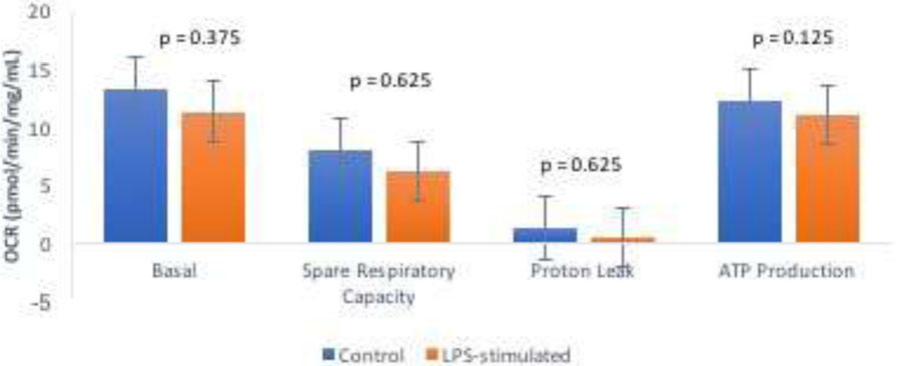
Cellular metabolism as measured in oxygen consumption rate (OCR) (n = 4). Basal denotes energetic demand of the cell under baseline condition; spare respiratory capacity denotes the capability of the cell to respond to energetic demand; proton leak denotes remaining basal respiration not coupled to ATP production, can be a sign of mitochondrial damage; ATP production shows ATP produced by the mitochondria to meet the energetic need of the cell.

**Fig. 2 (abstract P001).**
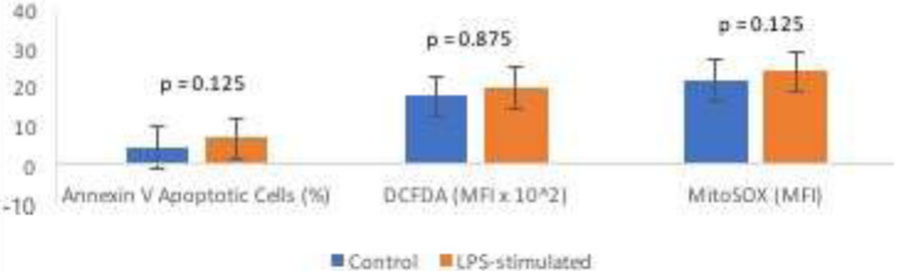
Number of apoptotic cells, total cellular ROS, and mitochondrial superoxide as measured by Annexin V, DCFDA, and MitoSOX (n = 4).

## P002 Noninvasive technique using fluorescence imaging to assess vascular permeability in sepsis

### T Shimazui^1^, T Nakada^1^, L Fujimura^2^, A Sakamoto^2^, M Hatano^2^, S Oda^1^

#### ^1^Chiba University Hospital, Chiba; Japan, ^2^Chiba University, Chiba, Japan

**Introduction:** Conventional assay technique to quantify vascular permeability in animal studies requires sacrifice animals; this becomes a barrier to evaluate of temporal changes or responses to therapeutic approaches in a single individual. In vivo fluorescence imaging potentially quantifies vascular permeability without sacrifice animals. However, the use of this noninvasive approach for the assessment of vascular permeability in remote organ injury caused by systemic inflammatory disease such as sepsis has not been reported.

**Methods:** Cecal ligation and puncture (CLP)-induced septic mouse model was compared to sham and hydrocortisone pretreated (CLP + HC) mouse models. The lung was assumed as an injured remote organ and the footpad was assumed as a noninvasive observational site. The mixture of Evans blue (EB) and fluorescent dye of Genhance 750 were injected into mice, and the extraction of EB in harvested lung was assessed as a conventional indicator of vascular permeability. Fluorescent intensities in the harvested lung or footpad were assessed and their correlation was analyzed to investigate this novel, noninvasive approach to estimation of lung vascular permeability.

**Results:** EB extraction in the harvested lung in the CLP group was significantly higher than in the other groups (CLP vs. sham, P=0.0012; CLP vs. CLP + HC, P=0.011). Fluorescent intensity in the footpad and harvested lung in the CLP group was also significantly higher than in the other groups (footpad, CLP vs. sham, P<0.0001; CLP vs. CLP + HC, P=0.0004; lung, CLP vs. sham, P<0.0001; CLP vs. CLP + HC, P<0.0001). The fluorescent intensity of the footpad was strongly correlated with that of the lung (r=0.95).

**Conclusions:** The fluorescence imaging technique may be useful for assessment of vascular permeability based on EB quantification. The footpad fluorescent intensity was strongly correlated with that of the lung, and may be a suitable indicator in noninvasive estimation of lung vascular permeability.

## P003 Correlation of systemic and regional glycocalyx injury parameters in non-septic critically ill patients

### T Tamosuitis^1^, A Pranskunas^1^, N Balciuniene^1^, D Damanskyte^1^, E Sirvinskas^1^, A Vitkauskiene^1^, E Sneideris^1^, C Boerma^2^

#### ^1^Lithuanian University of Health Sciences, Kaunas, Lithuania, ^2^Medical Center Leeuwarden, Leeuwarden, Netherlands

**Introduction:** The relationship between systemic glycocalyx degradation markers and regional glycocalyx thickness in non-septic critically ill patients is unclear. Conjunctival sidestream dark field-imaging for the purpose of glycocalyx thickness estimation has never been performed. We aimed to investigate whether changes in glycocalyx thickness in conjunctival and sublingual mucosa are associated with global glycocalyx shedding markers.

**Methods:** In this single-centre prospective observational study, using techniques for direct in-vivo observation of the microcirculation, we performed a single measurement of glycocalyx thickness in both ocular conjunctiva and sublingual mucosa in mixed cardio surgical (n=18) and neurocritical patients (n=27) and compared these data with age-matched healthy controls (n=20). In addition we measured systemic syndecan-1 levels

**Results:** In the sublingual and conjunctival region we observed a significant increase of the perfused boundary region (PBR) in both neuro critical and cardiac surgical ICU patients, compared to controls (2.20[2.04-2.42] vs 1.76[1.63-2.08] and 2.19[2.01-2.36] vs. 1.70[1.61-2.00], p<0,05).There was a significant increase of syndecan-1 in ICU patients comparing with controls and in cardiac patients comparing with neurological (120.0[71.0-189.6] vs. 18.0[7.2-40.7], p<0,05). We detected a weak correlation between syndecan-1 and sublingual PBR(r=0.40, p=0.002) but no correlations between global glycocalyx damage markers and conjuctival glycocalyx thickness.

**Conclusions:** Conjunctival glycocalyx thickness evaluation using SDF videomicroscopy is suitable and is impaired in non-septic ICU patients but only measurements in sublingual mucosa are correlating with systemic glycocalyx shedding markers. Global glycocalyx damage is more severe in cardiac comparing to neuro critical patients.

## P004 Glycocalyx degradation in sepsis at admission

### D Beurskens^1^, M Bol^2^, B Broddin^2^, C Reutelingsperger^1^, T Delhaas^1^, M Poll^2^, G Nicolaes^2^, J Sels^2^

#### ^1^Maastricht University, Maastricht, Netherlands; ^2^MUMC, Maastricht, Netherlands

**Introduction:** The purpose of this study is to investigate the endothelial glycocalyx at the onset of sepsis. We hypothesize that the perfused boundary region (PBR) in microvessels (5-25μm) measured by side stream darkfield imaging (SDF) and plasma markers of glycocalyx shedding is increased in non-survivors.

**Methods:** We studied 31 sepsis patients and divided them into survivors (n=17) and non-survivors (n=14) (30-day mortality). SDF measurements and blood sampling were performed within 24h of ICU-admission. ELISAs were used to quantify syndecan-1, angiopoietin-1 (Ang-1) and angiopoietin-2 (Ang-2). Non-parametric tests (Spearman for correlations, Mann Whitney for group comparison) were used to assess statistical significance. A p<0.05 was considered significant. Results are presented as median (25th-75th percentile).

**Results:** Syndecan-1 levels were higher in non-survivors compared to survivors (519.2 (255.6-1056.7) vs. 178.0 (58.1-298.2) ng/ml, p=0.005) (Fig. 1). PBR tended to be higher in non-survivors (2.0 (1.9-2.2)μM) than in survivors (1.9 (1.7-2.1)μM) (p=0.05) (Fig. 2). Syndecan-1 correlated positively with APACHE II (ρ =0.60; p=0.02) and Ang-2/Ang-1 ratio (ρ =0.59; p=0.004), but not with PBR.

**Conclusions:** Plasma markers of glycocalyx shedding at ICU admission are predictors of 30-day mortality and correlate with APACHE II score. However, there is no correlation between these plasma markers and glycocalyx thickness measured by SDF imaging on ICU admission. Further studies should address the cause of this apparent discrepancy to define the role of SDF imaging in the assessment of glycocalyx shedding in sepsis.

**Fig. 1 (abstract P004).**
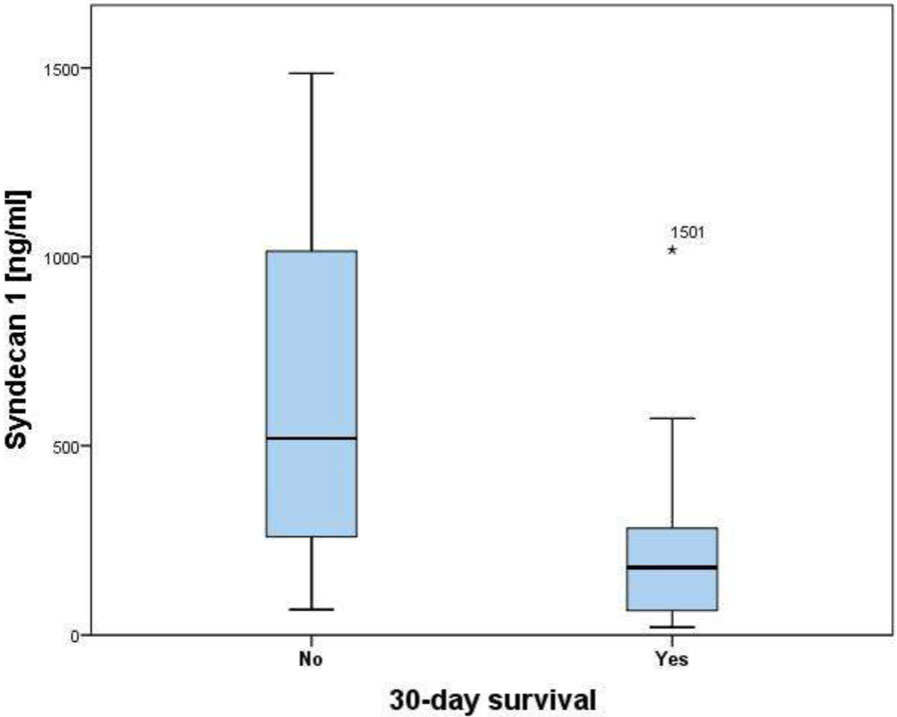
boxplot of Syndecan-1 for survivors and non-survivors

**Fig. 2 (abstract P004).**
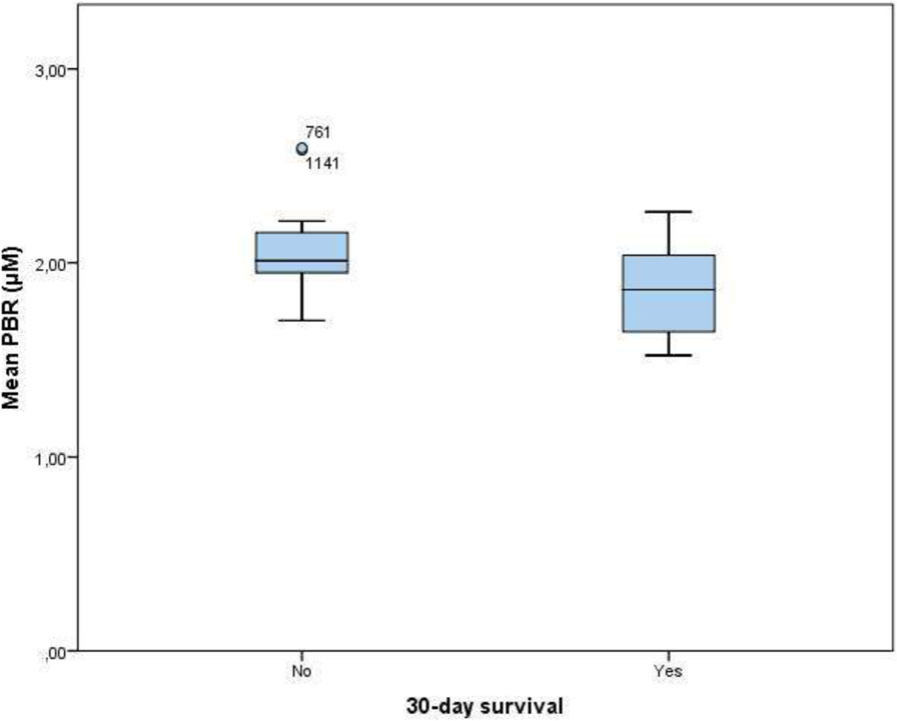
boxplot of PBR for survivors and non-survivors

## P005 The role of glycocalyx shedding and platelet adhesion in sepsis-induced microvascular dysfunction

### CL Manrique-Caballero, CJ Baty, MH Oberbarnscheidt, A Frank, FG Lakkis, BS Zuckerbraun, MR Pinsky, JA Kellum, H Gomez

#### University of Pittsburgh, Pittsburgh, PA, USA

**Introduction:** Sepsis is associated with endothelial activation leading to alterations in global microcirculatory blood flow distribution. These effects might constitute key mechanisms leading to organ dysfunction. The aim of this study was to investigate the role of glycocalyx shedding and platelet adhesion on the development of microvascular dysfunction in the renal peritubular capillary system, and its association to the development of acute kidney injury (AKI).

**Methods:** C57BL/6 (n=6-8/group) mice received vehicle (control), hyaluronidase (140 IU IA q8h during 24 hours) or underwent cecal ligation and puncture (CLP). Platelet adhesion and rolling in renal peritubular capillaries were assessed and quantified using multiphoton IVM in five different areas [1]. Plasma syndecan (SDC-1) and hyaluronan were measured to assess glycocalyx shedding. Neutrophil Gelatinase-Associated Lipocaline (NGAL) and creatinine levels were used as markers of AKI and measured using commercially available assays.

**Results:** Hyaluronidase and CLP resulted in shedding of the glycocalyx (Fig. 1A) and increased platelet adhesion and rolling as compared to control (Fig. 2). Enzymatic-induced shedding did not involve a systemic inflammatory response, as shown by IL-6 levels (Fig. 1B). Irrespective of a systemic inflammatory response, shedding of the glycocalyx and increased platelet adhesion and rolling was associated with increased markers of AKI (Fig. 1C, D).

**Conclusions:** Shedding of the glycocalyx increases platelet adhesion and rolling, resulting in AKI, suggesting this as a potential mechanism of organ injury. The increase in AKI markers in the noninfectious hyaluronidase model without IL-6 elevation indicate that glycocalyx denudation and secondary platelet adhesion may be mechanisms of organ injury independent from sepsis-induced systemic inflammation.

**Reference**

1. Singer, G., et al. Microcirculation 13(2):89-97, 2006.

**Fig. 1 (abstract P005).**
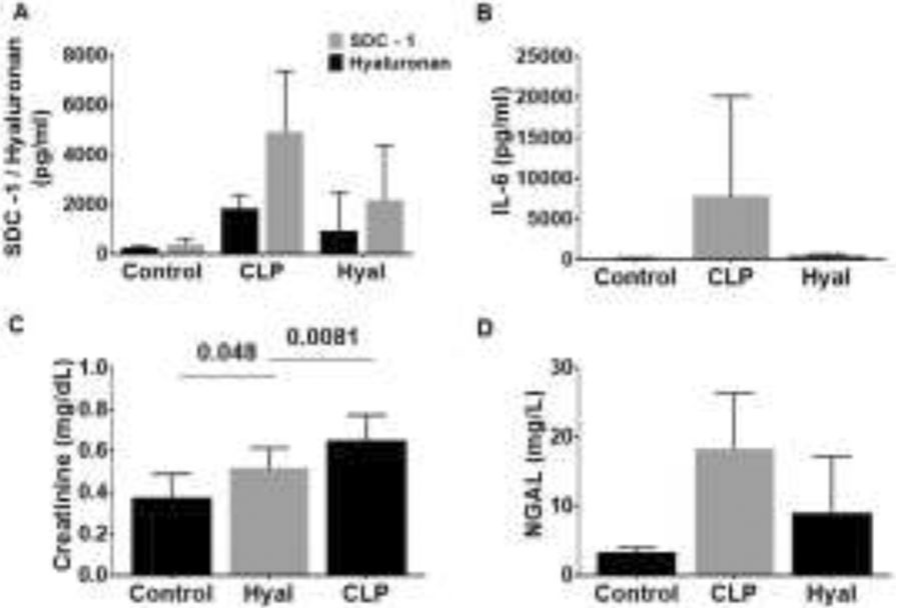
Glycocalyx components, IL-6 and AKI markers

**Fig. 2 (abstract P005).**
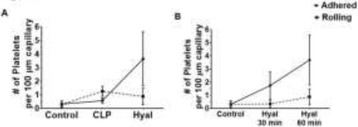
Platelet adhesion and rolling

## P006 AMPK protects against sepsis-induced endothelial dysfunction through cytoskeleton modulation

### M Angé^1^, L Bertrand^1^, C Beauloye^1^, S Horman^1^, D Castanares-Zapatero^2^

#### ^1^Institut de Recherche Experimentale et Clinique - Pôle de Recherche Cardiovasculaire, Brussels, Belgium; ^2^Cliniques Universitaires Saint Luc - Intensive Care Unit, Brussels, Belgium

**Introduction:** Endothelial dysfunction plays a major role in the sepsis related organ dysfunction, and is featured by vascular leakage. AMP-activated protein kinase (AMPK) is known to regulate actin cytoskeleton organization and interendothelial junctions (IEJs), contributing to endothelial barrier integrity. We have already demonstrated its role in defence against sepsis induced hyperpermeability [1], but the underlying mechanisms remain unknown. This project aims to identify molecular targets involved in the beneficial action of AMPK against endothelial barrier dysfunction.

**Methods:** Experiments have been performed in human microvascular dermal endothelial cells. α1AMPK activity has been modulated via the use of a specific siRNA or treatment by two pharmacological AMPK activators (AICAr, 991). We have investigated the effect of this modulation on the expression/phosphorylation of Connexin 43 (Cx43) and Heat shock protein 27 (HSP27), two proteins playing a key role in maintenance of IEJs and actin dynamics respectively.

**Results:** We show that α1AMPK is required to sustain the level of Cx43 expression as it was drastically reduced in cells transfected with a siRNA targeting specifically α1AMPK. Regarding HSP27, its expression level was not affected by α1AMPK deletion. However, both AMPK activators increased its phosphorylation on Ser82, in a α1AMPKdependent manner, while they had no effect on Cx43. Our results also reveal that HSP27 phosphorylation concurred with the appearance of actin stress fibers at the periphery of cells, suggesting a beneficial role for pHSP27 as well as F-actin stress fibers in vascular barrier function through reinforcing the endothelial tethering.

**Conclusions:** Our work identifies the regulation of Cx43 expression and HSP27 phosphorylation as potential protective responses underlying the beneficial action of AMPK against endothelial barrier dysfunction. AMPK could consequently represent a new therapeutic target during sepsis.

**Reference**

1. Castanares D et al. Crit Care Med. 41(12):e411-e422, 2013

## P007 Melusin protects from LPS-induced cardiomyopathy through modulation of calcium channel signalling

### P Arina^1^, A Costamagna^1^, L Brazzi^1^, M Brancaccio^2^, R Giuseppe^1^, N Vitale^2^, L Del Sorbo^1^, P Capello^3^, A Mazzeo^4^, L Mascia^5^, VM Ranieri^6^, M Sorge^2^, V Fanelli^4^

#### ^1^Università di Torino, Dipartimento di Anestesia e Rianimazione, Torino, Italy; ^2^Università di Torino, Molecular Biotechnology Center - Torino, Torino, Italy; ^3^Università di Torino, Department of Molecular Biotechnology and Health Sciences, Torino, Italy; ^4^Università di Torino, Dipartimento di Scienze Chirurgiche, Torino, Italy; ^5^Università degli Studi di ROMA "La Sapienza", Dipartimento di Scienze E Biotecnologie Medico-Chirurgiche, Roma, Italy; ^6^Università degli Studi di ROMA "La Sapienza", Dipartimento di Scienze Cardiovascolari, Respiratorie, Nefrologiche, Anestesiologiche E Geriatriche, Roma, Italy

**Introduction:** Sepsis Induced Cardiomyopathy (SIC) is a serious condition during sepsis with a mortality rate up to 70% (1). SIC is clinically manifested with left ventricle impaired contractility (2). Melusin is a muscle-specific protein involved in sustaining cardiomyocyte survival thorough the activation of AKT signaling pathways (3). PI3K– AKT signaling pathway plays a pivotal role in regulating calcium channel activity (4). We hypothesized that Melusin overexpression could exert a protective effect on cardiac function during septic injury.

**Methods:** Animals were treated with an intraperitoneal injection of lipopolysaccharide (LPS) at 12 mg/kg. SV129 strain Knockout mice (KO) for Melusin gene and FVB strain with cardiac-specific overexpression (OV) of Melusin were compared. Each group was studied together with a control group (WT). Hemocardiac parameters were studied at 0 hour and 6 hours through echocardiography. Another cohort of animals was sacrificed 6 hours after 20 mg/kg LPS treatment and cardiac tissues and blood sample were harvested for Wb analysis to quantify the expression of AKT, P-AKT and CACNA1C and Elisa analysis for Troponin levels.

**Results:** SV129 WT, KO Melusin and FVB WT mice groups, fractional shortening (FS) was significantly impaired after LPS challenge and was associated with compensatory tachycardia (Fig. 1). FVB OV mice group didn’t show decrease in FS. Consistent with the increased AKT phosphorylation observed in OV mice, the expression of CACNA1C was also significantly higher both at basal levels and after LPS treatment in OV mice compared to WT mice (Fig. 2). Troponin levels didn’t differ between mice groups after LPS treatment

**Conclusion:** Melusin has protective role in LPS induced cardiomyopathy, likely through Akt phosphorylation controlling the CACNA1C protein density.

**References**

1. Neri M et al. Mediators Inflamm. 2016:3423450, 2016

2. Sato R et al. J Intensive Care. 3:48, 2015

3. De Acetis M et al. Circ Res. 96(10):1087–94, 2005

4. Catalucci D et al. J Cell Biol. 184(6):923–33, 2009.

**Fig. 1 (abstract P007).**
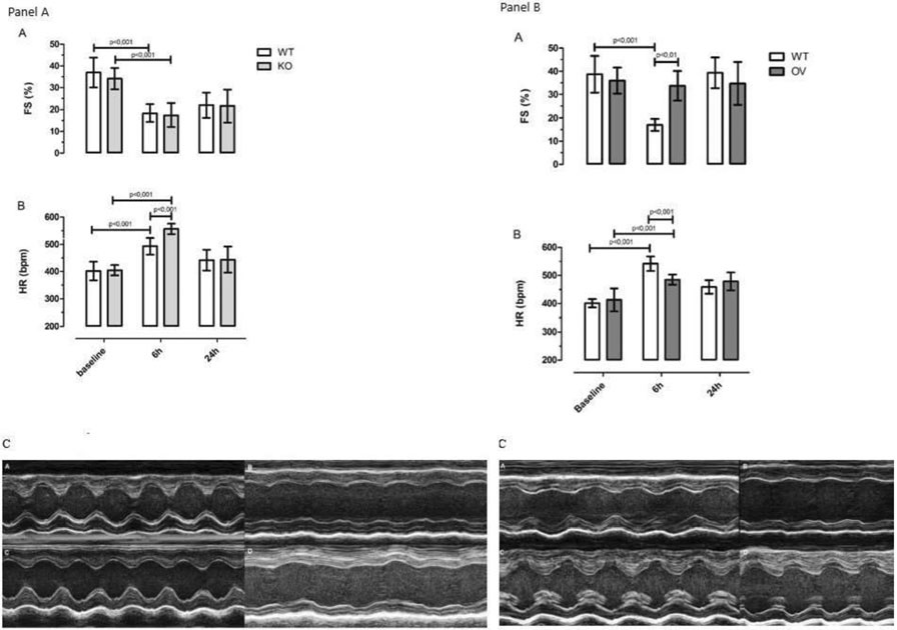
Panel A A. Changes in Fractional Shortening (FS) versus time. Experimental groups are: SV129 WT, SV129 KO for Melusin expression, where n=12 for both groups. B. Changes in Heart Rate (HR) versus time. The groups were the same in A. C. M-mode echography of Left Ventricle (LV) before and after LPS challenge. Panel B A. Changes in Fractional Shortening (FS) versus time. Experimental groups are: FVB WT, FVB OV for Melusin expression, where n=8 for FVB WT and n=12 for FVB OV. B. Changes in Heart Rate (HR) versus time. The groups were the same in A. C. M-mode echography of Left Ventricle (LV) before and after LPS challenge.

**Fig. 2 (abstract P007).**
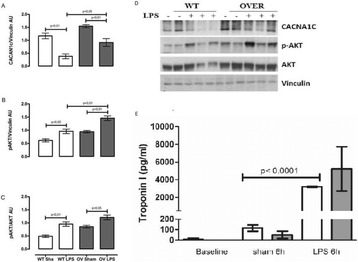
A. Changes in CACNA1C expression versus LPS exposure. Experimental groups are: FVB WT sham and FVB OV for Melusin Expression sham, where n=2 for both groups, B. Changes in pAKT expression versus LPS exposure. Experimental groups are the same in A C. Changes in pAKT/AKT expression versus LPS exposure. Experimental groups are the same in A. D. Western Blot E. Changes in plasmatic Troponin level before and after LPS challenge. Experimental groups are: FVB WT (in white sham and treated), FVB OV (in gray sham and treated).

## P008 Hepatic function is impaired after abdominal surgery and prolonged sedation and mechanical ventilation

### S Liu^1^, M Jakob^2^, A Kohler^2^, D Berger^1^, S Jakob^1^

#### ^1^Inselspital, Bern University Hospital, University of Bern, Department of Intensive Care Medicine, Bern, Switzerland; ^2^Inselspital, Bern University Hospital, University of Bern, Department of Visceral Surgery and Medicine, Bern, Switzerland

**Introduction:** Liver dysfunction is frequent in sepsis, but its pathophysiology remains incompletely understood. Since altered liver function has also been described in ICU patients without sepsis [1,2], the influence of sepsis may be overestimated. We hypothesized that sedation and prolonged mechanical ventilation after abdominal surgery is associated with impaired liver function independent of sepsis.

**Methods:** Sedated and mechanically ventilated pigs underwent abdominal surgery for regional hemodynamic monitoring and were subsequently randomized to fecal peritonitis and controls, respectively (n=4, each), followed by 80 h observation. Indocyanine green (ICG) retention rate 15 minutes after injection of 0.25mg/kg ICG (ICG R15) was determined at baseline, and 11, 32 and 80 h after sepsis induction (SI), and at the same time points in controls. Concurrent with ICG R15, plasma volume, total hepatic perfusion (ultrasound transit time), and bilirubin and liver enzymes were measured. ANOVA for non-parametric repeated measurements was performed in both groups separately.

**Results:** ICG R15 increased over time without significant differences between groups (Table 1). There was a parallel increase in bilirubin in septic but not control animals. The other measured parameters were similar in both groups at the end of the experiment.

**Conclusion:** Liver function was impaired under sedation and prolonged mechanical ventilation after abdominal surgery, even in animals without sepsis. The underlying reasons should be further explored.

**References**

1. Koch A, et al. Crit Care 15:R266, 2011.

2. Sander M, et al. Crit Care 13:R149, 2009.

**Table 1 (abstract P008).** Friedman test was used to evaluate the time effect on parameters in each time point, but only Baseline and SI + 80h were shown in this table. No differences between groups. SI, sepsis induction; PV, plasma volume; HF, hepatic blood flow; ALT, Alanine amino transferase; AST, Aspartate amino transferase; PT, prothrombin time

Parameter	Group	Baseline [Median, (quartile)]	SI + 80h [Median, (quartile)]	P value (Friedman)
ICG R15 (%)	Sepsis (n=4)/Control (n=4)	6.2 (5.3, 19.3)/4.3 (3.3, 19.5)	35.4 (18.7, 47.5)/20.2 (17.4, 33.8)	0.02/0.04
PV (L)	Sepsis (n=4)/Control (n=4)	2.8 (2.5, 3.1)/2.5 (2.4, 2.7)	3.0 (2.3, 3.7)/3.2 (3.0, 3.5)	0.83/0.02
HF (ml/min)	Sepsis (n=4)/Control (n=4)	819 (634, 1054)/1319 (1036, 1969)	890 (631, 1062)/1058 (814, 1265)	0.21/0.44
Bilirubin (μ mol/L)	Sepsis (n=4)/Control (n=4)	0.7 (0.4, 1.0)/1.6 (1.0, 1.9)	4.6 (0.8, 16.3)/0.9 (0.4, 1.8)	0.06/0.13
ALT (IU)	Sepsis (n=4)/Control (n=4)	38.5 (27.0, 49.3)/30.5 (17.2, 36.3)	29.0 (22.5, 40.8)/22.0 (18.8, 24.5)	0.27/0.62
AST (IU)	Sepsis (n=4)/Control (n=4)	60.5 (41.3, 67.0)/68.5 (31.8, 116.5)	60.5 (35.8, 78.5)/39.0 (25.5, 41.3)	0.15/0.02
PT (s)	Sepsis (n=4)/Control (n=4)	13.6 (13.2, 14.5)/13.3 (12.7, 13.8)	14.3 (13.9, 14.7)/13.4 (12.9, 14.2)	0.24/0.27

## P009 Preclinical evaluation of non-anticoagulant heparin in mouse models of inflammation and sepsis.

### GA Nicolaes^1^, DM Beurskens^1^, P Garcia de Frutos^2^, J Van Daal^3^, R Schrijver^3^, B Kool^3^, C Aresté^2^, S De Kimpe^3^, CP Reutelingsperger^1^

#### ^1^Cardiovascular Research Institute Maastricht, Maastricht, Netherlands; ^2^Department of Cell Death and Proliferation, Institute of Biomedical Research of Barcelona, Barcelona, Spain; ^3^Matisse Pharmaceuticals BV, Geleen, Netherlands

**Introduction:** Previous work has shown the cytoprotective properties of antithrombin-affinity depleted heparin (AADH), by neutralization of cytotoxic extracellular histones [1], major mediators of death in sepsis [2,3]. AADH was produced from clinical grade heparin, resulting in preparations that have lost >99,5% of their anticoagulant activity. To gain insight into the mechanisms and the basic pharmacological aspects of AADH protective properties, we performed a systematic analysis of how AADH is tolerated in mice and ascertained its effects in three different in vivo models of inflammation and infection.

**Methods:** Dose ranging studies, short term and medium term, were performed in C57BL/6 mice. The effects of i.v. administration of extracellular histones in the presence or absence of AADH were assessed in mice. We further analysed the effect of AADH in models of Concanavalin A- and MRSA-mediated lethality. In all studies we assessed clinical signs, lab parameters and histology.

**Results:** AADH was well tolerated in both short term and intermediate term (till 7 days) experiments in mice, in the absence of any signs of tissue bleeding. AADH was able to revert the cytotoxic properties of i.v. administered histones.

In a Concanavalin A mediated model of sterile inflammation, we confirmed that AADH has protective properties that counteract the cytotoxic effects of extracellular histones. In an in vivo lethal MRSA model, for the first time, AADH was shown to induce a survival-benefit.

**Conclusions:** We conclude that AADH contributes to the overall increased survival by means of neutralization of extracellular histones and represents a promising product for further development into a drug for the treatment of inflammatory diseases and sepsis.

**References**

[1] Wilhagen et al, Blood 123:1098-1101, 2014

[2] Xu et al, Nat Med 15:1318-1321, 2009

[3] Thromb Res 136:542-7, 2015

## P010 Differential modulation of plasminogen activator mediated thrombolysis by recombinant thrombomodulin and activated protein c

### Z Siddiqui^1^, S Raghuvir^1^, J Fareed^1^, R Wasmund^1^, O Iqbal^1^, W Jeske^1^, D Hoppensteadt^1^, K Tanaka^2^, K Tsuruta^2^

#### ^1^Loyola University Medical Center, Maywood, IL, USA; ^2^Asahai Kasei Pharma America Corporation, Waltham, MA, USA

**Introduction:** Urokinase (UK) and tissue plasminogen activator (tPA) mediate thrombolytic actions by activating endogenous plasminogen. Thrombomodulin (TM) complexes with thrombin to activate Protein C and thrombin activatable fibrinolysis inhibitor (TAFI). Activated Protein C (APC) modulates coagulation by digesting factors V and VIII and activates fibrinolysis by decreasing PAI-1 functionality.

**Methods:** The purpose of this study is to compare the effects of rTM and APC on urokinase and tPA mediated thrombolysis utilizing thromboelastography.

**Results:** Native whole blood was activated using a diluted intrinsic activator (APTT reagent, Triniclot). The modulation of thrombolysis by tPA and UK (Abbott, Chicago, USA) was studied by supplementing these agents to whole blood and monitoring TEG profiles. APC (Haematologic Technologies, VT, USA) and rTM (Asahi Kasai Pharma, Tokyo, Japan) were supplemented to the activated blood at 0.02 – 3.0 ug/ml. The modulation of tPA and UK induced thrombolysis by APC and rTM was studied in terms of thromboelastograph patterns. The effect of both APC and rTM on plasma based systems supplemented with tPA was also investigated.

**Conclusions:** In comparison to rTM, APC produced a stronger anticoagulant effect in terms of r time, k time, angle and MA. 3.0ug/ml rTM and APC did not produce any direct fibrinolytic effects. APC also produced strong augmentation of the lytic of effects of tPA and urokinase. rTM at lower concentrations produced stabilization of clot resisting fibrinolysis.

## P011 Anticoagulant actions of recombinant thrombomodulin and activated protein c and their neutralization by factor viii inhibitor bypass activity (FEIBA)

### Z Siddiqui^1^, W Jeske^1^, M Lewis^1^, P Aggarwal^1^, O Iqbal^1^, D Hoppensteadt^1^, K Tsuruta^2^, J Fareed^1^, S Mehrota^1^, R Wahi^1^

#### ^1^Loyola University Medical Center, Maywood, IL, USA; ^2^Asahai Kasei Pharma America Corporation, Waltham, MA, USA

**Introduction:** Thromboelastographic (TEG) analysis represents a global approach to monitor the clotability of the native whole blood. rTM (recombinant Thrombomodulin) is a mild anticoagulant with pleiotropic actions which are modulated through its complexation with thrombin. Activated protein C (APC) is a stronger anticoagulant which mediates its action via digestions of factor Va and VIIIa. FEIBA (Factor VII Inhibitor Bypass Activity) contains non activated factors II, IX and X and activated factor VII. The purpose of this study is to compare the relative anticoagulant effects of rTM and APC, employing the thromboelastographic analysis and their neutralization by graded amounts of FEIBA.

**Methods:** Citrated whole blood samples were supplemented with rTM and APC at a concentration of 3 ug/mL (n=20). TEG analysis was performed on a TEG 5000 system in which clotting was initiated by re-calcification of the whole blood and set parameters as R time, K time, MA and Angle were measured. The relative neutralization profiles of the APC and rTM by FEIBA at 1.0, 0.1, and 0.01 U/ml were investigated.

**Results:** At 3ug/ml rTM produced a mild anticoagulant effect as evident by its TEG profile in terms of prolongation of R and K times and marked decrease in angle and maximum amplitude. APC at 3 ug/mL produced a relatively stronger anticoagulant effects on all of the parameters in the TEG profile. The supplementation of FEIBA at 0.1 U/mL to the whole blood mixtures containing 3 ug/mL completely neutralized rTM and resulted in the partial neutralization of APC.

**Conclusions:** These results indicate APC is a stronger anticoagulant in comparison to rTM as measured by the TEG analysis. FEIBA is very effective in the neutralization of the anticoagulant effects of rTM and results in a weaker neutralization of APC. These results suggest that FEIBA can be used to neutralize rTM at much lower levels than the proposed dosing for the bleeding control of hemophilia.

## P012 HLA-DRA and CD74 on intensive care unit admission related to outcome in sepsis

### S Cajander^1^, B Stammler Jaliff^2^, A Somell^2^, A Bäckman^1^, H Alpkvist^2^, V Özenci^2^, J Wernerman^2^, K Strålin^2^

#### ^1^Faculty of Medicine and Health, Örebro University, Örebro, Sweden; ^2^Karolinska University Hospital, Stockholm, Sweden

**Introduction:** mRNA expressions of the major histocompatibility complex class II-related genes *HLA-DRA* and *CD74* have been found to be promising markers for sepsis-induced immunosuppression. In the present study we aimed to study how expression of *HLA-DRA* and *CD74* on intensive care unit (ICU) admission were related to death and/or secondary infections in patients with sepsis.

**Methods:** During a full year adult patients admitted to the ICU of Karolinska University Hospital Huddinge were consecutively subjected to blood sampling within 1 hour from ICU admission. Patients treated with antibiotic therapy were eligible for inclusion. The plausibility of infection (definite, probable, possible, none) was determined based on the Centers for Diseases Control (CDC) criteria. Patients with sepsis (definite/probable/possible infection and a SOFA score increase of >=2) were screened for death within 60 days and secondary infections 48 h to 60 days after ICU admission, using the CDC criteria. *HLA-DRA* and *CD74* mRNA expressions were determined by reverse transcription quantitative PCR.

**Results:** Among 579 ICU admissions, a blood sample for RNA analysis was collected in 551 cases. Two hundred fifty-seven patients met the inclusion criteria and provided written informed consent. Sepsis was noted in 134 patients. The sepsis patients experienced death in 36 cases (27%), secondary infection in 32 cases (24%), and death and/or secondary infection in 60 cases (45%). Table 1 shows the results of *HLA-DRA* and *CD74* expression related to death and secondary infections.

**Conclusions:** The mRNA expression of *HLA-DRA* on ICU admission was significantly decreased in patients with sepsis who died or contracted secondary infections within 60 days. *CD74* expression was not significantly decreased in patients with negative outcome.

**Table 1 (abstract P012).** Median values and interquartile ranges (IQR) of HLA-DRA and CD74 in 134 sepsis patients. Calculations were performed on logarithmic scale due to log-normal distribution

Event	HLA-DRA (IQR)	CD74 (IQR)
Death (n=36)	1.14 (0.73-2.19) vs 1.64 (1.01-2.78) p=0.017	2.05 (1.52-3.64) vs 2.70 (1.71-4.18) p=0.22
Secondary infection (n=32)	1.37 (0.65-2.53) vs 1.51 (0.96-2.62) p=0.045	2.25 (1.39-3.43) vs 2.82 (1.74-4.18) p=0.062
Death and/or secondary infection (n=60)	1.21 (0.72-2.53) vs 1.71 (1.06-2.78) p=0.007	2.10 (1.52-3.64) vs 2.99 (1.75-4.19) p=0.070

## P013 Acid-base profile of patients with infection during the first 24 hours of intensive care unit admission

### RM Roepke, BA Besen, PV Mendes, LU Taniguchi, M Park

#### Hospital das Clínicas HCFMUSP, Faculdade de Medicina, Universidade de São Paulo, São Paulo, Brazil

**Introduction:** Acid-base disturbances are common in patients with infection admitted to the intensive care unit (ICU). More attention is given to hyperlactatemia in this patient population as a prognostic factor, although other acid-base disturbances may also have an impact on patient outcomes. Our objective is to describe the acid-base profile of this patient population and determine the association between different acid-base abnormalities and ICU mortality.

**Methods:** Retrospective cohort of patients admitted with infection to an intensive care unit. Patients were stratified according to pH (<7.35; 7.35 – 7.45; > 7.45) and, then, according to the standard base excess (SBE) (< -2; -2 – +2; > +2). In each of these strata and the whole population, the proportions of acid-base disturbances were quantified during the first 24 hours of ICU admission. To assess the association between acid-base disturbances and outcome, a logistic regression model was fit, adjusting for age, sex and SAPS 3 score.

**Results:** 605 patients were analysed. 304 (50%) patients were acidemic and 244 (40%) presented with a normal pH. Metabolic acidosis (as assessed by SBE) was observed in all subgroups, regardless of pH levels (pH < 7.35: 287/304 [94%]; pH 7.35 – 7.45: 184/244 [75%]; pH > 7.45: 34/57 [60%]). Lactic acidosis was observed in 71% of the whole population; SIG (Strong ion gap) acidosis, in 75%; SID (hyperchloremic) acidosis, in 58%; metabolic alkalosis, in 7%; and respiratory acidosis, in 13% of the patients. In multivariate analysis, lactic acidosis (OR 1.85 [95% CI 1.19 – 2.88]), albumin (OR 0.49 [95% CI 0.34 – 0.69]) and phosphate (OR 1.15 [95% CI 1.05 – 1.26]) were the acid-base variables independently associated with ICU mortality.

**Conclusions:** The most common form of acid-base disturbance in patients with infection is SIG acidosis, although only lactic acidosis is independently associated with worse outcomes among strong ions. Weak anions variations are also independently associated with worse outcomes.

## P014 The relationship between serum zinc level and sepsis-induced coagulopathy

### Y Irie, H Ishikura, R Hokama, M Koie, K Muranishi, K Hoshino, R Yuge, T Kitamura, M Iwaasa

#### Fukuoka University Hospital, Fukuoka city, Japan

**Introduction:** Recently, it was clarified that zinc plays a pivotal role of inflammation and its deficiency resulted in deterioration of severe organ dysfunction including coagulopathy in patients with sepsis.

The purpose of present study is to clarify the relationship between serum zinc level and organ dysfunction especially sepsis-induced coagulopathy.

**Methods:** The present study was conducted a single-center retrospective observational study from June 2016 to September 2017. Blood samples were collected on ICU admission.

**Results:** 128 patients were enrolled. Of the 108 patients, 100 patients were sepsis and 8 patients were non-sepsis. The serum zinc levels were significantly lower in the sepsis group than in the non-sepsis group (33.5±19.2 vs 66.5±14.2μ g/dL, p<0.01). Next, we divided sepsis group into two groups using SOFA score (SOFA≧8 and SOFA <8). Zinc level was significantly lower in group of SOFA>8 group than in group of SOFA <8. However, there were no significant between two groups such as calcium, phosphorus and magnesium. We analyzed the relationships between zinc and each factor of SOFA score. There was the most significant correlation between zinc level and Coagulation (r=-0.49, p<0.001). We performed ROC analysis to predict of DIC. The area under the curve of serum zinc level was 0.700, and cut off value of zinc was 25μ g/dL (sensitivity 0.629 and specificity 0.783, p<0.001). Using this zinc cutoff value, the sepsis group was divided into two groups. The 28-day mortality rate of zinc<25μ g/dL group was significantly higher than that of zinc>25 μ g/dL group.

**Conclusions:** Our result suspected that zinc levels are closely related to sepsis induced coagulopathy.

## P015 Association between inflammatory markers and cognitive outcome in patients with acute brain dysfunction due to sepsis

### G Orhun^1^, E Tuzun^2^, P Ergin Ozcan^1^, C Ulusoy^2^, E Yildirim^2^, M Kucukerden^2^, H Gurvit^3^, F Esen^1^

#### ^1^Istanbul University; Medical Faculty of Istanbul, Anesthesiology and Intensive Care, Istanbul, Turkey,^2^Istanbul University, Institute of Experimental Medicine, Neuroscience, Istanbul, Turkey,^3^Istanbul University; Medical Faculty of Istanbul, Department of Neurology, Behavioral Neurology and Movement Disorders Unit, Istanbul, Turkey

**Introduction:** Sepsis-induced brain dysfunction has been neglected until recently due to the absence of specific clinical or biological markers. There is increasing evidence that sepsis may pose substantial risks for long term cognitive impairment.

**Methods:** To find out clinical and inflammatory factors associated with acute sepsis-induced brain dysfunction (SIBD) serum levels of cytokines, complement breakdown products and neurodegeneration markers were measured by ELISA in sera of 86 SIBD patients and 33 healthy controls. Association between these biological markers and cognitive test results was investigated.

**Results:** SIBD patients showed significantly increased IL-6, IL-8, IL-10 and C4d levels and decreased TNF-α, IL-12, C5a and iC3b levels than healthy controls. No significant alteration was observed in neuronal loss and neurodegeneration marker (neuron specific enolase (NSE), amyloid β, tau) levels. Increased IL-1β, IL-6, IL-8, IL-10, TNF-α and decreased C4d, C5a and iC3b levels were associated with septic shock, coma and mortality. Transient mild cognitive impairment was observed in 7 of 21 patients who underwent neuropsychological assessment. Cognitive dysfunction and neuronal loss were associated with increased duration of septic shock and delirium but not baseline serum levels of inflammation and neurodegeneration markers.

**Conclusions:** Increased cytokine levels, decreased complement activity and increased neuronal loss are indicators of poor prognosis and adverse events in SIBD. Cognitive dysfunction and neuronal destruction in SIBD do not seem to be associated with systemic inflammation factors and Alzheimer disease-type neurodegeneration but rather with increased duration of neuronal dysfunction and enhanced exposure of the brain to sepsis-inducing pathogens.

## P016 Altered serum profile of aromatic metabolites reflects the biodiversity reduction of gut microbiota in critically ill patients

### N Beloborodova, E Chernevskaya, A Pautova, A Bedova, A Sergeev

#### Federal Research and Clinical Center of Intensive Care Medicine and Rehabilitology, Moscow, Russia

**Introduction:** High levels of some aromatic microbial metabolites (AMM) in serum are related to the severity and mortality of critically ill patients [1]. Several studies have discussed the imbalance and loss of the diversity of gut microbiota but there are practically no data on the gut microbial metabolites in critical conditions, only a little - in healthy people [2, 3]. The aim of this work is to analyze the connection between serum and fecal levels of AMM in ICU patients.

**Methods:** 13 simultaneously serum and fecal samples (SFS) from ICU patients with nosocomial pneumonia (group I), 21 SFS from ICU neurorehabilitation patients (group II) and 5 SFS from healthy people were taken for GC/MS analyses. The following AMM were measured: phenylpropionic (PhPA), phenyllactic (PhLA), p-hydroxybenzoic (p-HBA), p-hydroxyphenyllactic (p-HPhLA), p-hydroxyphenylacetic (HPhAA), p-hydroxyphenylpropionic (p-HPhPA) and homovanillic (HVA) acids. Data were presented as medians with interquartile range (IR, 25-75%) using STATISTICA 10.

**Results:** The sum of the level of 4 most relevant metabolites (4AMM) - PhLA, p-HPhLA, p-HPhAA, and HVA - in serum samples from group I and group II were equal to 0.9 (0.6-9.6) μ M and 0.7 (0.5-1.0) μ M, respectively, and were higher than in healthy people – 0.4 (0.4-0.6) μ M (p<0.05). We suppose the presence of the correlation of AMM profile in blood and intestine. Particularly, SFS of healthy people are characterized by the prevalence of PhPA; AMM are not detected in feces of non-survivors but only HVA dominates in their serum in the absence of other (Fig. 1).

**Conclusions:** The AMM profiles in gut and serum are interrelated; AMM in serum probably reflect the violation and loss of biodiversity of the gut microbiota in critically ill patients.

**References**

1. Beloborodova NV. Sepsis. Chapter 1. 2017. DOI: 10.5772/68046

2. McDonald D et al. mSphere 1(4): e00199-16, 2016

3. Jenner AM et al. Free Radic Biol Med 38:763–772, 2005

**Fig. 1 (abstract P016).**
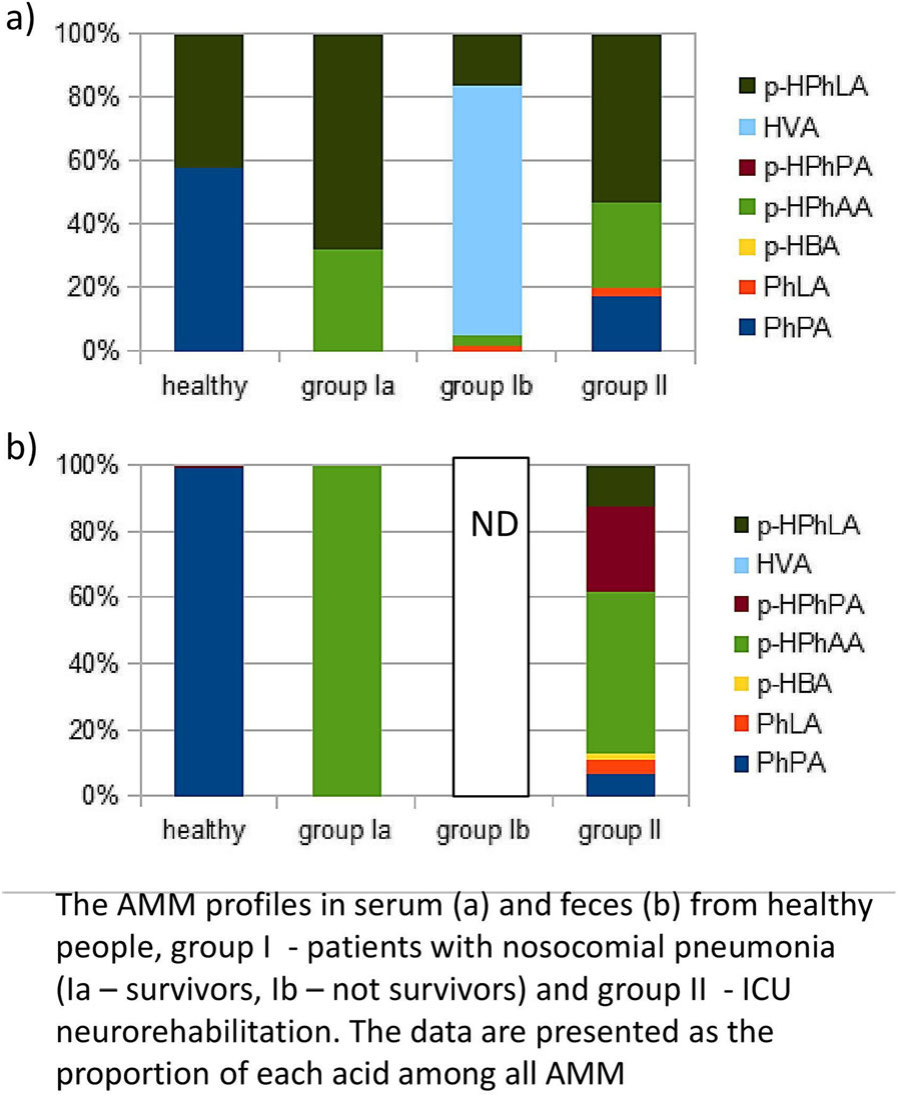
Comparison of the quality profiles of AMM in serum and feces

## P017 Can biomarkers help identifying the type of blood stream infection in septic patients?

### H Brodska ^1^, V Adamkova^1^, K Pelinkova^1^, A Studena^1^, J Zavora^1^, T Drabek^2^

#### ^1^Charles University, Prague, Czech Republic,^2^University of Pittsburgh, Pittsburgh, PA, USA

**Introduction:** Sepsis is one of the most prevalent causes of morbidity and mortality in hospitalized patients worldwide. Early initiation of targeted antibiotic therapy is crucial. Blood stream infections are commonly divided to Gram positive (G+) or Gram negative (G-). However, blood cultures (BC) read-out may be delayed or cultures may be negative (NEG). Biomarkers may help to guide antibiotic therapy prior to BC results [1]. We tested the hypotheses that 1) biomarkers will discriminate between BC- vs. BC+ patients; 2) biomarkers will discriminate between G+ and G- sepsis; 3) biomarkers will correlate with severity of illness.

**Methods:** With IRB approval, a patient cohort (n=60) admitted to mixed ICU for suspected sepsis were enrolled in a prospective observational study. BC and biomarkers of sepsis (C-reactive protein, CRP; procalcitonin, PCT; presepsin, PRE; leukocytes, LEU) were assessed and SOFA and qSOFA were determined on admission. Data are displayed as mean±SD or median [IQR]. One-way ANOVA with post-hoc Tukey’s test, Kruskal-Wallis test or Mann-Whitney test were used as appropriate. Pearson’s test was used to assess correlation between SOFA and biomarkers.

**Results:** CRP was the only biomarker different between BC- (33 [3, 64] mg/L) vs. BC+ (147 [51, 256] mg/L) patients (p=0.003). Numerically higher values were observed in G- patients. CRP was higher in G+ (p=0.006) and G- (p=0.023) vs. NEG. PCT was higher in G+ vs. NEG (p=0.037). LEU were higher in G- vs. NEG (p=0.044) and vs. G+ (p=0.015). PRE was not different between groups (Table 1). In BC- patients, SOFA score did not correlate with any biomarkers. In BC+ patients, SOFA correlated with CRP (p=0.005) and LEU (p=0.023). PRE correlated with SOFA in G+ patients (p=0.032).

**Conclusions:** In our limited sample-size pilot study, tested biomarkers showed limited capacity to identify BC+ patients and the type of infection. Higher values of biomarkers observed in G- sepsis warrant further study.

**Reference**

[1] Brodska H et al. Clin Exp Med 13(3):165-70, 2013.

**Table 1 (abstract P017).**
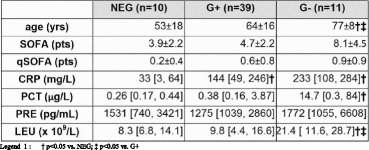
See text for description

† p<0.05 vs. NEG; ‡ p<0.05 vs. G+

## P018 Clinical and diagnostic value of plasma concentration of nitrogen oxide in newborns with respiratory diseases

### MG Pukhtinskaya, V Estrin

#### State Medical University, Rostov-on-Don, Russia

**Introduction:** Since nitrogen oxide (NO) is an essential component of the immune system, the dynamics of plasma NO concentration was studied in order to predict the development of sepsis [1, 2].

**Methods:** With the permission of the Ethics Committee included the 200 full-term newborns with respiratory diseases on a ventilator, retrospectively divided into two groups (I, n=46 - sepsis 4-5 days; II, n=154 without sepsis), at 1, 3-5, 20 days was studied by ELISA the plasma concentration of NO, NOS-2, NOS-3, ADMA (Multilabel Coulter Victor-21420, Finland). To select points "Cut-Off" used the method of ROC-Lines.

**Results:** The statistical power of the study was 86.7% (Î±<0.05). At admission in patients of groups I and II decrease the concentration of NO and increased ADMA in plasma (p<0.05) relative to healthy newborns. After 3-5 days, relatively in patients of groups I and II increased (p<0.05) plasma concentrations of NO, NOS-2, NOS-3, ADMA. NO concentration in patients with sepsis (I) was lower (p<0.05) compared to group II patients at all stages of observation.

NO concentration in plasma of less than 7.30 μmol/l at admission predicted the development of sepsis with a sensitivity of 88.00% and a specificity of 82.66%.

**Conclusions:** The significance of a low concentration of NO in the development of sepsis confirms the relevance of further study of the efficacy, safety and cost-effectiveness of prevention of sepsis, inhaled nitric oxide or other donators NO.

**References**

1. Ryazantseva NV. Cytology 54:105-111.105-111, 2012.

2. Puhtinskaya M. Critical Care 18:288, 2014.

## P019 Plasma myeloperoxidase conjugated-DNA level as predictors of outcome and organ dysfunction in septic shock patients: possible therapeutic effect of hemoperfusion with a polymyxin B immobilized fiber column

### N Takeyama, Y Maruchi, T Gocho, H Mori, N Takenaka, M Tsuda, H Noguchi

#### Aichi Medical University, Aichi, Japan

**Introduction:** We have previously reported that hemoperfusion with polymyxin B immobilized on polystyrene fibers in a cartridge (PMX-DHP) reduces endothelial damage by selective removal of activated neutrophils. Ex vivo perfusion experiments demonstrated that activated neutrophils adhered preferentially to PMX filters and that the remaining neutrophils caused less endothelial damage. There is, however, no report about the effect of PMX-DHP on neutrophil extracellular traps (NETs) formation. The objectives of this study were to investigate the correlations between plasma myeloperoxidase (MPO) conjugated-DNA level with degree of organ dysfunction, disease severity, and ICU mortality in septic shock patients. We also investigated the effect of PMX-DHP on MPO-DNA level.

**Methods:** Sixty-five septic shock patients admitted at the ICUs of 35 Japanese hospitals treated with PMX-DHP were enrolled. Septic shock was identified using old definition according to the ACCP/SCCM in 1997. Plasma MPO-DNA was measured by sandwich ELISA with anti-MPO and anti-DNA monoclonal antibodies.

**Results:** On day 1, septic shock patients displayed a marked increase of plasma MPO-DNA level compared with the healthy volunteers (p=0.008). Plasma MPO-DNA levels were significantly decreased on days 3 and 7 after PMX hemoperfusion. By correlation study, the MPO-DNA level on 7th day was inversely correlated with both the mean arterial pressure (p=0.048) and P/F ratio (p=0.003) at 7th day. Positive correlation was observed between plasma MPO-DNA level on 7th day and SOFA at 7th day (p=0.017). Using Wilcoxon signed-rank test the high MPO-DNA on 3rd day was found to be associated with the hospital mortality (p=0.019).

**Conclusions:** High MPO-DNA levels at 3rd and 7th days of septic shock patients are associated with the degree of organ dysfunction and hospital mortality. The beneficial effects of PMX-DHP may be at least partially due to the inhibition of excessive NETs formation.

## P020 Prognostic role of neutrophil lymphocyte ratio (NLR) in critical illness

### M Ferrari, G Keegan, K Williams, ID Welters

#### Royal Liverpool University Hospital, Liverpool, UK

**Introduction:** Neutrophil-to-lymphocyte ratio (NLR) has been used to predict patient outcomes, with higher values linked to negative prognosis [1]. However, most studies investigate NLR on admission to Critical Care only [2]. In this study we analysed NLR values and trends over a 7-day observation period to define different time courses in survivors and non-survivors of critical illness.

**Methods:** This retrospective study included Intensive Care Unit (ICU) patients admitted to the Royal Liverpool University Hospital over a 4-year period. Age, gender, sepsis status (type of sepsis and date of sepsis), length of ICU stay, 28-day mortality and ICU outcome were collected. WCC, lymphocytes and neutrophils values were recorded for the first 7 days after ICU admission. NLR values were calculated for the first 7 days of intensive care admission. Patients with haematological malignancies and readmissions were excluded.

**Results:** Data were available for 542 patients. 28-day mortality was 20.3% and ICU mortality was 16.6%. Mean NLR in survivors decreased over the 7-day period, whereas mean NLR in non-survivors remained high throughout the 7-day period. Comparing the mean NLR value of the first 2 days (days 1 and 2) with the mean NLR value of the last 2 days (days 6 and 7), patients were divided into 3 groups: decreasing, stable and increasing NLR. The 28-day mortality was respectively 12,4%, 29,9% and 37,8% (P value < 0,01) and the ICU mortality was 9,7%, 24,8% and 32,4% (P-value < 0,01).

**Conclusions:** NLR trend over the first 7 days correlates with mortality in ICU, differing significantly between survivors and non-survivors, and could be used as an outcome predictor.

**References**

1. Hwang SY et al. Am J Emerg Med 35(2):234-239, 2017.

2. Salciccioli JD et al. Crit Care 19:13, 2015.

## P021 The platelet, eosinophil, age, red cell distribution width (PEAR) score and out-of-hospital outcomes in critical illness survivors

### L Maher^1^, L Roeker^2^, T Moromizato^3^, F Gibbons^4^, KB Christopher^5^

#### ^1^The Lahey Hospital & Medical Center, Burlington, VT, USA,^2^Memorial Sloan Kettering Cancer Center, New York, NY, USA,^3^Okinawa Southern Medical Center and Children’s Hospital, Naha, Japan,^4^Massachusetts General Hospital, Boston, MA, USA,^5^Brigham and Women's Hospital, Boston, MA, USA

**Introduction:** Simple and accurate identification of high-risk critical illness survivors may allow for targeted monitoring and interventions that may improve outcomes. Our primary study objective was to determine if a risk prediction score based on demographics and surrogates for inflammation measured at ICU admission was predictive for post-hospital outcomes.

**Methods:** The PEAR score was previously derived and validated for mortality utilizing ICU admission data (age, gender, surgical vs. medical patient type, red cell distribution width, platelet count and peripheral blood eosinophil count). We performed a 2 center cohort study of 67,591 patients admitted to a MICU or SICU from 1997-2012, who survived to hospital discharge. The primary outcome was 90-day post-hospital discharge mortality. Adjusted odds ratios were estimated by logistic regression models including terms for the PEAR score, sepsis, number of acute organ failures, Deyo-Charlson comorbidity index, and the actual length of stay less the national geometric mean length of stay.

**Results:** The cohort was 58% male, 49% surgical ICU with a mean age of 62 years and a 90-day mortality rate of 7.8%. 10% were diagnosed with sepsis. Mean length of stay was 11.2 days. Unplanned 30-day readmission rate was 14%. Patients with the second highest and highest quartile of PEAR Score have an adjusted OR of 90-day post-discharge mortality of 3.95 (95%CI, 3.45-4.52; P< 0.001) and 8.21 (95%CI, 7.16-9.43; P< 0.001) respectively, relative to patients with the lowest quartile of PEAR Score. The AUC for the adjusted prediction model was 0.77. Further, patients with the second highest and highest quartile of PEAR Score have an adjusted OR of 30-day readmission of 1.38 (95%CI, 1.29-1.48; P< 0.001) and 1.64 (95%CI, 1.52-1.78; P< 0.001) respectively, relative to patients with the lowest quartile of PEAR Score.

**Conclusions:** Our simple PEAR score robustly predicts the risk of out of hospital outcomes in critical illness survivors.

## P022 Validation of the neutrophil-lymphocyte count ratio and leukocyte score as prognostic markers in community acquired pneumonia

### M Morales-Codina, R Subirana, A Pérez, N Bacelar, J Font, N Angrill, M Gallego, E Diaz, J Vallés

#### CSU Parc Tauli, Sabadell, Spain

**Introduction:** Some studies suggest the Neutrophil-Lymphocyte Count Ratio (NLCR) and Leukocyte Score (LS) are better in stratifying Community Acquired Pneumonia (CAP) severity than traditional methods. Both can be quickly performed with standard practice cost-effective exams but require further investigation [1, 2].

**Methods:** A retrospective analysis was performed on all ED adults with CAP from Oct. 2009 to Jan. 2011. Demographics, FINE, CURB-65, ATS criteria and initial blood tests were collected, admission to ICU and outcome at 30 days evaluated. The scoring systems prognostic value were compared through ROC curves. Cutoffs used were >=10:1 for NLCR and a score >=2 points for LS.

**Results:** 1059 patients were enrolled (mean age 65.3 ± 19.7 years, 61.7% male). The most prevalent comorbidities were COPD (18.2%), chronic renal disease (10.3) and solid neoplasm (7.2). ICU admission rate was 6.2% with an average APACHE II of 17.6 points. Overall mortality was 8.3%, 16.7% in ICU. The average CURB-65 scoring was 1.4 ± 1.2 points and FINE 92.6 ± 43.5. 21.3% met >=3 minor ATS criteria.

In our population the area under the ROC curve were, respectively for predicting admission to ICU and mortality at 30 days, 0.616 and 0.530 for NLCR, 0.559 and 0.615 for IS; versus 0.654 and 0.875 for FINE score, 0.756 and 0.739 for the ATS criteria, whereas 0.706 and 0.796 for the CURB-65.

**Conclusions:** The NLCR and LS showed a lower discriminative power compared to traditional FINE, ATS criteria and CURB-65 when applied in a much larger population than their original studies.

**References**

1. Cornelis PC et al. PLoS One 7:e46561, 2012

2. Blot M et al. Open Forum Infectious Diseases 1:ofu075, 2014

## P023 Biomarkers of platelet activation and their prognostic value in patients in sepsis associated coagulopathy

### D Hoppensteadt^1^, G Wegryzn^1^, A Walborn^1^, P Maia^1^, S Walborn^1^, R Green^1^, M Mosier^1^, M Rondina^2^, J Fareed^1^

#### ^1^Loyola University Medical Center, Maywood, IL USA, ^2^University of Utah School of Medicine, Salt Lake City, UT, USA

**Introduction:** Sepsis-associated disseminated intravascular coagulation (SAC) is associated with decreased platelet counts and formation. The widespread activation of platelets contribute to vascular occlusions, fibrin deposition, multi-organ dysfunction, contributing to a two-fold increase in mortality. The purpose was to measure markers of platelet function in the plasma of patients with clinically established SAC and to determine association to disease severity and outcome.

**Methods:** Plasma samples from 103 adult intensive care unit (ICU) patients with sepsis and suspected SAC were collected at baseline and on days 4 and 8. DIC scores were calculated using platelet count, D-Dimer, INR, and fibrinogen. Patients were categorized as having no DIC, non-overt DIC, or overt DIC. Plasma levels of CD40L, von Willebrand Factor (vWF), platelet factor-4 (PF-4), and microparticles (MP) were quantified using commercially available ELISA methods.

**Results:** Markers of platelet activation were significantly elevated in patients with sepsis alone and with suspected DIC compared to normal healthy individuals on ICU day 0 (p<0.001). Levels of platelet-associated biomarkers were compared between survivors and non-survivors. PF-4 was significantly decreased in non-survivors compared to survivors (p = 0.0156). Patients were stratified based on platelet count and levels of markers were compared between groups. CD40L, vWF, PF4, and MP showed significant variation based on platelet count, with all markers exhibiting stepwise elevation with increasing platelet count.

**Conclusions:** Markers of platelet activation were significantly elevated in patients with SAC compared to healthy individuals. PF4 levels showed significant difference based on DIC score or mortality, and differentiated the non-survivors compared to survivors. CD40L, vWF, PF4, and MP showed significant association with platelet count, increasing in a stepwise manner with increases in platelet count (Table 1).

**Table 1 (abstract P023).** Markers of platelet activation on Day 0 vs. platelet count

	<50 (k/ul)	50-99 (k/ul)	100-149 (k/ul)	>150 (k/ul)
CD40L (pg/ml)	127.0 ± 46.0	158.0 ± 42.0	274.0 ± 37.0	496.0 ± 94.0
vWF (%)	218.0 ± 35.0	268.0 ± 18.0	274.0 ± 16.0	232.0 ± 8.6
PF4 (ng/ml)	46.0 ± 3.4	52.0 ± 5.6	81.0 ± 8.2	84.0 ± 4.6
MP (nM)	20.0 ± 4.8	20.0 ± 5.5	29.0 ± 4.3	47.0 ± 4.0
n	5	16	20	61

## P024 Prognostic value of mean platelet volume in septic patients: a prospective study

### A Chaari

#### King Hamad University Hospital, Bussaiteen, Bahrain

**Introduction:** Mean Platelet Volume (MPV) has been reported as a valuable marker of inflammatory diseases. The aim of the current study is to assess the prognostic value of MPV in septic patients.

**Methods:** Prospective study including all patients admitted to the intensive care unit (ICU) with sepsis or septic shock. Demographic, clinical and laboratory data were collected. The MPV was checked on admission and on day 3. Two groups were compared: Survivors and non-survivors.

**Results:** Thirty-four patients were included. Median age was 69[62-77] years. sex-ratio was 1.8. Median APACHEII score was 21[16-28]. Platelets count on admission was 264[177-391] with a MPV of 8.4[8-9.3] FL. On day3, platelets count was 183[101-265] with a MPV of 8.6[8-9.4] FL. MPV increased on day 3 in 19 patients (55.9 %). Mechanical ventilation was required for 20 (58.8 %) patients and CRRT was required for 14 (41.2 %) patients. The ICU length of stay was 7[3-12] days. Twelve patients died in the ICU (35.3 %).

Survivors were younger than non-survivors (66[60-76] versus 73[69-86] years; p = 0.016) and had lower APACHEII score (20[15.8-25.5] versus 27[20-33.5]; p = 0.04). MPV on admission and on day 3 were comparable between the two groups (respectively 8.3[8-9.5] versus 8.5[7.6-9.3] FL; p = 0.999 and 8.5[7.9-9.3] versus 9[8.2-9.6] FL; p = 0.369). However, the platelets count on D3 was significantly lower in the non-survivors (227[132-336] versus 113[44-231]; p = 0.049). The ICU length of stay was 7[3-12] days in survivors and 8.5[3.5-12] days in non-survivors (p=0.623).

**Conclusions:** The decrease of the platelet count but not the increase of the MPV was associated with increased mortality in critically-ill septic patients.

## P025 Endotoxin activity assay levels measured within 24 hours after ICU admission affected patients’ severity assessments

### A Kodaira^1^, T Ikeda^2^, S Ono^2^, S Suda^2^, T Nagura^2^

#### ^1^Tokyo Medical University, Tokyo, Japan,^2^Tokyo Medical University, Hachioji Medical Center, Tokyo, Japan

**Introduction:** Endotoxin is a major component of the cell wall of Gram-negative bacteria and is the principal molecule responsible for the induction of septic shock. A prospective cohort study (MEDIC study) of 857 consecutive new ICU patients evaluated the usefulness of endotoxin activity assay (EAA) as a diagnostic tool in sepsis and septic shock.

**Methods:** This study was performed to classify EAA values measured within 24 hours of ICU admission into a high risk (H) group (EAA>0.6; N=154; mean age ± SD = 64±13; median 70) and a low risk (L) group (EAA<0.4; N=174; 68±16; 72), and then, to evaluate patient severities (APACHE 2 score, SOFA score) and sepsis-related biomarkers (procalcitonin, IL-6, angiopoietin 2), comparing groups. Results were expressed as the mean ± SD (median). The Mann-Whitney U-test and chi-square test or Fisher’s test were used for statistical analysis.

**Results:** The APACHE 2 score of the H-group was 26.5±9.5 (27.0), while that in the L-group was 19.9±9.1 (18.0), and the difference between the groups was statistically significant (p<0.05). The SOFA score of the H-group was 9.6±4.1 (10.0) and that of the L-group was 7.2±4.6 (6.5) (p<0.05).

PCT of the H-group was 37.8 ±58.5(11.7)and that in the L-group was 9.6±25.5 (1.6), but there was no significant difference between the groups. IL-6 of the H-group was 19,483±61,281 (1160) and that of the L-group was 6256±39,321 (144). Angiopoietin 2 of the H-group was 12,822±10,593 (10,100) and that in the L-group was 6004±4441 (4105). These two biomarkers indicated significant differences between the groups.

Survival rate of the H-group was 78.9% (153 survived, 41 died), and that of the L-group was 85.5% (147 survived, 25 died). Statistically, there was no significant difference between the groups.

**Conclusions:** These results indicate that the EAA value measured within 24 hours after ICU admission is a useful marker for a patient’s severity assessment, but not for outcome prediction.

## P026 Interleukin 10 release after ex vivo stimulation of whole blood predicts clinical outcomes in sepsis

### H Perrichet^1^, C Martin-Chouly^2^, JT Ross^3^, C Rousseau^2^, P Seguin^1^, N Nesseler^1^

#### ^1^University Hospital Pontchaillou, Rennes, France,^2^Rennes 1 University, Rennes, France,^3^University of California, San Francisco, CA, USA

**Introduction:** Sepsis profoundly alters immune homeostasis by inducing first a systemic pro-inflammatory, then an anti-inflammatory state. We evaluate the prognostic value of ex vivo lipopolysaccharide (LPS) stimulation of whole blood in septic patients, at day 1 and 7 after intensive care unit (ICU) admission.

**Methods:** This prospective cohort study included patients with severe sepsis or septic shock admitted to a surgical ICU of a university hospital. Blood was drawn on day 1 and day 7, and stimulated ex vivo with LPS for 24 hours. Tumor necrosis factor alpha (TNF), interleukin (IL) 1, IL6 and IL10 were measured. Twenty-three healthy adults served as controls. Outcomes were ventilator and ICU-free days, SOFA score at day 1 and 7, and need for dialysis during the course of sepsis.

**Results:** Forty-nine patients were included (mean age 62 ± 15 years). The blood of septic patients was less responsive to ex vivo stimulation with LPS than that of healthy controls, as demonstrated by lower TNF, IL1, IL6 and IL10 release (Fig. 1). At day 1, patients above the 50th percentile of IL10 release had significantly fewer ventilator and ICU-free days than those in the lower 50th percentile (Fig. 2). In contrast, patients in whom IL10 release increased between day 1 and day 7 had significantly lower SOFA scores at day 1 and 7 and need for dialysis, and more ICU-free days than patients in whom IL10 release decreased (Table 1).

**Conclusions:** Greater LPS-stimulated IL10 release in septic patients at day 1 was associated with poorer clinical outcomes and may reflect the severity of the forthcoming immunoparalysis. However, an increase in IL10 release between day 1 and day 7 was associated with favorable outcomes, perhaps signaling immune restoration.

**Table 1 (abstract P026).** Clinical outcomes of septic patients according to interleukin 10 release evolution between day 1 and 7 after ICU admission

	Patients who decrease IL10 release (n=11)	Patients who increase IL10 release (n=24)	p
Age (years, mean ± SD)	58 ± 17	61 ± 15	0.4
SOFA score at day 1 (mean ± SD)	9 ± 4	6 ± 3	0.02
SOFA score at day 7 (mean ± SD)	5 ± 5	2 ± 2	0.02
ICU free-days (days, mean ± SD)	12 ± 10	20 ± 6	0.04
Ventilation free-days (days, mean ± SD)	20 ± 9	25 ± 4	0.2
Dialysis (n, %)	6 (54)	1 (4)	0.002
3-month mortality (n, %)	1 (9)	2 (8)	1

**Fig. 1 (abstract P026).**
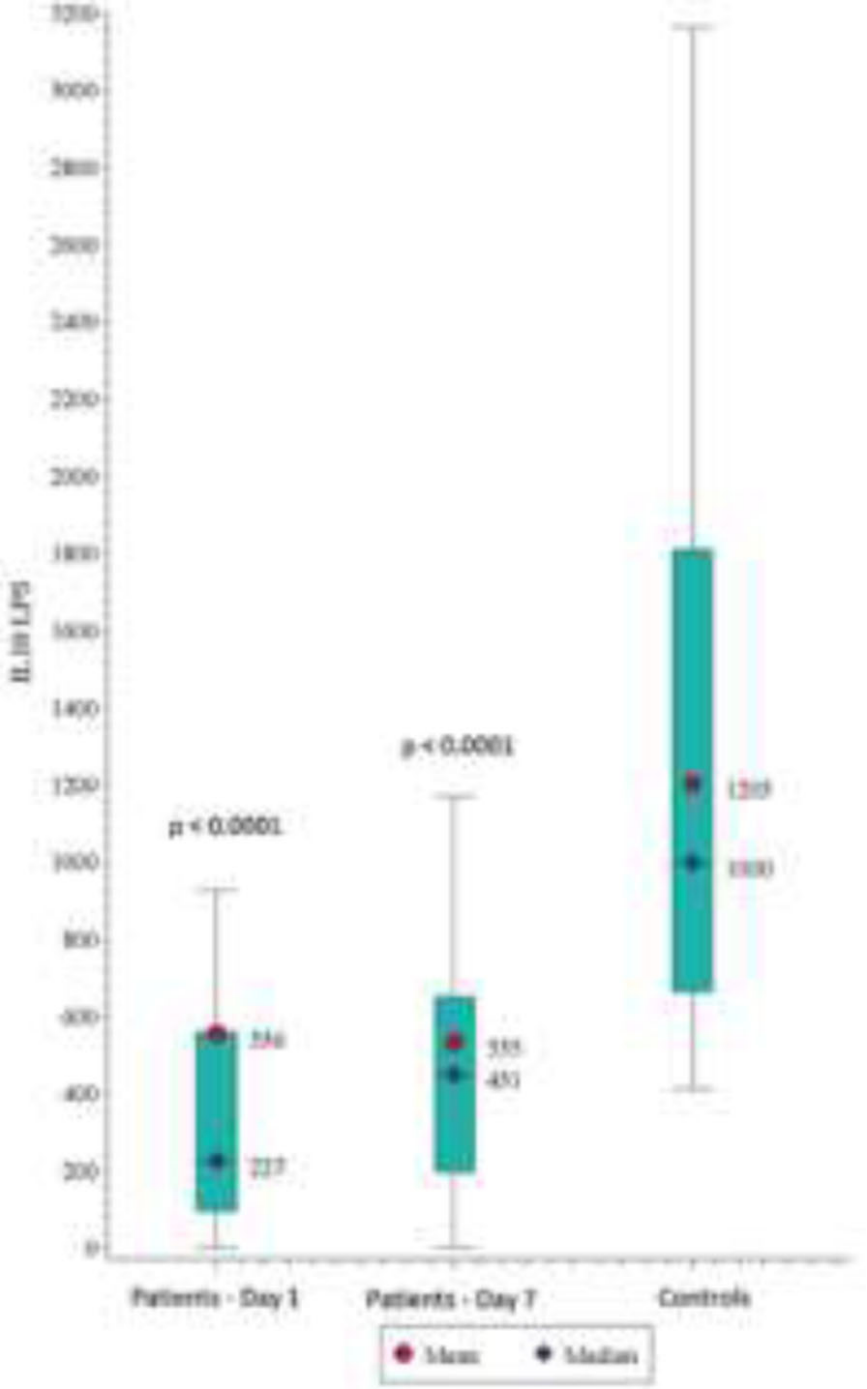
LPS-stimulated IL10 levels in septic patients and controls at day 1 and 7 after ICU admission. Results are presented in pictograms per milliliter (pg/mL)

**Fig. 2 (abstract P026).**
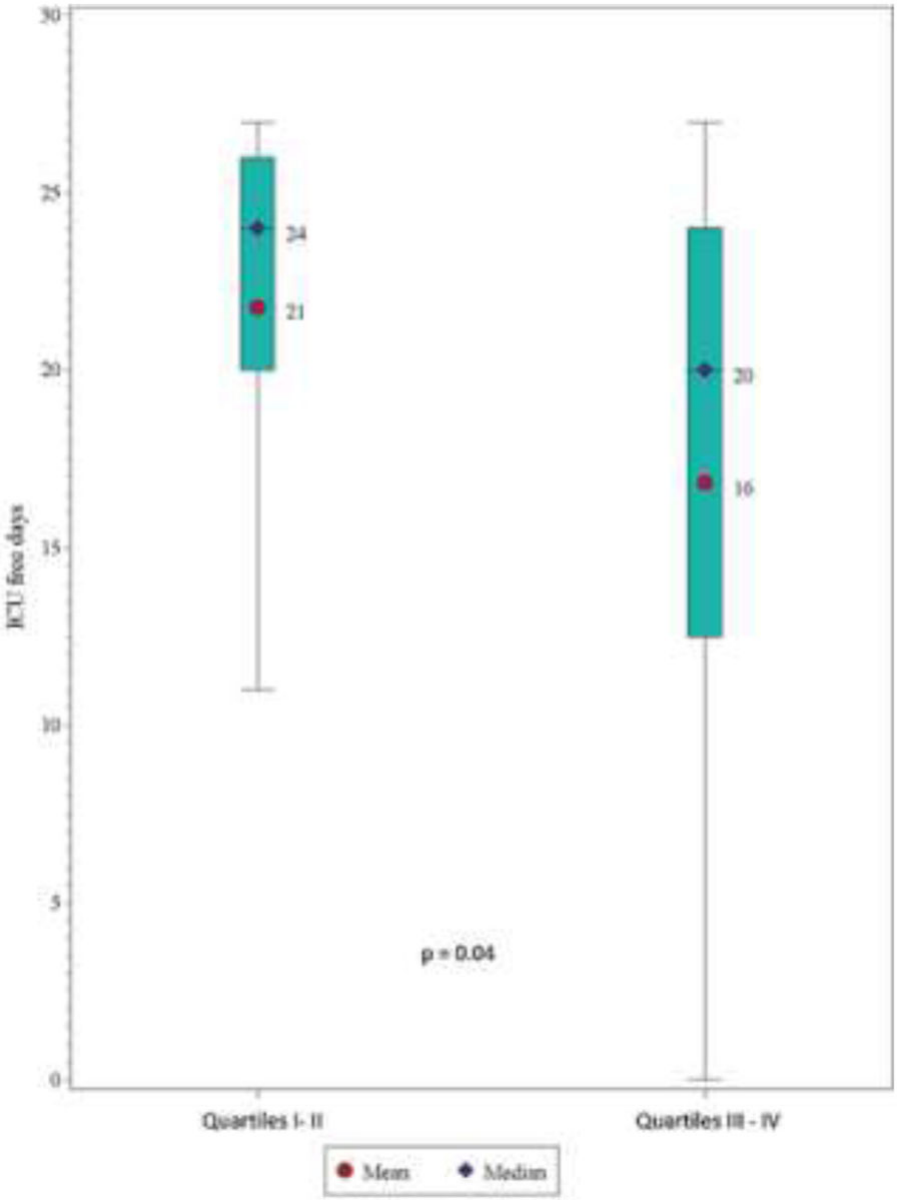
Number of ICU-free days to day 28 between the lowest group of LPS-stimulated IL10 production (quartiles I and II) and the highest group of LPS-stimulated IL10 production (quartiles III and IV)

## P027 Is serum procalcitonin a reliable marker of bacterial sepsis after hyperthermic intraperitoneal chemotherapy with cytoreductive surgery (HIPEC-CRS)?

### Y Al Drees, A Alrbiaan, A Elhazmi, T Amin, N Salahuddin

#### King Faisal Specialist Hospital and Research Centre, Riyadh, Saudi Arabia

**Introduction:** Hyperthermic Intraperitoneal Chemotherapy with Cytoreductive Surgery (HIPEC-CRS) is a curative treatment modality for peritoneal carcinomatosis. Extensive debulking surgery, peritoneal stripping and multiple visceral resections followed by intraperitoneal installation of heated high-dose chemotherapeutic agents, a process leads to a ‘high-inflammatory’ syndrome. Serum procalcitonin (PCT), a biomarker for bacterial sepsis, in the heightened inflammatory state after HIPEC-CRS might be of limited utility. Our aim is to determine the trends of PCT in the early postoperative phase of HIPEC-CRS and to identify trends in patients with and without bacterial sepsis

**Methods:** In a case-control design, we reviewed all patients undergoing HIPEC-CRS over a 24-month period (2015-2017). Patients were divided into 2 groups based on whether they developed bacterial sepsis in the first 5 days after surgery (infected v/s non-infected). Summary data are expressed as medians and ranges. Two-tailed nonparametric tests were performed and considered significant at p values of less than 0.05

**Results:** 82 patients’ data was analyzed. Infections developed in 16% (13 patients) with Escherichia coli as the predominant pathogen isolated (36% isolates). PCT levels (ngm/ml) were elevated postoperatively in both infected and non-infected patients; Day 1 infected 0.97 (IQR 0.5, 3.2) v/s non-infected 0.68 (0.2, 1.6) p=not significant (ns), Day 2 infected 1.14 (0.8,4.2) v/s 1.2 (0.5, 3.4) p=ns, Day 3 infected 1.82 (0.6, 11.5) v/s 0.73 (0.3, 2.2) p=0.05. The differences became statistically significant only by the 4th day (Fig. 1); Day 4 infected 1.32 (0.4, 8.2) v/s 0.53 (0.19, 1.1) p=0.012, Day 5 infected 0.85 (0.09, 3.9) v/s 0.28 (0, 0.7) p=0.047

**Conclusions:** HIPEC-CRS is associated with an early postoperative increase in PCT levels, independent of the presence of bacterial sepsis. The study demonstrate that HIPEC-CRS is a stimulus for PCT release and that decisions for antimicrobial therapy should not be based solely on elevated PCT values

**Fig. 1 (abstract P027).**
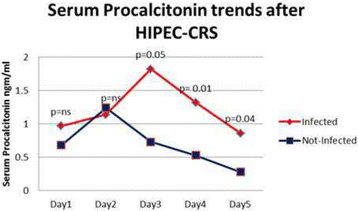
Daily serum Procalcitonin levels post HIPEC-CRS shows statistically significant difference between infected and non-infected patients only on the 4th post-operative day

## P028 Early prognostic value of serum procalcitonin in post cardiac surgery patients with fever.

### S Sudarsanan, A Pattath, A Omar, P Sivadasan, S Aboulnaga, F Hamwi, A Al Khulaifi

#### Heart Hospital, Hamad Medical Corporation, Doha, Qatar

**Introduction:** Early outcome in cardiac surgery has been an area of growing interest where the given risks raise several predictive models for assessment of postoperative outcome [1]. Procacitonin (PCT) emerges as a possible predictive tool in cardiothoracic intensive care unit (CTICU).We aim at testing the predictive power of PCT for early morbidity, prolonged ventilation, ICU and hospital stay, in patients developing early fever after cardiac surgery

**Methods:** A retrospective descriptive study done in tertiary cardiac center, enrolling patients who stayed for more than 24 hours post-operatively in the CTICU Risk stratification included additive Euro score and PCT immunoluminometricaly prior to surgery and every 48 hours in response to onset of fever.

**Results:** We screened 501 consecutive patients who underwent open heart cardiac, of which 119 patients were enrolled in the study. Patients were divided into two groups based on the level of PCT, those with value > 2 ng/ml (Group 1) and those with level < 2 ng/ml (Group 2). Patients in group 1 as compared to Group 2, over the postoperative course was associated with prolonged ICU stay (P=0.04), length of mechanical ventilation (P=0.05), length of hospitalization (p=0.05), acute kidney injury (P=0.04) and culture positivity (P=0.02). Multivariate analysis showed that PCT >2ng/ml was was significantly associated with positive cultures. (p=0.023)

**Conclusions:** A rise of serum PCT carries the signals of early ICU morbidity and lengths of ventilation, ICU stay and hospital stay

**Reference**

1. Iyem H. Cardiovasc J Afr 20(6):340-3, 2009.

## P029 Analysis of the trend of procalictonin levels in post cardiac surgery patients

### S Sudarsanan, A Pattath, A Omar, P Sivadasan, S Aboulnaga, F Hamwi, A Al Khulaifi

#### Heart Hospital, Hamad Medical Corporation, Doha, Qatar

**Introduction:** The diagnostic utility of procacitonin (PCT) in cardiac surgery remains controversial [1] where the systemic inflammatory response (SIRS) induced by the cardiopulmonary bypass is claimed to be associated with elevated levels of PCT [2]. We aim to find a correlation between the level of PCT and the yield of positive blood culture in post operative fever in patients with intensive care unit (ICU) stay more than 24 hours post cardiac surgery.

**Methods:** Single center retrospective descriptive study over five years, enrolling patients who stayed for more than 24 hours post-operative, in the cardiothoracic ICU. PCT was assayed immunoluminometricaly prior to surgery and every 48 hours in response to onset of fever

**Results:** We screened 501 patients, of which 119 were enrolled in our study.Patients were divided into two groups according to the presence (Group 1), or absence of positive culture (Group 2).The mean PCT was significantly higher in Group 1 (19.0±4.6 versus 9.9±2.7, p=.033). Moreover, patients in Group 1 were associated with prolonged ICU stay,hospital stay and length of mechanical ventilation (p=0.00, 0.00, and 0.01 respectively)

**Conclusions:** The results showed that post cardiac surgery bacterial infections were associated with rise of PCT in contrast with patients who develop SIRS. The outcome measures were significantly worse in the culture positive group.

**References**

1. Boeken U, et al. Cardiovasc Surg 8(7):550-4, 2000.

2. Prat C, et al. J Cardiac Surg 23(6):627-32, 2008

## P030 Prognostic value of procalcitonin, pro-adrenomedullin, copeptin and atrial natriuretic peptide as predictors of duration of artificial lung ventilation and length of stay in intensive care unit for newborns and children of the first year of life after cardiac surgery with cardiopulmonary bypass

### A. Khrustalev, D. Popov, O. Stepanicheva

#### Bakoulev Scientific Center for Cardiovascular Surgery, Moscow, Russia

**Introduction:** Due to sequalae in the postoperative period children of the first year of life may require prolonged artificial lung ventilation (ALV) and prolonged length of stay (LOS) in the intensive care unit (ICU) after cardiac surgery. Procalcitonin (PCT), pro-adrenomedullin (MR-proADM), copeptin (CT-proAVP) and atrial natriuretic peptide (MR-proANP) can be predictors of duration of ALV and LOS in ICU.

**Methods:** 42 patients aged 153 (44-252) days (4-360 days) underwent cardiac surgery with cardiopulmonary bypass for severe congenital heart disease. In the dynamics levels of PCT, MR-proADM, CT-proAVP and MR-proANP were measured before surgery and on the 1, 2, 3 and 6 days after the operation with the Kryptor compact plus analyzer. Data are presented as medians with interquartile range. The Mann-Whitney U-test was used to compare the data. Values of p <0.05 were statistically significant.

**Results:** 24 patients (57%) required ALV for more than 72 hours. In this group statistically significant higher levels of PCT, MR-proADM and MR-proANP were found throughout the period (Table 1). The level of CT-proAVP had increased to statistical significance since the 3 day after the operation. 23 patients were in the ICU for more than 168 hours. In this group statistically significant higher levels of PCT, MR-proADM were found throughout the whole period (Table 2). The higher level of MR-proANP was statistically significant on the 1st and 6th days after surgery, MR-proANP had a tendency of increasing values on 2nd and 3rd days. CT-proAVP increased to statistical significance since the 2nd day after the operation and persisted throughout the studied period.

**Conclusions:** PCT, MR-proADM and MR-proANP can be used as predictors of prolonged ALV for children of the first year of life after cardiac surgery with cardiopulmonary bypass. The level of CT-proAVP can be considered since the 3 day after surgery. PCT and MR-proADM may be used to predict the LOS in the ICU. MR-proANP and CT-proAVP can be considered since the 1 and 2 days after surgery respectively.

**Fig. 1 (abstract P030).**
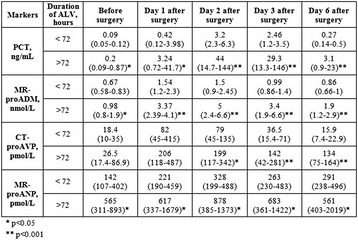
Duration of ALV

**Fig. 2 (abstract P030).**
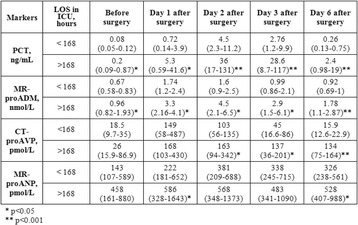
Length of stay in ICU

## P031 Association of inflammatory and hemostatic biomarkers with inflammasomes in septic patients at risk of developing coagulopathy

### D Hoppensteadt, R Green, A Walborn, G Wegrzyn, S Walborn, M Mosier, J Fareed

#### Loyola University Medical Center, Maywood, IL, USA

**Introduction:** Inflammasome contributes to the innate immune response identification of pattern recognition receptors (PRRS) on pathogens including bacterial and viruses. The purpose of this study is to quantitate inflammasome levels in defined sepsis associated patients and to determine its potential relevance to various biomarkers of hemostatic dysregulation.

**Methods:** Plasma samples from 52 adults with sepsis and suspected coagulopathy were analyzed. Fibrinogen was measured using a clot based method on ACL-ELITE coagulation analyzer. Cortisol, D-dimer, PAI-1, NLRP-3 inflammasomes, MP-TF, Fibronectin, and CD40L were measured using commercially available ELISA assays.

**Results:** When comparing patients with sepsis and suspected DIC to the normal plasma samples, there was a significant elevation in NLRP-3 inflammasome levels in the sepsis cohort (p = < 0.0001) (Fig. 1). The NLRP-3 inflammasome concentration in the sepsis cohort did not correlate with other biomarkers. An elevated level of NLRP-3 inflammasomes was significantly associated with an increased levels of PAI-1 (p < 0.0004) (Table 1).

**Conclusions:** The current study shows a significant relationship between inflammasomes and PAI-1 levels in patients with sepsis associated coagulopathy. The positive correlation between NLRP-3 inflammasomes and PAI-1 shows that the activation of inflammasomes may have a role in the upregulation of PAI-1.

**Table 1 (abstract P031).** Inflammatory and hemostatic biomarkers correlated with NLRP-3 inflammasome levels in patients with sepsis and suspected DIC

NLRP-3 Inflammasome Correlation	p (Mann-Whitney)	Spearman r
CD40L	0.5716	-0.08028
PAI-1	0.0041	0.3915
MP-TF	0.1491	0.2528
Fibrinogen	0.7685	0.04314
Fibronectin	0.8291	-0.03067
Cortisol	0.0758	0.2484
D-Dimer	0.3272	0.1495

**Fig. 1 (abstract P031).**
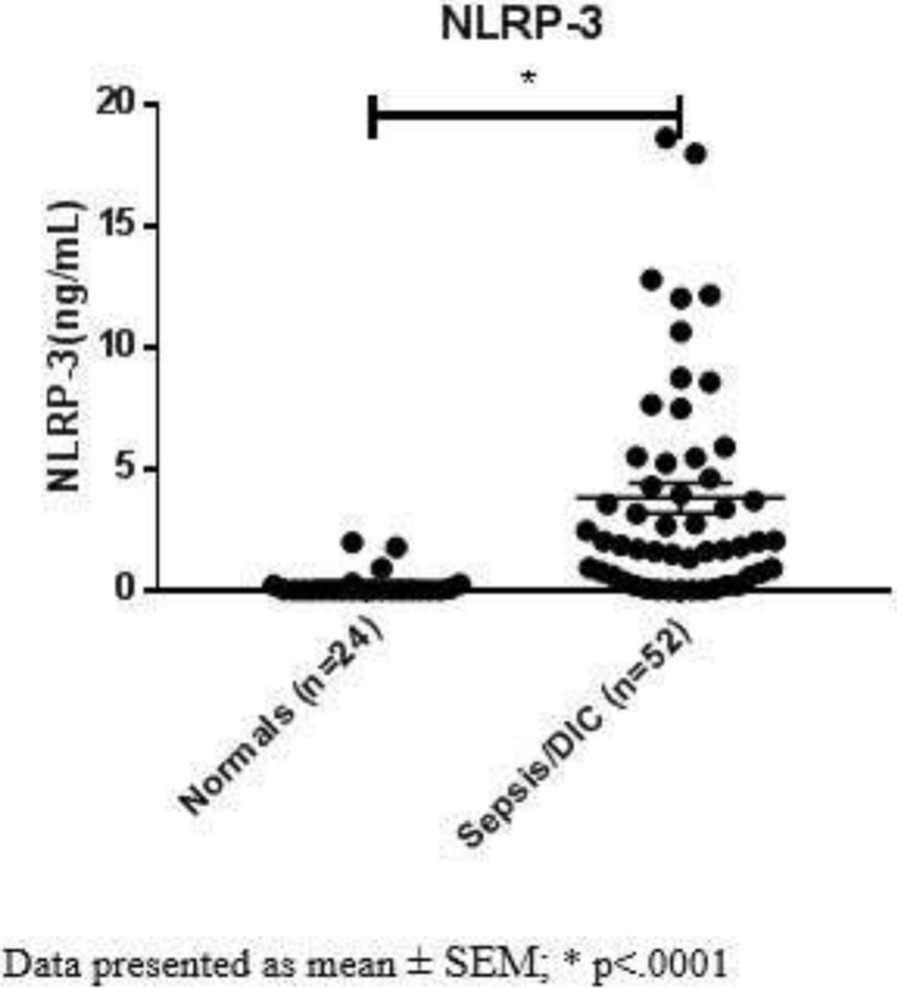
NLRP-3 inflammasomes in patients with sepsis and suspected DIC on Day 0 (n=52) compared to normal healthy controls (n=24)

## P032 Heparin-binding protein (HBP) as an index of pro-inflammation in sepsis

### T Gkavogianni, E Tzouveli, D Benas, G Papagiannopoulou, S Grigoropoulou, A Spanos, E Giamarellos-Bourboulis

#### ATTIKON University Hospital, Athens, Greece

**Introduction:** Easily measurable biomarkers to indicate the state of immune activation in sepsis remain an unmet need. HBP is secreted from neutrophils and it is increased in sepsis. However, its association with the innate immune function is poorly understood.

**Methods:** Plasma was isolated on three consecutive days from 30 patients with ventilator-associated pneumonia meeting the Sepsis-3 definitions. Monocytes were also isolated on day 1 and stimulated with lipopolysaccharide (LPS) for the production of tumour necrosis factor-alpha (TNFalpha). HBP, ferritin and TNFalpha were measured by an enzyme immunoassay. Over-time changes of HBP were associated with final outcome.

**Results:** A positive association was found between ferritin concentrations and circulating HBP on day 1 (rs: +0.371, p: 0.0002). The median value of HP on day 1 was 177 ng/ml. The stimulated production of TNFalpha in relation to the median HBP level is shown in Fig. 1. Among 20 survivors, mean change of HBP from the baseline was -12.2% +/- 20.2%; this was +146.8% +/- 89.4% among 10 non-survivors (p: 0.028). After ROC curve analysis it was found that more than 18% increase of HBP after 48 hours was associated with 81% specificity for 28-day mortality (odds ratio for unfavorable outcome 8.50; p: 0.017).

**Conclusions:** HBP seems to indicate patients who rely at the pro-inflammatory arm of sepsis since it correlates positively with ferritin and with the increased stimulated production of TNFα from circulating monocytes. Increases the first 48 hours by more than 18% indicate progression towards unfavorable outcome.

**Fig. 1 (abstract P032).**
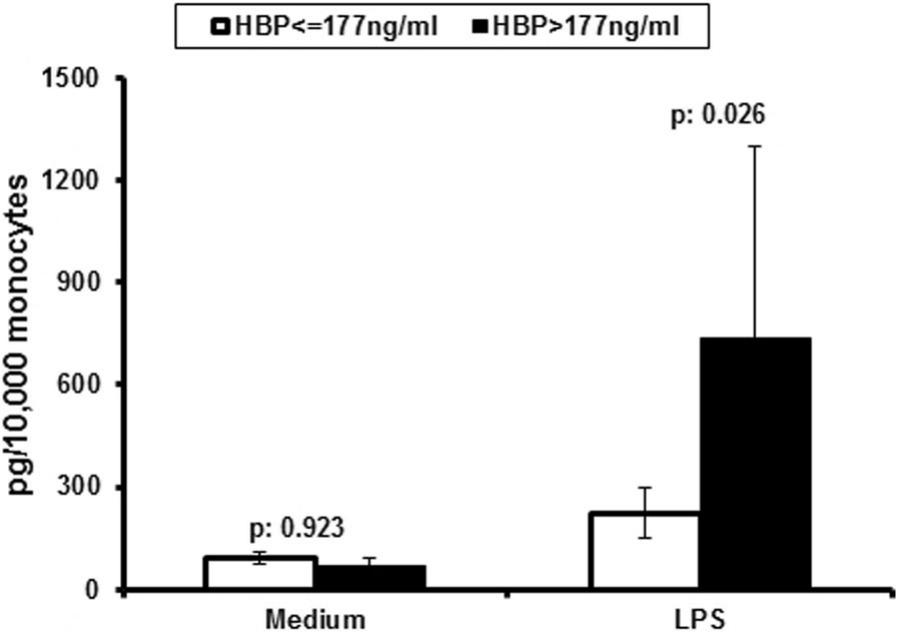
See text for description

## P033 Integration of heparin-binding protein (HBP) and one sign of quick SOFA score (qSOFA) to predict 30-day outcome.

### E Kyriazopoulou, C Psarrakis, C Moschopoulos, I Christou, P Fragkou, T Marantos, E Karofyllakis, K Roussakis, E Giamarellos-Bourboulis

#### Attikon University Hospital, Athens, Greece

**Introduction:** Early prediction of the risk of death among patients admitted at the Emergency Department (ED) remains an unmet need. The prognostic performance of HBP that is secreted by neutrophils was prospectively validated in a series of sequential ED admissions.

**Methods:** HBP and elements of qSOFA were analyzed prospectively in 310 serial ED admissions (main reasons for admission: acute abdominal pain 28.4%; fever 24.5%; vomiting/diarrhea 23.9%; dyspnea 22.3%; neurologic signs 11.3%; non-specific complaints 38.1%; most patients admitted for more than one reasons). Upon ED admission patients were scored as low-risk, intermediate-risk and high-risk at the discretion of the physician. HBP was measured in blood samples upon admission by an enzyme immunosorbent assay.

**Results:** HBP was significantly greater among patients who died very early (Fig. 1). In five out of six of patients dying early HBP was greater than 15 ng/ml. We combined HBP more than 15 ng/ml and the presence of one sign of qSOFA into a new score; this had 82.4% sensitivity to predict 30-day mortality. The respective sensitivity of two signs of qSOFA was 23.5% (p: 0.002). The use of this new score allowed better stratification of patients originally considered at the triage as low-risk into high-risk (Fig. 2).

**Conclusions:** We propose HBP more than 15 ng/ml and one qSOFA sign as an early score for 30-day mortality at the ED.

**Fig. 1 (abstract P033).**
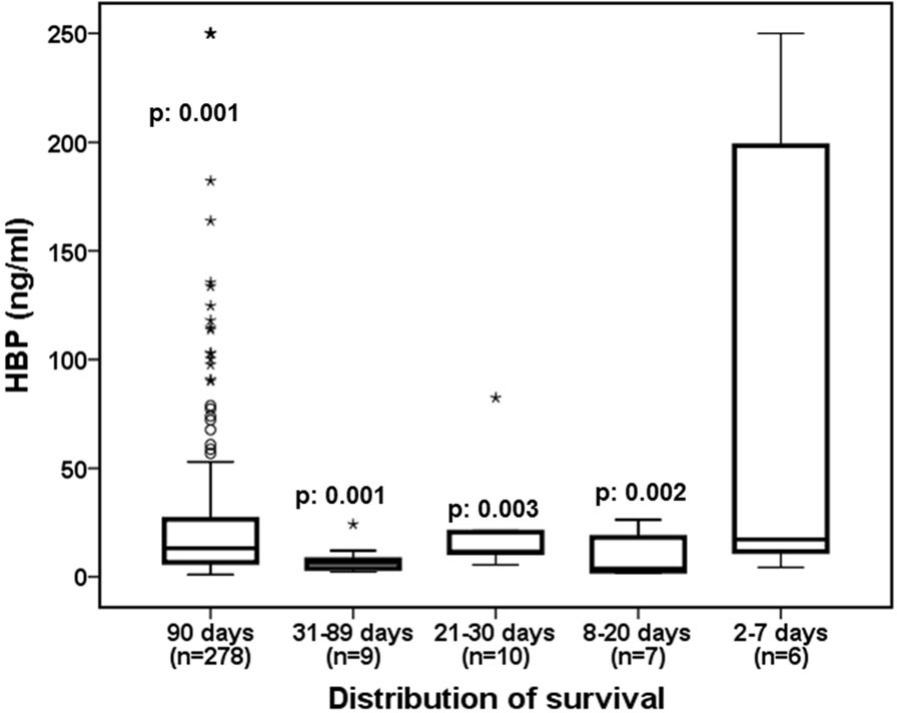
See text for description

**Fig. 2 (abstract P033).**
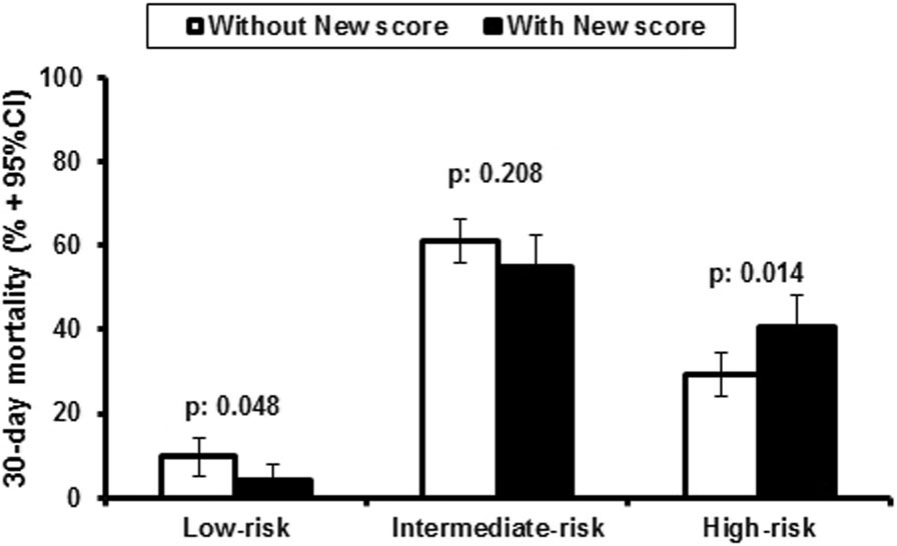
See text for description

## P034 Usefulness of heparin binding protein in early diagnosis of septic shock

### C Balci, E Haftaci, B Koyun

#### Health Sciences University, Kocaeli Derince Traning Hospital, Kocaeli, Turkey

**Introduction:** Despite of our growing knowledge in pathophysiology of septic shock still remain one of the most important factors of hospital mortality. It is thought that early diagnosis and treatment at early stage of septic shock would decrease its mortality. There have been on-going studies in recent years which research the usability of Heparin Binding Protein (HBP) in early diagnosis of sepsis [1]. To seek the usability of C- reactive protein (C-RP), procalcitonin (PCT) and HBP biomarker combination in early diagnosis of septic shock.

**Methods:** 30 patients, who have the diagnosis of septic shock, that are expected to stay in intensive care unit more than 24 hours, and aged between 22-75 are included in the study. Data are collected from the patients’ blood samples that are drawn on admission, on the 24th hour, and on the day of discharge or death.

**Results:** It has been found in our study that, best “cut-off” value 124 ng/mL, specificity 0.82 and sensitivity 0.77 for HBP. Compared with other biomarkers, HBP was the best predictor of progression to organ dysfunction (area under the receiver operating characteristic curve (AUC) = 0.801).

**Conclusions:** Although there have been many biomarkers for early diagnose of septic shock, C-RP and PCT are the most common used markers in nowadays’ clinical practice. The usability of HBP in early diagnosis of sepsis is still being researched. We concluded that PCT, C-RP and HBP biomarker combination is usable to diagnose septic shock at the end of our study.

**Reference**

1. Holub M, Beran O. Crit Care 16(3):133, 2012.

## P035 Change of ADAMTS-13 during sepsis is associated with outcome

### I Vasileiadis^1^, M Politou^2^, N Rovina^1^, S Dimopoulos^2^, E Tripodaki^3^, A Kyriakoudi^1^, E Ntouka^1^, E Stavrou^1^, A Koutsoukou^1^

#### ^1^Sotiria Hospital, National and Kapodistrian University of Athens, Athens, Greece,^2^Onasseio Cardiac Surgery Center, Athens, Greece,^3^Agios Savvas Regional Cancer Hospital, Athens, Greece

**Introduction:** Reduced ADAMTS-13 and increased von Willebrand Factor (vWF)/ADAMTS-13 ratio have been observed in sepsis and are associated with the severity of the disease [1,2]. However, their change during the septic episode and in the event of a change in the clinical status of the septic patients has not been investigated. The aim of the study was to assess the variation of these hemostatic parameters in critically ill patients during the course of a septic episode.

**Methods:** We monitored 34 septic patients admitted in the Intensive Care Unit (ICU). 23 improved (group A) while 11 deteriorated (group B). We assessed vWF, ADAMTS-13 and the vWF/ADAMTS-13 ratio on admission in ICU (time point 0) and at the time of a change in patients’ clinical condition (remission or deterioration, time point 1).

**Results:** In group A, ADAMTS-13 and the vWF/ADAMTS-13 ratio did not significantly change (567.0±296.0 vs 670.7±534.5 ng/ml, p=0.238 and 0.709±0.588 vs 0.876±0.687, p=0.34 respectively) while vWF increased (326.2±122.7 vs 407.0±157.6 % of norm., p=0.028) at time point 1 compared to time point 0. In group B, ADAMTS-13 decreased (831.4±586.1 vs 482.0±277.8 ng/ml, p=0.026) while vWF and the vWF/ADAMTS-13 ratio increased (389.5±170.5 vs 525.3±141.0 % of norm., p=0.02 and 0.779±0.851 vs 1.490±1.060, p=0.002) at time point 1 compared to time point 0. There was a non-statistical greater increase (% change) of vWF (53±63 versus 35±63%, p=0.4) in group B patients compared to group A patients. ADAMTS-13 percentage difference (>or<= 22%) was associated with sepsis outcome (χ =8.7; HR:5.86; 95% CI:1.6-22.1; p=0.009).

**Conclusions:** Hemostatic disorders, as assessed by vWF and ADAMTS-13 levels were detected in septic patients, while their changes differed according to the evolution of the septic episode. ADAMTS-13 changes may be associated with outcome.

**References**

1. Fukushima H et al. Shock 39(5):409-14, 2013.

2. Azfar MF et al. Clin Invest Med 40(2):E49-E58, 2017.

## P036 Integration of biomarkers and clinical signs for the early diagnosis of sepsis

### L Lazaridis^1^, S Karatzas^2^, A Prekates^3^, K Toutouzas^2^, C Mathas^4^, J Popp^5^, J Olsen^6^, E Giamarellos-Bourboulis^1^

#### ^1^Attikon University Hospital, Athens, Greece,^2^Ippokrateion General Hospital, Athens, Greece,^3^Tzaneion Hospital, Piraeus, Greece,^4^Aghia Olga Hospital, Athens, Greece,^5^Leibniz Instiute for Photonic Technology, Jena, Germany,^6^Virogates SA, Copenhagen, Denmark

**Introduction:** The Sepsis-3 Task Force has introduced quick sequential organ failure assessment (qSOFA) as a diagnostic tool for the early diagnosis of sepsis. However, the Sepsis-3 criteria and qSOFA have not yet been prospectively validated. INTELLIGENCE-1 (ClinicalTrials.gov NCT03306186) is aiming in this prospective validation through the integration of clinical signs and biomarkers.

**Methods:** 100 adult patients with at least one sign of qSOFA and infection or acute pancreatitis or after operation were prospectively followed-up. Blood was sampled the first 24 hours; those with HIV infection, neutropenia and multiple injuries were excluded. Sepsis was diagnosed using the Sepsis-3 criteria. Soluble urokinase plasminogen activator receptor (suPAR) was measured by an enzyme immunoassay.

**Results:** Sixty patients were classified with sepsis using the Sepsis-3 definitions. Presence of at least two signs of qSOFA had 56.7% sensitivity, 95.0% specificity, 92.8% positive predictive value and 38.0% negative predictive value for the diagnosis of sepsis. The integration of qSOFA signs and suPAR improved the diagnostic performance (Fig. 1).

**Conclusions:** Conclusions Two signs of qSOFA have significant positive prognostic value for sepsis but low sensitivity. This is improved after integration with suPAR.

The INTELLIGENCE-1 study is supported by the European Commission through the Seventh Framework Programme (FP7) HemoSpec.

**Fig. 1 (abstract P036).**
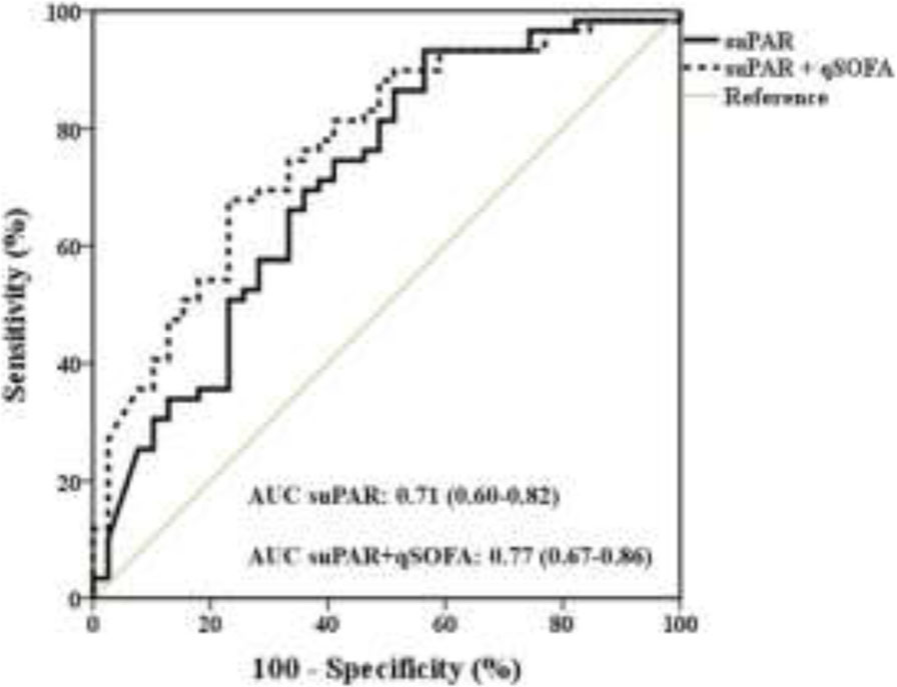
See text for description

## P037 TRIAGE Study protocol: Assessment of biomarkers to predict clinical worsening of patients with sepsis admitted in the Emergency Department

### T Lafon^1^, C Vallejo^1^, L Barbier^2^, MA Cazalis^2^, T Daix^1^, A Desachy^1^, V Gissot^3^, PF Laterre^4^, K Tazarourte^5^, B François^1^

#### ^1^Centre Hospitalier Universitaire Dupuytren, Limoges, France,^2^bioMérieux SA, Marcy l’Etoile, France,^3^CHU de Tours/Université François Rabelais, Tours, France,^4^Cliniqies Saint-Luc, Brussels, Belgium,^5^Groupement Hospitalier Edouard Herriot - Hospices Civils de Lyon, Lyon, France

**Introduction:** Sepsis is a frequent reason for admission in the Emergency Department (ED) and its prognostic mainly relies on early diagnosis. In addition, no validated prognostic tool is currently available. Therefore, identification of patients at high risk of worsening in the ED is key. The TRIAGE objective was to assess the prognostic value of a blood marker panel to predict early clinical worsening of patients admitted in the ED with suspected sepsis.

**Methods:** TRIAGE was a prospective, multicenter (11 sites in France and Belgium) study on biological samples conducted in partnership with bioMerieux S.A. Patients admitted in the ED with suspected or confirmed community-acquired infection for less than 72h were included. Exclusion criteria were: admission in the ED for more than 12 hours, septic shock at admission, immunodepression, sepsis syndrome 30 days prior to admission. The protocol included 5 clinical and biological time points (H0, H6, H24, H72, D28). Patients were classified in 3 groups at admission (infection, sepsis, severe sepsis) and divided into 2 evolution/prognosis groups depending on worsening or not from their initial condition to severe sepsis or septic shock and SOFA score’s evolution. The evolution criteria were centrally evaluated by an independent adjudication committee of sepsis experts including emergency physicians and intensivists. Patients were followed up to day 28 for mortality.

**Results:** The study duration was 3 years with 600 patients included (102 excluded). The centralized analysis is in progress to select the combination of biomarkers with the best prognostic performance comparing both evolution/prognosis groups. Currently, 125 patients have been classified as worsening and some results will be available in 2018.

**Conclusions:** TRIAGE is the largest prospective multicenter study assessing the prognostic value of a panel of blood markers in EDs which could help identification of septic patient at risk of worsening at time of admission in the ED and develop specific management.

## P038 Immune profiling of host response biomarkers through transcriptomic analysis using the FilmArray® system.

### DM Tawfik^1^, L Ganee^1^, A Bocquet^1^, V Moucadel^1^, J Textoris^1^, T Rimmele^2^, G Monneret ^2^, S Blein ^1^, M Rol ^3^, J Montgomery^4^, F Mallet^1^, A Pachot ^1^, J Yugueros Marcos ^1^,.REALISM Study group^5^

#### ^1^bioMerieux, Lyon, France,^2^Hospices Civils de Lyon, Lyon, France,^3^Bioaster Technology Research Institute, Lyon, France,^4^BioFire Diagnostics LLC Salt Lake City, UT, USA,^5^REALISM Study group, Lyon, France

**Introduction:** Immune status characterization in Intensive Care Unit (ICU) patients presents a major challenge due to the heterogeneity of response. In this study, the FilmArray® system was used with customized gene assays to assess the immune profile of critically-ill ICU patients compared to healthy volunteers; from within the REALISM cohort.

**Methods:** A customized FilmArray® pouch containing 24 assays was designed; 16 target and 8 reference genes. Detection and semi-quantification of assays from whole blood collected in PAXgene tubes occurs in the device within 1 hour. A total of 20 subjects from the REALISM cohort were tested in duplicates: 1 trauma, 5 septic shock and 5 surgery patients, along with 9 healthy volunteers. The patients’ selection was based on HLA-DR expression on monocytes, and PHA-(Phytohaemagglutinin) stimulated T-cell proliferation assay, to have various immune profiles.

**Results:** Quantification cycle values of the target genes were normalized by the geometrical mean of reference genes to account for the different cell counts among specimens. The number of the CD3+ cells and HLA-DR, determined by flow cytometry, showed good correlation to CD3D and CD74 gene expression, respectively. Seven genes showed significant differences in expression levels between the healthy volunteers and patient groups: CD3D, CD74, CTLA4 & CX3CR1 were down-regulated, while IL-10, IL1RN and S100A9 were up-regulated in the patient populations. The use of relative quantitative difference of some markers was able to distinguish and emphasize the variability between the patient groups while homogenizing the discrepancy among healthy volunteers.

**Conclusions:** The FilmArray® system was shown to allow host transcriptomics analysis of immune-relevant genes directly from PAXgene tubes, in only one hour. These results show great potential for the development of a fully automated immune profiling tool, enabling close monitoring of critically-ill patients.

## P039 Rapid biophysical analysis of host immune cells enables diagnosis of sepsis in the emergency department

### M Macdonald^1^, R Sheybani^1^, A DeWitt^2^, S Brierre^3^, T Caffery^3^, T Jagneaux^3^, C Thomas^3^, D Di Carlo^4^, H Tse^1^, A Shah^1^, H O’Neal^3^

#### ^1^CytoVale, San Francisco, CA, USA,^2^Baton Rouge General Medical Center, Baton Rouge, LA, USA,^3^Louisiana State University Health Sciences Center, Baton Rouge, LA, USA,^4^University of California, Los Angeles, CA, USA

**Introduction:** Early, rapid diagnosis is integral to the efficient effective treatment of sepsis; however, there is no gold standard for diagnosis, and biochemical surrogates are of limited and controversial utility. The CytoVale system measures biophysical properties of cells by imaging thousands of single cells per second as they are hydrodynamically stretched in a microfluidic channel. This platform has been shown to measure dozens of mechanical, morphological, and cell surface biomarkers of WBC activation simultaneously [1,2]. In this study, we show the performance of the CytoVale system in measuring biophysical markers for sepsis detection in the emergency department (ED).

**Methods:** We conducted an IRB-approved prospective cohort study of Emergency Department (ED) patients with 2+ SIRS criteria and evidence of organ dysfunction. 307 patients were included for analysis. Blood samples for the Cytovale assay were collected in the ED, and the diagnosis of sepsis was adjudicated by blinded clinician review of the medical record. Captured imaging data were analyzed using computer vision to quantify mechanical parameters per cell, and a logistic model was trained to discriminate patients who had sepsis from those who did not.

**Results:** We found substantial biophysical differences between cells from septic and non-septic patients as observed at both the single cell level (Fig. 1) and when looking at the overall leukocyte populations (Fig. 2). A multiparameter classification algorithm to discriminate septic from non-septic patients based on biophysical markers currently yields a sensitivity of 88% with a negative predictive value of 95%.

**Conclusions:** In patients presenting to the ED with 2 of 4 SIRS criteria and evidence of organ dysfunction, the CytoVale system provides a potentially viable means for the early diagnosis of sepsis via the quantification of biophysical properties of leukocytes.

**References**

1. Gossett DR et al. PNAS 20:7630–5, 2012

2. Crawford K et al. AJRCCM, under review, 2017

**Fig. 1 (abstract P039).**
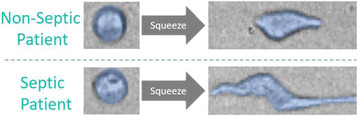
Mechanical phenotyping reveals new biophysical markers of WBC activation

**Fig. 2 (abstract P039).**
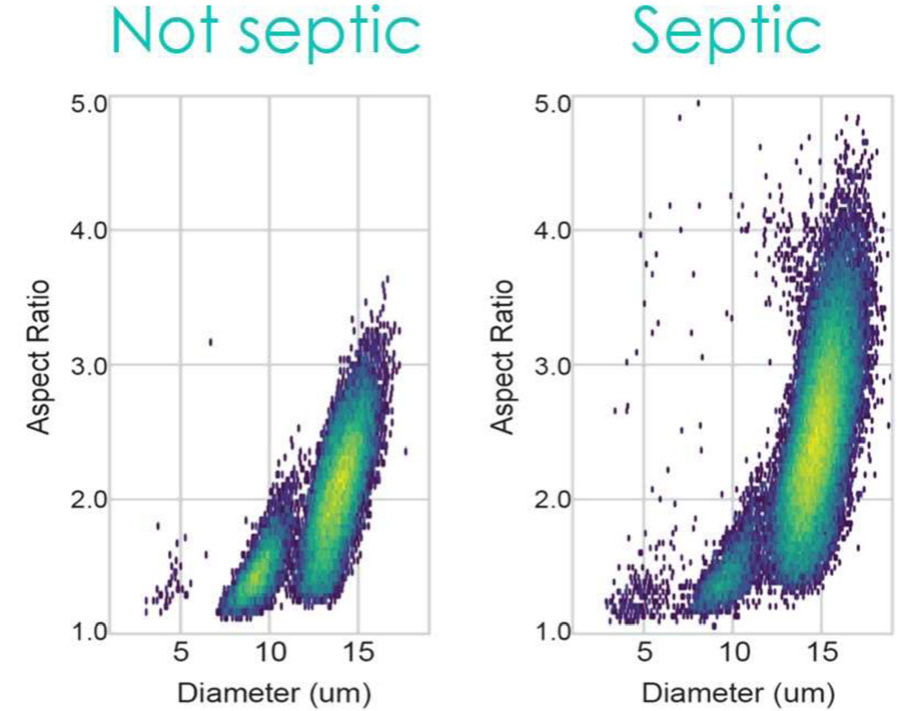
Performance of two biophysical markers (diameter and aspect ratio) in discriminating non-septic and septic patients at the leukocyte population level

## P040 Oxidative stress and other biomarkers to predict the presence of sepsis in ICU patients

### V Tsolaki, M Karapetsa, G Ganeli, E Zakynthinos

#### ICU, Larissa, Greece

**Introduction:** Early identification of sepsis adds a survival benefit in ICU patients. Several biomarkers have been evaluated, yet an optimal marker is still lacking [1].

**Methods:** We prospectively determined oxidative status in patients admitted in a general Intensive Care Unit of the University Hospital of Larisa. Oxidative status was determined measuring the novel static (sORP) and capacity (cORP) oxidation-reduction potential markers. Other biomarkers (BNP, presepsin, CRP) were measured, and the discriminative properties for the detection of sepsis were evaluated.

**Results:** Oxidative status was evaluated in a hundred and fifty two consecutive patients. Patients with severe sepsis and septic shock had significantly higher sORP values than patients without sepsis (173.31± 20.44 vs 164.11±18.78, p=0.006), while cORP did not differ (0.34±0.31 vs 0.37±0.20, ns). Patients with cerebral damage had the lowest sORP on admission while surgical and medical patients had the highest sORP values (157.2 ±18.33 vs 174.04±18.1 respectively, p<0.001). sORP could predict the presence of sever sepsis (OR 1.107, p=0.009), along with presepsin (OR 1.002, p<0.0001), C-Reactive Protein values (OR 1.161, p=0.013) and Brain Natriuretic Peptide (OR 1.001, p=0.046). The best discriminating properties had presepsin (AUC 0.893, p<0.0001) and CRP (AUC 0.743 p<0.0001). The presence of a microorganism in blood or bronchial secretions could be predicted from the values sORP (AUC 0.633, p=0.042) and CRP (AUC 0.653, p=0.02).

**Conclusions:** Oxidative status differs between patients admitted in the ICU and could serve as a prognostic marker for the presence of sepsis.

**Reference**

1. Singer M, et al. JAMA 315(8):801-810, 2016

## P041 Relationship between pre-operative C-reactive protein elevation and major cardiovascular events after vascular surgery

### I Ben Naoui, A El Ghali, AG Ammous, I Nefzi, A Saidi, A Ammous, A Cherif

#### La Rabta Hospital, Tunis, Tunisia

**Introduction:** C-reactive protein (CRP), is reported to be an effective marker for the assessment of vascular inflammation activity and acute coronary events prediction [1].We hypothesized that preoperative CRP elevation is related to the occurrence of postoperative adverse cardiovascular outcomes.

**Methods:** We prospectively included patients scheduled to undergo different vascular surgeries from december 2016 to september 2017. we assessed demographic data, comorbidities, revised cardiac risk index (RCRI) and biomarkers (CRP, cardiac troponin high sensitive Ths, creatinine and urea) in the preoperative period. we also noted type and duration of surgery, intraoperative blood loss, ICU stay and mortality. we evaluated CRP as a predictive marker of major cardiovascular events defined as chest pain, Ths elevation, electrocardiogram changes, arrhythmia, pulmonary embolism, stroke occuring within postoperative 3 months.

**Results:** During our study, 30 patients were scheduled to undergo vascular surgeries. From the 30 patients, 66% developed adverse cardiac events (Table 1). We showed the predictive value of CRP in major cardiovascular event in a ROC analysis (Fig. 1). The cuttoff value of CPR was 54 giving 85% of sensitivity and 82% of specificity.

**Conclusions:** Our study pointed out that CRP preoperative elevation could have a very strong predictive value of post-operative cardiovascular events in vascular surgery, this is in line with results showed by previous studies [1].

**Reference**

1. Yunxiang Li. Int J Clin Exp Pathol 9:11904-11910, 2016

**Table 1 (abstract P041).** Major cardiovascular events immediately after surgery

Cardio-vascular complications	percentage
Chest pain	2 (6.6%)
Ths elevation	13 (43.3%)
Electrocardiogram changes	1(3.3%)
Arrhythmia	2(6.6%)
Pulmonary embolism	1(3.3%)
Stroke	1(3.3%)

**Fig. 1 (abstract P041).**
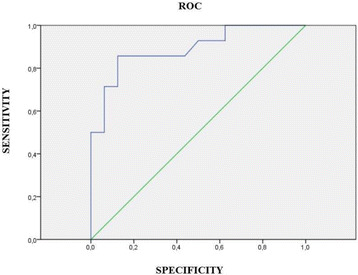
The area under the curve for CRP elevation is 0.891

## P042 Impact of age in critically ill infected patients: a post-hoc analysis of the INFAUCI study

### S Moreira^1^, J Baptista^1^, F Froes^2^, J Pereira^3^, J Gonçalves-Pereira^4^, C Dias^5^, J Paiva^3^

#### ^1^Centro Hospitalar e Universitário de Coimbra, Coimbra, Portugal,^2^Hospital Pulido Valente, Centro Hospitalar Lisboa Norte, Lisboa, Portugal,^3^Centro Hospitalar São João, Porto, Portugal,^4^Hospital Vila Franca de Xira, Vila Franca de Xira, Portugal,^5^Centro de Investigação em Tecnologias e em Serviços de Saúde, Porto, Portugal

**Introduction:** Elderly are particularly susceptible to bacterial infections and sepsis, and they comprise an increasing proportion of intensive care unit (ICU) admissions. Our aim was to evaluate the impact of age on critically ill infected patients.

**Methods:** We performed a post-hoc analysis of all infected patients admitted to ICU enrolled in a 1-year prospective, observational, multicenter study involving 14 ICUs. Patients aged <65, 65-74 and >=75 years were compared (group A, B, and C). Multidrug-resistance (MDR) was defined as acquired non-susceptibility to at least one agent within three or more antimicrobial categories.

**Results:** Of the 3766 patients analyzed, 1652 (43.9%) were infected on ICU admission. Of these, 828 (50%) belonged to group A, 434 (23%) to group B and 440 (27%) to group C. Group C were more dependent, had higher SAPS II and Charlson scores (p<0.05). ICU and hospital length of stay did not differ between groups. Microorganism isolation and bacteremia were higher in group B (53% and 24%, respectively) than groups A (45% and 19%, respectively) and C (47% and 17%, respectively; p<0.05). Septic shock was present in 58% of patients and was more frequent in groups B (55%) and C (55%) than group A (48%). The most common sources of infections were respiratory and intra-abdominal. Isolation of gram-negative bacteria was significantly increased in group B and C (p=0.034). The most common isolated bacteria were *Escherichia coli* (17%), *Staphylococcus aureus* (15%) and *Pseudomonas aeruginosa* (8%) for all groups. In total, 151 isolates (22%) corresponded to MDR bacteria, of which 57% were *Staphylococcus aureus*. Age was not a risk factor for infection by MDR. All-cause mortality in ICU and hospital was: 23% and 30%; 29% and 40%; 36% and 53% - respectively for groups A, B, and C (p < 0.001).

**Conclusions:** Old patients (65-74 years) were more prone to present with bacteremia, which could account for the increased severity of sepsis and higher all-cause mortality. Age was not a risk factor for MDR infection.

## P043 Review of rejected microbiology specimens from intensive care

### AR Garg^1^, E Sherry^1^, L Verrinder^1^, JM Patel^2^

#### ^1^University Hospital Birmingham, Birmingham, UK,^2^University of Birmingham, Birmingham, UK

**Introduction:** The rapid identification of pathogens using patient samples is crucial. Delays in this can potentially have serious implications for patients and infection prevention/control [1]. The aim of this project was to identify the number of microbiology samples sent, the number rejected and reasons for rejection, with the intention to reduce such instances.

**Methods:** Data was collected retrospectively on ICU admissions from January-June 2017 to a university hospital in the UK. Patients were identified and data collected using the Intensive Care National Audit and Research Centre (ICNARC) database and from electronic patient records. Data collected included: demographics, length of stay, microbiology samples sent and details on the rejected samples.

**Results:** 530 patients were identified with a total of 4725 (median: 4 samples/patient) samples sent to microbiology. 144 were rejected (3%). 100 (18%) patients had at least 1 sample rejected. The median number of samples rejected per patient was 1 (Range: 1-10). The most common samples rejected were urine (22%), blood (20%), faeces (19%) and sputum (8%). 69 (48%) of the samples were resent for testing (median 1 day; range 0-20). Reasons for sample rejection are shown in Table 1. Most rejections occurred within 48-hours of admission (Fig. 1).

**Conclusions:** This study confirms a high number of samples are sent to microbiology. Although a few are rejected, overall this represents a large number, with most occurring during the first days of admission. Reasons for sample rejection are remedial through improved training and vigilance. A bespoke guide to sample collection for microbiology coupled with a training program for healthcare professionals has been introduced with the aim to reduce sample rejections from 3% to 0.5%.

**Reference**

1. Vincent JL et al. Crit Care Med 43:2283-2291, 2015

**Table 1 (abstract P043).** Reasons for sample rejection

Reason for rejection	n
Insufficient sample	46/143 (32%)
Wrong sample type	37/143 (25%)
Patient Identification error	24/143 (16%)
Incorrect info on form	23/143 (16%)
Incorrect timing	9/143 (6%)
Others (Wrong lab, Test unavailable)	2/143

**Fig. 1 (abstract P043).**
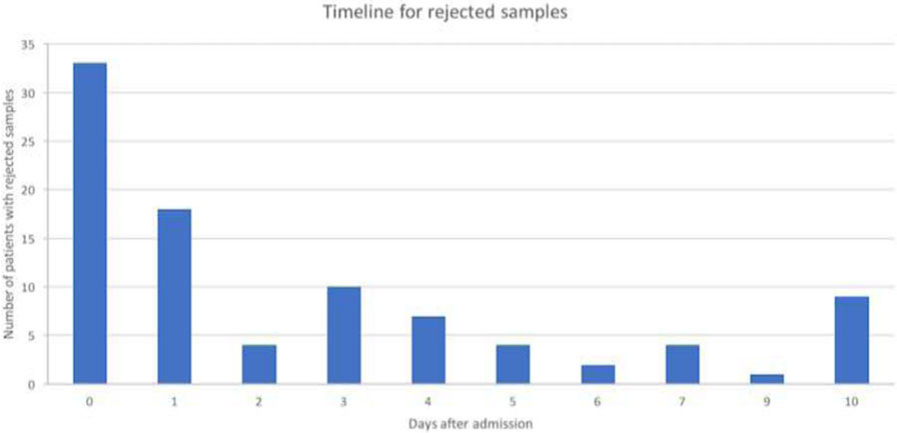
Timeline for rejection of samples

## P044 Microbiological colonization of ICU healthcare workers’ mobile phones

### A Galazzi^1^, E Broggi^1^, A Grancini^1^, M Panigada^1^, I Zainaghi^1^, F Binda^1^, T Mauri^2^, G Grasselli^2^, I Adamini^1^, A Pesenti^2^

#### ^1^Fondazione IRCCS Ca’ Granda Ospedale Maggiore Policlinico, University of Milan, Milano, Italy,^2^Fondazione IRCCS Ca’ Granda Ospedale Maggiore Policlinico, Dipartimento di Fisiopatologia Medico-Chirurgica e dei Trapianti, University of Milan, Milan, Italy

**Introduction:** Careful hand hygiene of health-care workers (HCWs) is recommended to reduce transmission of pathogenic microorganisms to patients [1]. Mobile phones are commonly used during work shifts and may act as vehicles of pathogens [2,3]. The purpose of this study was to assess the colonization rate of ICU HCWs’ mobile phones before and after work shifts.

**Methods:** Prospective observational study conducted in an academic, tertiary-level ICU. HCWs (including medical and nursing staff) had their mobile phones sampled for microbiology before and after work shifts on 6 different days. Samples were taken with eSwab in a standardized modality and seeded on Columbia Agar plus 5% sheep blood. A semiquantitative growth evaluation was performed at 24 and 48 hours after incubation at 35°C.

**Results:** Fifty HCWs participated in the study (91% of department staff). One hundred swabs were taken from 50 mobile phones. Forty-three HCWs (86%) reported a habitual use of their phones during the work shift, and 38 of them (88.4%) usually kept their mobiles in the uniform pocket. All phones (100%) were positive for bacteria. The most frequently isolated bacteria were Coagulase Negative Staphylococcus, Bacillus sp. and MRSA (97%, 56%, 17%, respectively). No patient admitted to the ICU during the study period was positive for bacteria found of HCWs’ mobile phones. No difference in bacteria types and burden was found between the beginning and the end of work shifts.

**Conclusions:** HCWs’ mobile phones are always colonized mainly by flora resident on HCW’s hands, even before the work shift and irrespective of the microbiological patients’ flora. Further studies are warranted to investigate the role of mobile phones’ bacterial colonization in the ICU setting and to determine whether routine cleaning of HCWs’ mobile phones may reduce the rate of infection transmission in critical patients.

**Reference**

1. WHO Guidelines on Hand Hygiene in Health Care. 2009

2. Ulger F et al. Ann Clin Microbiol Antimicrob 8:7, 2009

3. Russotto V et al. J Intensive Care 3:54, 2015

## P045 Microbiological contamination of mobile phones carried by health care workers in intensive care units and operating room

### P Mogrovejo, S Castro, V Arízaga, E Guerrero, A Loja, L Tamayo, H Aguirre

#### Santa Inés Hospital, Cuenca, Ecuador

**Introduction:** Mobile phones (MP) of health care workers (HCWs) could be colonized by pathogenic bacteria. It can be a vector of drug resistant bacteria and could increase nosocomial infections. The aim of this study is to evaluate the prevalence of MP bacterial colonization in the adult intensive care unit (AICU), pediatric intensive care unit (PICU) and operating room (OR) in a tertiary level hospital.

**Methods:** Sixty samples were collected from AICU (n=25), PICU (n=15) and OR (n=20) during August to September 2017. Samples were randomly selected and taken at the end of the HCWs duty with a sterile swab covering all MP surfaces. The inoculation was made into blood sheep and eosyn methilene blue agar for culture. Isolated bacteria were identified according to standard microbiological techniques. Antibiotic sensitivity testing was performed using disc diffusion method.

**Results:** Overall MP bacterial colonization rate was 95%. Main results are detailed in Table 1. Most common non pathogenic bacteria was Staphylococcus epidermidis n=18 (90%). Isolated pathogenic bacteria were Meticilin-susceptible Staphylococcus aureus n=14 (38%), Methicillin-resistant Staphylococcus aureus n=10 (27%), resistant Staphylococcus epidermidis n=4 (11%), Acinetobacter baumanni n=4 (11%), Klebsiella pneumoniae n=4 (11%) and resistant Acinetobacter baumanni n=1 (2%). No significant difference was found in colonization rates of pathogenic bacteria between MP with case n=35 (59%) versus MP without case n=25 (41%) (p< .753).

**Conclusions:** We found high rates of MP colonization with pathogenic bacteria. An educational program is necessary to reduce the contamination and transmission of these high risk microorganisms.

**References**

**Table 1 (abstract P045).** Colonization rates per department and subtype of isolated bacteria

Department	Non pathogenic bacteria	Pathogenic bacteria	Pathogenic bacteria	Negative
		Non drug resistant	Drug resistant	
AICU	5 (20%)	8 (32%)	9 (36%)	3 (12%)
PICU	8 (54%)	4 (26%)	3 (20%)	-
OR	7 (35%)	10 (50%)	3 (15%)	-
Total	20 (33%)	22 (37%)	15 (25%)	3 (5%)

## P046 Assessment of the variability of airborne contamination levels in an intensive care unit over a 24 hour period

### M Booth^1^, L Dougall^2^, E Khoo^3^, H Hood^3^, S MacGregor^2^, M Maclean^2^

#### ^1^Glasgow Royal Infirmary, Glasgow, UK,^2^University of Strathclyde, Glasgow, UK,^3^University of Glasgow, Glasgow, UK

**Introduction:** The objective of this study was to evaluate the variability in the dynamics and levels of airborne contamination within a hospital Intensive Care Unit in order to establish an improved understanding of the extent to which airborne bioburden contributes to cross-infection of patients. Microorganisms from the respiratory tract or skin can become airborne by coughing, sneezing and periods of increased activity such as bed changes and staff rounds. Current knowledge of the clinical microflora is limited however it is estimated that 10-33% of nosocomial infections are transmitted via air.

**Methods:** Environmental air monitoring was conducted in Glasgow Royal Infirmary ICU, in the open ward and in patient isolation rooms. A sieve impactor air sampler was used to collect 500 L air samples every 15 minutes over 10 hour (08:00-18:00 h) and 24 hour (08:00-08:00 h) periods. Samples were collected, room activity logged and the bacterial contamination levels were recorded as CFU/m^3^ of air.

**Results:** A high degree of variability in levels of airborne contamination was observed over the course of a 10 hour day and a 24 period in a hospital ICU. Counts ranged from 12-510 CFU/m^3^ over 24 hours in an isolation room occupied for 10 days by a patient with C. difficile infection. Contamination levels were found to be lowest during the night and in unoccupied rooms, with an average value of 20 CFU/m^3^. Peaks in airborne contamination showed a direct relation to increased room activity.

**Conclusions:** This study demonstrates the degree of airborne contamination that can occur in an ICU over a 24 hour period. Numerous factors were found to contribute to microbial air contamination and consideration should be given to potential improved infection control strategies and decontamination technologies which could be deployed within the clinical environment to reduce the airborne contamination levels, with the ultimate aim of reducing healthcare-associated infections from environmental sources.

## P047 New practice of fixing the venous catheter of the jugular on the thorax and its impact on the infection

### F Goldstein, C Carius, A Coscia

#### QuintaD’or, Rio de Janeiro, Brazil

**Introduction:** Central Line-associated Bloodstream Infection (CLABSI) is an important concern in the ICU, mainly in those with a high density of use of central venous catheter. Any measures that may have an impact on the reduction of CLABSI are important in reducing morbidity and mortality of hospitalized patients. Therefore we present a retrospective study comparing the fixation site (neck vs. thorax) of the catheters implanted in the jugular vein, guided by ultrasonography and evaluating its impact on the incidence of CLABSI. The purpose of our study was to identify if there is any positive impact on the reduction of CLABSI when the catheter is fixated on the thorax.

**Methods:** A retrospective unicentric study comparing the infection rates between the year of 2012, when the traditional technique of catheter fixation on the neck was used, and 2015, when 100% of the catheters were fixated on the thoracic region. The criteria for CLABSI were defined by the Infection Commission of QuintaD`or Hospital and the data on CLABSI were provided by the same commission. During this period there were no changes in the team of our unit and the patient's profile was the same. No deep vein catheter impregnated with antibiotics were used in the patients included in the study. The comparison used Fisher ´s test as a tool. All the patients hospitalized in the intensive care unit with indication of the central venous catheter of short permanence in the internal jugular vein were included. Patients with the central venous catheter of short permanence in other topographies, patients with hemodialysis catheter or with PICC were excluded.

**Results:** During the year of 2012, 98 internal jugular vein catheters were installed in our unit using the traditional technique, fixing the catheter on the neck. In this period, 6 cases of CLABSI were detected. On the other hand, in the year of 2015, 127 internal jugular vein catheters were installed in the same unit, all of them, using the thorax as the point of fixation. Although the number of catheters installed this year was higher, there was no case of CLABSI. It appears that this position, provides a better fixation of the catheter, avoiding that the bandage gets uncovered.

**Conclusions:** During the year of 2015, though there were more patients using deep vein catheters of short permanence, we had less CLABSI events on our unity compared to the year of 2012. Fisher's exact test identified a p-value of this association of 0.476. Fixation of the internal jugular vein catheter in the thorax seems to contribute to the prevention of CLABSI. Further prospective and randomized studies are required to evaluate the contribution of fixation of the jugular vein catheter in the thorax in the CLABSI prevention.

## P048 The Oral Biofilm Index in patients hospitalized on an intensive care unit

### R Marinho^1^, J Marinho^1^, A Marinho^1^, J Frias-Bulhosa^2^

#### ^1^Centro Hospitalar do Porto, Porto, Portugal,^2^Universidade Fernando Pessoa, Porto, Portugal

**Introduction:** The oral cavity of a patient who has been hospitalized presents a different flora from normal healthy people. After 48h hours of hospital stay, the flora presents a bigger number of microorganisms that can be responsible for secondary infections, like pneumonia, because of their growth and proliferation. The objective of our study was to assess the dental plaque index on patients on admission to an Intensive Care Unit, and reassess 7 days later, to evaluate the efficacy of oral hygiene.

**Methods:** Prospective, descriptive and observational study in an Intensive Care Unit of the CHP. Demographic, admission motive, hospital length of stay, feeding protocol, respiratory support need and oral hygiene protocol data was collected. The Greene & Vermillion Simplified Oral Hygiene Index (IHO-S) was used as the assessment tool on the first 24h and on 7th day.

**Results:** 74 patients were evaluated, 42 of which were excluded for not meeting the minimal dentition. 32 patients had a mean age of 60,53 ± 14,44 years, 53,1% were males and most of medical and surgical scope (37,5% each). Mean hospital length of stay was 15,69±6,69 days. The majority of patients were sedated (75%), under ventilator support (81,3%) and with enteric nutritional support, under nasogastric tube feeding. Initial IHO-S score was 0,67±0,45, rising to 1,04±0,51 (p<0,05) 7 days later.

**Conclusions:** Various studies have proven the importance of a good oral hygiene to avoid bacterial growth and reduce the risk for nosocomial infections. In this study, we’ve observed a significant worsening of oral hygiene one week after admission. Although this could be unimportant for a one week staying patient, it could indicate an increased risk for nosocomial infections for longer staying patients, which could benefit from a more efficient oral hygiene protocol.

## P049 Positive pocket cultures and infection risk after cardiac electronic device implantation-a retrospective observational single-center cohort study

### P Pekić^1^, M Bura^2^, N Marić^1^

#### ^1^University Hospital "Sveti Duh", Zagreb, Croatia,^2^Neuropsychiatric hospital "dr. Ivan Barbot", Popovača, Croatia

**Introduction:** Positive pocket cultures after implantation of cardiac implantable electronic devices (CIEDs) are often found without clinically apparent infection. Infections related to CIEDs are a serious complication requiring complete device removal, prolonged antimicrobial therapy and can have an adverse patient outcome.

**Methods:** We performed a retrospective observational single-center cohort study on 251 patients who received de novo implantation of pacemaker, cardioverter-defibrillator or cardiac resynchronization therapy device in a two-year period. Each patient was implanted using standard aseptic procedure according to local protocol and antibiotic (cefazolin) prophylaxis before the procedure. Pocket aspirate was taken after irrigating the wound with normal saline just before device placement.

**Results:** We analyzed 251 patients (58.6% male, 41.4% female). The most often implanted device was a DDD pacemaker followed by a VVI pacemaker. Mean length of hospital stay was 12.02±8.34 days. There were 54 (21.5%) positive cultures with overall 3 (1.19%) clinically apparent infections which required prolonged iv antibiotics, removal of device and reimplantation after infection resolution. In regard to microbiology, S. epidermidis (48.2%) and coagulase negative Staphylococcus (29.6%) were the most often finding which is in contrast to the cultures described in the literature. The only statistically significant risk factor for positive pocket culture was male sex and presence of a urinary catheter. Invasive vascular devices, previous intrahospital infection, and diabetes were not found to increase the likelihood of positive pocket culture.

**Conclusions:** Positive pocket cultures after CIED implant are a frequent finding mostly due to contamination and colonisation. The risk factors for such a finding differ from the usual and expected clinical circumstances. Our results are consistent with those in the literature. It turns out that the most important preventive measure in CIED implantation is strict aseptic procedure.

## P050 Use of dry bathing for intensive care patients

### W Yacov, Y Polishuk, A Geal-dor, G Yosef- hay

#### Kaplan Medical Center, Rehovot, Israel

**Introduction:** Intensive care patients are in constant risk of contamination due to suppression of their immune system, use of invasive procedures and medical equipment and health associated infections (HAI). Chlorhexidine Gluconate (CHG) is an antiseptic and disinfectant product. In medical research it has been found that daily CHG bathing is affective in reducing levels of skin and central line related infections (Climo, 2013). It is also referred to in the recommendations of the ministry of health "prevention of septicemia due to central lines" (2011).

**Methods:** Unit guide lines for patient Dry Bathing were written in May 2015 and thereafter began the implementation and instruction of nursing staff. Quality control was inspected by observation. There was a 15 phase questioner that included several categories such as: preparation of the CHG solution, staff protection actions, infusions and surgical wound dressings, bathing performance and documentation.

**Results:** A gradual rise of 97%was observed in theperformance ofdry bathing according to the unit guidelines

**Conclusions:** 97% of observed dry baths where performed according to the guide lines. Points for improvement: Correct care of infusions and surgical wound dressing and verify use of separate wipes for each body part. Next we will examine the correlation between the use of dry baths and theextent of infections in the unit. Dry Baths are nowconsidered an integralpart of the daily nursing routine. They have no substantial costs, help prevent complications from infection and add to the patient’s safety.

## P051 Pragmatic selective digestive decontamination (SDD): ventilator-associated pneumonia (VAP) rates & local antibiotic resistance

### J Highgate^1^, A Rashid^2^, J Kilic^1^, F Baldwin^1^

#### ^1^Brighton and Sussex University Hospitals NHS Trust, Brighton, UK,^2^University of Brighton, Brighton, UK

**Introduction:** Despite reductions in mortality reported with SDD, concerns about bacterial resistance and alteration of microbiome limit use. A retrospective observational study was conducted into the effect of local SDD protocols on VAP rates and resistance patterns. Over a 2-year period, 2 regimens were used dependent on drug availability and hospital antibiotic stewardship concerns. The study was designed to review practice and identify any risks of partial implementation.

**Methods:** Patients ventilated on a general intensive care were identified via clinical information systems. Three periods were reviewed for adherence to SDD protocols, Pre SDD (Jan - Feb 14), Full (July - Sept 15) and Partial (July - Sept 16). High-risk patients during both SDD periods also received IV antibiotics for 96 hours. Patients admitted with pneumonia or tuberculosis were excluded from VAP analysis. Remaining patients’ records were reviewed and the Clinical Pulmonary Infection Score (CPIS) calculated for each ventilated day to identify VAP rates. Positive respiratory microbiological results for all patients admitted to the ICU during each time period were reviewed to assess for wider changes in local resistance patterns.

**Results:** Protocol adherence was assessed in 71 patients during the full SDD period and 70 during the partial (Table 1). The number of patients included for analysis of VAP rates during each period was 38 pre SDD, 50 during full SDD and 37 during partial SDD. There were no significant changes in resistance patterns or Clostridium difficule rates (Table 2).

**Conclusions:** Compliance with the available enteral antibiotics was reasonable but with IV antibiotics was poor. It is accepted that alterations and non-adherence to protocols risk development of resistant bacterial strains. Within our unit no decrease in VAP rates was seen but reassuringly no increased rates of extended bacterial resistance were identified during the treatment periods.

**Table 1 (abstract P051).** SDD Protocol adherence & VAP rates

SDD Period	Protocol	Adherence % NG Gentamicin	Adherence % IV antibiotics	VAP cases per 1000 ventilator days (P=0.8)
Pre	Oral chlorhexidine	N/A	N/A	16.6
Full	Oropharyngeal SDD paste & NG gentamicin/nystatin	80.3	45	20.1
Partial	NG gentamicin/nystatin	70	60	16.7

**Table 2 (abstract P051).** Number of resistant organisms isolated

Organism & resistance	Pre	Full	Partial
Enterobacteriaceae	N=24	N=22	N=23
Ceftazidime	4	2	6
Gentamicin	0	0	1
Pseudomonas sp.	N=10	N=12	N=11
Ceftazidime	2	0	1
Meropenem	0	0	2
MRSA	N=1	N=1	N=3

## P052 Arterial catheter colonization and related bloodstream infection in ICU: is this an issue?

### S Moreira, C Silva, JP Baptista, C Monteiro, A Marques, P Coutinho, J Pimentel

#### Centro Hospitalar e Universitário de Coimbra, Coimbra, Portugal

**Introduction:** Arterial catheters are commonly used in Intensive Care Units (ICU) and are among the most frequently manipulated vascular access devices. Our aim was to evaluate the rate of arterial catheter-related bloodstream infection and colonization.

**Methods:** This was a 12-month, prospective and monocentric cohort study, performed in a multipurpose ICU. All arterial catheters, inserted in or presented to the ICU, were cultured and assessed for colonization or catheter-related bloodstream infection (CRBI).

**Results:** We enrolled 119 patients (63.8% males, average age 59±17 years, SAPS 2 42±21) of whom a total of 141 arterial catheters were analyzed for a total of 1552 catheter-days. Radial arterial catheters were inserted in 88.7% (n=125), femoral arterial catheters in 7.8% (n=11) and other arterial catheters in 3.5% (n=5). Signs of dysfunction were found in 28.8% and 45.5%, respectively. Radial arterial catheters colonization (n=5) and CRBI (n=1) occurred at a rate of 3.0 and 0.8/1000 catheter-days. Femoral arterial catheters colonization (n=2) and CRBI (n=1) occurred at a rate of 10.8 and 5.4/1000 catheter-days, respectively. Mean catheter time insertion was significantly higher in colonized catheters/CRBI (21±8 days; 95% CI: 14-28) when compared to arterial catheters with negative cultures (10±8 days; 95% CI: 9-12); p = 0.002). Colonized lines showed *Acinetobacter baumannii* (n=3), *Staphylococcus epidermidis* (n=1), Enterococcus spp (n=1) and *Pseudomonas aeruginosa* (n=1). CRBI were caused by *Staphylococcus epidermidis* (n=1) and *Staphylococcus haemolyticus* (n=1).

**Conclusions:** The incidence of radial arterial catheters colonization and CRBI were lower than reported rates in literature. Colonization and CRBI rates were higher in femoral catheters. Femoral catheters showed dysfunction more frequently. Prolonged catheterization was associated with colonization and CRBI.

## P053 A multimodality approach to decreasing ICU infections by hydrogen peroxide, silver cations and compartmentalization and applying acinetobacter as infection marker

### A Al Bshabshe^1^, M Hamid ^1^, A Assiri^2^, M Joseph^1^

#### ^1^King Khalid University, Abha, Saudi Arabia, ^2^Aseer Central Hospital, Abha, Saudi Arabia

**Introduction:** Nosocomial infections at the intensive care unit (ICU) represent a substantial health threat [1, 2]. ICU infections are mainly attributed to the extended hospital delay which results in high morbidities and mortalities.

**Methods:** A cross sectional study was conducted at the Intensive Care Unit, Aseer Central Hospital, Saudi Arabia over 13 months period (2014-2015). The intervention program included the application of mist of hydrogen peroxide and silver cations, physical separation and compartmentalization of the intensive care unit. The GLOSAIR™ 400 System was used to deliver a mist of hydrogen peroxide and silver cations. Hydrogen peroxide is an oxidizing agent, which kills microorganisms.

**Results:** A total of 103 strains of Acinetobacter species were identified from the patients over the 13 months period (Fig. 1). The mean infection rates decreased from 14.3 in the first three months of the program to 4 in the last three month after continuous.

**Conclusions:** The program using the three procedures offered a significant decrease in infections at the ICU as measured by Acinetobacter count, which is one of the most hazardous nosocomial pathogens.

**References**

1. Burgmann H et al. Intensive Care Med 36:1597-1601, 2010.

2. Boncagni F et al. Minerva Anestesiol 81:765-775.3, 2015.

**Fig. 1 (abstract P053).**
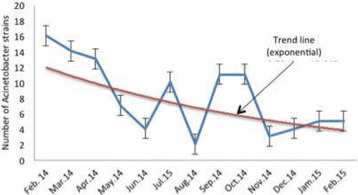
A linear regression of Acinetobacter species recovered from the intensive care unit, Aseer Central Hospital, Saudi Arabia: A one year trend analysis (y = - 0.7912x + 13.615; R^2^ = 0.44)

## P054 A review of current practice of continuous antibiotic infusions on intensive care units in England

### G Page, E Turner, C Day

#### Royal Devon and Exeter Hospital, Exeter, UK

**Introduction:** The efficacy of ß lactam antibiotics is related to the time above MIC. Continuous or extended infusions can be used to increase the time above MIC, especially in patients with normal or increased drug clearance. Administering antibiotics by continuous infusion is not a new concept. A review in 1992 looks at the outcomes of continuous infusions [1]. More recently an improvement in mortality has been demonstrated [2]. Our perception was that uptake of this low cost intervention was not common, so we undertook a survey to determine how commonly continuous infusions are used in England.

**Methods:** A telephone survey of all intensive care units in England was undertaken. Questions included:

- Are you using continuous or extended antibiotic infusions?
- Which antibiotics are you using for continuous or extended infusions?
- If not currently using has it been considered?

Data was collected over a week in June 2017.

**Results:** There was an 87% response rate. 73 (44.5%) of the units continuously infuse some antibiotics, however 71.2% of those only infuse vancomycin and not ß lactams. Only 21 of the total responders (12.8%) infuse antibiotics other than vancomycin (i.e. ß lactams).

**Conclusions:** The theoretical advantage of continuous infusion of ß lactam antibiotics has been described for over 20 years. There is now evidence that this may improve survival. Despite this, uptake in England has been slow.

**References**

1. Craig WA et al. Antimicrob Agents Chemotherap 36(12):2577-83, 1992

2. Roberts JA et al. Am J Respir Crit Care Med 194(6):681-91, 2016

## P055 Infections in a tertiary referral hospital intensive care unit in Rwanda

### J Mvukiyehe^1^, P Banguti^1^, R Elisabeth^2^, J Richard^3^, E Tuyishime^1^

#### ^1^University of Rwanda, Kigali, Rwanda,^2^Harvard University, Boston, MA, USA,^3^Minnesota University, Minneapolis, MN, USA

**Introduction:** Infections contribute to a significant proportion of morbidity and mortality worldwide. While many infections are successfully managed with antimicrobial therapy, rates of antimicrobial resistance (AMR) are increasing. Certain patient populations such as those admitted to intensive care units (ICU) are at high risk.

**Methods:** We conducted a retrospective, observational study of all ICU patients at a tertiary referral hospital in Rwanda from January 2015 through December 2016 We collected data on diagnosis, ICU length of stay, mortality and hospital length of stay, as well as microorganism, site of culture, AMR and antibiotics prescribe.

**Results:** Overall, 331 patients were admitted to the ICU. Most patients were admitted from the main operating theater (n=150, 45%).The most common admitting diagnoses were sepsis (n=113, 34%), head trauma (n= 90, 27%). A total of 268 samples were collected from 331 patients. The samples were from blood (n=110, 33%), tracheal aspirate (n=22, 7%),. The most common organisms isolated were Klebsiella (n=30, 29%), Acinetobacter (n=20, 19%), E.coli (n=16, 15%), Proteus (n=15, 14%), Citrobacter (n=8, 8%), S aureus (n=7, 7%), Pseudomonas (n=5, 5%), and other (n=9, 9%). Of Klebsiella isolates, 100% and 76% were resistant to ceftriaxone and cefotaxime, respectively. Of E.coli isolates, 86% and 71% were resistant to ceftriaxone and cefotaxime, respectively. All Acinetobacter isolates were resistant to ceftriaxone and cefotaxime.

**Conclusions:** There is an alarming rate of antimicrobial resistance to commonly used antibiotics in the ICU. Expanding antibiotic options and strengthening antimicrobial stewardship are critical for patient care.

## P056 The last three days

### G Latten^1^, P Stassen^2^

#### ^1^Zuyderland MC, Sittard-Geleen, Netherlands,^2^Maastricht UMC+, Maastricht, Netherlands

**Introduction:** This study provides an overview of the prehospital course of patients with a (suspected) infection in the emergency department (ED). Most research on serious infections and sepsis has focused on the hospital environment, while potentially most delay, and therefore possibly the best opportunity to improve treatment, lies in the prehospital setting.

**Methods:** Patients were included in this prospective observational study during a 4 week period in 2017. All patients aged 18 years or older with a suspected or proven infection were included. Prehospital, ED and outcomes were registered.

**Results:** In total, 2452 patients visited the ED during the study period, of whom 440 (17.9%) patients had a (suspected) infection. (Fig. 1) Median duration of symptoms before ED visit was 3 days (IQR 1-7 days), with 23.9% of patients using antibiotics before arrival in the ED. Most patients (83%) had been referred by a general practicioner (GP), while 41.1% of patients had visited their GP previously during the current disease episode. Twenty-two patients (5.0%) experienced an adverse outcome (ICU admission and/or 30-day all- cause mortality): these patients were less often referred by a general practicioner (GP) (59.1 vs. 84.2%, p=0.001) and were considered more urgent both by EMS and in the ED.

**Conclusions:** The prehospital phase of patients with an infection provides a window of opportunity for improvement of care. Patients become ill 3 days before the ED visit and 41.1% already visited their GP previously during the current disease episode, while 23.9% is currently using antibiotics. Future research should focus on quality improvement programs in the prehospital setting, targeting patients and/or primary care professionals.

**Fig. 1 (abstract P056).**
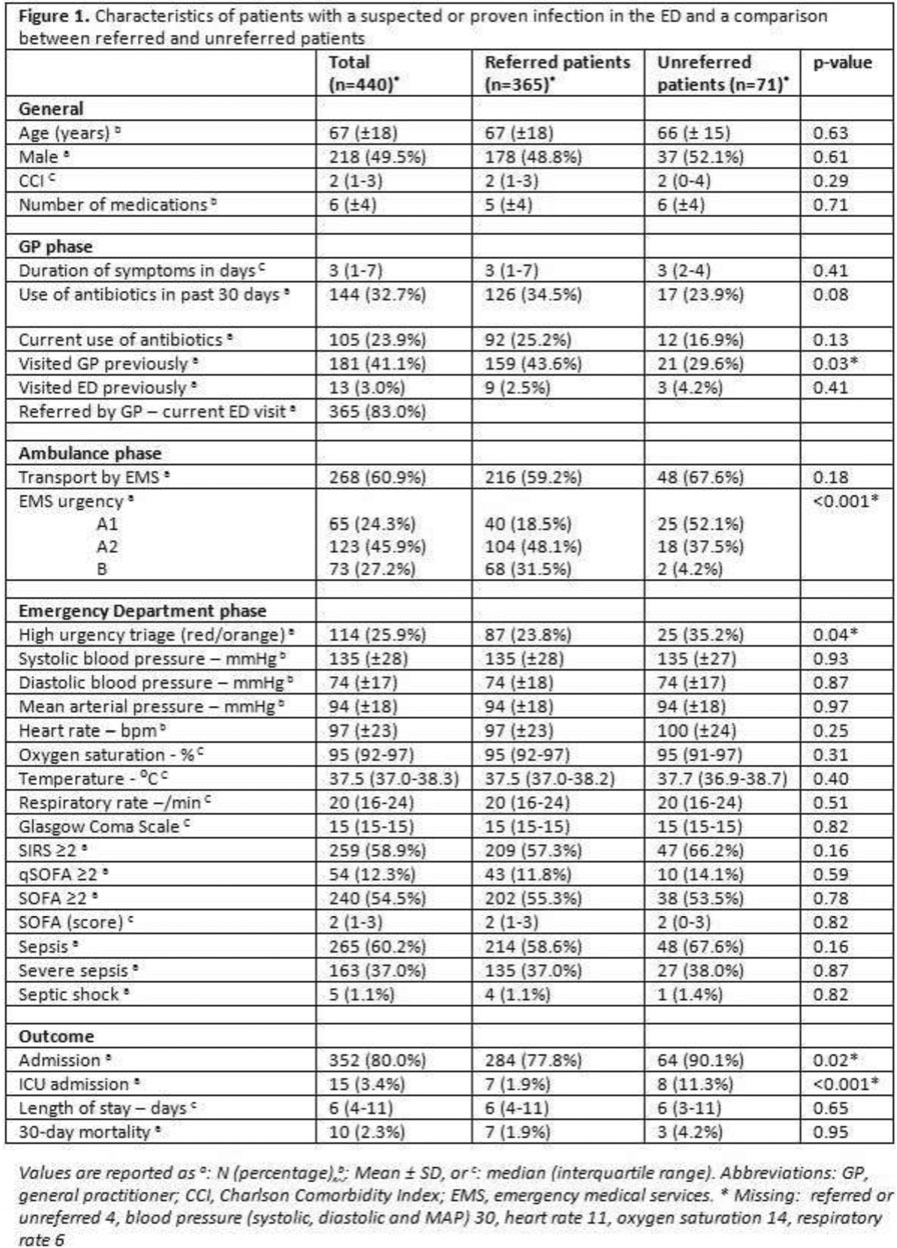
See text for description

## P057 Severe tetanus: clinical data in a Moroccan medical intensive care unit

### H Ezzouine, Z Sghier, Y Hafiani, K Mediouni, A Benslama

#### Faculty of medicine and pharmacy.University Hassan II.Casablanca, Casablanca, Morocco

**Introduction:** Worldwide, the prevalence of tetanus has decreased.However, even if progress has been made in the combat to eradicate tetanus it may be a cause of admission to intensive care.The objectives of our study are to determine epidemiological,clinical and prognostic characteristics for severe tetanus in our unit.

**Methods:** We conducted a retrospective study in the medical intensive care unit of Ibn Rushd hospital in Casablanca in Morocco from 2010 to 2016.We studied the epidemiological,clinical and prognostic characteristics of the patients who were admitted for severe tetanus.

**Results:** The incidence of severe tetanus was 2.04% affecting male in 100%.41.9% were aged between 31 and 40 years old. In 85.7% there were a integumentary portal of entry. Contractures were present in 69%of the cases. At intensive care unit admission, 21.4% of the patients were sedated. The anti-tetanus vaccination was never updated. According to the Dakar score 28.6% of the patients were listed Dakar 1, 54.8% Dakar 2 and 16.6% Dakar 3. For the Mollaret score, the crude form was found in 44.2%, the acute generalized form was found in 32.6% and the severe form in 20.9% of the cases.Mechanical ventilation was necessary in 83.3%. Diazepam and baclofen were used in 92.9%, phenobarbital in76.2% and propofol in 42.85%. A serotherapy was used for all the patients and a preliminary vaccination dose for 26.9%. All the patients received antibiotics, penicillin G 33.33% and metronidazole 76.2%. The mortality was 61.9%. The length of intensive care stay was significantly higher. The need for an intubation,its duration and the occurrence of autonomic dysfunction have significantly influenced the mortality.

**Conclusions:** To improve the prognosis in these serious forms of tetanus,it is highly important to identify the warning signs and refer patients in intensive care for early and appropriate management in intensive care.

## P058 Bloodstream infections in the ICU of a tertiary care hospital: analysis of resistance patterns

### T Melissopoulou, S Kolovou, M Panoutsopoulou, T Kypraiou, M Papadimitriou, O Apostolou, J Deliolanis, A Pantazatou, A Skiada, J Pavleas, J Floros

#### Laiko Hospital, Athens, Greece

**Introduction:** Bloodstream infections (BSIs) are associated with increased mortality in the ICU. The aim of the study was to evaluate the epidemiology and resistance patterns during the period 2013 to 2017.

**Methods:** Bacteria and fungi isolated from the blood of patients hospitalized in a mixed ICU during the study period were retrospectively analyzed. Sensitivity testing was performed with disk diffusion (Kirby-Bauer) and Microscan Walkaway 96 plus for minimal inhibitory concentrations.

**Results:** During the study period 1198 patients were hospitalized in the ICU. BSIs were diagnosed in 284 cases (23.7%). The isolated microorganisms were *Acinetobacter baumannii* (29%), *Klebsiella pneumoniae* (15%), other Enterobacteriaceae (8%), *Pseudomonas aeruginosa* (6%), *Stenotrophomonas maltophilia* (1%), enterococci (20%), staphylococci (8%) and *Candida* spp. (13%). Of the *A. baumannii* isolates, 97% were resistant to carbapenems, 9.6% to colistin, and 31% to tigecycline. Of the *K. pneumoniae* isolates 80% were resistant to carbapenems, 70% to colistin, and 4.5% to tigecycline. Of the *P. aeruginosa* species 44% were resistant to carbapenems and they were all susceptible to colistin. The rate of resistance to vancomycin was 56% for the *E. faecium* isolates, 5.5% for the *E. faecalis*, while the resistance to methicillin of the coagulase negative staphylococci was 90%. The most commonly isolate species of Candida was *C. albicans*.

**Conclusions:** Multi-drug resistant isolates, especially *A. baumannii* and Enterobacteriaceae, are a serious problem in our ICU. Gram positive bacteria are less common, but the resistance of enterococci to vancomycin is significant. Antibiotic stewardship and infection control measures should be applied in a more strict way.

## P059 Nosocomial sinusitis in intensive care unit patients

### I Titov ^1^, S Melnyk^2^, M Grynovska^1^

#### ^1^Ivano-Frankivsk National Medical University, Ivano-Frankivsk, Ukraine,^2^Ivano-Frankivsk Regional Clinical Hospital, Ivano-Frankivsk, Ukraine

**Introduction:** Nosocomial sinusitis (NS) is a complication of critically ill patients which develops 48-72 h after admission and is mostly linked but not limited to such invasive procedures as nasotracheal intubation and nasogastric tube placement. NS is often overlooked as a source of pyrexia of unknown origin, meningeal manifestations, sepsis and ventilator associated pneumonia in ICU patients. CT scanning and sinus puncture are used to confirm the inflammatory process and identify the pathogen behind it.

**Methods:** A retrospective case study of 6.479 ICU patients for a period of 2012-2016 was performed. We have analysed data from the CT scans of paranasal sinuses and bacteriological findings of samples obtained from sinus puncture.

**Results:** 644 (9.9%) patients were suspected of NS on the 5-7th day of stay in the ICU. The CT scan confirmed pathological changes in 464 patients (7.1%). Hemisinusitis was detected in 422 patients (90.9%) and pansinusitis in 41 patients (8.8%). There was also an isolated case of maxillary sinusitis in 1 patient (0.2%). The pathogenic culture was identified only in 297 (64%) samples, 34.6% of which revealed isolated bacteria and 65.4% a polymicrobial association. Gram positive bacteria were detected in 16.1% of cases and Gram negative in 49.5%. Most cases revealed multiple antibiotic resistance.

**Conclusions:** 1. NS has proved to be largely caused by Gram negative bacteria and polymicrobial associations. The use of broad spectrum antibiotics in ICU may justify the presence of sterile cultures.

2.Early identification of risk patients in ICU as well as the use of screening CT scan may benefit timely diagnosis and adequate treatment of patients.

3.Preventive considerations include: patient’s bed head elevation, the use of oral gastric tube in sedated and coma patients on ventilation, nasotracheal intubation only if indicated, removal of nasogastric tube at night, proper hygiene.

## P060 The impact of TB on ICU in a high incidence area of the UK: a ten year analysis

### J Barrett^1^, A Keeley^1^, D Keane^1^, L John^1^, W Lynn^2^, N Sabir^1^

#### ^1^Northwick Park Hospital, London, UK,^2^Ealing Hospital, London, UK

**Introduction:** Despite wide availability of effective treatment a minority of patients with TB will become critically ill. Here we describe the presentation, demographics and outcomes of patients with TB admitted to ICU in our trust which is based in an area of high TB incidence in London (incidence >50/100000).

**Methods:** The London TB register was cross-referenced against ICU records from 01.01.07 to 31.12.2016. Clinical data were collected for matched patients.

**Results:** 78 patients identified; 50% of South Asian origin, 29.5% of African origin, 12% UK born 31% of patients had multifocal TB. TB sensitivities: 89% fully sensitive, 7% mono-drug resistance, 4% multi-drug resistant TB. Median length of ICU stay was 5 days (IQR 2-14). 6 patients were readmitted. 23 patients died on ICU (29.5%), 10 patients died prior to hospital discharge. Median time to death following ICU admission: 15 days (IQR 5-35).

**Conclusions:** Only 78 of 4,011 TB patients (2%) required critical care intervention (Table 1). Those admitted to ICU were older and more likely to have pulmonary, CNS, miliary or abdominal TB (Table 2). Mortality was high despite critical care input in a unit familiar with managing TB, and 24 hour access to Infectious Diseases advice within the trust, likely due to overwhelming organ dysfunction, patient frailty and advanced TB infection. Rates of drug resistant TB were low and comparable to UK-wide rates over that period (5% mono-drug resistant, 2% MDR) thus less likely a contributory factor to the majority of deaths.

**Table 1 (abstract P060).** Reasons for ICU admission

Reason for admission	No (%)
Type 1 respiratory failure	30
Type 2 respiratory failure	8 (10)
Sepsis/septic shock	18 (23)
Renal failure	12 (15)
Neurological dysfunction	7 (9)
>1 reason for admission	24 (31)

**Table 2 (abstract P060).** Characteristics of ICU TB patients vs non-ICU TB patients

	ICU TB patients (n=78)	Non-ICU TB patients (n=3933)
Mean age	56 (range 17-84)	28 (range 0-96)
%Male	68%	58%
TB smear +/culture +	28%/54%	20%/49%
Pulmonary TB	64%	44%
Abdominal TB	17%	8%
Miliary TB	13%	3%
TB meningitis	12%	2%

## P061 Short term antibiotics prevent early VAP in patients treated with mild therapeutic hypothermia after cardiac arrest

### T Daix^1^, A Cariou^2^, F Meziani^3^, PF Dequin^4^, C Guitton^5^, N Deye^6^, G Plantefève^7^, JP Quenot^8^, A Desachy^9^, T Kamel^10^, S Bedon-Carte^11^, JL Diehl^12^, N Chudeau^13^, E Karam^14^, F Renon-Carron^1^, A Hernandez Padilla ^1^, P Vignon^1^, A Le Gouge^4^, B François^1^

#### ^1^Centre Hospitalier Universitaire Dupuytren, Limoges, France,^2^hôpital Cochin (APHP) et université Paris Descartes, Paris, France,^3^Université de Strasbourg (UNISTRA), Faculté de Médecine, Hôpitaux universitaires de Strasbourg/Nouvel hôpital civil, Strasbourg, France,^4^CHU Bretonneau, Tours, France,^5^CHU de Nantes, Nantes, France,^6^CHU Lariboisière, APHP, Paris, France,^7^CH Victor Dupouy, Argenteuil, France,^8^CHU François Mitterrand, Dijon, France,^9^Centre Hospitalier d’Angoulême, Angoulême, France,^10^CHR d’Orléans, Orléans, France,^11^Centre Hospitalier de Périgueux, Périgueux, France,^12^HEGP, AP-HP, Paris, France,^13^Centre hospitalier du Mans, Le Mans, France,^14^Centre hospitalier général, Brive la Gaillarde, France

**Introduction:** Patients treated with mild therapeutic hypothermia after cardiac arrests with shockable rhythm are at high risk of ventilator-associated pneumonia (VAP) [1]. Despite retrospective trials suggesting a benefit of short-term (48h) antibiotics in this setting [2], it is not recommended. The primary objective was to demonstrate that systematic antibiotic prophylaxis can reduce incidence of early VAP (<7 days). The impact on incidence of late VAP and on Day 28 mortality was also assessed.

**Methods:** Multicenter, placebo-controlled, double-blinded, randomized trial. ICU patients >18 years, mechanically ventilated after out-of-hospital resuscitated cardiac arrest related to initial shockable rhythm and treated with mild therapeutic hypothermia were included. Moribund patients and those requiring extracorporeal life supports, with ongoing antibiotic therapy, known chronic colonization with multiresistant bacteria or known allergy to beta-lactam antibiotics were excluded. Either IV injection of amoxicillin-clavulanic acid (1g/200mg) or placebo was administered 3 times a day for 2 days. All pulmonary infections were recorded and blindly confirmed by an adjudication committee.

**Results:** In intention to treat analysis, 196 patients were analyzed, (treatment group n=99; mean age 60.5±14.4 years, sex ratio=4, SOFA score 8.7±3.1). Global characteristics of cardiac arrest were similar (no flow= 3.5min vs 3.8min, low-flow= 21.8min vs 18.2min). 60 VAP were confirmed incl. 51 early VAP, 19 in treatment group vs 32 in placebo group (HR=0.546; IC 95%=[0.315; 0.946]) (Fig. 1). Occurrence of late VAP (4% vs 5.1%) and Day 28 mortality (41.4% vs 37.5%) was not affected by the study procedure.

**Conclusions:** Short-term antibiotic prophylaxis significantly decreases incidence of early VAP in patients treated with mild therapeutic hypothermia after out-of-hospital cardiac arrest related to shockable rhythm and should be recommended.

**Reference**

1. Mongardon N et al Crit Care Med 39:1359-64, 2011.

2. Davies KJ et al Resuscitation 84:616-9, 2013

**Fig. 1 (abstract P061).**
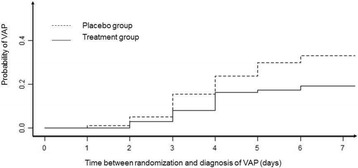
Incidence of early VAP

## P062 Withdrawn

## P063 Clinical burden of inappropriate empirical antimicrobial therapy of in-hospital complicated intra-abdominal infections

### S Carelli, T Taccheri, MS Vallecoccia, SL Cutuli, V Di Gravio, G De Pascale, M Antonelli

#### Fondaz. Policlinico A. Gemelli, Rome, Italy

**Introduction:** Complicated intra-abdominal infections (cIAIs) remain a common cause of morbidity and mortality among ICU patients and therapeutic failure still occurs. This study aimed to assess the clinical impact of an initial inappropriate empirical therapy of cIAIs.

**Methods:** This retrospective study enrolled patients admitted to the ICU of the Fondaz. Pol. A. Gemelli in Rome, between February 2010 and February 2017 with a diagnosis of in hospital cIAI. Comparisons between patients receiving initial inappropriate antimicrobial therapy (IIAT) and initial appropriate antimcrobial therapy (IAAT) were performed.

**Results:** A total of 137 patients were included (IIAT=44 and IAAT=93). Baseline characteristics were comparable, with the exception of SOFA score at infection which was higher in IAAT (p=0.04). Secondary peritonitis was the main type of cIAI (45.5% in IIAT and 40.9% in IAAT) followed by abdominal abscess and biliary tract infection. Secondary bacteraemia was significantly higher in IIAT (p=0.03). Conversely, IAAT had an higher rate of adequate source control (p=0.01). Empirical therapy of IAAT patients included more frequently anti gram-positive (p=0.016) and carbapenems (p=0.01), while empirical dual anti gram negative and antifungal coverages rate were not different (Fig. 1). MDR and polimicrobial infections rate was significantly higher in IIAT when associated with septic shock at occurrence of infection (p=0.03; Fig. 2). IAAT showed significantly lower mortality at 28 and 90 days (p<0.01) as well as higher rate of clinical cure and microbiological eradication than IIAT (p<0.01). At the multivariate analysis, adequate source control [p=0.04, OR 0.25 (0.09-0.65)] and IIAT [(p<0.01, OR 11.4 (4.02-32.3)] turned out to be independently related with 28 days mortality.

**Conclusions:** In cIAIs an appropriate empirical antibiotic therapy and an early infection source control are closely associated with better outcomes.

**Fig. 1 (abstract P063).**
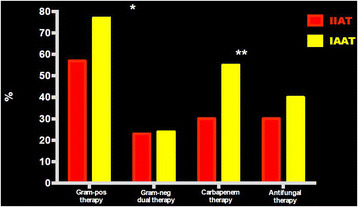
See text for description

**Fig. 2 (abstract P063).**
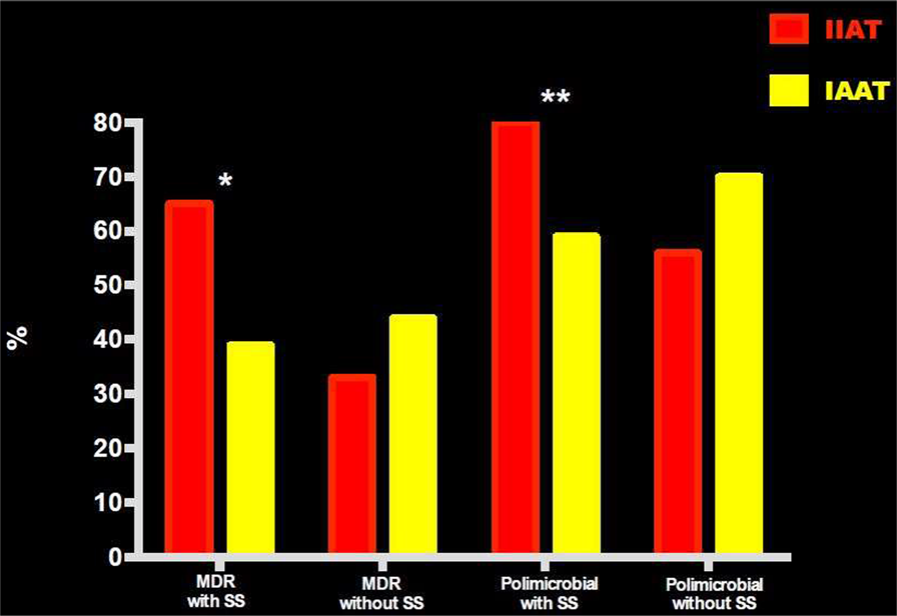
See text for description

## P064 Prospective observational study evaluating antibiotic prescription pattern and microbial isolates and their correlation with hemodynamic stability in icu patients

### M Bhattacharyya ^1^, A Saha^2^, T Chatterjee^2^, S Todi^1^

#### ^1^AMRI Hospitals, Kolkata, India,^2^Jadavpur University, Kolkata, India

**Introduction:** Antibiotics are the most commonly prescribed drugs in ICU.In the era of antibiotic resistance it is difficult to choose antibiotics during septic episode.The choice antibiotics mainly depends on clinical diagnosis,culture sensitivity and local flora. Whether severity of illness really maters is not well known. To study antibiotic prescription pattern and whether the choice of antibiotic varies according to hemodynamic stability in patients admitted in ICU.To study of microbiological isolates and their variability according to hamodynamic stability in ICU patients.

**Methods:** All ICU patients of more than 18 years age who received antibiotics and where cultures had been sent were included in the study.Patients discharged against medical advice and where treatment had been withdrawn were excluded in this study. This prospective observational study was conducted between July 2016 to March 2017.Patients were divided into stable and unstable group according to hemodynamic parameter and usage of antibiotics and microbiological isolated were correlated. ICU mortality and length of stay were correlated between hemodynamically stable and unstable group.

**Results:** 786 sepsis episode were analysed. Mean age was 65 years, male predominant, and average APACHE IV score was 58(SD25). We had 444 patients in unstable group of which 71% patients got discharged and 86% of patients got discharged in stable group. Antibiotic combination therapy was used more in hemodynamically unstsble patients(p 0.3). BLBLI was used more in stable group. Drug resistance in microbiological isolates did not reveal any statistically significant difference among stable or unstable group.

**Conclusions:** There is a tendency to administer combination antibiotics in sicker group of patients with hemodynamic instability. Prevalence of microbial flora did not show any statistical difference. outcome is worse in hemodynamically unstable patients.

## P065 The clinical significance of Candida score in critically ill patients with candida infection

### H Al-Dorzi^1^, R Khan^1^, T Aldabbagh^1^, A Toledo^1^, S Al Johani^1^, A Almutairi^2^, S Khalil^2^, F Siddiqui^2^, Y Arabi^3^

#### ^1^King Abdulaziz Medical City, Riyadh, Saudi Arabia,^2^MSD, Riyadh, Saudi Arabia,^3^King Saud bin Abdulaziz University for Health Sciences, Riyadh, Saudi Arabia

**Introduction:** Candida score (CS) is used to identify patients with invasive candidiasis in the ICU, but its clinical use has not become widespread. Our objective was to evaluate the clinical significance of CS in a mixed population of ICU patients.

**Methods:** This was a prospective observational study of critically ill patients who had Candida species growth during their stay in any of six different ICUs of a tertiary-care center. Two intensivists classified patients as having Candida colonization or invasive candidiasis according to predefined criteria. CS was calculated for each patient on the day of Candida species growth as follows: 1 point for parenteral nutrition + 1 point for surgery + 1 point for multifocal Candida colonization + 2 points for severe sepsis. The Receiver Operating Characteristic (ROC) curve was plotted to assess CS ability to discriminate between invasive candidiasis and Candida colonization.

**Results:** CS was 1.6±0.9 in patients with Candida colonization (N=261) and 2.4±0.9 in those with invasive candidiasis (N=120) (p<0.001). However, only 38.7% of invasive candidiasis cases had CS >= 3 (compared with 8.0% of Candida colonization cases; p<0.001). The ROC curve (Fig. 1) showed that CS had fair ability to discriminate between invasive candidiasis and Candida colonization (area under the curve 0.71, 95% confidence interval 0.65 to 0.77; p<0.001). In patients with invasive candidiasis, CS was similar in hospital survivors and nonsurvivors (2.2±0.9 and 2.5±0.8, respectively; p=0.13). CS did not discriminate between survivors and nonsurvivors (area under the ROC curve 0.61, 95% confidence interval 0.46 to 0.75; p<0.15).

**Conclusions:** CS was higher in patients with invasive candidiasis than those with Candida Colonization. However, its ability to discriminate between these patients was only fair. CS was not associated with hospital mortality.

**Fig. 1 (abstract P065).**
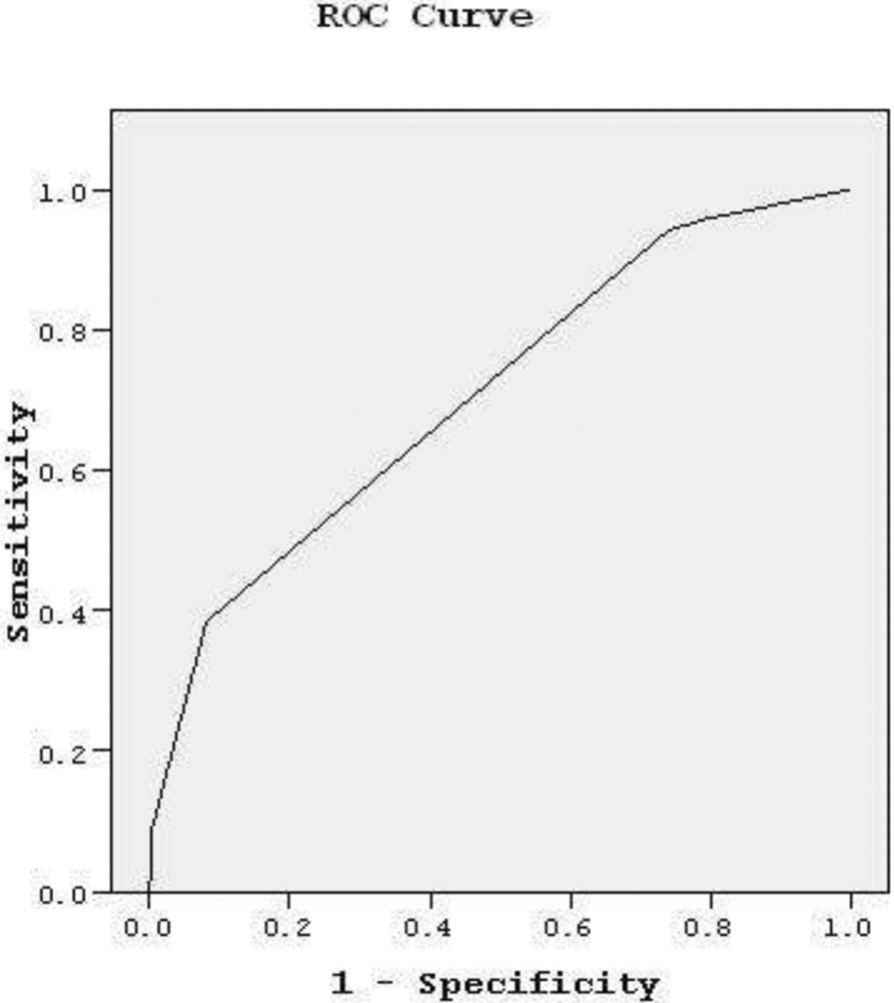
ROC curve for Candida score discrimintaing between invasive candidiasis and Candida colonization

## P066 Poor reliability of creatinine clearance estimates in predicting fluconazole exposure in liver transplant patients

### M Lugano, P Cojutti, F Pea

#### ASUIUD, Udine, Italy

**Introduction:** Invasive candidiasis (IC) is a frequent complication in liver transplant (LT) recipients, especially during the first 1-3 months after LT. Fluconazole is a triazole antifungal used for prophylaxis and treatment of IC. Due to its renal elimination, dose adjustments are usually based on estimated creatinine clearance (eCrCL). However, the reliability of eCrCL in predicting fluconazole clearance has never been investigated in this population. The aim of this study was to conduct a population pharmacokinetic (popPK) analysis in a cohort of LT patients who underwent therapeutic drug monitoring (TDM) in order to find out which covariates may influence fluconazole pharmacokinetics (PKs).

**Methods:** This retrospective study included LT patients who were admitted to the intensive care unit of our University Hospital between December 2007 and May 2016, and who were treated with intravenous fluconazole in the first months after LT. TDM of fluconazole was performed with the intent of attaining the efficacy pharmacodynamic target (AUC24h/MIC > 55.2). The tested covariates were: age, gender, CKD-EPI eCrCL, time from LT, serum albumin and transaminases, SAPS II score. PopPK was carried out with Pmetrics software.

**Results:** Nineteen patients (mean±SD age, weight and serum creatinine of 60±8.4 years, 75±16.8 kg, 1.0±0.62 mg/dL, respectively) with a total of 89 fluconazole trough plasma concentrations were included in the popPK analysis. Mean±SD fluconazole distribution volume (Vd) and clearance (CL) were 27.02±10.78 L and 0.55±0.19 L/h. Age and time from LT were the only clinical covariates significantly correlated with fluconazole Vd and CL, respectively. Conversely, CKD-EPI eCLCr was unable to predict fluconazole CL.

**Conclusions:** CKD-EPI eCLCr is unreliable in predicting fluconazole exposure in LT recipients. Consistently, in this population adaptation of fluconazole dose should be based on measured CrCL, and TDM may be helpful in optimizing drug exposure.

## P067 Outcomes of a candidiasis screening protocol in a medical ICU

### M Boujelbèn^1^, I Fathallah^1^, H Kallel^1^, D Sakis^1^, M Tobich^1^, S Habacha^1^, N Ben Salah^1^, M Bouchekoua^2^, S Trabelsi^2^, S Khaled^2^, N Kouraichi^1^

#### ^1^Hôpital Régional Yasminette, Ben Arous, Tunisia, ^2^Hôpital Charles Nicolle, Tunis, Tunisia

**Introduction:** The aim is to determine the incidence, characteristics and risk factors of invasive candidiasis (IC) in critically ill patients by using a weekly screening protocol.

**Methods:** A 9 months’ prospective study was conducted in a 6-bed MICU. The candidiasis screening consisted of the culture of plastic swabs (from different body sites), urine and respiratory tract samples.It was conducted upon admission and on weekly basis for all the patients. Decision to treat was based on clinical and microbiological features.

**Results:** 97 patients were included. The colonization rate with Candida spp was 28.8%(n=28). 415 screening samples were collected with a positivity rate at 27.9%(n=118). Table 1 describes the isolated Candida species by site. Antifungal resistance was tested in 72(62%) species. The resistance rate to fluconazole was 13.8%(n=10). The antifungal resistance of Candida albicans is detailed in Table 2. 14(14.4%) patients presented an IC with a mean age and mean SAPS II at 54.3 ± 18 years and 48±18.7 respectively. 7(50%) presented acute renal failure upon admission. 85.7% (n=12) of the patients needed mechanical ventilation. The median length of stay was 29 days [18.5-62.5] and the mortality rate was 42.9%(n=6). The mean SOFA score upon infection was 8.5±2.79. The candida score was >= 2.5 and the colonization index was >= 0.5 in 92.8%(n=13) and 78.5%(n=11) of the patients respectively. Only one patient had a positive blood culture. Mannan antigen and anti-mannan antibodies were screened only in five patients with a positivity rate at 100%(n=5). The most isolated species was: *Candida albicans* 64.3%(n=9). Multivariate analysis showed that prior use of Imipinem more than 6 days was a risk factor for IC (OR=9.37, CI95[1.15 ; 75.8], p=0.03).

**Conclusions:** This study showed the ecology and epidemiology of Candida species in our MICU with an increased IC rate and high mortality. Prior Imipinem use was a risk factor for IC.

**Table 1 (abstract P067).** Isolated Candida species by site

Site/Candida species	Nasal	Oral	Axillary	Respiratory tract	Inguinal	Rectal	Urine	Chronic Wound	Total
C. albicans	20	23	4	2	11	13	2	1	76
C. glabrata	1	1	1	0	3	3	1	1	11
C. tropicalis	1	2	0	2	0	1	1	0	7
C. krusei	0	0	1	0	2	1	0	0	4
C. famata	0	3	0	0	1	2	0	0	6
C. parapsilosis	2	0	2	0	0	0	0	0	4
C. dubliniensis	0	1	0	0	0	0	0	0	1
C. lusitaniae	0	0	0	0	2	1	0	0	3
Unidentified Candida spp	1	1	1	0	0	3	0	0	6
Total	25	31	9	4	19	24	4	2	118

**Table 2 (abstract P067).** Candida albicans’ antifungal resistance

	5-fluorocytosine	Amphotericin B	Fluconazole	Itraconazole	Voriconazole
Susceptible n(%)	47(92.1)	48(94.7)	46(90.2)	44(86.3)	48(94.7)
Intermediate n(%)	1(2.6)	0(0)	0(0)	0(0)	0(0)
Resistant n(%) n(%)	3(5.3)	3(5.3)	5(9.8)	7(13.7)	3(5.3)

## P068

**Withdrawn**

## P069 Invasive fungal infections (IFI) – harmonizing the guidelines for India

### Y Mehta^1^, O Shrivastav^2^, K Bajan^3^, A Khurana^4^, A Qamra^4^

#### ^1^Medanta The Medicity, Gurgaon, India,^2^Jaslok Hospital, Mumbai, India,^3^PD Hinduja Hospital, Mumbai, India,^4^Wockhardt, Mumbai, India

**Introduction:** ICU-acquired infection is as high as 42.7 episodes per 1000 patient-days in lower-middle income countries like India (WHO). Almost three times higher than in high-income countries [1]. Candida Infection is the 3rd most commonly acquired nosocomial infection in India burdening the debilitated patient with longer ICU stay [2]. There are no definite guidelines on whether & when to start anti-fungal treatment, specific to India where IFI risk is high and diagnostic facilities are limited. Currently, the Intensivists across India are using antifungals, according to their clinical experience and selective application of international guidelines leading to non-uniformity of patient outcomes.

**Methods:** In an endeavour to synchronize anti-fungal therapy and educate intensivists from small cities of India, 2 Intensivists and 1 Infectious Disease specialist of international repute were approached to design a module on ‘Invasive Fungal Infections – When to Start Anti-fungals in ICU [Fig. 1]. The IFI in India was summarised into a compact 1 hour session for dissemination of knowledge using IDSA 2016 as a reference guideline. 12 Intensivists from across India were trained on the module by our faculty. The module was rolled out to Intensivists and Pulmonologists focussing particularly on the tier-2 & tier -3 cities where avenues for learning are limited [Fig. 2].

**Results:** The module covered epidemiology, diagnostic challenges & anti-fungal therapies in Candidemia. It also included Candiduria, Aspergillosis & Mucormycosis that intensivists infrequently encounter. 4 meetings have been conducted and over 150 intensivists have been trained so far and more such trainings are planned in near future.

**Conclusions:** This module serves as a good academic tool to create awareness, education and harmonisation of anti-fungal treatment amongst HCPs across India.

**References**

1. WHO Report Burden of Endemic HCI, 2011

2. Oberoi JK JIMSA 23 No. 1, January - March 2010

**Fig. 1 (abstract P069).**
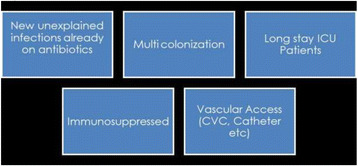
When to start Anti-fungal therapy in my patient?

**Fig. 2 (abstract P069).**
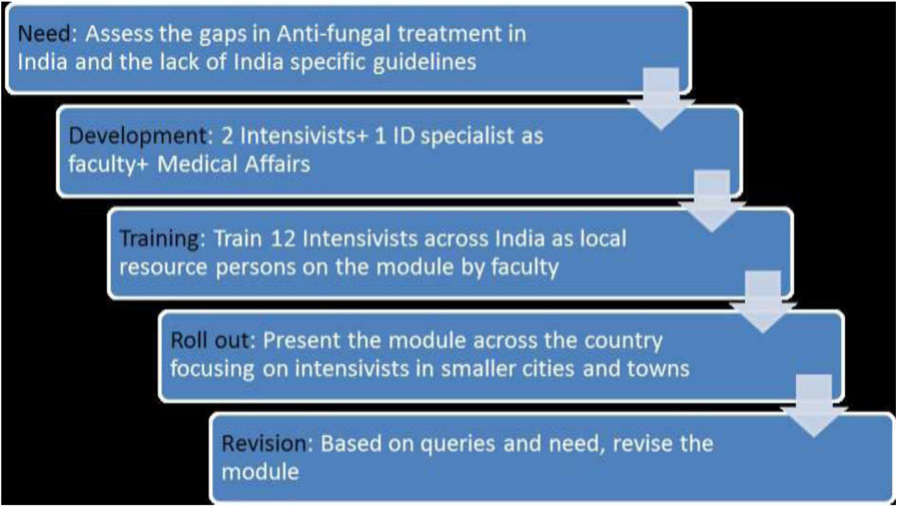
Design of Anti-fungal module development

## P070 Incidence of Trichosporon spp. urinary tract infections in ICU

### E Belesiotou, C Routsi, C Vrettou, E Magira, E Douka, P Kaltsas, M Nepka, E Perivolioti, E Kraniotaki, S Zakinthinos

#### Evaggelismos General HOSPITAL, Athens, Greece

**Introduction:** Trichosporon species are fungi found in nature and human normal flora but they can be an opportunistic pathogen, associated with medical devices (biofilm formation), especially in intensive care unit(ICU) patients.

**Methods:** After cultivation of urine samples, the identification was based on system Vitek 2, API 20C AUX, API ID 32C(bioMérieux@) and the susceptibility test on Vitek 2 and E test.

**Results:** During the last two years, 1/10/2015-30/9/2017, in 2359 ICU patients, 27884 patient days, we detected Trichosporon spp in 31patients. The minimum stay was 28 days. 24(77,4%) men and 7(22,6%) women. 27/31(87%) strains were T. Asahii and 4(13%) T. mucoides. All patients were exposed to antibiotics, had medical devices and other infections with other multi drug resistant strains. In 10 patients the infection persisted over one month, and 2 patients died during Trichosporon spp. funguria. According to the antifungal susceptibility testing, 3 strains *T. Asahii* had intermediate sensitivity (MIC:16:I) and 3 strains were resistant (MIC:16:R) to Fluconazole.. All strains *T. Asahii* were sensitive to Amphotericin-B. All *T. mucoides* were sensitive to Flucytosine. 2/4 (50%). *T. mucoides* were resistant to voriconazole(MIC:4 R), 2/4(50% R) to Amphotericin-B (MIC:8-16 R), and 1/4(25%) to Fluconazole (MIC:32 R). The echinocandins are not active against Trichosporon spp.. In most patients treatment was administration of voriconazole.

**Conclusions:** The Trichosporon spp after 28 days in ICU percentage is 31/2359(1,31%) and the incidence is 31/2788(1.11%). The majority were men. The azole drugs and Amphotericin-B showed activity against Trichosporon spp but recommendations must be based on in vitro susceptibility data and clinical experience and features.

## P071 *Acinetobacter baumannii* ventilator-associated pneumonia epidemiology, risk and prognosis factors

### W Sellami, W Essmat, I Ben mrad, Z Hajjej, H Gharssallah, I Labbene, M Ferjani

#### Military Hospital, Tunis, Tunisia

**Introduction:** Ventilator-associated pneumonia (VAP) is the most common nosocomial infection in critically ill patients, reaching up to 30 to 50%, with a high mortality rate. *Acinetobacter baumannii* (AB) has emerged as a pathogen frequently incriminated in VAP’s in Tunisia. The aim of this study was to describe the epidemiological characteristics of *Acinetobacter baumannii* VAP, to identify the risk factors and the predictors of poor outcome of VAP with AB.

**Methods:** A retrospective study was conducted in the intensive care unit of the Military Hospital of Tunis, from January 2015 to December 2016. All patients with VAP’s documented infection were included. VAP’s patients with AB vs VAP’s patients due to other pathogens.

**Results:** Seventy patients (10%) developed VAP. The incidence of VAP with AB was 6.28%. Previous antibiotic therapy was identified as a risk factor for *Acinetobacter baumanii*-induced pneumonia, unlike the underlying disease. AB was resistant to ceftazidime in 100%, imipenem in 97.5% with sensitivity to colistin in 100% of cases. Multidrug-resistant AB accounted for 22.5% and highly resistant AB accounted for 77.5%. Patients with AB pneumonia were more frequently complicated by acute respiratory distress syndrome compared to other patients (37.5% versus 8.9%, p = 0.02), leading to higher mortality (52.5% versus 20%, p = 0.02).

**Conclusions:** The increasing incidence of VAP in multidrug-resistant and highly resistant AB predicts a high morbidity and mortality. Hence, the risk factors related to poor outcome in VAP’s need to be identified. The implementation of infection-control measures, mainly the cross-transmission, may be needed to improve outcome.

## P072 Single versus combination antibiotic therapy in the treatment of gram negative infections

### S Chatterjee ^1^, A Bhakta^1^, J Basak^2^, S Todi^1^

#### ^1^AMRI Hospitals, Kolkata, India,^2^Jadavpore University, Kolkata, India

**Introduction:** This study assessed whether empiric combination antibiotic therapy directed against Gram-negative bacteria is associated with lower intensive care unit (ICU) mortality compared to single antibiotic therapy.

**Methods:** Retrospective cohort study on prospectively collected data conducted in the ICU of a tertiary care hospital in India between July2016 to March2017. All consecutive infection episodes treated with empiric antibiotic therapy and with subsequent positive culture for Gram-negative bacteria were included. Primary and secondary outcomes were all cause ICU mortality and ICU length of stay (LOS). Outcomes were compared between infection episodes treated with single vs.combination antibiotic therapy.

**Results:** Of total 214 episodes of gram-negative infections 66.4% received combination-antibiotic therapy. Baseline demographic and clinical characteristics between single vs. combination therapy groups were similar (mean age: p=0.07; sex: p=0.3; mean APACHE IV score: p=0.07). Overall ICU mortality did not significantly differ between single and combination antibiotic groups (30.2% vs. 27%; p=0.7). In single antibiotic group, ICU mortality was significantly higher for antibiotic-resistant compared to antibiotic-sensitive bacteria (77.8% vs. 18.5%, p=0.0002). In combination group, significantly lower ICU mortality was noted if bacteria was sensitive to even one antibiotic compared to pan-resistant bacteria (21.4% vs. 63.6%, p=0.0001). ICU LOS was similar between antibiotic-sensitive bacteria and antibiotic-resistant bacteria, both in single and combination therapy groups (single, antibiotic-sensitive vs. antibiotic-resistant: mean LOS±SD 14.6±12.7 vs.12.8±11days; p=0.6; combination, antibiotic-sensitive vs. antibiotic-resistant: 15.5±13.3 vs.11.2 days; p=0.1).

**Conclusions:** Irrespective of the number of antibiotics prescribed as empiric therapy, outcome of patients solely depends on the sensitivity pattern of the bacteria isolated.

## P073 Pharmacokinetics of trimethoprim and sulfametrole in critically ill patients on continuous haemofiltration

### R Welte^1^, J Hotter^1^, T Gasperetti^1^, R Beyer^1^, R Bellmann-Weiler^1^, S Eschertzhuber^1^, M Zaruba^1^, I Lorenz^1^, M Ströhle^2^, M Joannidis^1^, R Bellmann^1^

#### ^1^Medical University of Innsbruck, Innsbruck, Austria,^2^General Hospital of Innsbruck, Innsbruck, Austria

**Introduction:** The combination of trimethoprim and sulfametrole (TMP-SMT, Rokiprim®) is active against multi-drug resistant bacteria and Pneumocystis jirovecii. In critically ill patients undergoing continuous veno-venous haemofiltration (CVVH), however, its use is limited because of lacking pharmacokinetic data.

**Methods:** Pharmacokinetics of both drugs were determined after standard doses in patients on CVVH and in critically ill patients with approximately normal renal function. Quantification of TMP and SMT was done by high pressure liquid chromatography (HPLC) and UV detection after pre-purification by solid phase extraction. The total clearance (CLtot) was estimated from arterial plasma levels and the haemofilter clearance (CLHF) from plasma and ultrafiltrate concentrations.

**Results:** Six patients on CVVH (3 after the first dose, 3 at steady state) and nine patients off CVVH have been enrolled (4 after first dose, 7 at steady state). After a single dose, CLtot of SMT was 3.5 (1.8-3.8, median [range]) and 1.7 (1.1-2.7) L/h on and off CVVH, respectively. At steady state, we observed a CLtot of 1.0 (0.5-1.0) and 0.3 (0.2-0.9) L/h, respectively, on and off CVVH. Steady state trough levels (Cmin) of SMT amounted to 52-113 mg/L in patients on CVVH and 18-145 in patients off CVVH. CLtot of TMP was 4.4 (2.5-5.3) L/h on CVVH and 5.4 (3.2-9.9) L/h off CVVH after the first dose. At steady state, its CLtot amounted to 0.8 (0.4-0.8) and 1.0 (0.6-1.9) L/h on and off CVVH, respectively. Cmin was 4-12 mg/L on CVVH and 3-9 mg/L in patients off CVVH. CLHF accounted for 22-68% of CLtot of SMT and 28-72% of CLtot TMP.

**Conclusions:** Exposure to both antimicrobial agents is highly variable, but comparable in patients on and off CVVH. As considerable amounts of SMT and TMP are eliminated by CVVH, no excessive accumulation appears to take place during treatment with standard doses.

## P074 The positive impact of meropenem stewardship intervention at a Brazilian intensive care unit

### W Freitas^1^, M Davi^1^, L Souza^1^, M Couto^1^, L Lourenço^1^, R Eiras^1^, H Primo^1^, J Páramo^1^, J Garcia^1^, M Alves^2^

#### ^1^Hospital Casa de Portugal, Rio de Janeiro, Brazil,^2^Universidade Federal de Juiz de Fora, Juiz de Fora, Brazil

**Introduction:** This study aimed to evolutionary analyze the decrease of the Meropenem Defined Daily Doses (DDD), at a Brazilian Intensity Care Unity (ICU) before and after six months of the antimicrobial stewardship intervention.

**Methods:** From June to November 2017, the meropenem use to treat inpatients at the Hospital Casa de Portugal ICU, Rio de Janeiro, Brazil, was reduced to seven days and Meropenem DDD, CRE ID, consumption of saline 100 mL for dilution, equipment for infusion of antibiotics, and global mortality values and BSI deaths were noted. Same data were retrospectively collected from December 2016 to May 2017. Results of both periods were analyzed by Student’s T Test.

**Results:** Meropenem DDD values ranged from 143.01 to 187.70 and 22.87 to 160.76, from December 2016 to May 2017 and June to November 2017, respectively, with average of 174.7 and 101.6 in this order, with T(10) of 3.60 (p = 0.005). CRE ID also decreased, with values ranging from 9.6 to 34.9 (December 2016 to May 2017) and 1 to 18.1 (June to November 2017), with average of 16.2 and 7.2, respectively. A decrease in the use of saline for dilution by 1000 patients-day with values from 2930 to 4436 and 2619 to 2980 from December 2016 to May 2017 and from June to November 2017 respectively, with 3310 and 2868 (average) in this order was detected. We have observed concomitant decrease in equipment for infusion by 1000 patients-day with values from 995 to 2116 and from 918 to 1054, from December 2016 to May 2017 and from June to November 2017, respectively, with average values of 1236 and 967, in this order. The global mortality values varied of 17.41 to 22.96 (December 2016 to May 2017) and 11.61 to 17.9 (June to November 2017), with average of 19.5 and 14.5 respectively, with T(10) of 3.78 (p = 0.004). A drop in the mean number of BSI deaths from December 2016 to May 2017 and June to November 2017 of 2.6 to < 1, in this order, was also observed.

**Conclusions:** Meropenem stewardship intervention had a positive impact in the Intensive Care Unity evaluated.

## P075 Colistin ‘MIC’ creep, a harbinger for resistance? Study for monitoring antimicrobial resistance trends (SMART) - South Africa 2015-2016

### B Magazi, R Holl

#### MSD, Midrand, South Africa

**Introduction:** Loss of colistin as a clinical option has profound public health implications. Widespread use of colistin in agriculture and humans has seen the emergence of Mcr-1 mediated resistance amongst South African patients [1]. We sought to describe the trends of colistin minimum inhibitory concentrations (MIC) over two years using data collected by SMART.

**Methods:** SMART monitors the in vitro susceptibility of clinical aerobic and facultative gram-negative bacterial isolates to selected antimicrobials of importance, enabling longitudinal analyses to determine changes over time. The dataset comprised bacterial isolates from four different South African private pathology laboratories and one public sector pathology laboratory from 2015 - 2016. The methods used in the study have been described elsewhere [2]. Isolate proportions between years were compared using the chi-squared test with Yates’ continuity correction.

**Results:** 146 and 198 *Pseudomonas aeruginosa* isolates were called in 2015 and 2016 respectively. Isolates with MICs=2 increased from 4.8% to 14.6 % between 2015 and 2016 (p=0.00278) (Table 1). In both years none of the isolates had an MIC>2. Non-significant changes were observed in *Klebsiella pneumoniae* and *Escherichia coli*.

**Conclusions:** While the clinical and public health significance of this MIC creep amongst *P. aeruginosa* is unknown, it is disconcerting. Could this be similar to the precipice witnessed in the hVISA phenotype in *Staphylococcus aureus* [3]? Further studies are required to elucidate this. Meanwhile, we call for more prudent use of this agent and the development of colistin sparing treatment options for *P. aeruginosa*.

**References**

1. Coetzee J et al. 106:449-450, 2016

2. Morrissey I et al. Pharmaceuticals. 6(11):1335-1346, 2013

3. McGuinness WA et al. Yale Bio Med 90(2):269-281, 2017

**Table 1 (abstract P075).**
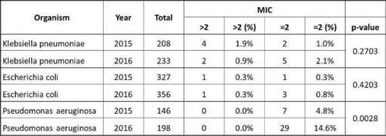
Colistin MIC Levels 2015-2016

## P076 Ceftazidime/avibactam for treating severe infections in the critically ill due to carbapenemases producing Klebsiella pneumoniae

### A Corona, A Veronese, S Antinori, S Santini, C Soru, M Corbellino, F Cantarero, E Catena

#### ASST Fatebenefratelli Sacco, PO SACCO - Milano, Milano, Italy

**Introduction:** Carbapenemases producing (cp) Klebsiella pneumoniae (KP) infection rate ranges between 5-50% and is associated with a high mortality (19-51%). The use of ceftazidime-avibactam - a 3-generation cephalosporin plus a new inhibitor of class A (KPC), C (AmpC) and D (OXA48) ESBL, may resolve life-threatening conditions.

**Methods:** Case-report of 14 patients undergoing compassionate treatment.

**Results:** From April to November 2017, 14 patients (10 M and 4 F), median age 57 (IQR = 42.5-70.5) were given ceftazidime/avibactam for major KP-cp (meropenem MIC > 16) infections: (i.e. 9 bacteramia 3 secondary peritonitis and 2 UTI. 10/14 (71.5%) patients, developed a septic shock [median (IQR) SOFA score 10 (8-17)) and needed mechanical ventilation [median (IQR) 8 (4-17) days], norepinephrine infusion [median (IQR) 3 (2-5) days]; 4 patients underwent renal replacement therapy. The median treatment duration (IQR) was 14 (13-14) days. In 41.6% of cases, antibiotic-therapy therapy combination (phosphomycin and colistin) was chosen. All the patients experienced a clinical response by 72/96 hours from the ceftazidime/avibactam commencing. In 8/9 bacteraemic patients negativization of blood culture occurred by 96 hours as well as of the rectal swab in 5/14 patients. A (B) recurred and a second treatment was given. 11/14 (78.5%) patients survived, whereas death was caused by multi-organ failure. The susceptibility test of strains showed sensitivity to ceftazidime/avibactam, whereas 100% of resistance to carbapenems, quinolones and III/IV generation cephalosporin, tigecycline and piperacillin/tazobactam; 62.5% of susceptibility to fosfomycin and colistin; (v) less than 50% of suceptibility to aminoglicosides.

**Conclusions:** The 14 strains of KP-cp were susceptible to ceftazidime-avibactam despite the high carbapenem-resistance recorded in our ICU, because od rare identification of KP-cp VIM/NDL +. The preliminary data seems to confirm the efficacy and clinical utility of this antibiotic for the critically ill patients.

## P077 Phage-based therapy against Acinetobacter baumannii lung infection in mice

### SM Wienhold^1^, MC Brack^1^, C Rohde^2^, G Nouailles^1^, C Seitz^3^, A Ross^3^, H Ziehr^3^, K Dietert^4^, C Gurtner^4^, O Kershaw^4^, AD Gruber^4^, M Rohde^5^, N Suttorp^1^, M Witzenrath^1^

#### ^1^Charité – Universitätsmedizin Berlin, corporate member of Freie Universität Berlin, Humboldt-Universität zu Berlin, and Berlin Institute of Health, Berlin, Germany,^2^Leibniz Institute DSMZ - German Collection of Microorganisms and Cell Cultures, Braunschweig, Germany,^3^Fraunhofer Institute for Toxicology and Experimental Medicine (ITEM), Braunschweig, Germany,^4^Freie Universität Berlin, Berlin, Germany,^5^Helmholtz Centre for Infection Research, Braunschweig, Germany

**Introduction:** Multidrug resistant bacteria (MDR) are an increasing problem on intensive care units. Lung infections caused by *Acinetobacter baumannii* are frequently difficult to treat. Phages have regained attention as treatment option for bacterial infections due to their specificity and effectivity in lysis. The aim of this preclinical study was to determine efficacy and safety of a novel phage preparation in mice.

**Methods:** Mice were transnasally infected with a MDR *A. baumannii* strain [1] and 12 hours later treated intratracheally with a specific phage or solvent. Phage Acibel004 [2] was produced as suspension including efficient depletion of endotoxins. At defined time points, clinical parameters, bacterial burden in lung and bronchoalveolar lavage fluid (BALF) and cell influx were determined. Further, lung permeability and cytokine release were quantified and histopathological examination was performed.

**Results:** Mice treated with phages recovered faster from infection-associated hypothermia. 48 hours after infection, phage treatment led to a reduction in bacterial loads in lungs and BALF. In addition, lung permeability and cytokine production were reduced in phage-treated mice. Histopathological examination of the lungs showed less spreading of bacteria to the periphery in phage-treated mice, whereas cellular recruitment into the lung was unaffected. No adverse effects were observed.

**Conclusions:** For the first time a highly purified phage against *A. baumannii* was successfully used in vivo. The current preclinical data support the concept of a phage-based therapy against pulmonary *A. baumannii* infections.

**References**

1. Knapp S, et al. Am J Respir Crit Care Med. 1;173(1):122-9, 2006

2. Merabishvili M, et al. PLoS One. 11;9(8):e104853, 2014

## P078 Efficacy of phage therapy against lethal methicillin resistant *Staphylococcus aureus* (MRSA) ventilator associated pneumonia in rats (VAP)

### J Prazak^1^, P Reichlin^2^, D Grandgirard^1^, G Resch^3^, M Qiao^1^, S Nansoz^1^, Y Que^1^, M Haenggi^1^

#### ^1^Inselspital, University Of Bern, Bern, Switzerland,^2^Medical School, University of Bern, Bern, Switzerland,^3^University of Lausanne, Lausanne, Switzerland

**Introduction:** VAP is common in critically ill patients and associated with high morbidity and mortality, especially when caused by antibiotic resistant bacteria. Recently, phage therapy has emerged as a promising non-antibiotic based treatment of antibiotic resistant bacterial infections. However, proof-of-concept experimental and clinical studies are missing before its wider use in clinical medicine. The goal of this experimental study was to compare the efficacy of phage therapy versus antibiotics for the treatment of MRSA in a rat model of VAP.

**Methods:** Four hours after intubation and protective ventilation, rats were inoculated via the endotracheal tube with 6-9 x 10^9^ CFU (LD100) of the MRSA clinical isolate AW7. The animals were subsequently extubated. Two hours after bacterial challenge, rats were randomised to receive intravenously either teicoplanin (n= 8), a cocktail of 4 lytic anti-S. aureus bacteriophages (n=9) or combination of both (n=6). 5 animals served as control (no treatment). Survival by 96 hours was the primary outcome. Secondary outcomes were bacterial count in lungs, spleen and blood. Kaplan-Meier estimates of survival were done and multiple comparisons of survival rates performed using the Holm-Sidak method.

**Results:** Treatment with either phages, antibiotics or combination of both significantly increased survival (66%, 50%, 50% respectively, compared to 0% survival for controls, p<0.05). There were no statistical differences in survival rates between either forms of treatment (Fig. 1). Treatments hinder the systemic extension of the infection into the blood and spleen without impacting bacterial counts within the lungs, but the numbers are too small to perform statistical tests (Table 1).

**Conclusions:** Phage therapy performed as well as teicoplanin for the treatment of MRSA VAP when administered intravenously. Further experiments are needed to investigate whether phage administered via aerosols could heal infection within the lungs.

**Fig. 1 (abstract P078).**
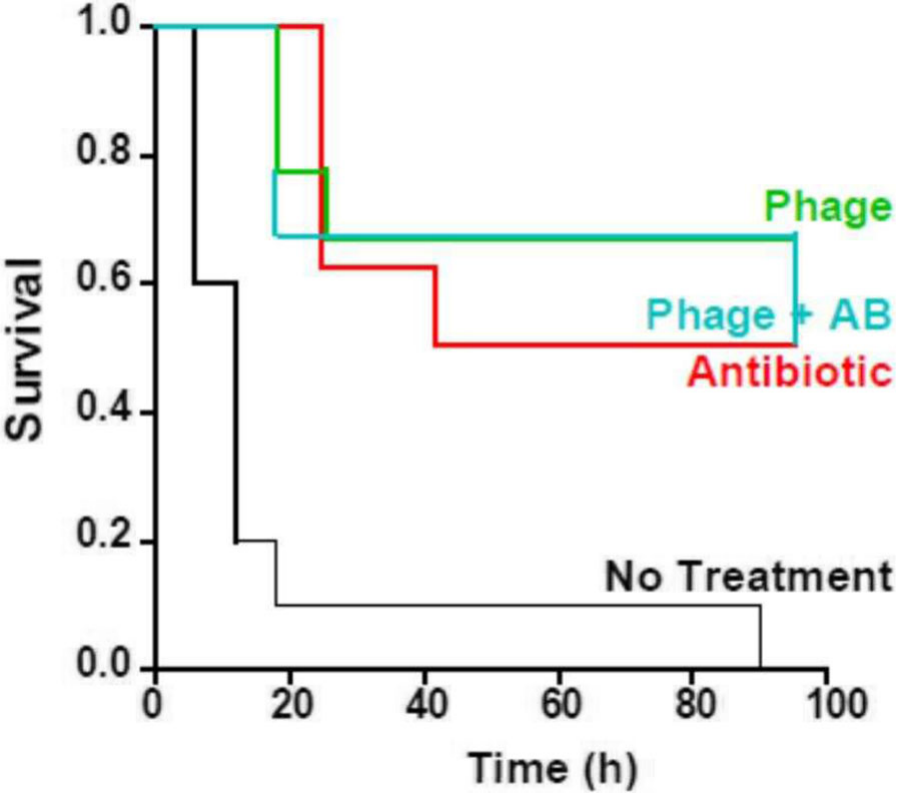
Effect of treatment with phages, antibiotics or combination of both on survival rate

**Table 1 (abstract P078).** Bacterial count in lung, spleen and blood at euthanasia. Median [IQR]

GROUP	cfu lung (log10 cfu/g tissue)	cfu spleen (log10 cfu/g tissue)	cfu blood (log10 cfu/ml blood)
antibiotic	4.05 [3.58 - 5.03] n: 8	0 [0 - 1.81] n: 8	6.51 [0 - 44.64] n: 6
antibiotic + phages	4.94 [4.31 - 6.91] n: 6	0 [0 - 0] n: 6	0 [0 - 0] n: 3
phages	5.88 [3.84 - 7.01] n: 9	0 [0 - 3.52] n: 9	0 [0 – 41.76] n: 7
control	4.26 [3.76 - 4.80] n: 5	4.51 [0 - 5.19] n: 5	34.77 [34.77 - 34.77] n: 2

## P079 Influence of amikacin inhalation on the efficacy of ventilation-associated pneumonia and ventilation-associated tracheobronchitis treatment caused by multi-drug resistant gram-negative bacteria: comparative study

### A Yaroshetskiy^1^, N Rezepov^2^, I Mandel^3^, V Khodak^4^, V Konanykhin^3^

#### ^1^Pirogov Russian National Research Medical University, Moscow, Russia,^2^City Clinical Hospital ^1^ 67, Moscow, Russia,^3^Sechenov University, Moscow, Russia,^4^City Hospital ^1^35, Nigniy Novgorod, Russia

**Introduction:** The aim of the study was comparative evaluation of the clinical and microbiological efficacy of combination of amikacin thru nebuliser Aeroneb Pro and standard antimicrobal therapy (AMTcomb) with standard antimicrobal therapy (AMTst) in treatment of ventilator-associated pneumonia (VAP) and ventilator-associated tracheobronchitis (VAT) caused by multi-drug resistant gram-negative bacteria.

**Methods:** In prospective two-center study with retrospective control included patients with VAP and VAT. In AMTst group (retrospective, n=25) we used combination of meropenem 1 g every 8h iv as continuous infusion, cefoperazon/sulbactam 4 g every 12 h iv as continuous infusion and amikacin 1 g iv every 24 h. In AMTcomb group (prospective, n=25) we used combination of AMTst and amikacin inhalation 500 mg every 12 h thru nebuliser Aeroneb Pro.

**Results:** In AMTcomb clinical cure rate was 84%, while in AMTst 29.2% (p<0.001), Clinical Pulmonary Infection Score (CPIS) on day 7 was 6 (4-7) points in AMTst and 2 (0-4) points in AMTcomb (p<0.001). Recurrence of VAP/VAT was 29.2% in AMTst and 12.5% in AMTcomb (p=0.008). On day 7 infectious agent titer in tracheal aspirate was 10^7^ (10^3^-10^8^) CFU/ml in AMTst group, while 10^3^ (no growth-10^6^) CFU/ml in AMTcomb (p=0.016). Microbiological eradication observed in 13 patients in AMTcomb vs in 1 patient in AMTst and microbiological persistance observed in 6 patients in AMTcomb vs 17 patients in AMTst (p=0.002). In AMTcomb on 3rd day sputum was less purulent (p=0.016). Amikacin nebulisation didn’t led to deterioration of organ dysfunction: on day 7 there was no difference in platelet count, creatinine and bilirubin levels as compared to day 0 (p=0.102; p=0.297, p=0.532, respectively).

**Conclusions:** Addition of amikacin inhalation 500 mg every 12 h thru Aeroneb Pro nebuliser in patients with VAP and VAT was more efficacious than intravenous standard antimicrobal treatment with comparable safety profile.

## P080 Aerozolized colistin is an effective adjunct to systemic antibiotics in ventilator-associated pneumonia

### A Kuzovlev, A Shabanov, A Goloubev, V Moroz, A Grechko

#### Federal Research and Clinical Center of Intensive Care Medicine and Rehabilitology, V.A. Negovsky research institute of general reanimatology, Moscow, Russia

**Introduction:** The aim of the study was to assess the effectiveness of inhaled colistin (IC) as an adjunct to systemic antibiotics in the treatment of ventilator-associated pneumonia (VAP).

**Methods:** 110 ICU patients with VAP were enrolled in this observational study. Resolution of VAP was assessed as primary endpoint; eradication of pathogens in sputum, weaning time, duration of ICU stay and mortality were assessed as secondary outcomes. Patients were split into 2 groups: Gr.1 (n = 60) - addition of IC to systemic antibiotics without changing the basic regimen; Gr. 2 (n = 50) - change in systemic antibiotics according to sensitivity. Groups were comparable. IC was administered in a dose of 2 million IU TID (Xselia Pharmaceuticals ApS, Denmark). Statistical analysis was performed using Statistica 7.0 (M, σ, Newman-Keuls test; p <0.05).

**Results:** VAP resolution rate was 77% in Gr.1 (vs. 50% in Gr. 2, p = 0.0295); eradication of pathogens from sputum by the 7th day. treatment was achieved in 80% of Gr. 1 and 60% in the Gr. 2 (n = 12) (p> 0.05); in Gr. 1 weaning from ventilation was possible earlier than in Gr. 2 - 7.8±1.3 days. in Gr. 1 vs. 10.9±4.5 days. in Gr. 2 (p = 0.0000); in Gr. 1 duration of ICU stay was shorter than in Gr. 2 - 11.5±3.2 days vs. 17.1±2.3 days. in Gr. 2 (p = 0.0000). No mortality differences were detected.

**Conclusions:** Administration of inhaled colistin 2 million IU TID is effective as an adjunct to systemic antibiotics in the treatment of VAP. This modified treatment promotes a more rapid resolution of VAP, earlier weaning from ventilator, reduction of the duration of ICU stay, with no impact on mortality. The addition of IC to systemic antibiotics should be considered as second-line regimen in VAP patients.

## P081 Factors associated with no de-escalation of empirical antimicrobial therapy in ICU settings with high rate of multi-drug resistant bacteria

### C Routsi ^1^, K Arvaniti^1^, G Poulakou^1^, A Tourtoglou^1^, A Goufa^1^, A Vemvetsou^1^, A Kanavou^1^, E Hasou^1^, E Antoniadou^1^, C Nikolaou^1^, K Ntorlis^1^, S Kokkoris^1^, V Theodorou^1^, S Vasilliagkou^2^, H Giamarellou^2^

#### ^1^National and Kapodistrian University of Athens, Athens, Greece,^2^Hellenic Society of Chemotherapy Study Group, Athens, Greece

**Introduction:** De-escalation is recommended in the management of antimicrobial therapy in ICU patients [1]. However, this strategy has not been adequately evaluated in the presence of increased prevalence of multidrug-resistant (MDR) bacteria. The aim of this study was to identify factors associated with no de-escalation in ICUs with high rate of MDR bacteria [2].

**Methods:** Prospective, multicenter study conducted in 12 Greek ICUs over a 1-year period. Patients with laboratory confirmed infections were included. SOFA score on admission, on septic episode and thereafter every 24 h over 14 days, infection site(s), culture results, antimicrobial therapy, and mortality were recorded. Only the first septic episode was analyzed. In order to assess the factors associated with no de-escalation, a multivariate analysis was performed.

**Results:** A total of 211 patients (admission SOFA score 10±3) were analyzed. 43% of those had septic episode on ICU admission; 57% patients had an ICU-acquired. De-escalation was applied to 44 (21%) patients whereas it was not feasible in 75 patients (44 %) due to the recovery of MDR pathogens or it was not applied, although the microbiology results allowed it, in 92 patients (56 %). Septic shock on the day of septic episode was present in 67% and 79% of patients with and without de-escalation, respectively, p=0.072). Compared to no de-escalation, de-escalation strategy was associated with a shorter duration of shock (4±5 vs. 9±7 days, p<0.001) and all-cause mortality (15.4% vs. 46.4%, p<0.001). Multivariate analysis showed that the variables associated with no de-escalation were: a deteriorating clinical course as indicated by an increasing SOFA score (OR 14.7, p<0.001) and a lack of de-escalation possibility due to recovery of MDR pathogens (OR 27.3, p=0.008).

**Conclusions:** Deteriorating clinical course and MDR pathogens are independently associated with no de-escalation strategy in critically ill patients.

**Reference**

1. Rhodes A et al. Intensive Care Med 43:304, 2017

2. Dimopoulos G et al. Minerva Anestesiol 81:405, 2015

## P082 Corticosteroid treatment in patients with severe influenza pneumonia: a propensity score matching analysis.

### G Moreno^1^, A Rodríguez^1^, LF Reyes^2^, J Sole-Violan^3^, E Díaz^4^, M Bodí^1^, S Trefler^1^, J Guardiola^5^, JC Yébenes^6^, A Soriano^7^, I Martín-Loeches^8^, MI Restrepo^2^

#### ^1^Hospital Universitari Joan XXIII, Tarragona, Spain,^2^University of Texas Health at San Antonio, San Antonio, TX, USA, ^3^Hospital Dr. Negrín, Gran Canaria, Gran Canaria, Spain,^4^Hospital Parc Tauli, Sabadell, Spain,^5^University of Louisville and Robley Rex VA Medical Center, Louisville, Kentucky, USA,^6^Hospital de Mataró, Mataró, Spain,^7^Hospital Clinic, Barcelona, Spain,^8^Multidisciplinary Intensive Care Research Organization (MICRO). St James’s University Hospital. Trinity Centre for Health Sciences, Dublin, Ireland

**Introduction:** Patients with influenza infection could develop acute respiratory failure and ultimately ARDS. Corticosteroids have been broadly used as immunomodulatory despite lack of evidence supporting its effect in patients with influenza infection. Therefore, our aim was to determine the clinical predictors associated with corticosteroid administration and its association with ICU mortality.

**Methods:** This is a secondary analysis of a prospective cohort study of critically ill patients with confirmed influenza pneumonia admitted to 148 ICUs in Spain, between June 2009 and April 2014. Patients were stratified according to treatment with systemic corticosteroids when administrated within the first 24-hours of hospital admission. We use a propensity-score matching (PSM) analysis to reduce confounding factors and association of the administration of corticosteroids. Primary outcome was ICU mortality. Cox proportional hazard analysis was performed to investigate the association between baseline characteristics and steroid use and used ICU mortality as the dependent variable.

**Results:** The total population comprised 1,846 patients with H1N1 pneumonia, corticosteroids were administered in 604(32.7%) patients, being methylprednisolone the most frequently used medication(578/604 [95.7%]). The median daily dose was equivalent to 80 mg(IQR60-120) for a median duration of 7 days(IQR 5-10). Asthma, COPD, hematological disease and requirement of mechanical ventilation were independently associated with corticosteroid use(p<0.05). After adjusting with PSM, patients with H1N1 pneumonia who received corticosteroids had a higher ICU mortality(27.5%vs.18.8%, HR 1.44,1.18-1.76, p=0.005) compared to patients not treated with corticosteroids (Fig. 1).

**Conclusions:** Administration of corticosteroids in patients with severe influenza pneumonia was associated with an increased ICU mortality. Thus, we advocate caution to physician using these medications for influenza pneumonia.

**Reference**

Díaz E et al. J Infect 64(3):311-8, 2012

**Fig. 1 (abstract P082).**
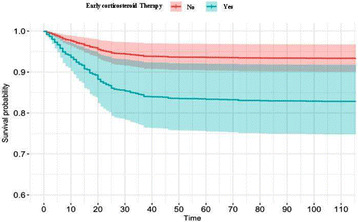
Cox hazard Regression analysis

## P083 Tracheostomy dependence increases the risk of medical emergency team activation in pediatric patients

### B McKelvie^1^, A Lobos^2^, J Chan^2^, F Momoli^2^, J McNally^2^

#### ^1^London Health Sciences Centre - Children’s Hospital, London, Canada,^2^Children’s Hospital of Eastern Ontario, Ottawa, Canada

**Introduction:** The purpose of this study was to determine if pediatric inpatients with tracheostomies (PTIs) were at increased risk of medical emergency team (MET) activation compared to other ward patients. The requirement for a tracheostomy confers a significant risk for morbidity and mortality [1]. METs have been implemented to improve the detection and management of patients at risk for clinical deterioration. Based on their risk factors, we hypothesized that PTIs would be at higher risk of clinical deterioration than other patients and so would have higher MET activation rates.

**Methods:** This retrospective cohort study was conducted at a tertiary pediatric hospital, Children’s Hospital of Eastern Ontario (CHEO), in Ottawa, Canada. PTIs were identified using lists from subspecialty services, decision support and the operating room. MET activation data was obtained from a prospectively maintained database.

**Results:** From 2008 to 2014 there were 42,041 admissions, including 264 involving PTIs. MET activations occurred in 1260 distinct admissions, including 33 PTI admissions. In the PTI group, the MET activation rate was significantly higher when compared to other ward patients (14 vs 2.9 per 100 admissions, p<0.001) and they had a 5.7 times increased odds of MET activation (OR 5.7, CI: 95% CI 3.6 to 9.1). Almost all PTI patients required intervention during the MET activation (94.6%) and 21.6% were admitted to PICU.

**Conclusions:** PTIs are at significantly higher risk for MET activation. After MET activation, almost 80% of PTIs remained on the wards, which suggests that the MET helped manage deterioration in these patients. Targeted strategies need to be developed to reduce the risk of PTIs on the wards, and based on our results the MET may play a central role in improving care for these patients.

**Reference**

1. Watters K et al. Laryngoscope 126:2611–2617, 2016

## P084 qSOFA versus SIRS versus SOFA for predicting sepsis and adverse outcomes of patients in the intensive care unit. Preliminary report of Russian national study

### M Astafeva^1^, V Rudnov^1^, V Kulabukhov^2^, V Bagin^1^

#### ^1^City Clinical hospital №40, Yekaterinburg, Russia,^2^AV Vishnevsky Institute of Surgery, Moscow, Russia

**Introduction:** In 2016 sepsis is defined as a life-threatening organ dysfunction caused by infection. This concept, called Sepsis 3 [Singer M at al], define only two stages – sepsis and septic shock. In addition, it is defined that quick SOFA (qSOFA) may be a better predictor of in-hospital mortality than the SOFA score [Seymour CW et al]. However, the possibility of application of the Sepsis-3 and qSOFA conception for use in low/middle-income countries is still unknown.

**Methods:** The aim of our study was to define the role of qSOFA, SIRS and SOFA for predicting sepsis and adverse outcomes of patients in the ICUs in Russia. Design: prospective observational multicenter study. We made ROC-analysis to estimate sensitivity, specificity, and predictive value of SIRS, qSOFA, and SOFA to identify sepsis and predict ICU-mortality.

**Results:** The study included 335 patients from 22 ICUs in Russia. Sepsis, according to the Sepsis 3 criteria, was identified in 179 (53%) patients, and septic shock – in 82 (24%) patients. 102 (30%) patients died within ICU stay. In the prognosis of sepsis the qSOFA scale, SIRS and SOFA demonstrated the following AUCROCs: 0.683 (95% CI 0.626-0.736); 0.719 (95% CI 0.674 to 0.764); 0.763 (95% CI 0.711 - 0.816) respectively (only the AUCROCs of qSOFA vs SOFA differ significantly, p<0.01). In the prognosis of mortality the qSOFA scale, SIRS and SOFA demonstrated the following AUCROCs: 0.736 (95% CI 0.682-0.791); 0.594 (95% CI 0.546-0.642); 0.843 (95% CI 0.798-0.889) respectively (all AUCROCs significantly differ from each other, p<0.01). For the break-point of the qSOFA score >1 in the prognosis of mortality, the specificity was 65.2%; the sensitivity was 70.6%.

**Conclusions:** The qSOFA scale in the prognosis of sepsis does not differ significantly from the SIRS criteria, but in the prognosis of mortality is significantly better than SIRS. qSOFA significantly worse in the prognosis of sepsis and death than the SOFA scale.

## P085 Detection of sepsis by qSOFA and SIRS in patients with infection in general wards

### J Luo, W Jiang, L Weng, J Peng, X Hu, C Wang, G Liu, H Huang, B Du

#### Peking Union Medical College Hospital, Beijing, China

**Introduction:** The international task force of Sepsis-3 introduced the quick Sequential Failure Assessment (qSOFA) score to supersede the systemic inflammatory response syndrome (SIRS) score as the screen tool for sepsis. The objective of this study is to prospectively access the diagnostic value of qSOFA and SIRS among patients with infection in general wards.

**Methods:** A prospective cohort study conducted in ten general wards of a tertiary teaching hospital. For a half-year period, consecutive patients who were admitted with infection or developed infection during hospital stay were included. Demographic data and all variables for qSOFA, SIRS and SOFA scores were collected. We recorded daily qSOFA, SIRS and SOFA scores until hospital discharge, death, or day 28, whichever occurred earlier. The primary outcome was sepsis at 28 days. Discrimination was assessed using the area under the receiver operating characteristic curve (AUROC) and sensitivities or specificities with a conventional cutoff value of 2.

**Results:** Of 409 patients (median age, 55 years [IQR, 40-67]; male, 225[55%]; most common diagnosis pneumonia, 234[57%]) who were identified with infection in general wards, 229(56%) developed sepsis at a median of 0 (IQR, 0-1) day, 146 patients (36%) and 371 patients (91%) met qSOFA and SIRS criteria at a median of 1 (IQR, 0-5) and 0 (IQR, 0-0) day, respectively. The qSOFA performed better than SIRS in diagnosing sepsis, with an AUROC of 0.75 (95% CI, 0.71-0.79) vs 0.69(95% CI, 0.64-0.74). With a conventional cutoff value of 2, qSOFA had lower sensitivity (53% [95% CI, 47%-60%] vs. 98% [95% CI, 95%-99%], p < 0.001) and higher specificity (87% [95% CI, 81%-91%] vs. 18% [95% CI, 13%-23%], p < 0.001) than SIRS (Table 1).

**Conclusions:** Among patients with infection in general wards, the use of qSOFA resulted in greater diagnostic accuracy for sepsis than SIRS during hospitalization. qSOFA and SIRS scores can predict the occurrence of sepsis with high specificity and high sensitivity, respectively.

**Table 1 (abstract P085).** Diagnostic performance of qSOFA and SIRS scores for sepsis-3

	qSOFAmax	SIRSmax	SIRSmax
Cutoff value	2	2	3
Sensitivity, % (95% CI)	53 (47-60)	98 (95-99)	86 (81-90)
Specificity, % (95% CI)	87 (81-91)	18 (13-23)	43 (36-51)
Positive Predictive value, % (95% CI)	84 (77-89)	60 (55-65)	66 (60-71)
Negative Predictive value, % (95% CI)	59 (53-65)	86 (71-95)	71 (61-79)
Positive Likelihood ratio (95% CI)	4.0 (2.7-5.9)	1.2 (1.1-1.3)	1.5 (1.3-1.7)
Negative Likelihood ratio (95% CI)	0.5 (0.5-0.6)	0.1 (0.1-0.3)	0.3 (0.2-0.5)

## P086 Prognostic accuracy of quick sequential organ failure assessment (qSOFA) score for mortality: systematic review and meta-analysis

### B Brandao Barreto^1^, M Luz^2^, D Gusmao-Flores^1^

#### ^1^Pós-graduação em Medicina e Saúde, Salvador, Brazil,^2^Hospital da Mulher, Salvador, Brazil

**Introduction:** The purpose of this study was to summarize the evidence assessing the qSOFA [1], calculated in admission of the patient in emergency department (ED) or intensive care unit (ICU), as a predictor of mortality. The hypothesis was that this tool had a good prediction performance.

**Methods:** Systematic review and meta-analysis of studies assessing qSOFA as prediction tool for mortality found on PubMed, OVID, EMBASE, SCOPUS and EBSCO database from inception until November 2017. The primary outcomes were mortality (ICU mortality, in-hospital mortality, 30 and 90-day mortality). Studies reporting sensitivity and specificity of the qSOFA making it possible to create a 2x2 table were included. The diagnostic odds ratio (LnDOR) was summarized following the approach of DerSimonian and Laird using the software R (‘mada’ package). The summary ROC curve was created using the Reistma model (bivariate model). The RevMan 5 software was used to organize the data.

**Results:** The search strategy yielded 266 citations. Of 134 unique citations, 48 met the inclusion criteria (426,618 patients). The sensitivity and specificity from each study are shown in Fig. 1. The meta–analysis of the DOR was 4.838 (95% confidence interval (CI): 3.808 - 6.146) and of the LnDOR was 1.576 (95% IC: 1.337 - 1.816) (Fig. 2). The pooled area under the summary receiver operating characteristic (SROC) curve was 0.717. The summary estimative of the sensitivity was 0.55 and the false positive rate was 0.209, by bivariate diagnostic random-effects meta-analysis. The Chi-square goodness of fit test rejects the assumption of homogeneity, and the fit of the model for heterogeneity was better (p-value = 0.3661).

**Conclusions:** The qSOFA has a poor performance to predict mortality in patients admitted to the ED or ICU.

**Reference**

[1] Singer M et al. JAMA 315(8):801-10, 2016.

**Fig. 1 (abstract P086).**
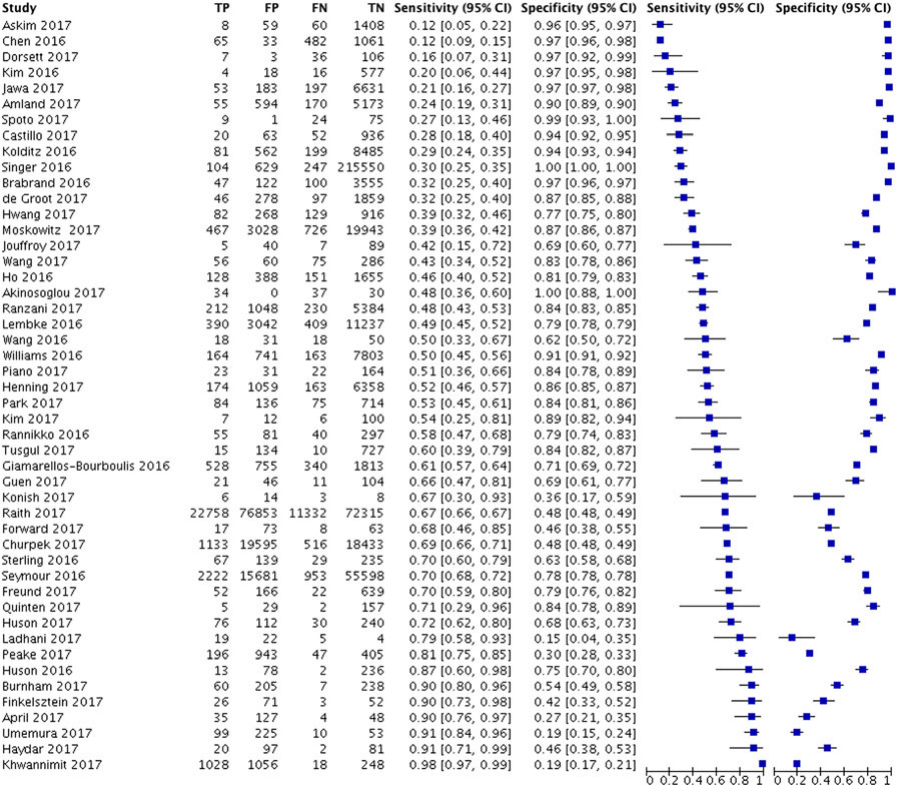
Sensitivity and specificity of all included studies

**Fig. 2 (abstract P086).**
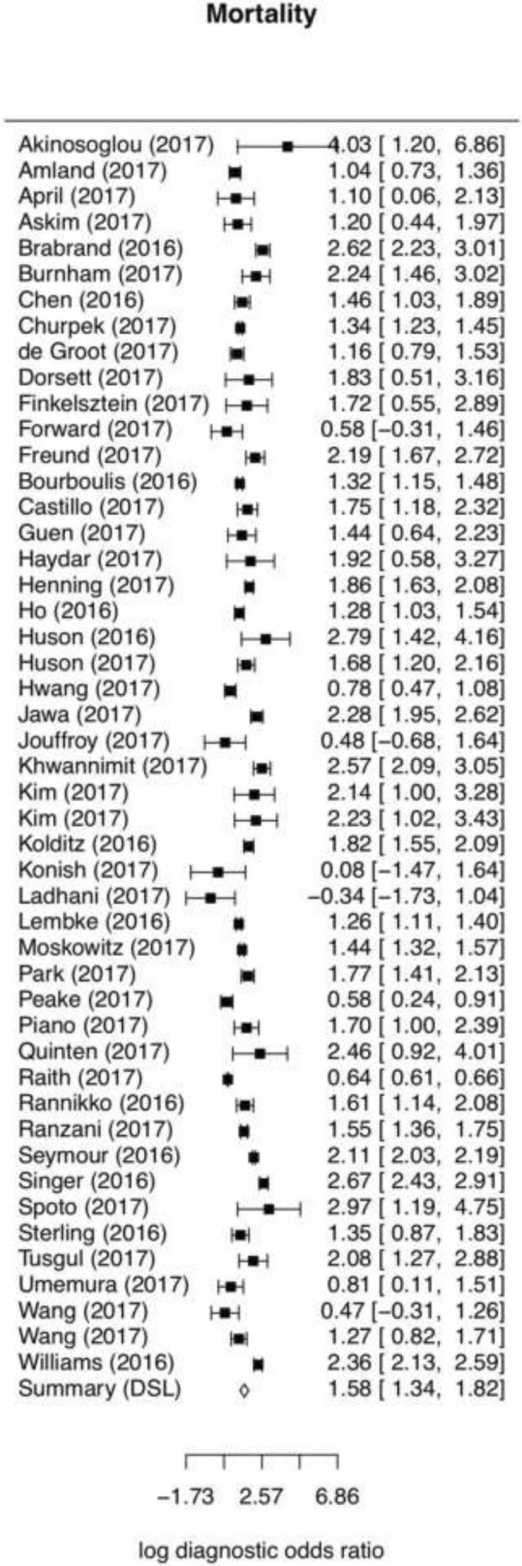
Forest plot lnDOR

## P087 Can early changes in SOFA score predict final outcome? An analysis of two cohorts

### E Karakike^1^, I Tsangaris^1^, A Savva^1^, C Routsi^1^, K Leventogiannis^1^, M Tsilika^1^, V Apollonatou^1^, I Tomos^1^, JL Vincent^2^, EJ Giamarellos-Bourboulis^1^

#### ^1^National and Kapodistrian University of Athens, Athens, Greece,^2^Erasme Hospital, Université Libre de Bruxelles, Brussels, Belgium

**Introduction:** Based on the new Sepsis-3 definitions, it may be hypothesized that changes of baseline SOFA (Sequential Organ Failure Assessment score, δ _SOFA_) may become a measure of treatment efficacy. To this end, the earliest time-point over the sepsis course where these changes are seen should be specified. This was attempted in the present study through test and validation cohorts.

**Methods:** We used clinical data coming from two randomized clinical trials where intravenous clarithromycin was compared to placebo in patients with Gram-negative infections, meeting the 1991 sepsis definitions [1, 2]. We depicted those patients who retrospectively met Sepsis-3 definitions; 449 patients were in the test cohort [1] and 199 in the validation cohort [2]. δ _SOFA_ on days 2, 3, 5, 7, 14 and 28 were calculated. The areas under the curves (AUC) of ROCs were designed to define association with 28-day mortality.

**Results:** The AUCs on follow-up days (Table 1) indicated earliest changes on day 7 to detect outcome. On that day, less than 25% decrease of SOFA was associated with 87.1% sensitivity for 28-day mortality (odds ratio 14.44; p=3.13 x10^-22^). This was 94.9% in the validation cohort (odds ratio 6.95; p=2.1 x10^-4^).

**Conclusions:** 25% decrease of SOFA by day 7 is associated with 28-day mortality and could guide decision-making in future sepsis trials.

The study was supported by the Horizon 2020 Marie-Curie Grant European Sepsis Academy

**References**

1. Giamarellos-Bourboulis EJ et al. J Antimicrob Chemother 69: 1111-8, 2014

2. Giamarellos-Bourboulis EJ et al. Clin Infect Dis 46: 1157-64, 2008

**Table 1 (abstract P087).** Change of SOFA to discriminate final outcome over-follow-up

	Area Under the Curve (95% CI)	P vs day 2
Day 2	0.77 (0.72-0.81)	
Day 3	0.77 (0.72-0.82)	0.941
Day 5	0.80 (0.76-0.85)	0.200
Day 7	0.84 (0.79-0.88)	0.030
Day 14	0.87 (0.83-0.91)	0.004
Day 28	0.90 (0.86-0.93)	<0.0001

## P088 Delta SOFA scores for prediction of treatment outcome in sepsis and septic shock patients

### T Lertwattanachai^1^, P Dilokpattanamongkol^1^, V Tangsujaritvijit^2^

#### ^1^Faculty of Pharmacy, Mahidol University, Bangkok, Thailand,^2^Faculty of Medicine Ramathibodi Hospital, Mahidol University, Bangkok, Thailand

**Introduction:** Sepsis and septic shock patients are the most common cause of death in intensive care units.[1] The aim of this study is to quantify the relationship between 72 hours sequential organ failure assessment (SOFA) scores change and in-hospital mortality as a treatment outcome in sepsis and septic shock patients.

**Methods:** A retrospective cohort study in tertiary hospital, Thailand was conducted. Sepsis or septic shock patients receiving carbapenems in medical intensive care unit during April to September 2017 were recruited. Delta SOFA scores, calculated by SOFA day 4 – SOFA day 1 after receiving the first dose of carbapenem, and in-hospital mortality were collected.

**Results:** There were 86 adult patients (54.70% men, mean age 66 + 17 years, and 70.90% septic shock) during the study period. In-hospital mortality rate was 43%. Mean delta SOFA scores were -0.060 + 3.572 points. Comparing between two groups, the mean delta SOFA scores were significant lower in survivor group than in non-survivor group (-1.140 + 3.116 vs. 1.380 + 3.669; P < 0.001).

**Conclusions:** The delta SOFA scores during the first few days were a useful predictor of in-hospital mortality in critically ill patients with sepsis and septic shock. An increase in delta SOFA score in the first 72 hours of treatment should be reassess for evaluate an adequacy of antibiotic therapy.

**Reference**

1. Bassetti M et al. Intensive Care Med. 44:73-75, 2017

## P089 Experience of having no set criteria for an outreach (rapid response) team in a new hospital

### H Tan, J Teh, F Lee, G Soo, N Horsahamay, S Tan, Y Lee, F Khan

#### Ng Teng Fong General Hospital, Singapore, Singapore

**Introduction:** An Outreach Team, akin to a Rapid Response Team, is made up of healthcare professionals assembled together for quick and effective reviews in managing of rapidly deteriorating or gravely deteriorated patients [1]. This study aimed to look at the variety of patient referrals in terms of their severity, patient dynamics, reasons for referral and their subsequent dispositions.

**Methods:** 258 patient records were randomly reviewed retrospectively from July to October 2017. Data were collated in an excel spreadsheet for comparison and then sorted in accordance with the clinical questions and percentages calculated.

**Results:** From the 258 referrals, the severity criteria was done by calculating the National Early Warning Score (NEWS). It was found that 51% patients had a score of 0-4, 23% had a score of 5-6, and 26% scored more or equal to 7. 50% of patients were in the age range 61-70 years old. 78% referrals came from the Emergency Department (ED) where a consultant was involved in the decision of the referral; of this, 46% were referred during office hours of 8AM to 5PM where there was greater manpower to aid management. 19% referrals came from inpatients on the General Wards; 32% were done during office hours. 65% of referrals were transferred to IC/HD upon review; 35% were not, from whom 9 died and 7 were later admitted after procedures (2%) or because they deteriorated further (1%). For reasons for referrals and disposition decisions, see Fig. 1.

**Conclusions:** Despite having no set criteria for Outreach Team referrals, the accuracy rate was nearly 65% admissions to IC/HD based on clinician concerns. There was only 1% re-admission rate having been re-reviewed when the patients had not been deemed suitable for IC/HD admission initially. Therefore referrals were done accurately and safely with the protocol of clinician referral openness directly to IC consultants.

**Reference**

DeVita MA et al. BMJ Quality & Safety 13: 251-254, 2004.

**Fig. 1 (abstract P089).**
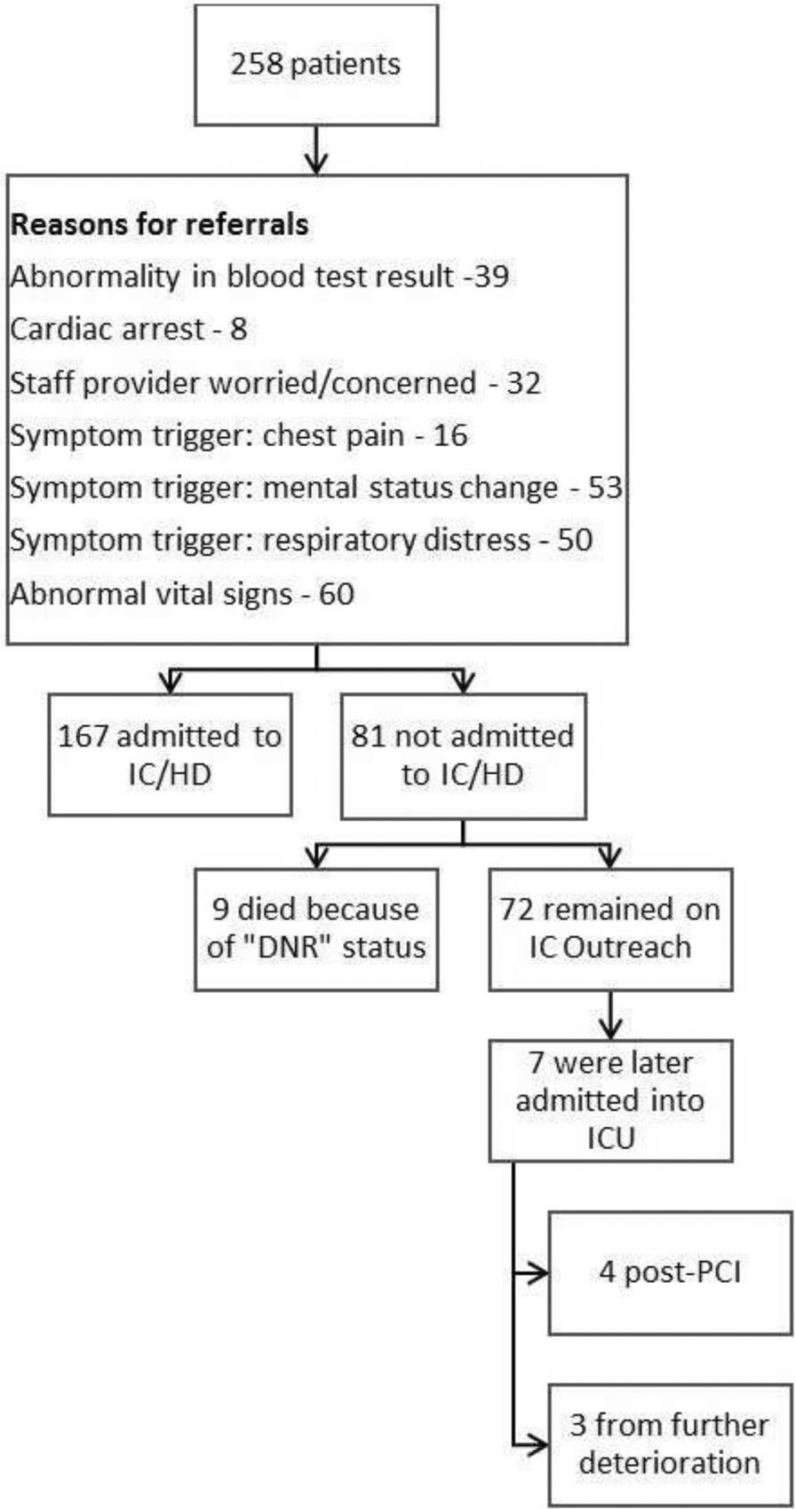
Consort diagram of reasons for referral and various dispositions

## P090 A novel model for early detection of patient deterioration in ICU

### Y Lichter^1^, D Stavi^1^, U Keler^2^, IM Pessach^3^, H Artsi^1^, N Adi^1^, I Matot^1^

#### ^1^Tel-Aviv Sourasky Medical Center, Tel Aviv, Israel,^2^Intensix, Netanya, Israel,^3^Sheba Medical Center, Safra Children’s Hospital, Tel-Hashomer, Israel

**Introduction:** Prompt recognition of patient deterioration allows early initiation of medical intervention with reduction in morbidity and mortality. This digital era provides an opportunity to harness the power of machine learning algorithms to process and analyze big data, automatically acquired from the electronic medical records. The results can be implemented in real-time. Intensix (Netanya, Israel) has developed a novel predictive model that detects early signs of patient deterioration and alerts physicians. In this study we prospectively validated the ability of the model to detect patient deterioration in real time.

**Methods:** The model was developed and validated using a retrospective cohort of 9246 consecutive patients admitted to the Intensive Care Unit in the Tel-Aviv Sourasky medical center – a tertiary care facility in Israel, between January 2007 and December 2015. In this study, we tested model performance in real time, on a cohort of 333 patients admitted to the same ICU between June 2016 and August 2017. Significant events that lead to major interventions (e.g. intubation, initiation of treatment for sepsis or shock, etc.) were tagged upon medical case review by a senior intensivist, blinded to model alerts. These tags were then compared with model alerts.

**Results:** A total of 136 patients suffered major events during study period, out of which 109 were detected by the model, resulting in a sensitivity of 0.80, specificity of 0.93 and a PPV of 0.89. The model AUC-ROC was 0.86.

System operation and algorithm execution were fluent and reliable.

**Conclusions:** We developed a machine-learning model that can reliably recognize patient deterioration in real time. Ongoing research aims at showing improved model validity and verifying its ability to precede clinical detection. Future research is needed to demonstrate positive effect on patient outcome.

## P091 Detecting acute deterioration in hospital – the NEWS is not enough

### T Buttle, P Parulekar, G Glover

#### Guys and St Thomas’ NHS Foundation Trust, London, UK

**Introduction:** The UK National Early Warning Score (NEWS) is advocated to detect acute deterioration in hospital [1], however NEWS may lack sensitivity to detect all patients requiring Critical Care (CC) admission.

**Methods:** An analysis of the Rapid Response Team (RRT) database for a university hospital; all acute reviews/emergency calls, 1st March – 31st May 2017. Protocolised RRT calling criteria are NEWS >=5/single parameter score 3. For patients reviewed by RRT, probability of CC admission was calculated at each NEWS level. For admissions not meeting calling criteria (‘low NEWS’), reason for admission was investigated. Data is shown as median [IQR] or count (%).

**Results:** There were 2742 acute RRT reviews for 1009 patients; median [IQR] NEWS 4 [3-6]. 1240 reviews occurred despite ‘low NEWS’ (Fig. 1). RRT review led to CC admission in 315 (31.2%) cases; median [IQR] NEWS 5 [3-8]. Probability of admission increased with higher NEWS (Fig. 1), however 82 admissions had ‘low NEWS’. Of these 51 were excluded due to high NEWS trigger in the preceding 24hrs or post-operative status. The remaining 31 (9.8%) represented genuine low NEWS cases; age 54 [36-64], 50% male, admission APACHE II 12 [8-17] and day 1 SOFA 2 [1-5]. Admission source was emergency department 29%, medical 42%, surgical 29%. Diagnoses are shown in Table 1. No low NEWS patients with sepsis were qSOFA positive. CC length of stay was 2 [1-4] days and ICU mortality was 9.6%.

**Conclusions:** A high proportion of RRT activity occurs at low levels of abnormal physiology. Despite an association between NEWS and CC admission, NEWS fails to trigger for approximately one in ten admitted cases. Clinical concern remains an important component of the escalation of acutely ill patients. Meanwhile, novel markers of deterioration should be sought and validated.

**Reference**

1. Smith GB et al. Resuscitation 84:465-70, 2013

**Table 1 (abstract P091).** Diagnostic categories

Diagnostic category	No (%)
Airway	8 (25.8)
Respiratory Failure	7 (22.6)
Cardiovascular (Arrhythmias/haemorrhage)	6 (19.4)
Acute Kidney Injury (inc. hyperkalaemia)	8 (25.8)
Neurological	6 (19.4)
Endocrine/Metabolic/Electrolytes (other)	7 (22.6)
Sepsis	5 (16.1)

**Fig. 1 (abstract P091).**
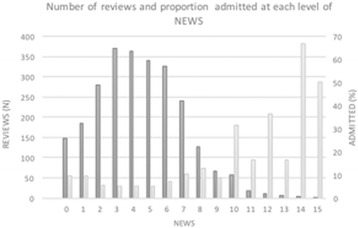
Number of reviews and proportion admitted at each level of NEWS

## P092 The impact of diurnal pattern of rapid response call activation on patients outcome

### J Silvestre, N Candeias, A Ricardo, R Marques, F Brás, J Nunes

#### Hospital dos Lusiadas, Lisbon, Portugal

**Introduction:** Although rapid response systems are known to reduce in-hospital cardiac arrest rate, their effect on mortality remains debated. The Rapid Response Call (RRC) is a system designed to escalate care to a specialised team in response to the detection of patient deterioration. There are diurnal variations in hospital staffing levels that can influence the performance of rapid response systems and patient outcomes. The objective of this study was to examine the relationship between the time of RRC activations and patient outcome.

**Methods:** Review of retrospectively collected, linked clinical and administrative datasets, at a private hospital during a 34-month period. All patients with medical emergency team activation were included. Rapid response calls occurring between 18:00-07:59 were defined as ‘out of hours’.

**Results:** Between January 2015 and October 2017 there were 209 RRC. The trigger for RRCs activation was nurse concern (101; 38.3%), modified early warning score (80; 28.3%) and cardiac arrest (28; 13.4%). 44 RRCs were “out of hours” being the main activation trigger a modified warning score > 5. “Out of hours” patients had higher ICU admissions (31.7% versus 20%) and were more likely to have an in-hospital cardiopulmonary arrest (OR=1.4, p<0.002).

**Conclusions:** The diurnal timing of RRCs appears to have significant implications for patient outcomes. Out of hours calls are associated to a poorer outcome. This finding has implications for staffing and resource allocation.

## P093 Sepsis in medical and surgical patients: a 6 years retrospective study

### G Zani, F Di Antonio, M Pichetti, A Garelli, C Gecele, F Menchise, M Valbonetti, S Mescolini, E Graziani, C Nencini, FD Baccarini, M Fusari

#### Santa Maria delle Croci Hospital, Ravenna, Italy

**Introduction:** Sepsis is life-threatening organ dysfunction caused by a dysregulated host response to infection [1, 2]. We compared organ failure incidence and evolution in medical versus surgical septic patients in ICU.

**Methods:** Septic patients admitted to a general ICU were retrospectively analyzed from 2012 to 2017 for: SAPSII, SOFA score at ICU admission and worst value during ICU stay, site of infection and severity, duration of MV, need and timing for tracheotomy, need and duration of vasoactive drugs, need for RRT, ICU-acquired infections, ICU and post-ICU LOS and outcome. Traumatic and neurological patients were excluded. P value <0.05 was considered significant.

**Results:** 956 septic patients were enrolled: 56% medical and 44% surgical. Medical patients were younger (66vs70yy, p<0.05) and with worst SAPSII (53vs49, p<0.05). At ICU admission SOFA score was higher in medical patients (9vs7, p<0.05), due primary to neurological and renal dysfunction. During ICU stay, medical patients revealed an haemodynamic worsening (8% shock increase, p<0.05). Moderate ARF was prevalent in both groups; surgical patients had a higher need of MV (96%vs83%, p<0.05), but with a shorter duration than medical ones that were mostly treated with tracheotomy (26vs15%, p<0.05). AKI was more severe in medical patients and worsened in both groups without differences on need of RRT. Targeted antibiotic therapy was higher in medical patients (63vs35%, p<0.05), but no differences emerged for duration and superinfections. Medical patients had a longer ICU LOS (8vs6dd, p<0.05), with a higher ICU mortality rate (26vs17%, p<0.05); they showed a shorter post-ICU LOS (11vs16dd, p<0.05) with a higher but not significant inhospital mortality rate (37vs33%).

**Conclusions:** Septic medical patients had a worst outcome in comparison to surgical ones, probably related to a more severe clinical state at ICU admission and a worsening in organ function.

**References**

[1] Rhodes A et al. Intensive Care Med 43:304-377,2017

[2] Michael D et al. JAMA 317:847-848,2017

## P094 The use of the medication based disease burden index and drug counts to quantify comorbidity during intensive care and to predict long term mortality

### C McMechan^1^, M Booth^2^, J Kinsella^1^

#### ^1^University of Glasgow, Glasgow, UK,^2^Glasgow Royal Infirmary, Glasgow, UK

**Introduction:** The aims of the project were to use the Medication based Disease Burden Index (MDBI) and drug counts to quantify comorbidity at ICU admission and assess how levels of comorbidity change during a stay in ICU and to assess how comorbidity affects long term survival after a stay in ICU. Pharmacy data offers an alternative method of quantifying comorbidity and the MDBI is one method of doing this that can predict mortality.

**Methods:** Data was collected from patients admitted to Glasgow Royal Infirmary ICU between 01/01/14 to 31/12/15. Ethical approval was sought. This data was used to produce an MDBI score and a drug count before and after an ICU stay. Information on long term mortality was also collected. T tests were used to determine the difference in comorbidity levels pre and post ICU. Kaplan Meier curves were used to establish if comorbidity affects long term mortality. Logistic regression was used to determine which method of measuring comorbidity was better at predicting mortality.

**Results:** A paired t test was performed on 437 patients that showed comorbidity increases after a stay in ICU, as measured by the MDBI and drug counts. Kaplan Meier curves demonstrated that as comorbidity increases, long term survival decreases. Survival time was calculated as time from ICU discharge date to the date of death or end of study (31/01/17). The hazard ratio for the high MDBI group was 1.89 when compared to the zero MDBI group. The hazard ratio for the high drug count group was 1.81 when compared to the zero drug count group. Cox proportional hazard models were performed and results remained significant after adjusting for age. Logistic regression showed that the MDBI was better at predicting long term mortality than drug counts.

**Conclusions:** This study increases the ever-growing evidence that the MDBI is a useful predictive tool for quantifying comorbidity and predicting long term mortality. Further research is required to replicate its use in other populations, and potentially other specialities.

## P095 Adverse events among those admitted to the ICU: a retrospective cohort study using administrative data

### KM Sauro, A Soo, HT Stelfox

#### University of Calgary, Calgary, Canada

**Introduction:** The objective of this study is to estimate the frequency and type of, and factors associated with adverse events (AEs) among those with an intensive care unit (ICU) admission. AEs are unintended, negative consequences of care that compromise patients’ health and are costly to the healthcare system. In Canada, an estimated 7.5 per 100 hospital admissions are associated with an AE, but evidence suggests that the rate of AEs varies by hospital unit and by patient population. However, few studies examine AEs in the ICU.

**Methods:** This retrospective cohort study included patients admitted to 30 adult ICUs & CCUs in Alberta, Canada (n=30) between May 2014 and April 2017. Validated ICD-10CA algorithms for 18 patient safety indicators were used to estimate the frequency of any AE and each type of AE. Regression analysis was used to examine factors associated with AEs.

**Results:** Of 49,447 admissions, the typical admission was a 62 (IQR=21) year old male (64%), admitted for a non-surgical cardiac reason (35%). At least 1 AE was experienced by 12,549 (25%) ICU patients during their hospital admission. The most common AEs were respiratory complications (10%) and hospital acquired infections (9%). Those who were re-admitted to ICU (OR=4.83, 95% CI=4.48, 5.20), admitted for a general surgical vs. non-surgical cardiac reason (OR=9.49, 95% CI=8.84, 10.20) and had >=2 comorbidities (OR=1.82, 95% CI=1.73, 1.92) had increased odds of an AE, while those who spent >50% of their hospital admission in ICU (OR=0.42, 95% CI=0.41, 0.44) had decreased odds of an AE. Those who experienced an AE stayed 5.8 days longer in ICU and 23.5 days longer in hospital, and had increased risk of hospital mortality (OR=2.41, 95% CI=2.27, 2.55) than those who did not experience an AE.

**Conclusions:** AEs are common among patients admitted to ICU, highlighting the need for ongoing quality improvement initiatives to improve the safety of care.

## P096 Clinical characteristics and outcomes of toxic epidermal necrolysis in a Tunisian tertiary critical care unit (icu)

### I Ben Saida, W Zarrougui, E Ennouri, N Sma, N Fraj, S Kortli, N Fathallah, M Boussarsar

#### Farhat Hached Teaching hospital, Sousse, Tunisia

**Introduction:** Toxic epidermal necrolysis (TEN) is a rare, potentially life threatening mucocutaneous disease. The aim of the study was to determine clinical characteristics and outcomes of patients admitted to ICU with a diagnosis of TEN.

**Methods:** A retrospective study was performed in the ICU of Farhat Hached hospital of Sousse between January 1995 and September 2017. Data were collected by reviewing the medical patients’ charts. A multivariate regression analysis was used to identify risk factors for ICU mortality in those patients.

**Results:** A total of 27 patients were recorded. Mean age was 43 years (range, 17 to 76). 19(70.4%) were male. Median of CHARLSON index was 1 [0-4]. Mean SAPS II was 29.59±16. The average affected skin area was 50.5 ± 28.95% of total body surface area. Mucous membrane involvement was seen in the mouth or pharynx (21, 77.8%), eye (18, 66.7%) and genital area (15, 55.6%). NIKOLSKY sign was positive in 25 patients. The most common drugs that triggered TEN were antibiotics (8/27, 29.62%), allopurinol (6/27, 22.22%), anticonvulsants (5/27, 18.51 %), non-steroidal anti-inflammatory drugs (3/27, 11.11%), and antipsychotic drugs (1/27, 3.7%). 6 patients (22.2%) required mechanical ventilation, 7 (25.9%) vasoactive drugs and 2 (7.4%) renal replacement therapy. The major complications were acute renal failure (51.9%) and sepsis (29.6 %). The mortality rate was 40.6%. This rate was much higher than predicted mortality according to a severity-of-illness scoring system for TEN prognosis (SCORTEN) score. In univariate analysis, predictors of fatal outcome were: invasive mechanical ventilation (p=0.0.27), vasoactive drugs (p=0.026), acute renal failure (p=0,012), age (p=0.042) and CHARLSON index (p=0.01). Acute renal failure was the only independent factor of ICU mortality (OR, 19.8 ; 95%CI, [1.94- 201.62] ; p=0.012).

**Conclusions:** The present study demonstrated a severe prognosis in TEN patients. Acute renal failure was identified as the sole independent factor associated to mortality.

## P097 D-dimer and the national early warning score: identifying medical patients at low risk of 30-day mortality in a Danish emergency department

### H Lyngholm^1^, C Nickel^2^, J Kellett^1^, S Chang^1^, M Brabrand^1^

#### ^1^Hospital of South West Jutland, Esbjerg, Denmark,^2^University Hospital Basel, Emergency Department, Basel, Switzerland

**Introduction:** The aim of this study was to prospectively validate the use of low D-dimer levels in combination with a low National Early Warning Score (NEWS) to identify medical patients at low risk of 30-day mortality in an unselected cohort representative of acutely ill patients normally seen in an emergency department (ED).

**Methods:** In this prospective observational study, plasma D-dimer levels and NEWS of all acute adult consenting medical patients presenting to the ED at the Hospital of South West Jutland were assessed at arrival. 30-day survival status was extracted from the Danish Civil Registration System which ensured complete follow-up. Patients were sorted by high and low d-dimer with a cut-off value of 0.50 mg/L and additionally by high and low NEWS with a cut-off score of 2.

**Results:** The final study population consisted of 1516 patients with a median (25-75 percentile) age 66 years (52-77) of which 49.4% were female. 791 (52.2%) patients had a low D-dimer (<0.50 mg/L) of which 3 (0.38%, 95%CI 0.12-1.17%) died within 30 days; all of these patients had a low NEWS (<2). 725 (47.8%) patients had a high d-dimer (>=0.50 mg/L) of which 32 (4.4%, 95%CI 3.14-6.18) died; 12 (37.5%) of these had a low NEWS (<2). Comparing 30-day mortality for patients with high and low D-dimer, the odds ratio is 12.3 (95%CI 3.7-40.3). 14 of the 35 patients (40.0%) who died had a low NEWS at presentation to the ED.

**Conclusions:** Low D-dimer levels appear to identify patients at low risk of 30-day mortality. The addition of NEWS does not appear to increase this ability. Further validation is needed.

## P098 Comparison of severity score models based on different sepsis definitions for predicting in-hospital mortality of sepsis patients in medical intensive care unit

### T Songsangjinda, B Khwannimit

#### Prince of Songkla University, Hat Yai, Thailand

**Introduction:** There are three generations of sepsis definition concepts: Systemic Inflammatory Response Syndrome (SIRS), Predisposition, Insult, Response, Organ dysfunction (PIRO), and Sequential Organ Failure Assessment (SOFA). However, the performance between these concepts had not been compared. The aim of our study to evaluate and compare the performance between severity score models based on different sepsis definitions in order to predict outcomes among sepsis patients.

**Methods:** A retrospective analysis over a 10-year period. The primary outcome was in-hospital mortality and the secondary outcome was the composite of hospital death and ICU stay of more than 72 hours.

**Results:** A total of 2,152 sepsis patients were enrolled. The hospital mortality was 45.9%. Mean APACHE-II score was 23.9. The SOFA score had the highest performance for predicting hospital mortality with an area under the receiver operating characteristic curve (AUC) of 0.86. The AUC of SOFA score was statistically greater than the other scores (p<0.001, Fig. 1). Also, the SOFA and qSOFA presented good discrimination for secondary outcome. The AUC of SOFA (0.76) and qSOFA (0.76) for predicting secondary outcome was statistically greater than those of SIRS (0.59, p<0.001) and the PIRO models (Howell 0.72, Robulotta 0.71, p=0.01). In the subgroup analysis (n=1,239), serum lactate value (>2 mmol/L) was shown to improve qSOFA specificity from 32.4% to 54.1% with comparable sensitivity (96.9% and 94.7%). Howell’s PIRO performance was not significantly changed.

**Conclusions:** The SOFA score had the best performance for predicting hospital mortality among ICU sepsis patients. Our findings support the Sepsis-3 using SOFA in an ICU setting.

**Fig. 1 (abstract P098).**
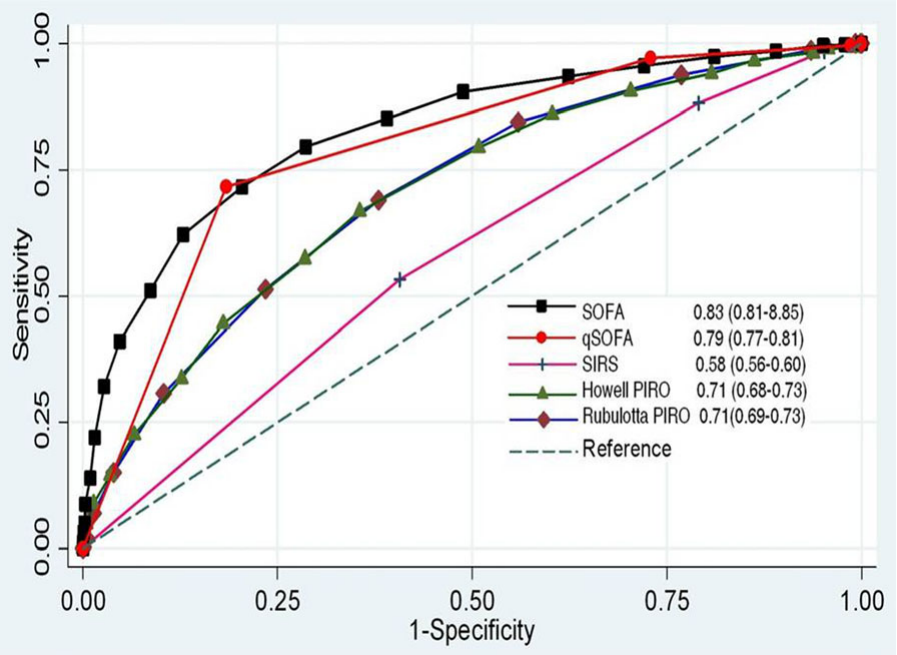
Comparison of the area under the receiver operating characteristic curve of all scores for predicting hospital mortality in ICU sepsis patients.

## P099 Trends in infection and sepsis incidence and mortality in Germany

### A Mikolajetz

#### Jena University Hospital, Jena, Germany

**Introduction:** Sepsis is one of the most prevalent diseases among hospitalized patients and an important contributor to hospital mortality. The study aims to assess trends in infection and sepsis incidence and mortality between 2010-2015 in Germany.

**Methods:** We analyzed hospital discharge data from the years 2010-2015 by using the Diagnosis-related Groups Statistics of the German Federal Statistical Office, which contains nearly complete data of all inpatient hospital treatments in Germany. We identified cases of infection, infection and organ dysfunction, sepsis (incl. severe sepsis and septic shock) and severe sepsis (incl. septic shock) using ICD-10 codes coded as primary and secondary discharge diagnoses and procedural OPS codes. We assessed incidences and discharge disposition incl. mortality.

**Results:** Incidences, mortalities and discharge disposition comparing 2010 and 2015 and the mean annual increase in incidence rates are reported in Tables 1 and 2.

**Conclusions:** The annual increase in standardized sepsis incidence rates is greater than in infections, but similar to the increase in infectious disease patients with organ dysfunction, which are less prone to coding incentives than sepsis codes. An increasing number of patients is discharged to nursing homes and hospice. Given the alarming increase in sepsis cases and deaths, this analysis confirms sepsis as a key priority for health care systems.

**Table 1 (abstract P099).** Infections, 2010-2015 in Germany: Incidence, mortality and discharge disposition

	Infection in 2010	Infection in 2015	Infection and organ dysfunction in 2010	Infection and organ dysfunction in 2015
Cases	3,691,241	4,173,237	770,258	1,143,674
Incidence/100.000 (age- and sex-standardized)	4,515	4,894 (mean annual increase +1.6%)	942	1,315 (mean annual increase +7.0%)
Deaths	221,098	243,902	147,849	189,529
Mortality	6.0%	5.6%	19.2%	16.3%
Discharge to hospice	0.11%	0.14%	0.19%	0.25%
Discharge to nursing home	3.56%	4.41%	5.03%	6.62%
Discharge to rehab	2.79%	2.33%	4.51%	3.65%

**Table 2 (abstract P099).** Sepsis, 2010-2015 in Germany: Incidence, mortality and discharge disposition

	Sepsis in 2010	Sepsis in 2015	Severe sepsis in 2010	Severe sepsis in 2015
Cases	229,214	320,198	87,973	136,542
Incidence/100.000 (age- and sex-standardized)	280	371 (mean annual increase +5.8%)	108	158 (mean annual increase +7.9%)
Deaths	61,068	75,227	42,084	56,875
Mortality	26.6%	23.1%	47.8%	41.2%
Discharge to hospice	0.17%	0.23%	0.12%	0.19%
Discharge to nursing home	3.77%	5.04%	2.85%	4.31%
Discharge to rehab	4.67%	3.65%	5.86%	4.49%

## P100 Initial management of sepsis by day of the week in the north west of England

### W Angus^1^, P Turton^2^, E Nsutebu^2^, C McGrath^1^

#### ^1^Wirral University Teaching Hospital NHS Foundation Trust, Wirral, UK,^2^Royal Liverpool and Broadgreen University Hospitals NHS Trust, Liverpool, UK

**Introduction:** The Advancing Quality Sepsis Programme is an established approach to reducing variation and improving outcomes in the North West of England. It aims to improve clinical care by producing and implementing evidence-based bundles of care across a collaborative network of hospitals. Data is collected, analysed and fed back enabling monitoring and comparison of quality of sepsis care in the form of an Appropriate Care Score (ACS), mortality rate and length of stay.

**Methods:** Between September 2014 and 2016 data from 25,358 patients who generated an inpatient sepsis code (ICD10) were collected. Of these 11,301 patients had confirmed sepsis (Sepsis 2 criteria) at presentation. 5207 patients were either hypotensive (SBP <90mmHg) or hyperlactataemic (Lactate >4mmol/L) at presentation. ACS, mean time to antibiotics, blood cultures and lactate measurement were calculated for each day of the week. Mortality and length of stay were measured, enabling comparison of weekday and weekend presentation. Data was analysed using SPSS software.

**Results:** Comparing weekend to weekday presentation did not reveal any significant differences in ACS, time to antibiotics, blood cultures or lactate measurement. Mortality rates and length of stay were not significantly different between the groups. There does not appear to be a weekend effect in sepsis care for this cohort of patients. There were more patients with hypotension and/or hyperlactaemia presenting on a Monday.

**Conclusions:** Quality of sepsis care was not significantly different between weekend and weekday presentation for patients in this cohort. There were no significant differences in mortality or length of stay when comparing weekday or weekend presentation. There were more septic patients with hyperlactataemia and/or hypotension presenting on a Monday, which may indicate a reluctance of septic patients to present over the weekend.

## P101 Weekend effect is not associated with delays in antibiotic administration in septic patients in the emergency department

### R Passos, J Ramos, B Fahel, C Starteri, G Silva, M Manciola, M Barbosa, J Caldas, S Farias, M Teixeira, A Gobatto, M Ribeiro, P Batista

#### Hospital Sao Rafael, Salvador, Brazil

**Introduction:** Patients with urgent admissions to the hospital on weekends may be subjected to a higher risk of worse outcomes, which may be due to differences in compliance to established processes. Because delays to antibiotic administration is an important measure of sepsis protocol efficiency and has been associated to worse outcomes, we aimed to assess the association of the weekend effect (admissions on weekend) with timing to antibiotic administration.

**Methods:** Patients included in the sepsis protocol in the emergency department (ED) of Hospital Sao Rafael, from January 2016 to July 2017 were retrospectively evaluated. Sepsis protocol is supposed to be activated to every patient with a suspected sepsis diagnosis in the ED. We evaluated the association of weekend (saturday or sunday) admission with timing to antibiotic administration.

**Results:** In the study period, 257 patients were evaluated, of which 121 (47%) were male, with a mean age of 59±23 years. Mortality was 27% (70 patients) and 113 (44%) were admitted to the ICU. Mean SOFA score was 2±1.8 and mean Charlson comorbidity index was 4±3.2. Sixty-eight (26%) patients were admitted during weekend. There was no difference in time to antibiotic administration between patients admitted during weekend (31±42 minutes) and patients admitted during weekdays (31±41 minutes). Also, mortality was similar for both groups of patients [OR(95%CI)=0.83(0.47-1.59)].

**Conclusions:** In this cohort of patients with a suspicion of sepsis in the ED, admission during weekend was not associated to worse outcomes.

## P102 Relationship between intensive care unit hypotension and morbidity in patients diagnosed with sepsis

### K Maheswari^1^, M Stevens^2^, S Munson^3^, B Nathanson^4^, S Hwang^3^, A Khanna^1^

#### ^1^Cleveland Clinic, Cleveland, OH, USA,^2^Edwards Lifesciences, Irvine, CA, USA,^3^Boston Strategic Partners, Inc., Boston, MA, USA,^4^OptiStatim, LLC, Longmeadow, MA, USA

**Introduction:** Current sepsis guidelines emphasize resuscitation of hypotension to a mean arterial pressure (MAP) of at least 65 mmHg [1]. A MAP less than 90 mmHg appears to be associated with poor outcomes in postoperative patients in the intensive care unit (ICU) [2]. However, extent of hypotension in critically ill septic patients during ICU stay and its relationship with adverse outcomes is poorly defined. We determined the magnitude of hypotension in ICU patients with a diagnosis of sepsis and its association with major complications.

**Methods:** With IRB approval we evaluated records from a large US electronic health records database (Cerner HealthFacts®, Kansas City, MO) of adult patients with a diagnosis of sepsis and ICU stay >= 24 hours from Jan 2010 - Nov 2016. Patients with a history of acute myocardial infarction or acute kidney injury (AKI) for six months prior to ICU admission or < 5 MAP readings/ICU day were excluded. Hypotension exposure was defined and analyzed via three methods: total time spent below MAP <65 mmHg; time-weighted average (TWA) MAP <65 mmHg, and the number of MAP readings <65 mmHg. Analyses were repeated for different MAP thresholds (<55, <75, <85 mmHg). We estimated association between hypotension exposure and a major morbidity composite defined by mortality, myocardial injury and AKI using multivariable logistic regression models.

**Results:** 10,495 patients met all qualifying criteria. 74% of sepsis patients experienced ICU hypotensive events with MAP <65 mmHg; 40% with MAP <55 mmHg. The number of minutes the average patient spent with MAP < 65 mmHg per ICU day will be presented, as will unadjusted/adjusted rates for the morbidity outcome, and unadjusted rates for composite components for all MAP thresholds.

**Conclusions:** The result of this analysis will determine the amount and duration of ICU hypotension that is associated with major morbidity in patients with sepsis.

**References**

1. Rhodes et al. Crit Care Med 45:486-552, 2017

2. Khanna AK et al. SCCM 2018 (Abstract #177)

## P103 The role of hyperoxia in sepsis mortality

### CV Cosgriff^1^, LA Celi^2^

#### ^1^Harvard T.H. Chan School of Public Health, Boston, MA, USA,^2^Massachusetts Institute of Technology, Cambridge, MA, USA

**Introduction:** The role of hyperoxia during oxygen administration in sepsis mortality remains uncertain and controversial [1, 2]. We hypothesized that the duration of hyperoxia while ventilated in the ICU was associated with increased in-hospital expiration in septic patients.

**Methods:** The Medical Information Mart for Intensive Care database (MIMIC-III), containing data for ~60,000 ICU admissions at Beth Israel Deaconess Medical Center from 2001 to 2012, was used to derive a cohort of ventilated sepsis patients [3]. Hyperoxia was defined as arterial oxygen saturation by pulse oximetry (SpO2) >98%. Extracted SpO2 were transformed to estimate time spent hyperoxic. Patients were grouped by hyperoxic duration into bins derived from quartiles of the hyperoxic duration. Group 1 (lowest quartile) was taken as the reference. The association between hyperoxic group and in-hospital mortality was examined by logistic regression.

**Results:** Of the 46,476 patients in MIMIC-III, 2,591 met criteria for inclusion. After adjustment for for age, gender, ethnicity, unit type, length of stay, duration of ventilation, disease severity, burden of comorbidity, and the use of vasopressors, hyperoxic group 4 was significantly associated with in-hospital mortality (OR = 2.47, p = 0.004, 95%CI: [1.35, 4.59]), as was group 3 (OR = 1.93, p = 0. 013, 95%CI: [1.16, 3.28]). Group 2 was not associated (OR = .84, p = 0. 564, 95%CI: [0.46, 1.53]). Figure 1 summarises these results.

**Conclusions:** Odds of in-hospital death were nearly double and more than double in group 3 and 4 respectively, and we conclude that longer durations of hyperoxia are associated with increased in-hospital mortality in sepsis patients.

**References**

[1] Vincent JL et al. Can Respir J 2017: 2834956, 2017

[2] Hafner S et al. 5:42. 2015

[3] Johnson AEW et al. Scientific Data 3:160035, 2016

**Fig. 1 (abstract P103).**
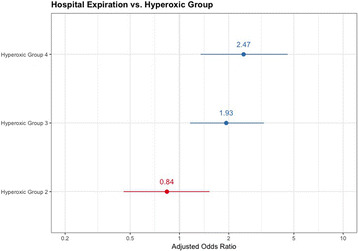
Adjusted OR for in-hospital expiration for the hyperoxic groups as compared to group 1 (lowest quartile of hyperoxic duration).

## P104 Treatment and outcomes of vasodilatory shock in an academic medical center

### ND Nielsen ^1^, F Zeng^2^, ME Gerbasi^3^, G Oster^3^, A Grossman^3^, NI Shapiro^4^

#### ^1^Tulane School of Medicine, New Orleans, LA, USA,^2^LaJolla Pharmaceutical Company, San Diego, CA, USA,^3^Policy Analysis Inc, Brookline, MA, USA,^4^Beth Israel Deaconess Medical Center, Boston, MA, USA

**Introduction:** Consensus clinical guidelines recommend maintaining mean arterial pressure (MAP) >=65 mmHg for vasodilatory shock (VS) patients. This study uses the Medical Information Mart for Intensive Care (MIMIC-III) database to examine treatment and outcomes in patients with severe VS in a real-world setting.

**Methods:** We identified patients in the MIMIC-III database which contains information for 61,532 admissions to intensive care units (ICU) at Beth Israel Deaconess Medical Center (BIDMC) in Boston, Massachusetts between 2001 and 2012. Inclusion criteria: 1) aged >=18 years, 2) treated with vasopressors for >=6 hours. Exclusion criteria: cardiac surgery, vasoplegia, cardiogenic shock, intra-aortic balloon pump, extracorporeal membrane oxygenation, large amount of blood transfusion, cardiac tamponade or pulmonary embolism. The primary outcome was mortality.

**Results:** There were 5,922 ICU admissions. Among these patients, those who consistently maintained MAP above 65 mmHg (n=167, 3%), 60 mmHg (n=418, 7%), and 55 mmHg (n=949, 16%) while in ICU had lower mortality rates than patients with one or more MAP excursions below these thresholds (n=5755, n=5504, and n=4973, respectively): 11% vs 31% for MAP <65 (p<0.0001); 10% vs 32% for MAP <60 (p<0.0001); and 10% vs 34% for MAP <55 (p<0.0001). When assessing the exposure of hypotension for >=2 continuous hours, ICU mortality rates were 31% for 65 threshold (n=4741), 34% for 60 threshold (n=3498), and 41% for 55 threshold (n=2090). ICU mortality rates were 41%, 52% and 66% for patients with MAP below 65 (n=1686), 60 (n=746), and 55 (n=354), respectively, for >=8 continuous hours.

**Conclusions:** Most patients did not have MAP control based on current clinical guidelines, and not achieving the recommended target MAP was associated with worse outcomes. However, since association does not imply causation, trials aimed at more aggressively achieving a MAP >=65 are warranted, and quality of care initiatives aimed at improving MAP control for patients with VS may be helpful.

## P105 Igm enriched immunoglobulins as adjunctive treatment to antimicrobial therapy for patient on septic shock

### A Corona, A Veronese, C Soru, F Cantarero, S Santini, I Cigada, G Spagnolin, E Catena

#### ASST Fatebenefratelli Sacco, PO SACCO - Milano, Milano, Italy

**Introduction:** Sepsis is responsible of both an immune hyperactivity damage from inflammation and an immune suppression and paralysis. A few studies support the role of IgM enriched immunoglobulins G as adjunctive of antimicrobial treatment [1].

**Methods:** Case-control prospective study. Since 12/2016 to 11/2017, patients experiencing a septic shock - admitted with a first 24h SAPS II > 25, associated with a SOFA-score > 4 – underwent treatment with IgM-e-IG, given for three days at the total dosage of 500 mg/kg. The therapy response was based on clinical, microbiological and rheological data. All cases were 1:1 matched with analogous controls.

**Results:** Over the study period 17 patients [cases, median age 50 (49-62),] experiencing severe infection (peritonitis, meningitis, community acquired pneumonia and UTI) and in septic shock, were recruited and treated with IgM enriched IgG (Pentaglobin) and matched with specific controls. No differences were found in basic patients characteristics but in median (IQR) SAPS II, little bit higher in cases [56 (43-67) vs. 46 (35-61), p=0.464]. No differences were found in the trend of the 1st 72 hrs in WBC, PCT, CRP, Lactate and PLT, even though SOFA score decreased significantly more in cases [-4 (-5;-2) vs. -1.5 (-2;-0.5), p=0.015]. 28th day survival was 100% in cases and 75.5% in controls (Log Rank = 1.93, p=0.165. VLAD (variable life adjusted display) showed 4.48 more lives saved than those expected by SAPS II for cases than controls (0.77), despite a similar SMR (p=0.419).

**Conclusions:** Reduced mortality may be supposed to be correlated to a quicker recovery of organ damage sepsis related. PCRTs should be warranted in the future to corroborate these preliminary data.

**Reference**

1. Cavazzuti I et al. Intensive Care Med 40(12):1888-96, 2014

## P106 A phase 1b study of anti-PD-L1 (BMS-936559) in sepsis

### RS Hotchkiss^1^, E Colston^2^, S Yende^3^, DC Angus^4^, LL Moldawer^5^, ED Crouser^6^, GS Martin^7^, CM Coopersmith^8^, S Brakenridge^5^, FB Mayr^3^, PK Park^9^, K Zhu^2^, M Wind-Rotolo^2^, T Duan^2^, J Ye^2^, Y Luo^2^, IM Catlett^2^, K Rana^2^, DM Grasela^2^

#### ^1^Washington University School of Medicine, St Louis, MO, USA,^2^Bristol-Myers Squibb, Inc., Lawrenceville, NJ, & Wallingford, CT, USA,^3^Veterans Affairs Pittsburgh Healthcare System and University of Pittsburgh, Pittsburgh, PA, USA,^4^University of Pittsburgh Critical Care Medicine CRISMA Laboratory, Pittsburgh, PA, USA,^5^University of Florida, Gainesville, FL, USA,^6^The Ohio State University, Columbus, OH, USA,^7^Dept. of Medicine, Division of Pulmonary, Allergy, Critical Care & Sleep Medicine, Emory University, Atlanta, GA, USA,^8^Dept. of Surgery, Emory University, Atlanta, GA, USA,^9^University of Michigan, Ann Arbor, MI, USA

**Introduction:** The PD-1/PD-L1 immune checkpoint pathway is involved in sepsis-associated immunopathy. We assessed the safety of anti-PD-L1 (BMS-936559, Bristol-Myers Squibb) and its effect on immune biomarkers and exploratory clinical outcomes in participants with sepsis-associated immunopathy.

**Methods:** Participants with sepsis/septic shock and absolute lymphocyte count <=1100 cells/μ L received BMS-936559 i.v. (10–900mg; n=20) or placebo (PBO; n=4) + standard of care and were followed for 90d. Primary endpoints were death and adverse events (AEs); secondary endpoints were monocyte (m)HLA-DR levels and clinical outcomes.

**Results:** Apart from the treated group being older (median 62y treated pooled vs 46y PBO) and sicker ([>=]3 organ dysfunctions: 55% treated pooled vs 25% PBO), baseline characteristics were comparable. 6/24 (25%) participants died (10mg: 2/4 [50%]; 30mg: 2/4 [50%]; 100mg: 1/4 [25%]; 300mg: 1/4 [25%] 900mg: 0/4; PBO: 0/4). All participants had AEs (grade 1–2: 75%), with one participant (30mg) having potentially drug-related AEs (grade 1–2 increases in amylase, lipase and LDH). 3/20 (15%) treated pooled and 1/4 (25%) PBO had a serious AE, with none deemed drug-related. AEs of special interest (AEOSI, i.e. potentially immune-related) occurred in [>=]1 participant per group, with diarrhea (33%) the most common. All but 3 AEOSI (1 lung infiltration, 2 diarrhea) were grade 1–2. At the two highest doses there was a trend toward an increase in mHLA-DR expression (>5000 mAb/cell) that persisted beyond 30d. No clear dose-relationship or between-group difference in clinical outcomes (duration of organ support, viral reactivation, ICU/hospital length of stay) was seen.

**Conclusions:** In this sick population, BMS-936559 was well tolerated. There were no AEs indicative of an excessive drug-induced pro-inflammatory state. At higher doses, a trend toward sustained restoration of mHLA-DR expression was seen. These findings justify further study of PD-1/PD-L1 inhibitors in sepsis.

## P107 Effects of a non-neutralizing humanized monoclonal anti-adrenomedullin antibody in a porcine two-hit model of hemorrhage and septic shock

### C Thiele^1^, TP Simon^1^, J Szymanski^1^, C Daniel^2^, C Golias^1^, J Struck^3^, G Marx^1^, T Schürholz^4^

#### ^1^Uniklinik RWTH Aachen, Aachen, Germany,^2^Universität Erlangen-Nürnberg, Erlangen, Germany,^3^Adrenomed AG, Hennigsdorf, Germany,^4^Universitätsmedizin Rostock, Rostock, Germany

**Introduction:** Adrenomedullin (ADM) is a vasoactive peptide improving endothelial barrier function in sepsis, but may cause hypotension and organ failure. Treatment with an ADM monoclonal antibody (mAB) showed improvement in murine sepsis models. Here, we tested effects of the humanized anti-ADM mAB Adrecizumab (AC) in a porcine two hit model of hemorrhagic (HS) and septic shock (SSH).

**Methods:** In a randomized, blinded study 12 German Landrace pigs (31+/-2 kg) were bled to half of baseline MAP for 45 minutes (HS). SSH was induced using an E.coli clot (7-9x10^11CFU/kg BW) placed into the abdominal cavity 6 hours after HS. Animals received either 2 mg/kg BW Adrecizumab or vehicle (VH) immediately after SSH induction. After 4 hours, resuscitation was initiated using balanced crystalloids and noradrenalin to maintain a CVP of 8-12 mmHg, a MAP >65mmHg and a ScvO2 >70% for another 8 hours. Hemodynamics, laboratory parameters and kidney histology were assessed. General linear model, MWU or Chi2 test were used for statistics where appropriate. P<0.05 was considered significant.

**Results:** Volume resuscitation was significantly lower in the AC compared to VH group (5300 vs. 6654ml; p=0.036). Vasopressor therapy was necessary in significantly less animals in the AC group (33 vs. 100%; p=0.014). Horowitz index was higher in the AC group (375 vs 286mmHg, p=0.055). Kidney histology showed significantly lower granulocytes in both cortex (9.1 vs. 31.1 n/mm_2_; p=0.02) and medulla (19.3 vs 53.0 n/mm_2_; p=0.004) in AC treated animals. After induction of sepsis, plasma ADM increased immediately in both groups, but increased quicker and more pronounced in the AC group (p=0.003 for time*group effect).

**Conclusions:** In this two hit shock model treatment with Adrecizumab overproportionally increased plasma ADM levels. Hemodynamics and pulmonary function were improved and histological kidney damage was reduced. Thus, therapy with Adrecizumab may provide benefit in septic shock, and clinical investigation candidate is warranted.

## P108 Prognosis of patients excluded by the definition of septic shock based on their lactate levels after initial fluid resuscitation: a prospective multi-center observational study

### B Ko^1^, W Kim^2^, T Lim^1^

#### ^1^Hanyang University Hospital, Seoul, Korea, Seoul, South Korea,^2^University of Ulsan College of Medicine, Asan Medical Center, Seoul, South Korea

**Introduction:** Lactate levels should be measured after volume resuscitation (as per the Sepsis-3 definition) [1]. However, currently, no studies have evaluated patients who have been excluded by the new criteria for septic shock. The aim of this study was to determine the clinical characteristics and prognosis of these patients, based on their lactate levels after initial fluid resuscitation.

**Methods:** This observational study was performed using a prospective, multi-center registry of septic shock. We compared the 28-day mortality between patients who were excluded from the new definition (defined as <2 mmol/L after volume resuscitation) and those who were not (lactate level >=2 mmol/L after volume resuscitation), from among a cohort of patients with refractory hypotension, and requiring the use of vasopressors.

**Results:** Of 567 patients with refractory hypotension, requiring the use of vasopressors, 435 had elevated lactate levels, while 83 did not have elevated lactate levels (neither initially nor after volume resuscitation), and 49 (8.2%) had elevated lactate levels initially, which normalized after fluid resuscitation (Fig. 1). Thus, these 49 patients were excluded by the new definition of septic shock. Significantly lower 28-day mortality was observed in these patients than in those who had not been excluded (8.2% vs 25.5%, p=0.02).

**Conclusions:** It seems reasonable for septic shock to be defined by the lactate levels after volume resuscitation, however due to small sample size further large scale study is needed.

**References**

1. Shankar-Hari M et al. JAMA 315(8):775-787, 2016.

**Table 1 (abstract P108).** Comparison of outcomes between patients with restored perfusion and those compatible with the Sepsis-3 definition

Outcomes	Sepsis with restored perfusion (n=49)	Sepsis with restored perfusion (n=49)	p
28-day mortality	4 (8.2%)	111 (25.5%)	0.02
In-hospital mortality	6 (12.2%)	131 (30.1%)	0.03
SOFA score	6.0 (4.0–8.0)	7.0 (5.0–9.0)	0.09
Max SOFA score	9.0 (6.0–10.0)	9.0 (7.0–12.0)	0.03
APACHE II	21.0 (15.0–26.0)	21.0 (16.0–27.0)	0.92
ICU stay (day)	4.0 (3.0–5.3)	5.0 (3.0–9.0)	0.26
Hospital stay (day)	12.0 (7.0–16.0)	14.0 (7.0–26.0)	0.26

**Fig. 1 (abstract P108).**
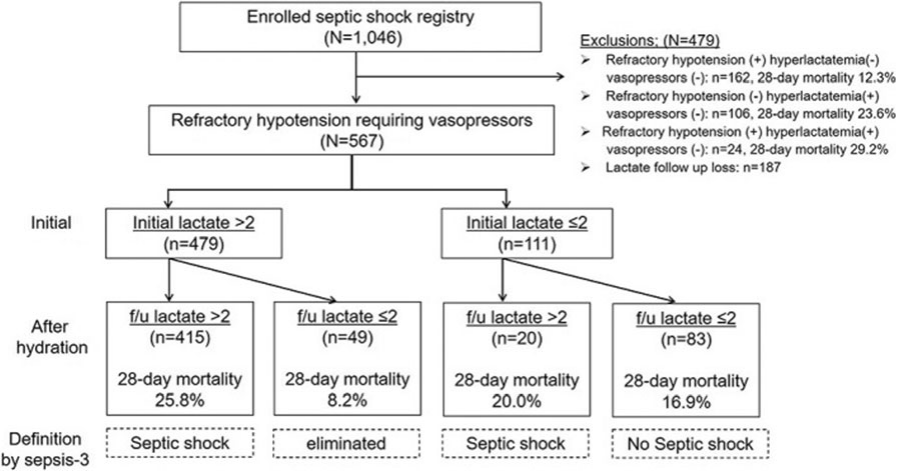
Patient Flow Diagram

## P109 Apoptotic cells induce cytokine homeostasis following LPS treatment

### D Mevorach

#### Hadassah-Hebrew University, Jerusalem, Israel

**Introduction:** Allocetra™, donor leukocytes containing early apoptotic cells and no necrotic cells, was shown as safe and potentially efficacious for the prevention of aGVHD(Mevorach et al. BBMT 2014). We tested the effects of early apoptotic cells on cytokines/chemokines of patients with aGVHD, and in mice treated with LPS and IFN-g.

**Methods:** LPS and IFN-g were used to trigger cytokine/chemokine release in vitro and in vivo in mice, and in patients treated for aGVHD. Cytokines/chemokines were evaluated in 13 patients. Mouse and human IL-1ß, IL-2 to 10, IL-12p70, IL-13, IL-15, IL-17A, IL-22, IL-27, Il-31, IL-32, IP-10, RANTES, GRO, IFN-g, GM-CSF, TNF-a, MIP-1a, MIP-1ß, MIP-2, MCP-1, MCP-3, MIG, ENA-78, were evaluated (Luminex technology, Merck Millipore). The IFN-g effect was evaluated by STAT1 phosphorylation.

**Results:** Significant downregulation (p<0.01) of about 30 pro- and anti-inflammatory cytokines, including IL-6, IP-10, TNF-a, MIP-1a, MIP-1ß, IL-10, was documented. IFN-g effect on macrophages and dendritic cells was inhibited at the level of phosphorylated STAT1. IFN-g-induced expression of CXCL10 and CXCL9 in macrophages was reduced. Patients treated in vivo with higher dosages of apoptotic cells had lower cytokine/chemokine levels compared to those treated with lower levels, and in inverse correlation to aGVHD staging. In vitro binding of apoptotic cells to LPS was documented.

**Conclusions:** The cytokine storm is significantly modified towards homeostasis following apoptotic cell treatment. The mechanism is multifactorial and was shown to include TAM receptor triggering, NFkb inhibition, and LPS binding. These results together with previous studies showing significantly higher murine survival in sepsis models of LPS and cecal ligation puncture suggest that apoptotic cells may be used to treat patients with sepsis. A multicenter clinical trial in septic patients is planned in 2018.

## P110 Efficacy of continuous haemodiafiltration using a polymethylmethacrylate membrane haemofilter (PMMA-CHDF) in the treatment of sepsis and acute respiratory distress syndrome (ARDS)

### M Sakai

#### 12628 tomioka takeo-cho, Takeo, Japan

**Introduction:** CHDF using with a polymethymethacrylate membrane is currently widely applied for non-renal indications in Japan, this technique is used in the treatment not only of patients with sepsis but also of those with cytokine-induced critical illness such as ARDS and pancreatitis. This study aimed to investigate the clinical efficacy of PMMA-CHDF in the treatment of a patients with sepsis and ARDS.

**Methods:** Seventy- five patients diagnosed with sepsis (ARDS[n=30], Pyelonephritis [n=10], Cholangitisn [n=10], Tsutugamusi in Scrub typhus disease[n=1], Snake Mamushi biten[n=1], haemophagocytic syndrome[n=1],anti neutrophil cytoplasmic antibody(ANCA)lung disiese[n=1],beriberi heart disease[n=1] and unknown causes[n=18]) were enrolled in this study between August 2010 and March 2017. The common cause for ARDS in elderly patients aspiration pneumonia in elderly patients.

**Results:** Following initiation of PMMA-CHDF teatment, early improvement of haemodynamics was observed, along with an increase in the urine output. The average survival rates of patients were75.6%. The low survival rate among diseases 35% belonged to the Unknown group. The highest survival rate for patients with ARDS was 95%. Moreover, the urine output significantly increased in survival group.

**Conclusions:** The present study suggests that cytokine-oriented critical care using PMMA-CHDF might be effective the treatment of sepsis and ARDS, particularly,in the treatment of ARDS associated with aspiration pneumonia in elderly patients.

## P111 The polymyxin b immobilized fiber column direct hemoperfusion has an effect for septic shock but has no effect on sepsis: a cohort study and propensity-matched analysis

### K Hoshino^1^, H Ishikura^1^, Y Irie^1^, K Muranishi^1^, F Kiyomi^1^, M Hayakawa^2^, Y Kawano^1^, Y Nakamura^1^

#### ^1^Fukuoka University Hospital, Fukuoka, Japan,^2^Hokkaido University Hospital, Sapporo, Japan

**Introduction:** Many patients with sepsis receive Polymyxin B immobilized fiber column direct hemoperfusion (PMX-HP) as a rescue therapy. Recently, we are reported that PMX-HP reduces all-cause hospital mortality in patients with septic shock [1]. However, it is unclear that whether PMX-HP reduce not only septic shock patients but also sepsis patients. A purpose of this study is to clarify an effect of PMX-HP for the prognosis of patients with sepsis.

**Methods:** Data from patients admitted for severe sepsis (including septic shock) to Japanese ICUs were retrospectively collected from Jan 2011 to Dec 2013 through the Japan Septic Disseminated Intravascular Coagulation (J-SEPTIC DIC) study data base set. We analyzed the potential benefit of PMX-DHP using a propensity score–matched (1:1) cohort analysis in patients with sepsis.

**Results:** Of 2,952 eligible patients, 664 underwent PMX-HP. Propensity score matching created a matched cohort of 740 patients (370 pairs with and without PMX-HP). There was no significant difference between the two matched cohorts for the hospital and ICU mortality [Odds ratio (OR); 1.20, 95% confidence interval (CI), 0.93-1.52, p=0.150), OR; 0.98, 95%CI; 0.79-1.21, p=0.828, respectively].

**Conclusions:** In this demonstrated that PMX-HP had a no benefit on hospital and ICU survival when compared with conventional management (non-PMX-HP) in matched patients with sepsis. From this study we concluded that PMX-HP has an effect for septic shock but has no effect on sepsis.

**Reference**

1. Nakamura Y et al. Crit Care 21(1):134, 2017

## P112 A real world experience of novel extracorporeal cytokine adsorption therapy (Cytosorb) to manage sepsis and septic shock patients at tertiary care hospital in India

### SK Garg, D Juneja, O Singh

#### Max Hospital, New Delhi, India

**Introduction:** Sepsis and septic shock with very high mortality rate (30-50%) is associated with an inflammatory cascade and responsible for multiple organ dysfunction [1]. Extracorporeal cytokine adsorption device (Cytosorb) is an adjunctive therapy to modulate systemic inflammation in Sepsis and Septic shock patients. This retrospective data analysis from real world provides more insight in management of septic shock, as they reflect the management of patients in heterogeneity routine clinical settings.

**Methods:** In this retrospective study, data of 30 septic shock patients with SOFA score >10 admitted in ICU treated with hemoadsorption (Cytosorb) therapy was collected and analysed.

**Results:** 30 patients (22 Male and 8 Females; mean age 59.33 yrs.) were administered cytosorb in addition to standard of care, an average of 1.1 cartridge was used for every patients for 4-6 hrs. Out of 30, 13 patients showed substantial reduction of 40% in SOFA score. 7 out of 30 patients had their MAP above 70mm hg after Cytosorb treatment and vasopressors were reduced to 50% from baseline. 10 & 15 patients showed good improvement in Serum Lactate 44.2% (mean 6.64 Vs 3.37) and serum creatinine 43.5% (2.80 Vs 1.58) respectively.

**Conclusions:** We conclude that substantial difference was seen in Serum lactate, Serum Creatinine and vasopressor requirement after cytosorb therapy, however multi organ failure had already set in all patients before initiating cytosorb therapy, hence the above mentioned outcomes were not demonstrated in all patients.

**Reference**

1. Kogelmann et al. Crit Care 21,74, 2017

## P113 Early cytokine adsorption in septic shock (ACESS-trial): results of a proof concept, pilot study

### N Öveges^1^, F Hawchar^1^, I László^1^, M Forgács^1^, T Kiss^1^, P Hankovszky^1^, P Palágyi^1^, A Bebes^1^, B Gubán^1^, I Földesi^1^, Á Araczki^1^, M Telkes^1^, Z Ondrik^1^, Z Helyes^2^, Á Kemény^2^, Z Molnár^1^

#### ^1^University of Szeged, Szeged, Hungary,^2^University of Pécs, Pécs, Hungary

**Introduction:** Overwhelming cytokine release often referred to as “cytokine storm” is a common feature of septic shock, resulting in multiple organ dysfunction and early death. Attenuating this cytokine storm early by eliminating cytokines may have some pathophysiological rationale. Our aim was to investigate the effects of extracorporeal cytokine removal (CytoSorb) therapy on organ dysfunction and inflammatory response within the first 48 hours from the onset of septic shock.

**Methods:** Patients with: sepsis of medical origin, on mechanical ventilation, noradrenaline >10mg/min, procalcitonin >3ng/mL and no need for renal replacement therapy, were randomized into CytoSorb and Control groups. CytoSorb therapy lasted for 24 hours. In addition to detailed clinical data collection, blood samples were taken to determine IL-1, IL-1ra, IL-6, IL-8, IL-10, TNF-α, PCT, CRP levels. At this stage of the study, only PCT and CRP levels were analyzed. Data were recorded on enrollment (T0) then at T12, T24, and T48 hours. For statistical analysis, Mann-Whitney test was used.

**Results:** Twenty patients were randomized into CytoSorb (n=10), and Control-groups (n=10). Overall organ dysfunction as monitored by SOFA and MODS scores did not differ between the groups. In the CytoSorb-group noradrenaline requirement (T0=76±63, T24=48±43, T48=23±24 μg/min, p=0.016) showed a significant reduction in the CytoSorb-group but not in the Control-group. There was no difference in CRP, but PCT decreased significantly in the CytoSorb-group (T0=147.8±216.3, T48=78.9±140.1 mmol/L, p=0.004). Lactate decreased significantly in both groups.

**Conclusions:** These results suggest that a 24-hour long CytoSorb treatment at the early stages of septic shock has significant beneficial effects on noradrenaline requirement and PCT concentrations within the first 48 hours. Based on the results of this current pilot study we are planning to design a prospective randomized multicenter trial.

## P114 Usage pattern of polymyxin-b in clinical practice for critical care management in Indian patients: a multicentric prospective observational study

### Y Mehta^1^, K Zirpe^2^, R Pande^3^, S Katare^4^, A Khurana^4^, S Motlekar^4^, A Qamra^4^, A Shah^4^

#### ^1^Medanta The Medicity, Gurgaon, India,^2^Ruby Hall Clinic, Pune, India,^3^BLK Super Speciality Hospital, New Delhi, India,^4^Wockhardt Limited, Mumbai, India

**Introduction:** Emergence of bacterial pathogens with acquired resistance to almost all available antimicrobial agents has severely jeopardized therapeutic choices in the last decade. Furthermore, treatment of MDR gram negative infections has been a major challenge in developing countries like India. Weak research pipeline has led to re-emergence of older antibiotics like Polymyxin B with limited clinical data [1]. This study aims to evaluate the utilization profile of Polymyxin B in Intensive care practice in India.

**Methods:** This on-going prospective, observational multi-centric study has been approved by IRBs and is being conducted in 3 tertiary care centers in India. The interim data of 101 patients, who received polymyxin B as part of intensive care management, was analysed for utilization profile in terms of demographics, indication, dosage regimen, safety and clinical outcomes.

**Results:** Patients with mean age 53.1±19.3 years, (67.7% males) had baseline APACHE II, SOFA and GCS scores of 17.2±5.3, 13.7±4.9 and 9.8±5.2, respectively. Majority of patients had sepsis involving pulmonary (51.6%), neurological (25.3%) and cardiovascular (21.1%) systems (Fig. 1, Table 1). Commonest organisms isolated were *K. pneumoniae* and Acinetobacter spp. Major reasons for intensive care management were hemodynamic instability (52.5%), respiratory failure (42.6%), renal failure (14.9%) and trauma/surgery (37.6%). Mean daily Polymyxin B dose was 1.1±0.4 MIU administered for up to 14 days in 73.2% patients. Clinical response was observed in 67.2% patients. Clinical deterioration of serum Creatinine and all-cause mortality was seen in 28.8% and 32.7% patients, respectively.

**Conclusions:** In light of good clinical response, Polymyxin B can be considered as a feasible option in the intensive care management of gram negative infections.

**Reference**

1. Garg SK et al. Critical Care Research and Practice, 3635609:1-10, 2017

**Table 1 (abstract P114).** Distribution of body systems involved in patients receiving Polymyxin-B therapy

System/s Involved	Percentage of cases
Pulmonary	51.6
Neurological	25.3
Cardio-vascular	21.1
Abdominal	18.9
Soft Tissue	9.5
Renal	6.3
Miscellaneous	25.2

**Fig. 1 (abstract P114).**
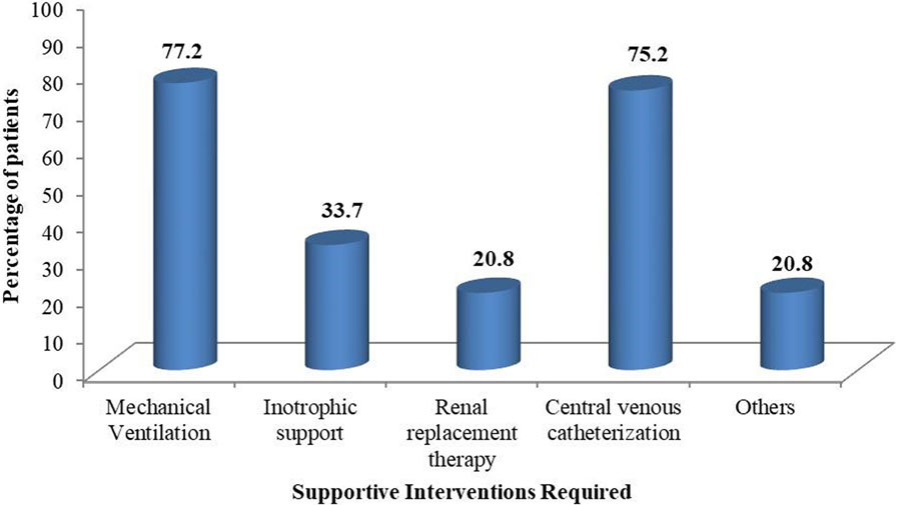
Distribution (Percentage) of Supportive interventions required

**Fig. 2 (abstract P114).**
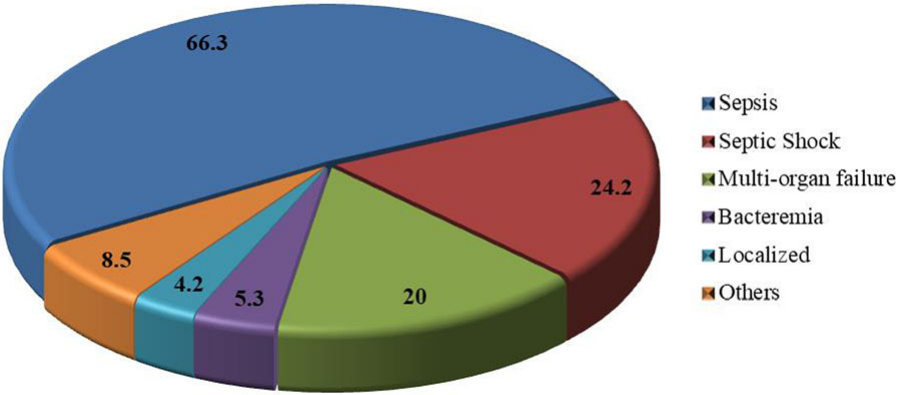
Distribution (percentage) of severity of bacterial infection

## P115 Determinants & outcomes of polymyxin-b use: single center critical care experience, India

### K Zirpe, A Deshmukh, S Patil

#### Ruby Hall Clinic, Pune, India

**Introduction:** Polymyxin B, though available since 1950s, was side-lined due to availability of safer antimicrobials. However, surge of multi-drug resistant gram negative infections has triggered resurgence of these older antimicrobials, despite paucity of available clinical data [1]. This paper reports clinical experience of determinants and outcomes associated with Polymyxin B therapy at our institute

**Methods:** This study prospectively captures clinical and drug usage profile of patients receiving Polymyxin B, from their medical records, at our tertiary care hospital in Pune, India. The analysis of first 28 completed patients has been summarized here.

**Results:** The analysed patients (n=28) included 78.6% males, with mean age of 45.7 (±19.9) years. Polymyxin B was most commonly used in sepsis involving respiratory (63%) and abdominal (37%) systems. All the patients were treated in intensive care setup, among which 96.4% required mechanical ventilation (Fig. 1). Most of patients were initiated on Polymyxin B presumptively and *Klebsiella pneumoniae* and Acinetobacter spp were most common isolated organisms. Polymyxin B was initiated using bolus dose (equivalent to total daily dose) and administered at mean daily dose of 0.97±0.33 MIU in two divided doses. Majority (66.7%) of patients received Polymyxin B therapy for <7 days, (mean duration of therapy, 5.37±4.22 days) (Fig. 2). Meropenem (85.7%) was most commonly co-administered antimicrobial. No unlisted adverse drug reactions were reported. The all-cause mortality rate was 28.6%

**Conclusions:** Our experience suggests Polymyxin B to be favourable choice for management of MDR gram-negative infection. Further systematic evaluations are required for cementing therapeutic status of Polymyxin B in multi-drug resistant gram negative infections in ICU set-up.

**Reference**

1. Garg SK et al. Critical Care Research and Practice, 3635609:1-10, 2017

**Fig. 1 (abstract P115).**
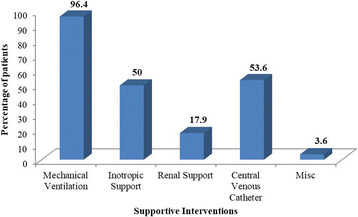
Distribution of supportive interventions required in Intensive Care Setup

**Fig. 2 (abstract P115).**
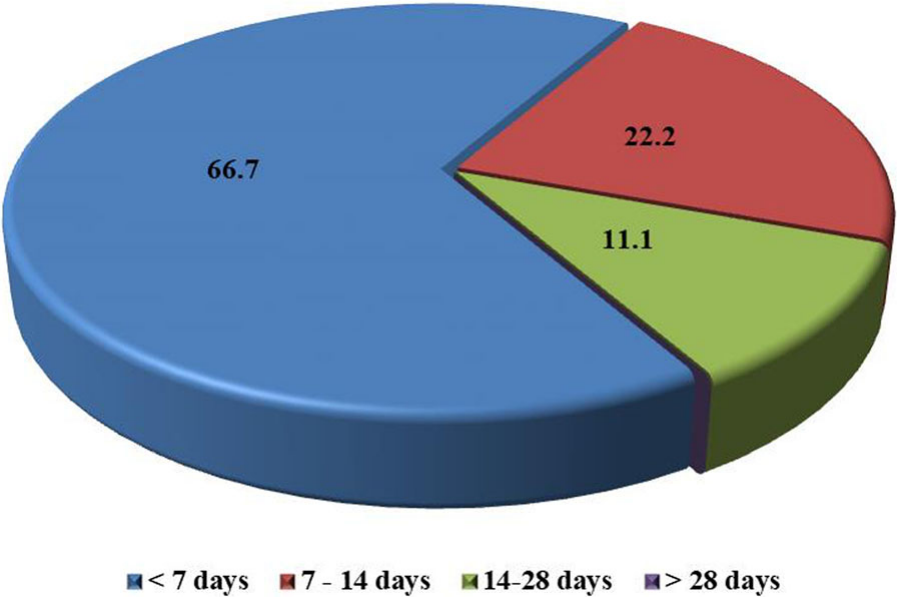
Distribution of duration of Polymyxin B therapy

## P116 Polymyxin b usage and outcomes: real world experience from an intensive care unit of tertiary care hospital in northern India

### Y Mehta, C Mehta, S Nanda, J Chandel, J George

#### Medanta The Medicity, Gurgaon, India

**Introduction:** Limited antimicrobial agents and dry pipeline has compelled the intensivists to revisit older antibiotics such as Polymyxins, Fosfomycin, etc. for MDR/XDR gram negative infections. With limited clinical data available, especially from India, we planned to assess the drug utilization pattern of Polymyxin B in our ICU settings.

**Methods:** After Institutional Ethics Committee approval, this prospective observational study was initiated to collect the usage, demography, clinical presentations, indications, bacterial species isolated, treatment regimens, concomitant antimicrobials used and clinical outcomes of Polymyxin B based regimens. The data is collected using structured case record forms and summarized using descriptive statistics. This is the interim analysis of first 73 patients of the ongoing study.

**Results:** The mean age of patients (n=73, 62.5% males) was 56.0 (±18.26) years with mean baseline APACHE II score of 17.1 (±5.44). Polymyxin-B was most commonly prescribed for Sepsis (72.1%) and the most commonly system involved was Respiratory (47.1%). The most common bacterial species isolated was *Klebsiella pneumoniae* (23.1%) followed by Acinetobacter spp & *Pseudomonas aeruginosa* (5.5% each) (Table 1). The mean daily dose of Polymyxin-B was 1.19 (±0.39) MIU with mean duration of therapy of 15.36 (±14.2) days (Fig. 1). Clinical response was observed in 63.6% patients (bacteriological cure 60%) (Fig. 2). All-cause mortality was 34.2%. 71.2% of patients did not have clinically significant increase in serum creatinine levels, though 31.1 % of these had raised serum creatinine at baseline. No unexpected treatment emergent adverse events were reported.

**Conclusions:** This data suggests Polymyxin B to be an effective antimicrobial agent with good clinical response and acceptable all-cause mortality in an Indian intensive care setup.

**Table 1 (abstract P116).** Profile of bacterial species isolated in patients receiving Polymyxin-B based antimicrobial regimen

Species	No. of cases (N = 73)	Percentage of cases
Klebsiella pneumoniae	17	23.3
Acinetobacter baumannii	4	5.5
Pseudomoas aeruginosa	4	5.5
E.coli & Enterobactericae family	6	8.2
Streptococcus	2	2.7
Staphylococcus	2	2.7
Miscellaneous or Pus cells (No isolation)	6	8.2

**Fig. 1 (abstract P116).**
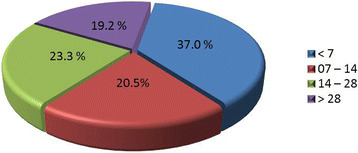
Duration of Polymyxin B Therapy (Days)

**Fig. 2 (abstract P116).**
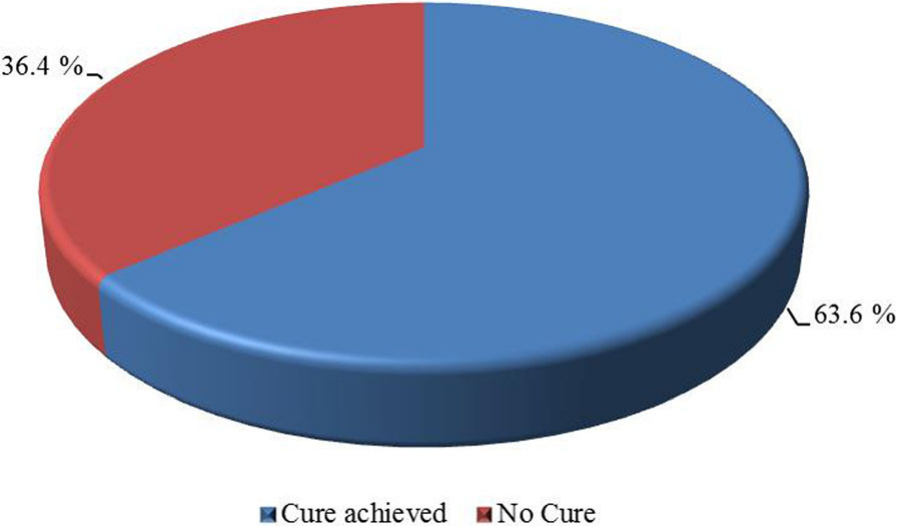
Response rate with Polymyxin B based antimicrobial therapy

## P117 Effect of selective plasma exchange for treatment of patients with severe sepsis due to multiorgan failure

### R Iscimen, P Rahimi, HZ Dundar, N Kelebek Girgin, F Kahveci

#### Uludag University, Bursa, Turkey

**Introduction:** The mortality rate in severe sepsis ranges between 30-50% and independent liver and renal dysfunction significantly affect intensive care mortality, 4 or more organ failure causes mortality to exceed 90%. Selective plasma exchange is the most commonly used artificial liver support system and effectively removes albumin bound toxins. Selective plasma Exchange (SPE) eliminates low- and medium-molecular weight materials such as cytokines, albumin-bound toxins etc. Therefore, SPE is thought to decrease sepsis mortality by preventing multiple organ failure. In this study, we investigate the effect of selective plasma exchange on the treatment of patients with multiorgan failure and septic shock.

**Methods:** Selective plasma exchange was performed with Evaclio™(2c-20) in patients diagnosed with septic shock due to multiple organ failure. Pre-and post-treatment laboratory values, Sequential Organ Failure Assessment (SOFA) scores and 28days ICU mortality were calculated. Demographic data of patients are shown on the Table 1.

**Results:** After ethics committee approval 64 patients(29female/35male) diagnosed with septic shock were included in the study, 125 sessions of selective plasma exchange were performed totally. Value changes before and after the first SPE are shown on the Table 2. There was statistically significant decrease in respiratory, coagulation, renal and liver SOFA scores by SPE. ICU mortality rate was 65%.

**Conclusions:** Selective plasma exchange is a technique developed to eliminate toxins and cytokines and appear be useful in reducing severe sepsis mortality by decreasing SOFA score and preventing multiple organ failure.

**Table 1 (abstract P117).** Demographic data

Age	47.54±17.16
Sex	Male:35 Female:29
APACHE II score	23.69±11.35
Hemodialysis	28

**Table 2 (abstract P117).** SOFA scores before and after SPE

SOFA score	Before	After	P Value
Renal	0.82±2.25	0.56±0.80	<0.001
Liver	3.55±0.77	3.50±0.69	<0.001
Coagulation	1.43±1.36	1.34±1.22	<0.001
Respiratory	0.98	0.96	<0.001
Cardiovascular	1.33±1.88	1.28±1.77	<0.001

## P118 Troponin elevation in septic shock: a study of the prognosis

### A Chaari, W Assar, K Abdelhakim, K Bousselmi, M El Koumy, V Kumar, V Kauts, M Al Ansari

#### King Hamad University Hospital, Bussaiteen, Bahrain

**Introduction:** The aim of the current study is to assess the prognostic value of increased troponin on the outcome of patients with septic shock.

**Methods:** Retrospective study conducted between 01/01/217 and 30/07/217. All adult patients admitted with septic shock were screened for inclusion. Patients with known ischemic heart disease were excluded. Demographic data, baseline clinical and laboratory findings were recorded. Troponin I level on admission and the highest troponin level during intensive care unit (ICU) stay were collected. The echocardiographic findings on admission were reviewed. Two groups (survivors and non-survivors) were compared

**Results:** Thirty-one patients were included in the study. Median age was 71[62-78] years. Median APACHEII score was 19 [16-26]. Troponin I level on admission was 0.06 [0 - 0.43] ng/ml. The highest troponin level during ICU stay was 0.09 [0.36-1.11] ng/ml. Nine patients (29 %) had wall motion abnormalities. Median left ventriculqr ejection fraction (LVEF) was 55 [30 - 60] %. Median duration of ICU stay was 4 [2.8-12.8] days. ICU mortality was 22.6 %. Troponin level on admission was comparable between survivors and non-survivors (respectively 0.08 [0-0.6] and 0.01 [0-0.16]; p=0.242) whereas the highest troponin during ICU stay was significantly higher in non-survivors (1 [0.36-11.1] versus 0.31 [0.06 - 0.65]; p=0.036). LVEF was comparable between survivors and non-survivors (respectively 55 [29-60] versus 56 [55- 58] %; p = 0.547). The highest troponin was not identified by the multivariate analysis as independent factor predicting ICU mortality (OR=1.1, CI95% = 1.1 [0.92-1.16]; p=0.489). The only factor identified by the analysis was acute kidney injury requiring renal replacement therapy (OR=105, CI95% [5.5-198]; p=0.002).

**Conclusions:** Increased serum troponin I level is common in patients with septic shock. Our study suggests that the increase of troponin is higher in non-survivors.

## P119 Real time needle visualisation pericardiocentesis in acute traumatic pericardial tamponade

### O Adi, A Azma Haryaty

#### Hospital Raja Permaisuri Bainun, Perak, Malaysia

**Introduction:** Blind pericardiocentesis leading to low success rate and high complication rates such as ventricular wall or oesophageal perforations, pneumothorax or upper abdominal organ injury.Real time needle visualisation is allowing us to avoid this major complication [1].

**Methods:** We presented 2 cases of acute traumatic cardiac tamponade secondary to severe chest injury. Both patients presented with haemodynamic instability and echocardiographic features of pericardial tamponade. Pericardiocentesis under ultrasound guidance at left parasternal area with needle directed from medial to lateral technique were performed(Fig. 1). Real time needle tip visualisation done throughout the procedure(Fig. 2a). Needle placement in pericardial space was confirmed with agitated saline and guidewire visualisation(Fig. 2b). Pigtail catheter was inserted and blood was aspirated until the patient were haemodynamically improved. Repeated ultrasound was done to confirm the absence of ultrasonographic features of tamponade and complications.

**Results:** We demonstrated a successful real time needle visualisation ultrasound guided pericardiocentesis in 2 cases acute traumatic pericardial tamponade. Procedural time (time from needle piercing the skin to time needle entering the pericardium) in both cases were less than 1 minute. Post procedural ultrasound confirmed no major complications.

**Conclusions:** The real time needle visualisation using ultrasound was important to reduce major complications during pericardiocentesis. The safety of the highly invasive procedure can be improved with real time needle visualisation.

**Reference**

Osman A et al. Eur J Emerg Med (in press), 2017

**Fig. 1 (abstract P119).**
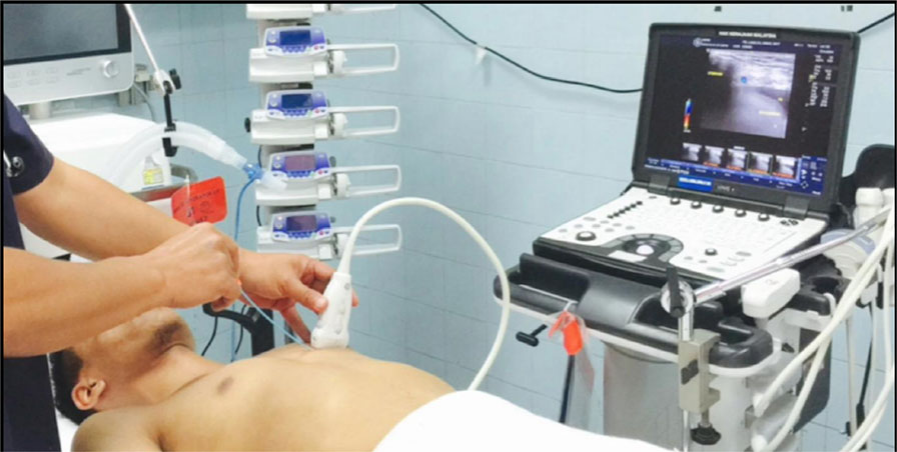
Parasternal approach with needle directed from medial to lateral technique.

**Fig. 2 (abstract P119).**
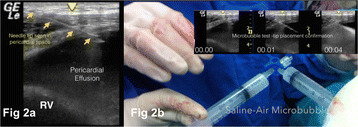
a: Real time needle visualisation. b: Agitated saline or micro bubble test for tip placement confirmation

## P120 A novel method for early identification of cardiac tamponade in patients with continuous flow left ventricular assist devices by use of sublingual microcirculatory imaging

### S Akin, C Ince, C Den Uil, A Struijs, R Muslem, I Ocak, G Guven, AA Constantinescu, OI Soliman, F Zijlstra, A J.J.C Bogers, K Caliskan

#### Erasmus MC, University Medical Center Rotterdam, Rotterdam, Netherlands

**Introduction:** Diagnosis of cardiac tamponade post continuous-flow left ventricle assist devices (cf-LVADs) is challenging due to missing pulsatility. Recent case study of sublingually microcirculation with incident dark-field imaging (IDF) provide a new improved imaging for clinical assessment of cardiac tamponade in a patient with cf-LVAD. We sought to examine the changes in microvascular flow index (MFI) as a sign of cardiac tamponade following LVAD implantation.

**Methods:** Off-site quantitative analysis of sublingual microcirculation clips with Automated Vascular Analyses software (AVA; MicroVision Medical©), and the velocity distributions followed during admission till discharge in patients with end-stage heart failure treated with cf-LVAD complicated by cardiac tamponade.

**Results:** Eleven out of thirty LVAD implantations, 9 males, mean age 58 ± 10 years, April 2015 to January 2017, ((8 Heart Mate 3 (HM 3) and 3 HeartMate II (HM II) (Thoratec Corp., CA)), were complicated by rethoracotomy due to early postoperative cardiac tamponade within 1 week. There sublingual microcirculation was examined by a novel incident dark-field imaging (IDF) before and daily post-LVAD implantation. Pre-LVAD microcirculation was typical for heart failure, characterized by slowly, sludging movement of red blood cells (RBCs), (Fig. 1A arrows). Directly after implantation, a normal microcirculatory flow was seen with a high RBCs velocity (Fig. 1B). On the day of tamponade the patients were stable except for severe failure of microcirculation as reflected by drop in MFI (Fig. 1C) and congestion in venules (* in Fig. 1C). In 8 out of 11 patients there was a significant drop in MFI before tamponade was clinically recognized (p<0.05). Shortly after rethoracotomy a quick restoration of microcirculatory flow has been found.

**Conclusions:** Sublingual microcirculation imaging is a simple and sensitive non-invasive tool in early detection of cardiac tamponade.

**Fig. 1 (abstract P120).**
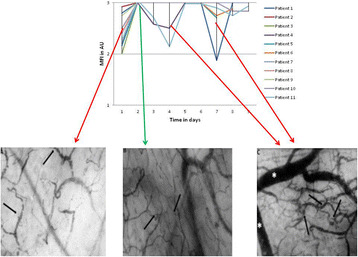
See text for description

## P121 Survey on the use of cardiovascular drugs in shock (ucards) – inotropes

### T Kaufmann^1^, I Van der Horst^1^, JL Teboul^2^, T Scheeren^1^

#### ^1^University Medical Centre Groningen, Groningen, Netherlands,^2^Hôpitaux Universitaires Paris-Sud, Paris, France

**Introduction:** Treatment decisions on patients with shock lack consensus. In an international survey we aimed to evaluate the indications, current practice, and therapeutic goals on the use of inotropes in the treatment of shock states.

**Methods:** From November 2016 to February 2017 an anonymous 27-question web-based survey was accessible to members of the European Society of Intensive Care Medicine (ESICM). A total of 27 questions focused on the profile of respondents, and the triggering factors, first line choice, dosing, timing, targets, additional treatment strategy, and suggested effect of cardiovascular drugs.

**Results:** A total of 827 physicians responded. As detailed in Table 1, the respondents think that dobutamine is first-line inotrope to increase cardiac pump function (N=695, 84%) and should be started when signs of hypoperfusion or hyperlactatemia despite adequate use of fluids and vasopressors in the context of low left ventricular ejection fraction are present (N=359, 43%). The most accepted target was an adequate cardiac output (N=369, 45%). The combination of noradrenaline and dobutamine was preferred to single treatment with adrenaline mainly due to possibility to titrate individually (N=366, 44%). The main reason for adding another inotrope was to use synergistic effects of two different mechanisms of action (N=229, 27%). According to respondents, phosphodiesterase-inhibitors should be used in the treatment of predominant right heart failure because of prominent vasodilatory effect on the pulmonary circulation (N=360, 44%). They also believe levosimendan is the only inotrope that does not increase myocardial oxygen demand (N=350, 42%). Vasodilators are used in cardiogenic shock to decrease left ventricular afterload (N=244, 30%). There is no experience or no opinion about the use of ß-blockers in shock states (N=268, 32%).

**Conclusions:** This web-based survey provided latest trends on inotrope use in shock states which showed considerable diversity among respondents in opinions about its use.

**Table 1 (abstract P121).**
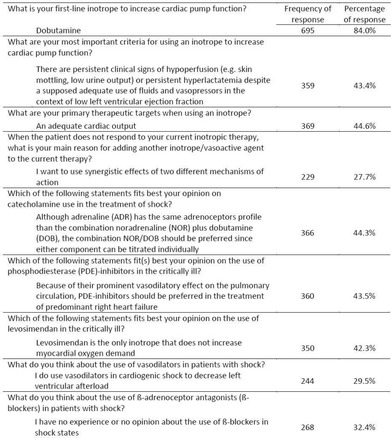
Survey questions on inotropes and other cardiovascular drugs with the most frequent response

## P122 Thermoshock: thermography assessment of thermal patterns in patients with shock: preliminary results

### M Tosi^1^, A Andreotti^1^, M Girardis^1^, F Despini^2^, A Muscio^2^, P Tartarini^2^

#### ^1^University Hospital of Modena, Modena, Italy,^2^University of Modena, Modena, Italy

**Introduction:** Recent literature data clearly indicated that in patients with shock the resuscitation of macro-circulation often does not match with microcirculation and tissue perfusion improvement.

Unfortunately, the bed-side assessment of regional perfusion remains difficult, particulary in critically ill patients. In the last years thermography has been used in different medical fields but no studies have been performed on the use of this technique in critically ill patients.

The aim of this study was to evaluate whether thermography is feasible and may provide useful data during resuscitation of patients with septic shock.

**Methods:** In 4 patients with septic shock we collected central systemic temperature and infrared images (FLIR-T640 digital camera) of limbs at 0, 3, 6 and 24 hours after shock occurrence. Thermal pattern distribution of the limbs was obtained by a specific analysis of the images (ThermaCAM™Researcher P). A systemic to peripheral temperature gradient called “∆ systemic-limb temperature” was calculated for each single temperature data collected.

**Results:** Macrocirculatory and perfusion parameters improved in all the patients throughout the study period: mean values of noradrenaline dose decreased from 0.21 to 0.13 γ/kg/min, mean MAP increased from 65 to 81 mmHg and mean blood lactate decreased from 6.6 to 4.2 mMol/L. The “∆ systemic-limb temperature” pattern showed an heterogenous time course in the 4 patients with a mean overall increase at 6 and 24 hours (Fig. 1).

**Conclusions:** As expected, the regional data obtained by thermography did not match with macrocirculatory and systemic perfusion parameters. The significance and the relationship between treatments and data observed will be investigated by appropriate studies.

**Fig. 1 (abstract P122).**
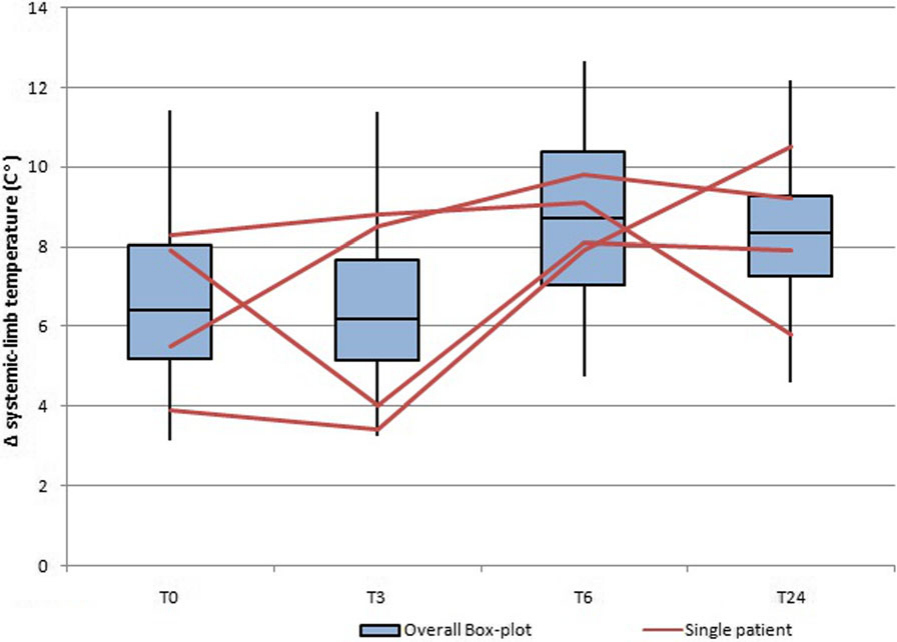
See text for description

## P123 Regional differences in the treatment of refractory septic shock – an analysis of the ATHOS-3 data

### M Abril^1^, A Khanna^2^, C McNamara^2^, D Handisides^3^, L Busse^4^

#### ^1^Emory University, Atlanta, GA, USA,^2^Cleveland Clinic, Cleveland, OH, USA,^3^La Jolla Phapmaceutical Company, La Jolla, CA, USA,^4^Emory St. Joseph’s Hospital, Atlanta, GA, USA

**Introduction:** Vasodilatory shock is a common syndrome with high mortality. Despite established care protocols, regional differences in treatment remain. We sought to characterize these differences using data from the recently published ATHOS-3 study [1].

**Methods:** Individual patient data were analyzed at baseline and at 48h for regional differences in demographics, clinical characteristics, and treatment patterns, and grouped according to four geographical areas: the United States (US), Canada (CA), Europe (EU) and Australasia (AU). P-values were calculated by Kruskal-Wallis tests for continuous data and chi-square tests for categorical data. Subsequent temporal analysis compared changes in the treatment of shock, indexed by changes in patient acuity level.

**Results:** Regional differences existed with respect to BMI (p=0.0076), albumin (p<0.0001), CVP (p=0.0383), MELD score (p=0.0191), APACHE II score (p=0.0007) and SOFA score (p=0.0076). Baseline norepinephrine (NE) and NE equivalent doses were significantly higher in EU (p<0.0001 and p=0.0494, respectively), and utilization of vasopressin was correspondingly lower (p<0.0001). At baseline, stress dose steroids were utilized to a greater extent in the US and CA (p=0.0011). Temporal analysis revealed differences in the utilization of vasopressin and steroids with changes in patient acuity: in EU, increasing acuity was associated with a lower utilization of vasopressin, and in CA, increased acuity was associated with a lower utilization of steroids. Steroid utilization was higher with increased level of acuity in AU and the US.

**Conclusions:** Significant differences in the treatment of vasodilitory shock exist globally, with important implications: (a) there are widespread differences of best practices, (b) heterogeneity may render global studies of shock difficult to interpret, and (c) outcomes may be improved through appropriate use of adjunctive therapies like non-catecholamine vasopressors or corticosteroids.

**Reference**

[1] Khanna et al. N Engl J Med; 377(5):419-430, 2017

## P124 Association of angiotensin II dose with all-cause mortality in patients with vasodilatory shock

### M McCurdy^1^, LW Busse^2^, MN Gong^3^, DW Boldt^4^, SN Chock^5^, R Favory^6^, KR Ham^7^, K Krell^8^, XS Wang^9^, LS Chawla^10^, GF Tidmarsh^10^

#### ^1^University of Maryland, School of Medicine, Baltimore, MD, USA,^2^Emory University, Atlanta, GA, USA,^3^Albert Einstein College of Medicine, Montefiore Medical Center, Bronx, NY, USA,^4^University of California, Los Angeles, Los Angeles, CA, USA,^5^Sunrise Hospital, Las Vegas, NV, USA,^6^CHU Lille, Critical Care Center and University of Lille School of Medicine, Lille, France,^7^Regions Hospital, University of Minnesota, St. Paul, MN, USA,^8^Eastern Idaho Regional Medical Center, Idaho Falls, ID, USA,^9^Duke University Medical Center, Durham, NC, USA,^10^La Jolla Pharmaceutical Company, San Diego, CA, USA

**Introduction:** Vasodilatory shock is associated with high risk of mortality. In the ATHOS-3 study, the addition of angiotensin II (Ang II) to standard vasopressors significantly increased mean arterial pressure (MAP) and decreased vasopressor utilization, with a trend towards improved survival to day 28. In this analysis of Ang II recipients in the ATHOS-3 study, we assess the relationship between different dose ranges of Ang II with MAP response and mortality.

**Methods:** Patients with persistent vasodilatory shock despite receiving >0.2 μg/kg/min of norepinephrine-equivalent dose vasopressors were randomized to receive IV Ang II, titrated per study protocol, or placebo with other vasopressors held constant for hours 0-3; for hours 3-48, all vasopressors could be titrated to maintain MAP. Ang II responsiveness was defined as the Ang II dose required to achieve a MAP of 75 mmHg. In this analysis, 28-day all-cause mortality was evaluated per prespecified categories based on the Ang II dose received 30 min after the start of dosing. These categories included “super-responders,” defined as patients who required physiological doses of Ang II (<=5 ng/kg/min), and patients who required higher doses.

**Results:** Among the 163 Ang II recipients, 79 (48.5%) were super-responders. Mortality at day 28 was 32.9% in super-responders vs 58.6% in patients requiring Ang II doses >5 ng/kg/min (n=84) and 53.9% in the placebo group (n=158). The hazard ratio for all-cause mortality in super-responders vs Ang II patients requiring higher doses was 0.45 (95% CI 0.28-0.72), p=0.0007; and 0.50 (95% CI 0.32-0.78), p=0.0018 for super-responders vs placebo.

**Conclusions:** Ang II super-responders, a large subgroup of Ang II recipients in ATHOS-3, had significantly reduced all-cause mortality at day 28 vs patients receiving placebo or higher doses of Ang II. Ang II doses <=5 ng/kg/min equates to a physiological level of Ang II, and may reflect a novel means of achieving normal homeostatic mechanisms to correct vasodilatory shock.

## P125 Outcomes in patients with acute respiratory distress syndrome receiving angiotensin II for vasodilatory shock

### L Busse^1^, T Albertson^2^, M Gong^3^, BT Thompson^4^, R Wunderink^5^, D Handisides^6^, G Tidmarsh^6^, L Chawla^6^

#### ^1^Emory St. Joseph’s Hospital, Atlanta, GA, USA,^2^University of California, Davis, Davis, CA, USA,^3^Montefiore Medical Center, Bronx, NY, USA,^4^Harvard Medical School, Boston, MA, USA,^5^Northwestern University, Feinberg School of Medicine, Chicago, IL, USA,^6^La Jolla Pharmaceutical Company, San Diego, CA, USA

**Introduction:** ATHOS-3 was a randomized, placebo-controlled, double-blind study of patients with severe vasodilatory shock (VS), which demonstrated that the addition of angiotensin II (Ang II) to standard vasopressors significantly increased mean arterial pressure (MAP) and decreased vasopressor utilization, with a trend towards improved survival to day 28. Given the location of angiotensin-converting enzyme in the pulmonary endothelium, we analyzed the effect of Ang II treatment in the subset of ATHOS-3 patients with acute respiratory distress syndrome (ARDS), in whom the pulmonary endothelium may be damaged.

**Methods:** Patients with persistent VS despite >0.2 μg/kg/min norepinephrine equivalent dose vasopressors were randomized to receive either IV Ang II or placebo. Patients with ARDS were classified based on the PaO2/FIO2 ratio, per the Berlin definition, as mild, moderate, and severe. Clinical outcomes, including MAP response were compared between groups.

**Results:** In the Ang II cohort, 34%, 30%, and 11% of the 163 patients were classified with mild, moderate, and severe ARDS, respectively as compared to 32%, 32% and 12% of the 158 patients in the placebo cohort. MAP response, defined as an increase from baseline of at least 10 mm Hg or an increase to at least 75 mm Hg, was achieved by 24%, 25%, and 16% of patients in the placebo group, for mild, moderate and severe ARDS respectively. In the Ang II group, MAP responses were achieved by 67% (OR=6.7, p<0.001), 69% (OR=6.6, p<0.001), and 61% (OR=8.4, p=0.005). In the placebo group, the 28-day mortality for mild, moderate and severe ARDS increased with severity, while the trend for worse survival was reduced in the Ang II cohort when compared to placebo for mild and severe ARDS (Table 1).

**Conclusions:** In patients with ARDS, MAP response was significantly improved in each ARDS subgroup, and the trend for increased 28-day mortality with an increase in ARDS severity was reduced in the Ang II group compared to the placebo group.

**Table 1 (abstract P125).** 28-day mortality % (95% Confidence Interval)

ARDS Category	Placebo	Angiotensin II
Mild	53 (40 - 67)	42 (30-56)
Moderate	55 (42 - 69)	55 (42-69)
Severe	74 (53 - 90)	50 (30-74)

## P126 Perioperative use of levosimendan: harmful or beneficious?

### F Guarracino^1^, S Bouchet^2^, M Heringlake^3^, P Pollesello^4^

#### ^1^Azienda Ospedaliero-Universitaria Pisana, Pisa, Italy,^2^University Hospital, Ghent, Ghent, Belgium,^3^Universitätsklinikum Schleswig-Holstein, Lübeck, Germany,^4^Orion Pharma R&D, Espoo, Finland

**Introduction:** Levosimendan is a calcium sensitizer and KATP-channel opener exerting sustained hemodynamic and symptomatic effects. In the past fifteen years, levosimendan has been used in clinical practice also to stabilize at-risk patients undergoing cardiac surgery. Recently, the three randomized, placebo-controlled, multicenter studies LICORN [1], CHEETAH [2] and LEVO-CTS [3] have been testing the peri-operative use of levosimendan in patients with compromised cardiac ventricular function. Over 40 smaller trials conducted in the past [4] suggested beneficial outcomes with levosimendan in peri-operative settings. In contrast, the latest three studies were neutral or inconclusive. We aim to understand the reasons for such dissimilarity.

**Methods:** We re-analyzed the results of the latest trials in the light of the previous literature to find sub-settings in which levosimendan can be demonstrated harmful or beneficious.

**Results:** None of the three latest studies raised any safety concern, which is consistent with the findings of the previous smaller studies. In LEVO-CTS, mortality was significantly lower in the levosimendan arm than in the placebo arm in the subgroup of isolated CABG patients (Fig. 1) [3]. The trend towards both hemodynamic and long term mortality benefits is maintained in recent meta-analyses [5,6] including the three larger recent studies.

**Conclusions:** Despite the fact that the null hypothesis could not be ruled out in the recent trials, we conclude that levosimendan can still be viewed as a safe and effective inodilator in cardiac surgery. Statistically significant mortality benefits seem to be limited to sub-groups, such as the isolated CABG procedures, and/or the low EF patients.

**References**

1. Cholley B et al. JAMA 318:548-556, 2017

2. Landoni G et al. N Engl J Med 376(21):2021-2031, 2017

3. Mehta RH et al. N Engl J Med 376(21):2032-2042, 2017

4. Harrison RH et al. J Cardiothorac Vasc Anesth 27:1224-1232, 2013

5. Sanfilippo F et al. Crit Care 21(1):252, 2017

6. Chen QH et al. Crit Care 21(1):253, 2017

**Fig. 1 (abstract P126).**
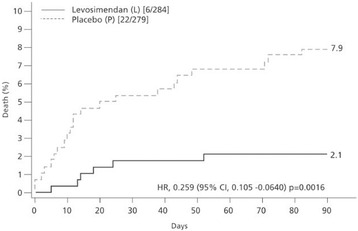
Ninety-day mortality among patients in the LEVO-CTS trial [3] in the subgroup of isolated CABG patients (n=563)

## P127 Effects of levosimendan on weaning from mechanical ventilation of patients with left ventricular dysfunction

### I Kaltsi, C Gratsiou, S Nanas, C Routsi

#### National and Kapodistrian University of Athens, Athens, Greece

**Introduction:** This study aims to assess the effects of levosimendan, a calcium sensitizer, in the treatment of patients with impaired left ventricular function and difficult weaning from mechanical ventilation.

**Methods:** Difficult- to- wean from mechanical ventilation [failed >= 3 consecutive spontaneous breathing trials (SBTs)] patients, who had left ventricular dysfunction defined as left ventricular ejection fraction (LVEF) of less than 40%, were studied.For each patient, 2 SBTs were studied: the first one before and the second one after a continuous infusion of levosimendan over 24 h (Control day and Study day, respectively). On both days transthoracic echocardiography (TTE) was performed on mechanical ventilation and at the end of the SBT. Also, serum levels of troponin I were measured at the same time points.

**Results:** Eleven patients (7 men; mean age of 72 ± 9 years) were studied. Whereas weaning failed in all patients on Control day, levosimendan administration enabled a successful spontaneous breathing trial in 8 out of 11 patients on Study day. Compared to the Control Day, left ventricular ejection fraction significantly increased on Study Day (from 27.5±9.6% to 36.9±2.8%, p< 0.05) whereas E/A significantly decreased both on mechanical ventilation and at the end of SBT: from 1.14±0.76 to 0.92±0.26, p<0.05 and from 1.29±0.73 to 0.88±0.12, respectively, p<0.05). Also, troponin I value decreased significantly on mechanical ventilation (from 217±268 to 163±194 mg/l) and at the end of SBT (from 196±228 to 146±180 mg/l). At the end of SBT, levosimendan treatment resulted in a lesser extent of arterial PO2 decrease: 96±35 vs. 66±27 mmHg, p<0.05) and similarly of ScvO2 decrease: 67±10 vs.60±8, p<0.05 (compared to the values on mechanical ventilation).

**Conclusions:** Levosimendan may provide significant benefit to difficult- to- wean patients with impaired left ventricular function.

## P128 Levosimendan for weaning veno-arterial ECMO (VA ECMO)

### G Haffner, G Ajob, M Cristinar, S Marguerite, W Oulehri, B Heger, M Kindo, PM Mertes, A Steib

#### Hôpitaux Universitaires, Strasbourg, France

**Introduction:** VA ECMO weaning is a challenging process. The aim of the study was to evaluate the putative benefit of levosimendan, for VA ECMO weaning.

**Methods:** This retrospective study, from 2014 to 2016, included patients referred to our ICU for primary cardiogenic shock or following cardiotomy, with VA ECMO in whom an attempt was made to wean mechanical support (death under VA ECMO or bridge to long-term device or transplantation were excluded). Incidence of weaning failure, VA ECMO support duration, length of stay in ICU and length of mechanical ventilation were compared in patients who received levosimendan or not in the whole population and in the post-cardiotomy sub-group. Levosimendan was used at doctor’s discretion. Independent factors associated with weaning failure were determined. Statistics were made throughout bayesian paradigm.

**Results:** 27 patients were included in levosimendan group and 36 in control group. In the whole population, weaning failure incidence and mortality was comparable between the 2 groups (respectively 24% vs 20%, Pr 0, 34 and 36% vs 38%, Pr=0,6). Higher assistance duration, longer stay under mechanical ventilation and longer duration of stay in critical care unit were observed in Levosimendan group. In the post-cardiotomy sub-group (Table 1), weaning failure was lower in levosimendan group (12% vs 29%, Pr 0,9) and levosimendan was an independent protective factor from weaning failure (OR 0,073, Pr 0,92). Positive impact of levosimendan may be explained in part by his calcium sensitizer effect and by facilitating recovery of myocardial calcium homeostasis in postcardiotomy cardiac stunning.

**Conclusions:** Levosimendan failed to reduce the incidence of ECMO weaning failure, except for post-cardiotomy population.

**Table 1 (abstract P128).**
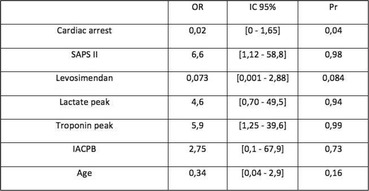
Multivariate analysis for weaning failure in post-cardiotomy sub-group (IACPB : Intra-Aortic ConterPulsion Balloon)

## P129 Renal outcomes of vasopressin and its analogues in distributive shock: a systematic review and meta-analysis of randomized trials

### T Rech, W Nedel, J Pellegrini, R Moraes

#### Hospital de Clínicas de Porto Alegre, Porto Alegre, Brazil

**Introduction:** Previous trials have suggested a lower incidence of the need for renal replacement therapy (RRT) and acute kidney injury (AKI) in patients with shock treated with vasopressin or its analogues (VA) compared to other vasopressors [1, 2]. The aim of the present study was to systematically review the literature and synthesize evidence concerning the effects of VA compared to other vasopressors in distributive shock, focusing on renal outcomes.

**Methods:** MEDLINE, Embase, Cochrane CENTRAL and Clinicaltrials.gov databases were searched through June, 2017 without language restrictions. Randomized clinical trials that compared VA with other vasopressors and reported renal outcomes in adult patients with distributive shock were included. Paired reviewers independently screened citations, conducted data extraction and assessed risk of bias. Odds ratio (OR) and weighted mean differences (WMD) with 95% confidence intervals (CI) were used to pool effect estimates from trials. Four prespecified subgroup analysis was conducted. Sensitivity analysis was applied to explore heterogeneity. The quality of evidence for intervention effects was summarized using GRADE methodology.

**Results:** 3,026 potentially relevant studies were identified and 30 papers were reviewed in full. Sixteen studies met the inclusion criteria, including a total of 2,054 individuals. Of these, 10 studies (1,840 individuals) were suitable for quantitative meta-analysis. Overall, the evidence was of low to moderate quality. Patients who received VA had a reduced need for RRT (OR 0.5 [0.33 - 0.75]; I2 = 7%, P for heterogeneity = 0.37) and a lower AKI incidence (OR 0.58 [0.37 - 0.92]; I2 = 63%, P for heterogeneity = 0.004).

**Conclusions:** In patients with distributive shock, VA use is associated with a reduced need for RRT and lower AKI incidence, although these results are supported by low quality evidence.

**References**

1. Hajjar LA et al. Anesthesiology 126:85-93, 2017

2. Russell JA et al. N Engl J Med 358:877-87, 2008

## P130 The effect of norepinephrine on venous return during VA-ECMO

### A Hana^1^, PW Moller^2^, PP Heinisch^1^, J Takala^1^, D Berger^1^

#### ^1^Inselspital, Bern University Hospital, Bern, Switzerland,^2^Institute of Clinical Sciences at the Sahlgrenska Academy, University of Gothenburg, Sahlgrenska University Hospital, Gothenburg, Sweden

**Introduction:** Venous return (VR) is driven by the difference between mean systemic filling pressure (MSFP) and right atrial pressure (RAP) and determines the maximum ECMO flow. MSFP depends on stressed volume and vascular compliance. It can be modified by absolute blood volume changes and shifts between stressed and unstressed volume. Norepinephrine (NE) may increase stressed volume by constriction of venous capacitance and at the same time increase the resistance to systemic flow. We therefore studied the effects of NE on MSFP, maximum ECMO flow and the ECMO pressure head (MAP-RAP).

**Methods:** MSFP was measured with blood volume at Euvolemia and NE 1 to 3 (0.05, 0.125 and 0.2μg/kg/h) in a closed-chest porcine VA-ECMO model (n=9, central cannulation with left atrial vent and av-shunt) in ventricular fibrillation. The responses of RAP and VR (measured as ECMO flow, QECMO) were studied at variable pump speeds including maximum possible speed without clinically apparent vessel collapse at constant airway pressure.

**Results:** The ECMO pump speed and QECMO showed a strictly linear relationship (r^2^ 0.95 to 0.995, range over all conditions) despite increased pressure head, indicating that the maximum QECMO was determined by VR alone. NE led to both increases in MSFP and QECMO in a dose dependent way, indicating a rightward shift in the VR plot (Fig. 1) via recruitment of stressed from unstressed volume (Table 1, Fig. 2). This resulted in an increased MSFP during NE despite decreased absolute blood volume (3.9±0.4 L vs. 3.3±0.3L, p=0.009). The reduced blood volume was associated with hemoconcentration suggesting plasma leakage.

**Conclusions:** NE shifts the VR curve to the right, allowing a higher maximum ECMO flow. The NE induced increase in MSFP results from recruitment of unstressed volume to stressed volume, which may be modified by changes in vascular compliance. The effects on pump afterload were not limiting.

**Table 1 (abstract P130).** Effects of NE on MSFP, QECMO, pressure head and hemoglobin as compared to euvolemia

	MSFP; mmHg	Max. QECMO; mL/min	Max. pressure head; mmHg	Hemoglobin; g/L
Euvolemia	7.1 ± 1.1	4376 ± 887	93 ± 27	94 ± 6
NE 1	6.7 ± 0.6	4369 ± 802	102 ± 21	100 ± 7
NE 2	7.4 ± 1.0	4670 ± 746	114 ± 36	106 ± 7
NE 3	7.9 ± 0.6	4967 ± 977	107 ± 37	108 ± 7
p value (RM ANOVA)	0.021	0.012	0.056	<0.001

**Fig. 1 (abstract P130).**
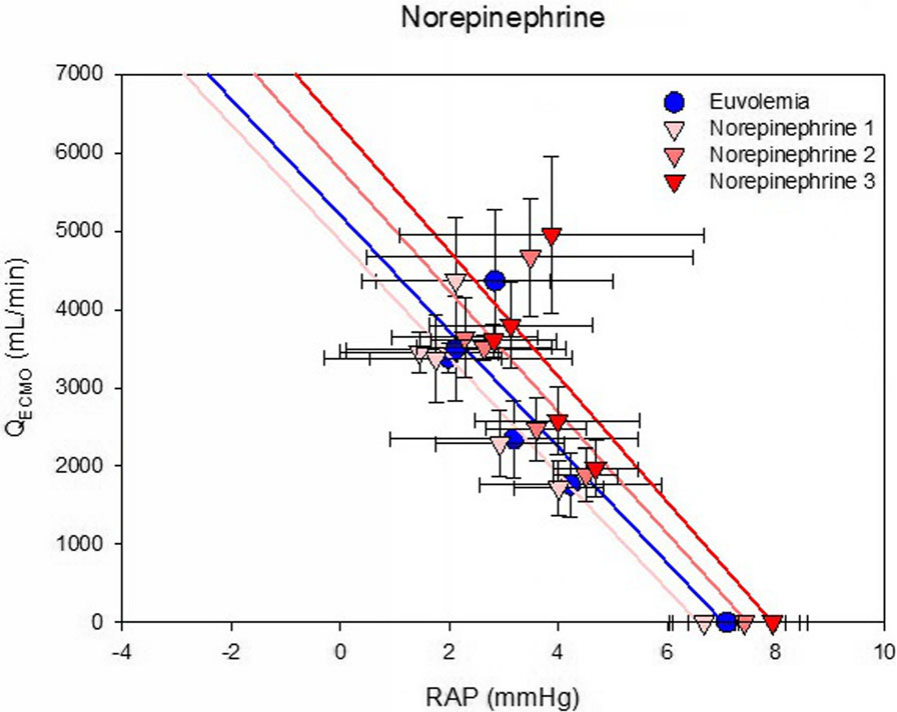
Effect of NE on VR curve as compared to euvolemia

**Fig. 2 (abstract P130).**
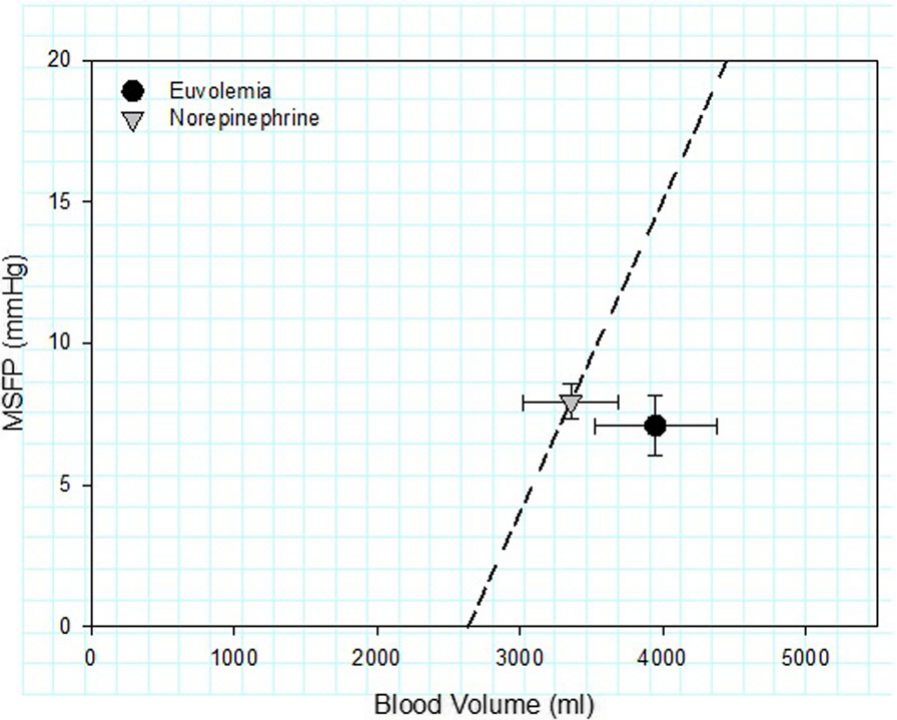
Effects of NE on blood volume, MSFP and vascular elastance

## P131 Predictive value of right heart hemodynamics for acute kidney injury after heart transplantation

### G Guven, M Brankovic, AA Constantinescu, JJ Brugts, DA Hesselink, S Akin, A Struijs, O Birim, C Ince, OC Manintveld, K Caliskan

#### Erasmus MC, University Medical Center Rotterdam, Rotterdam, Netherlands

**Introduction:** Acute kidney injury (AKI) is a major complication after heart transplantation (HTx), but the relation with the preoperative right heart hemodynamics (RHH) remain largely unknown. The aim of this study was to determine whether the routine and novel RHH could predict the development of AKI and 1-year patient survival in patients with HTx.

**Methods:** Data of all consecutive HTx patients (n=595) in our tertiary referral center, between 1984 and 2016, were collected and analyzed for the occurrence of AKI and survival at 1 year.

**Results:** AKI was developed in 430 (72%) patients; 278 (47%) stage-1, 66 (11%) stage-2, and 86 (14%) stage-3. Renal replacement therapy (RRT) was needed in 41 (7%) patients, with subsequent increased risk for chronic RRT-dependency at 1-year (odds ratio: 3.3 [95% CI: 1.6–6.6], p=0.001). Patients with higher AKI stages had also higher baseline right atrial pressure (RAP) (median: 7, 7, 8, 11 mmHg, p-trend=0.021), RAP/PCWP ratio (0.37, 0.36, 0.40, 0.47, p-trend=0.009), and lower pulmonary artery pulsatility index (PAPi) values (2.83, 3.17, 2.54, 2.31, p-trend=0.012). Patients with higher AKI stages had significantly lower survival at 1-year (5, 7, 15, 14 %, log-rank, p-trend=0.021) with worst outcome for RRT patients (1-year mortality with RRT vs no RRT: 22% vs 8%, log-rank, p=0.001).

**Conclusions:** AKI is highly frequent early after HTx and is inversely associated with 1-year patient survival. The routinely collected preoperative PAPi and RAP predict the development and severity of AKI early after HTx and could be used to timely intervene and prevent the development of AKI.

## P132 Haemorrhage versus sepsis in maternity HDU

### A Taylor, S Stevenson, J Gardner, K Lake, K Litchfield

### Princess Royal Maternity Unit, Glasgow Royal Infirmary, Glasgow, UK

**Introduction:** Haemorrhage and sepsis are the most common reasons for admission to our maternity HDU.

**Methods:** Data was gathered over a 26 month period from July 2015 to September 2017 looking at maternity HDU admissions with sepsis or haemorrhage. Using Excel length of stay, cardiovascular instability, monitoring, vasoactive drug use and baby with Mum was analysed. Local ethical approval granted.

**Results:** The total number of patients admitted was 514. 259 (50.4%) had a primary diagnosis of haemorrhage or sepsis. Haemorrhage was separated in to antepartum, 14 (9%) and postpartum 37 (90.7%). The most common reason for postpartum haemorrhage was uterine atony 62 (45.5%). 38 (35%) of sepsis admissions were antepartum and 70 (65%) postpartum. 1 (0.6%) was discharged to surgical HDU and 150 (99.3%) to ward. The most common source was gynaecological infection 29 (26%). 46 (42%) of admissions were from home; 26 (24%) antepartum and 20 (18%) postpartum. Most admissions 62 (57%) were via surgical or labour ward or post-theatre. 5 (3.9%) were discharged to medical or surgical HDU, 3 (2.7%) to ICU and 100 (92%) to ward. See Table 1.

**Conclusions:** Most admissions to HDU, 206 (79.5%) are in the postpartum period with haemorrhage accounting for the majority. These patients are more cardiovascularly unstable, require more invasive monitoring and blood pressure treatment but have a shorter overall stay. They are generally discharged straight to ward. This reflects the expected quicker improvement after haemostasis in well young women. The discrepancy between mum with baby (62% in sepsis cf 84% in bleeding) may reflect the higher likelihood of baby needing NICU in the sepsis group. Septic patients stay longer with higher risk of deterioration and escalation of care. This mirrors the recent MBRRACE [1] report stating sepsis as a major cause of morbidity.

**Reference**

1. Knight M et al. MBRRACE-UK Saving Lives, Improving Mothers’ Care 2016.

**Table 1 (abstract P132).** Cardiovascular instability and monitoring of maternity HDU patients with either sepsis or haemorrhage

	Haemorrhage	Sepsis	P value
Number of patients	151	108	
Mean length of stay (unit days)	0.91 Median 0.6 SD 0.88	1.38 Median 0.9 SD 1.29	
Arterial line	50 (33%)	23 (21%)	0.038
Central line	9 (5.9%)	8 (7%)	0.65
CVS instability	63 (41.7%)	21 (19.4%)	<0.01
Single vasoactive drug	9 (5.9%)	3 (2.7%)	0.23
Baby with mum if postpartum	115 (84%)	44 (62%)	<0.01

## P133 Real-time guidance or prelocation using ultrasound for pediatric central venous catheterization; a systematic review and network meta-analysis

### K Hosokawa, N Shime, M Kyo, Y Iwasaki, Y Kida

#### Hiroshima University Hospital, Hiroshima, Japan

**Introduction:** To locate vessels for percutaneous central venous catheterizations, it may be helpful to apply not only real-time ultrasound (US) guidance but also US-assistance vein prelocation. The aim of this study was to evaluate the superiority of two US methods compared to surface landmark methods by reviewing randomized control trials (RCTs).

**Methods:** As updating an earlier systematic review [1], we searched PubMed and CENTRAL in November 2017. We included RCTs which compared the failure rates of internal jugular or femoral venous cannulations among 1) real-time US guidance, 2) US-assistance vein prelocation and 3) surface landmark methods. A frequentist network meta-analysis was conducted using the netmeta package on R.

**Results:** Out of 1395 citations, 11 RCTs (935 patients) were eligible. The number of studies comparing outcomes between real-time US guidance vs. surface landmark methods, US-assistance vein prelocation vs surface landmark methods and real-time US guidance vs US-assistance vein prelocation was 7, 3 and 1. Regarding cannulation failure rate, network meta-analysis in a fix-effect model showed that a p-score was lower in the real-time US guidance than US-assistance vein prelocation (0.61 vs. 0.88), by reference to surface landmark methods, and also regarding arterial punctures, a p-score was lower in the real-time US guidance than US-assistance vein prelocation (0.64 vs. 0.83).

**Conclusions:** Based on the present network meta-analysis of RCTs, p-scores of cannulation failure and arterial puncture were lower in the real-time US guidance, suggesting that the US-assistance vein prelocation is superior than the real-time US guidance, both of which achieve lower rates of failure and arterial puncture compared to the landmark methods. We speculates that the inferiority of real-time guidance is associated with difficulties in manipulating the needle together with an echo probe in targeting relatively smaller veins in children.

**Reference**

1. Shime N et al. Pediatr Crit Care Med 16(8):718-25, 2015.

## P134 A beriberi bad heart - a case report

### M Charalambos, A Revill, D Howland

#### Torbay Hospital, Torquay, UK

**Introduction:** We present a case report of ‘Shoshin beriberi’ in a young female who was ‘fussy with food’ that developed an acutely progressive metabolic acidosis and multi-organ failure requiring intensive care support.

**Methods:** Our patient was a 36-year-old British woman who presented to the emergency department (ED) with a ten-day history of diarrhea, vomiting and increasing fatigue. She had a past medical history of gastroparesis, polycystic ovary syndrome (on metformin), laparoscopic cholecystectomy and hysteropexy. She lived with her husband and two children who had viral gastroenteritis two weeks previously.

**Results:** The patient had a metabolic acidosis (pH 6.9) with raised lactate (>16) on initial blood gas in the ED. A 1.26% sodium bicarbonate infusion and hemofiltration were commenced overnight. The patient’s pH and lactate remained static with an increasing work of breathing over this period. By morning she developed flash pulmonary oedema and hypotension, the first signs of acute cardiac failure. An echocardiogram displayed severely impaired left ventricular function with ejection fraction of 17%. The patient was intubated and inotropic support was commenced. It was thought that a micronutrient deficiency may have caused a rapid onset cardiac failure. Pabrinex (containing 250ml of Thiamine Hydrochloride) was commenced and within 9 hours the patient’s metabolic acidosis markedly improved (Fig. 1). Complete reversal of the cardiac failure occurred over 96 hours.

**Conclusions:** Shoshin is a rare clinical manifestation of thiamine deficiency [1]. It is an important differential diagnosis to bear in mind after excluding more common aetiologies of heart failure. Especially in this case as our patient had no obvious risk factors at the time of presentation. We suggest empiric use of thiamine should be considered in treatment algorithms for young patients presenting with acute cardiac failure. The pateint had provided informed consent for publication.

**Reference**

1. Naidoo DP, et al. Clinical diagnosis of cardiac beriberi. S Afr Med J 77(3):125-7. 1990.

**Fig. 1 (abstract P134).**
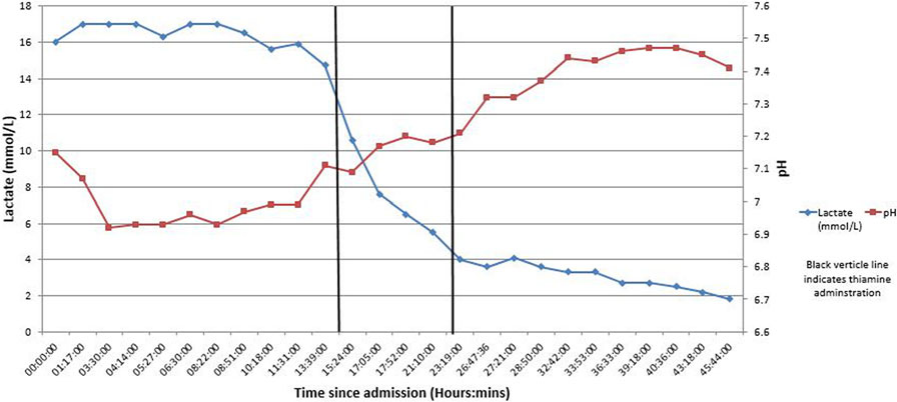
Serum lactate and pH with thiamine administration

## P135 Wells score is not a reliable predictor of the risk of pulmonary embolism in critically ill patients: a retrospective cohort study

### T Rech, A Girardi, R Bertiol, M Gazzana

#### Hospital de Clínicas de Porto Alegre, Porto Alegre, Brazil

**Introduction:** Pulmonary embolism (PE) is a commonly missed deadly diagnosis [1]. At the emergency department, the pretest probability of PE can be assessed using clinical prediction tools. However, critically ill patients are at a high risk for PE and prediction rules have not been validated in this population. The aim of the present study was to assess the Wells scoring system as a predictor of PE in critically ill patients.

**Methods:** Computed tomographic (CT) pulmonary angiographies performed for suspected PE in adult critically ill patients during their intensive care unit stay were identified by the radiology information system. Wells score was retrospectively calculated based on medical records and its reliability as a predictor of PE was determined using a receiver operator characteristic (ROC) curve.

**Results:** From the 144 patients evaluated, 39 (27%) were positive for PE based on CT pulmonary angiography. Mean Wells score was 3.9 ± 2.7 in patients with PE versus 2.4 ± 1.5 in patients without PE (P <0.001). Sixty patients (41.6%) were considered as low probability for PE (Wells score <2). From them, 13 patients (22%) presented with filling defects on CT scan, including two patients with main stain pulmonary artery embolism and one patient with lobar artery embolism. The area under ROC curve was 0.656. When a Wells score >4 was used to predict risk of PE, the sensitivity was 43%, specificity was 88%, PPV was 59% and NPV was 88.6%.

**Conclusions:** In this population of critically ill patients, Wells score was not a reliable predictor of risk of PE.

**Reference**

Wiener RS et al. BMJ 347: F3368, 2015

## P136 Acute kidney injury in cardiogenic shock syndrome: prevalence and outcome

### C Facciorusso, M Bottiroli, A Calini, D De Caria, S Nonini, R Pinciroli, F Milazzo, M Gagliardone

#### ASST Grande Ospedale Metropolitano Niguarda, Milan, Italy

**Introduction:** Cardiogenic shock (CS) is a syndrome due to acute heart failure. Acute kidney injury (AKI) could be secondary to tissue congestion and hypoperfusion. While many studies have evaluated the incidence of AKI in post-ischemic CS there is a paucity of data on non-ischemic CS etiology. The aim of this study is to evaluate clinical and prognostic relevance of AKI in this setting.

**Methods:** Monocentric, retrospective observational study on patients with CS. Study period: 2010-2016. Exclusion criteria: ischemic and postcardiotomy etiology of CS. Demographic, clinical and biochemical variables were collected at baseline and during hospital stay. AKI was defined according to AKIN criteria. Continuous variables are presented as median (IQR). Data were analyzed using comparative statistics and multivariate analysis was performed with Cox regression. Survival analysis was performed with Kaplan-Meier.

**Results:** We recruited 71 patients. Etiologies of CS were: 44 acute decompensation of chronic cardiomyopathies (CCM), 14 acute myocarditis, and 13 other causes. AKI occurred in 47 (66%) pts; among these AKIN stage 1, 2 and 3 occurred respectively in 16, 6 and 25 patients. In 14 pts a CRRT was required. Patients characteristics at baseline are summarized in Table 1 (* = p <0.05). Hospital mortality was 48% (n=23) in AKI pts vs 16% (n=4) in NON-AKI (p=0.01). 90 days cumulative survival was stratified for AKIN stages (Fig. 1, Log Rank < 0.003).AKIN stage3 and lactate level were independent predictors of death at 90 days: HR were respectively 3.1 (1.3-7.2) and 1.1 (1-1.2) per unit.

**Conclusions:** In patients with CS caused by non ischemic etiologies AKI is a frequent clinical complication associated with poor prognosis.

**Table 1 (abstract P136).** Baseline data

	NON AKI	AKI
AGE y	43 (29-62)	49 (37-63)
SOFA	8 (7-10)	11 (8-13)
MAP mmHg	66 (58-78)	67 (56-74)
LACTATE* mmol	2.1(0.8-4)	4.8(1.9-9)
INOTROPIC SCORE	10 (6-15)	18 (11-26)
CREATININE* mg/dl	0.8 (0.7-1.3)	1.9 (1.4-2.8)
LVEF %	20 (15-28)	20 (15-25)

**Fig. 1 (abstract P136).**
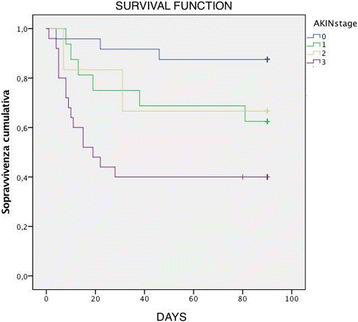
Kaplan-Meier 90 days survival

## P137 Takotsubo syndrome in critically ill patients

### A Shilova, M Gilyarov, E Gilyarova, A Nesterov, A Svet

#### Moscow City Hospital #1 n.a. N. Pirogoff, Moscow, Russia

**Introduction:** Takotsubo syndrome (TS) is known to be an acute transient cardiac condition accompanied with acute heart failure. TS is often triggered by critical illness but that has been rarely studied in ICU practice.Therefore, it is known, that the use of catecholamines can directly induce TS, worsen LVOT obstruction, and delay spontaneous recovery in TS patients, it is nearly impossible to avoid their administration in critically ill [1].

**Methods:** We have analyzed medical records from 23 patients with TS, that were revealed during year 2017 in our hospital. TS was defined due to Mayo criteria, including transient regional wall motion abnormalities, mildly elevated troponin level and no signs of obstructive CAD on coronary angiography.

**Results:** Out of 23 patients who developed TS in ICU or ICCU, hemodynamic instability occurred in acute phase of TS in 12 (52%) cases. 9 (39%) of patients were admitted to ICU in due to septic shock (2 patients), major bleeding (1), cerebral mass lesion (1) and ARDS (2) and required treatment with catecholamines. General mortality rate in TS patients was 7 (30%), and 5 (55%) in critically ill TS patients. Mean duration of noradrenalin infusion was 7,2 days, dobutamine infusion 4,3 days. Patients with TS needed more ICU resources and longer ICU-stay. Mortality rate was higher in TS patients (55%) vs the ICU-population (28%), p = 0.02.

**Conclusions:** TS seems to be an often cause of LV dysfunction and acute heart failure in critically ill. It seems that TS could be a predictor of worse prognosis in critically ill patients. Although catecholamine administration may worsen the patient prognosis and induce further AHF in critically ill patients it rearely can be avoided.

**Reference**

1. Templin C et al. N Engl J Med 373:929–938, 2015

## P138 Institutional case-volume and in-hospital mortality after orthotopic heart transplantation: a nationwide study between 2007 and 2016 in Korea

### S Choi^1^, E Jang^2^, K Nam^1^, G Kim^3^, H Ryu^1^

#### ^1^Seoul National University College of Medicine, Seoul, South Korea, ^2^Andong National University, Andong, South Korea, ^3^Kyungpook National University, Daegu, South Korea

**Introduction:** The positive effect of case volume on patient outcome seen in complex surgical procedures such as coronary artery bypass graft surgery has not been shown in heart transplantation (HT). The relationship between institutional case volume and patient outcome in adult HTs performed in Korea were analyzed

**Methods:** The Health Insurance Review and Assessment Service (HIRA) data from 2007 to 2016 was analyzed for in-hospital and long-term mortality, ICU length of stay, and hospital length of stay in patients undergoing HT, depending on the case volume of the institution.

**Results:** A total of 852 heart transplantation were performed between 2007 and 2016. The operative mortality after HTs was 8.6% (73/852). The operative mortality in institutions performing more than 20 cases/year was 3.5% (13/367) as compared to 8.0% (23/287) in institutions performing 10-19 cases/year and 18.7% (37/198) in institutions performing less than 10 cases/year. After adjusting for other potential factors for operative mortality, HT at intermediate volume centers 2.41 (95% CI 1.19–4.87, p=0.014) and low volume centers 6.74 (95% CI 3.41–13.31, p<0.001)were identified as risk factors of in-hospital mortality.

**Conclusions:** Our study results showed that HTs performed at institutions with higher case volume were associated with lower mortality.

## P139 Intensive care unit readmission following left ventricular assist device implantation: causes, associated factors, and association with patient mortality

### J Hui^1^, W Mauermann^1^, J Stulak^2^, A Hanson^3^, S Maltais^2^, D Barbara^1^

#### ^1^Department of Anesthesiology and Perioperative Medicine, Rochester, USA; ^2^Department of Cardiovascular Surgery, Rochester, USA; ^3^Department of Biostatistics, Rochester, USA

**Introduction:** Previous studies on readmission following LVAD implantation have focused on hospital readmission after dismissal from the index hospitalization. Since there are very little data existing, the purpose of this study was to examine intensive care unit (ICU) readmission in patients during their initial hospitalization for LVAD implantation to determine reasons for, factors associated with, and mortality following ICU readmission.

**Methods:** This was a retrospective, single center, cohort study in an academic tertiary referral center. All patients at our institution undergoing first time LVAD implantation from February 2007 to March 2015 were included. Patients dismissed from the ICU who then required ICU readmission prior to hospital dismissal were compared to those not requiring ICU readmission prior to hospital dismissal.

**Results:** Among 266 LVAD patients, 45 (16.9%) required ICU readmission. The most common reasons for admission were bleeding and respiratory failure (Fig. 1). Factors found to be significantly associated with ICU readmission were preoperative hemoglobin level of less than 10 g/dL, preoperative estimated glomerular filtration rate <35mL/min/1.73m2, preoperative atrial fibrillation, preoperative dialysis, longer cardiopulmonary bypass times, and higher intraoperative allogeneic blood transfusion requirements. Mortality at 1 year was 30.2% in patients requiring ICU readmission vs. 11.9% in those not requiring ICU readmission (age-adjusted OR=3.0, 95% CI 1.4 to 6.6, p=0.005).

**Conclusions:** ICU readmission following LVAD implantation occurred relatively frequently and was associated with significant one-year mortality. These data can be used to identify LVAD patients at risk for ICU readmission and implement practice changes to mitigate ICU readmission. Future larger and prospective studies are warranted.

**References**

Hasin T et al. J Am Coll Cardiol 61(2):153-63, 2013

Tsiouris A et al. J Heart Lung Transplant 33(10):1041-7, 2014

Forest SJ et al. Ann Thorac Surg 95(4):1276-81, 2013

**Fig. 1 (abstract P139).**
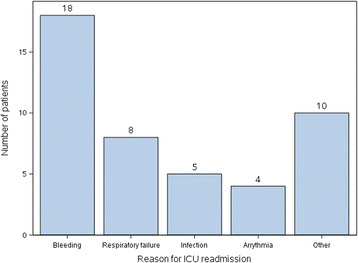
Reasons for ICU readmission during index hospitalization after LVAD placement. Of the 18 patients requiring ICU readmission for bleeding, 8 involved chest bleeding (e.g. hemothorax, cardiac tamponade), 7 gastrointestinal bleeding, 1 retroperitoneal bleeding, 1 bilateral subdural hemorrhage, and 1 tracheostomy site bleeding. Other reasons for ICU readmission included non-hemorrhagic cerebrovascular accident (2), LVAD malfunction (2), hyperactive psychosis (1), hyperkalemia (1), right ventricular failure (1), renal failure (1), syncope (1), and acute arterial thrombosis (1)

## P140 Atrial fibrillation and infection among acute patients in the emergency department: a multicentre cohort study of prevalence and prognosis

### T Graversgaard

#### Odense University Hospital, Odense, Denmark

**Introduction:** Patients with infection presenting with atrial fibrillation (AF) are frequent in emergency departments (ED). This combination is probably related to a poor prognosis compared to lone AF or infection, but existing data are scarce.

Aim: to describe the prevalence and prognosis for AF and infection individually and concomitantly in an ED setting.

**Methods:** Cohort study in adult (>=18 years) ED patients with ECG performed on presentation at Odense University Hospital and Hospital of South West Jutland, Denmark, from March 13 2013 to April 30 2014. AF was identified by electronic ECG records, and infection was identified based on discharge diagnoses. The absolute 30-day mortality and stroke rate were calculated for all patients, for those with AF, infection and for those with both.

**Results:** Among 39393 contacts to the ED, 27879 patients (median age 66, 50 % women) had an ECG recorded and were included in the study. 2341 (8.4%) had AF, 5672 (20.3%) had an infection and 670 (2.4%) had both infection and AF, of which 230 (34.3%) had no previous AF diagnosis or AF identified by ECG in the past 10 years (new-onset AF). In these groups, 30-day mortality was 11.3% in patients with infection, 10.4% in patients with AF and 22.6% in patients with new-onset AF and infection. One-year stroke rate in patients with AF was 61.7/1000 person-years (95% CI, 49.6 to 76.7), 21.2/1000 person-years (95% CI, 17.2 to 26.2) in patients with infection and 62.5/1000 person-years (95% CI, 39.1 to 120.2) in patients with new-onset AF and infection. Among patients with new-onset AF and infection, 42.6% had registered further AF episodes within one year after discharge, compared to 36.4% in patients with new-onset AF without infection.

**Conclusions:** Compared to ED patients with lone AF or infection, patients with concomitant new-onset AF and infection show an increased 30-day mortality, one-year stroke rate, and increased risk of further AF episodes.

## P141 Effect of positive end expiratory pressure on left ventricular contractility

### M Gruebler, O Wigger, S Bloechlinger, D Berger

#### Inselspital, University Hospital Bern, Bern, Switzerland

**Introduction:** Its afterload reducing effects make PEEP the treatment of choice for cardiogenic pulmonary edema. Studies indicate that PEEP may lower coronary blood flow. Its effects on left ventricular contractility is unclear. Most of the surrogate measures for cardiac contractility are dependent on afterload and contractility assessment under PEEP may therefore be biased. We have investigated cardiac contractility under PEEP with the endsystolic pressure volume relationship (ESPVR) as a load-independent measure of contractility.

**Methods:** 23 patients scheduled for coronary angiography were ventilated with CPAP and a full face mask at three levels of PEEP (0, 5 and 10 cmH2O) in random order. Structural cardiac pathologies were excluded with echocardiography. At every PEEP level, left ventricular pressure volume loops (Millar conductance catheter with INCA System, Leycom, Netherlands) were obtained. The endsystolic elastance was derived from a PV-loop family under preload reduction with an Amplatzer sizing balloon in the inferior caval vein. All participants gave written informed consent. The study was approved by the Bernese ethics committee.

**Results:** 5 women and 18 men with an age 59±6 years were studied. Ejection fraction was 70±8 % at baseline. Mean ESPVR at PEEP levels of 0, 5 and 10 were 2.64±1.3, 2.56± 1.18 and 2.33±0.88 mmHg/mL (p = 0.318, repeated measurements ANOVA). dP/dt and ejection fraction did not differ between the PEEP levels (p=0.138 and 0.48).

**Conclusions:** Moderate levels of PEEP did not influence endsystolic elastance. Higher PEEP and patients in cardiogenic shock should be investigated.

## P142 Biventricular 3D volumetric analysis with transthoracic echocardiography in the critically ill

### S Orde^1^, M Slama^2^, N Stanley^3^, SJ Huang^4^, AS Mclean^4^

#### ^1^ICU, Sydney, NSW, Australia; ^2^Amiens University Hospital, Amiens, France; ^3^Midland Hospital, Perth, Australia; ^4^Nepean Hospital, Sydney, Australia

**Introduction:** We sought to assess the feasibility of 3D volumetric analysis with transthoracic echocardiography in critically ill patients. We choose a cohort typical of ICU where accurate volumetric analysis is important: hypoxic, mechanically ventilated patients. 3D analysis is enticing in simplicity and wealth of data available. It is accurate in cardiology patients [1] but has not been assessed in the ICU.

**Methods:** Patients were imaged within 24 hours of admission. Inclusion criteria: adult, hypoxic (P:F <300), mechanically ventilated, Doppler stroke volume (SV) assessment possible. Echocardiography: Seimens SC2000 real-time volumetric analysis with standard B-mode and Doppler assessment. Images unacceptable if >2 segments unable to be seen in 2 volumetric planes. 3D Left ventricle (LV) and right ventricle (RV) analysis with Tomtec Imaging and Seimens Acuson respectively and compared to Doppler derived SV. 30% limit of agreement considered clinically acceptable [2]. Imaging was optimised for volumetric analysis (20-45 vols/sec).

**Results:** 92 patients, 83 in sinus, 9 in AF. No significant difference seen between Doppler vs 2D Simpson’s biplane, 3D LV or 3D RV SV estimation. Feasibility, SV values and bias are reported in Table 1 and Fig. 1. Limit of agreement for corrected Doppler vs LV 3D SV = -48% to 55%; RV 3D SV = -62.7% to 84.3%.

**Conclusions:** 3D LV and RV volumetric analysis is feasible in majority of patients requiring mechanical ventilation, however lacks agreement with Doppler derived stroke volume assessment. Although images may appear sufficient, the semi-automated software appears to underestimate stroke volume. Further larger studies using thermodilution are warranted.

**References**

1. Pedrosa J et al. Curr Pharm Des 22:105-21, 2016

2. Critchley L et al. J Clin Monit Comput 15:85-91, 1999

**Table 1 (abstract P142).** Doppler vs 2D and 3D stroke volume assessment

Parameter	Feasibility, n(%)	Stroke volume, Median (IQR)	Bias, mean difference (ml)	95% Confidence Interval
Doppler	92 (100%)	54 (43-64)	-	-
2D Simpson’s biplane	78 (85%)	52.2 (40-65)	-0.17	-2.9 to 2.5
3D LV	66 (72%)	49.5 (35-59)	-2.6	-5.5 to 0.2
3D RV	51 (55%)	43 (33-58)	-4.1	-9 to 0.9

**Fig. 1 (abstract P142).**
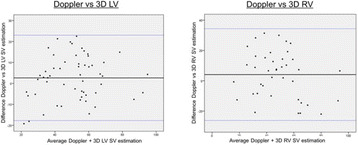
Bland-Altman plots for Doppler vs 3D biventricular stroke volume assessment

## P143 Determinants of venous return during trendelenburg position and effect of hepatic vascular waterfall

### S Liu^1^, P Moller^2^, A Kohler^1^, G Beldi^1^, D Obrist^3^, A Hana^1^, D Berger^1^, J Takala^1^, S Jakob^1^

#### ^1^Inselspital, Bern University Hospital, University of Bern, Bern, Switzerland; ^2^Institute of Clinical Sciences at the Sahlgrenska Academy, University of Gothenburg, Sahlgrenska University Hospital, Gothenburg, Sweden; ^3^University of Bern, Bern, Switzerland

**Introduction:** Body position changes such as leg raising are used to determine fluid responsiveness. We hypothesized that the Trendelenburg position increases resistance to venous return. Together with abolishment of the hepatic vascular waterfall, this may limit the increase in regional blood flow.

**Methods:** Inferior vena cava (IVC), portal vein (PV), hepatic, superior mesenteric (SMA) and carotid artery blood flows and arterial, right atrial (RA) and hepatic (HV) and portal venous blood pressures were measured in anesthetized and mechanically ventilated pigs in supine and 30° Trendelenburg positions. All hemodynamic parameters were measured during end-expiration at 5 cmH2O PEEP, and at inspiratory hold with increasing airway pressures (AWP) of 15, 20, 25 and 30 cmH2O, respectively. Paired t test was used to compare pressures and flows in different positions during end-expiration. Repeated measures ANOVA was performed to evaluate the effects of AWP on hemodynamic parameters.

**Results:** Trendelenburg position significantly increased RA, HV and PV blood pressures at end-expiration, while Qpv and Qsma remained unchanged, Qha increased and Qivc showed a trend to decrease (Table 1). In both positions, all blood flows decreased with increasing AWP, and the difference between Ppv and Qsma became smaller, indicating splanchnic blood pooling (Table 2). In the Trendelenburg position, splanchnic blood pooling was less severe compared to supine position.

**Conclusions:** Trendelenburg position tended to decrease venous return from inferior vena cava. Further increases in RAP by augmenting AWP led to a decrease in all flows and signs of abolished hepatic vascular waterfall. Passive manoeuvers to assess fluid responsiveness evoke complex hemodynamic reactions which are not fully understood.

**Table 1 (abstract P143).** Paired t test was used to evaluate the differences between groups

Parameters	Supine (n=13)	Trendelenburg (n=13)	P value (Paired t test)
RAP mmHg	7.8 ± 2.5	10.4 ± 3.3	<0.001
HVP mmHg	9.0 ± 2.2	11.4 ± 3.2	<0.001
PVP mmHg	10.3 ± 1.8	12.8 ± 2.7	<0.002
Qpv L/min	1.14 ± 0.17	1.12 ± 0.24	0.661
Qha L/min	0.13 ± 0.07	0.16 ± 0.08	0.007
Qivc L/min	1.91 ± 0.43	1.75 ± 0.54	0.055
Qsma L/min	0.83 ± 0.25	0.81 ± 0.22	0.636

One pig was excluded because no Trendelenburg was performed. All pressures and hepatic arterial flow increased in the Trendelenburg position. Values presented as Mean ± SD. RAP: right atrial pressure; HVP: hepatic venous pressure; PVP: portal venous pressure; Qpv: portal venous flow; Qha: hepatic arterial flow; Qivc: inferior vena cava flow; Qsma: superior mensenteric arterial flow

**Table 2 (abstract P143).** Repeated measures ANOVA was used to assess the effects of AWP and body position

Parameters	Body position	Expiration	Inspiration 30	AWP effect/interaction of AWP and group
Qpv	Supine (n=12) / Trendelenburg (n=12)	1.19 ± 0.13 / 1.14 ± 0.24	0.75 ± 0.11 / 0.89 ± 0.19	0.000 / 0.002
Qsma	Supine (n=12) / Trendelenburg (n=12)	0.83 ± 0.25 / 0.81 ± 0.23	0.56 ± 0.16 / 0.65 ± 0.20	0.000 / 0.203
Qha	Supine (n=12) / Trendelenburg (n=12)	0.13 ± 0.07 / 0.15 ± 0.09	0.07 ± 0.03 / 0.09 ± 0.04	0.001 / 0.849
Qivc	Supine (n=12) / Trendelenburg (n=12)	2.01 ± 0.38 / 1.78 ± 0.56	1.22 ± 0.25 / 1.33 ± 0.38	0.000 / 0.414
Qca	Supine (n=12) / Trendelenburg (n=12)	0.22 ± 0.07 / 0.25 ± 0.09	0.14 ± 0.04 / 0.17 ± 0.06	0.000 / 0.362
Qpv-Qsma	Supine (n=12) / Trendelenburg (n=12)	0.36 ± 0.33 / 0.31 ± 0.35	0.18 ± 0.23 / 0.23 ± 0.29	0.000 / 0.043

Two pigs were excluded from each group due to incomplete inspiratory hold maneuvers. AWP significantly affected all flows. Interactions with groups were found in Qpv and Qpv-Qsma

## P144 Link between bioelectrical impedance analysis derived phase angle and late mortality in cardiac surgery patients

### D Ringaitiene, D Grazulyte, D Zokaityte, L Puodziukaite, V Vicka, J Sipylaite

#### Department of Anesthesiology and Intensive Care, Institute of Clinical Medicine, Faculty of Medicine, Vilnius University, Vilnius, Lithuania

**Introduction:** Increasing age and frailty of patients undergoing cardiac surgery complicate the selection of patients. The aim of this study was to determine whether bioelectrical impedance analysis (BIA) phase angle is linked to long-term results after cardiac surgery and could be used as predictor.

**Methods:** This observational retrospective study included all of the patients who underwent any of the STS defined elective cardiac surgery type from 2013 to 2014 at the Vilnius University Hospital. Patients who died in the hospital during the first post-operative month were excluded. BIA was performed prior surgery, demographic and comorbidity data were gathered in perioperative period. We evaluated 3-5 year all-cause mortality rate. Patients were categorized according to the BIA provided phase angle (PhA) value, which was standardized for age and gender; long-term predictors were determined by Cox regression analysis.

**Results:** Among the cohort of 642 patients undergoing cardiac surgery, the median age was 67.8 [59 - 73] years; most of them were men (67.8%). Long term mortality rate was 12.3% (n=79). Most of the cases were low risk with median EuroSCORE II value of 1.78 [1.07 - 2.49]. The rates of standardized PhA were as follows: <5th 10.4% (n=67), <10th 17.3% (n=111), <15th 22.7% (n=146), <20th 27.6% (n=177), <25th 35.5% (n=228), <30th 42.2 % (n=271), <35th 47.4 % (n=304), <40th 52% (n=334), <45th 58.6% (n=386). The Cox regression analysis of all percentiles revealed the most potent predictor – phase angle value below 25th of the reference range (OR 2.42, 95% CI: 1.49-3.94, p<0.001), with a mean difference in survival of 13.22 months (64.60 vs 51.38 p<0.001). This relation persisted after adjustment with EuroSCORE II value.

**Conclusions:** BIA provided phase angle value can be used for long-term survival estimation before cardiac surgery. However, further studies are needed to prove the independent effect of these assumptions.

## P145 Screening ultrasound compression testing in critically ill patients performed by general nurses - validation study

### R Skulec^1^, A Kohlova^2^, L Miksova^1^, J Benes^1^, V Cerny^1^

#### ^1^Masaryk Hospital Usti nad Labem, JEP University, Usti nad Labem, Czech Republic; ^2^J.E. Purkinje University, Usti nad Labem, Czech Republic

**Introduction:** Despite of preventive measures, the incidence of deep venous thrombosis (DVT) in ICU patients is estimated to range from 5-31%. While clinical diagnostics is unreliable, ultrasound compression test (UCT) has proven to be a highly sensitive and specific modality for the recognition of lower extremity DVT [1]. Delegating this competence to ICU nurses can increase UCT availability and enable preventive DVT screening. Therefore, we decided to conduct a clinical study to evaluate the sensitivity and specificity of UCT performed by general ICU nurse in ICU patients compared to an investigation by ICU physician certified in ultrasound.

**Methods:** Prior to the study, each nurse participating in the study completed one-hour training in UCT and examined 5 patients under supervision. Then, ICU patients without known DVT underwent UCT in the femoral and popliteal region of both lower extremities performed by trained general ICU nurse. On the same day, the examination was repeated by an ICU physician. The results of the examinations of each patient were blinded to each other for both investigators until both tests were performed. In case of a positive test, the nurse immediately reported the result to the ICU physician. The sensitivity and specificity of the test performed by general nurse was calculated in comparison with the examination by a specialist.

**Results:** A total of 80 patients were examined. Both lower extremities were examined in all patients. The prevalence of DVT of 11,25% has been found. The overall sensitivity of the examination performed by general nurse was 90.0%, the specificity 100% with negative predictive value of 98.61%, positive predictive value of 100% and accuracy of 98.77%.

**Conclusions:** The results of our study have shown that general ICU nurses are able to perform bedside screening of DVT by compression ultrasound test with a high degree of reliability after a brief training.

**Reference**

1. Minet C et al. Crit Care 19:287, 2015.

## P146 Effect of cytoadsorbant device on coagulation factors during cardio-pulmonary bypass

### E Poli, L Alberio, A Bauer-Doerries, C Marcucci, A Roumy, M Kirsch, E De Stefano, C Gerschheimer, L Liaudet, A Schneider

#### Centre Hospitalier Universitaire Vaudois (CHUV), Lausanne, Switzerland

**Introduction:** Cytokine Hemoadsorption (HA) might improve outcomes of patients undergoing cardiac surgery with cardiopulmonary bypass (CPB). However, the effect of HA on coagulation factors remains unknown. This substudy nested within a randomized control trial comparing HA device with standard of care (NCT02775123) aims at evaluating the effect of a cytoadsobent device on coagulation factor activity.

**Methods:** A Cytosorb® (Cytosorbents, New Jersey, USA) HA device was inserted within the CPB circuit in ten patients undergoing elective cardiac surgery. One hour after CPB onset, the activity of coagulation factors (Antithrombin (AT), von Willebrand Factor (vWF), factors II, V, VIII, IX, XI, and XII) were measured before and after the device. Pre and post device measurements were compared using student t-test, a p value <0.05 was considered statistically significant.

**Results:** Patients’ mean age was 60.6 ± 21.4 years, 20% were female, the mean EuroSCORE II was 6.2 ± 8.1. Procedures were: coronary artery bypass graft (CABG) (2/10), aortic root replacement (6/10) and CABG combined with aortic valve replacement (2/10). Mean CPB duration was 161.8 ± 52.3 min. Pre and post HA measurements of coagulation factors activity are presented in Fig. 1. Post-device AT and FII activity was significantly lower (respectively from 70.4 to 66.6, p=0.01 and from 61.5 to 57.1, p=0.03) compared to pre-device measurement. There was no statistically significant difference between pre-and post- HA measurements for all other coagulation parameters

**Conclusions:** Pre and post HA Cytosorb® measurements for coagulation factor activity were not different except for a small decrease in AT and FII activity. This might be related with intra-device consumption or adsorption. Further analyses accounting for CPB fluid balance, the entire study population and timepoints are pending.

**Fig. 1 (abstract P146).**
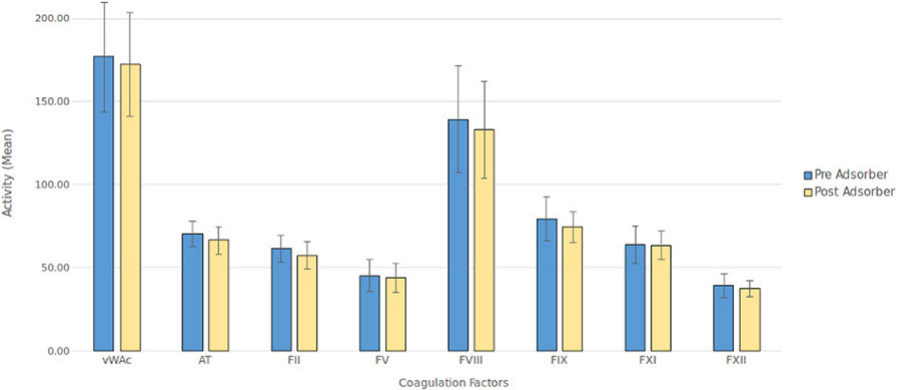
Comparison of mean activity levels of coagulation parameter factors pre and post device. Error bars correspond to standard deviation

## P147 Changes in microvascular perfusion during blood purification with cytosorb in septic shock

### S Zuccari, V Monaldi, S Vannicola, R Castagnani, A Damia Paciarini, A Donati

#### Università Politecnica delle Marche, Ancona, Italy

**Introduction:** The aim of this study is to evaluate changes in hemodynamics and microvascular perfusion during extracorporeal blood purification with Cytosorb in patients with septic shock requiring renal replacement therapy.

**Methods:** Eight adult patients with septic shock requiring continuous renal replacement therapy for acute renal failure were enrolled and underwent a 24-hour treatment with the emodasorption cartridge Cytosorb. Measurements were taken at baseline before starting Cytosorb, after 6h (t1) and 24h (t2) and included: blood gases, macro-hemodynamic parameters (Picco2), vasopressor and inotropic dose, plasma levels of cytokines (interleukin [IL]-1, IL6, IL8, IL10, tumor necrosis factor alpha) and parameters of microvascular density and perfusion (sublingual sidestream dark field videomicroscopy). Procalcitonin was measured at baseline and after 24h of treatment.

**Results:** A non-significant decrease in plasma levels of cytokines was observed over time. Hemodynamic parameters and vasopressor requirement remained stable. The microvascular flow index increased significantly at t2, total vessel density and perfused vessel density increased at t1 and t2 (Figs. 1 and 2).

**Conclusions:** In patients with septic shock requiring continuous renal replacement therapy for acute renal failure, blood purification with Cytosorb was associated with an improvement in sublingual microvascular perfusion.

**Fig. 1 (abstract P147).**
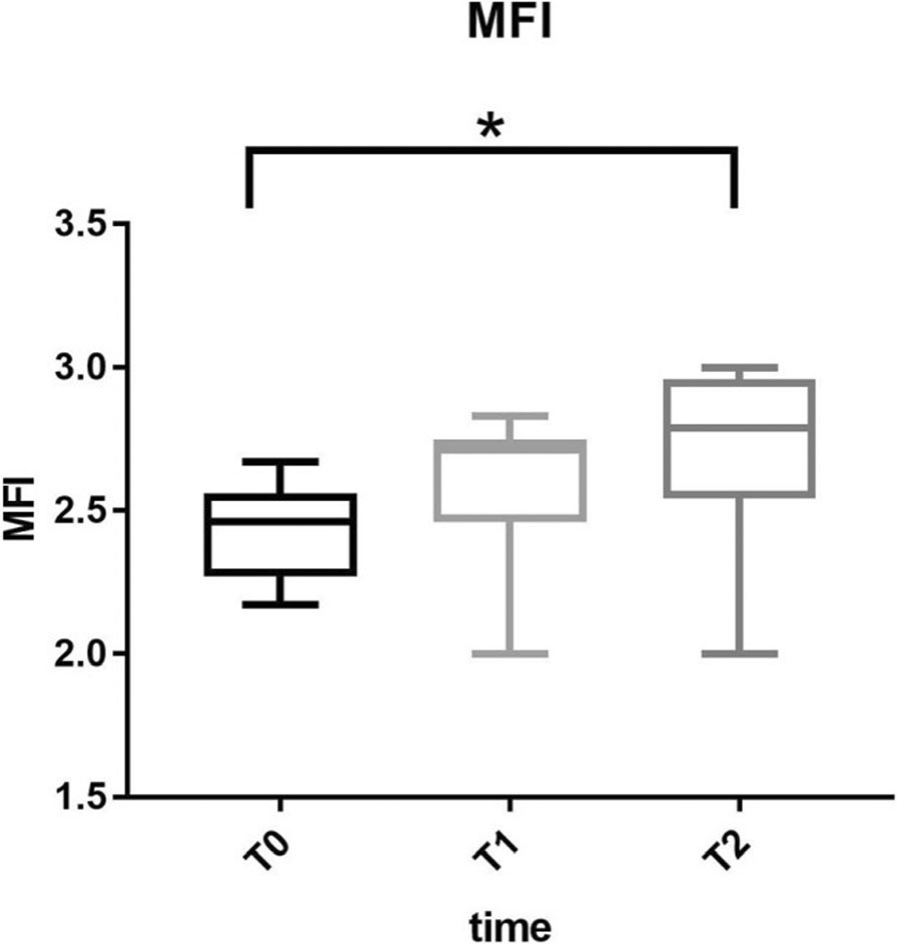
Microvascular Flow Index

**Fig. 2 (abstract P147).**
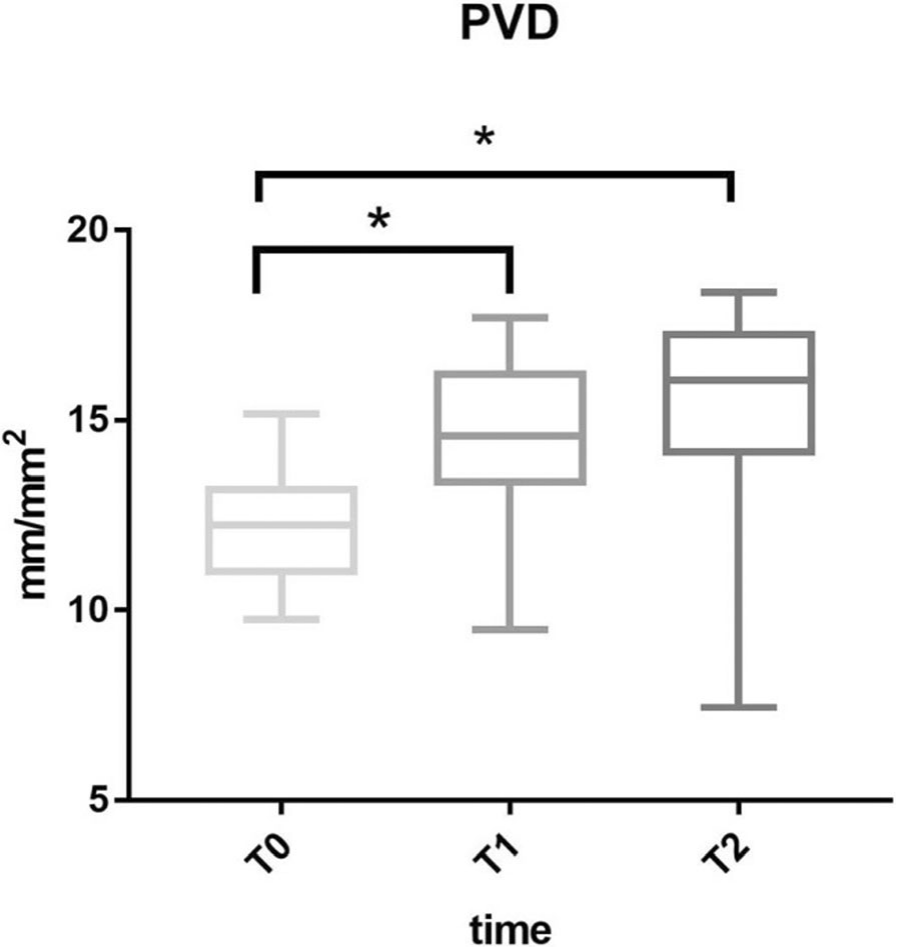
Perfused Vessel Density

## P148 Renal replacement therapy with the OXIRIS filter decreases inflammatory mediators and improves cardiorenal function in septic patients better then CVHDF.A cohort study and a propensity–matched analysis

### F Turani ^1^, S Busatti^1^, S Martini^1^, M Falco^2^, F Gargano^2^, R Barchetta^2^, L Weltert^2^, F Leonardis^3^

#### ^1^Aurelia Hospital, Rome, Italy; ^2^European Hospital, Rome, Italy; ^3^University of Tor Vergata, Rome, Italy

**Introduction:** Objective renal replacement therapy (RRT) with the OXIRIS filter is used in sepsis septic shock with AKI, but few clinical studies compare the adsorbing effect of Oxiris filter on the inflammatory mediators to RRT.

The aim of this study is 1- to confirm whether oxiris decreases cytokines and procalcitonin in sepsis septic shock.2- This effect is superior to RRT.3- This translates in a better cardio renal response.

**Methods:** A coohort study and a propensity–matched analysis included 73 patients admitted to three Intensive Care (Aurelia Hospital, European Hospital, Tor Vergata – Rome) with a diagnosis of septic shock.50 patients were submitted to RRT with oxiris filter and 23 patients to RRT.Il 6, Procalcitonin, the cardiorenal indices and SOFA score were compared before (T0) and at the end of the treatments (T1). All data are expressed as mean±sd. ANOVA one way was used to compare the changes of the variables in the time. P< 0.05 was considered statistically significant.

**Results:** Of 50 patients submitted to RRT with the oXiris filter 32 could be matched to 22 septic patients who received RRT. IL6 and Procalcitonin decreased in the Oxiris group (p< 0.01) but not in the RRT group.MAP increased (p< 0.01) and noradrenaline dosage decreased in oxiris group (p< 0.01), but non in RRT group. Also PaO2/FIO2 ratio, diuresis, SOFA improved only in the in the oxiris group (p<0.05).

**Conclusions:** In sepsis/septic shock patients with AKI, IL6 and procalcitonin decrease more in the oXirs group then in the RRT group.This is associated with an improvement of the cardio - renal function and the clinical condition.The study confirms that RRT with oXiris filter may be useful in sepsis/septic shock when other convective/diffusive techinques fail.

**Reference**

1. Journal of Hepatology 2015 vol. 63 j 634 ̈C642

## P149 ADVOS reduces liver and kidney disease markers and corrects acidosis: the Hamburg experience

### VH Fuhrmann, D Jarczak, O Boenisch, S Kluge

#### University Medical Center Hamburg-Eppendorf, Hamburg, Germany

**Introduction:** ADVOS (Hepa Wash GmbH, Munich, Germany) is a recently developed CE-certified albumin-based hemodialysis procedure for the treatment of critically ill patients. In addition to the removal of water-soluble and albumin-bound substances, acid-base imbalances can be corrected thanks to an automatically regulated dialysate pH ranging 7.2 to 9.5.

**Methods:** Patients treated with the ADVOS procedure between in the Department of Intensive Care Medicine of the University Medical Center Hamburg-Eppendorf were retrospectively analyzed. Overall 102 treatments in 34 critically ill patients (Mean SOFA Score 16) were evaluated. Additionally, subgroup analysis for hyperbilirubinemia, respiratory acidosis and non-respiratory acidosis were conducted.

**Results:** Severe hyperbilirubinemia (>6 mg/dl) was present in 60 treatments, while 26 and 14 treatments were performed to treat respiratory (PaCO2>45 mmHg) and non-respiratory (PaCO2<45 mmHg) acidosis (pH<7.35), respectively. Mean treatment duration was 16 h.

ADVOS procedure was able to correct acidosis and reduce bilirubin, BUN and creatinine levels significantly. The subgroup analysis shows an average bilirubin reduction of 21% per ADVOS multi treatment in the hyperbilirubinemia group (15.24mg/dL vs 11.77mg/dL, p<0.05). Moreover, pH (7.23 vs. 7.35, p<0.001) and PaCO2 (65.88 vs. 53.61 mmHg, p<0.001) were corrected in the respiratory acidosis group, while in the non-respiratory acidosis group, an improvement in pH (7.19 vs. 7.37, p<0.001), HCO3 (15.21 vs. 20.48, p=0.002) and base excess (-12.69 vs. -5.10, p=0.004) could be observed.

There were no treatment-related adverse events during therapy.

**Conclusions:** ADVOS is a safe and effective hemodialysis procedure, which is able to remove water soluble and protein bound markers and correct severe acidosis in critically ill patients.

## P150 Score for timely prescribing (STOP) renal replacement therapy in intensive care unit - preliminary study of a mneumonic approach

### E Leal Bastos de Moura, A Alves de Sousa, F Ferreira Amorim, J Rodrigues Trindade Jr, M De Oliveira Maia

#### Hospital Santa Luzia Rede D’Or São Luiz, Brasília, Brazil

**Introduction:** The moment of initiation of renal replacement therapy (RRT) in critically ill patients and a reason for debate, without having objective criteria that indicate it. The objective of this study was to propose a score to help identify the ideal time for the initiation of RRT, and if there is correlation between this score and Intensive Care Unit length of stay and mortality.

**Methods:** patients admitted to the Intensive Care Unit, > 18-years-old, to whom RRT were indicated by the intensivist. The study protocol was approved by the Hospital das Forças Armadas Ethical Committe, and written informed consent was obtained from all patients. The STOP was assigned according to the presence or not of each of the items (Fig. 1). They were classified into groups A and B according to Fig. 2, and the group change was recorded.

**Results:** 80 patients admitted to ICU in the period, 2 excluded for limitation of therapeutic efforts. 78 were admitted to the study, with the mean age of 75.2 years; 64,1% males (n=50). Distribution among the groups: A1 (n=1, 1.2%), A2 (31, 39.7%), A3 (5, 6.4%), B1 (6, 7.6%), B2 (35, 44.8%) e B3 (no patients). There were statistically significant correlation between group change and mortality (p 0.02), and between the STOP and nephrologist agreement (p 0.01). There was no correlation between STOP value and ICU LOS (p 0,75) or STOP and mortality (p 0.8).

**Conclusions:** The STOP value is correlated with hemodialysis indication agreement between intensivists and nephrologists, and not correlated with ICU LOS or mortality. The group change was correlated to increased mortality, in the study population. The significance of STOP as a tool in determining the moment of initiation of renal replacement therapy remains a work in progress.

**Fig. 1 (abstract P150).**
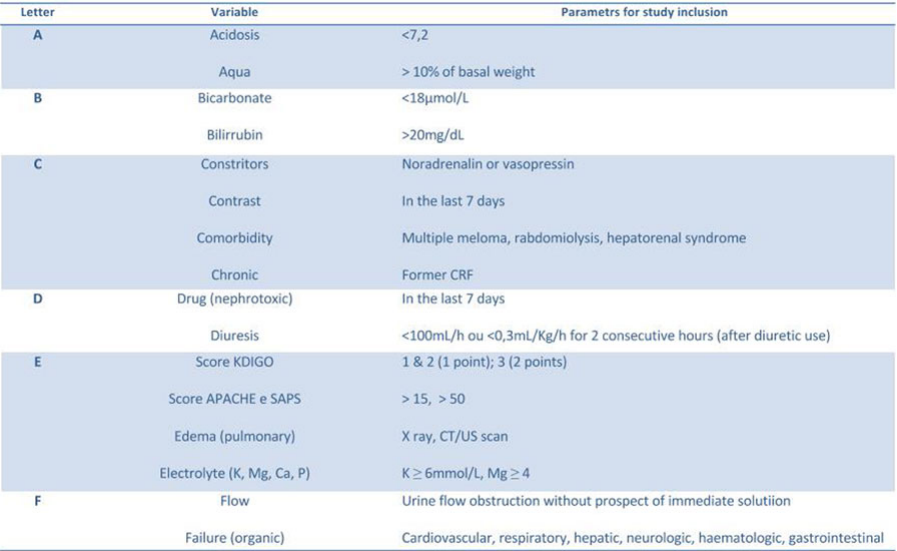
STOP items description

**Fig. 2 (abstract P150).**
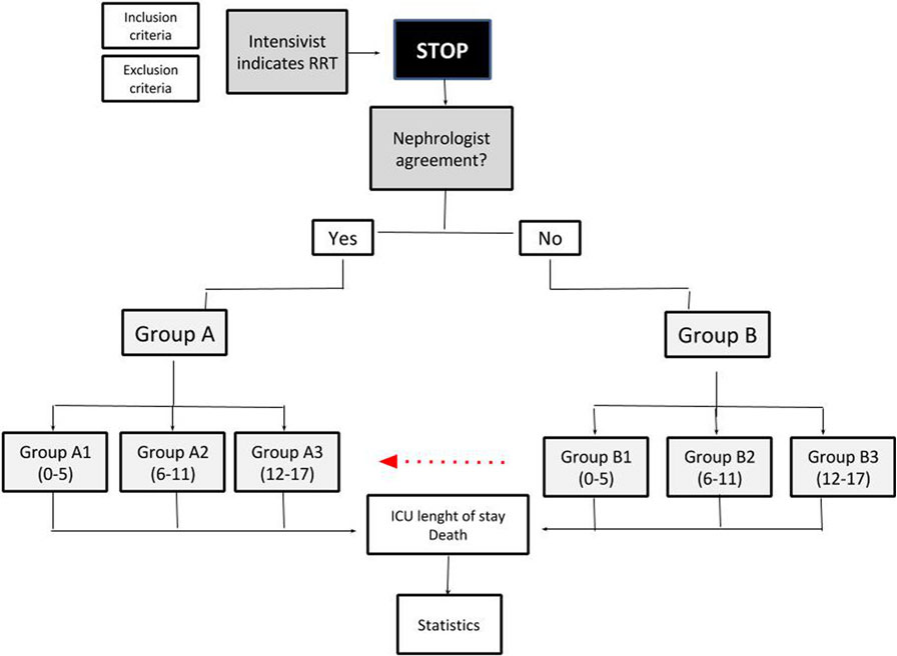
Description of the study

## P151 Intraoperative continuous renal replacement therapy in liver transplantation: a pilot randomized controlled trial (INCEPTION study)

### C Karvellas, S Taylor, T Ozelzel, E Bishop, D Cave, D Bigam, S Bagshaw

#### University of Alberta, Edmonton, Canada

**Introduction:** Liver transplant (LT) in patients with renal dysfunction presents intraoperative challenges and portends postoperative morbidity. Continuous renal replacement therapy (CRRT) is increasingly used for intraoperative support; however, there is a paucity of data to support this practice.

**Methods:** Pilot randomized open-label controlled trial in adults receiving cadaveric LT with a Modification of End-Stage Liver Disease (MELD) score >=25 and preoperative acute kidney injury (KDIGO stage 1) and/or estimated glomerular filtration rate <60 mL/min/1.73m2. Patients were randomized to intraoperative CRRT (iCRRT) or standard of care. Primary endpoints were feasibility and adverse events. Secondary endpoints were changes in intraoperative fluid balance, complications, and hospital mortality. Analysis was intention-to-treat.

**Results:** Sixty patients were enrolled, 32 (53%) were randomized (17 to iCRRT; 15 to control). Mean (SD) was age 49 (13) years, MELD was 36 (8), 75% (n=24) had cirrhosis; 63% (n=20) received preoperative RRT; and 66% (n=21) were transplanted from ICU. One patient allocated to iCRRT did not receive LT. Seven (41%) allocated to control crossed over intraoperatively iCRRT (high central venous pressure [n=4]; abdominal distension [n=1]; massive transfusion [n=1]; hyperkalemia [n=1]). No adverse events occurred. Operating time was similar (513 [140] vs. 463 [115] min, p=0.30). CRRT duration was 379 (137) min, with only 3 interruptions (all due to access). iCRRT fluid removal was 2.8L (range 0–14.5). Fluid balance was 5.3L (2.9) for iCRRT vs. 4.3L (6.1) for control (p=0.57). Postoperative CRRT was similar (77% vs. 50%, p=0.25). There were no differences in reexploration (p=0.36), mechanical ventilation time (p=0.87), reintubation (p=0.18), sepsis (p=0.56), or mortality (p=0.16).

**Conclusions:** In this pilot trial of high acuity LT patients, iCRRT was feasible and safe. These data will inform the design of a large trial to define the role of iCRRT during LT.

**References**

ClinicalTrials.gov: NCT01575015.

## P152 The uptake of citrate anticoagulation for continuous renal replacement therapy in intensive care units across the United Kingdom

### A Soni, M Borthwick

#### Oxford University Hospitals NHS Foundation Trust, Oxford, UK

**Introduction:** The purpose of this descriptive study is to report the trend of citrate anticoagulation uptake, used for continuous renal replacement therapy (CRRT), in intensive care units (ICUs) across the United Kingdom (UK). Citrate anticoagulation has been used in the UK since 2008, but its uptake since then is unknown [1].

**Methods:** A survey questionnaire targeted pharmacists working in UK adult ICUs providing CRRT. Invitations to participate were distributed utilising the United Kingdom Clinical Pharmacy Association online forum as a platform for access. Survey administration was by self-completion and submissions were accessible over a total of six weeks. Basic demographic data, ICU specifications, the citrate system in use and implementation details were sought. A descriptive statistical analysis ensued.

**Results:** 70 responses were received of which 67 were analysed after duplication removal. 45 trusts, encompassing a total 67 units, in the UK confirmed use of citrate anticoagulation for CRRT. Units reported a mean of 71 days to implement a citrate system (range 0 to 645 days). Prismaflex® (Baxter) and Multifiltrate (Fresenius) were reported as the most commonly used citrate systems; 32 (47.8%) and 28 (41.8%) units respectively.

**Conclusions:** There are 279 ICUs in the UK [2]. We conclude that a minimum of 67 units (24%) use citrate anticoagulation for CRRT in UK critical care centres. Citrate systems of anticoagulation are becoming an increasing popular choice for regional anticoagulation, falling in line with international guidance [3]. These guidelines were introduced in 2012 which corresponds to increase national uptake.

**References**

1. Borg R et al. Intensive Care Soc 18:184-192, 2017.

2. Borthwick M et al. Int Pharm Pract (in press), 2017.

3. Kidney Disease: Improving Global Outcomes Acute Kidney Injury Working Group. Kidney Int Suppl 2:1-138, 2012.

## P153 Is vasopressor/inotrope requirement effected by ultrafiltrate volume and 24 hour fluid balance in intensive care patients undergoing acute renal replacement therapy?

### A Lal^1^, M Shaw^2^, A Puxty^1^

#### ^1^Glasgow Royal Infirmary, Glasgow, UK; ^2^University of Glasgow, Glasgow, UK

**Introduction:** Patients requiring renal replacement therapy (RRT) whilst on significant doses of vasoactive medications have often been deemed unsuitable to undergo ultrafiltration (UF). However with better understanding of the pathophysiology of renal injury [1] in intensive care patients we hypothesise that vasopressor/inotrope requirement will not significantly increase with UF or with a more negative fluid balance (FB).

**Methods:** Data was retrospectively collected in a general ICU/HDU of adult patients requiring acute RRT for acute kidney injury. Patients on chronic dialysis were excluded. Percentage change in vasopressor index and mean arterial pressure were combined to form the Combined Percentage Change (CPC) which we used as an index of patient stability.

**Results:** 38 patients were assessed undergoing a total of 206 RRT sessions. The mean age was 57 with 23 females and 15 males. Mean FB for the 24 hours from start of RRT was +651mls (range -2317 to +14850mls). Using a model to correct for significant covariates and plotting 24 hour FB against CPC we found no significant effect of FB on stability p=0.98 (Fig. 1). Mean UF volume was 880mls (range 0-3009mls). There was a non linear relationship between UF and stability with moderate volumes improving but larger volumes worsening stability (Fig. 2). This did not reach statistical significance (p=0.074) so may be due to chance but is likely due to a lack of power.

**Conclusions:** Fluid balance has no effect on cardiovascular stability during RRT in our cohort but there may be a varying effect of UF depending on volume.

**Reference**

Perner A et al Intensive Care Med 43:807-815, 2017

**Fig. 1 (abstract P153).**
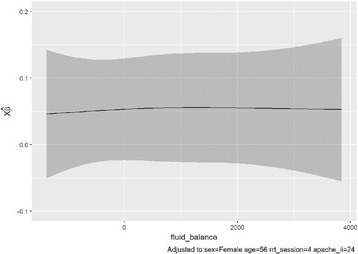
See text for description

**Fig. 2 (abstract P153).**
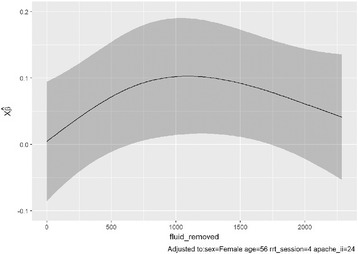
See text for description

## P154 Hemostatis parameter changes induced by filter change in infants on continuous renal replacement therapy

### A Akcan Arikan, PR Srivaths, N Tufan Pekkucuksen, JR Angelo, TM Mottes, MC Braun

#### Baylor College of Medicine, Houston, TX, USA

**Introduction:** Exposure of blood to a foreign surface such as a continuous renal replacement therapy (CRRT) filter could lead to activation of platelets (plt) and fibrinogen (fib) trapping. Thrombocytopenia has been reported in adults on CRRT but data in pediatrics are scarce. Our institution uses regional citrate anticoagulation (RCA) as standard of care with prefilter hemodilution and HF1000 filters (polysulfone, surface area (SA) 1.1 m2) regardless of patients’ (pts) age and size. As filter SA is relatively larger in younger pts, we aimed to investigate the impact of CRRT filter change on hemostasis parameters in infants on CRRT in up to first three filter changes.

**Methods:** Retrospective chart review

**Results:** 30 patients < 10 kg were included, age 4.3 (0.5-8) months, weight 5.4+2.4 kg, with 88 filters. Metabolic disease was the most common principal diagnosis (7/30, 23%), liver failure (LF) was the most common comorbidity (12/30, 40%). All patients received prefilter continuous venovenous hemodiafiltration with minimum dose of 2000 ml/1.73m2/h. Thrombocytopenia was common at CRRT start (28/30, 93%). Plts decreased in 74% filter changes (65/88) by 15+70% (pre vs post plt 71 (44-111) vs 50(30-83), p<0.001). Fibrinogen also decreased from 201 (152-261) to 170 (134-210), p<0.001; there was no change in PTT, PT, or INR values before and after filter changes. Bleeding events were seen in 13/30 (43%) of pts (8/12 of LF pts vs 5/18 others, p=0.04), but were not more common in pts who had decrease in plts or fib with filter changes (41% with drop in plts vs 57% without, p=0.66; 47% with drop in fib vs 75% without, p=0.58).

**Conclusions:** Thrombocytopenia is common in infants on CRRT. Further decreases in plt and fibrinogen can be seen in with CRRT filter changes if the filters are relatively large compared to patient size. Bleeding events seems more related to underlying comorbidity, and less to changes in hemostatis parameters observed with filter change but would need to be confirmed with further studies.

## P155 Intensive monitoring of post filter ionized calcium concentrations during CVVHD with regional citrate anticoagulation: is it still required?

### D Khadzhynov, F Halleck, O Staeck, L Lehner, T Slowinski

#### Charite Universitaetsmedizin, Berlin, Germany

**Introduction:** The aim of the present study was to evaluate the role of postfilter calcium concentrations (pfCa) in terms of safety and efficacy in large retrospective cohort of patients treated with CVVHD and regional citrate anticoagulation.

**Methods:** Retrospective, observational study at a university hospital with 6 ICUs. All patients treated with RCA-CRRT were included in the study.

**Results:** Among 1070 patients treated with RCA-CVVH pfCa at the start of the CVVHD was available in 987 pts. The pfCa concentrations were in target range (0.25-0.35 mmol/L) in the majority of patients (70%), whereas 17% and 13% of patients had the pfCa below or above the target range, respectively. In the further 72h of CVVHD treatment the propotion of patients with targeted pfCa increased to 86% and remained stable. At the start of the RCA-CVVHD there was a significant but weak correlation between the pfCa and ionized systemic Ca (iCa) with a Spearman rank-order correlation coefficient (rho) of 0.374 (p < 0.001). The coefficient of variation of pfCa concentraions was significantly higher if compared to the coefficient of variation of iCa concentration. Using per protocol adaptations the incidence of a severe hypocalcemia (<0.9 mmol/L) was low and present only at first 12 hours of therapy: 4% and 2% of patients with pfCa below the target range and 0.7% and 0.4% of patients with pfCa in target range, at 0h and 12h respectively (p<0.001). There was no correlation between pfCa concentrations and filter lifetime.

**Conclusions:** The results of the present study support the previous reports about higher measurements variation of pfCa compared to systemic iCa (1). Nevertheless due to the weak correlation of iCa and pfCa as well as a low number of patients with a severe metabolic complication, the results of our study question the necessity of intensive pfCa monitoring during RCA-CRRT. Present results need to be validated in further trials.

**Reference**

1. Schwarzer P. et al, Crit Care. 2015 Sep 8;19:321

## P156

**Withdrawn**

## P157 Association of pain in the critically ill patient with acute kidney injury risk in the intensive care unit (ICU)

### JM Vieira Junior, LB Herranz, LC Pontes de Azevedo, I Castro

#### Hospital Sírio Libanês, Sao Paulo, Brazil

**Introduction:** In critically ill patients, occurrence of pain is frequent and usually correlates with worse outcomes, such as prolonged ICU length of stay (LOS) and mechanical ventilation. In this regard, pain leads to sympathetic activation, inflammatory mediators and therefore, potentially to organic dysfunction. The aim of this study is to evaluate the relationship between acute pain in critically ill patients and their association with acute kidney injury (AKI).

**Methods:** Retrospective cohort with 6345 adults patients admitted between June 2013 and June 2016, from the ICU of Hospital Sírio Libanês Hospital in Sao Paulo (Brazil). Main exclusion criteria were: length of stay < 48h, coma and previous AKI. The predictor pain was obtained through daily electronic records according to numerical verbal scale (0-10). The outcome was defined as serum creatinine elevation equal to or greater than 0.3mg/dl and/or greater than 50% increase at any time after the first 48 hours in the ICU. The multivariate analysis was performed by Binary Logistic Regression through distinct groups of early or late predictive factors in relation to AKI.

**Results:** After the exclusion of 3220 patients, the incidence of pain with numerical verbal scale equal to or greater than 3 points was 23.6%. The outcome occurred in 31.7% of the cohort. In the binary regression, using the more early predictive factors, sex and pain presented independent relation with the outcome - adjusted OR 1.24 (1.12-1.36) and 1.63 (1.34-1.98), respectively (p <0.001). In the analysis of late association factors, mechanical ventilation over 3 days - OR 4.71 (3.01-7.36), use of strong opioid - OR 2.7 (1.58- 4.60) and PCR- t over 5.2mg/dl - OR 2.27 (1.15-4.47) presented the highest positive association with AKI (p<0,001).

**Conclusions:** Poor management of ICU pain is associated to worse outcomes, including increased risk to AKI. The search for a better pain management strategy in the ICU scenario should therefore be reinforced.

## P158 Incidence and outcomes of acute kidney injury in 3 large inner city hospitals

### S Channon^1^, B Girling^1^, B Trivedi^2^, S DeFreitas^2^, C Kirwan^2^, J Prowle^1^

#### ^1^Queen Mary University, London, UK; ^2^Barts Health NHS Trust, London, UK

**Introduction:** Acute Kidney Injury (AKI) is a common complication in hospitalised patients, strongly associated with adverse outcomes [1]. A lack of baseline incidence and outcome data limits our ability to assess local strategies aimed at improving AKI care.

**Methods:** In an audit in three linked inner London hospitals we interrogated our electronic patient data warehouse (Cerner Millennium power insight electronic data warehouse) with a specially written query to identify cases of AKI, defined by KDIGO creatinine criteria, in patients aged over 18y admitted for >24h during January to June 2016. We excluded palliative care and obstetric patients. In the absence of premorbid baseline (median 7-365d pre-admission) the admission creatinine value was used. End stage renal disease (ESRD) and primary sepsis diagnosis was obtained from ICD10 coding.

**Results:** Of 28872 admissions, we excluded 1052 with pre-existing ESRD (Hospital mortality 6.0%) and 8833 with fewer than one creatinine result who could not be assigned AKI status (mortality 1.1%). Of the remaining 18987 there were 3145 with AKI (16.6%), with mortality increasing from No AKI group (2.4%), to AKI stage 1 (12.6%), and a further increase to AKI stages 2-3 (22.4%) (p<0.001) (Table 1). Patients with AKI were older (p<0.001), more likely to be medical than surgical (p<0.001), more likely to have a primary sepsis diagnosis (p<0.001) and had higher baseline creatinine (median 91 vs 79 p<0.001). No known baseline was found in 29.7% of patients with AKI, but their mortality did not significantly differ to those with a baseline (14.2% vs 16.6%, p=0.093).

**Conclusions:** An electronic query identified the local burden of AKI and it’s associated hospital-mortality; such baseline data is essential to assess the effect of Quality Improvement interventions in AKI prevention and care.

**Reference**

1. Coca SG et al. Am J Kidney Dis 53(6):961-73, 2009

**Table 1 (abstract P158).** Incidence and associations of AKI

	All	All AKI	No AKI	AKI-1	AKI-2	AKI-3	p AKI vs No AKI
Number	18987	3145	15842	2087	566	492	-
Mortality (%)	4.6	15.9	2.4	12.6	20.8	24.2	p<0.001
Age (median)	65	70	63	70	69	69	p<0.001
Medical (%)	65.1	69.1	64.3	68.3	69.8	71.5	p<0.001
Sepsis (%)	7.2	8.7	6.9	9.0	7.8	8.3	p<0.001

## P159 Loop diuretics to treat acute kidney injury in critically ill patients: a systematic review and meta-analysis

### K Rosas^1^, D Gutierrez^2^

#### ^1^Hospital Espanol de Mexico, Ciudad de Mexico, Mexico; ^2^Hospital Angeles Acoxpa, Ciudad de Mexico, Mexico

**Introduction:** Acute kidney injury (AKI) is a common condition in critically ill patients [1, 2]. Loop diuretics are generally used as first line treatment. However, controlled trials show controversial results. We ought to search systematically and realize a meta-analysis on the matter.

**Methods:** An electronic search of randomized clinical trials in adult patient treated with diuretics for AKI compared with standard treatment or a control group was conducted. The primary objective of the analysis was to assess recovery of renal function. Secondary endpoints included time to recovery of renal function, need for Renal Replacement Therapy (RRT), mortality in the Intensive Care Unit (ICU) and complications.

**Results:** The search obtained 7 studies for the analysis. A total of 853 patients, 446 in the intervention group and 407 in the control group were included. Comparing those treated with diuretic vs control, the analysis showed relative risk (RR) 1.11 for renal recovery (95% CI [0.74 - 1.67], p = 0.62), RR 1.29 for recovery time (95% CI [- 3.30 - 0.72], p = 0.21), the need for TRR with RR 0.96, (95% CI [1.23 - 0.75], p = 0.74) and mortality in the ICU with RR 0.80 (95% CI [0.48 - 1.31], p = 0. 52) (Fig. 1). The intervention group had an increased risk of complications compared to control (RR 1.83, 95% CI [1.40 - 2.40], p < 0.0001).

**Conclusions:** The use of loop diuretic to treat AKI showed no difference in the recovery of renal function, the need for RRT or mortality in the ICU. However, it exhibited higher risk of complications.

**References**

1. Hoste EAJ et al. Intensive Care Med 41(8):1411-1423, 2015

2. Hoste AJ et al. Crit Care 10(3):R73, 2006

**Fig. 1 (abstract P159).**
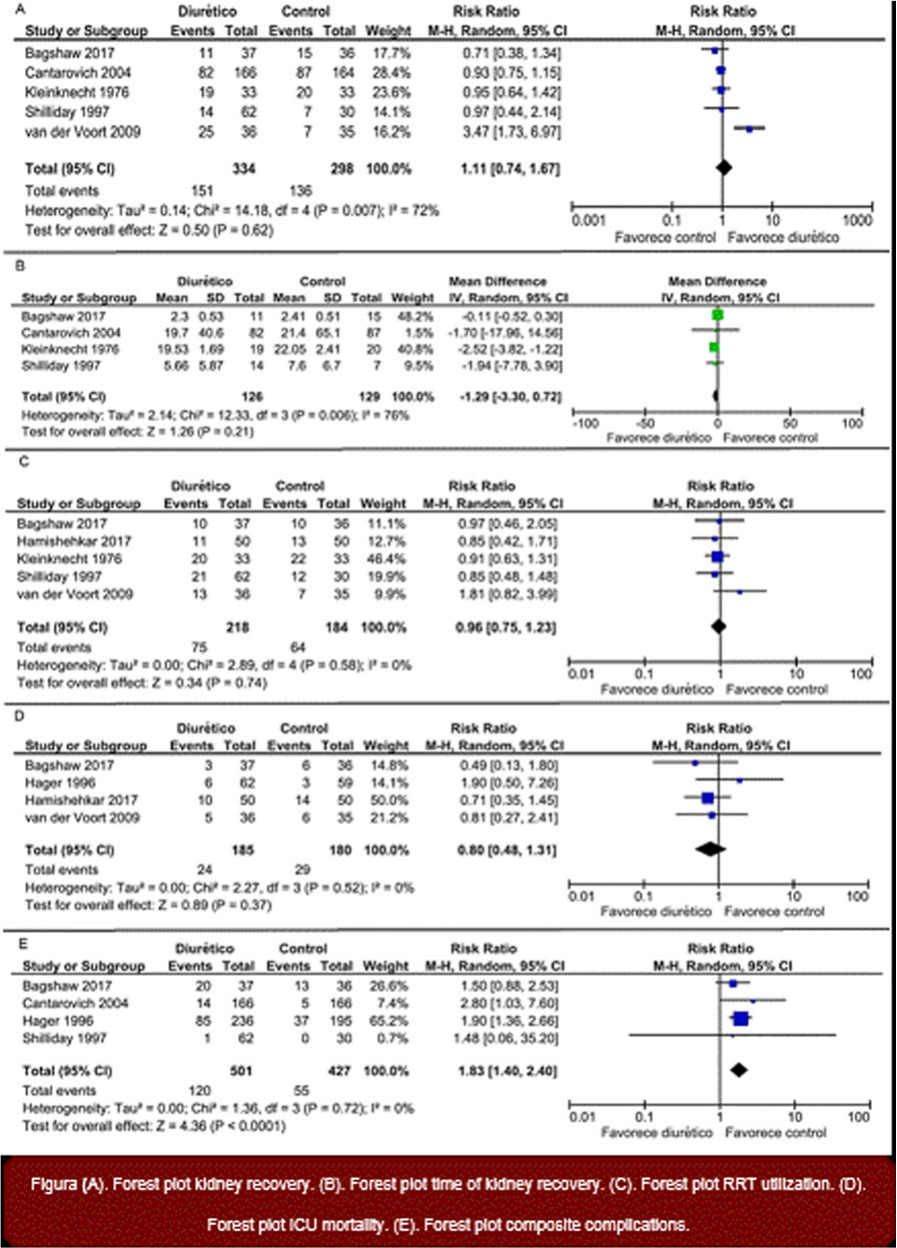
Forest plot

## P160 Alterations in portal vein flow and intra-renal venous flow are associated with acute kidney injury after cardiac surgery: a prospective cohort study

### W Beaubien-Souligny^1^, A Benkreira^1^, P Robillard^1^, Y Lamarche^1^, J Bouchard^2^, A Denault^1^

#### ^1^Montreal Heart Institute, Montréal, Canada; ^2^Hôpital Sacré-Coeur, Montréal, Canada

**Introduction:** Increased venous pressure is one of the mechanism leading to acute kidney injury (AKI) after cardiac surgery. Portal flow pulsatility and discontinuous intra-renal venous flow are potential ultrasound markers of the impact of venous hypertension on organs. The main objective of this study was to describe these signs after cardiac surgery and to determine if they are associated with AKI.

**Methods:** This single center prospective cohort study (NCT02831907) recruited adult patients able to give consent. Ultrasound studies were performed before cardiac surgery and repeated on post-operative day (POD) 0, 1, 2 and 3. Abnormal portal and renal venous flow patterns are defined in Fig. 1. The association between the studied markers and the risk of new onset of AKI in the following 24 hours period following an assessment was tested using logistic regression with a 95% confidence interval. Clinical variables associated with the detection of the signs were tested using generalized estimating equation models. This study was approved by the local ethics committee.

**Results:** During the study period, 145 patients were included. The presence of the studied ultrasound signs is presented in Fig. 2. During the week following cardiac surgery, 49 patients (33.8%) developed AKI, most often on POD 1 (71.4%). The detection of portal flow pulsatility and severe alterations in renal venous flow (Pattern 3) at ICU admission (POD 0) were associated with AKI in the subsequent 24 hours period and was independently associated with AKI in multivariable models including EUROSCORE II and baseline creatinine (Table 1). The variables associated with the detection of abnormal portal and renal patterns were associated with lower perfusion pressure, higher NT-pro-BNP and inferior vena cava measurements (Table 2).

**Conclusions:** Abnormal portal and intra-renal venous patterns are associated with early AKI after cardiac surgery. These Doppler features must be further studied as potential treatment targets to personalize management.

**Table 1 (abstract P160).** Assessment of echographic parameters on post-operative day 0 and the risk of AKI in the subsequent 24 hours period

Echographic markers	N (%) of AKI	OR (CI) p-value	Adjusted OR* (CI) p-value
Normal portal flow (PF<50%)	23/122 (18.9%)	1.0	1.0
Portal pulsatility (PF>=50%)	12/21 (57.1%)	5.7 (2.2-15.2)<0.01	3.7 (1.3-10.5) 0.02
Renal pattern 1	17/97 (17.5%)	1.0	1.0
Renal pattern 2	6/23 (26.1%)	1.7 (0.6-4.8) 0.35	1.4 (0.5-4.3) 0.54
Renal pattern 3	11/18 (61.1%)	7.4 (2.5-21.8)<0.01	4.8 (1.5-15.8) 0.01

*Multivariable model included baseline creatinine and EUROSCORE II

**Table 2 (abstract P160).** Association between the studied echographic markers and clinical parameters after surgery

	Portal flow pulsatility (PF>50%)	Renal pattern 2	Renal pattern 3
	OR (CI) p	OR (CI) p	OR (CI) p
NT-pro-BNP (per 1 log increase)	10.7 (4.1-27.4) <0.01	6.9 (2.8-17.1) <0.01	10.4 (3.9-27.9) <0.01
Inferior vena cava diameter (per cm increase)	1.8 (1.1-2.9) 0.02	2.1 (1.4-3.2) <0.01	2.0 (1.4-2.9) <0.01
Perfusion pressure (DBP-CVP) (per 10 mmHg)	0.6 (0.4-0.9) 0.01	0.7 (0.5-1.0) <0.02	0.5 (0.3-0.8) <0.02
Cumulative fluid balance (per 1L increase)	1.3 (1.1-1.5) <0.01	1.1 (0.9-1.2) 0.34	1.1 (0.9-1.3) 0.34

**Fig. 1 (abstract P160).**
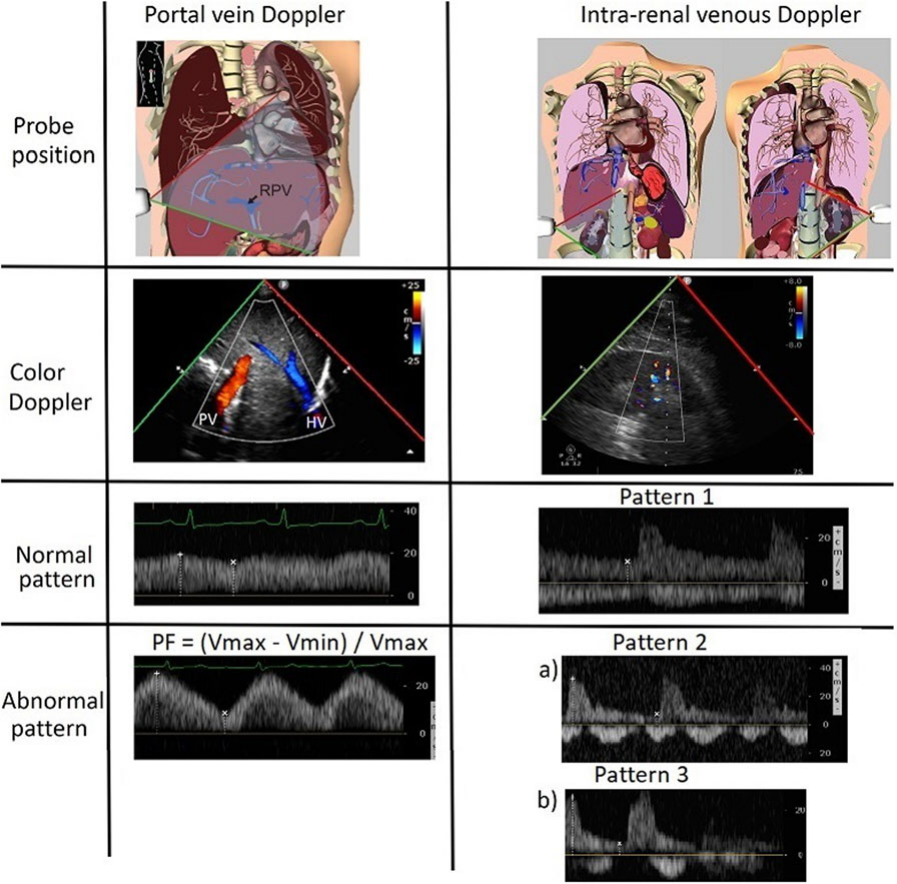
Portal vein and intra-renal venous Doppler assessment using trans-thoracic ultrasound. Probe position in showed using the Vimedix simulator (CAE Healthcare, St-Laurent, Canada). Abnormal pattern of intra-renal venous flow can be separated into a) Pattern 2 defined as discontinuous with flow in both systole and diastole and b) Pattern 3 defined as discontinuous with flow only in diastole.

**Fig. 2 (abstract P160).**
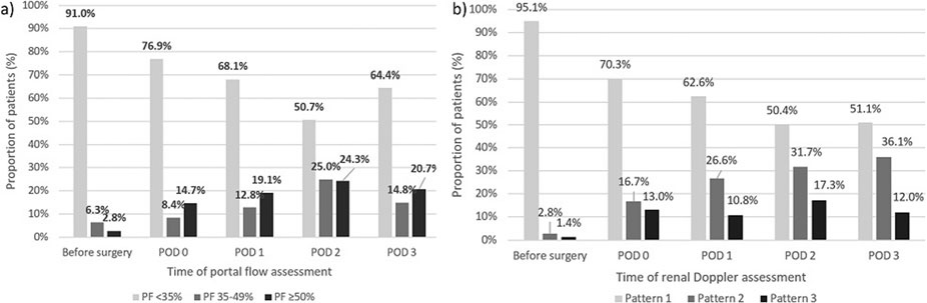
Portal and intra-renal venous flow patterns during the peri-operative period. (PF: pulsatility fraction, POD: post-operative day)

## P161 A clinical prediction model for severe AKI after pediatric cardiac surgery

### I Scharlaeken^1^, M Flechet^2^, D Vlasselaers^1^, L Desmet^1^, G Van den Berghe^1^, F Güiza^2^, G Meyfroidt^1^

#### ^1^University Hospitals Leuven, Leuven, Belgium; ^2^KU Leuven, Leuven, Belgium

**Introduction:** Acute kidney injury (AKI) is very prevalent after cardiac surgery in children, and associated with poor outcomes [1]. The present study is a preplanned sub-analysis of a prospective blinded observational study on the clinical value of the Foresight near-infrared spectroscopy (NIRS) monitor [2]. The purpose of this sub-analysis was to develop a clinical prediction model for severe AKI (sAKI) in the first week of PICU stay.

**Methods:** sAKI was defined as serum creatinine (SCr) >/= 2 times the baseline, or urine output < 0.5 ml/kg/h for >/= 12h. Predictive models were built using multivariable logistic regression. Data collected during surgery, upon PICU admission, as well as monitoring and lab data until 6h before sAKI onset, were used as predictors. Relevant predictors with a univariate association with sAKI, were included in the models. Accuracy of the models was tested using bootstraps, by AUROC and decision curves.

**Results:** 177 children were enrolled, admitted to the PICU of the Leuven University Hospitals after cardiac surgery, between October 2012 and November 2015. 5 patients were excluded. 70 children (40.7%) developed sAKI in the first week of PICU stay. A multivariate model with 5 admission parameters (maximum lactate during surgery, duration of CPB, baseline sCr, RACHS1 and PIM2 scores), and 4 postoperative measurements (average heart rate, average blood pressure, hemoglobin, lactate), was most predictive for sAKI (Fig. 1).

**Conclusions:** The risk of sAKI in children after congenital cardiac surgery could be predicted with high accuracy. Future models will also include medication data. These models will be compared against and combined with NIRS oximetry data to investigate the independent and added predictive value of the Foresight monitor.

**References**

1. Kaddourah A et al. N Engl J Med 376:11-20, 2017

2. ClinicalTrials.gov Identifier: NCT01706497

**Fig. 1 (abstract P161).**
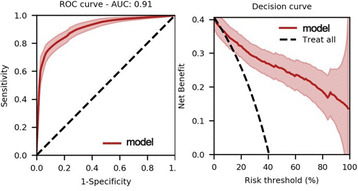
Performance of the multivariate LR model for sAKI

## P162 Renal perfusion, function and oxygenation in early clinical septic shock

### J Skytte Larsson, V Krumbholz, A Enskog, G Bragadottir, B Redfors, S Ricksten

#### Institution for clinical sciences, Göteborg, Sweden

**Introduction:** Acute kidney injury (AKI) occurs in over 50% of the patients in the intensive care unit (ICU). The predominantly ethiology of AKI is septic shock, the most common diagnosis in the ICU. AKI significantly increases the risk of both morbidity and mortality[1].

**Methods:** 8 ICU patients with septic shock was studied within 24 hrs from admission. 58 patients after cardiac surgery served as control group. All patients were sedated and mechanically ventilated. Renal blood flow (RBF) and glomerular filtration rate (GFR) were obtained by the infusion clearance of paraaminohippuric acid (PAH) and by extraction of 51Cr-ethylenediamine (51Cr-EDTA). N-acetyl-β -D-glucosaminidase (NAG), was measured.

**Results:** RBF was 19% lower, renal vascular resistance 19% higher and the relation of RBF to cardiac index was 29% lower in patients with septic shock compared to the control group. GFR (32%, p=0.006) and renal oxygen delivery (RDO2) (24%) where both significantly lower in the study group (Table 1). There was no difference between the groups in renal oxygen consumption (RVO2) but Renal oxygen delivery was almost 30% lower in septic shock patients. Renal oxygen extraction was significantly higher in the study group than in the control group. In the study group, NAG was 5.4 ± 3.4 units/mikromol creatinine more, i.e 5 times the value in patients undergoing cardiac surgery [2].

**Conclusions:** Sepsis related AKI is caused by a renal afferent vasoconstriction resulting in a reduced RBF and lowered RDO2 In combination with an anchanged RVO2, this results in a renal oxygen supply/demand mismatch.

**References**

1. Hoste EA, et al. Intensive Care Med 41(8):1411-23, 2015

2. Lannemyr L et al. Anesthesiology 126(2):205-213, 2017

**Table 1 (abstract P162).** Renal variables in early clinical septic shock

	Control group (n=58)	Septic shock (n=8)	p-value
Renal oxygen extraction	0.097 ± 0.027	0.124 ± 0.039	0.022
Renal blood flow (ml/min)	858 ± 26	696 ± 166	0.068
Renal vascular resistance (mmHg/ml/min)	0.082 ± 0.023	0.098 ± 0.023	0.069
Glomerular filtration rate (ml/min)	79.8 ± 24.3	54.4 ± 17.6	0.006
Renal blood flow/cardiac index	0.31 ± 0.79	0.22 ± 0.67	0.003
Renal oxygen delivery (ml/min)	122.3 ± 42.4	92.7 ± 23.5	0.037
Renal oxygen consumption (ml/min)	11.4 ± 3.1	11.4 ± 4.0	0.956

## P163 Utility of daily creatine kinase measurement within adult intensive care

### P Henderson^1^, J Adams^2^, A Blunsum^1^, M Casey^3^, N Killeen^1^, S Linnen^1^, J McKechnie^3^, A Puxty^1^

#### ^1^Glasgow Royal Infirmary, Glasgow, UK; ^2^Royal Alexandra Hospital, Paisley, UK; ^3^Queen Elizabeth University Hospital, Glasgow, UK

**Introduction:** The primary aim was to determine if the addition of daily creatine kinase (CK) measurement was usefully guiding decision making in intensive care units within Greater Glasgow and Clyde.

**Methods:** After a change to the daily blood ordering schedule to include CK, a retrospective audit was carried out covering a 5-month period within 3 intensive care units. All patients with CK >870 units/litre were included. Basic demographics, APACHE 2 score and admitting diagnosis were recorded. Utility of CK was assessed by determining the associated diagnosis and whether the diagnosis was first considered (diagnostic trigger) due to CK level, clinical suspicion or haematuria. Additionally, it was determined if and what actions had been taken based on the raised CK and associated diagnoses.

**Results:** Data was collected from 01/08/2016 to 31/12/2016. 276 patients were captured with CK >870 units/litre from an average combined admission rate of 200 patients/month [1]. Total male patients 191 (69.2%) and female 85 (30.8%). Age range 17 to 95 years (mean 54.7). APACHE 2 score range 0 to 45 (mean 20.9) with estimated mean mortality of 36.7%. 176 patients (63.8%) had associated diagnoses with elevated CK including: burns 2 (0.7%), compartment syndrome 7 (2.5%), myocardial infarction 20 (7.2%), myositis/myocarditis 2 (0.7%), neuroleptic malignant syndrome 1 (0.4%), rhabdomyolysis 61 (22.1%), serotonin syndrome 7 (2.5%), surgical procedure 76 (27.5%). As outlined in Fig. 1 the diagnostic trigger was the routine CK measurement in 65 patients (23.6%), prior clinical suspicion 108 (39.1%), haematuria 1 (0.4%) and unclear in 102 (36.9%). Action was required based on CK result/associated diagnosis in 95 patients (34.4%) with the specific actions outlined in Table 1.

**Conclusions:** With a raised CK resulting in a new diagnosis rate of 23.6% and a change in treatment rate of 34.4% the introduction of this test has proven useful within this cohort.

**Reference**

1. Cole S et al. SICSAG Audit of critical care in Scotland 10:30-32, 2017

**Table 1 (abstract P163).** Action taken

Action Taken	Number of patients (%)
Discontinuation of drugs	3 (1.1)
Fluid resuscitation/forced diuresis	54 (19.6)
Renal replacement therapy	25 (9.1)
Surgery	6 (2.2)
Diagnostic imaging	7 (2.5)
No action taken	181 (65.5)
Total	276 (100)

**Fig. 1 (abstract P163).**
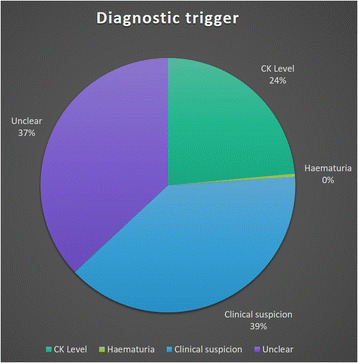
Diagnostic trigger

## P164 Creatinine-based formula (MDRD) versus cystatin c-based formula (simple cystatin c formula) for estimation of GFR in critically ill patients

### R Marinho^1^, T Neves^2^, M Santos^3^, A Marinho^1^

#### ^1^Centro Hospitalar do Porto, Porto, Portugal; ^2^Instituto de Ciencias Biomedicas Abel Salazar – Universidade do Porto, Porto, Portugal; ^3^Faculdade de Ciencias da Nutricao e Alimentacao da Universidade do Porto, Porto, Portugal

**Introduction:** Plasma or serum creatinine is the most commonly used diagnostic marker for the estimation of glomerular filtration rate (GFR) in clinical routine. Equations to estimate GFR based on serum creatinine have been introduced and the most validated and applied are the MDRD equation. Lately, the low molecular weight protein cystatin C was introduced as a GFR estimate (eGFR) superior to creatinine. However, there are conflicting reports regarding the superiority of Cys C over serum creatinine (Cr), with a few studies suggesting no significant difference. The aim of our study was to compare MDRD formula against Simple Cystatin C (Scys) formula for estimation of GFR in Critically Ill Patients.

**Methods:** 71 critically ill patients (56.3% women, mean age 66,23±13.77 years, with a mortality rate of 18.3%) were enrolled. In each patient, GFR on the first day of admission to an ICU, was calculated using MDRD equation and Scys formulas. Statistical analysis was performed using MedCalc software.

**Results:** The mean serum creatinine was 1.39±0.95 mg/dl, mean GFR (MDRD) was 77.23±44.4 mL/min/1,73m2. The mean serum cystatin was 1.655±1.06 mg/L, mean GFR (Scys) was 62.23±36.41 mL/min/1,73m2. The correlation coefficient (r value) between calculated GFR based on MDRD method and Simple Cystatin C (Scys) formula was 0.716 (P = 0.01)

**Conclusions:** The correlation analysis showed the eGFRs from every formula could all to some extent reflect the glomerular function or GFR accurately. The GFR (Scys) formula was a quickly and accurate method for estimating GFR and may apply clinically in critically ill patients.

## P165 Perioperative chloride levels and acute kidney injury after liver transplantation: a retrospective observational study

### S Choi^1^, C Jung^1^, H Lee^1^, S Yoo^1^, H Ryu^1^, H Jang^2^

#### ^1^Seoul National University College of Medicine, Seoul, South Korea; ^2^Samsung Medical Center, Sungkyunkwan University College of Medicine, Seoul, South Korea

**Introduction:** The risk of developing acute kidney injury (AKI) after liver transplantation in the immediate postoperative period ranges between 17 to 95%. Most studies in critically ill and surgical patients evaluated the link between chloride-rich resuscitation fluids, not serum chloride levels, and the incidence of AKI. The association between preoperative chloride level or difference in perioperative chloride levels and the incidence of postoperative AKI after liver transplantation were evaluated.

**Methods:** Adult patients (>=18 years old) who underwent liver transplantation at Seoul National University Hospital between 2004 and 2015 were included in the retrospective analysis. The difference between preoperative serum chloride level and the immediate postoperative serum chloride level was defined as intraoperative chloride loading. Postoperative AKI within 7 days of liver transplantation was diagnosed according to the RIFLE criteria. Patients were divided into normochloremia group (96-106 mEq/L), hypochloremia group (<96 mEq/L), or hyperchloremia group (>106 mEq/L) according to their preoperative chloride level. Intraoperative chloride loading was defined as the difference between preoperative serum chloride level and immediate postoperative serum chloride level.

**Results:** AKI developed in 58.8% (630/1071) of the patients. AKI was more frequent in patients with hyperchloremia (adjusted OR 1.44 [95% CI 1.08-1.90], P=0.01) and hypochloremia (adjusted OR 1.25 [95% CI 1.03-1.53], P=0.03) compared to patients with preoperative normochloremia. MELD scores > 11 and age >56 years were also associated with increased risk of AKI. Intraoperative chloride loading was not a significant risk factor for AKI after liver transplantation.

**Conclusions:** Preoperative hyperchloremia and hypochloremia were both associated with an increased risk of developing AKI in the immediate postoperative period after liver transplantation.

## P166 Urinary strong ion difference as an early marker of acute kidney injury in septic patients

### M Cicetti, A Dell’anna, C Dominedò, A Ionescu, C Sonnino, E Tarascio, I Barattucci, SL Cutuli, M Antonelli

#### Fondazione Policlinico A. Gemelli Università Cattolica Sacro Cuore, Rome, Italy

**Introduction:** Sepsis is a major cause of acute kidney injury (AKI) which is associated with increased morbidity and mortality. Serum creatinine (sCr) increase is a late marker of AKI. Urinary Strong Ion Difference (SIDu = [Na+]u + [K+]u – [Cl-]u) reflects the kidney’s ability to compensate blood pH variations. The aim of this study is to evaluate the role of SIDu as an early marker of septic AKI.

**Methods:** This prospective, observational, monocentric study included adult patients admitted to the ICU with sepsis and preserved kidney function. We excluded patients treated with diuretic before enrollment. Patients were daily evaluated to assess AKI according to KDIGO criteria. We studied the biochemical profile of blood and urine and calculated plasmatic apparent SID (SIDa), effective SID (SIDe), SIDu.

**Results:** Fifty-five patients (men 33, age 58±17) were included in the analysis from September 2016 to June 2017 (Table 1), 19 (34%) of whom developed AKI. There was no significant difference in SIDu values between patients who developed AKI (AKIyes) and those who did not (AKIno). No association was found between SIDu and sCr, SIDu and creatinine clearance (Table 2). Urinary Na+ ([Na+]u) and Cl- ([Cl-]u) were not correlated to their plasmatic values. [Na+]u and [Cl-]u were significantly lower in the AKIyes group (baseline: Na+: 36.5[13-76] vs 91[51-139]; Cl-: 51[33-70] vs 110[66-170]), p=0.01) and these alterations occurred before the onset of derangements in urinary output and sCr (Figs. 1 and 2). [Na+]u and [Cl-]u showed a better AUC in predicting AKI compared to sCr (sCr: 0.54; [Na+]u: 0.76; [Cl-]u: 0.77, p=0.047 and 0.036 respectively).

**Conclusions:** SIDu was not found to be a reliable marker of AKI but further studies are needed to evaluate its diagnostic value. Conversely, [Na+]u and [Cl-]u could be simple and inexpensive markers of renal dysfunction to be used in AKI diagnosis and management. Written informed consent was obtained from all patients.

**Table 1 (abstract P166).** General characteristics at baseline

	AKIyes (19)	AKIno (36)	P
Age (years)	59±16	57±17	ns
SAPS II	41[31-50]	37.5[27.5-44.5]	ns
SOFA score	5[3-7]	5[3.5-8]	ns
IN (mL)	3189[2474-3751]	3202[2725-3615]	ns
OUT (mL)	1955[1381-2421]	2765[2215-3600]	0.007
Urine Output (mL)	1890[1381-2081]	2735[2075-3462]	0.0017
Balance (mL)	885[162-1623]	191[-653-1425]	0.0505

**Table 2 (abstract P166).** Arterial blood gas analysis at baseline for patients with and without AKI

	AKIyes (19)	AKIno (36)	P
pH	7.43[7.39-7.49]	7.43[7.40-7.46]	ns
Na+(mmol/L)	142±6	143±5	ns
Cl- (mmol/L)	105±5	106±4	ns
SIDa(mmol/L)	38.3±4	39±4	ns
Na+u (mmol/L)	36.5[13-76	91[51-139]	0.006
Cl-u (mmol/L)	51[33-70]	110[66-170]	0.047
SIDu (mmol/L)	11[-2-29]	14[1-42]	ns

**Fig. 1 (abstract P166).**
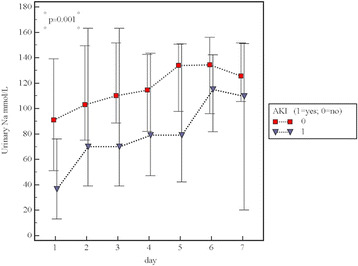
ANOVA urinary Na during the first 7 days since admission

**Fig. 2 (abstract P166).**
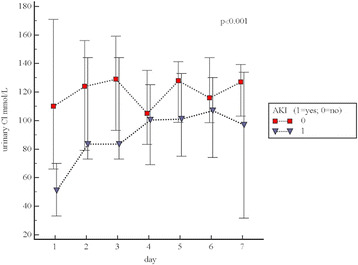
ANOVA urinary Cl during the first 7 days since admission

## P167 Urinary electrolytes as early indicators of acute kidney injury after major abdominal surgery

### D Marouli, E Papadakis, P Sirogianni, G Papadopoulos, E Lilitsis, E Pediaditis, A Papaioannou, D Georgopoulos, H Askitopoulou

#### University Hospital Heraklion, Heraklion, Greece

**Introduction:** Perioperative Acute Kidney Injury (AKI) is associated with significant morbidity and mortality [1]. Certain urinary biochemical parameters seem to have a standardized behavior during AKI development and may act as surrogates of decreased glomerular filtration rate (GFR) aiding in early AKI diagnosis [2]. Aim of this prospective observational study was the evaluation of urinary biochemical parameters as early indicators of AKI in a cohort of major surgery patients.

**Methods:** 68 patients were studied. AKI was defined according to AKIN criteria within 48 hrs after surgery [3]. At pre-defined time points (preoperatively, recovery room [RR] and on postoperative days [POD] 1 to 3) simultaneous serum and urine samples were analyzed for urea, creatinine, Na, K, Cl, while fractional excretions of Na (FENa), Urea (FEUrea), K (FEK), urinary strong ion difference (SIDU) and estimated GFR (eGFR) were calculated.

**Results:** 16 patients (23.5%) developed AKI. While there was no difference in preoperative eGFR between AKI and non-AKI patients (75.3±16 vs 83.9±15.2ml/min/m2, p=0.09), RR eGFR was already lower in AKI patients (69.5±18.7 vs 85.7±15.6ml/min/m2, p=0.001). This was accompanied by significantly lower NaU (82.7±26.8 vs 108.1±41.9mEq/l, p=0.002) and ClU (94.7±32.9 vs 114.5±33.4mEq/l, p=0.041) values, as well as significantly higher FEK (62.5±41.5 vs 24.8±16.5%, p=0.002). FENa and FEUrea differed significantly between the two groups on POD 1, whereas SIDU did not differ.

**Conclusions:** In a general surgery population low NaU and ClU values, as well as high FEK values were already evident immediately after surgery, probably representing GFR impairment preceding formal AKI diagnosis. Additional studies must confirm these findings and reevaluate these simple parameters as potential AKI monitoring tools.

**References**

1. Hobson C et al. Ann Surg 261:1207-14, 2015

2. Maciel AT et al. Renal Failure 38(10):1607-15, 2016

3. Acute Kidney Injury Work Group: Kidney Int; 2:1-138, 2012

## P168 Urinary liver-type fatty acid-binding protein is the novel biomarker for diagnosis of acute kidney injury secondary to sepsis

### T Komuro, T Ota

#### Shonan Kamakura General Hospital, Kamakura, Kanagawa, Japan

**Introduction:** Acute kidney injury (AKI) is the predictor of poor prognosis for the patient with sepsis and septic shock. Several diagnostic criteria for AKI is used on clinical settings, but useful biomarker is not known yet. Urinary liver-type fatty acid-binding protein(L-FABP) is associated with kidney function and AKI[1], But that is not still discussed about AKI secondary to sepsis. Thus, we conducted the study of the association between urine L-FABP and AKI with secondary to sepsis.

**Methods:** From May 2017 to October 2017, We collected adult sepsis patients admitted to our Intensive Care Unit(ICU). Patients were diagnosed with sepsis-3 definition[2].Kidney Disease Improving Global Outcomes (KDIGO) criteria was used for diagnosis of AKI.L-FABP was measured when patient admitted to our ICU. Sensitivity and Specificity of L-FABP for diagnosis of AKI was assessed by AUROC curve.

**Results:** Ninety-five patients participated in this study. Systemic Organ Falure Assessment (SOFA) score was 7(median, IQR:5-10). Fifty-seven(60%) patients were diagnosed with AKI by KDIGO criteria. Serum creatinine level of AKI patients was 1.65mg/dl (median, IQR:1.40-2.53). Urine L-FABP level of AKI patient was 109.23μ g/g Cr(median, IQR:27.58-671.33). Urine output was 1147.5ml(median, IQR:725.25-1747.5). The estimated sensitivity of urinary L-FABP level for diagnosing AKI was 81.1% and specificity was 53.4%. AUROC was 0.705(95%CI:0.6-0.811) (Fig. 1). The cut-off line of L-FABP was 95.71μg/g Cr.

**Conclusions:** L-FABP can be the novel biomarker for diagnosis of AKI. Further investigation need for diagnostic value of L-FABP and usefulness of early intervention for AKI used by L-FABP.

**References**

1. Susantitaphong P et al. Am J Kidney Dis 61(3):430-9, 2013

2. Singer M et al. JAMA 23;315(8):801-10, 2016

**Fig. 1 (abstract P168).**
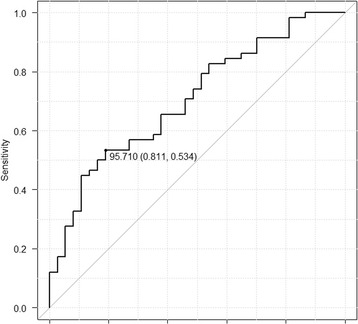
The AUROC curve of L-FABP for diagnosis of AKI

## P169 Vitamin D metabolite concentrations in critically ill patients with acute kidney injury

### L Cameron, U Blanco Alonso, A Bociek, A Kelly, G Hampson, M Ostermann

#### Guy’s and St Thomas’ NHS Foundation Trust, London, UK

**Introduction:** Biotransformation of 25-hydroxyvitamin D to active 1,25(OH)_2_D occurs primarily in the kidney. Our aim was to explore whether this process was altered in patients with acute kidney injury (AKI).

**Methods:** Consecutive patients admitted to critical care at a tertiary hospital were recruited. The AKI group comprised patients with KDIGO stage II or stage III AKI; the non-AKI group were patients requiring cardiovascular or respiratory support, but with no AKI. Vitamin D metabolite concentrations were measured on days 0, 2 and 5. Statistical analysis included comparison between groups at each time point, and longitudinal profiles of vitamin D metabolites.

**Results:** Interim analysis of 55 participants (44% of the recruitment target) showed that 1,25(OH)_2_D concentrations were significantly lower in patients with AKI at day 2 and day 5. Considering longitudinal changes, 25-hydroxyvitamin D profiles were not different between the groups (Fig. 1) but there was a trend towards a longitudinal increase in 1,25(OH)_2_D in patients without AKI, which was not seen in AKI patients (Fig. 2).

**Conclusions:** Interim analysis indicates significant differences in concentrations of 1,25(OH)_2_D, but not 25(OH)D, in critically ill patients with AKI. Recruitment is ongoing and further results are awaited.

**Fig. 1 (abstract P169).**
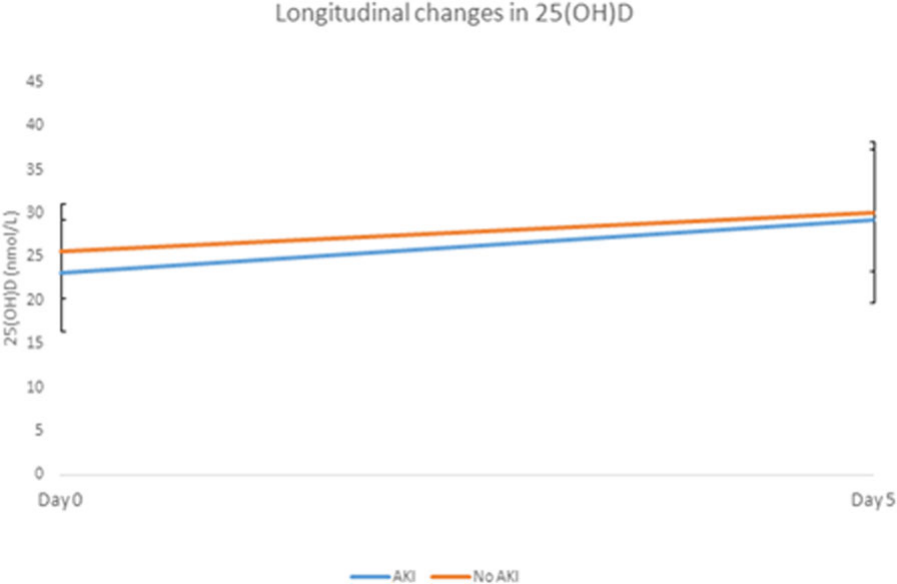
Longitudinal changes in 25(OH)D.No significant difference in the longitudinal profiles of patients with AKI and those with no AKI was found.

**Fig. 2 (abstract P169).**
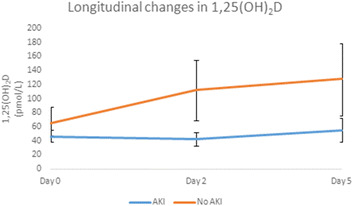
Longitudinal changes in 1,25(OH)2D. A significant difference in the longitudinal profiles of patients with AKI and those with no AKI was found (p=0.047). This effect was attenuated with a statistical correction for unequal variance between groups.

## P170 Effects of fenoldopam on renal function in critically ill patients undergoing a strictly conservative strategy of fluid management

### S Tribuzi, LP Bucci, C Scaramucci, V Vano, A Naccarato, M Mercieri

#### Sant’Andrea, Rome, Italy

**Introduction:** Acute renal failure affects from 1% to 25% of patients in the intensive care units (ICUs)1 and it is associated with excess mortality. Hydratation is a useful preventive measure but it is often controindicated in critically ill patients who, on the contrary, often benefit by a strictly conservative strategy of fluid management. Fenoldopam, a selective dopamine 1-receptor agonist, increases renal blood flow and glomerular filtration rate by vasodilating selectively the afferent arteriole of renal glomerulus. The aim of our study is to compare renal effects of fenoldopam and placebo in critically ill patients undergoing a restrictive fluid management.

**Methods:** We enrolled 130 patients admitted to our ICU. Patients were assigned by randomization to study groups: fenoldopam (n=64) and placebo (n=66). Fenoldopam was infused continuously at 0,1 mcg/Kg/min and equivalent volume for placebo during a period of seven days.

Creatinine, cystatin C and creatinine clearance were daily measured as markers of renal function. The incidence of AKI according to RIFLE criteria (Risk, Injury, Failure, Loss, End Stage kidney disease) was also calculated.

**Results:** Patients with a negative fluid balance at the end of the week (~ -5000 ml, p=0,0001) were included in the analysis, 32 in the placebo group and 38 in the fenoldopam group. There were not significant differences in the trend of creatinine, creatinine clearance, cystatin C and in the incidence of AKI between the groups during the week of infusion.

**Conclusions:** A continuous infusion of fenoldopam at 0,1 mcg/kg/min does not improve renal function and does not prevent AKI in critically ill patients undergoing a strictly conservative strategy of fluid management.

**Reference**

1. Bellomo R et al. Intensive Care Med 27:1685-1688, 2001

## P171 Dysphagia triage protocol as a tool in the intensive care risk management

### CE Bosso^1^, PC Dutil Ribeiro^2^, M Valerio^2^, O Alves de Souza^2^, A Pireneus Cardoso^1^, RD Jorge Caetano^3^, L De Oliveira^3^, BR Correa^3^, F Fernandes Lanziani^3^

### ^1^Instituto do Coração de Presidente Prudente, Presidente Prudente, Brazil; ^2^UNOESTE, Presidente Prudente, Brazil; ^3^Santa Casa de Misericórdia de Presidente Prudente, Presidente Prudente, Brazil

**Introduction:** This study aims to evaluate the efficacy of a protocol implemented for dysphagia risk factors [1] in hospitalized patients in a CICU (Coronary Intensive Care Unit).

**Methods:** Patients hospitalized in the CICU of a medium-sized hospital in Presidente Prudente, SP, Brazil, were subjected to a survey that screened for dysphagia during the period from January of 2016 to September of 2017. Patients with at least one risk factor for dysphagia were evaluated by a phonoaudiologist and are the subject of this study. The information was statistically analyzed using EPI INFO, version 7.2.2.2 software. Considering significant P <0.05 two-tailed, for logistic regressions multivariate estimated in the sample.

**Results:** For this study 1018 patients were selected, of which 57.41% were male and the mean age was 71.77 ± 10.96 years. A higher incidence of dysphagia was observed among patients who had at least one of the following risk factors: stroke (Odds Ratio 9.58 p<0.001); brain tumor (OR 4.49 p=0.0013); chronic obstructive pulmonary disease (COPD) (OR 3.45 p=0.023); degenerative diseases (OR 16.76 p<0.001); lower level of consciousness (OR 13.62 p<0.001); ataxic respiration (OR 2.24 p<0.001); aspiration pneumonia (OR 7.04 p<0.001); orotracheal intubation >48h (OR 13.35 p<0.001); tracheostomy (OR 12.99 p<0.001); airway secretion (OR 24.91 p<0.001); nasoenteral tube (OR 14.9 p<0.001); gastrostomy (OR 4.58 p=0.030). There was no statistical significance for age >60, traumatic brain injury, oropharyngeal surgery and unfavorable dentition. Four factors appeared less than 3 times and could not be analyzed (chagas disease, human immunodeficiency virus (HIV), orofacial burn and excess saliva).

**Conclusions:** We concluded that the dysphagia triage protocol insertion was effective to identify dysphagic patients and can be used as an additional tool in the intensive care risk management.

**Reference**

1. Werle RW et al. CoDAS 28: 646-652, 2016.

## P172 Apnea oxygenation : a novel respiratory system model for physiological studies using high-flow nasal cannula oxygen therapy

### V Masy, B Bihin, J Petit

#### CHU UCL Namur - Godinne, Yvoir, Belgium

**Introduction:** Since the advent of high-flow nasal cannula (HFNC) oxygen therapy, apnea oxygenation has once again been the subject of numerous clinical studies whose results are conflicting. The physiological bases of this age old concept, more recently applied to endotracheal intubation, have never been confirmed by current methods. We therefore decided to study the effects of an apnea oxygenation period under HFNC oxygen therapy by means of a novel modelization of the respiratory system.

**Methods:** Firstly, an airway model was built with anatomical, physical and physiological attributes similar to that of a healthy subject (Fig. 1). This system reproduces the physiological evolution of intrapulmonary gases during apnea by progressively increasing CO_2_ levels after having cut off previous O_2_ supplies (FIO_2_ 21%). Secondly, the effects of a HFNC apnea oxygenation of 50l/min with an FIO_2_ of 100% were analyzed by collecting intrapulmonary gas samples at regular intervals (Fig. 2).

**Results:** After 1 minute of apnea oxygenation, intrapulmonary oxygen levels remain stable at 21%. After 5 minutes, oxygen fraction reaches 33%, and increases up to 45% in 10 minutes. Regarding CO_2_ levels, no significant modifications were observed.

**Conclusions:** A novel experimental and physiological model of the respiratory system has been developed and confirms the existence of an alveolar oxygen supply as well as the lack of a CO_2_ washout during HFNC apnea oxygenation. However, these effects are only observed after a delay of about 1.5 to 2 minutes. Therefore, the clinical interests of this technique to reduce apnea-induced desaturation during intubation of a hypoxemic patient in the ICU seem limited without adequate preoxygenation. Combination of both preoxygenation and apnea oxygenation by HFNC can most likely explain positive results observed in other clinical studies.

**Fig. 1 (abstract P172).**
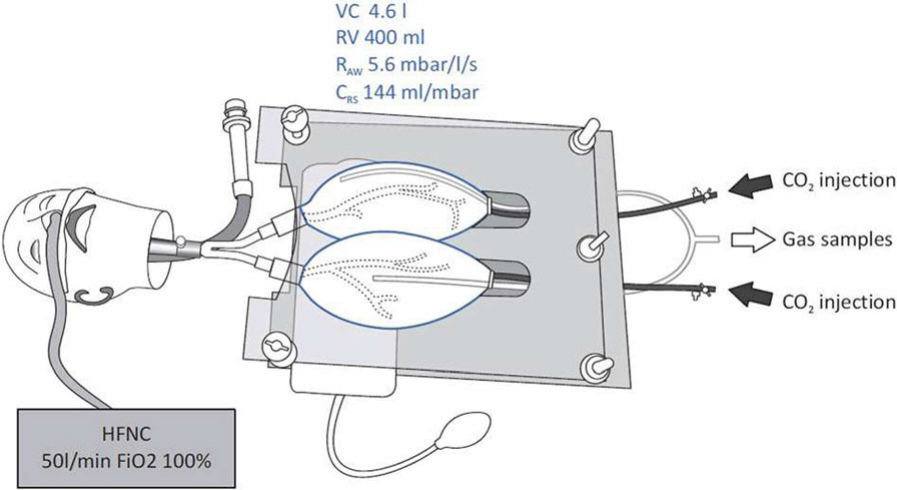
Respiratory system model

**Fig. 2 (abstract P172).**
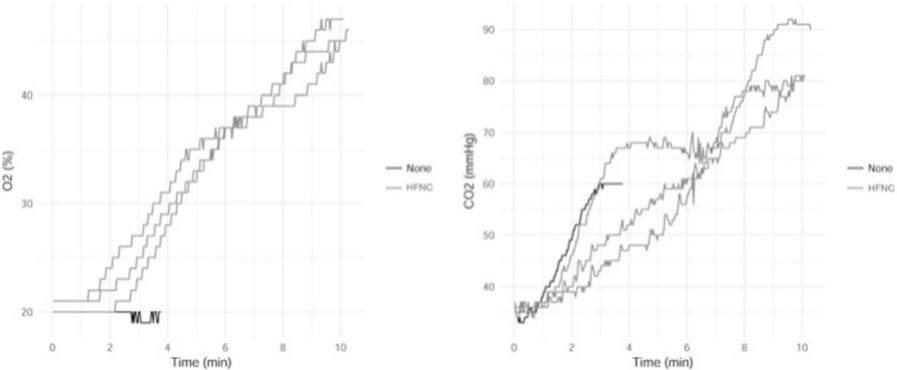
Evolution of intrapulmonary gases during apnea; HFNC : high-flow nasal cannula

## P173 Effect of 4% nebulized lignocaine versus 2% nebulized lignocaine for awake fibreoptic nasotracheal intubation in maxillofacial injuries in emergency department

### H Abbas, L Kumar

#### King George’s Medical University,Lucknow,India, Lucknow, India

**Introduction:** Topical lignocaine is most commonly used pharmacological agent for anaesthetizing upper airway during fibreoptic bronchoscopy. We compare the effectiveness of two different concentrations, 2% lignocaine and 4% lignocaine, in nebulised form for airway anaesthesia during awake fibreoptic nasotracheal intubation in terms of patient’s comfort and optimal intubating conditions, intubation time.

**Methods:** Institutional Ethics Committee approved the study and written informed consent obtained; patients of either sex, between 18-55 years age with anticipated difficult airway planned for intubation were included for this study. Patients were randomly allocated into two groups (A and B) based on sealed envelope method; patients and observers were blinded by using prefilled syringes of lignocaine.One group was nebulized with 10ml of 4% lignocaine(Group A) and other with 10 ml of 2% lignocaine(Group B) in coded syringes via ultrasonic nebuliser for 10 minutes followed by Inj midazolam 0.05 mg/kg IV and Inj Fentanyl 1 microgram/kg IV just before the procedure. The fibreoptic broncoscope was introduced via nostril and the other nostril was used for oxygen insufflation (3–4 L/min). The fibroscope was introduced through the glottic opening and visualising tracheal rings and carina.The endotracheal tube railroaded over the fiberscope and cuff inflated.

**Results:** The primary outcome measure was patient’s comfort during awake fibreoptic nasotracheal intubation. The mean patient comfort Puchner scale score of Group A was 1.30 ± 0.08 and of Group B was 2.23 ± 0.12. The mean value of Puchner scale of Group B was significantly higher.The mean procedural time of Group B was significantly higher (15.1%) as compared to Group A (p<0.001). The no of intubations attempts did not differ between the two groups.

**Conclusions:** 4% nebulised lidocaine provided adequate airway anaesthesia and optimal intubating conditions, patient comfort, stable hemodynamics.

**Table 1 (abstract P173).** Puchner Comfort Scale

1	No reaction	1
2	Slight grimacing	2
3	Heavy grimacing	3
4	Verbal objection	4
5	Defensive movements of head and hands	5

**Fig. 1 (abstract P173).**
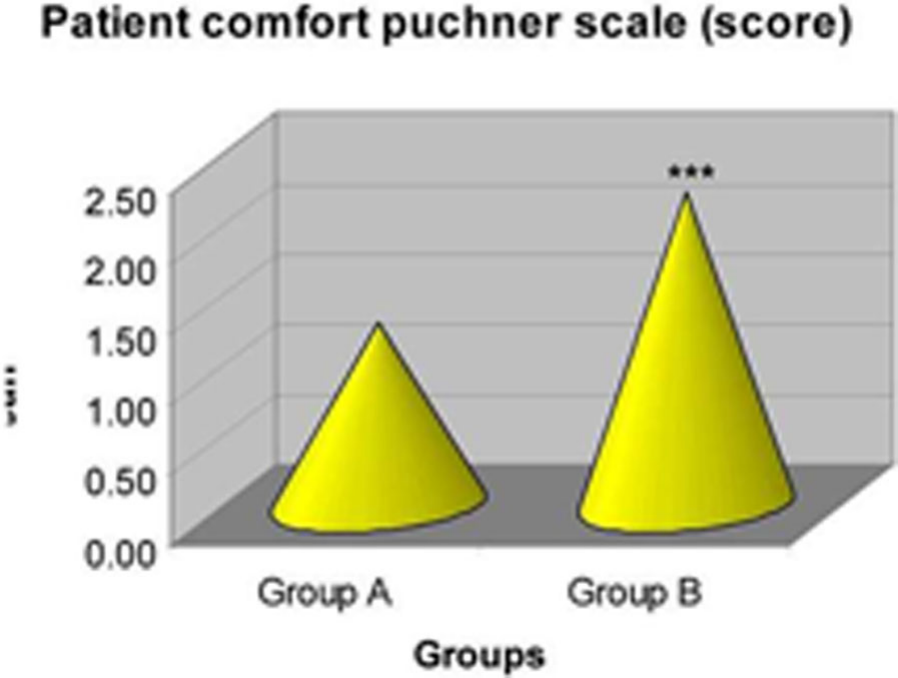
Five point Puchner scale. Group A (4% lignocaine) having more patient comfort with mean value of score 1.30 ± 0.08, as compared to Group B (2% lignocaine) having mean value of 2.23 ± 0.12 (p < 0.001)

**Fig. 2 (abstract P173).**
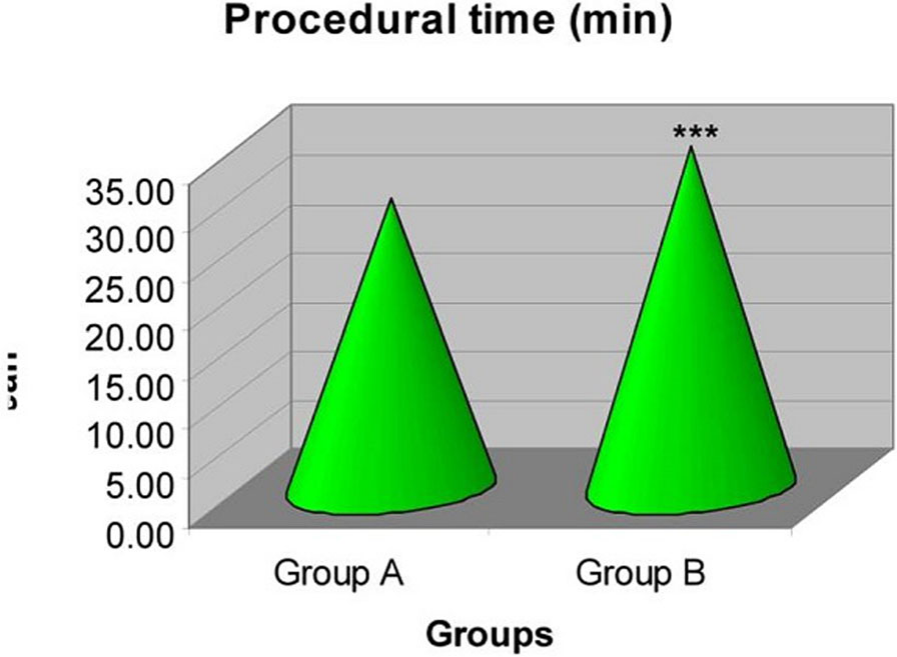
The mean procedural time to secure airway in Group A (4% lignocaine) was 29.67 ± 5.40 minutes and in Group B (2% lignocaine) it was 34.93 ± 5.52 minutes. The difference in mean time duration was statistically significant (p<0.001)

## P174 Video laryngoscopy versus direct laryngoscopy for emergency orotracheal intubation outside the operating room - a systematic review and meta-analysis

### V Bennett^1^, N Arulkumaran^2^, J Lowe^3^, R Ions^4^, M Mendoza Ruano^5^, M Dunser^6^

#### ^1^St George’s Hospital, London, UK; ^2^University College London, London, UK; ^3^Arrowe Park Hospital, Merseyside, UK; ^4^Musgrove Park Hospital, Taunton, UK; ^5^Hospital Universitario y Politecnico La Fe, Valencia, Spain; ^6^Innsbruck Medical University, Innsbruck, Austria

**Introduction:** This systematic review and meta-analysis aims to investigate whether video laryngoscopy (VL) improves the success of orotracheal intubation, when compared with direct laryngoscopy (DL).

**Methods:** A systematic search of Pubmed, Embase, and CENTRAL databases was performed to identify studies comparing VL and DL for emergency orotracheal intubations outside the operating room. The primary outcome was rate of first pass intubation. Subgroup analyses by location, device used, clinician experience, and clinical scenario were performed. The secondary outcome was rate of complications.

**Results:** The search identified 32 studies with 15,064 emergency intubations. There was no overall difference in first-pass intubation with VL compared to DL. Subgroup analysis showed first-pass intubations were increased with VL in the intensive care unit (ICU) (2.02 (1.43-2.85); p<0.01), but not in the emergency department or pre-hospital setting. Rate of first-pass intubations were similar with Glidescope® and DL, but improved with the CMAC® (1.32(1.08-1.62); p=0.007). There was greater first-pass intubation with VL than DL among novice/trainee clinicians (OR=1.95 (1.45-2.64); p<0.001), but not among experienced clinicians or paramedics/nurses. There was no difference in first-pass intubation with VL and DL during cardiopulmonary resuscitation or trauma. VL was associated with fewer oesophageal intubations than DL (OR=0.31 (0.14-0.69); p=0.004), but more arterial hypotension (OR=1.49 (1.00-2.23); p=0.05).

**Conclusions:** In summary, compared to DL, VL is associated with greater first-pass emergency intubation in the ICU and among less experienced clinicians. VL is associated with reduced oesophageal intubations but a greater incidence of arterial hypotension.

## P175 Compared success rate between direct laryngoscope and video laryngoscope for emergency intubation, in Emergency Department: Randomized Control Trial

### P Sanguanwit, N Laowattana

#### Ramathibodi Hospital, Bangkok, Thailand

**Introduction:** Video Laryngoscope was used as an alternative to intubate in the Emergency room, designed for tracheal intubation more success [1, 2].

**Methods:** We performed a prospective randomized controlled trial study of 158 patients who had sign of respiratory failure or met indication for intubation from July 2015 to June 2016. Patients were randomly by SNOSE technique; assigned to Video laryngoscope first or Direct laryngoscope first. We collect the Demographics, Difficult Intubation Predictor, Rapid Sequence Intubation, attempt, Cormack-Lehane view and immediate complication. Primary outcome was first attempt success rate of intubation.

**Results:** First attempt success rate of Video laryngoscope was 73.1% trend to better than Direct laryngoscope was 58.8%, (P=0.06), Good Glottic view (Cormack-Lehane view 1-2) of Video laryngoscope was 88.5% better than Direct laryngoscope 71.3%, and statistically significant (P=0.03), no statistical significant in immediate serious complication between Direct laryngoscope or Video laryngoscope.

**Conclusions:** Compared to the success rate between using Video laryngoscope or Direct laryngoscope for intubation, Video laryngoscope trend to better success rate, and better glottic view.

**References**

1. Choi HJ et al. Emerg Med J 27(5):380-2, 2010

2. Mosier JM et al. J Emerg Med 42(6):629-34, 2012.

## P176 10-year cohort of prehospital intubations and rescue airway techniques by helicopter emergency medical service physicians: a retrospective database study

### P De Jong, C Slagt, N Hoogerwerf

#### Radboudumc, Nijmegen, Netherlands

**Introduction:** In the Netherlands the pre-hospital Helicopter Emergency Medical Service (HEMS) is physician based and an adjunct to ambulance services. All four HEMS stations together cover 24/7 specialist medical care in the Netherlands. In many dispatches the added value is airway related [1]. As part of our quality control cycle, all airway related procedures were analysed. High quality airway management is characterized by high overall and first pass endotracheal intubation (ETI) success [2].

**Methods:** The HEMS database was analysed for all patients in whom prehospital advanced airway management was performed in the period 2007-2017. Balloon/mask ventilation, supraglottic airway (SGA) devices, total intubation attempts, Cormack & Lehane (C&L) intubation grades, successful ETI, primary and rescue surgical airway procedures and professional background were reviewed.

**Results:** In the 10-year period, there were 17075 dispatch calls. In total 8127 patients were treated in the prehospital setting by our HEMS. Of those, 3233 required a secured airway. ETI was successful in 3078 of 3148 (97.8%). In the remaining 70 patients (Fig. 1) an alternative airway was needed. Rescue surgical airway was performed in 1.4%, 0.5 % received a rescue SGA, rescue balloon/mask ventilation was applied in 0.2% of cases, 1 was allowed to regain spontaneous ventilation and in 0.1% of patients all airway management failed. HEMS physicians, ambulance paramedics, HEMS paramedics and others (e.g. German emergency physicians) had ETI first pass success rates of 83.4%, 59.6%, 62.4% and 84.5% respectively (Fig. 2). Difficult laryngoscopy (no epiglottis visible) was reported in 2.2% of patients (Table 1).

**Conclusions:** Our data show that airway management performed by a physician based HEMS operation is safe and has a high overall ETI success rate of 97.8%. The total success rate is accompanied by a high first pass ETI success rate.

**References**

1. Slagt C et al. Air Med J 23:36-7, 2004

2. Peters J et al. Eur J Emerg Med 22:391-4, 2015

**Table 1 (abstract P176).** C&L intubation grade

	Number of patients (%)
I	1632 (64.0)
II	644 (25.3)
III	218 (8.6)
IV	57 (2.2)
Unknown	681 (21.1)

**Table 2 (abstract P176).** Airway attempts

	Number of patients (%)
1	1960 (70.3)
2	630 (22.6)
3	160 (5.7)
>3	40 (1.4)
Unknown	443 (13.7)

**Fig. 1 (abstract P176).**
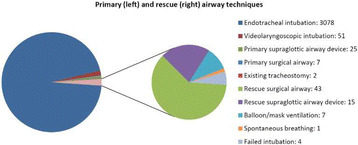
Primary and rescue airway techniques. Total number of patients: 3233

**Fig. 2 (abstract P176).**
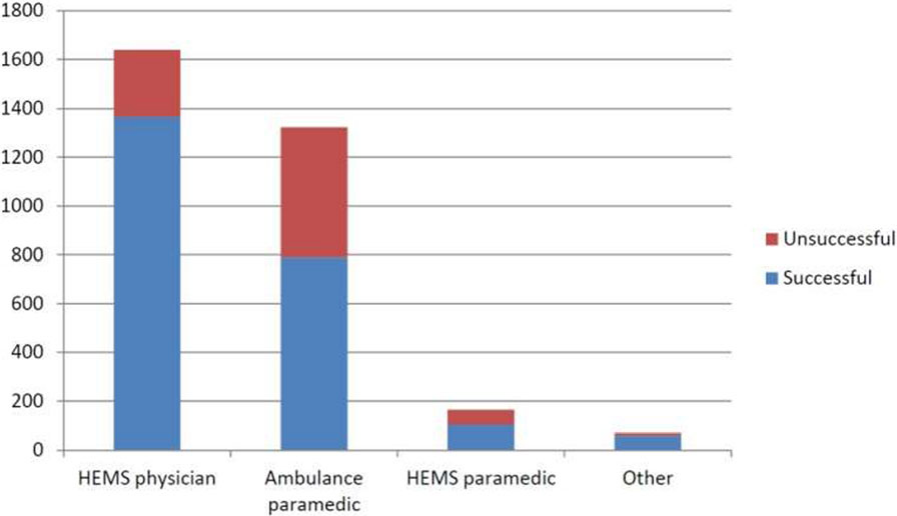
First pass endotracheal intubation success rates by professional background

## P177 Identification of factors associated with event occurrence due to unsafe management of endotracheal tubes

### E Ishida^1^, K Kobayashi^1^, Y Kobayashi^1^, Y Shiraishi^1^, M Yamamoto^1^, T Kuroda^1^, Y Iesaki^1^, Y Tsutsumi^1^, R Hosoya^1^, H Yasuda^2^

#### ^1^Japanese Red Cross Musashino Hospital, Tokyo, Japan; ^2^Kameda Medical Center, Chiba, Japan

**Introduction:** Incidences associated with endotracheal tubes are frequent during mechanical ventilation (MV) of intensive care unit (ICU) patients and can be associated with poor outcomes for patients and detrimental effects on health care facilities. Here, we aimed to identify factors associated with Event occurrence due to Unsafe Management of Endotracheal Tubes (E-UMET).

**Methods:** A retrospective observational study was conducted in three ICUs: one surgical ICU, one stroke ICU, and one emergency department, at a tertiary hospital in Japan from 1 April 2016 to 31 March 2017. Patients requiring MV and oral intubation during their ICU stay were included. The primary finding was the incidence rate of E-UMET (biting, unplanned extubations, and/or displacement of the endotracheal tube). The patients were divided into two groups: with or without E-UMET. To investigate E-UMET, potential factors possibly related to its occurrence were obtained from electronic medical records. We conducted univariable and multivariable analyses to investigate E-UMET factors.

**Results:** Of 410 patients, E-UMET occurred in 112 (27.3%). The mean and standard deviation for age and Acute Physiology and Chronic Health Evaluation (APACHE) II score were 66 (17) and 25 (7), respectively. According to a multivariate logistic-regression analysis, significant risk factors associated with E-UMET included patients of neurosurgery (odds ratio (OR) 3.3; 95% CI, 1.51-7.46; p=0.003), sedative administration (OR 2.9; 95% CI, 1.63-5.32; p<0.001), and higher Richmond Agitation-Sedation Scale (RASS) scores (OR 1.4; 95% CI, 1.24-1.77; p<0.001). The use of a restraint (OR 0.4; 95% CI, 0.22-0.95; p=0.003) was an independent factor associated with a lower probability of E-UMET.

**Conclusions:** This study suggests that risk factors associated with E-UMET include neurosurgery, higher RASS scores, and the administration of sedatives. Patients with these factors and longer oral intubation periods might require extra care.

## P178 Critical care extubation in Type II respiratory failure with nasal high flow therapy

### R D’Espiney, J Martin-Lazaro, M Brys, J Grundlingh, J Napier

#### Newham University Hospital, London, UK

**Introduction:** The use of nasal high flow (NHF) as a respiratory support therapy post-extubation has become increasingly more common. NHF has been shown to be non-inferior to NIV and reduces escalation needs compared to conventional oxygen therapy. Clinical outcomes using NHF in patients with Type II Respiratory Failure (RF) is less well understood. Our aim was to determine if NHF can be used successfully when extubating Type II RF patients compared to Type I RF.

**Methods:** We conducted a retrospective observational study on the use of NHF as an extubation respiratory support in 56 (n=56) consecutive patients in ICU over a 12-month period. Primary outcome was the need for escalation in therapy (NIV, Intubation and Palliation) post extubation. Patients were categorised as high risk if they scored >=1 from: Age>=75 years, BMI>=30 and >=1 medical comorbidity.

**Results:** Analysis was conducted on all fifty-six (n=56) patients. Type I RF group was composed of 25 (n=25) patients with a mean age of 62.7 (±SD) years. Type II RF group had 31 (n=31) patients with a mean age of 65.5 (±SD) years. In Type I RF 22 patients (88%) were successfully extubated with NHF compared to 21 patients (67.7%) in Type II. In Type II RF the outcomes were more variable with a greater requirement for NIV. Of these patients 16% required NIV, 3.2% required intubation and 12.9% received NHF therapy for palliation. A higher average BMI (30.32 vs 27.16 kg/m2) was found in unsuccessfully vs successfully extubated patients in Type II RF. In Type I RF escalation of therapy was equally distributed with 4% in each category.

**Conclusions:** The use of NHF for respiratory support post-extubation may become standard practice for Type I RF in critical care settings. Our data suggests that NHF can be used but with caution in Type II RF and clinicians should risk stratify patients to identify those at risk of re-intubation and post-extubation respiratory failure.

## P179 High flow nasal oxygen in critical care: an audit of practice and outcome

### T Tasnim^1^, A Kuravi^2^

#### ^1^University Hospital Birmingham, Birmingham, UK; ^2^Walsall Manor Hospital, Walsall, UK

**Introduction:** High Flow Nasal Oxygen (HFNO) is a relatively new therapy. This retrospective audit reviews the use of HFNO in relation to local guidelines in a critical care unit following its introduction.

**Methods:** Patients were identified by reviewing ICU charts between August 2015 and September 2016. Data was collected from electronic patient records and the ICNARC database. This included patients’ age, indication and duration of HFNO, mode of oxygen therapy and blood gases before, during and after. HFNO therapy interrupted by >24 hours were analysed as separate episodes. From the 47 patients identified, there were 53 episodes. These were subdivided into the respiratory pathology group (RPG) or post-extubation elective group (EG) based on the indication for HFNO therapy. Two episodes were excluded from the analysis due to the indication for HFNO.

**Results:** The median age of patients was 67 years. The mean duration of HFNO was 2.56 days. The mean APACHE score in RPG & EG were 17 and 18 respectively. In the RPG, 59.1% were weaned to Nasal Speculum(NS) or to Room Air(RA) with a further 13.6% to Face mask(FM). The mean, median(SD) PO2 noted before & during the HFNO therapy were 10.3, 9.45(2.58) & 10.07, 9.46(2.08) KPa. The mean, median(SD) of pH was 7.26, 7.45(1.1) pre HFNO, changing to 7.44, 7.46(0.05) on HFNO. In the EG, 57.1% were weaned to NS and 14.3% to FM. The mean, median(SD) of PaO2 before and during the therapy were 10.72, 10.1(3.66) & 9.08, 9.2(1.96) KPa. The mean, median(SD) of pH was 7.43, 7.45(0.05) pre HFNO changing to 7.47, 7.47(0.07) on HFNO. In 15% of episodes HFNO was used despite contraindications with no adverse events.

**Conclusions:** In most episodes, HFNO was used according to the local guidelines with no reported adverse events. More than 50% in both groups were successfully weaned to NS or RA. Only 27.3% in RPG and 28.6% in EG were escalated to NIV and Intubation. Despite the small cohort and multiple confounding factors, the audit has shown a general trend towards benefit from HFNO.

## P180 Infusion of antiseptic in an oral model. An innovative technique for possible prevention of ventilator associated pneumonia

### A Omar, F Teunissen, S Aboulnaga, S Hanoura, S Doiphode, A Alkhulaifi

#### Hamad medical corporation, Doha, Qatar

**Introduction:** Pathogenesis of ventilator-associated pneumonia (VAP) relies on colonization and microaspiration. Oral topical decontamination reduced the VAP incidence from 18 to 13% [1]. The persistence of antiseptic effect in the oral cavity is questionable; we hypothesize that continuous oral antiseptic infusion may offer a better decontamination.

Aim of the work: We developed endotracheal tube that allows continuous oral infusion of chlorhexidine (CHX), and we want to test the technique versus the conventional on bacterial colonization. (Provisional patent: 62359944)

**Methods:** A two identical bio models for the upper airways were manufactured by (3DX Diagnostics, USA) to adapt the modified and the ordinary endotracheal tubes (ETT). The two techniques tested were using six hourly disinfection with CHX (group A) versus disinfection through the 24 hours infusion technique (Group B). Five microorganisms plus mixed bacteria were used and each was tested for five times. Normal saline was used constantly to irrigate the biomodels and Ten ml aliquot was collected by the procedure end. Culturing of the aliquots from decanted broth pre and post disinfection was performed. The time to apply CHX by practitioner was also compared.

**Results:** There was a trend towards lower bacterial growth in group A in 5 experiments which reach statistical significance only with *Pseudomonas aeruginosa* (p=0.045). In one experiment the growth was lower in group B (Fig. 1). Additionally there was time saving advantage in group B (15±3.3 versus 5±1.2 min, p=0.01).

**Conclusions:** The novel technique got at least non inferior results, plus time saving advantage. These results may warrant future clinical trial.

**Reference**

1) Koeman M, et al. Am J Respir Crit Care Med. 173(12):1348-55, 2006.

**Fig. 1 (abstract P180).**
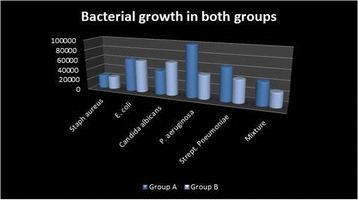
Bacterial growth in both groups

## P181 Non invasive measurement of particle flow from the small airways using PExA in vivo and during ex vivo lung perfusion in DCD donation

### E. Broberg, L. Pierre, M. Wlosinska, L. Algotsson, S. Lindstedt

#### Skane University Hospital, Lund, Sweden

**Introduction:** The optimal mechanical ventilation in the different phases in the LTX DCD (Lung Transplantation Donation after Cardio-circulatory Determination of Death) donation (in vivo, post mortem and ex vivo) is on debate. Monitoring airways non invasive online analysing different particle flow from the airways is never done before. In the present study we use a new technology for airway monitoring using mass spectrometric analysis of particle flow and their size distribution (PExA Particles in Expired Air). The exhaled particles are collected onto a substrate and possible for subsequent chemical analysis for biomarkers. Our hypothesis was that by analysing the particle flow online, we could optimise the mechanical ventilation. Our hypothesis was that a small particle flow would probably be more gentle for the lung than a large particle flow when the lung is squeezed out and the majority of all small airways are open.

**Methods:** In the present study we analyse the particle flow from the airways in vivo, post mortem and during ex vivo lung perfusion using different ventilation modes; Volume Controlled Ventilation (VCV) and Pressure Controlled Ventilation (PCV) comparing small tidal volumes(1) versus big tidal volumes(2) at different PEEP (Positive End-Expiratory Pressure) and after distribution of different drugs in six domestic pigs.

**Results:** We found that VCV resulted in a significant lower particle flow than PCV in vivo but in ex vivo settings the opposite was found (Fig. 1). In both in vivo and ex vivo settings we found that big tidal volume resulted in a larger particle flow than small tidal volumes.air.

**Conclusions:** The opening and the closure of the small airways reflect the particle flow from the airways. We found that different ventilation modes resulted in different particle flow from the airways. We believe this technology will be useful for monitoring mechanical ventilated patients to optimise ventilation and preserve the lung quality and has a high potential to detect new biomarkers in exhaled air.

**Fig. 1 (abstract P181).**
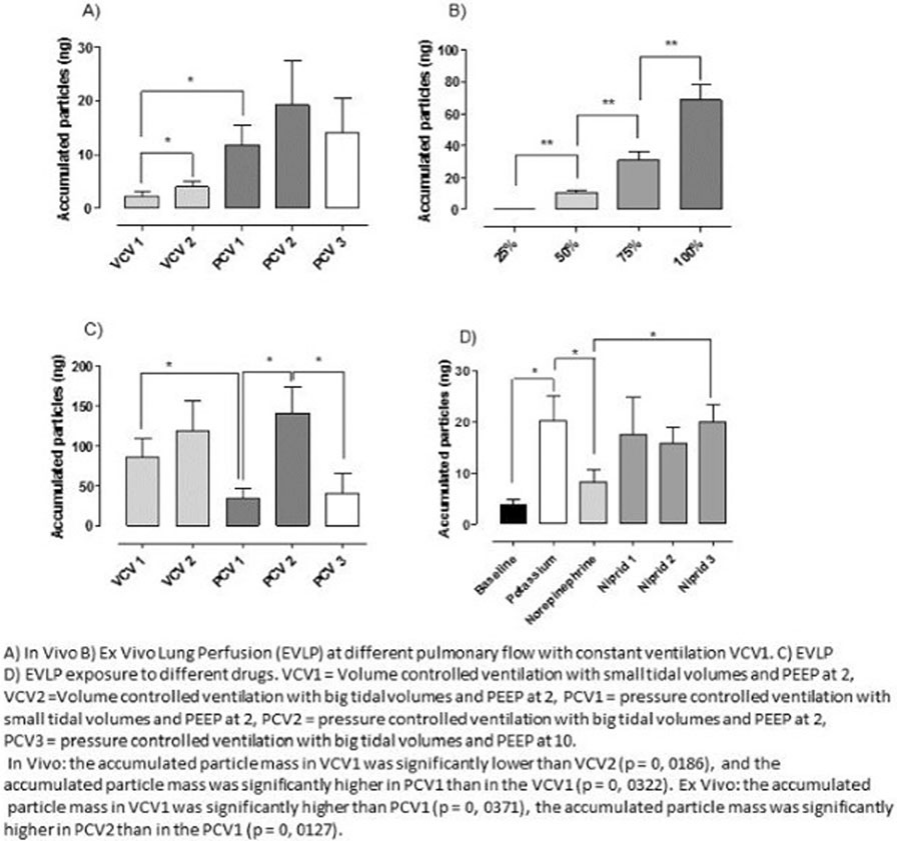
DCD PExA

## P183 Incentive to better practices in non-invasive ventilation

### N Matsud, G Correa, D Tucunduva, L Silva, V Veiga, T Alvarisa, N Postalli, P Travasos, R Vale, S Rojas

#### Hospital BP - A Beneficência Portuguêsa de São Paulo, São Paulo, Brazil

**Introduction:** Protocols for the use of non-invasive ventilation are associated with better outcomes in ICUs due to the reduction of the need for invasive ventilation and associated complications. The objective of this study is to evaluate the adherence to the noninvasive ventilation protocol in a large hospital intensive care unit.

**Methods:** We included all patients who used a non-invasive ventilation device from February 2016 to May 2017, based on the institutional protocol of Noninvasive Ventilation Indication.

**Results:** In the period, 4963 patients were admitted in the sector, and 641 (12.91%) used noninvasive ventilation, according to institutional protocol. The mean SAPS3 in the period was 43.9 points, with an expected mortality of 22.3%. The actual mortality rate was 11.2%. The average adherence to the protocol was 88.45% in 2016, rising to 98.4% in 2017. This increase was associated with an organization culture, training of the professionals involved - physicians and physiotherapists, monthly feedback of the results, with established plans. The main nonconformities were related to failure of records, indication of the resource or choice of interface and time of therapeutic response.

**Conclusions:** The adoption of protocols for the indication of non-invasive ventilation in highly complex patients was shown to be safe and effective in patients of high complexity, making it possible to reduce the number of patients on invasive ventilation and its complications.

## P184 Can non-invasive ventilation change the result of malaria with pulmonary dysfunction?

### A Costa e Silva, R Barbara, L Figueirôa

#### Clínica Multiperfil, Luanda, Angola

**Introduction:** Malaria is a common problem in underdeveloped countries, with an estimated mortality of more than one million people per year. Pulmonary involvement is one of the most serious manifestations of Plasmodium falciparum malaria. Non-invasive ventilation (NIV) decreases muscular works and improves gas exchange by recruitment of hypoventilated alveolus. In this context, we analyze the impact of the use of non-invasive ventilation in malaria with pulmonary dysfunction.

**Methods:** It’s a retrospective cohort study. We analyzed electronic records of patients who were diagnosed with malaria, with acute respiratory failure, who underwent respiratory therapy with NIV between 2015-2016 within the intensive care unit (ICU). The study variables were: ICU mortality, length of hospital stay, NIV time and outcome groups. Statistical analysis was performed with the Pearson correlation coefficient, with significance level of p <0.01. The statistics were performed using the BioEstat 3.0 program.

**Results:** Thirty-one patients were included in the study. Four results were analyzed according to Table 1 and Fig. 1. 94% of the patients were discharged from the hospital. Pearson’s correlation coefficient analysis showed statistical significance in the Group (NIV/Discharge) in the analysis of patients hospitalized versus NIV (95% CI = 0.24 to 0.83 <(p) = 0.0036).

**Conclusions:** The use of NIV was positive in patients using this resource as first-line treatment of malaria in the fight against respiratory decompensation, with improvement of symptoms.

**Table 1 (abstract P184).** Serial analysis of intervention groups

Outcomes	Number of patients*	Days of hospitalization*	Days of NIV*	Time of NIV (minutes)*
NIV/Intubation/Death	2	11	1	75
NIV/Discharge	20	9.8	3.9	57.3
Intubation/NIV/Dischargeb	1	23	7	60
NIV/Intubation/NIV/Discharge	8	16.1	5.1	75

*average

**Fig. 1 (abstract P184).**
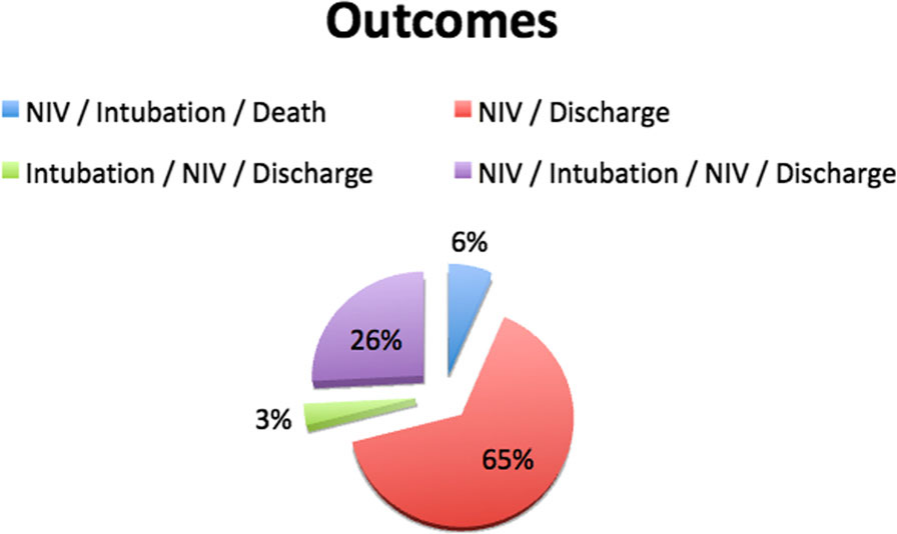
Outcome groups.

## P185 Factors predicting postoperative noninvasive ventilation failure in a tertiary care university hospital: a retrospective study

### S Chatmongkolchart, B Choochot

#### Faculty of Medicine, Prince of Songkla University, Songkla, Thailand

**Introduction:** Noninvasive ventilation (NIV) is a therapeutic choice in the management of acute respiratory failure in the postoperative period. NIV failure is associated with poor outcomes and increased mortality. The aim of this study is to determine the associated factors of NIV failure in the postoperative period.

**Methods:** Data was obtained from a total of 206 surgical patients who experienced postoperative respiratory complications and were managed by NIV between January 2012 and December 2016 at surgical intensive care unit in Songklanagarind hospital. Predictive factors for NIV failure were determined using multivariate logistic regression models.

**Results:** The incidence of postoperative NIV failure was 39.3%. In multivariate analysis, factors independently predicting NIV failure were smoking (odds ratio (OR) 3.24, 95% confidence interval (CI) 1.63-6.44), independent functional status (OR 2.05, 95% CI 1.05-4.03), American Society of Anesthesiologists (ASA) status 4-5 vs 2-3 (OeR 2.72, 95% CI 1.36-5.46), and intracranial surgery (OR 2.66, 95% CI 1.19-5.96) (Table 1). NIV failure was also associated with a higher incidence of postoperative pulmonary complication such as pneumonia (81.2% vs 18.8%, P <0.001), prolonged hospital stay (41.2±35.5 vs 26.1±21.0 days, P <0.001) and cardiac arrest (88.2% vs 11.8%, P = 0.004) comparing with NIV success.

**Conclusions:** Pre-and perioperative predictors of NIV failure were smoking, independent functional status, ASA status 4-5, and intracranial surgery. Management of NIV in patients at risk of failure requires close monitoring because of the failure of NIV may be associated with higher risk of postoperative complications.

**Table 1 (abstract P185).** Final logistic regression model of factors predicting postoperative noninvasive ventilation failure

Predicting factor	Adjusted OR (95% CI)	P-value (Wald’s test)
Smoking	3.24 (1.63,6.44)	<0.001
Functional status: Independent vs Dependent	2.05 (1.05,4.03)	0.036
ASA status: 4-5 vs 2-3	2.72 (1.36,5.46)	0.005
Intracranial surgery	2.66 (1.19,5.96)	0.017

## P186 Noninvasive ventilation (NIV) failure in critically ill patients with COPD and influenza infection: risk factors and outcomes

### C Domínguez-Curell^1^, A Rodríguez^1^, L Reyes^2^, M Bodi^1^, A Esteban^3^, F Gordo^3^, I Martín-Loeches^3^, J Solé-Violán^4^, E Díaz^5^, A Anzueto^2^, M Restrepo^2^

#### ^1^Hospital Universitari de Tarragona Joan XXIII, Tarragona, Spain; ^2^University of Texas Health Science Center at San Antonio and South Texas Veterans Health Care System, San Antonio, Texas, USA; ^3^Multidisciplinary Intensive Care Research Organization (MICRO), St.James’s University Hospital, Trinity Centre for Health Science, Dublin, Ireland; ^4^Hospital Dr. Negrín, Gran Canaria, Las Palmas, Spain; ^5^Hospital Parc Tauli, CIBERES, Sabadell, Spain

**Introduction:** The effectiveness of NIV in COPD patients with acute respiratory failure (ARF) due to influenza infection remains controversial. The aim of this study was to characterize COPD patients at risk to NIV failure (NIVf) and their impact on ICU mortality.

**Methods:** Secondary analysis from a prospective, observational, multi-center study of COPD subjects admitted to the ICU with ARF due to influenza infection. Demographics data, clinical and laboratory variables and severity of illness were recorded. Three groups were studied: (1) COPD subjects who received NIV at ICU admission and failed(NIVf group); (2) COPD subjects who received NIV at ICU admission and succeeded(NIVs group); and (3) COPD subjects who received invasive mechanical ventilation at ICU admission(IMV group). Univariate and multivariate analysis was performed to determine factors independently associated with NIVf and ICU mortality.

**Results:** Of 476 patients, 211 (44.3%) required IMV and 265 (55.6%) NVI. Failure occurred in 118 (44.5%) patients and were more likely to have high severity(SOFA 7 vs.4,p<0.001),shock(72.9%vs.13.6%,p<0.05),acute renal failure (31.4%vs. 13.6%, p<0.05), bacterial co-infection(25.4% vs. 10.9%,p<0.05) and hematological disease (5.9% vs. 1.4%,p<0.05) compared to NIVs group. Shock (OR=13.4[6.36-28.8]) was independently associated with NIVf. The overall ICU mortality was 31% in the IMV group, 39% in the NIVf group and 5% in the NIVs group, respectively (p<0.001 comparing NIV groups). In the multivariate analysis, only number of quadrants infiltrates(OR=1.42[1.03-1.94]p<0.02), hematological disease (OR=6.27[1.09-35.9]p=0.04), chronic renal failure (OR=7.3[2.1-24.4]p=0.02) and NIVf (OR=9,27[2.9-28.7]p<0.001) were variables independently associated with ICU mortality.

**Conclusions:** NIVf is frequent a complication in COPD patients admitted to the ICU with influenza infection. Shock presence should alert clinicians to consider IMV due to the high risk of NIV failure and ICU mortality.

## P187 Non-invasive ventilation and oxygen therapy after extubation in patients with acute respiratory failure: a meta-analysis of randomized controlled trials

### S Bhattacharjee, S Maitra

#### All India Institute of Medical Sciences, New Delhi, New Delhi, India

**Introduction:** Role of non-invasive ventilation (NIV) following extubation in patients with acute respiratory failure is debatable. NIV may provide benefit in post surgical patients [1], but its role in non-surgical patients is controversial.

**Methods:** PubMed and Cochrane Central Register of Controlled Trials (CENTRAL) were searched (from 1946 to 20th November 2017) to identify prospective randomized controlled trials, where post extubation NIV has been compared with standard oxygen therapy in adult patients with acute respiratory failure. For continuous variables, a mean difference was computed at the study level, and a weighted mean difference (MD) was computed in order to pool the results across all studies. For binary outcomes, the pooled odds ratio (OR) with 95% confidence interval (95% CI) was calculated using the inverse variance method.

**Results:** Data of 1525 patients from 11 randomized trials have been included in this meta-analysis. Two trials used NIV to manage post-extubation respiratory failure. Pooled analysis found that mortality rate at longest available follow-up [OR (95% CI) 0.84 (0.50, 1.42); p=0.52; Fig. 1] and re-intubation rate [OR (95% CI) 0.75 (0.51, 1.09); p=0.13] were similar between NIV and standard oxygen therapy. NIV did not decrease intubation rate when used as preventive modality [OR (95% CI) 0.65 (0.40, 1.06); p=0.08]. Duration of ICU stay was also similar in the two groups [MD (95% CI) 0.46 (-0.43, 1.36) days; p=0.31; Fig. 2].

**Conclusions:** Post extubation NIV in non- surgical patients with acute respiratory failure does not provide any benefit over conventional oxygen therapy.

**Reference**

Faria DA, et al. Cochrane Database Syst Rev CD009134, 2015.

**Fig. 1 (abstract P187).**
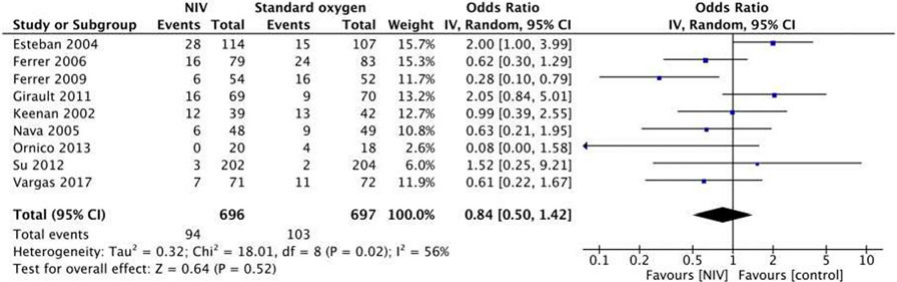
Forest plot for pooled analysis of mortality at longest available follow-up

**Fig. 2 (abstract P187).**
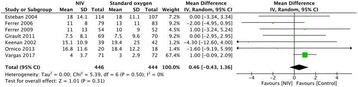
Forest plot for pooled analysis of need for re-intubation

## P188 A randomized comparative study of helmet CPAP versus facemask CPAP in acute respiratory failure (ARF)

### O Adi, S Salleh

#### Raja Permaisuri Bainun Hospital, Ipoh, Perak, Malaysia

**Introduction:** CPAP is used to improve oxygenation in patient with ARF. We aimed to determine non-inferiority (NI) of helmet CPAP to facemask in ARF based on physiological (heart rate (HR) and respiratory rate (RR)) and blood gas parameters (PaO2 and PaCO2). We also compared patients’ perception in dyspnea improvement after CPAP using dyspnea scale (visual analogue scale (VAS)) and Likert score.

**Methods:** We randomized 123 patients to helmet (n=64) and facemask (n=59) with 71.7% of ARF was due to acute pulmonary edema. CPAP was applied for 60 minutes. Patients’ physiological and blood gas parameters were recorded before and after intervention. Patients then marked on dyspnea scale and Likert score. NI of helmet would be declared if confidence interval (CI) of mean difference between groups (helmet’s mean minus facemask’s mean) in improving physiological, blood gas parameters and dyspnea scale was no worse than predetermined non-inferiority margin (NIM). Secondary outcome was to compare incidence of discomfort and mucosal dryness between groups.

**Results:** Both intention to treat and per protocol (PP) analysis showed mean difference for HR, RR and dyspnea scale were above NIM thus conclude helmet (NI) to facemask. PP analysis of mean differences for HR, RR and dyspnea scale as followed: HR mean difference was -4.43 beats per minute (upper bound 97.5% CI 1.43), mean difference of RR was -0.41 breaths per minute (upper bound of 97.5% CI 0.48) and mean difference of dyspnea scale was 10.98mm (lower bound 97.5% CI 1.94) (Fig. 1) . Both PaO2 and PaCO2 level improved in helmet, but it was inferior. Analysis of Likert score for dyspnea improvement also conclude helmet was better than facemask (mean rank 67.81 vs 55.69, p=0.04). Incidence of discomfort and mucosal dryness was significantly lower with helmet.

**Conclusions:** CPAP delivered by helmet improves HR, RR and dyspnea in ARF with less discomfort and dryness of mucosa and it is the alternative for patients who are unable to tolerate facemask.

**Fig. 1 (abstract P188).**
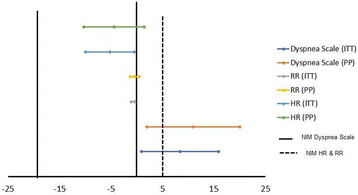
Mean difference between helmet and facemask for heart rate, respiratory rate and dyspnea scale

## P189 Effects of varying ventilatory parameters on FiO2 delivery of a boussignac CPAP in a mechanical model of acute respiratory distress

### M Bibombe^1^, A Penaloza Baeza^2^, G Liistro^2^, N Delvau^2^

#### ^1^Université Catholique de Louvain, Brussels, Belgium; ^2^Cliniques Universitaires St-Luc, Université Catholique de Louvain, Brussels, Belgium

**Introduction:** Acute cardiogenic pulmonary edema is a sudden onset respiratory distress. Its management includes, drug therapy, oxygen and airway support by spontaneous ventilation in continuous positive airway pressure (CPAP). The Boussignac CPAP is powered by pure oxygen, its ‘open’ character allows the patient to increase his inspiratory demand himself, which may influence the delivered FiO2, the patient also breathing ambient air with 21% O2. Previous studies assessed the FiO2 delivered by the device under conditions of respiratory distress but did not focuse on insipratory flow. The aim of this study was to measure the FiO2 actually delivered by the device under simulated conditions of respiratory distress.

**Methods:** In this benchmark study, FiO2 was measured by varying the respiratory rate (FR, 10 up to 45/min), tidal volume (Vt, between 150 and 750mL) and inspiratory flow (between 30 and 90L/min) at the target pressure of 8 cmH2 O. The assembly included a Boussignac CPAP fed with 100% O2 via a flowmeter up to 30 L/min, a double Vygon mask sealed, a Vygon manometer, a Michigan test lung driven by a Dräger Evita 4 ventilator, and a Citrex analyzer. Each measurement was done 3 times and the average of the 3 values was retained.

**Results:** The O2 flow required to maintain the target pressure of 8cmH20 was <= 25L/min. Depending on FR and inspiratory flow, for a Vt <= 250mL, the delivered FiO2 ranged between 70 and 99%. For a 350mL <= Vt <= 500mL, the FiO2 was between 57 and 90%. For a given Vt, FiO2 decreased when FR and/or inspiratory flow were increased (Fig. 1).

**Conclusions:** The Boussignac CPAP delivered high values of FiO2 at a flow rate of O2 <= 30L/min. However, FiO2 varied with FR, Vt and inspiratory flow. These changes in FiO2 observed during simulated severe respiratory distress conditions should be taken into account and compared to other CPAP devices.

**References**

Templier F et al. Eur J Emerg Med 10(2):87-93, 2003

**Fig. 1 (abstract P189).**
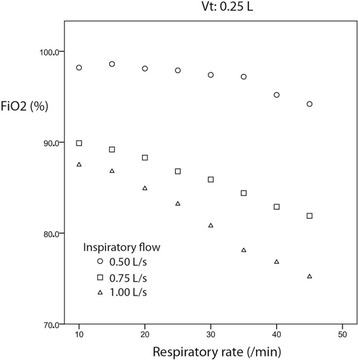
Effect of varying RR and inspiratory flow on FiO2 at a Vt of 0.250 L

## P190 Recurrent respiratory deterioration events during intensive care unit stay and mortality among mechanical ventilated patients

### D Stavi^1^, Y Lichter^1^, H Artsi^1^, U Keler^2^, AM Lipsky^3^, N Adi^1^, I Matot^1^

#### ^1^Tel Aviv Sourasky Medical Center, Tel Aviv, Israel; ^2^Intensix, Netanya, Israel; ^3^Rambam Health Care Campus, Haifa, Israel

**Introduction:** Recurrent respiratory deteriorations in Mechanically Ventilated (MV) patients may occur during Intensive Care Unit (ICU) stay. Knowledge of the association of such events and mortality is limited.

**Methods:** This is a single center retrospective study performed in the ICU of Tel Aviv Medical Center, Israel, a tertiary academic referral hospital. Using the electronic medical record system and Intensix predictive critical care system for analysis, all patients admitted to the ICU between 1.2007 and 12.2014 were assessed. Respiratory deterioration in MV patients was defined as acute adjustment of FiO2 increase >20% or PEEP increase > 5 cmH2O that persisted for at least 2 hours. The primary outcome was ICU mortality. Secondary outcome was length of ICU stay (LOS). A Chi square test for trends was used for the significance of mortality data and a one way ANOVA test for LOS.

**Results:** 5376 MV patients were admitted to the ICU with an overall mortality of 16.5%. Mortality and LOS were tripled in patients who experienced at least one respiratory deterioration when compared to no events (33.8% vs. 9.9 %, p<0.0001 and 10.7 vs. 2.2 days, p<0.0001 respectively) (Fig. 1). Increased events of respiratory deteriorations showed significant trend of increased mortality (p<0.0001).

**Conclusions:** In MV patients, a single respiratory deterioration event carries a 3 times higher mortality rate and Length Of Stay (LOS). Any additional event further increases both parameters.

**Table 1 (abstract P190).** Results Table

No. of Deteriorations	No. of Patients	Age – mean (+/-SD)	ICU mortality (%)	Median LOS days (interquartile range)
0	3890	59.4 (20.6)	385 (9.9)	2.2 (0.8-5.1)
1	764	59.2 (19.4)	226 (29.6)	7.4 (3.3-13.3)
2	328	58.6 (19.1)	122 (37.2)	10.6 (5.5-16.7)
3	166	60.1 (17.2)	60 (36.1)	15.4 (8.9-22.6)
>=4	228	56.4 (19.3)	94 (41.2)	21.3 (13.9-30.6)

## P191 Association of lung ultrasound score with mortality in mechanically ventilated patients

### J Taculod, JT Sahagun, Y Tan, V Ong, K See

#### National University Hospital Singapore, Singapore, Singapore

**Introduction:** Lung ultrasound is an important part of the evaluation of critically ill patients. It has been shown to predict recruitability in acute respiratory distress syndrome. However, little is known about the application of lung ultrasound in predicting mortality in mechanically ventilated patients.

**Methods:** Observational study of mechanically ventilated patients admitted to the medical intensive care unit (ICU) of a tertiary hospital (National University Hospital, Singapore) in 2015 and 2016. Only the first ICU admissions of these patients were studied. Lung ultrasound was done at six points per hemithorax and scored according to Soummer (Crit Care Med 2012): normal aeration = 0; multiple, well-defined B lines =1; multiple coalescent B lines = 2; lung consolidation = 3. The Lung Ultrasound (LUS) score was calculated as the sum of points (score range 0-36). We analysed the association of LUS score with ICU/hospital mortality, using logistic regression, adjusted for age and Acute Physiology and Chronic Health Evaluation (APACHE) II score.

**Results:** 247 patients were included (age 62.0 ± 16.2 years; 89 female [36.0%]; APACHE II 29.7 ± 7.9; 88 sepsis diagnosis [35.6%]). ICU and hospital mortality were 16.2% and 29.6% respectively. LUS score was associated with increased ICU (OR 1.04, 95% CI 1.00-1.09, P=0.07) and hospital (OR 1.04, 95% CI 1.00-1.08, P=0.045) mortality, adjusted for age and APACHE II score.

**Conclusions:** LUS score was associated with increased ICU/hospital mortality and may be useful for risk stratification of mechanically ventilated patients admitted to ICU.

## P192 A survey on mechanical ventilator practice and synchrony management in Thailand

### N Kongpolprom

#### King Chulalongkorn Memorial Hospital, Bangkok, Thailand

**Introduction:** Ventilator asynchrony results in morbidities and mortality. The aim of this study was to explore whether and how physicians used patient-ventilator interactions(PVI) to set mechanical ventilators(MV) in Thailand.

**Methods:** Thai physicians treating MV patients were asked to respond to questionnaires distributed in conferences and to e-mails sent. Types of asynchronies encountered and frequency of MV adjustment guided by PVI were evaluated. In addition, correlations between physician’s knowledge and 1)confidence to manage asynchronies and 2)their experience were analyzed.

**Results:** Two hundred and eleven physicians answered the questionnaires. Most of them were medical residents and ICU specialists. 82% of them set and adjusted MV by asynchrony guidance and the majority used waveform analysis to more than a half of their patients. The most and the least common asynchronies encountered were double triggering and reverse triggering, respectively, while the most difficult-to-manage and the most easily managed asynchronies were periodic/?A3B2 show $132#?>unstable breathing and flow starvation, respectively. Lack of confidence and knowledge of PVI were the major reasons of physicians who did not perform asynchrony assessment. For knowledge evaluation, more than 50% of physicians incorrectly managed asynchrony. Chest and ICU fellows had the greatest skills in waveform interpretation and asynchrony management with the mean score of 2.62 from the total 5, compared with specialist(2.58), medical residents(1.85), internists(1.84) and general practitioner(0.85). There were poor correlations between years’ experience in MV management and the skill in waveform interpretation (r = 0.15, p=0.034) and between physician’s confidence in PVI management and the clinical skill (r = 0.27, p<0.001)

**Conclusions:** The majority of Thai physicians realized the importance of PVI, but the skill in asynchrony management was moderate. Intensive programs should be provided to improve their clinical performance.

## P193 Single step versus intermittent evacuation of simple pleural effusion in ventilated patients

### N Makhoul

#### Galilee Medical Center, Naharia, Israel

**Introduction:** Early and expeditious evacuation of simple pleural effusion (SPE) in ventilated patients may improve their respiratory condition. Employment of ultrasonography (US) and pigtail catheters for SPE management has shown beneficial results and widely accepted. Nevertheless, methods of SPE evacuation are still under discussion. Objective of the study is to evaluate efficacy and safety of single step versus intermittent SPE evacuation in ventilated patients.

**Methods:** Our retrospective study included 81 adult ventilated ICU patients with SPE from 05.2015 to 08.2017. US has been used for diagnosis and navigation of pigtail catheters insertion. In the a-group (40 patients) SPE evacuation was done by single step during one hour. In the b-group (41 patients) SPE evacuation was performed intermittently for 24 hours as usually recommended. In both groups SPE was evacuated by gravitation method. The outcomes of intervention were evaluated by chest US and mobile x-ray film.

**Results:** Of 81 patients (52 males, 29 females; mean age 68,9) the main causes of SPE were: congestive heart failure (30%), pneumonia (22%), and malignancy (20%). The median evacuated volume was 781 ml (range 300- 2400 ml). Catheters were removed if minimal fluid discharge (<100 ml) has been observed during following 48 hours in a-group, and 72 hours in b-group. In both groups US and chest x-ray demonstrated re-expansion of compressed lung. There were no complications in either group.

**Conclusions:** Single step evacuation provided safe, less time-consuming management of any SPE volume with the same efficacy in comparison to intermittent technique in ventilated patients.

## P194 Positional effects on the distributions of ventilation and end-expiratory gas volume in the asymmetric chest –a quantitative lung computed tomographic analysis

### GA Cortes-Puentes^1^, K Gard^2^, A Adams^2^, D Dries^3^, R Oeckler^1^, M Quintel^4^, L Gattinoni^4^, JJ Marini^3^

#### ^1^Mayo Clinic, Rochester, MN, USA; ^2^Regions Hospital, Saint Paul, MN, USA; ^3^University of Minnesota, Minneapolis, MN, USA; ^4^University of Göttingen, Göttingen, Germany

**Introduction:** Body positioning affects the configuration and dynamic properties of the chest wall and therefore may influence decisions made to increase or decrease ventilating pressures and tidal volume. We hypothesized that unlike global functional residual capacity (FRC), component sector gas volumes and their corresponding regional tidal expansions would vary markedly in the setting of unilateral pleural effusion (PLEF), owing to shifting distributions of aeration and collapse as posture changed.

**Methods:** Six deeply anesthetized swine underwent tracheostomy, thoracostomy and experimental PLEF with 10 ml/kg of radiopaque saline randomly instilled into either pleural space. Animals were ventilated at VT=10ml/kg, frequency=15bpm, I/E=1:2, PEEP=1cmH2O, and FIO2=0.5. Quantitative lung computed tomographic (CT) analysis of regional aeration and global FRC measurements by nitrogen wash-in/wash-out technique were performed in each of these randomly applied positions: semi-Fowler’s (inclined 30° from horizontal in the sagittal plane); prone, supine, and lateral positions with dependent PLEF and non-dependent PLEF (Fig. 1).

**Results:** No significant differences in FRC were observed among the horizontal positions, either at baseline (p=0.9037) or with PLEF (p=0.58) (Fig. 2A). However, component sector total gas volume in each phase of the tidal cycle were different within all studied positions with and without PLEF (p=<.01). Compared to other positions, prone and lateral position with non-dependent PLEF had a more homogenous VT distribution among quadrants (p=.051, Fig. 2B). Supine was associated with most dependent collapse (Fig. 2C) and greatest tendency for tidal recruitment (48% vs ~22%, p=0.0073, Fig. 2D).

**Conclusions:** Changes in body position in the setting of effusion-caused chest asymmetry markedly affected the internal distributions of gas volume, collapse, ventilation, and tidal recruitment, even when commonly used global FRC measurements provided little indication of these important positional changes.

**Fig. 1 (abstract P194).**
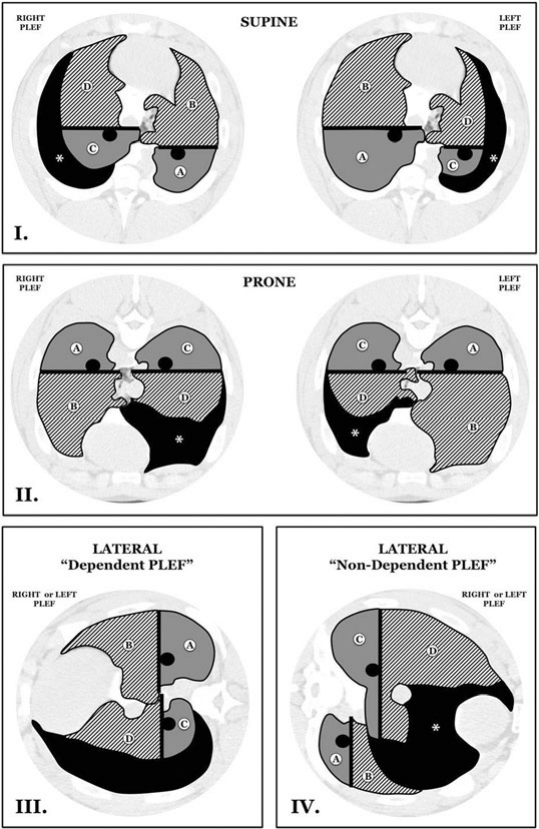
Nomenclature For Analysis of Regional Aeration: I. Supine and II. Prone, where quadrants were defined as: Non-PLEF Dorsal (A); Non-PLEF Ventral (B); PLEF Dorsal (C); and PLEF Ventral (D). III. Lateral position with “Dependent Pleural Effusion”, where quadrants were defined as: Non-PLEF, Non-Dependent Dorsal (A); Non-PLEF, Non-Dependent Ventral (B); PLEF Dependent Dorsal (C); and PLEF Dependent Ventral (D). IV. Lateral position with “Non-Dependent Pleural Effusion”, where quadrants were defined as: Non-PLEF, Dependent Dorsal (A); Non-PLEF, Dependent Ventral (B); PLEF, Non-Dependent Dorsal (C); and PLEF Non-Dependent Ventral (D). PLEF: Pleural Effusion, *: anatomical distribution of PLEF

**Fig. 2 (abstract P194).**
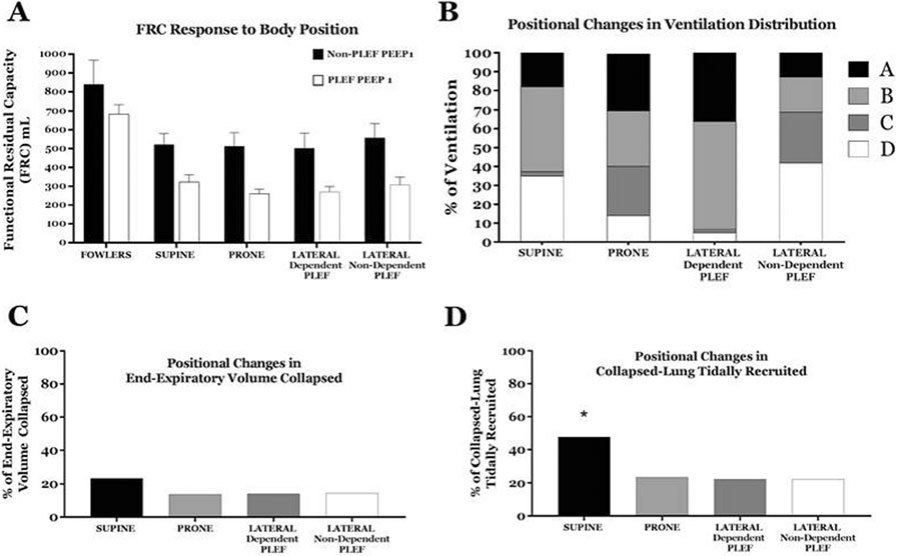
A. Global FRC response to body position; B. Distribution of tidal ventilation; C. Positional Changes in End-Expiratory Collapsed Volume; D. Positional Changes in Tidal Recruitment

## P195 Intensive care unit doctors’ self-reported preferences for arterial oxygen tensions in mechanically ventilated patients

### OL Schjørring^1^, AP Toft-Petersen^2^, KH Kusk^1^, EE Sørensen^1^, P Mouncey^2^, A Perner^3^, J Wetterslev^3^, BS Rasmussen^1^

#### ^1^Aalborg University Hospital, Aalborg, Denmark; ^2^Intensive Care National Audit & Research Centre (ICNARC), London, UK; ^3^Rigshospitalet, Copenhagen University Hospital, Copenhagen, Denmark

**Introduction:** Optimal oxygenation targets in critically ill patients treated in the intensive care unit (ICU) are not established. As high oxygen levels may be harmful, doctor’ preferences for oxygen administration in mechanically ventilated patients are of significant interest as these have the largest exposure to high fractions of inspired oxygen (FiO_2_). To quantify a multinational segment of ICU doctors’ preferences for oxygen administration in a variety of mechanically ventilated ICU patients, we conducted an international questionnaire-based survey.

**Methods:** Based on a previous survey [1] we constructed and validated a 17-part questionnaire on oxygen administration. The questionnaire was electronically distributed through spring and summer 2016 to 1080 ICU doctors at various educational levels in 233 hospitals in Denmark, Finland, Norway, Sweden, England, Wales and Northern Ireland. Repeated reminders were sent.

**Results:** In total, 681 doctors responded yielding a response rate of 63%. Of the respondents, 80% were affiliated with multidisciplinary ICUs, 14% with thoracic and/or cardiac ICUs and 6% with neuro-ICUs. Most respondents (79%) had completed their specialist training. Overall, arterial oxygen tension (PaO_2_) was the preferred parameter for the evaluation of oxygenation (Fig. 1). The proportions of doctors’ preferences for increasing, decreasing or not changing an FiO_2_ of 0.50 in two (out of six) patient categories at different PaO_2_ levels are presented in Table 1 and Table 2.

**Conclusions:** This is the largest survey of the preferred oxygenation targets among ICU doctors. PaO_2_ seems to be the preferred parameter for evaluating oxygenation. The characterisation of PaO_2_ target levels in various clinical scenarios provide valuable information for future clinical trials on oxygenation targets in critically ill ICU patients.

**Reference**

1. Mao C et al. Crit Care Med 27(12):2806-2811, 1999

**Table 1 (abstract P195).** Doctors’ preferences for adjusting an FiO2 of 0.50 in a patient with acute respiratory distress syndrome (n = 654 to 655)

PaO_2_	Increase	No change	Decrease
8 kPa	53% (49%-56%)	47% (43%-51%)	0% (0%-1%)
10 kPa	2% (1%-3%)	73% (70%-76%)*	25% (22%-28%)
12 kPa	0% (0%-1%)	26% (23%-29%)*	74% (71%-77%)
14 kPa	0% (0%-0%)	3% (2%-4%)*	97% (96%-98%)

The scenario involves a patient expected to receive mechanical ventilation for at least 24 hours in the ICU. All proportions are percentages of respondents with 95% confidence intervals. *p < 0.001 for comparisons of proportions of “No change” versus adjacent lower PaO2 level (McNemar’s test)

**Table 2 (abstract P195).** Doctors’ preferences for adjusting an FiO2 of 0.50 in a patient with chronic obstructive pulmonary disease with known habitual hypercapnia (n = 649)

PaO_2_	Increase	No change	Decrease
8 kPa	11% (9%-14%)	85% (82%-87%)	4% (3%-6%)
10 kPa	1% (0%-2%)	38% (34%-42%)*	61% (58%-65%)
12 kPa	0% (0%-1%)	4% (3%-6%)*	95%(93%-96%)
14 kPa	0% (0%-1%)	1% (0%-2%)*	99% (97%-99%)

The scenario involves a patient expected to receive mechanical ventilation for at least 24 hours in the ICU. All proportions are percentages of respondents with 95% confidence intervals. *p < 0.001 for comparisons of proportions of “No change” versus adjacent lower PaO2 level (McNemar’s test)

**Fig. 1 (abstract P195).**
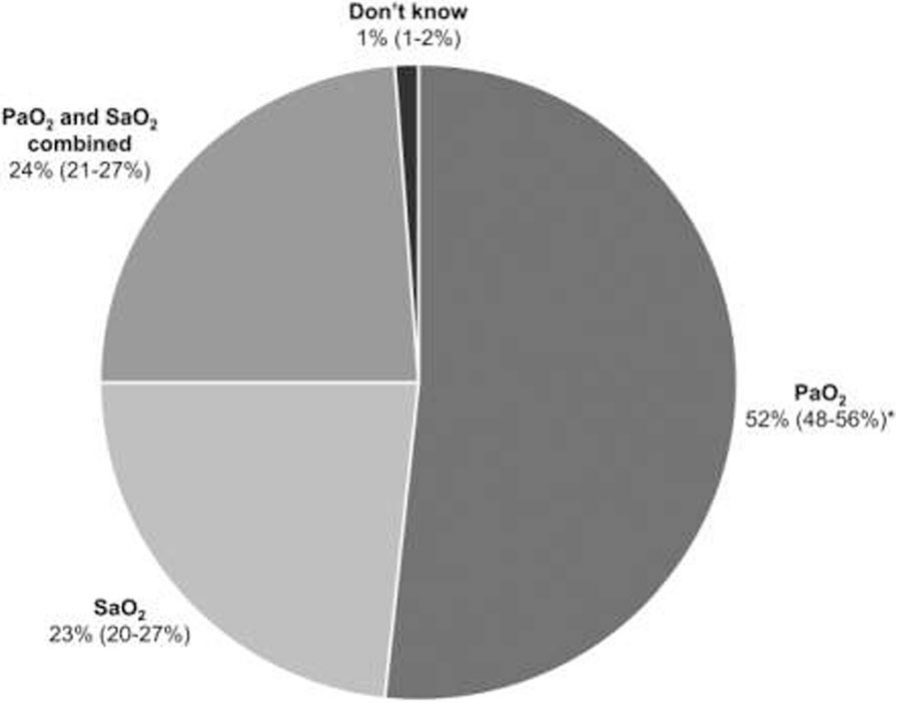
Preferred parameter in the evaluation of oxygenation (n = 677). *Most doctors preferred PaO2 in their evaluation of oxygenation in mechanically ventilated patients (p < 0.001, simple binomial test against the equally distributed proportion)

## P196 Diaphragm ultrasound to predict weaning outcomes in mechanically ventilated patients

### S Abdallah, A Ben Souissi, W Yaakoubi, S Kamoun, I Ben Naoui, MS Mebazaa

#### Mongi Slim university Hospital, Tunis, Tunisia

**Introduction:** Sonographic assessment of diaphragmatic excursion and muscle thickening fraction have been suggested to evaluate diaphragm function during weaning trial [1]. The purpose of this study is to compare these two parameters to predict extubation success.

**Methods:** This prospective study was carried out during 9 months from March to November 2017. We enrolled patients who were mechanically ventilated for more than 48h and met all criteria for extubation. The non inclusion criteria were: age < 18 years, history of neuromuscular disease or severe chronic respiratory failure. We excluded subjects who needed reintubation for upper airway obstruction, neurological or hemodynamic alteration. The US exam was performed during spontaneous breathing test or pressure support trial, measuring both diaphragm excursion (DE) and diaphragmatic thickening fraction (DTF) within 24 hours before extubation

**Results:** Among 36 enrolled patients, four were excluded and 78.1% were successfully extubated, whereas 21.9% needed reintubation or noninvasive ventilation within 48h from extubation. The median degree of DE was higher in patients with extubation success than those with failure (17.8 mm vs. 11.9 mm, p=0.01). Patients with extubation success had a greater DTF (41.7% vs. 29.7%, p = 0.007). The area under the ROC for DE was 0.800, 95% CI [0.578-1], while it was 0.717 for DTF 95% CI [0.453-0.981], p=0.001. Cut off values associated with successful extubation were 12 mm for DE and 31% for DTF giving respectively 84% and 96% of sensibility, 71% and 57% of specificity, 7.6 and 9.7 of likelihood ratio. Combining both parameters decreased sensibility to 79% but increased specificity to 100%.

**Conclusions:** US may be a valuable tool in the evaluation of diaphragm dysfunction. The diaphragm excursion seems more accurate than the diaphragm thickening fraction to predict extubation success

**References**

Mayo P et al. Intensive Care Med 42:7, 2016

## P197 Impact of adhesion of ventilatory weaning protocol on the incidence rate of pneumonia associated with mechanical ventilation in the tracheostomized patient

### P Travassos^1^, E Teixeira^2^, L Freitas^2^, P Darin^2^, L Junqueira^2^, T Kawagoe^2^, N Postalli^2^, R Vale^2^, V Veiga^2^, S Rojas^2^

#### ^1^Sao Paulo, Sao Paulo, Brazil; ^2^Hospital BP - A Beneficência Portuguesa de São Paulo, São Paulo, Brazil

**Introduction:** Ventilatory weaning protocols are important for the reduction of pneumonia associated with mechanical ventilation in tracheostomized patients. The objective of this study was to evaluate the impact of the adhesion of the ventilatory weaning protocol on the incidence rate of pneumonia associated with mechanical ventilation in the tracheostomized patient in a large hospital neurological intensive care unit.

**Methods:** The tracheostomized patients were retrospectively assessed from January 2015 to May 2017, correlating time of ventilatory weaning and pneumonia associated with mechanical ventilation.

**Results:** In the period, 8,485 patients were admitted to the unit, with a mean age of 66.5 years, with an average stay of 5.6 days; 56% of the hospitalizations were surgical, with an expected mortality of SAPS3 of 22.3% and real mortality of 11.2%. In this group, 497 were tracheostomized patients and 276 eligible for ventilatory weaning according to institutional protocol. Ninety-six percent of patients completed ventilatory weaning. Prior to protocol initiation, the mean ventilatory weaning time was 12.8 days, which decreased to 5.2 days in 2015 and 1.6 days in 2016. The incidence rate of ventilator-associated pneumonia was 1.09 by 2015; 1.49 in 2016 and 0.75 in 2017.

**Conclusions:** The implementation of ventilatory weaning protocol contributed safely to standardization of the weaning process in the unit, reduction of mechanical ventilation time and low rate of pneumonia associated with mechanical ventilation.

## P198 Evaluation of diaphragmatic thickness with ultrasound in patients undergoing controlled and assisted mechanical ventilation

### C Abbruzzese ^1^, D Ferlicca^2^, S Francesconi^2^, V Ormas^3^, E Lupieri^3^, S Calcinati^4^, S Cenci^3^, E Chiodaroli^5^, A Facchini^2^, A Pesenti^1^, G Foti^4^, G Bellani^3^

#### ^1^Fondazione IRCCS Ca’ Granda Ospedale Maggiore Policlinico, Milano, Italy; ^2^ASST Monza, Desio Hospital, Desio, Italy; ^3^University of Milano-Bicocca, Monza, Italy; ^4^ASST Monza, San Gerardo Hospital, Monza, Italy; ^5^Università degli Studi di Milano, Milan, Italy

**Introduction:** Ventilator Induced Diaphragmatic Dysfunction is known to be a contributor to weaning failure. Some data suggest that assisted ventilation might protect from diaphragmatic thinning. Aims of this study are to evaluate, by ultrasound (US), the change in diaphragm thickness and thickening in patients undergoing controlled and assisted mechanical ventilation (MV) and clinical factors associated with this change.

**Methods:** We enrolled patients who underwent either controlled MV (CMV) for 48 cumulative hours or 48 hours of pressure support (PSV) if ventilation was expected to last for at least 5 days. Patients < 18 years old, with neuromuscular diseases, phrenic nerve injury, abdominal vacuum dressing system and poor acoustic window were excluded. Diaphragm thickness and thickening were measured with US as described by Goligher and clinical data were collected every 48 hours until ICU discharge.

**Results:** We enrolled 44 patients, 13 were excluded because they had less than 4 measurements and 2 for low quality images, leaving 29 patients for analysis. As expected, during CMV diaphragm thickening was almost absent and significantly lower than during PSV (p<0,001). Diaphragm thickness did not reduce significantly during CMV (p=0.201), but during PSV significantly increased (p<0.048) (Fig. 1, where “day 0” represents the first day of PSV). During CMV, in 10/21 patients diaphragm thickness showed a >=10% reduction. They had a significantly higher fraction of days spent in CMV (p=0.005) and longer neuromuscular blocking drugs (NBDs) infusion (p=0.043). During PSV, 17/26 patients showed an increase in diaphragm thickness >=10%. Duration of hospital stay was significantly lower for these patients (p 0.048). Differences between the two groups are reported in Table 1.

**Conclusions:** Longer time spent in CMV and with NBDs infusion seems associated with a decrease in diaphragm thickness. Assisted ventilation promotes an increase in diaphragm thickness, associated with a reduction in the length of hospitalization.

**Table 1 (abstract P198).** Characteristics of patient subgroups according to increase in diaphragm thickness during PSV

	>=10% increase in diaphragm thickness (n=17)	<10% increase in diaphragm thickness (n=9)	P value
Age, mean (SD)	59 (10)	59 (13)	0,973
SOFA score at baseline, median (IQR)	7 (6-9)	9 (6-10)	0,181
Tidal Volume, ml/kg mean (SD)	7 (2)	7 (2)	0,977
Pressure Support, cmH2O median (IQR)	8 (7-9)	8 (7-10)	0,598
PEEP, cmH2O mean (SD)	8 (2)	10 (2)	0,04
Days in hospital for survivors, median (IQR)	36 (24-44)	62 (45-81)	0,048
ICU outcome, n death (%)	1 (6)	3 (33)	0,116

**Fig. 1 (abstract P198).**
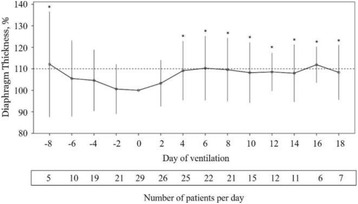
Changes in diaphragm thickness over time. Day “0” is the first day of PSV; days before “0” are days of CMV, days after “0” are days of PSV. Dashed line represents 10% cut-off value selected a priori to define clinically relevant increases in diaphragm thickness. *: p value < 0,05 versus day 0.

## P199 Prediction of intrinsic positive end-expiratory pressure using diaphragmatic electrical activity in neutrally-triggered and pneumatically-triggered pressure support

### F Xia

#### Nanjing Zhongda Hospital, Southeast University, Nanjing, China

**Introduction:** Intrinsic positive end-expiratory pressure (PEEPi) may substantially increase the inspiratory effort during assisted mechanical ventilation. Our purpose of the study was to assess whether electrical activity of the diaphragm (EAdi) can be reliably used to estimate PEEPi in patients undergoing conventional pneumatically-controlled pressure support (PSP) ventilation and neutrally-controlled pressure support (PSN) and whether PSN was beneficial in comparison with PSP in patients affected by PEEPi.

**Methods:** Twelve intubated and mechanically ventilated COPD patients with static PEEPi>=5cm H2O underwent PSP and PSN at different levels of extrinsic PEEP (PEEPe) (at 0%, 40%, 80%, and 120% of static PEEPi, for 12 minutes at each level on average), at matching peak airway pressure. We simultaneously recorded EAdi, airway, esophageal pressure, and flow. Tracings were analyzed to measure dynamic PEEPi (decrease in esophageal pressure to generate inspiratory flow), and intrinsic-EAdi (EAdi value at the onset of inspiratory flow), and IDEAdi (inspiratory delay between the onset of EAdi and the inspiratory flow).

**Results:** Mean airway pressure was comparable for PSP and PSN at same target levels. The pressure necessary to overcome PEEPi, intrinsic-EAdi, and IDEAdi was significantly lower in PSN as compared with PSP, decreased with increase in PEEPe, although the effect of external PEEP was less pronounced in PSN. Intrinsic-EAdis at different PEEPe in PSP were tightly correlated with dynamic PEEPi (r2 =0.46, r2 =0.56, r2 =0.71, r2 =0.62, respectively).

**Conclusions:** In COPD patients with PEEPi, PSN compared with PSP, led to a decrease in the pressure necessary to overcome PEEPi, which could be reliably monitored by the electrical activity of the diaphragm before inspiratory flow onset (intrinsic-EAdi) in PSP.

## P200 Distribution of ventilation in obese and non-obese patients: obesity greatly attenuates peep-induced hyperdistension.

### G Alcala^1^, C Morais^1^, R De Santis^2^, M Tucci^1^, M Nakamura^1^, E Costa^1^, M Amato^1^

#### ^1^University of Sao Paulo School of Medicine, Sao Paulo, Brazil; ^2^Massachusetts General Hospital, Boston, MA, USA

**Introduction:** Atelectasis develops in critically ill obese patients submitted to mechanical ventilation. The pressure exerted by the abdominal weight on the diaphragm causes maldistribution of ventilation with increased pleural pressure and diminished response to PEEP. Our objective was to analyze the effects of PEEP in the distribution of ventilation in obese and non-obese patients according to BMI (obese >= 30 kg/m^2^, or non-obese: 20 to 29.9 kg/m^2^), using electrical impedance tomography (EIT).

**Methods:** We assessed the regional distribution of ventilation of surgical and clinical patients submitted to a decremental PEEP itration monitored by EIT. We calculated the percent ventilation to the nondependent (anterior) lung regions at the highest and lowest PEEP applied. The highest compliance of respiratory system was consistently observed at intermediate values of PEEP (between those extreme values), indicating that the highest PEEP caused pulmonary overdistension, whereas the lowest PEEP likely caused dependent lung collapse

**Results:** Were enrolled 37 patients, with 15 non-obese patients (25,7±2 kg/m^2^) and 22 obese patients (32.4± 1.7 kg/m^2^). All patients presented progressively decreased ventilation to dependent (posterior) lung regions when PEEP was lowered (P<0.001). Obese patients consistently presented higher ventilation to the anterior lung zones (when compared no nonobese), Fig. 1, at equivalent PEEP levels: (68±2 % vs 59±1 %, P<0.001 at the lowest PEEP; 40±0.1 % vs 32±0.1%, P<0.001 at the highest PEEP). The Higher and Lower PEEP levels applied were similar in obese vs. non-obese.

**Conclusions:** Obese patients present a displacement of ventilation to the anterior region when compared to non-obese patients.

**Fig. 1 (abstract P200).**
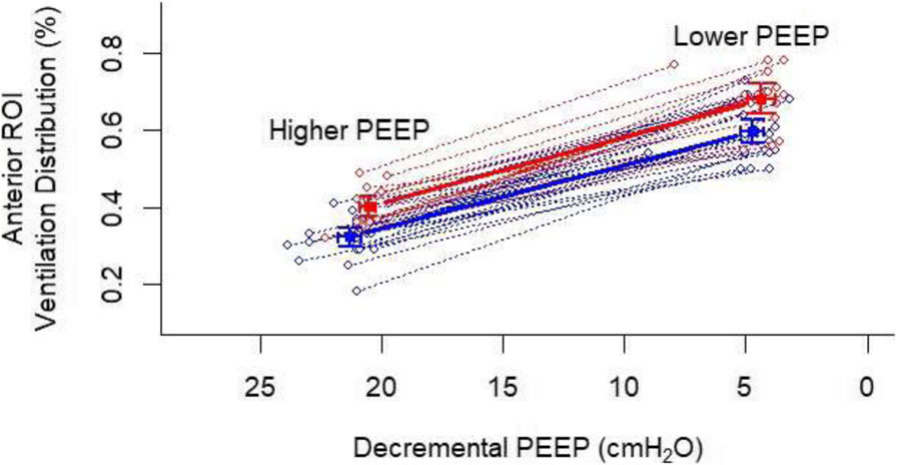
Distribution of ventilation to non-dependent lung regions. Red= obese patients; Blue = non-obese patients

## P201 Low flow CO2 removal in combination with renal replacement therapy effectively reduces ventilation requirements in hypercapnic patients – results of a pilot study

### J Nentwich^1^, S Kluge^2^, D Wichmann^2^, H Mutlak^3^, S Lindau^3^, S John^1^

#### ^1^Klinikum Nürnberg, Nuremberg, Germany; ^2^University Medical Center Hamburg-Eppendorf, Hamburg, Germany; ^3^University Hospital Frankfurt, Frankfurt, Germany

**Introduction:** Lung protective ventilation is the mainstay of mechanical ventilation in critically ill patients [1]. Extracorporeal CO2 removal (ECCO2R) can enhance such strategies [2] and has been shown to be effective in low flow circuits based on renal replacement platforms [3, 4, 5]. We show the results of a pilot study using a membrane lung in combination with a hemofilter based on a conventional renal replacement platform (Prismalung™) in mechanically ventilated hypercapnic patients requiring renal replacement therapy (NCT02590575).

**Methods:** The system incorporates a membrane lung (0.32 m2) in a conventional renal replacement circuit downstream of the hemofilter. 26 mechanically ventilated patients requiring renal replacement therapy were included in the study. Patients had to be hypercapnic at inclusion under protective ventilation. Changes in blood gases were recorded after implementation of the extracorporeal circuit. Thereafter ventilation was intended to be decreased per protocol until baseline PaCO2 was reestablished and changes in VT and Pplat were recorded. Data from 20 patients were included in the final analysis.

**Results:** The system achieved an average CO2 removal rate of 43.4±14.1 ml/min which corresponded to a PaCO2 decrease from 68.3±11.8 to 61.8±11.5 mmHg (p<0.05) and a pH increase from 7.18±0.09 to 7.22±0.08 (p<0.05) [Fig. 1]. After adaption of ventilator settings we recorded a decrease in VT from 6.2±0.9 to 5.4±1.1 ml/kg (p<0.05) and a reduction of Pplat from 30.6±4.6 to 27.7±4.1 cmH2O (p<0.05). These effects were even more pronounced in the “per protocol” analysis [Fig. 2].

**Conclusions:** Low flow ECCO2R in combination with renal replacement therapy provides partial CO2 removal at a rate of over 40 ml/min can significantly reduce invasiveness of mechanical ventilation in hypercapnic patients.

**References**

[1] ARDSNet 2000,

[2] Bein 2013

[3] Terragni 2009

[4] Forster 2013

**Fig. 1 (abstract P201).**
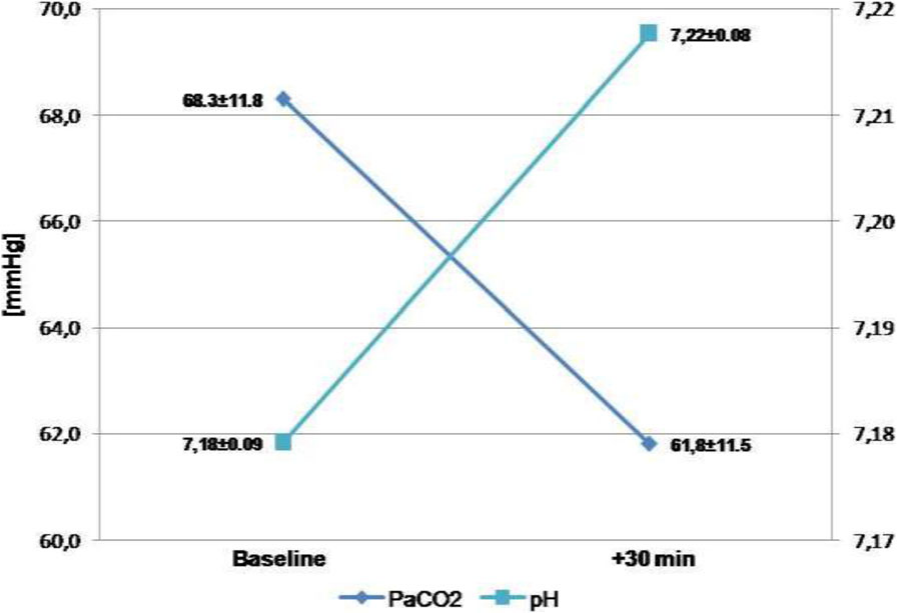
30 minutes after implementation of the combined renal replacement and ECCO2R circuit a moderate decrease in PaCO2 (-6.5 mmHg) corresponding to a slightly higher pH (0.04) was observed

**Fig. 2 (abstract P201).**
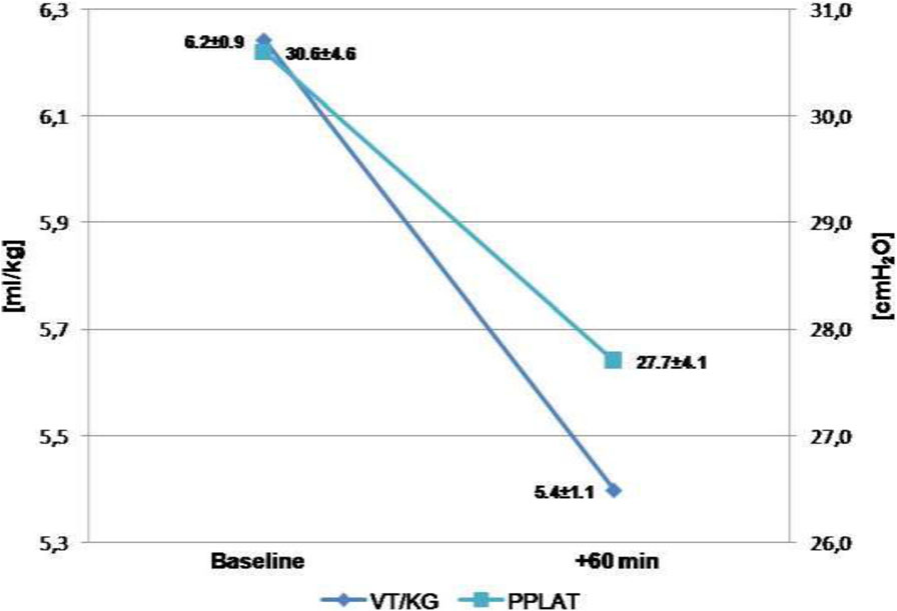
Reestablishment of initial PaCO2 resulted in a significant decrease in tidal volume (-0.8 ml/kg, p<0.05) and plateau pressure (-2.9 cmH2O, p<0.05)

## P202 Is the filters setting relevant in terms of performances during low flow(lf)-ECCO2R in combination with CRRT?

### S Mantovani^1^, F Biagi^1^, R Baldini^1^, A Gmerek^1^, C Barth^1^, VM Ranieri^2^

#### ^1^B. Braun Avitum AG, Melsungen, Germany; ^2^Sapienza University, Rome, Italy

**Introduction:** In ECCO2R-CRRT, efficiency of CO2 removal is higher positioning the oxygenator (OXY) up-stream than down-stream the haemofilter due to higher blood flow (BF) [1]. We tested whether this effect was due to lower pre-filter pressure (PFP).

**Methods:** ECCO2R-CRRT circuit was tested in-vitro (n=10) with the following settings: 5 L bovine blood; BF 450 ml/min; OXY 1.81 m2 (Euroset); CVVH post mode; substitution flow 2500 ml/h; UF rate function off; 1.5 m2 haemofilter (Diapact®, B.Braun Avitum); sweep air flow 4.5 l/min. PFP was evaluated at baseline, 24, 48 and 72 hours. CO2 extraction was measured at BF of 100, 300 and 500 ml/min. Sweep air flow/blood ratio was 1:10. CO2 was add to obtain PaCO2 of 80 mmHg. CO2 removal rate calculation (2): CO2 removal rate = (CO2 ECCO2R inlet– CO2 ECCO2R outlet)* blood flow (Eq.1)

CO2 molar volume at 25 °C [l/mol] = 24; solubility of CO2 at 37 °C = 0.03 mmol/(l*mmHg); HCO3i = inlet HCO3 concentration [mmol/l]; HCO3o = outlet HCO3 concentration [mmol/l]; Pi CO2 = inlet CO2 partial pressure [mmHg]; PoCO2 = outlet CO2 partial pressure [mmHg] equation1 becomes: CO2 removal rate=24 x ((HCO3i + 0.03 x PiCO2) - (HCO3o + 0.03 x PoCO2)) x blood flow (Eq.2)

**Results:** BF of 450 ml/min was always reached with the up-stream configuration. BF was reduced to 400 ml/min with the down-stream configuration due to high PFP alarm (Table 1). CO2 removal increased to 34.5±13.9 to 69.1±29.5, and 126.0±28.4 ml/min, at BF of 100, 300 and 500 ml/min (p<0.05).

**Conclusions:** BF of 500 ml/min can be reached only with the up-stream configuration due to lower circuit PFPs. BF directly correlates to CO2 removal efficiency. We may speculate that simultaneous use of CRRT and LF-ECCO2R and activation of the UF rate function with the down-stream setting may further increase PFP thus forcing to more enhanced reduction of BF and less effective CO2-removal.

**References**

1. Crit Care Med 2015;43(12):2570-81

2. Anaesth Crit Care Pain Med 34;(2015):135-140

**Table 1 (abstract P202).** Pre-filter pressures dependent on OXY position (ANOVA, p<0.001)

Setting	baseline	24 h	48 h	72 h
up-stream PFP (mmHg)	278.4 ± 9.4	370.0 ± 7.6	415.0 ± 5.2	451.4 ± 7.6
down-stream PFP (mmHg)	380.8 ± 7.2	447.0 ± 4.7	488.2 ± 5.1	481.8 ± 6.0

## P203 Low-flow combined extracorporeal renal-pulmunary support: a retrospective observational case series

### G Consales^1^, L Zamidei^1^, G Michelagnoli^1^, B Ammannati^2^, G Boscolo^3^, G Dipalma^4^, P Isoni^5^, F Turani^6^

#### ^1^Santo Stefano Hospital, Prato, Italy; ^2^University of Florence, Florence, Italy; ^3^Ospedale dell’Angelo, Maestre, Italy; ^4^IRCCS Policlinico San Donato, San Donato, Italy; ^5^ Marino Hospital, Cagliari, Italy; ^6^Aurelia and European Hospital, Rome, Italy

**Introduction:** We describe the use of a novel low-flow ECCO2R-CRRT device (PrismaLung-Prismaflex, Baxter Healtcare Gambro Lundia-AB-Lund, Sweden) for simultaneous lung-renal support.

**Methods:** A retrospective review of patients submitted to PrismaLung-Prismaflex due to AKI associated to hypercapnic acidosis during the period May 2016 - August 2017 at Prato Hospital ICU was performed. Data collected were: demographic, physiologic, complications, outcome. Data were presented as mean ± DS; Anova Test was used to compare changes of parameters over time; significance was set at P< 0,05.

**Results:** We identified 13 patients (mean age 71 ± 13 yr, mean SOFA 12 ± 3). Causes of hypercapnia were moderate ARDS (n=4) and AE-COPD (n=9). In all patients a 13fr double lumen cannula was positioned and 350 ml/min blood-flow with 10 lt oxygen sweep-gas-flow was maintained; iv-heparin aiming to double aPTT was used. Haemo-diafiltration (effluent flow 35 ml/kg/hour) was delivered. In all cases PrismaLung-Prismaflex improved respiratory and metabolic parameters (Figs. 1 and 2) without any complications. All patients survived to the treatment, nevertheless 2patients (1AE-COPD; 1ARDS) died during ICU stay due to irreversible cardiac complications. In ARDS cases: 3 patients were successfully weaned from IMV, mean duration of the treatment was 88 ± 31hours, mean duration of IMV after ECCO2R-CRRT was 2 ± 2 days. In AE-COPD cases: intubation was avoided in 3 patients at risk of NIV failure, 6 patients were successfully weaning from IMV, mean duration of the treatment was 79 ± 31 hours, mean duration of IMV after ECCO2R-CRRT was 0,1 ± 0,3 days.

**Conclusions:** The use of PrismaLung-Prismaflex has been safe and effective: it may be argued that it could be due to the low-blood-flow used. The positive results of this preliminary study may constitute the rational for the design of a larger randomized control trial.

**Fig. 1 (abstract P203).**
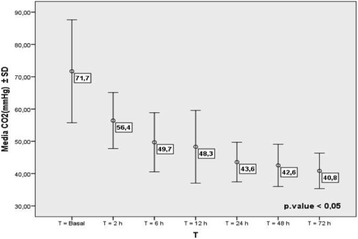
Mean CO2 ± DS (mmHg) during ECCO2R-CRRT

**Fig. 2 (abstract P203).**
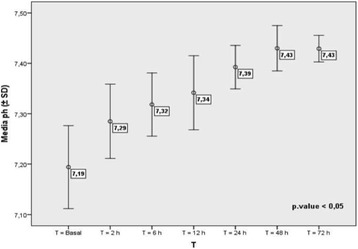
Mean pH ± DS during ECCO2R-CRRT

## P204 Systemic IL-18 production and spontaneous breathing trial (SBT) outcome: the effect of sepsis

### A Kyriakoudi, N Rovina, O Koltsida, M Kardara, I Vasileiadis, M Daganou, N Koulouris, A Koutsoukou

#### National and Kapodestrian University of Athens Medical school, Athens, Greece

**Introduction:** Spontaneous breathing trial (SBT), a routine procedure during ventilator weaning, entails cardiopulmonary distress, which is higher in patients failing the trial. An intense inflammatory response, expressed by increased levels of pro-inflammatory cytokines, is activated during SBT. Sepsis, a common condition in ICU patients, has been associated with increased levels of the pro-inflammatory cytokine IL-18. IL-18 produced among others by skeletal muscles, has been associated with severe muscle wasting and maybe by ICU acquired weakness. We hypothesised that IL-18 increases during SBT, more evidently in SBT failures. We anticipate this response to be more pronounced in formerly septic patients fulfilling the criteria for SBT.

**Methods:** 75 SBTs of 30-min duration were performed and classified as SBT failure or success. Blood samples were drawn before, at the end of the SBT and 24 hours later. Serum IL-18 levels and other inflammatory mediators, commonly associated with distress, were determined and correlated with SBT outcome. Subgroup analysis between septic and non-septic patients was performed.

**Results:** SBT failure was significantly higher in septic patients compared to non-septic (41% vs 13%, OR=4.5 95% CI: 1.16-17.68, p 0.022). Septic patients had significantly elevated IL-18 levels at baseline compared to non-septic (p<0.05). A trend of increase in IL-18 at 30 min of SBT was observed in both groups. Serum levels of IL-18 were returned to baseline values after 24 h. These changes however, were not significantly different. Increased levels of IL-18 in septic patients were correlated with a rapid shallow breathing index >75 (p=0.028) and APACHE II score (p=0.05). No changes were observed in the remaining inflammatory mediators in both groups.

**Conclusions:** Elevated IL-18 levels in septic patients were associated with cardiopulmonary distress and disease severity. IL-18 may have a potential role as a predictor of SBT failure in septic patients.

## P205 Assessing the effects of duration of apnea on adequacy of ventilation in the post-operative environment

### JE Freeman^1^, H Essber^2^, B Cohen^2^, M Walters^2^, D Chelnick^2^, L Glosser^2^, C Hanline^2^, A Turan^2^

#### ^1^Respiratory Motion, inc., Waltham, MA, USA; ^2^Cleveland Clinic Foundation, Cleveland, OH, USA

**Introduction:** Maintaining respiratory sufficiency is a vital component of post-operative care, yet clinicians often rely on subjective assessments or secondary indicators. Patients with apneic episodes are often considered to have respiratory insufficiency regardless of whether these apneic episodes are compensated by large recovery breaths. We used a non-invasive respiratory volume monitor (RVM) to measure minute ventilation (MV) to assess ventilation in patients experiencing apnea in the PACU and general floor.

**Methods:** We used an RVM (ExSpiron1Xi, Respiratory Motion, Inc.) to continuously monitor MV for 48h following elective abdominal surgery. MV was expressed as percent of predicted MV (MVPRED); Low MV was defined as MV<40%MVPRED. For each apnea (ie, respiratory pause >10 seconds), we calculated the patient’s corresponding MV over the 30, 60, 90, and 120s windows following the start of the apnea.

**Results:** 216 patients (110 males, BMI: 26.7 (15.1-41.2)kg/m2) were monitored for 42.0±0.9 hours. 49985 apneas were identified ranging from 10-117s (Fig. 1A). Apneas were observed in 99% of patients, suggesting low predictability of respiratory insufficiency. The average MV was 73±2.4%MVPRED, as patients were often sleeping or mildly sedated. We assessed the effects of each apnea on the temporally associated MV (Fig. 1B). While apneas ranging in length from 10-18s decrease MV by as much as 30%, their effect over 1min is <10%. On a 2min time scale, even 60s apneas led to LowMV just 20% of the time (Fig. 1C).

**Conclusions:** While apneas were ubiquitous, they seldom led to LowMV over clinically relevant time scales. Large compensatory breaths following an apnea generally restored MV to near pre-apnea levels. Nonetheless, some apneas can become dangerous when ignored, as when subsequent sedation decreases compensatory breath size. RVM data provide a better metric of respiratory competence, driving better assessment of patient risk and individualization of care.

**Fig. 1 (abstract P205).**
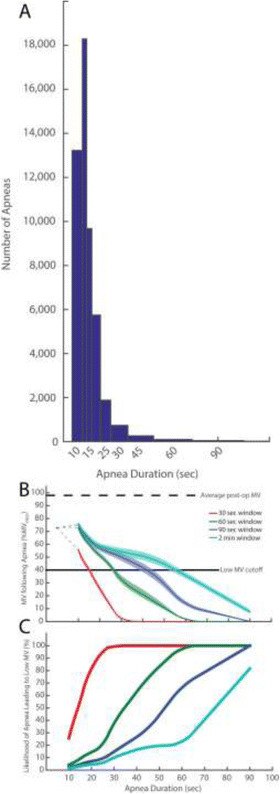
The effect of apneas on respiratory status. (A) Distribution of apneas across a surgical population. Apneas >10sec were recorded in 99% (214 out of 216) patients in the post-operative period and 92% of the recorded apneas were <20s long. (B) Sustained MV over various time windows (30-sec – red, 60-sec – green, 90-sec – purple, 2-min – cyan) following the onset of apnea. Note that, an apnea of 30-sec will (by definition) drive MV over a 30-sec window down to 0, but will only decrease MV over a 60-sec window down to ~35% MVPRED and to less than 60% over a 2-min window. (C) Likelihood of an apnea of specific length to decrease MV below the Low MV cutoff over various time windows. Note that a single 10-sec apnea has just a 25% chance to decrease MV below 40% in a 30-sec window and less than 2% chance to decrease MV below the cutoff over a 2-min window. Even 60-sec apneas have just 20% chance of decreasing sustained MV over a 2-min window below the 40% MVPRED cutoff

## P206 Spectrum of histopathological findings and underlying causes in 59 patients with diffuse alveolar hemorrhage

### S Sangli, M Baqir, J Ryu

#### Mayo Clinic, Rochester, MN, USA

**Introduction:** Diffuse alveolar hemorrhage (DAH) is an acute life-threatening event and recurrent episodes of DAH may result in irreversible interstitial fibrosis. Identifying the underlying cause is often challenging but is needed for optimal treatment. Lung biopsy is often performed in the diagnostic evaluation of patients with suspected DAH. However, the role of lung biopsy in this clinical context is unclear. Hence, we sought to identify the spectrum of histopathologic findings and underlying causes in patients with DAH who underwent lung biopsy, surgical or transbronchial.

**Methods:** We identified 59 patients who underwent surgical lung biopsy (n = 25) or bronchoscopic biopsy (n = 34) in the evaluation of DAH over a 19-year period from 1999 to 2017. We extracted relevant clinical pathologic and laboratory data.

**Results:** The median age in our cohort was 67 years with 51% females. Serologic evaluation was positive in 47% of patients (n=28). Most common histopathologic findings on surgical lung biopsy included alveolar hemorrhage (AH) with capillaritis in 11 patients of whom six had necrotizing capillaritis, followed by AH without capillaritis in 7 patients. The most common histopathologic finding on bronchoscopic lung biopsy was AH without vasculitis/capillaritis in 22 patients, followed by AH with capillaritis in 11 patients. There were no procedure related complications or mortality observed with either method of lung biopsy. The clinico-pathologic diagnoses in these patients are shown in Tables 1 and 2.

**Conclusions:** In patients with DAH undergoing lung biopsy alveolar hemorrhage without capillaritis was found to be the most common histopathologic finding followed by pulmonary capillaritis. These histopathologic findings contributed to the final clinico-pathologic diagnoses of granulomatous polyangiitis and microscopic polyangiitis in a substantial portion of cases. Future studies are needed to ascertain the benefits vs. risks of lung biopsy in patients with suspected DAH.

**Table 1 (abstract P206).** Histopathological findings on surgical lung biopsy and common clinico-pathologic diagnoses

Histopathology:	Clinical Diagnoses:
AH with capillaritis(11)	Microscopic polyangitis(4)
	Granulomatosis polyangitis(4)
AH without capillaritis(7)	Antiphospholipid syndrome(1)
	Microscopic polyangitis(1)
AH with diffuse alveolar damage(4)	Microscopic polyangitis(3)
AH with Pulmonary vascular changes(1)	Pulmonary hypertension(1)

**Table 2 (abstract P206).** Histopathological findings on bronchoscopic lung biopsy and common clinico-pathologic diagnoses

Histopathology:	Clinical Diagnoses:
AH without capillaritis (22)	Granulomatosis polyangitis (4)
	Microscopic polyangitis (3)
AH with capillaritis (11)	Granulomatosis polyangitis (7)
	Drug induced (1)
AH with diffuse alveolar damage (1)	Enterococcus bacteremia in AIDS (1)

## P207 Cardiovascular risk in patients with severe COPD in early stages of renal dysfunction: the role of vitamin D

### N Trembach, V Yavlyanskaya

#### Kuban State Medical University, Krasnodar, Russia

**Introduction:** The aim of research was to study the frequency and structure of acute cardiovascular events (ACE) in patients with severe COPD in the early stages of renal dysfunction with different levels of vitamin D.

**Methods:** The study included 102 patients with 3-4 stage of COPD. All patients were diagnosed with 1-2 stage of chronic kidney disease according to the recommendations of KDIGO (2012). In addition to general clinical examination, the level of vitamin D was assessed in all patients and the frequency and structure of ACE in the previous 12 months were studied.

**Results:** The average level of vitamin D was 7.2 ± 2.1 ng/ml. The incidence of insufficiency, deficiency and severe deficiency was 5%, 33% and 64%, respectively. The frequency of occurrence of the ACE was 42 cases. Acute coronary syndrome (ACS) and pulmonary thromboembolism (PTE) were observed relatively more frequently. When ranking according to the degree of severity of vitamin D level reduction, it was found that acute cardiovascular events occurred significantly more frequently in patients with severe vitamin D deficiency: 24 cases out of 43, including ACS (5 cases), PTE (2 cases), ischemic stroke (4 cases), severe arterial hypertension (5 cases), cardiac arrhythmia (8 cases). In patients with vitamin D levels of 10-20 ng/ml 12 cases of ACE were observed (p <0.05 compared to group with severe deficiency) - 3 cases of ACS, 2 cases of ischemic stroke, 4 cases of severe arterial hypertension, 3 cases of cardiac arrhythmia). Only 6 cases of ACE were observed (p <0.05 compared to group with severe deficiency) in patients with insufficiency of vitamin D (2 cases of ACS, 3 cases of ischemic stroke, 1 OHMK, 1 case of PTE).

**Conclusions:** The frequency of acute cardiovascular events is significantly higher in patients with severe COPD and lower values of vitamin D. Thus, vitamin D can be considered as a possible target for the control and therapy of severe COPD.

## P208 Intravenous magnesium sulfate in the treatment of acute exacerbations of COPD: a randomized controlled trial

### E Pishbin, E Vafadar Moradi

#### Mashhad University of Medical Sciences, Mashhad, Iran

**Introduction:** Intravenous (IV) magnesium has an established role in the treatment of acute asthma attack. It is attractive as a bronchodilator because it is relatively cheap and has minimal side effects. There are few studies evaluating the effect of magnesium on acute exacerbation of COPD and the results of them are contradictory.

The aim of this study was to investigate the effect of IV magnesium sulfate in the treatment of patients presenting to the emergency department (ED) with acute exacerbation of COPD.

**Methods:** In this randomized controlled trial, adult patients presented to the ED with acute exacerbation of COPD were randomly allocated in 2 groups. Patients in the study group received the standard treatment, plus 2 gram of IV magnesium sulfate and patients in the control group received the standard treatment, plus placebo. The outcomes included admission rate, intubation rate, changes in PEFR, SpO2 and dyspnea severity score. PEFR, SpO2 and dyspnea severity score were documented before the treatment and 20 minutes after administration of MgSO4 or placebo.

**Results:** 34 patients were included in the study (17 patients in each group), 16 patients were men and 18 patients were women. The study group had significantly more improvement in the dyspnea severity score (P=0.001) and SpO2 (P= 0.004) as compared to the control group. But the difference in the changes in PEFR, intubation rate and admission rate were not clinically significant between 2 groups. (P>0.05)

**Conclusions:** Adding magnesium sulfate to the standard treatment in patients with acute exacerbation of COPD leads to more improvement in dyspnea severity score and SpO2 although it does not reduce admission rate and intubation rate.

Small sample size and the dependency of PEFR on patient’s effort are limitations of the present study. Furthermore we did not exclude the patients with the Cor pulmonale which may had an effect on the low response rate to MgSO4.

## P209 Assessment of peripheral chemoreflex sensitivity in patients with COPD

### N Trembach, I Zabolotskikh

#### Kuban State Medical University, Krasnodar, Russia

**Introduction:** Assessing the sensitivity of the peripheral chemoreflex (SPCR), we can predict the likelihood of developing respiratory and cardiovascular disorders. SPCR is one of the markers of disease progression and good prognostic marker [1]. Disturbed respiratory mechanics can make it difficult to evaluate. Breath-holding test may be helpful in such situation, the results of this test are inversely correlated with peripheral receptor sensitivity to carbon dioxide in healthy people [2].The aim of the study was to compare the breath-holding test to single-breath carbon dioxide test in the evaluation of the sensitivity of the peripheral chemoreflex in subjects with COPD.

**Methods:** The study involved 78 patients with COPD with FEV1/FVC <70% of predicted, all participants were divided into two groups depending of disease severity (GOLD classification, 2017). In group 1 (mild-to-moderate COPD, n=46) all patients had FEV1>=50% and in group 2 (severe-to-very severe COPD, n=32) all patients had FEV1<50%. Breath-holding test was performed in the morning before breakfast: voluntary breath-holding duration was assessed three times, with 10 min intervals [2]. A mean value of the duration of the three samples was calculated. The single-breath carbon dioxide test [3] was performed the next day. The study was approved by the local ethics committee. All subjects provided signed informed consent to both tests. The reported study was funded by RFBR, research project No. 16-34-60147 mol_a_dk.

**Results:** The average SPCR measured with single-breath carbon dioxide test was 0.42±0.12 L/min/mmHg in group 1 and 0.26±0.08 L/min/mmHg in group 2. The average breath-holding duration was 44±13 seconds in group 1 and 37±13 seconds in group 2. During the correlation analysis a significant negative correlation between the results of two tests was noted (-0.79, p <0.05) in group 1 and a weak negative correlation in group 2 (-0.32, p <0.05).

**Conclusions:** Peripheral chemoreflex sensitivity to carbon dioxide can be indirectly evaluated by a breath-holding test in patients with mild-to-moderate COPD, its assessment in severe COPD need further investigations.

**References**

1. Giannoni A et al. J Am Coll Cardiol 53:1975–80, 2009

2. Trembach N et al. Respir Physiol Neurobiol 235:79–82, 2017

3. Chua TP et al. Eur Clin Invest 25:887–92, 1995

## P209A Outcomes of patients admitted to the intensive care unit with acute exacerbation of pulmonary fibrosis.

### S Sahota, J Paddle, C Powell

#### Royal Cornwall Hospital, Truro, UK

**Introduction:** We investigated outcomes of patients admitted to our general adult ICU for acute exacerbation of pulmonary fibrosis. British Thoracic Society (BTS) and National Institute for Health and Care Excellence (NICE) guidelines state that these patients should not routinely be admitted to ICU for respiratory support as the mortality is very high [1,2]. We decided to investigate our mortality rates for this cohort of patients and review them in the context of the BTS and NICE guidelines.

**Methods:** This retrospective observational study reviewed all patients admitted to the ICU for respiratory support between October 2012 and January 2017. The data was collected from the hospital electronic and paper notes, and data collected was mortality rate, APACHE II score, ICNARC score, type of respiratory support received and whether there was documentation of advanced decisions in case of acute deterioration.

**Results:** There were 12 patients admitted to the ICU with acute respiratory failure as a complication of pulmonary fibrosis. The median APACHE II score was 22 and ICNARC standardised mortality ratio was 5.2. Nine patients died on ICU (75%) and hospital mortality was ten (83%). Eight patients (67%) received high flow nasal oxygen, six (50%) received non-invasive ventilation, and two (17%) received invasive ventilation. The median time to death was 3.7 days. Of 11 patients for whom paper notes were available, no patient had any documented ceiling of care or end of life decisions.

**Conclusions:** Our study confirmed a very high mortality in this cohort of patients, supporting national guidance that invasive respiratory support has limited value. We advise that frank discussion with patients and their families should happen early after diagnosis, such that end of life plans are already in place in the event of acute deteriorations.

**References**

1. Wells et al. Thorax 63. Suppl 5 v1-v58, 2008

2. NICE Clinical Guidelines: Idiopathic pulmonary fibrosis in adults: diagnosis and management (June 2013) www.nice.org.uk, accessed 15 Nov 2017

## P210 Acute respiratory failure (ARF): differences in diaphragmatic ultrasonography in medical and surgical patients

### G Zani, F Di Antonio, M Pichetti, V Cricca, A Garelli, C Gecele, M Campi, F Menchise, M Valbonetti, S Mescolini, E Graziani, M Diamanti, C Nencini, FD Baccarini, M Fusari

#### Santa Maria delle Croci Hospital, Ravenna, Italy

**Introduction:** ARF is common in critically ill patients. We compared diaphragm contractile activity in medical and surgical patients admitted to ICU with a diagnosis of ARF.

**Methods:** Adult medical and major abdominal laparotomic surgical patients admitted to a general ICU with a diagnosis of ARF were enrolled. ARF was defined as a PaO2/FiO2 ratio<=300 mmHg/% and need for mechanical ventilation (MV) for at least 24 hours. Diaphragmatic ultrasound was realized bedside when the patient was stable and able to perform a trial of spontaneous breathing. A convex probe was placed in right midaxillary line (8th-10th intercostal space) to evaluate right hemidiaphragm. Diaphragmatic respiratory excursion and thickening were evaluated in M-mode on 3 consecutive breaths and thickening fraction (TF) was calculated. Antropometric, respiratory and hemodynamic parameters, SAPS2, SOFA score, duration of MV, need for tracheotomy and timing, septic state and site of infection, superinfections, ICU and inhospital length of stay (LOS) and outcome were recorded. Patients with trauma and neuromuscular disorders were excluded. P<0.05 was considered significant.

**Results:** We enrolled 30 patients: 40% medical and 60% surgical, without differences for age, sex, BMI, SAPS2, SOFA score, sepsis and superinfections. Moderate ARF was prevalent in both groups. During diaphragmatic examination, no differences were recorder for respiratory rate, hemodynamic state and fluid balance. Surgical patients showed a lower but not significant diaphragm excursion (1.6vs1.8cm), instead TF was significantly reduced (58vs90%,p<0.05). No differences emerged on duration of MV, but tracheotomy were higher in medical ones (30vs11%,p<0.05). ICU and inhospital LOS do not differ between medical and surgical patients and mortality rate was respectively 17% and 22%.

**Conclusions:** In ARF, surgical patients showed a lower diaphragm contractility compared to medical ones, maybe due to the combination of anesthetic and surgical effects, but with no influence on outcome.

## P211 Ultrasound and diaphragmatic dysfunction: impact of surgical approach

### M Luperto^1^, M Bouard^2^, S Mongodi^1^, F Mojoli^1^, B Bouhemad^2^

#### ^1^Fondazione IRCCS Policlinico San Matteo, University of Pavia, Pavia, Italy; ^2^Centre Hospitalier Régional Universitaire de Dijon Bourgogne, Dijon, France

**Introduction:** Diaphragm ultrasound (DUS) easily identifies diaphragmatic dysfunction (DD) in ICU patients [1], a potential cause of weaning failure (WF).

**Methods:** Prospective observational monocenter study. We enrolled adults ICU patients at day 1 after surgery, extubated, with no neuromuscolar diseases. In each patient DUS (caudal displacement (CD) and thickening fraction (TF)[2]), was performed. WF was defined as NIV or reintubation within 48h after extubation.

**Results:** We enrolled 43 patients (25 males, age 70.9±10.2 years, BMI 27.1±5, MV length 5h [4-6], ICU stay 1.5 days [1.0-2.0]). Surgery was performed by laparotomy (La-28%), sternotomy (St-53%) or right thoracotomy (Rt-19%). No differences were remarked in patients’ characteristics, MV length, ICU stay Table 1. Rt patients had WF more than other approaches (p=0.0080); right CD and TF were significantly lower in Rt (p<0.05, Table 2). Rt was a risk factor for WF (OR 17.5, 95% CI 1.38-222.62, p=0.0024).

**Conclusions:** Postsurgical right DD is significantly affected by surgical approach; Rt has the highest percentage of DD, probably explaining the highest rate of WF.

**References**

1. Zambon et al Annual Update in Inten Care and Emerg Med 427-438, 2013

2. Umbrello M et al. Respir Care 2016

**Table 1 (abstract P211).** Population characteristics (#thoracotomy significant vs. sternotomy and laparotomy; MV mechanical ventilation, BMI body mass index)

	Laparotomy (n=12)	Sternotomy (n=23)	Thoracotomy (n=8)	p
Age (years)	68.0 [60.5-81.5]	72.0 [63.0-81.0]	76.0 [68.0-79.5]	0.7809
Males	7 (58.3%)	13 (56.5%)	5 (62.5%)	0.9570
BMI	27.2 [23.4-32.0]	26.0 [23.2-29.3]	26.7 [23.2-29.6]	0.6588
Weaning failure	3 (25.0%)	7 (30.4%)	7 (87.5%)#	0.0080
MV length (min)	285 [220-375]	285 [240-360]	300 [240-450]	0.8595
ICU stay (days)	1 [1-3]	1 [1-2]	2 [2-6]	0.2156

**Table 2 (abstract P211).** Ultrasound results (#thoracotomy significant vs. sternotomy and laparotomy)

	Laparotomy (n=12)	Sternotomy (n=23)	Thoracotomy (n=8)	p
Right CD (cm)	1.23 [0.82-1.71]	1.43 [1.01-1.79]	0.74 [0.54-1.22]#	0.0375
Right TR (%)	25.6 [16.6-31.5]	26.0 [13.5-31.4]	11.4 [4.2-18.6]#	0.0393
Left CD (cm)	0.98 [0.68-1.61]	1.43 [1.21-2.0]	1.61 [1.25 -2.16]	0.0999
Left TR ratio (%)	21.1 [16.4-34.4]	25.7 [19.0-36.5]	22.0 [14.7-29.4]	0.5091

## P212 Does it make difference to measure diaphragm thickness with M mode or B mode?

### B Kalın, G Gursel

#### Gazi University School of Medicine, Ankara, Turkey

**Introduction:** Diaphragm dysfunction occurs quickly in mechanically ventilated patients and is associated with prolonged mechanical ventilation and poor outcome. Recent literature suggest that diaphragm thickening fraction (DTF) measured by ultrasound can be useful to predict weaning outcome. However, there is no standardized approach in the measurement of diaphragm thickness (DT) and limited data exist comparing different measurement techniques of diaphragm thickness (M mode-MM or B mode-BM). For example movements of the diaphragm relative to the transducer during the respiratory cycle may result in unintended comparison of different points of the diaphragm at end expiration and inspiration during MM measurements. The goal of this study was to compare MM with BM in the measurement of diaphragm thickness and DTF in the ICU patients.

**Methods:** DT was measured in right diaphragm during tidal breathing. Three measurements of the DT were taken both in MM and BM and averaged to report the mean. DT was measured during inspiration and expiration and DTF was calculated. Bias and agreement between the 2 measurement methods was evaluated with Blant and Altman test.

**Results:** Forty-two patients were enrolled in the study. Diaphragm could not visualize or measured in 8 patients and measurements of 34 patients were analyzed. Despite existance of different degree diaphragm movements relative to the ultrasound transducer secondary to respiratory movement there was no significant difference between the measurement results of MM and BM. There was good agreement and no significant proportional bias between the measurements of 2 modes (p>0.05). BM and MM measurements during the inspiratory (0.29± 0.07& 0.30±0.07 cm; p: 0.159), expiratory (0.22±0.06 & 0.22±0.06 cm; p:0.9) phases and DTF were (29±15 & 32±15 %, p:0.217) respectively.

**Conclusions:** Our results suggest that there is no significant difference between in the measurement of diaphragm thickness with MM or BM.

## P213 Diaphragmatic dysfunction in critically ill patients and its impact on outcome. Assessment of diaphragmatic thickness and excursion by ultrasound

### Y Nassar, M Amin, M Ghonemy, H Abulwafa

#### Cairo University, Giza, Egypt

**Introduction:** The diaphragm is considered as the main respiratory muscle, and its dysfunction predisposes to many respiratory complications. Ultrasound (US) is now an accepted method of measuring Diaphragmatic Excursion (DE) and Diaphragmatic thickness (DT). We aimed to detect Diaphragmatic Dysfunction (DD) in critically ill patients and its impact on outcome.

**Methods:** We prospectively recruited consecutively any critically ill adult requiring admission to MICU with SOFA >= 2. Exclusion criteria: diaphragmatic or spine injury, neuromuscular disease, any usage during hospital stay of paralytic agents, aminoglycosides, sedatives or analgesia other than morphine. The right hemi-diaphragm was evaluated by M mode US for DE and B mode US for DT with the patients in the supine position. DT and DE and laboratory measurments were taken on admission and every 48 hr for a total of 3 readings (Day 0, 2, 4).Patients were followed up for length of ICU stay and 30 day mortality. DD was diagnosed if a DT <=0.2 cm and DE <=1.0 cm.

**Results:** The study included 106 subjects. In the total studied group mean age was 51.4 ± 16.3 years, 49 (46.2 %) were males while 57 (53.7 %) were females. The meanSOFA was 5.33 ± 3.66 and Mean APACHE II was 15.42 ±7.91.DD group included 38 (35.8 %) vs. Non DD group included 68 (64.1 %) subjects (p<0.001). PaCO2 was higher (48.2±4.1 vs. 39.3±6.9 mmHg, p=0.01) in DD vs. NDD group respectively. WBCs was higher (15.18×103 ± 7.2×103 vs. 11.78×103 ± 2.9×103 cell/ml, p=0.01) in DD vs. NDD group respectively. LOS was significantly higher (9.70±0.4 vs. 6.68±1.3 days, p=0.02) in DD vs. NDD group respectively. Comorbidities in the two groups are shown in Fig. 1. Mortality was significantly higher (81.5% vs. 23.5%, p<0.001) in DD vs. NDD group respectively.

**Conclusions:** DD was present in nearly one third of ICU patients which was largely associated with a longer ICU stay and a higher mortality. PaCO2 and WBCs were associated with a negative effect on diaphragmatic function.

**Fig. 1 (abstract P213).**
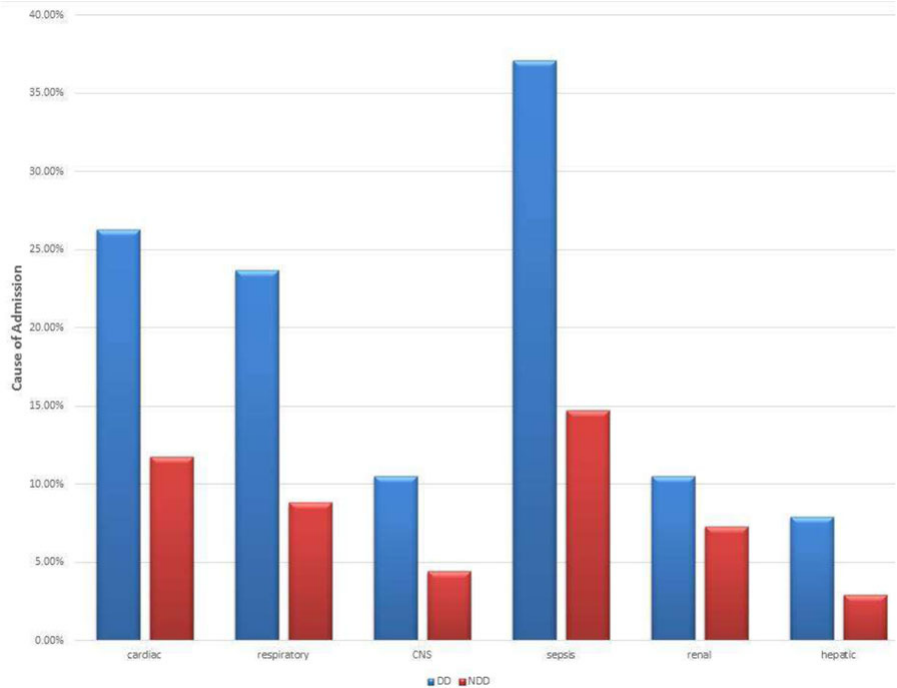
Comorbidities on admission in Diaphragmatic Dysfunction (DD) and Non Diaphragmatic Dysfunction (NDD)

## P214 Multimodal evaluation of diaphragm function after cardiac surgery

### R Pinciroli^1^, M Bottiroli^2^, D Ceriani^2^, S Checchi^1^, A Danieli^3^, A Calini^2^, MP Gagliardone^2^, G Bellani^1^, R Fumagalli^1^

#### ^1^University of Milan-Bicocca, Monza, Italy; ^2^ASST Grande Ospedale Metropolitano Niguarda, Milan, Italy; ^3^University of Milan, Milan, Italy

**Introduction:** Diaphragmatic dysfunction may occur following open-heart surgery. Diaphragm Ultrasound (US) provides a reliable evaluation of diaphragmatic motion. Surface Electromyography (sEMG) is a novel non-invasive technique to assess its electrical activity. [1] Aim of this study is to evaluate, through both sEMG and US, postoperative changes of diaphragm function in patients undergoing cardiac surgery.

**Methods:** US measurements of right and left hemidiaphragm excursion during Quiet Breathing (QB) and Deep Breathing (DB) were obtained before surgery, and postoperatively, at the first spontaneous breathing trial. We simultaneously recorded bilateral sEMG traces. Values of Diaphragmatic Excursion (DE) and sEMG amplitude were analyzed and compared. Unilateral DD was defined as an asymmetry index (right/left excursion at DB) of >1.5 (left DD) or < 0.5 (right DD), as previously reported [2]. DP was defined as an excursion lower than 0.1 cm at DB, or evidence of paradoxical inspiratory motion.

**Results:** 18 adult patients undergoing elective open-heart surgery were enrolled. A significant overall reduction of DE could be identified postoperatively, particularly at DB (5.4 [4.5-6.3] cm vs. 3.2 [2.8-3.6] cm, before vs. after surgery, p< 0.0001). As well, a significant reduction of sEMG amplitude could be measured during DB (18.1 [11.7-27.3] vs. 5.1 [3.2-7.4] μ V, p< 0.0001). DD with evidence of asymmetry was detected through US in 4/18 patients (22%) postoperatively (Fig. 1). Patients with DD showed a higher reintubation rate (2/4 vs. 0/14, DD vs. no DD), leading to a longer time of mechanical ventilation, ICU and hospital length of stay.

**Conclusions:** Compared to baseline, postoperative diaphragmatic function was globally reduced in our patients, as shown by both US and sEMG data. A subgroup of subjects showed a monolateral DD, with an apparent impact on clinical outcome, despite the small sampled population.

**References**

1. Lerolle N et al. Chest 2009

2. Houston JG et al. Clin Radiol 1992

**Fig. 1 (abstract P214).**
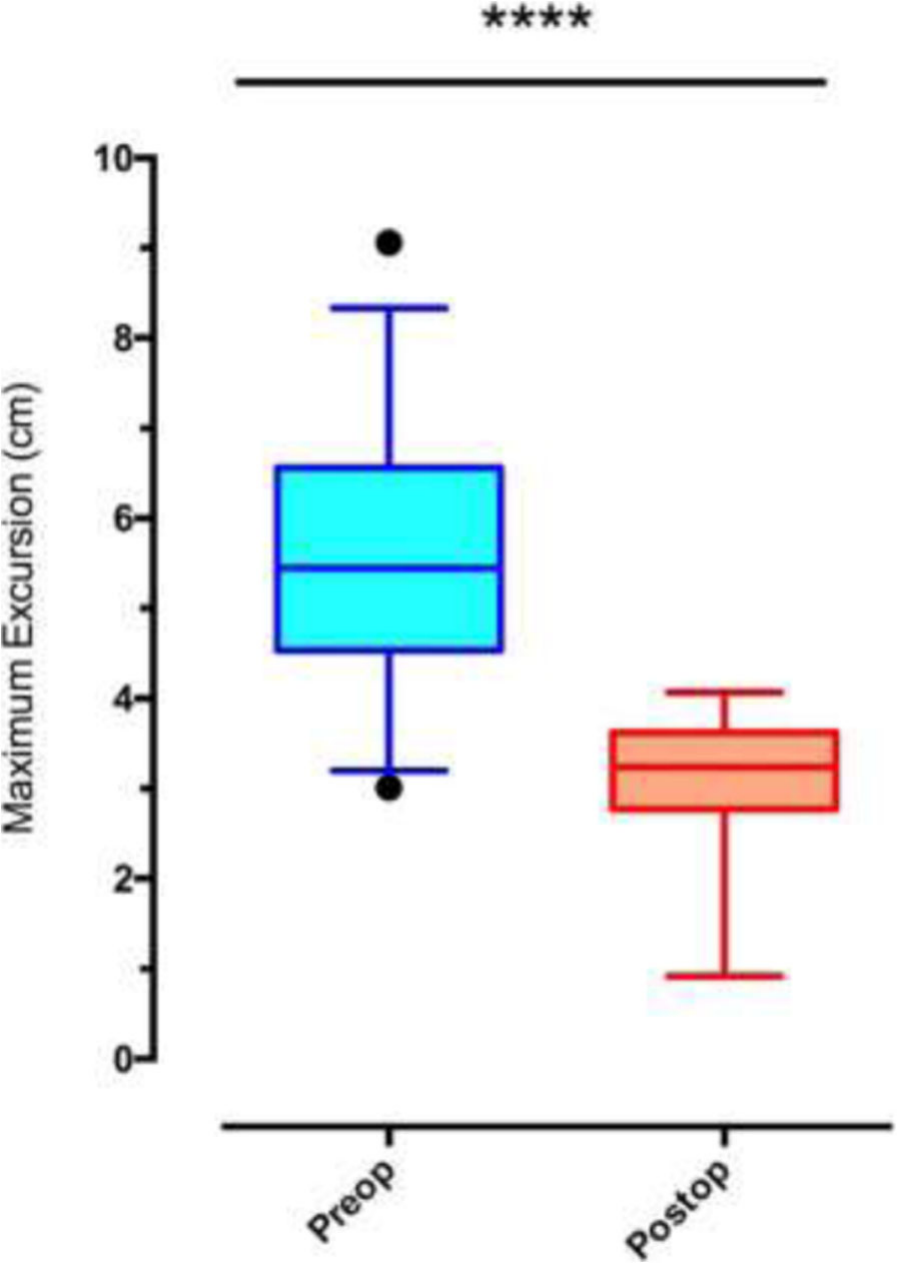
Diaphragmatic excursion at deep breathing

## P215 Esophageal pressure and diaphragm

### A Menis, D Makris, V Tsolaki, E Zakynthinos

#### University Hospital of Larisa, Larissa, Greece

**Introduction:** Proper diaphragmatic function is of great importance for ICU patient under mechanical ventilation in order to facilitate the weaning process.

**Methods:** We conducted a physiological study to correlate information derived from esophageal pressure measurements with measurements of diaphragmatic thickness in patients ventilated on a Pressure support mode, breathing through a T-piece tube and a situation of resistive breathing

**Results:** We studied 12 patients hospitalized in the Intensive Care Unit of the University Hospital of Larisa. During the Pressure Support mode we found a positive correlation between lung compliance and diaphragmatic thickness during inspiration and expiration (r: 0.842, p=0.001 and r: 0.777, p=0.003 respectively) and also between Diaphragmatic thickness and Tidal volume (inspiration r: 0.650, p=0.022 and expiration r: 0.680, p=0.015). Transdiaphragmatic pressure generated when the patients on a T tube was correlated with Diaphragmatic thickness during inspiration and expiration (r:0.550, p=0.001 and r:0.471, p=0.004, respectively). The same was found between diaphragmatic thickness and the Tidal Volume (inspiration r:0.539, p<0.001, expiration r:0.465, p=0.004). Tidal Volume also correlated with Diaphragmatic displacement during inspiration (r: 0.463, p=0.004). During resistive breathing, both diaphragmatic thickness during inspiration and expiration were positively correlated with the generated tidal volume (r: 0.358 p=0.010 and r: 0.454, p=0.001, respectively) and so was the diaphragmatic displacement during inspiration (r:0.533, p<0.0001).

**Conclusions:** In this study we found that the result of diaphragmatic function, meaning transdiaphragmatic pressure and the generated tidal volume, can be assessed focusing on the changes of the diaphragmatic thickness during the respiratory cycle.

**Reference**

1. Supinski G et al. Chest, 2017

## P216 The relationship between transpulmonary pressure, end expiratory lung volume and intraabdominal pressure in patients with respiratory and intraabdominal pathologies

### D Diktanaite^1^, A Kalenka^2^, M Fiedler^1^, E Simeliunas^1^

#### ^1^University of Heidelberg, Heidelberg, Germany; ^2^Hospital Bergstrasse, Heppenheim, Germany

**Introduction:** Measuring transpulmonary pressure (P_L_), end expiratory lung volume (EELV) and intraabdominal pressure (IAP) is a new approach for setting parameters in mechanical ventilation. We analyzed the relationship between these parameters in two challenging patient groups: group 1 with ARDS or severe pneumonia and group 2 with intraabdominal hypertension or BMI>45.

**Methods:** Intensive care patients at the hospital Bergstrasse (Heppenheim, Germany), requiring mechanical ventilation>72h, were included in the study. Esophageal pressure (Pes), EELV and IAP were measured daily at 3 PEEP levels: 15, 10 and 5. Pes was measured via nasogastric catheter with an esophageal balloon, EELV – by nitrogen washout method and IAP as bladder pressure through a bladder catheter. Patients were in supine position and under full muscle relaxation. Tidal volume – 6ml/kg. P_L_ and chest wall elastance (Ecw) were calculated using the elastance-derived method [1].

**Results:** 18 Patients (N=18) were included in the study (N1=9; N2=9). In 53 measurements (N1=25; N2=28) there was no difference between groups regarding EELV (p>0.05). At PEEP of 5, patients in group 2 had higher Pinsp, Pes and IAP (Pinsp22±4 vs 18±2, p<0.01); (Pesmin 11.8±4 vs 9.9±3.5, p=0.04); (IAP 11.1±3 vs 8.7±3, p<0.01) Table 1. There was a strong positive correlation between EELV and Cstat at PEEP 5 (R=0.7, p=0.02), as well as Cstat and end expiratory PL (PLexp) (R=0.6, p<0.01) [Fig. 1]. In group 2 we saw a strong correlation between IAP and Ecw (R=0.6, p<0.01) and mild correlation between IAP and Cstat (R=0.4, p=0.02) [Fig. 2].

**Conclusions:** Higher transpulmonary end expiratory pressure ensures better Cstat. In patients with intraabdominal pathology, the chest wall elastance strongly depends on the intraabdominal pressure.

**Reference**

1. Chiumello D et al. Am J Respir Crit Care Med 178:346-55, 2008

**Table 1 (abstract P216).** Differences between groups, showed in mean values ± standard deviation

PEEP 5	group 1	group 2	P value
EELV	1226±547	1271±519	0.391
Cstat	55 ±21	52 ±18	0.062
Pinsp	18.2 ±2.8	22.3 ±4.5	0.001
Pesmin	9.9 ±3.5	11.8 ±4.0	0.04
Pesmax	13.7 ±3.2	16.9 ±4.3	0.002
Δ PL	9.5 ±2.9	10.7 ±4.8	0.01
IAP	8.7 ±3.2	11.1 ±3.3	0.007

**Fig. 1 (abstract P216).**
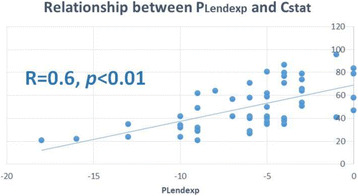
A strong correlation between end expiratory transpulmonary pressure (PLendexp) and static compliance (Cstat) at PEEP 5

**Fig. 2 (abstract P216).**
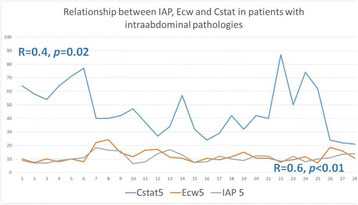
A strong correlation between IAP and Ecw and a mild correlation between IAP and Cstat at PEEP level of 5 in patients with intraabdominal pathologies

## P217 Occlusion test for esophageal manometry: large artifacts caused by fast compression

### C Lima^1^, C Morais^1^, T Yoshida^2^, S Gomes^1^, G Plens^1^, O Ramos^1^, R Roldan^3^, S Pereira^1^, M Tucci^1^, E Costa^1^, M Amato^1^

#### ^1^Heart Institute (InCor), Hospital das Clínicas, Faculdade de Medicina da Universidade de São Paulo, São Paulo, Brazil; ^2^University of Toronto, Toronto, Canada; ^3^Hospital Rebagliati, Lima, Peru

**Introduction:** Pleural pressure (Ppl) is estimated by measuring esophageal pressure (Pes) with an esophageal balloon. To validate the correct position and inflation volume of the balloon, a positive or negative pressure occlusion test is used, considered valid when the ratio between oscillations in Pes (Δ Pes) versus airway-pressure (Δ Paw) are equivalent. We investigated the impact of two different compression/decompression rates during chest compression.

**Methods:** We studied 8 pigs with injured lungs, paralyzed and supine. An esophageal balloon was placed at the lower portion of esophagus and a Ppl sensor (wafer-type, flat balloon) was placed at most dependent lung region. Both devices were inflated with minimal (non-stressed) volume derived from in-vitro and in-vivo PV curves. Two rates of thoracic compression (slow and fast) were tested and recorded at different PEEP levels. Plots of Pes/Paw and Ppl/Paw were fitted into a hysteresis and linear-regression model.

**Results:** 70 repeated measurements were performed in 8 animals (PCV mode, PEEP = 12±3 cmH2O and VT = 6 ml/kg). The compression-time for slow maneuver was 0.49 ± 0.10 s, vs. 0.19 ± 0.04 for the fast one. Plots of Ppl/Paw showed small hysteresis, with phase angles of 1 vs. 3 grad for slow vs. fast maneuvers. In contrast, plots of Pes/Paw showed higher hysteresis, with phase angles of 3 vs. 32 grad (slow vs. fast) (Fig. 1). The slope of the regression line for Pes/Paw plots was consistently higher for slow compressions (0.98 ± 0.08), as compared to fast ones (0.84 ± 0.05). A good agreement between Δ Pes and Δ Paw (Fig. 2) was found during slow maneuvers, but not during the fast ones.

**Conclusions:** Slow chest compressions must be used when checking position/inflation of esophageal balloon. The fast maneuver produces hysteresis and underestimation of Δ Pes (but not in direct Δ Ppl). Pes monitoring at high respiratory rates may be problematic.

**References**

**Fig. 1 (abstract P217).**
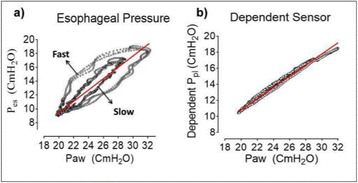
Representative model between slow chest compression with time of velocity 0.61 seconds versus fast chest compression with 0.20 seconds. a) Behavior of hysteresis between Paw with Pes. b) Behavior of hysteresis between Paw and direct Ppl measurement.

**Fig. 2 (abstract P217).**
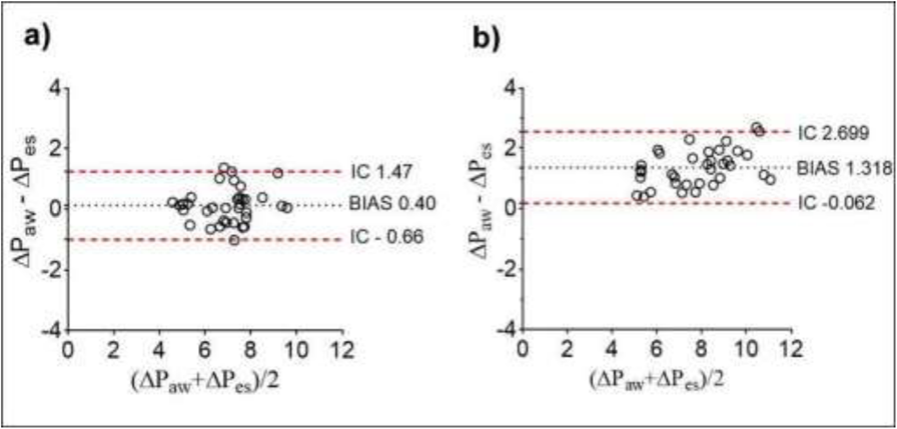
Bland-Altman plots showing the agreement between ∆Pes and ∆Paw in two different strategies: a) slow chest compression and b) fast chest compression

## P218 Electrical impedance tomography guided ventilation

### R Knafelj, M Noc, V Gorjup

#### University Medical Center Ljubljana, Ljubljana, Slovenia

**Introduction:** In protective ventilation strategy tidal volume of 6 mL/kg IBW, PEEP to prevent tidal derecruitment, driving pressure <15 cm H2O, plato pressure <28 cm H2O and FiO2 <0.6 is used. While PEEP can be set individually using techniques like quasi static pressure-volume (PV) curve, expiratory transpulmonary pressure (PtPEEP) and electrical impedance tomography (EIT), real time visual bed-side techniques to determine overdystension is possible by EIT only. We compared different strategies for PEEP optimization during protective ventilation approach in comatose patients admited after cardiac arrest with no previous known pulmonary diseases and screened for inspiratory overdystension

**Methods:** 20 consecutive comatose post cardiac arrest patients were ventilated with volume assist ventilation (6 mL/kg IBW, PEEP 5 cm H2O) using Elisa 800EIT (Lowenstein Medical, GE). Orogastric tube (NutriVent, Sidam, IT) was inserted, and EIT vest (swisstom AG, CH) was applied in all patients. Measurements were performed 60 min after admission and after 3 hrs (Fig. 1). Optimal PEEP was defined as lower inflection point using PV curve (PV), positive PtPEEP (Ptp) and optimal Regional Stretch/Silent Spaces (EIT)

**Results:** Methods to determine PEEP using PV, Ptp and EIT were comparable in non obese patients (p=NS). Measures after 180 min were consistent with the first measures. PEEP set initially was too low in 7 patients and too high in 5 patients. When highest PEEP (Ptp, EIT, PV) was selected, anterior hyperinflation was present in 3 patients. In 2 obese patients (BMI 34 and 36) PV suggested lower PEEP, while EIT and Ptp measures suggested higher PEEP (6 vs 12 and 14 respectively), p=0.02

**Conclusions:** Using protective ventilation strategy in comatose post cardiac arrest patients with no previous known lung disease does not prevent from anterior hyperinflation in some non-obese patients. PV, EIT and Ptp are comparable methods in determining PEEP in non obese patients, while in obese patients EIT was superior

**Reference**

CritCare 2017;21:183

**Fig. 1 (abstract P218).**
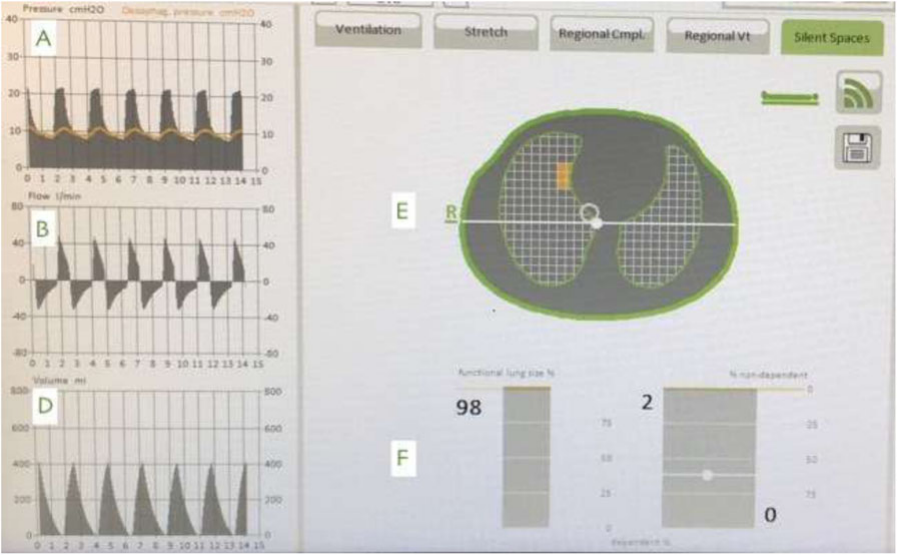
A Ptp, B Flow, C Vt tracing, E, F Silent Spaces

## P219 Validity of esophageal pressure measurement by a commercially available ventilator: a comparison with an in vivo calibration method

### M Kyogoku^1^, S Mizuguchi^2^, T Miyasho^3^, Y Endo^3^, Y Inata^4^, T Ikeyama^1^, K Tachibana^4^, K Yamashita^3^, M Takeuchi^4^

#### ^1^Aichi Children’s Health and Medical Center, Obu-shi, Aichi, Japan; ^2^Kyushu University, Fukuoka-city, Fukuoka, Japan; ^3^Rakuno Gakuen University, Ebetsu, Hokkaido, Japan; ^4^Osaka Women’s and Children’s Hospital, Izumi, Osaka, Japan

**Introduction:** Assessment of pleural pressure by measuring esophageal pressure (Pes) is useful to guide optimal ventilator settings in patients with respiratory failure. However, the measurement of Pes is affected by several factors, including filling volume of an esophageal balloon and an esophageal wall elastance. Recently, Mojoli et al. have reported an in vivo calibration method (M method) to make reliable the measurement of both absolute values of Pes (abs Pes) and tidal swings of Pes (Δ Pes). In contrast, the validity of Pes measurement by a commercially available ventilator has not been investigated. Thus, we evaluated the accuracy of abs Pes and Δ Pes measured by AVEA ventilator (CareFusion, Yorba Linda, USA), which regulates a filling volume of balloon automatically and is the only approved equipment to measure Pes in Japan, by comparison with the M method.

**Methods:** Four anesthetized and paralyzed pigs (40.9-42.8 kg) were mechanically ventilated and subjected to lung injury by saline lung lavage. In each pig, two different extrathoracic pressures were applied by using a thoraco-abdominal belt. Abs end-expiratory Pes and Δ Pes were measured by AVEA ventilator and the M method, and obtained values were compared for their correlation and agreement.

**Results:** Comparison of the two methods showed that correlation coefficients of abs end-expiratory Pes and Δ Pes were 0.85 (P = 0.007) and 0.88 (P = 0.004), respectively. By Bland-Altman analysis, the bias and precision of abs end-expiratory Pes were -2.2 and 2.3, and those of Δ Pes were 0.1 and 3.4, respectively (Figs. 1 and 2).

**Conclusions:** Abs end-expiratory Pes tended to be higher in the A method than in the M method. Δ Pes of the two methods were well correlated. In our animal model, the accuracy of Pes measured by AVEA ventilator was clinically acceptable when compared to the M method.

**Fig. 1 (abstract P219).**
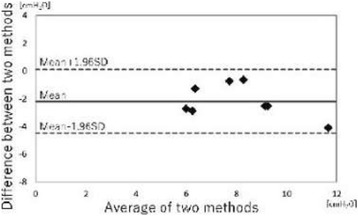
Agreement between the two methods of measuring absolute end-expiratory Pes by Bland-Altman analysis

**Fig. 2 (abstract P219).**
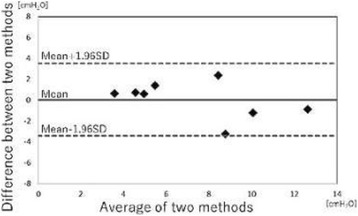
Agreement between the two methods of measuring tidal swings of Pes by Bland-Altman analysis

## P220 Comparison of esophageal pressure and CVP-derived pleural pressure in spontaneously breathing children

### N Okuda^1^, M Takeuchi^2^, M Kyogoku^3^, K Moon^2^, Y Inata^2^, T Hatachi^2^, Y Shimizu^2^

#### ^1^Tokushima University Hospital, Tokushima, Japan; ^2^Osaka Women’s and Children’s Hospital, Osaka, Japan; ^3^Aichi Children’s Health and Medical Center, Aichi, Japan

**Introduction:** Maintaining inspiratory pleural pressure (Ppl) in an optimal range is important to prevent lung injury, respiratory muscle fatigue and ventilator-induced diaphragmatic dysfunction. While esophageal pressure (Pes) has been used as a gold standard surrogate for Ppl, the measurement of Pes has several challenges including correct positioning of an esophageal catheter. We hypothesized that Ppl in spontaneously breathing children during mechanical ventilation can be estimated by using the change in central venous pressure (Δ CVP).

**Methods:** Spontaneously breathing children under mechanical ventilation with acute respiratory failure (PaO2/FIO2 <300), who has a central venous catheter and an esophageal catheter for clinical purposes, were enrolled. Correct positioning of the esophageal balloon catheter was ensured by an occlusion test (OT), in which the changes in Pes and airway pressure (Δ Pes and Δ Paw, respectively) were confirmed to be close to unity. First, we obtained a ratio (k) of Δ Paw (≈Δ Ppl) to Δ CVP during OT. Second, assuming k was the same as the ratio of Δ Ppl to Δ CVP during spontaneous breathing, Δ Ppl during pressure support of 10, 5, and 0 cmH2O was calculated by using Δ CVP as follows: CVP-derived Δ Ppl = k * Δ CVP. Finally, CVP-derived Δ Ppl was compared to Δ Pes at each ventilator setting.

**Results:** Eight patients (Median age 4.8±3.3 months; median body weight 4.7±1.3 kg) were included in the analysis. CVP-derived Δ Ppl and Δ Pes were correlated significantly (y=0.59x+3.6, R2=0.56, p<0.01). Bland-Altman analysis showed that bias and precision of these two methods were -0.53 and 3.5 cmH2O, respectively (Fig. 1).

**Conclusions:** In spontaneously breathing children during mechanical ventilation, CVP-derived Δ Ppl correlated well with Δ Pes. It could be used as a guide to estimate pleural pressure without using an esophageal balloon catheter.

**Fig. 1 (abstract P220).**
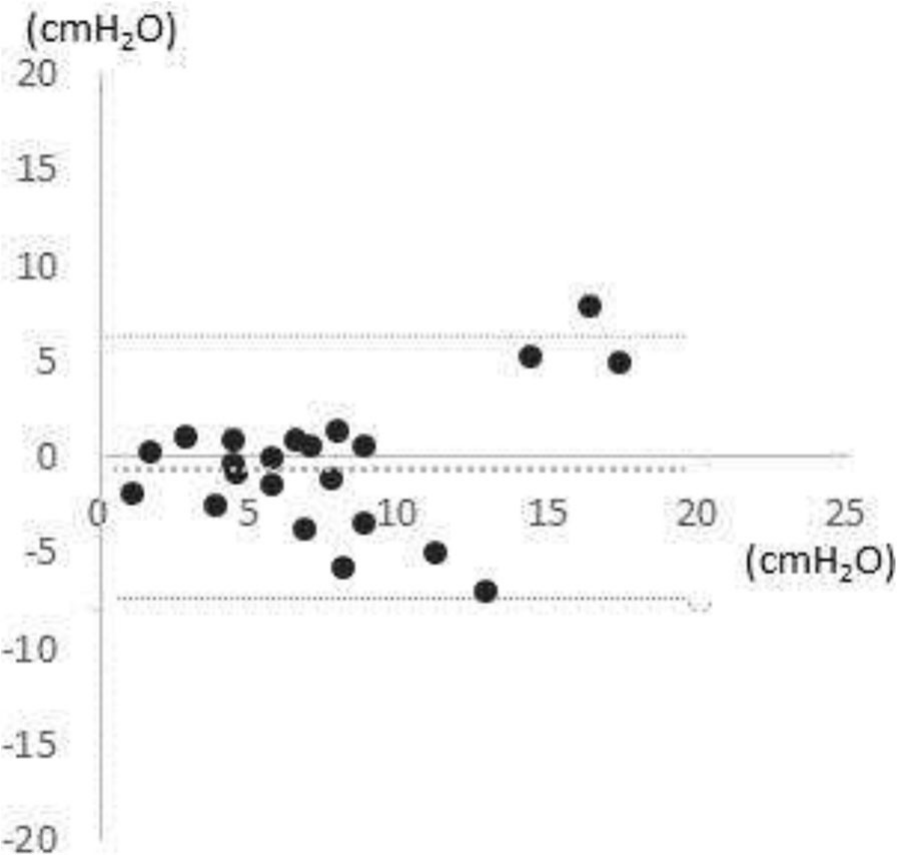
Bland-Altman analysis demonstrated that CVP-derived ΔPpl and ΔPes were correlated significantly

## P221 Driving pressure during assisted ventilation: an observational study

### A Proklou^1^, E Akoumianaki^1^, E Pediaditis^1^, C Psarologakis^1^, E Koutsiana^2^, I Chouvarda^2^, E Kondili^1^, D Georgopoulos^1^, K Vaporidi^1^

#### ^1^University Hospital of Heraklion, Heraklion, Greece; ^2^Medical School, Aristotle University of Thessaloniki, Thessaloniki, Greece

**Introduction:** The driving pressure of respiratory system (DP) reflects the extent of lung stretch during tidal breathing, and has been associated with mortality in ARDS patients during controlled mechanical ventilation [1]. Aim of this study was to examine DP during assisted ventilation, and examine if and when high DP occurs in patients in assisted ventilation with PAV+.

**Methods:** Critically ill patients hospitalized in the ICU of the University Hospital of Heraklion, on mechanical ventilation in PAV+ mode were studied. Continuous recordings of all ventilator parameters were obtained for up to three days using a dedicated software. DP was calculated from the PAV+ computed compliance (C) [2], and the measured exhaled tidal volume (VT, DP=VT/C). Periods of sustained DP above 15 cmH2O were identified, and ventilation and clinical variables were evaluated.

**Results:** Sixty-two patients and 3200 hrs of ventilation were analyzed. In half of the patients, DP was lower than 12 cmH2O in 99% of the recording period, while high-DP (>15cmH2O) more than 10% of the total time was observed in 10% of patients. ICU non-survivors had more time with high DP than survivors (p=0.04). Periods of sustained high-DP (>15cmH2O for >1h) were observed in 9 patients. Level of assist, minute ventilation, and respiratory rate were not different between the periods of high DP and the complete recordings, while VT was higher and C was lower during the high-DP period compared to the complete recording. The median compliance was below 30 ml/cmH2O during the high-DP period, and above 50 ml/cmH2O during the complete recording.

**Conclusions:** High DP is not common, but does occur during assisted ventilation, predominantly when compliance is below 30 ml/cmH2O, and may be associated with adverse outcome.

**References**

[1] Amato MB et al. N Engl J Med. 372(8):747-55, 2015

[2] Younes M et al. Am J Respir Crit Care Med. 164(1):50-60, 2001

## P222 Comparison of respiratory volume monitoring vs. capnography during intravenous propofol-based anesthesia

### JE Freeman^1^, S Pentakota^2^, E Blaney^2^, B Kodali^2^

#### ^1^Respiratory Motion, inc., Waltham, MA, USA; ^2^Brigham and Women’s Hospital, Boston, MA, USA

**Introduction:** Capnography (EtCO2) is the current standard of care for monitoring ventilation in patients under general anesthesia. However, in non-intubated patients, EtCO2 monitoring is challenging and clinicians therefore often rely on pulse oximetry, a late indicator of respiratory depression, or on subjective assessment. A noninvasive respiratory volume monitor (RVM) provides accurate and continual monitoring of minute ventilation (MV), tidal volume (TV) and respiratory rate (RR). Here, we compared RVM and EtCO2 monitoring in patients receiving propofol-based sedation.

**Methods:** In an observational study approved by Partners IRB, simultaneous data were recorded by an RVM (ExSpiron, Respiratory Motion, Inc.) and capnography (Capnostream 20, SmartCapnoLine, Covidien) during colonoscopy procedures. Baseline MV, TV, and EtCO2 were established prior to sedation. Periods of High EtCO2 (>50mmHg or >130%Baseline), Low EtCO2 (<20mmHg or <70%Baseline), and Low MV (<40%Baseline) were identified. The number of High EtCO2 and Low EtCO2 events that were preceded by Low MV events was quantified.

**Results:** 50 patients (22 males, 52±16years; 26.1±6.3kg/m2) were monitored for 33.2±14.7min. Table 1 summarizes the percent of monitored time with reported data for the two devices. Figure 1 depicts MV decrease following propofol and cannula dislodgement following a jaw thrust. Table 2 presents the number of EtCO2 events across all patients. Low MV events preceded all 9 High EtCO2 events by 7.7min. Low MV events preceded 43 out of 47 of the Low EtCO2 events by 2.9min. The 4 Low EtCO2 events that did not correspond to a change in MV were potentially false alarms.

**Conclusions:** In non-intubated patients, the RVM identified all High EtCO2 events and detected the decrease in MV 7.7min before EtCO2. The RVM provides reliable measurements when capnography data is unavailable and is not subject to the EtCO2 limitations of nasal cannula placement, dilution with O2, and mouth breathing.

**Table 1 (abstract P222).** Percent of monitored time with data reported

		RVM	Capnography
% Time with Data	MV/EtCO2	99.2 ± 1.3%	89.5 ± 19.7%
% Time with Data	RR	99.2 ± 1.3%	89.5 ± 19.7%

**Table 2 (abstract P222).** Low Minute Ventilation Events and EtCO2 Alarms

	High EtCO2	Low EtCO2
Alarm criteria	EtCO2 > 50 mmHg or EtCO2 > 130% Baseline EtCO2	EtCO2 < 20 mmHg or EtCO2 < 70% Baseline EtCO2
# of Alarms	13	47
# of Alarms with corresponding LMVe	13	43
Delay between LMVe and EtCO2 Alarm	7.7 ± 2.7 min	2.9 ± 0.6 min

**Fig. 1 (abstract P222).**
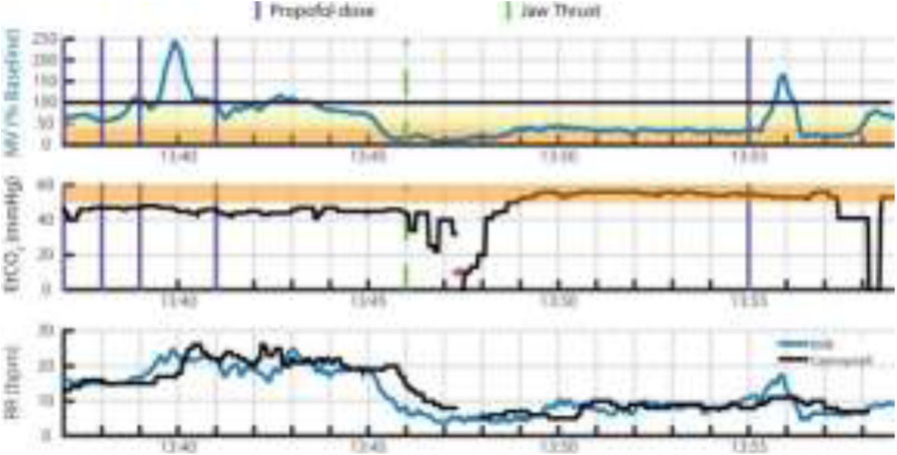
MV (top) decreases in response to propofol (purple), resulting in an LMVe at 13:45. EtCO2 (middle) stays relatively constant at the start of the LMVe without triggering either high or low EtCO2 alarms. A jaw thrust (13:46), results in dislodged nasal canula. MV increased slightly, but still remained low (40% MVBaseline). EtCO¬2 increased to >50 mmHg and remained high

## P223 Evaluation of the negative arterial to end-tidal co2 pressure gradient as a predictor of survival in acute respiratory failure

### C Ko^1^, M Yeh^1^, Y Chen^1^, C Chen^2^

#### ^1^Chang Gung Memorial Hospital, Yulin, Taiwan; ^2^National Chung Hsing University, Taichung, Taiwan

**Introduction:** Measurement of CO2 concentrations in the expired air directly indicates changes in the elimination of CO2 from the lungs. In a healthy person, end tidal CO2 (elimination of PCO2 from alveolar) is lower than PaCO2 (arterial PCO2) by 2-5 mmHg. The Arterial to end-tidal CO2 pressure gradient [(a-ET) PCO2]is dependent on the amount of alveolar dead space. Negative (a-ET) PCO2 values were observed with increased cardiac output and increased CO2 production, reduced FRC and low compliance. Thus, (a-ET) PCO2 monitoring can be used as a simple index of pulmonary blood flow.The objective of this study was to investigate the association between (a-ET) PCO2 and mortality in patients with acute respiratory failure.

**Methods:** We retrospective studied 197 intubated patients undergoing mechanical ventilation due to acute respiratory failure patients between 2014 and 2015 in Chang Gung Memorial Hospital. PaCO2 to end-tidal CO2 pressure gradient with clinical data and outcomes were measured after admission.

**Results:** Forty patients with negative (a-ET) PCO2 values had lower mortality, lower FiO2 setting, lower respiratory rate and higher blood pressure (Table 1). Odds ratios (95% confidence intervals) associated with Age(<65yr), gender(male), PaO2/FiO2(<300) and minute volume(>8L/min) were 0.078(0.010-0.624), 0.148(0.042-0.521), 0.089(0.011-0.701) and 0.229(0.075-0.698), respectively (Table 2). Negative (a-ET) PCO2 was strongly associated with good outcome and were significantly associated with overall survival (Fig. 1)

**Conclusions:** In conclusion, the negative arterial to end-tidal CO2 pressure gradient may predict patient survival in some subgroups.

**Table 1 (abstract P223).** Comparison of patients who Negative (a-ET) PCO2 values with Positive (a-ET) PCO2 values

	Negative value	Positive value	p value
Age	67.95±18.57	67.07±16.83	NS
Mortality(%)	6/40(15)	66/157 (42)	.001
FiO2 settting	69.43±14.67	80.87±16.98	.001
Respiratory Rate	18.77±3.73	20.27±4.1	.036
Mean artery pressure	99.01±20.05	89.19±22.2	.013
PEtCO2	43.35±14.49	32.75±10.64	.001
PaO2	234.07±111.27	170.67±131	.005

**Table 2 (abstract P223).** Associations between negative (a-et) pco2 values and mortality in subgroups

	Negative value	Positive value	Odds ratio (95% CI)	p value
	[no. of deaths/no. of patients (%)]	[no. of deaths/no. of patients (%)]		
All patients	6/40(15)	66/157 (42)	0.243(0.097-0.613)	0.001
Age<65	1/16(6.3)	30/65(46.2)	0.078(0.010-0.624)	0.003
Male	3/28(10.7)	47/105(44.8)	0.148(0.042-0.521)	0.001
Female	6/12(50)	22/52(42.3)	0.579(0.139-2.403)	NS
PaO2/FiO2<300	1/16(6.3)	47/110(42.7)	0.089(0.011-0.701)	0.003
Minute volume>8	4/29(13.8)	49/119(41.2)	0.229(0.075-0.698)	0.004

**Fig. 1 (abstract P223).**
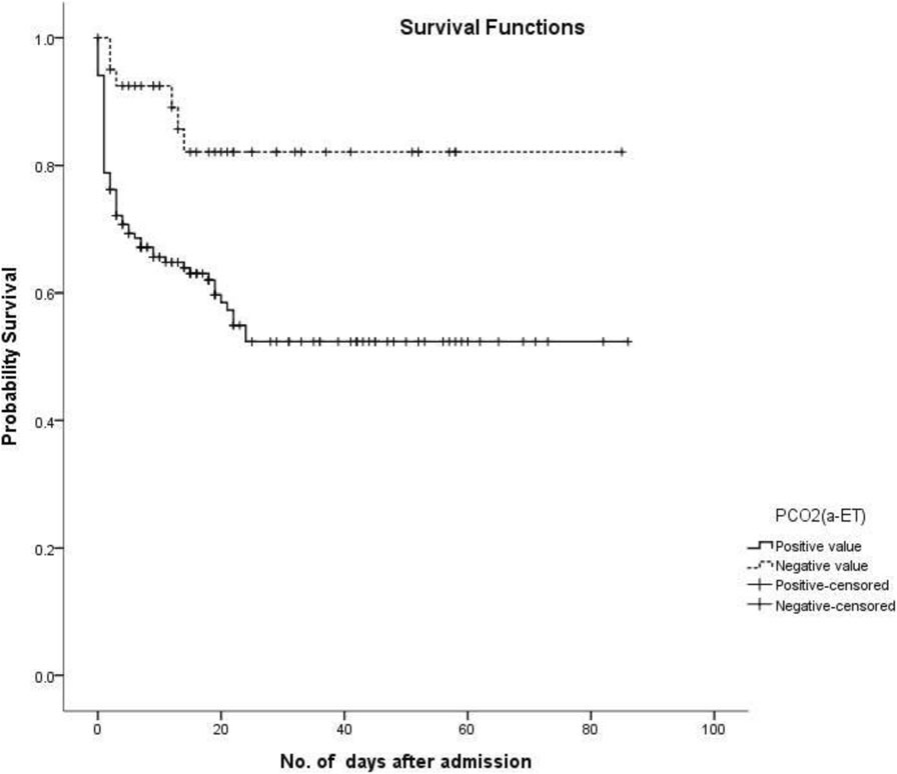
Kaplan-Meier survival curves of patients

## P224 Predictive indexes in the extubation success or failure: role of the expiratory peak flow

### IR Porto, MM Silva, RS Zaponi, JL Jaskowiak, LR Abentroth, BA Kanezawa, ER Penteado, PS Quadros, TC Schnaufer, EF Osaku, CR Costa, SM Ogasawara, MA Leite, AC Jorge, P Duarte

#### Hospital Universitario do Oeste do Parana, Cascavel, Brazil

**Introduction:** Cough efficiency may be an important factor at weaning, and expiratory Peak Flow (ePF) could be a marker of risk for extubation failure. Objective: To verify the relationship between predictive indexes and extubation failure in mechanically ventilated patients, analyzing specifically the role of the ePF.

**Methods:** Retrospective study, conducted from January to December 2016, in a 14-beds General Intensive Care Unit (ICU), from a University Hospital. Predictive indexes, such as Maximal Inspiratory Pressure (MaxIP), Rapid Shallow Breathing Index (RSBI) and ePF, were collected on the day of extubation of adult patients with MV >24h (excluded tracheostomized). For statistical analysis, the data were described by mean, standard deviation and percentage. Pearson’s Chi-square test was used for correlation, adopting p <0.05.

**Results:** It was included 154 patients. Most commom causes of admission: medical non-neurological (33%), elective postoperative (20%), and Traumatic Brain Injury (TBI) (14%); mean APACHE II 25.6, 1st day SOFA 9.6, mean age 48.2y, 58% female. The duration of MV and sedation were 123.2 and 57.3 hours, respectively. ICU and hospital length of stay were 10.7 and 25.1 days. Mean MaxIP was 28.0 ± 14.22 mmHg, RSBI 58.7 ± 35.76, and ePF 57.6 ± 33.86 L/min. Among the patients included in the study, 99% were extubated and of these, 12% had extubation failure. There was a correlation between MaxIP and ePF (p < 0.001), and extubation failure was associated with MaxIP (p<0.001) and ePF (p < 0.001).

**Conclusions:** MaxIP and ePF (collected at the extubation day) were correlated with extubation failure, demonstrating that they are reliable indices to predict extubation failure in adult MV patients.

## P225 Expression of proinflammatory and fibrogenetic biomarkers in ards: a case-series of 4 patients

### M Cozzolino^1^, M Del Re^2^, E Rofi^2^, G Rombolà^1^, A Franci^1^, M Bonizzoli^1^, R Danesi^2^, A Peris^1^

#### ^1^A.O.U. Careggi, Florence, Italy; ^2^University Hospital, Pisa, Italy

**Introduction:** ARDS may result from various diseases and is characterized by diffuse alveolar injury, lung edema formation, neutrophil-derived inflammation and surfactant dysfunction. Various biomarkers have been studied in diagnostics and prognostication of ARDS. The purpose of the study was to measure the expression of proinflammatory mediators like IL-8 and TNF, a cellular receptor with a role in innate immunity(TLR-2),and a biomarker of fibrogenesis (MMP-7) in different phases of ARDS patients.

**Methods:** We studied 4 patients admitted to our ICU with diagnosis of ARDS during the month of January 2016. Six ml of blood were prospectively collected at two times: during the acute phase and in a sub-acute phase before ICU discharge. Blood samples were centrifuged to obtain the platelet-rich plasma and plasmatic RNA (cRNA) was isolated from platelets.IL-8, TNF, TLR-2 and MMP-7 expression in cRNA was determined by the Droplet Digital™ PCR as copies/ml.

**Results:** All patient showed a decrease in IL-8, TNF, TLR2 and MMP-7 levels after the acute phase of ARDS (Fig. 1). Patient 1 and 3 were affected by influenza A virus (H3N2), patient 2 was admitted for pneumococcal pneumonia and patient 4 was affected by Legionella. Adequate ethiologic treatment was promptly started in patients with bacterial infection. Mean duration of mechanical ventilation was 17.5 days. All patient survived ICU stay and were discharged from hospital.

**Conclusions:** IL-8, TNF, TLR-2 and MMP-7 expression detected by extracted platelets RNA, may be a novel tool useful for clinicians indicating persistent inflammation with resulting progressive alveolar fibrosis and impaired lung function. More data are necessary to understand the real clinical significance of this biomarkers and their role in fibroproliferation and progression of ARDS.

**Fig. 1 (abstract P225).**
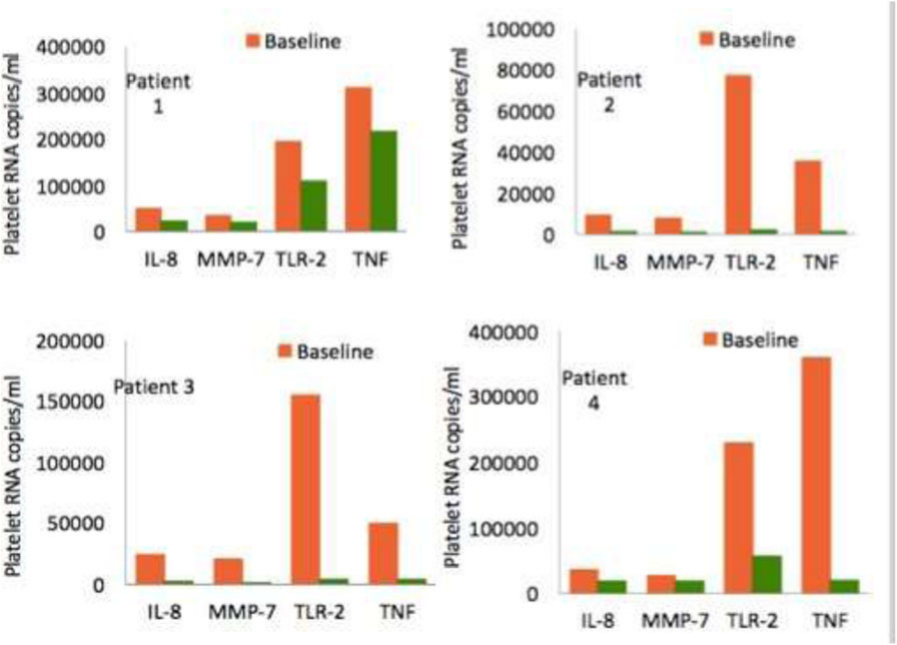
Biomarkers expression (copies/ml)

## P226 Genetic modification of mesenchymal stem cells overexpressing angiotensin II type 2 receptor increase lung engraftment in LPS-induced acute lung injury mice

### X Xu^1^, LL Huang^1^, LL Huang^1^, SL Hu^1^, JB Han^1^, HL He^2^, JY Xu^1^, JF Xie^1^, AR Liu^1^, SQ Liu^1^, L Liu^1^, YZ Huang^1^, FM Guo^1^, Y Yang^1^, HB Qiu^1^,

#### ^1^Nanjing Zhongda Hospital, School of Medicine, Southeast University, Nanjing, China; ^2^Affiliated Hospital of University of Electronic Science and Technology of China & Sichuan Provincial People’s Hospital, Chengdu, China

**Introduction:** Although mesenchymal stem cells (MSCs) transplantation has been shown to promote lung respiration in acute lung injury (ALI) in vivo, its overall restorative capacity appears to be restricted mainly because of low engraftment in the injured lung. Ang II are upregulated in the injured lung. Our previous study showed that Ang II increased MSCs migration in an Angiotensin II type 2 receptor (AT2R)-dependent manner [1]. The objective of our study was to determine whether overexpression of AT2R in MSCs augments their cell migration and engraftment after systemic injection in ALI mice.

**Methods:** A human AT2R expressing lentiviral vector was constructed and introduced into human bone marrow MSCs. We also down-regulated AT2R mRNA expression using a lentivirus vector carrying AT2R shRNA to transduce MSCs. The effect of AT2R regulation on migration of MSCs was examined in vitro. A mouse model of lipopolysaccharide (LPS) induce ALI was used to investigate the engraftment of AT2R-regulated MSCs and the therapeutic potential in vivo.

**Results:** Overexpression of AT2R dramatically increased Ang II-enhanced human bone marrow MSC migration in vitro. Moreover, MSC-AT2R accumulated in the damaged lung tissue at significantly higher levels than control MSCs 24h and 72h after systematic MSC transplantation in ALI mice. Furthermore, MSC-AT2R-injected ALI mice exhibited a significant reduction of pulmonary vascular permeability and improved the lung histopathology and had additional anti-inflammatory effects. In contrast, there were less lung engraftment in MSC-ShAT2R-injected ALI mice compared with MSC-Shcontrol after transplantation. Thus, MSC-ShAT2R-injected group exhibited a significant increase of pulmonary vascular permeability and resulted in a deteriorative lung inflammation.

**Conclusions:** Our results demonstrate that overexpression of AT2R enhance the migration and lung engraftment of MSCs in ALI mice and may provide a new therapeutic strategy for the injured lung.

**Reference**

1. Xu XP et al. Stem Cell Res Ther 8:164, 2017.

## P227 Hepatocyte growth factor protects against lipopolysaccharide-induced endothelial barrier dysfunction with Akt/mTOR/STAT-3 pathway

### S Meng, F Guo, X Zhang, W Chang, H Qiu, Y Yang

#### Department of Critical Care Medicine, Zhongda Hospital, School of Medicine, Southeast University, Nanjing, China

**Introduction:** Reorganization of endothelial barrier complex is critical for increased endothelial permeability implicated in the pathogenesis of acute respiratory distress syndrome. We have previously shown hepatocyte growth factor (HGF) reduced lipopolysaccharide (LPS)-induced endothelial barrier dysfunction. However, the mechanism of HGF in endothelial barrier regulation remains to be unclear.

**Methods:** Recombinant murine HGF with or without mTOR inhibitor rapamycin were introduced on mouse pulmonary microvascular endothelial cells (PMVECs) barrier dysfunction stimulated by LPS. Then, endothelial permeability, adherent junction protein (occludin), endothelial injury factors (Endothelin-1 and von Willebrand factor), cell proliferation and mTOR signaling associated proteins were tested.

**Results:** Our study demonstrated that HGF decreased LPS-induced endothelial permeability and endothelial cell injury factors, and attenuated occludin expression, cell proliferation and mTOR pathway activation.

**Conclusions:** Our findings highlight activation Akt/mTOR/STAT-3 pathway provides novel mechanistic insights into HGF protective regulation of LPS-induced endothelial permeability dysfunction.

**Fig. 1 (abstract P227).**
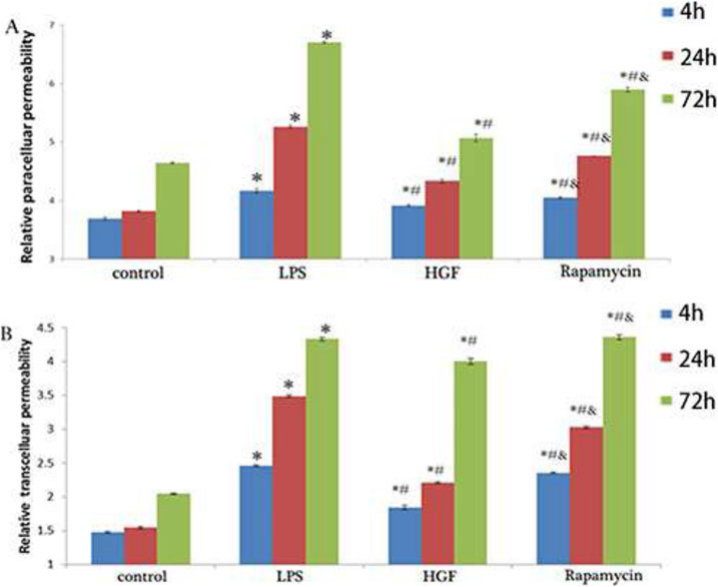
Fluorescein isothiocyanate-Dextran or fluorescein isothiocyanate-BSA analysis of the effect of HGF on PMVECs permeability

**Fig. 2 (abstract P227).**
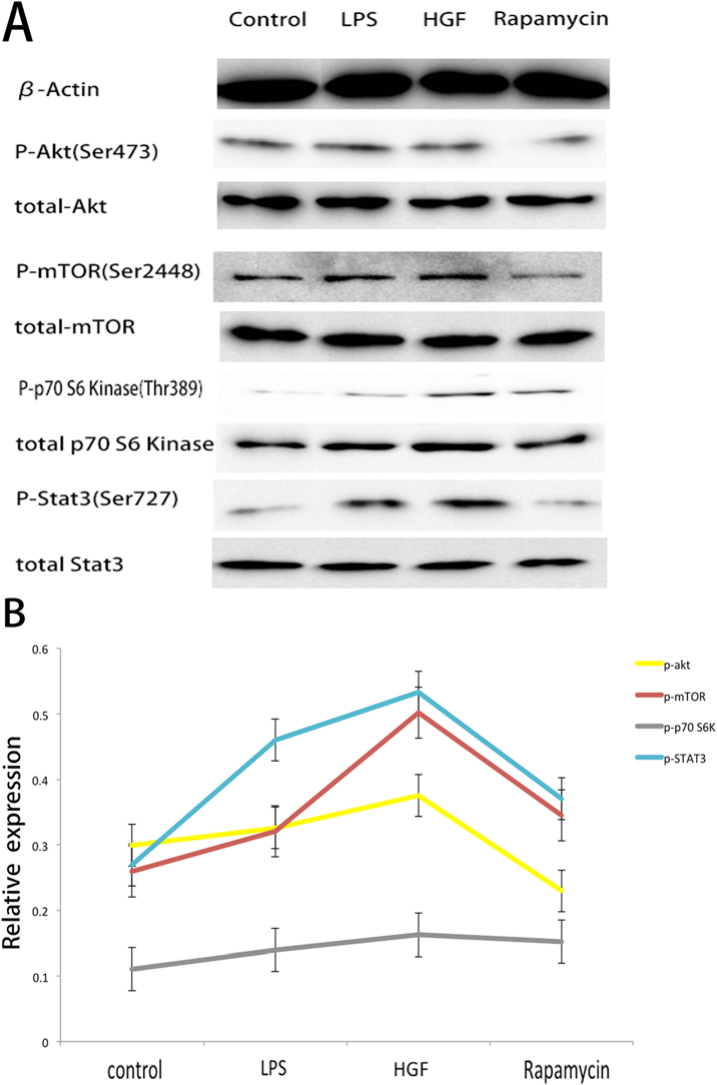
Western blot analysis of HGF on mTOR signaling pathway

## P228 The circadian clock protein BMAL1 regulates the severity of ventilator-induced lung injury in mice

### M Felten, LG Teixeira-Alves, E Letsiou, HC Müller-Redetzky, N Suttorp, A Kramer, B Maier, M Witzenrath

#### University Medicine Charité, Berlin, Germany

**Introduction:** Mechanical ventilation (MV) is a life-saving intervention for critically ill patients, but may also exacerbate pre-existing lung injury, a process termed ventilator-induced lung injury (VILI). Interestingly, we discovered that the severity of VILI is modulated by the circadian rhythm (CR). In this study, we are exploring the role of the myeloid BMAL1, a core clock component, in VILI.

**Methods:** We employed mice lacking Bmal1 in myeloid cells (LyzMCre-Bmal1-/-) and LyzMCre mice as controls. At circadian time (CT) 0 or CT12, mice were subjected to high tidal volume MV to induce VILI. Lung compliance, pulmonary permeability, neutrophil recruitment, and markers of pulmonary inflammation were analyzed to quantify VILI. To assess neutrophil inflammatory responses in vitro, myeloid cells from bone marrow of WT and Bmal1-deficient animals were isolated at dawn ZT0 (Zeitgeber time 0) and dusk (ZT12), incubated with DCFH-DA and stimulated for 15 min with PMA or PBS. Neutrophil activation (Ly6G/CD11b expression) and ROS production (DCFH-DA/Ly6G+ cells) were quantified.

**Results:** Injurious ventilation of control mice at CT0 led to a significant worsening of oxygenation, decrease of pulmonary compliance, and increased mortality compared to CT12. LyzMCre-Bmal1-/- mice did not exhibit any significant differences when subjected to MV at CT0 or CT12. Mortality in LyzMCre-Bmal1-/- mice after VILI was significantly reduced compared to LyzMCre controls (CT0). Neutrophils isolated from control mice at ZT0 showed a significantly higher level of activation and increased ROS production after PMA-stimulation compared to ZT12. ROS production of LyzMCre-Bmal1-/- neutrophils did not differ from ZT0 to ZT12.

**Conclusions:** The lack of the clock gene Bmal1 in myeloid cells leads to increased survival after injurious ventilation and to loss of circadian variations in neutrophil ROS production. This suggests that the internal clock in myeloid cells is an important modulator of VILI severity.

## P229 Hemodynamic resuscitation with fluids bolus and norepinephrine increases severity of the lung damage in an experimental model of septic shock

### P Guijo Gonzalez^1^, MI Monge García^1^, MA. Gracia Romero^1^, A Gil Cano^1^, M Cecconi^2^

#### ^1^Jerez Hospital SAS, Jerez de la Frontera, Spain; ^2^St. George’s Healthcare NHS Trust, London, UK

**Introduction:** Hemodynamic resuscitation by means of fluids and norepinephrine (NE) is currently considered as a cornerstone of the initial treatment of septic shock. However, there is growing concern about the side effects of this treatment. The aim of this study was to assess the relationship between the hemodynamic resuscitation and the development of the ARDS.

**Methods:** 18 New Zealand rabbits. Animals received placebo (SHAM=6) or lipopolysaccharide (LPS) with or without (EDX-R, n=6; EDX-NR, n=6) hemodynamic resuscitation (fluids: 20 ml/kg of Ringer’s lactate; and later NE infusion titrated up to achieve theirs initial arterial pressure). Animals were monitored with an indwelling arterial catheter and an esophageal Doppler. Respiratory mechanics were continuously monitored from a side-stream spirometry. Pulmonary edema was analyzed by the ratio between lung wet and lung dry weight (W/D), and the histopathological findings.

**Results:** SHAM group did not show any hemodynamic or respiratory changes. The administration of the LPS aimed at increasing cardiac output and arterial hypotension. In the LPS-NR group, animals remained hypotensive until the end of experiment. Infusion of fluids in LPS-R group increased cardiac output without changing arterial blood pressure, while the norepinephrine reversed arterial hypotension. Compared to the LPS-NR group, the LPS-R group had more alveolar neutrophils and pneumocytes with atypical nuclei, thicker alveolar wall, non-aerated pulmonary areas and less lymphocyte infiltrating the interstitial tissue. In addition, the airway pressure increased more in the group LPS-R, and the W/D, although slightly higher in the LPS-R, did not show significant differences.

**Conclusions:** In this model of experimental septic shock resuscitation with fluid bolus and norepinephrine increased cardiac output and normalized blood pressure but worsened lung damage.

## P230

### Withdrawn

## P231 Titration of positive end-expiratory pressure in severely obese patients with acute respiratory distress syndrome

### J Fumagalli^1^, R De Santis Santiago^2^, M Teggia Droghi^2^, G Larson^2^, S Kaneki^2^, S Palma^2^, D Fisher^2^, M Amato^3^, R Kacmarek^2^, L Berra^2^

#### ^1^Fondazione IRCCS Cà Granda Ospedale Maggiore Policlinico, Milano, Italy; ^2^ Massachusetts General Hospital, Boston, USA,^3^University of São Paulo, São Paulo, Brazil

**Introduction:** The selection of positive end-expiratory pressure (PEEP) in acute respiratory distress syndrome (ARDS) patients is an ongoing debate. Obese patients have been excluded from most of the clinical trials testing the effects of PEEP in ARDS. We hypothesized that in morbidly obese patients the massive load of the abdomen/chest further increases lung collapse thus aggravating the severity of respiratory failure due to ARDS.

**Methods:** We performed a clinical crossover study to investigate the contribution of lung collapse to the severity of respiratory failure in ARDS obese patients and to determine the specific contribution of titrated PEEP levels and lung recruitment to changes in lung morphology, mechanics and gas exchange. Patients were studied at the PEEP (PEEPICU) levels selected at our institution and at PEEP levels establishing a positive end-expiratory transpulmonary pressure (PEEPINC) and at PEEP levels determining the lowest lung elastance during a decremental PEEP (PEEPDEC) trial following RM.

**Results:** Thirteen patients were studied. At PEEPICU end-expiratory transpulmonary pressure was negative, lung elastance was increased and hypoxemia was present (Table 1). Regardless the titration technique there was no difference in the PEEP level obtained. At PEEPINC level end-expiratory lung volume increased, lung elastance decreased thus improving oxygenation. Setting PEEP according to a PEEPDEC trial after a RM further improved lung elastance and oxygenation. At PEEDEC level after a RM lung collapse and overdistension were minimized (Fig. 1). All patients maintained titrated PEEP levels up to 24 hours without complications.

**Conclusions:** In severely obese patients with ARDS, setting PEEP according to a PEEPINC trial or PEEPDEC trial following a RM identifies the same level of optimal PEEP. The improvement of lung mechanics, lung morphology and oxygenation at PEEPDEC after a RM suggests that lungs of obese ARDS patients are highly recruitable and benefit from a RM and high PEEP strategy.

**Table 1 (abstract P231).** Ventilator settings, respiratory mechanics, gas exchange

N=13	PEEP ICU	PEEP INCREMENTAL	PEEP DECREMENTAL
PEEP, cmH2O	12.7 ± 0.3	22.1 ± 0.8 *	21.7 ± 0.9 *
Driving Pressure, cmH2O	13.3 ± 1.1	10.9 ± 0.7 *	9.6 ± 0.5 *#
PLE, cmH2O	-5.5 ± 1.4	1.5 ± 1.0 *	1.4 ± 1.1 *
ElastanceL, cmH2O/L	27.8 ± 2.9	23.6 ± 1.6 *	19.6 ± 1.6 *#
ElastanceCW, cmH2O/L	8.1 ± 1.5	6.0 ± 0.9 *	5.3 ± 0.8 *
EELV, mL§	-	1040 ± 193 *	1090 ± 233 *
PaO2/FiO2	183.7 ± 30.1	242.6 ± 28.7 *	324.1 ± 23.2 *#

PEEP= Positive End-Expiratory Pressure; ICU= Intensive care Unit; PLE= Expiratory Transpulmonary Pressure; ElastanceL= Elastance of the Lung; ElastanceCW= Elastance of the Chest Wall; EELV= End-Expiratory Lung Volume; PaO2/FiO2= arterial partial pressure of oxygen to inspired fraction of oxygen ratio. § EELV is expressed as volume increment from the PEEP ICU level *p<0.05 compared to PEEP ICU (p < 0.05); # p<0.05 compared to PEEP INCREMENTAL

**Fig. 1 (abstract P231).**
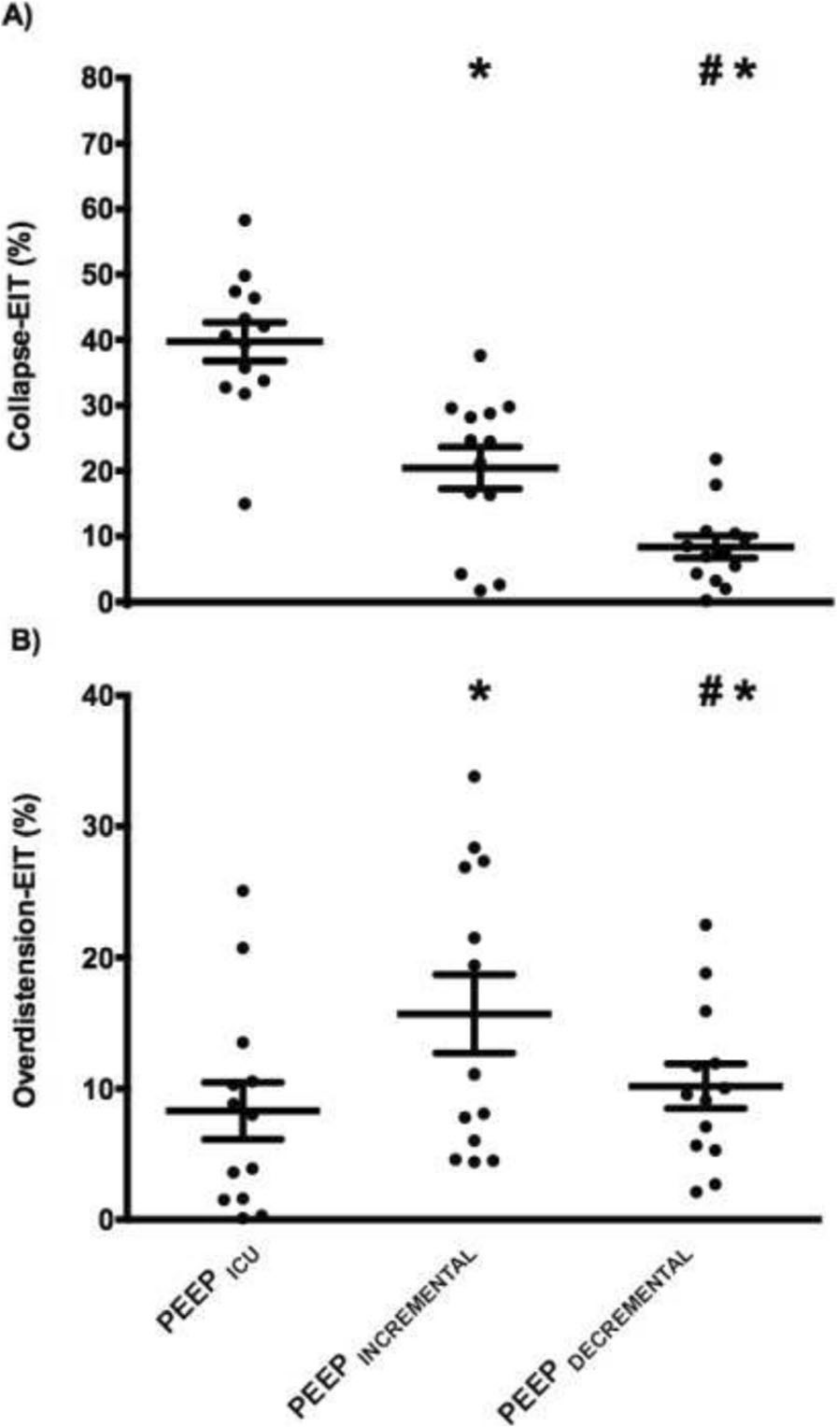
Lung Collapse and Lung Overdistension. PEEP= Positive End-Expiratory Pressure; ICU= Intensive care Unit; EIT= Electrical Impedance Tomography. *p<0.05 compared to PEEP ICU (p < 0.05); # p<0.05 compared to PEEP INCREMENTAL

## P232 hMSCs subjected to cyclic mechanical stretch change the damage of endothelial cell induced by LPS

### J Li, C Pan, H Qiu

#### Nanjing Zhongda Hospital, School of Medicine, Southeast University, Nanjing, China

**Introduction:** Mechanical stretch could change the paracrine function of mesenchymal stem cells (MSCs), and possibly change the damage of endothelial cell induced by LPS. Although previous studies have focused intensively on the effects of chemical signals that regulate MSC commitment, the effects of physical/mechanical cues of the microenvironment on MSC fate determination have long been neglected.

**Methods:** Human bone marrow MSCs proliferation was measured using CCK-8 assays after treatments with different time duration and stretch magnitude. To uncover the effect of stretch on MSC pro- and anti-inflammation, we measured the inflammation factors after been stimulated with the stretch. Additionally, we employed the morphological examination through Wright-Giemsa staining method to investigate the role of stretch on hMSCs. The VE-cadherin protein activities were assessed by using immunofluorescence.

**Results:** Cyclic mechanical stretch could significantly change the morphological and paracrine function of hMSCs, but do not alter the surface markers expression.Furthermore, stretched hMSCs deteriorate the endothelial permeability.

**Conclusions:** Our results showed that cyclic mechanical stretch significantly regulate human bone marrow MSC paracrine function. Therefore, more consideration would be took when MSCs engraftment in lung while breathing.This study provides insights into the mechanisms by which MSCs could be changed by the mechanical stretch.

## P233 Tidal volume reduction on ICU: a quality improvement project

### R Samanta, V Wong, R Talker, A Ecole, C Summers

#### Addenbrooke’s Hospital, Cambridge, UK

**Introduction:** Lung protective ventilation (LPV) strategies, principally focused around the use of tidal volumes <6 ml/kg predicted body weight (PBW) remains an enduring standard of care for ventilated patients. However, implementation of and compliance with LPV is highly variable. We used ‘nudge’-based interventions to assess if these can improve LPV.

**Methods:** Ventilation data analysis over 2 years (186000 hours in 685 patients) showed patients had been ventilated with a median tidal volume of 7.4 ml/kg PBW with a significant proportion receiving over 8 ml/kg PBW (Fig. 1), an effect more pronounced in female patients and those with higher BMI.

Interventions:

1. Creation of a software tool to easily identify and monitor patients receiving tidal volumes that were too high for their PBW
2. Attached laminated reference guides to each ventilator to calculate PBW
3. Presentation, opportunistic education and verbal prompts to relevant clinical care staff regarding importance of LPV and use of PBW rather than actual body weight
4. Incorporating checking of tidal volumes on a daily ward rounds from junior clinical members

**Results:** We collected hourly ventilation data of the patients over a 2-week period (2479 hours in 22 patients) following our interventions. There was, overall a statistically significant reduction tidal volume (p<0.001). There was improvement in the ventilation of male patients (p<0.001) but female patients endured higher tidal volumes. There was a mixed picture in different BMI grades.

**Conclusions:** Reducing tidal volumes in mechanically ventilated patients can be done through a mix of behavioural and educational interventions, as well as using technological shortcuts. This helps to reduce the effort on the part of clinical staff to adhere to best practices, and ultimately improve patient outcomes.

**Fig. 1 (abstract P233).**
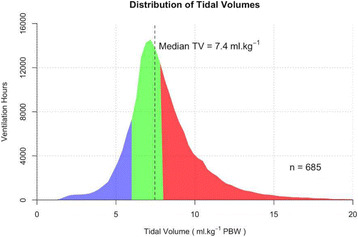
Distribution of tidal volumes recorded over a period of 2 years on the unit

## P234 Size matters: how can we improve intra-operative ventilation?

### L Raman, M Grover

#### Northwick Park Hospital, London, UK

**Introduction:** Lung protective ventilation (LPV) using a tidal volume (VT) of 6-8mL/Kg ideal body weight (IBW) is recommended in the intensive care unit and theatres to reduce the incidence of pulmonary complications. The aim of this audit was to assess the extent to which LPV is used in theatres in a busy district general hospital and to implement measures to promote adherence to the recommendations.

**Methods:** Anaesthetists completed questionnaires for all patients undergoing general anaesthesia at Northwick Park Hospital over 1 week. Demographics, actual body weight (ABW), height, American Society of Anesthesiologists (ASA) score, and procedural information were recorded. Ventilatory parameters included the ventilation mode, VT, and positive end expiratory pressure (PEEP). The body mass index (BMI), IBW and VT (expressed in mL/Kg of ABW and IBW) were calculated for each patient. A Mann Whitney U test was used to compare IBW and PBW and a Chi squared test was used to identify an association between VT and other variables.

**Results:** 129 patients were included; 65 males and 64 females. Mean age was 51. 73 patients were overweight (BMI >=25). 88% patients received PEEP. IBW was calculated in 106 patients and was significantly lower than ABW (61Kg [54-71] vs 72Kg [62-85] p<0.05) (Table 1). VT was higher when calculated from IBW than ABW (8.7mL/Kg [7.1-9.3] vs 7.5mL/Kg [5.8-7.9] p<0.05). 52 patients (49%) received LPV with VT of <8mL/Kg IBW in accordance with the recommendations (Fig. 1). Significantly more females (75%) received VT >=8ml/kg than males (29%) (p<0.01) (Fig. 2). VT was independent of age, ASA, BMI, ventilation mode, speciality, and patient position.

**Conclusions:** Over half of the patients received VT >=8ml/kg IBW. Females were more likely to be over ventilated. A likely contributing factor is the disparity between ABW and IBW in this cohort. We organised staff teaching and constructed IBW charts with the appropriate corresponding tidal volumes to be displayed in all theatres to promote the use of LPV.

**Table 1 (abstract P234).** Patient characteristics

Variable	Median	IQR
Actual body weight (Kg)	72	23
Ideal body weight (Kg)	61	17
Tidal volume (mL)	500	100
Tidal volume (mL/Kg ABW)	7.5	2.1
Tidal volume (mL/Kg IBW)	8.7	2.2
PEEP (cmH2O)	4	2
Duration of procedure (mins)	90	60

**Fig. 1 (abstract P234).**
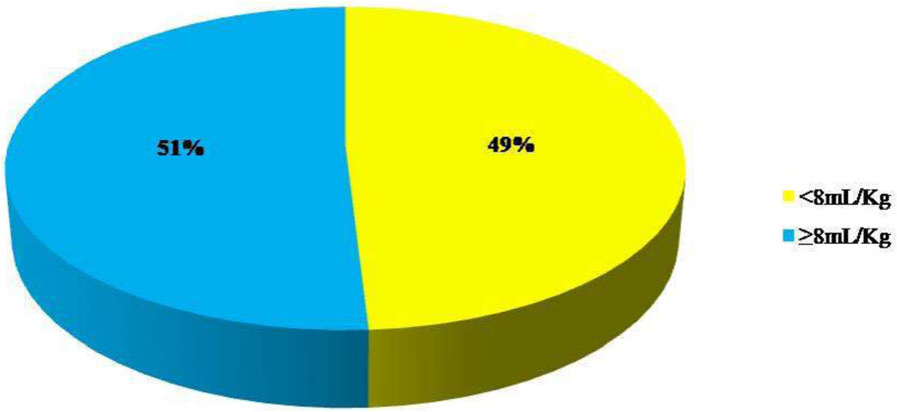
Lung protective vs Non lung protective ventilation

**Fig. 2 (abstract P234).**
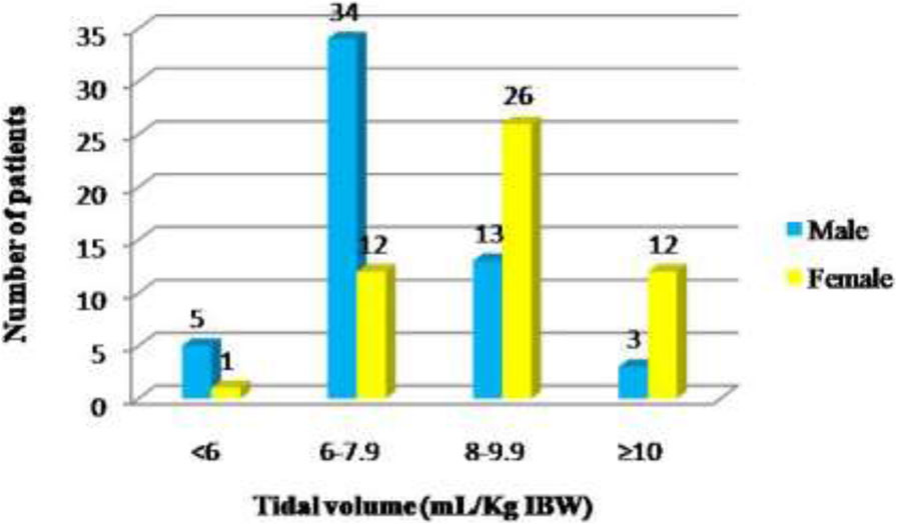
Sex specific tidal volumes

## P235 Protective mechanical ventilation to prevent acute respiratory distress syndrome

### A Kuzovlev^1^, A Shabanov^2^, T Smelaya^1^, A Goloubev^1^, V Moroz^1^

#### ^1^Federal Research and Clinical Center of Intensive Care Medicine and Rehabilitology, V.A. Negovsky research institute of general reanimatology, Moscow, Russia; ^2^N.V. Sklifosofsky Moscow City Research Institute of Emergency Care, Moscow, Russia

**Introduction:** Mechanical ventilation (MV) in protective mode seems the most reasonable way for prevention of acute respiratory distress syndrome (ARDS) in ventilator-associated pneumonia (VAP). The aim was to evaluate the efficiency of protective MV in preventing ARDS in VAP.

**Methods:** This retrospective study was done in 2013—2017. 102 patients with abdominal sepsis and VAP were enrolled in the stuidy. Patients were split in 2 groups: 1. protective MV: VAP patients were ventilated in protective mode (tidal volume (TV) 6-8 ml/kg); 2. standard MV: VAP patients were ventilated with TV 8—10 ml/kg. The ARDS incidence was assesed as primary endpoint. Secondary endpoints: duration of MV, length of ICU stay, 30-day mortality. Statistical analysis was done by Statistica 7.0 (±25—75 percentiles interquartile range (IQR); p <0.05).

**Results:** There were significant differences in ARDS incidence between groups: ARDS developed in 12.4% of protective MV groups vs. 68.3% of standard MV group (p=0.0001, Fisher’s exact test). VAP patients ventilated in a protective mode presented with lower duration of MV (12.2±4.2 days) and ICU stay(16.1±3.2 days) than patients with standard MV (17.2±5.2 and 20.1±5.5 days). There were significant differences in mortality rates between patient groups: 24.1% in protective MV and 47.2% in standard MV (p=0.0043, Fisher’s exact test).

**Conclusions:** Protective MV prevents the development of ARDS in VAP septic patients.

## P236 Ultra-protective ventilation with coaxial endotracheal tube and moderately high respiratory rates reduces driving pressure

### N Carvalho^1^, C Moraes^1^, A Beda^2^, M Nakamura^1^, M Volpe^3^, S Gomes^1^, O Stenqvist^4^, M Amato^1^

#### ^1^University of São Paulo, São Paulo, Brazil; ^2^Federal University of Minas Gerais, Belo Horizonte, Brazil; ^3^Federal University of São Paulo, Guarujá, Brazil; ^4^Sahlgrenska Universty Hospital, Goteborg, Sweden

**Introduction:** Reduction of tidal volumes (TV) below 6 mL/kg associated with low driving pressure (dP) might improve lung protection in patients with acute respiratory distress syndrome (ARDS). The current study tests the combination of coaxial double lumen endotracheal tube (to reduce instrumental dead-space) and moderately respiratory rate (RR) (<80 bpm) to maintain CO2 at clinically acceptable levels while using ultraprotective TV. The objective is to considerably reduce dP, which has been preconized as an index more strongly associated with survival than TV, per se, in ARDS patients. The ultraprotective ventilation setup proposed here kept the original tracheal tube and require nothing else than standard ventilator circuit and monitoring.

**Methods:** 8 juvenile pigs were anesthetized, intubated and mechanically ventilated. Severe lung injury (P/F<100) was induced using a double-hit model: repeated surfactant wash-out followed by injurious mechanical ventilation using low positive end-expiratory pressure and high dP (~40cmH2O) for 3 hours. Then VTs of 6, 4, and 3 ml/kg were used in random sequence for 30 min each, both using a standard and coaxial endotracheal tube. At each VT level, RR was adjusted to achieve PaCO2=60 mmHg but not exceeding 80 bpm. Lung functional parameters and blood gas analysis were measured at each VT level. Statistical analysis was performed using mixed linear model.

**Results:** Coaxial endotracheal tube, but not the conventional tube, allowed decreasing VT to 4 and 3 ml/kg, while keeping PaCO2 at approximately 60 mmHg and RR<80 bpm, reducing dP of 4.0 cmH2O and 6.0 cmH2O, respectively, compared to the conventional VT of 6 ml/kg (Fig. 1).

**Conclusions:** In this ARDS model, coaxial tube ventilation associated with moderately high RR allowed ultraprotective ventilation (VT=3 ml/kg) and reduced dP levels, maintaining PaCO2 at acceptable levels. This strategy might have a significant impact on mortality of severe ARDS patients.

**Fig. 1 (abstract P236).**
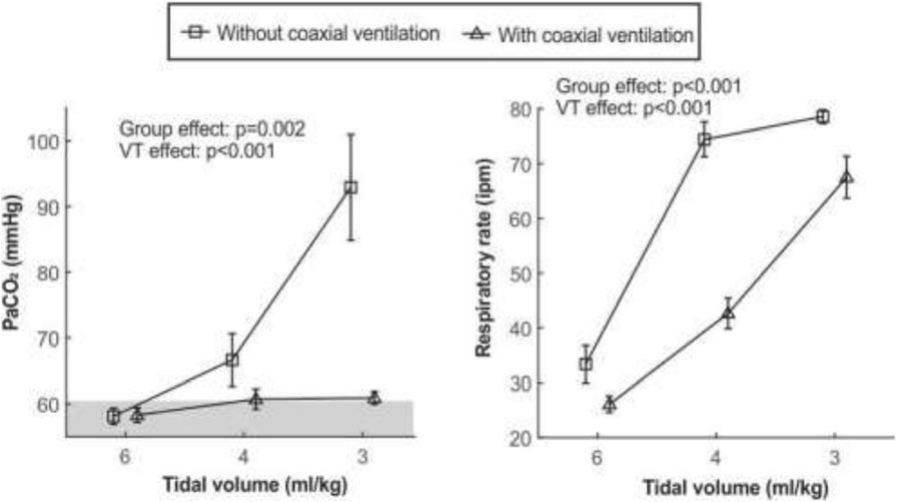
Arterial pressure of CO2 and respiratory rate at each studied tidal volume (6, 4, and 3 ml/kg) and group (with and without coaxial ventilation)

## P237 The use of acute cor pulmonale risk score for the diagnosis of acute cor pulmonale in acute respiratory distress syndrome

### H Elsayed, T Zaytoun, O Hassan, A Sarhan

#### Faculty of medicine - Alexandria university, Alexandria, Egypt

**Introduction:** Acute cor pulmonale (ACP) is a common sequel in acute respiratory distress syndrome (ARDS) patients and represents the most severe presentation of right ventricular dysfunction, secondary to pulmonary vascular dysfunction. Although most previous studies adopt trans-oesophageal echocardiography in the diagnosis of ACP in ARDS, transthoracic echocardiography (TTE) appears as an attractive alternative being noninvasive, more available with continuously improving expertise in its use by the intensivists. Our study aimed to test the accuracy of ACP risk score by using TTE.

**Methods:** Our study was carried out on 45 mechanically ventilated ARDS patients. The patients were mechanically ventilated using lung protective approach. TTE was performed within the first 72 hours of ARDS diagnosis. ACP was diagnosed when the ratio of right ventricular end-diastolic area/left ventricular end-diastolic area >0.6 in apical 4 chamber view (indicating right ventricular diastolic overload) associated with interventricular septum dyskinesia at end-systole (indicating right ventricular systolic overload). ACP risk score parameters were checked and scored (1 point for each parameter) (pneumonia as a cause of ARDS, driving pressure >= 18 cmH2O, PaCO2 >= 48 mmHg, PaO2/FiO2 ratio < 150 mmHg).

**Results:** ACP risk score showed high sensitivity (100%), average specificity (51.43%) and good overall accuracy (62.2%) when 2 was used as cut off value. Hypercapnia, hypoxia, pneumonia, high plateau pressure and high positive end-expiratory pressure were associated with increased incidence of ACP in ARDS patients and considered as independent factors of mortality in ARDS patients.

**Conclusions:** ACP risk score is a highly sensitive score in predicting and diagnosis of ACP in ARDS patients. The disease process, as well as the ventilatory maneuvers, may share in the development of ACP in ARDS.

## P238 Expiratory ventilation assistance improves arterial oxygenation in ARDS – a randomized controlled study in pigs

### J Schmidt, C Wenzel, S Spassov, S Wirth, S Schumann

#### Medical Center - University of Freiburg, Freiburg, Germany

**Introduction:** Mechanical ventilation aggravates ARDS. Expiratory ventilation assistance (EVA) showed an improved oxygenation in lung healthy pigs with similar tracheal pressure (Ptrach) amplitude and tidal volume (VT). We hypothesized that EVA improves gas exchange and attenuates ventilator induced lung injury in a porcine model of ARDS.

**Methods:** 19 pigs with an oleic acid induced moderate ARDS (initial Horovitz index (HI) 100-150 mmHg) were randomly allocated to volume controlled ventilation or EVA ventilation with identical ventilation parameters (FiO_2_ 0.8, VT 7 ml/kg body weight, PEEP 9 mbar, respiratory rate set to maintain arterial blood pH >7.2). PaO_2_ and Ptrach were measured every 30 min. After 3h lung tissue was excised, stained and alveolar wall thickness measured. Statistics were performed with linear mixed model analyses and unpaired t-test.

**Results:** 5 pigs were excluded due to HI < 100 mmHg (n=2), malignant arrhythmia (n=1) and software error (n=2). EVA elevated PaO_2_ (107±11.3 vs. 164±21 mmHg, p=0.04) and mean Ptrach (16.7±1.6 vs. 21.5±1.1 mbar, p<0.0001). Alveolar walls were thinner in the EVA group (7.8±0.2 vs. 5.5±0.1 μm, p<0.0001).

**Conclusions:** EVA ventilation improves gas exchange due to elevated mean Ptrach in experimental ARDS. Reduced alveolar wall thickness indicates potential lung protective effects.

Funding: European Union, Horizon 2020 research and innovation programme, Grant #691519.

## P239 Application of transpulmonary pressure measurement in severe ARDS

### S Jog, J Mulchandani, A Garg, P Kalyani, P Rajhans, P Akole, B Pawar, B Bhurke, S Chavan, P Dalvi, S Sable, A Kulkarni, V Giri, N Kunjir, N Mahale, G Ranade, R Desai

#### Deenanath Mangeshkar Hospital, Pune, India

**Introduction:** Transpulmonary pressure(TPP) measurement is a promising tool used for ventilatory adjustments in ARDS patients. Its usefulness and application in the management of severe ARDS remains unclear. Aim of this study is – To measure TPP in severe ARDS patients and describe the possible interventions in ventilatory settings.

**Methods:** Prospective observational study. TPP was measured by an esophageal balloon catheter(Nutrivent, Sidam, Italy) and a suitable ventilator(Hamilton G5, Switzerland). Inclusion criteria – Adult patients with severe ARDS (PO2/FiO2 ratio < 100) having PEEP >= 14 cm, Airway plateau pressure > 30 cm and under deep sedation with neuromuscular blockade. Exclusion Criteria – 1) Patients receiving ECMO 2) Patients in whom position of esophageal balloon and measurement of TPP could not be confirmed by cardiac oscillation and abdominal pressure technique. Ventilatory parameters and blood gas parameters were documented before measuring TPP and after measuring TPP. Ventilatory setting adjustments done were – i) increasing PEEP in hypoxic patients(SpO2 <88 %) till oxygen saturation remained > 90% provided plateau TPP remained < 23 cm and end expiratory TPP remained > 0 cm ii) Increasing tidal volume(>6 ml/kg) in hypercapnoeic acidotic patients (pH< 7.20 and PCO2> 80mm Hg) provided plateau TPP remained < 23cm

**Results:** Data of 10 patients analysed. 6 out of 10 patients were obese. Average plateau TPP was 21.1 ±2.91 cm. Average end expiratory TPP was 1.2 ± 1.1cm. None of these 10 patients developed pneumothorax even at average PEEP of 20.7± 2.53cm, highest PEEP being 24cm (Table 1). 5 out of 10 patients died due to sepsis and MODS.

**Conclusions:** TPP measurement is useful in setting appropriate PEEP and Tidal volume in severe ARDS patients in whom airway plateau pressure is more than 30 cm.

**References**

Talmor D et al NEJM 2008

Grasso S et al ICM 2012

**Table 1 (abstract P239).** Ventilatory settings and blood gas variables before and after TPP measurement

	Variable	Pre TPP	Post TPP
1	Tidal Volume ml	386 ± 32.61	412±71.24
2	PEEP cm of H2O	15.2±1.24	20.7±2.53
3	Airway Pplat cm of H2O	34.2±3.36	41.2± 2.54
4	pH	7.24±0.06	7.23±0.06
5	PO2 mm Hg	69.3±14.16	87.1±20.24
6	PO2/FiO2 ratio	80.9±17.54	105.9±23.81
7	PCO2 mm Hg	61.8±14.35	60.8±12.4

## P240 Impact of a stepwise increase in PEEP (PEEP-trial) on haemodynamics, static compliance, functional residual capacity and tidal volume: a study in patients with picco-monitoring

### W Huber, J Wettstein, S Rasch, T Lahmer, A Herner, M Heilmaier, G Batres-Baires, R Schmid, U Mayr

#### Klinikum rechts der Isar, Munich, Germany

**Introduction:** Advanced respirator technologies provide automated stepwise PEEP-increases in parallel with measurement of functional residual capacity (FRC) and static pulmonary compliance (C_stat). While this PEEP-Trial (PT) might facilitate optimal PEEP-setting, it also carries the risk of haemodynamic instability due to the reduced venous return. We hypothesized that a standard PT with stepwise increases in PEEP of a total of 8cm H2O might result in substantial haemodynamic changes. Since heart lung interactions have been suggested to diagnose volume responsiveness, the PT could be used as a combined approach to optimize ventilation and haemodynamics.

**Methods:** In 28 mechanically ventilated patients (Carescape R860; GE; pressure-controlled ventilation; PF-ratio of 249+-83) and PiCCO-monitoring, an automated five step PEEP-trial was performed. PEEP was increased from values 4cmH20 below to 4cmH2O above the preset PEEP-level. PiCCO-data, ventilator settings, FRC and C_stat were measured at baseline and after each increase.

**Results:** Mean values for none of the haemodynamic parameters including heart rate, MAP, CVP, global end-diastolic volume index GEDVI, cardiac index, stroke volume variation SVV, extravascular lung water index EVLWI and cardiac power index CPI were significantly different at the end compared to the start of the PT. PEEP (12.2+-1.3 vs. 4.4+-1.4cmH2O; p<0.001) and FRC (1884+-850 vs. 1546+-612ml; p<0.001) were significantly higher after the PT, while all other respiratory data including tidal volume and C_stat were not different after the PT. Individual maximum values during the PT were significantly higher for tidal volume (516+-126 vs. 467+-146mL; p<0.001) and C_stat (50+-24 vs. 40+-23mL/cmH2O; p<0.001) compared to baseline.

**Conclusions:** 1.) The PT did not impair haemodynamics. While the PT is haemodynamically safe, its use to detect volume deficiency seems to be low. 2.) Adjustment of PEEP according to the data of the PT substantially improves C_stat and tidal volume.

## P242 Restoration of homogenous ventilation does not impair the right heart function

### M Teggia Droghi^1^, R De Santis Santiago^2^, J Fumagalli^3^, S Kaneki^2^, G Galli^2^, S Palma^2^, G Larson^2^, M Bottiroli^4^, R Pinciroli^4^, F Marrazzo^2^, A Bagchi^2^, K Shelton^2^, A Sonny^2^, M Amato^5^, R Kacmarek^2^, L Berra^2^

#### ^1^University of Milano-Bicocca, Monza, Italy; ^2^Massachusetts General Hospital, Boston, MA, USA; ^3^Fondazione IRCCS Cà Granda Ospedale Maggiore Policlinico, Milan, Italy; ^4^ASST Grande Ospedale Metropolitano Niguarda, Milan, Italy; ^5^University of São Paulo, Heart Institute, São Paulo, Brazil

**Introduction:** High levels of PEEP during mechanical ventilation can impair right heart function by increasing afterload [1]. Due to increased pleural pressure, patients with morbid obesity and Acute Respiratory Distress Syndrome (ARDS) need Recruitment Maneuver (RM) and a higher level of PEEP to restore and maintain homogeneous ventilation, thereby avoiding atelectasis [2]. We hypothesize that optimizing PEEP will not impair RV function, even if high levels of PEEP are needed in morbidly obese patients with ARDS.

**Methods:** A clinical longitudinal study was performed in mechanically ventilated morbidly obese patients with ARDS admitted to the Surgical or Medical ICU. Echocardiographic indices of right heart function: TAPSE (Tricuspid annular plane systolic excursion) and S’ (Velocity of the tricuspid annulus during systole) were measured in the study cohort at 3 different levels of PEEP

1. Baseline: PEEP based on bedside saturation of oxygen (SatO2>92%).
2. Incremental: PEEP at +2cmH2O of transpulmonary pressure.
3. Decremental: PEEP titrated to best compliance of the respiratory system after a RM.

During each phase of the study ventilatory parameters and oxygenation were also recorded.

**Results:** 17 patients were enrolled (age: 50.4±14.2 years; BMI: 55.8±13.1 kg/m2). Table 1 shows oxygenation and respiratory mechanics. Figure 1: Echocardiographically measured right heart function.

**Conclusions:** In morbidly obese mechanically ventilated patients with ARDS an increase in PEEP by 9 cmH2O (from 12.5±1.5 cmH2O to 21.2±3.3 cmH2O) did not impair right heart function, but improved respiratory mechanics and oxygenation.

**References**

[1] Repessé et al. Chest 147.1: 259-265, 2015.

[2] Fumagalli et al. Crit Care Med 45.8: 1374-1381, 2017.

**Table 1 (abstract P242).** Ventilatory setting, oxygenation, mechanics and echocardiography measurements

	Baseline (n=17)	Incremental (n=17)	Decremental (n=17)
PaO2/FiO2 ratio	147 ± 86.7	194.1 ± 107.3	286.7 ± 95.9*^
PEEP (cmH2O)	12.5 ± 1.5	21.8 ± 3.3*	21.2 ± 3.3*
Driving Pressure (cmH2O)	13.5 ± 3.8	12.2 ± 3.0	10.1 ± 2.6*^
TAPSE (cm)	2.38 ± 0.42	2.27 ± 0.39	2.22 ± 0.43
S’ (cm/s)	13.85 ± 2.53	12.96 ± 2.35	12.78 ± 3.23

PaO2/FiO2= arterial partial pressure of oxygen to inspired fraction of oxygen ratio. PEEP=Positive End Expiratory Pressure, TAPSE (Tricuspid annular plane systolic excursion) and S’ (Velocity of the tricuspid annular systolic motion). All the results are shown as Average ± Standard Deviation. * significant compared to Baseline p<0.05; ^ significant compared to Incremental p<0.05.

**Fig. 1 (abstract P242).**
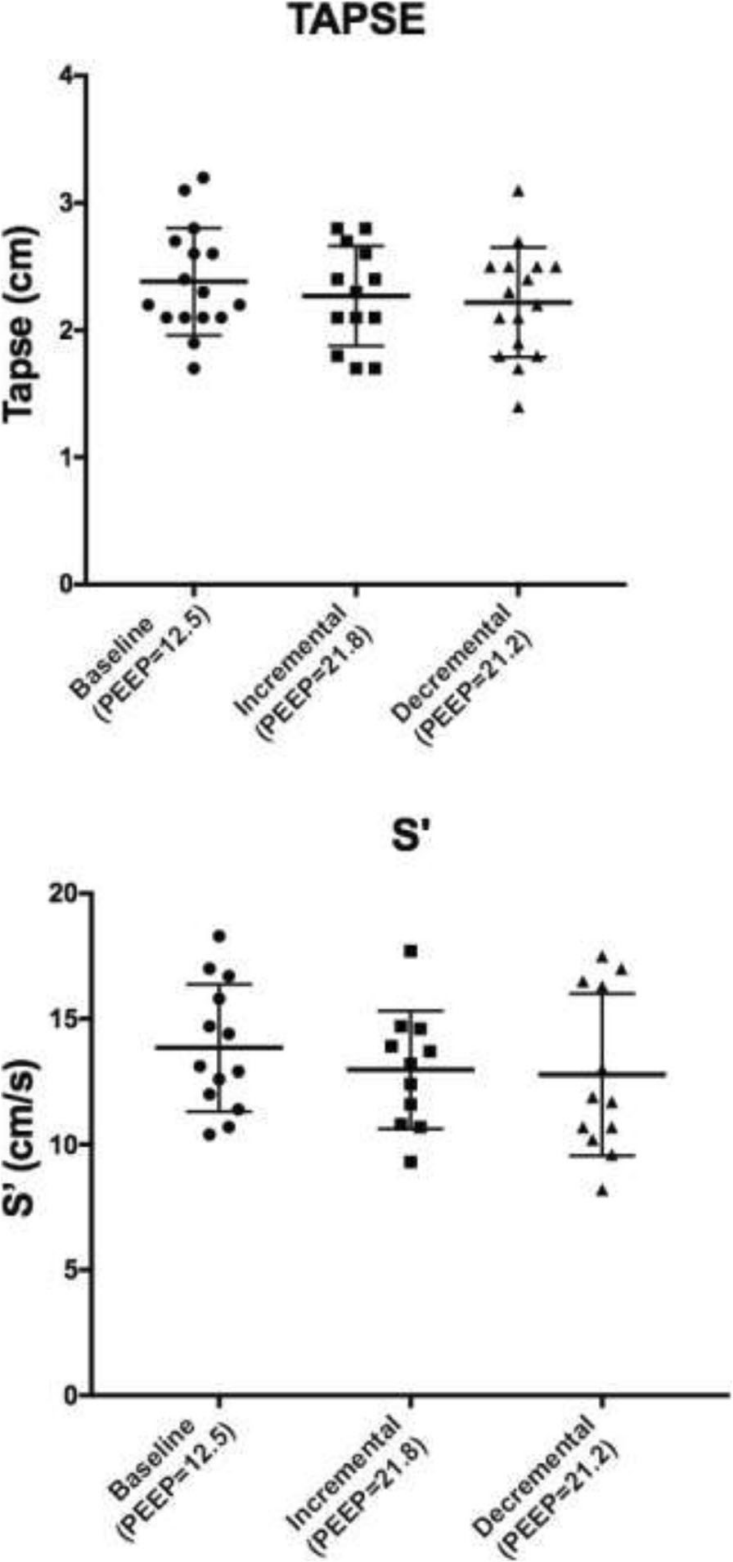
Mean and SD of TAPSE (Tricuspid annular plane systolic excursion) and S’ (Velocity of the tricuspid annular systolic motion) at the three different phases of the study: Baseline, Incremental, Decremental.

## P243 Heparin-binding protein in ventilator induced lung injury

### J Tyden^1^, N Larsson^1^, H Herwald^2^, M Hultin^1^, J Wallden^1^, J Johansson^1^

#### ^1^Umea university, Umea, Sweden; ^2^Lund university, Lund, Sweden

**Introduction:** Mechanical ventilation can, while being lifesaving, also cause injury to the lungs. The lung injury is caused by high pressures and mechanical forces but also by inflammatory processes which are not fully understood [1]. Heparin binding protein (HBP) released by activated granulocytes has been indicated as a possible mediator of increased vascular permeability in the lung injury associated with trauma and sepsis [2, 3]. We wanted to investigate if HBP levels were increased in bronco alveolar lavage (BAL) fluid or plasma in a pig model of ventilator induced lung injury.

**Methods:** Anaesthetized pigs were surfactant depleted by saline lavage and randomized to receive ventilation with either tidal volumes of 8 ml/kg with a PEEP of 8 cm H2O (controls, n=6) or 20 ml/kg with a PEEP of 0 cm H2O (ventilator induced lung injury (VILI) group, n=6). Plasma and BAL samples of HBP were taken at 0,1,2,4 and 6 hours (Fig. 1).

**Results:** Characteristics of pigs by study group are shown in Table 1. Plasma levels of HBP did not differ significantly between pigs in the control and VILI group at any time of sampling. HBP levels in BAL fluid were significantly higher in the VILI group after 1 (p=0.04), 2 (p=0.03), 4 (p<0.01) and 6 (p=0.02) hours of ventilation (Fig. 2).

**Conclusions:** In a model of ventilator induced lung injury in pigs, levels of Heparin binding protein in BAL fluid increased significantly over time compared to controls. Plasma levels however did not differ significantly between groups.

**References**

1. Slutsky AS et al. N Engl J Med 369(22):2126-2136, 2013

2. Johansson J et al. Acta Anaesthesiol Scand 57(5):580-586, 2013

3. Bentzer P et al. Intensive Care Med Exp 4(1):33, 2016

**Table 1 (abstract P243).** Characteristics of pigs by study groups. Data presented as median (interquartile range)

	Control n=6	VILI n=6	p value
Weight (kg)	35.5 (28 - 50)	35.5 (29 - 47)	0.818
Premature death (n)	0	2	
Tidal volume (ml)	280 (228 - 400)	710 (575 - 940)	0.002
Respiratory rate (breaths/min)	30 (26 - 38)	20 (20 - 20)	0.002
Average peak inspiratory pressure (cmH2O)	22 (20 - 23)	43(39 - 48)	0.002
FiO2	0.35 (0.29 – 0.40)	1.0 (1.0 – 1.0)	0.002
Worst P/F ratio (kPa)	46 (34 - 52)	14 (8 - 39)	0.041
Last P/F ratio	53 (43 - 64)	25 (8 - 43)	0.065
Last PCO2 (kPa)	4.6 (3.8 – 5.0)	5.9 (4.7 – 7.0)	0.093
Last pH	7.52 (7.47 – 7.55)	7.41 (7.31 – 7.48)	0.026
Total crystalloid (ml)	2000 (975 - 3000)	2500 (1800 - 3200)	0.394
Total kolloid (ml)	0 (0 - 500)	375 (0 - 500)	0.485
Average fluid admin. (ml/kg/h)	7.7 (4.4 – 8.2)	10.0 (8.5 – 12.0)	0.026
Average CVP (mmHg)	8 (7 - 8)	7 (6 - 9)	0.394
Average MAP (mmHg)	70 (67 - 75)	75 (53 - 102)	0.24
Average heart rate (bpm)	87 (82 - 95)	103 (94 - 111)	0.065
Average temp (Degr.C)	39.4 (38.3 – 39.7)	38.5 (38.0 – 39.3)	0.394
Recovered BAL volume (percent)	78 (62.5 – 84.0)	71 (60.0 – 79.0)7	0.168

**Fig. 1 (abstract P243).**
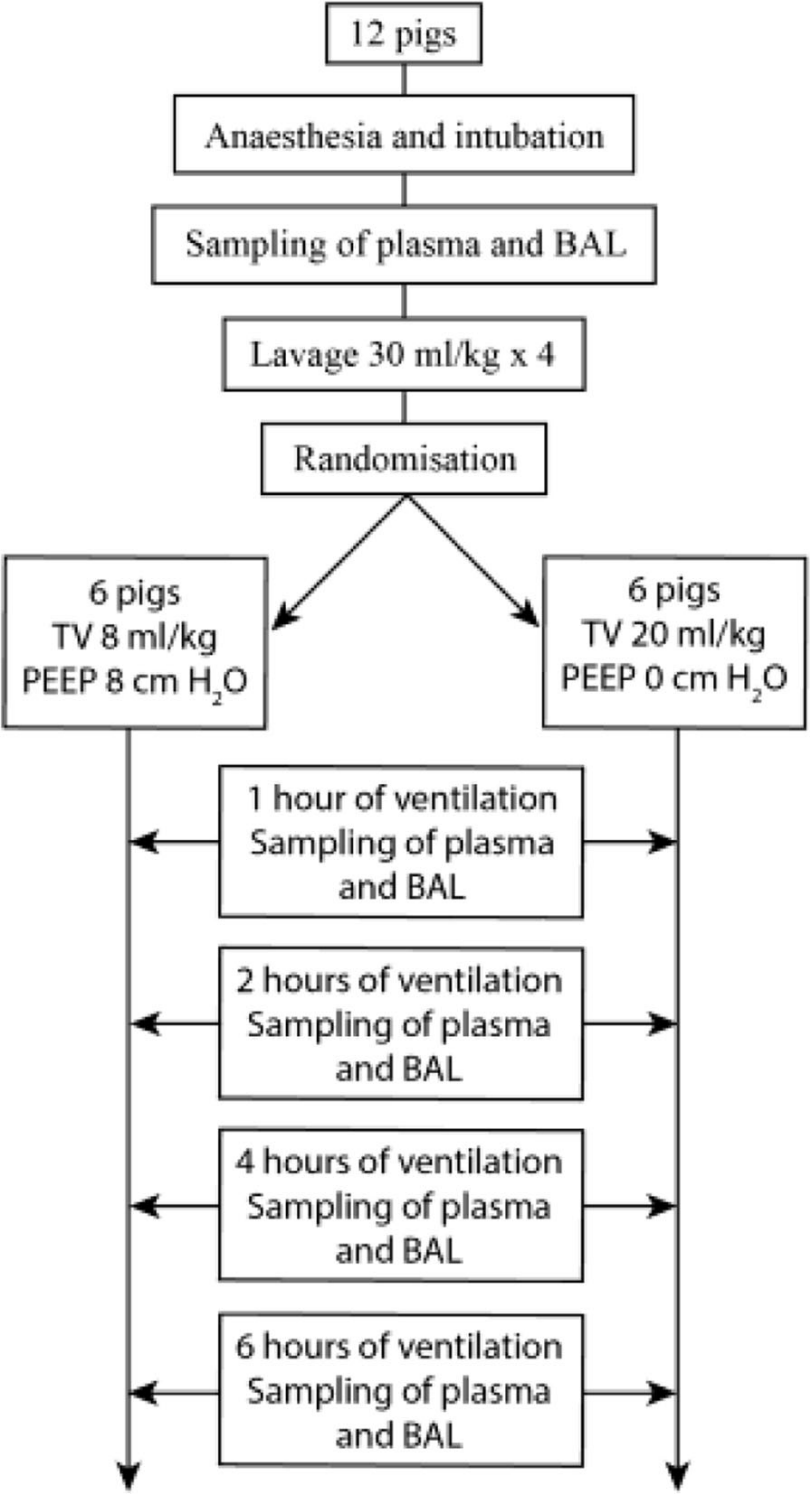
Schematic overview of protocol

**Fig. 2 (abstract P243).**
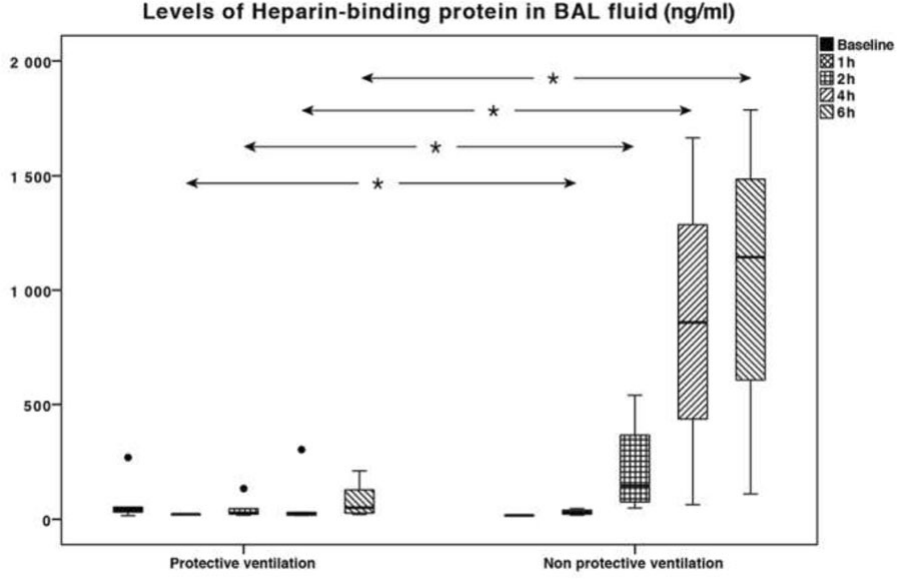
Levels of Heparin binding protein (HBP)(ng/ml) in bronco alveolar lavage (BAL) fluid. HBP levels were significantly higher in the group receiving harmful ventilation at 2 (p=0.03), 4 (p<0.01) and 6 (p=0.02) hours of ventilation.

## P244 Efficacy and safety of corticosteroids for acute respiratory distress syndrome: an updated meta-analysis

### N Owattanapanich^1^, N Phaetthayanan ^2^

#### ^1^Faculty of Medicine, Siriraj hospital, Bangkok, Thailand; ^2^Nakhonpathom hospital, Nakhonpathom, Thailand

**Introduction:** Background: The pharmacological treatment options for acute respiratory distress syndrome are limited. The use of corticosteroid remains controversial. In this study, we aimed to analyze the effect of corticosteroid regarding its efficacy and safety.

Objectives: The primary outcome of this study was to assess the mortality benefit in corticosteroid group. The secondary outcomes were improvement of lung function, ventilator day and nosocomial infection rate.

**Methods:** PubMed, Medline-Ovid, Scopus and EMBASE data searching were conducted. Additional searching was also performed by reviewing of relevant primary literatures and review articles. Randomized controlled trials and cohort studies which reported the mortality associated with corticosteroid treatment were selected. A random effect model was used to estimate mortality and other outcomes.

**Results:** Of the 1,254 initially reviewed studies, 20 were selected for this meta-analysis. There was no statistically significant decrease mortality in corticosteroid group (95% CI: 0.53, 1.11) (Fig. 1). Heterogeneity was observed with I2 69%, df= 17 (P<0.00001). Corticosteroid group showed improvement of lung function regarding PaO2/FiO2 ratio (95% CI: 19.60, 73.74). However, there was no significant difference in ventilator day (95% CI: -4.56, 0.27) In terms of safety, the corticosteroid group had found no evidence of significant increase in nosocomial infection (95%CI: 1.00, 1.87) (Fig. 2).

**Conclusions:** This meta-analysis concluded that corticosteroid treatment in ARDS provided no benefit in decreasing mortality. In addition, this treatment was not associated with increasing risk of nosocomial infection.

**References**

Meduri GU et al. Chest 131(4):954-63, 2007

**Fig. 1 (abstract P244).**
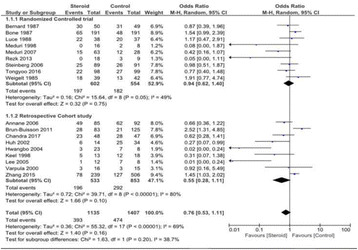
Forest plot showed effect of steroid on mortality

**Fig. 2 (abstract P244).**
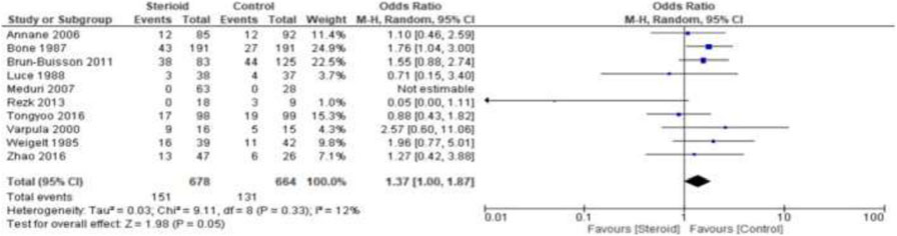
Forest plot showed incidence of nosocomial infection

## P245 The effect of surfactant administration on outcomes of adult acute respiratory distress syndrome patients: a systematic review and meta-analysis of randomized controlled trials

### S Meng, W Chang, Z Lu, J Xie, H Qiu, Y Yang, F Guo

#### Department of Critical Care Medicine, Zhongda Hospital, School of Medicine, Southeast University, Nanjing, China

**Introduction:** Acute respiratory distress syndrome(ARDS) patients usually lack of surfactant. Surfactant administration may be a useful therapy in adult ARDS patients. The purpose of this study was to perform a systematic review and meta-analysis of the effect of surfactant administration on outcomes of adult acute respiratory distress syndrome patients.

**Methods:** PubMed, EMBASE, Medline, Cochrane database, Elsevier, Web of Science and ClinicalTrials.gov were searched until December 2016.Randomized controlled trials comparing surfactant administration with general therapy in adults with acute respiratory distress syndrome were included. The primary outcome was mortality (7-10 days, 28-30 days and 90-180 days). Secondary outcome included a change in oxygenation (PaO2/FiO2 ratio). Demographic variables, surfactant administration, and outcomes were retrieved. Internal validity was assessed using the risk of bias tool. Random errors were evaluated with trial sequential analysis. Quality levels were assessed by Grading of Recommendations Assessment, Development, and Evaluation methodology.

**Results:** Eleven RCTs and 3038 patients were identified. Surfactant administration could not improve mortality of adult patients [RR (95%CI)=1.02(0.93-1.12), p=0.65]. Subgroup analysis revealed no difference of 7-10-day mortality [RR(95%CI)=0.86(0.52-1.43), p=0.56], 28-30-day mortality[RR(95%CI)=1.00(0.89-1.12), p=0.98] and 90-180-day mortality [RR(95%CI)=1.11(0.94-1.32), p=0.22] between surfactant group and control group (Fig. 1). The change in the PaO2/FiO2 ratio was significant [RR(95%CI)=0.29(0.12-0.46), p=0.0008] (Fig. 2). Finally, trial sequential analysis and GRADE indicated lack of firm evidence for a beneficial effect.

**Conclusions:** Surfactant administration may improve oxygenation but has not been shown to improve mortality for adult ARDS patients. Large rigorous randomized trials are needed to explore the effect of surfactant to adult ARDS patients.

**Fig. 1 (abstract P245).**
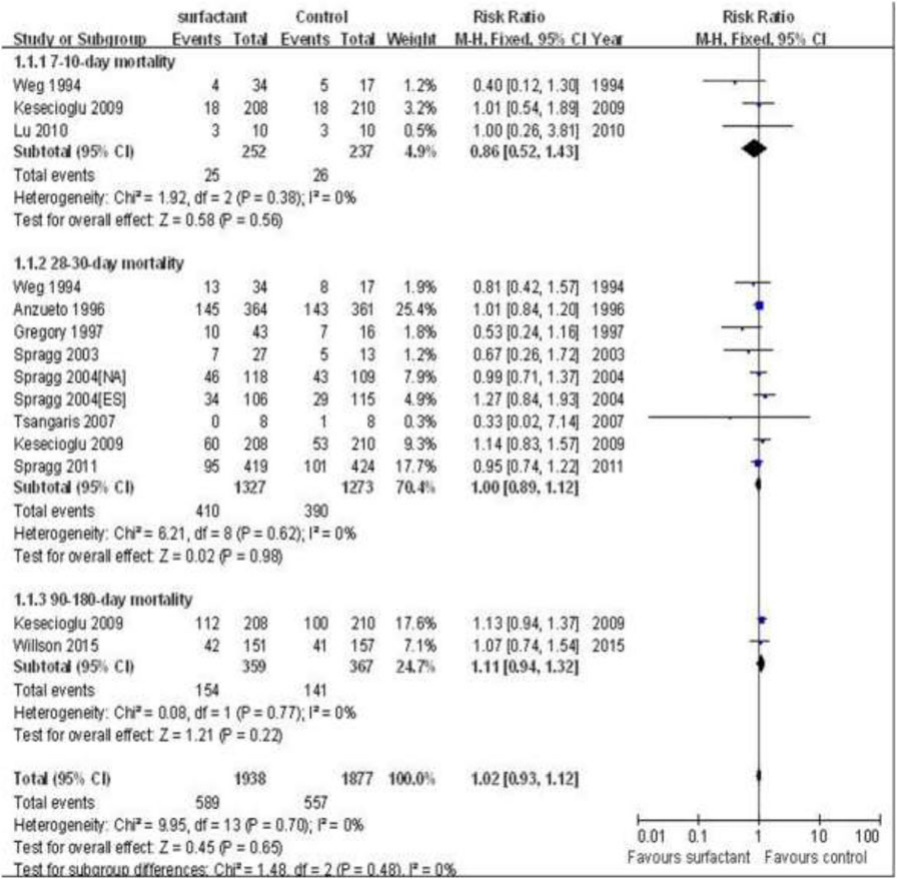
Forest plots of subgroup analyses on the effect of surfactant based on different days mortality. CI Confidence interval, M-H Mantel-Haenszel.

**Fig. 2 (abstract P245).**
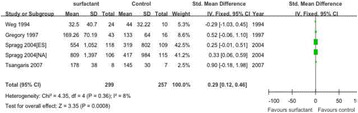
Forest plots of the effect of surfactant based on PaO2/FiO2. CI Confidence interval, M-H Mantel-Haenszel.

## P246 Moderate to severe acute respiratory distress syndrome in a population of primarily non-sedated patients, an observational cohort study

### L Bentsen, T Strøm, P Toft

#### Odense University Hospital, Odense C, Denmark

**Introduction:** Acute Respiratory Distress Syndrome (ARDS) is a common condition in the intensive care unit (ICU). It is characterized by hypoxemia, bilateral opacities at thoracic radiography and the need for mechanical ventilation. The current standard treatment for patients with ARDS is the use of lung protective ventilation and deep sedation. The use of non-sedation for ICU patients has shown that it can reduce ICU length of stay and time in mechanical ventilation, but haven’t been investigated for ARDS.

**Methods:** We conducted a retrospective study of all patients with ARDS admitted to the ICU of Odense University Hospital in the period from 1st January 2012 to 31st December 2016. All patients with moderate to severe ARDS as defined by The Berlin Definition of ARDS were included. We searched the ICU database for patients of at least 18 years of age, mechanical ventilated with at least 72 hours of ICU stay. At least two PaO2/FiO2 ratios of <200mmHg and intubation at some point during admission. The patients in the ICU are not sedated but treated with morphine bolus injections of 2.5 or 5mg until tolerance of the tracheal tube or tracheostomy. If non-sedation strategy is insufficient for patient treatment, sedation is targeted to a Richmond Agitation-Sedation Scale (RASS) of -2 to -3 with a daily wake up call.

**Results:** We evaluated 1446 patients in the ICU database and of these 306 had moderate or severe ARDS. The median age was 67 (57-74) and 201 were male (65.7%). 30-day mortality was 39.9% and 25% of the patients was mobilized out of bed within 14 days after admittance to the ICU. Median APACHE II were 27 (23-31) and median SAPS II were 50 (41-62). The median RASS were 0 (-3-0) at day 1 and 7 of ICU admittance. Most patients were ventilated using pressure support.

**Conclusions:** Non-sedation was used in patients with ARDS and the 30-day mortality was 39.9%. Compared to other studies our patients were more ill with similar mortality.

## P247 A new quantitative ct parameter as a one stop-shop to describe status and outcome of patients with acute respiratory distress syndrome

### P Leiser^1^, H Haubenreisser^1^, S Schönberg^1^, C Weiß^2^, M Hagmann^2^, J Schoettler^3^, FS Centner^3^, T Kirschning^3^, M Thiel^3^, J Krebs^3^

#### ^1^Institute of Clinical Radiology and Nuclear Medicine, Mannheim, Germany; ^2^Department for Medical Statistics and Biomathematics, Mannheim, Germany; ^3^Department of Anaesthesiology and Intensive Care Medicine, Mannheim, Germany

**Introduction:** The aim of this study was to establish quantitative CT (qCT) parameters for pathophysiological understanding and clinical use in patients with ARDS. The most promising parameter is introduced.

**Methods:** 28 intubated patients with ARDS obtained a conventional and a dual energy CT scan under an end-expiratory hold manoeuvre. Following manual segmentation, 138 volume-, perfusion- and lung weight-related qCT parameters were correlated with 71 anaesthesiological parameters such as applied ventilation pressures (PEEP, Pdrive), the patients’ oxygen supply (SO_2_, PaO_2_/FiO_2_) and established status and prognosis scores (SOFA, SAPS II). Multiple regression analysis was then performed to enable the prediction of these scores by a single CT scan.

**Results:** Of all examined qCT parameters, excess lung weight (ELW) displayed the most significant results [1]. ELW correlates positively with the amount of extravascular lung water (r=0.72), atelectatic lung volume (r=0.92), applied PEEP (r=0.37) and negatively with the lung’s mean CM-Enhancement (r=-0.65; all p<0.05). More significantly than any other anaesthesiological parameter it correlates with the patient’s SOFA- (p<0.0001, r=0.69) and SAPS II-Score (p=0.0005, r=0.62). A combination of ELW, mean CM and Pdrive can predict SOFA up to r^2^=87.95%.

**Conclusions:** ELW constitutes the best parameter to assess pathophysiology and status of patients with ARDS. It can help the clinician to predict outcome and mortality with higher accuracy than current standard Horowitz index (PaO_2_/FiO_2_) and should therefore be considered a first range diagnostic tool during the first hours of ICU treatment.

**Reference**

1. Cressoni M et al. Critical Care 17:R93, 2013

## P248 Bronchoalveolar lavage as a diagnostic and prognostic tool in acute respiratory failure

### Y Iwasaki, Y Kida, M Kyo, K Hosokawa, S Ohshimo, N Shime

#### Hiroshima University, Hiroshima, Japan

**Introduction:** Bronchoalveolar lavage (BAL) is a useful tool for diagnosing diffuse pulmonary diseases. However, the utility of BAL for acute respiratory failure in the intensive care unit (ICU) setting have not been well investigated.

**Methods:** We retrospectively collected consecutive 91 patients who were diagnosed as having acute respiratory failure with diffuse pulmonary involvements in an emergency and critical care unit of a University-affiliated Hospital from 2010 to 2017. We investigated the correlations between BAL analysis, clinical diagnosis and prognosis in these patients.

**Results:** There were 63 males and 28 females (median age, 68 years [IQR 60-75]; PaO_2_/FIO_2_(P/F) ratio, 124 [IQR 75-176]. All patients were diagnosed as having acute respiratory distress syndrome (ARDS) based on the radiological and laboratory findings. The major findings of BAL included alveolar hemorrhage (n=34), neutrophilia (n=84), lymphocytosis (n=13), increased total cell counts (n=49) and positive polymerase chain reaction of Pneumocystis jirovecii DNA (n=7). Diagnoses considering the BAL results included bacterial pneumonia (n=10), viral pneumonia (n=5), fungal pneumonia (n=1), acute exacerbations of interstitial pneumonia (n=29), diffuse alveolar hemorrhage (n=34) and pneumocystis pneumonia (n=10). No significant differences were found in the P/F ratio between before and after the procedure of BAL (172 vs. 174, p=0.33). In the receiver operating characteristic (ROC) curve analysis, low lymphocyte fraction in BAL was poor prognostic factor (AUC, 0.81; 95%CI, 0.71-0.91). In the multivariate analysis, low lymphocyte fraction in BAL was the independent poor prognostic factor (p=0.0004; hazard ratio, 0.92; 95%CI, 0.88-0.99) after adjustment by age, sex, APACHE II score and SOFA score.

**Conclusions:** BAL provided additional information for the clinically-diagnosed ARDS patients. Lymphocyte fraction in BAL sample could be a useful prognostic factor.

## P249 Oxygen delivery, carbon dioxide removal, energy transfer to the lungs and pulmonary hypertension behavior during venous-venous extracorporeal membrane oxygenation support: a mathematical modeling approach

### BA Besen^1^, LM Melro^1^, TG Romano^2^, PV Mendes^1^, M Park^1^

#### ^1^Hospital das Clínicas HCFMUSP, Faculdade de Medicina, Universidade de São Paulo, São Paulo, Brazil; ^2^ABC Medical School, Santo Andre, Brazil

**Introduction:** Our objective is to describe and analyze, in a hypothetical patient with severe acute respiratory distress syndrome (ARDS), the following: (1) the energy transfer from the ventilator to the lungs; (2) venous-venous extracorporeal membrane oxygenation (VV-ECMO) oxygen transfer to patient oxygen consumption (VO2) match; (3) ECMO carbon dioxide removal, and (4) the potential effect of systemic venous oxygenation on pulmonary artery pressure.

**Methods:** Mathematical modeling approach with hypothetical scenarios, using computer simulation.

**Results:** The transition from protective ventilation to ultraprotective ventilation in a severe ARDS patient with static respiratory compliance of 20 mL/cmH2O reduced the energy transfer from the ventilator to the lungs from 35.3 to 2.6 Joules/minute. A hypothetical patient with VO2 = 200 mL/minute, high cardiac output and slightly anemic can reach an arterial oxygen saturation of 80%, while keeping the match between ECMO oxygen transfer and patient VO2 (Fig. 1). Carbon dioxide is easily removed and normal PaCO2 is frequently reached. The venous blood oxygenation through ECMO circuit drives the PO2stimulus of pulmonary hypoxic vasoconstriction to normal values (Fig. 2).

**Conclusions:** Ultraprotective ventilation massively reduces the energy transfer from the ventilator to the lungs. Severe hypoxemia on VV-ECMO support may occur despite matching between ECMO oxygen transfer and patient’s VO2. Normal range of PaCO2 is easy to reach. VV-ECMO support potentially relieves hypoxic pulmonary vasoconstriction.

**Fig. 1 (abstract P249).**
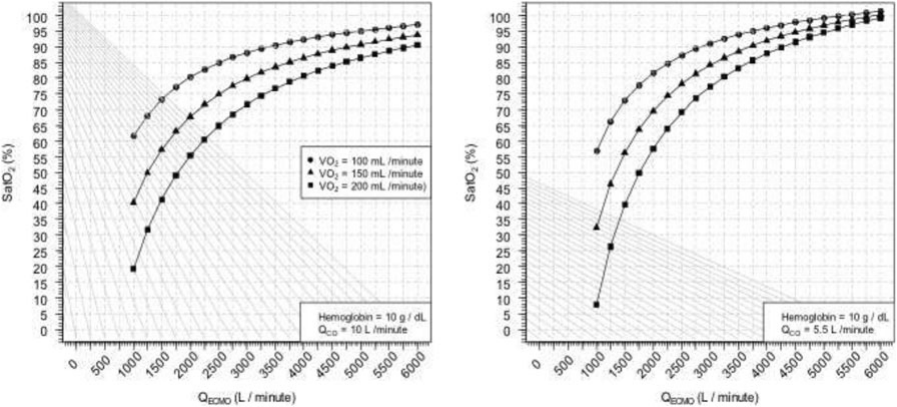
SatO2 according to progressive QECMO increment, in different VO2 levels and a QLshunt fixed at 95%. Panel A shows this relation with QCO of 10 L/minute and Panel B shows this relation with QCO of 5.5 L/minute. VO2: oxygen consumption; QCO: Cardiac Output; QECMO: ECMO blood flow; QLshunt: Shunt fraction

**Fig. 2 (abstract P249).**
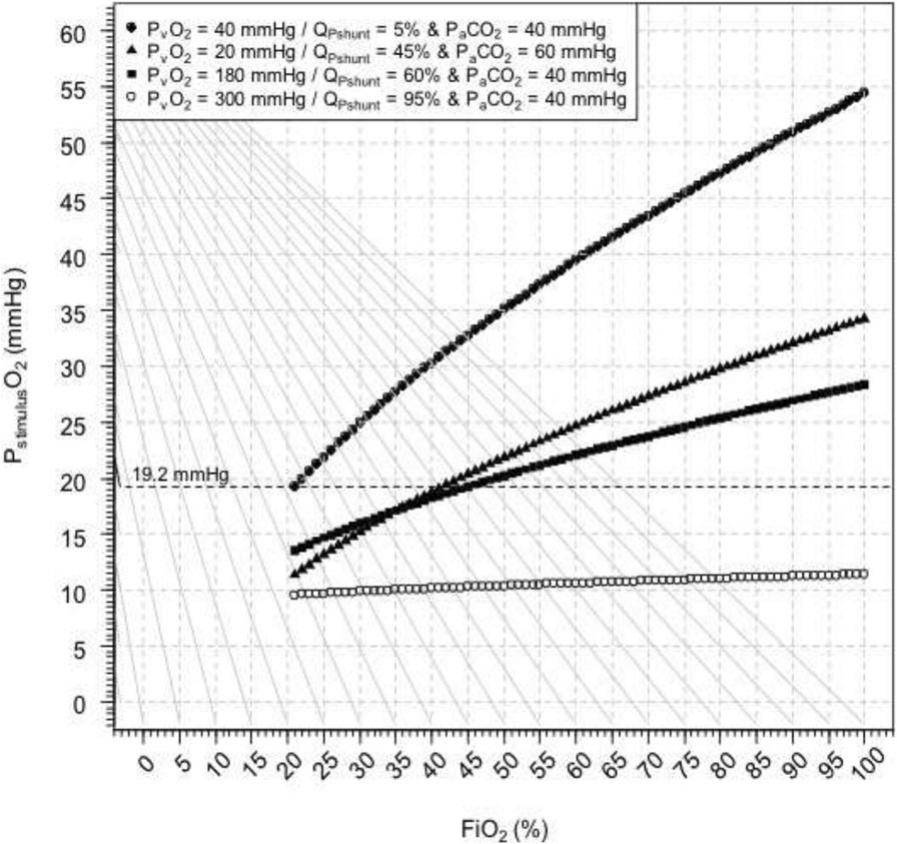
Oxygen partial pressure responsible for the hypoxic pulmonary vasoconstriction inhibition in four different clinical scenarios. The dotted line at PstimulusO2 of 19.2 mmHg represents the partial pressure during breathing of a healthy person. The other clinical scenarios reproduce mechanical ventilation in a severe ARDS patient with hypercapnia, pulmonary shunt fraction of 45%, and PvO2 = 20 mmHg (closed triangle); the closed squares represent this same patient after ECMO initiation, when the pulmonary shunt increased to 60% (Whitening-up phenomenon) and the PvO2 increased to 180 mmHg; at last, the open circles represent this same patient with a pulmonary shunt of 95% and a PvO2 of 300 mmHg

## P250 Early recovery from acute kidney injury during venovenous ECMO associates with improved survival in severe ARDS

### S Gaião, C Meng, R Vilares-Morgado, R Roncon-Albuquerque, J Paiva

#### São João Hospital Center, Oporto, Portugal

**Introduction:** Acute kidney injury (AKI) is frequently observed in patients with severe acute respiratory distress syndrome (ARDS) rescued with veno-venous extracorporeal membrane oxygenation (VV-ECMO) and has been associated with a negative impact in patient outcome. In the present study, we analyzed renal function of ARDS patients requiring VV-ECMO support and its association with clinical outcomes.

**Methods:** Single-center retrospective study of patients (n=147; 45±11.9 years; 63% males) undergoing VV-ECMO for severe ARDS. Renal function was evaluated before VV-ECMO initiation and at ECMO-Day-1, -Day-3 and -Day-7, using the Kidney Disease: Improving Global Outcomes (KDIGO) AKI classification.

**Results:** At intensive care unit admission, the median Simplified Acute Physiology Score II (SAPS-II) and Sequential Organ Failure Assessment (SOFA) scores were 45±15.9 and 9±3.1, respectively. Hospital mortality was 29.0%. At VV-ECMO initiation 86 patients (58.5%) had AKI, of which 54 (62.8%) improved renal function in the first week of VV-ECMO support. Patients with early recovery from AKI had lower SOFA (9±2.4 vs. 12±3.4), lactate (1.9±0.86 vs. 3.2±3.04; mM), renal replacement therapy (3.7 vs. 75.0; %) and hospital mortality (11.3 vs. 43.8; %), compared with patients without early AKI recovery. Regarding mechanical ventilation parameters before VV-ECMO initiation, patients with early recovery from AKI presented lower plateau pressure (30±6.1 vs. 35±8.9; cmH2O), lower driving pressure (18±5.9 vs. 22±8.1; cmH2O) and higher static respiratory system compliance (31±16.5 vs. 21±9.9; mL/cmH2O), compared with patients without early AKI recovery.

**Conclusions:** In severe ARDS, AKI is frequently observed at VV-ECMO initiation. Nevertheless, patients showing early recovery from AKI during VV-ECMO present low hospital mortality. The potential impact of mechanical ventilation parameters in AKI recovery of patients with severe ARDS requiring VV-ECMO deserves further investigation.

## P251 Feasibility and safety of low-flow extracorporeal CO2 removal managed with a renal replacement platform to enhance lung-protective ventilation of patients with mild-to-moderate ARDS

### M Schmidt^1^, S Jaber^2^, E Zogheib^3^, T Godet^4^, G Capellier^5^, A Combes^6^

#### ^1^APHP, Pitie Salpetrière, Paris, France; ^2^CHU de Montpellier, Montpellier, France; ^3^chu amiens, Amies, France; ^4^Centre Hospitalier Universitaire (CHU) Clermont-Ferrand, Clermont-Ferrand, France; ^5^Besançon University Hospital, Besançon, France; ^6^APHP, Paris, France

**Introduction:** Extracorporeal carbon-dioxide removal (ECCO2R) might allow ultraprotective mechanical ventilation with lower tidal volume (VT) (<6 mL/kg predicted body weight), plateau (Pplat) (<30cmH2O) and driving pressures to limit ventilator-induced lung injury. This study was undertaken to assess the feasibility and safety of ECCO2R managed with a renal replacement therapy (RRT) platform to enable ultraprotective ventilation of patients with mild-to-moderate ARDS.

**Methods:** 20 patients with mild (n=8) or moderate (n=12) ARDS were included. VT was gradually lowered from 6 to 5, 4.5 and 4 mL/kg, and PEEP adjusted to reach 23<=Pplat<=25 cm H2O. Stand-alone ECCO2R (PRISMALUNG, no hemofilter associated with the RRT platform) was initiated when arterial PaCO2 increased by >20% from its initial value. Ventilation parameters (VT, RR, PEEP), respiratory system compliance, Pplat and driving pressure, arterial blood gases, and ECCO2R-system characteristics were collected during at least 24 hours of ultraprotective ventilation. Complications, day-28 mortality, need for adjuvant therapies, and data on weaning off ECCO2R and mechanical ventilation were also recorded.

**Results:** While VT was reduced from 6 to 4 mL/kg and Pplat kept <25 cmH2O, PEEP was significantly increased from 13.4±3.6 at baseline to 15.0±3.4 cm H2O, and the driving pressure was significantly reduced from 13.0±4.8 to 7.9±3.2 cm H2O (both p<0.05). The PaO2/FiO2 ratio and respiratory-system compliance were not modified after VT reduction. Mild respiratory acidosis occurred, with mean pH decreasing from 7.39 ± 0.1 to 7.32 ± 0.10 from baseline to 4-mL/kg VT. Mean extracorporeal blood flow, sweep-gas flow and CO2 removal were 421±40 mL/min, 10±0.3 L/min and 51±25 mL/min, respectively. Mean treatment duration was 31±22 hours. Day-28 mortality was 15%.

**Conclusions:** A low-flow ECCO2R device managed with an RRT platform easily and safely enabled ultraprotective mechanical ventilation in patients with mild-to-moderate ARDS. (ClinicalTrials: NCT02606240)

## P252 Thromboelastography-based anticoagulation management during extracorporeal membrane oxygenation: a safety and feasibility pilot study

### M Panigada^1^, G Iapichino^2^, M Brioni^2^, G Panarello^3^, A Protti^1^, G Grasselli^1^, G Occhipinti^3^, C Novembrino^1^, D Consonni^1^, A Arcadipane^3^, L Montalbano^2^, L Gattinoni^4^, A Pesenti^1^

#### ^1^Fondazione IRCCS Ca’ Granda Ospedale Maggiore Policlinico, Milano, Italy; ^2^University of Milan, Milano, Italy; ^3^ISMETT IRCCS (Istituto Mediterraneo per i Trapianti e Terapie ad Alta Specializzazione), Palermo, Italy; ^4^University of Göttingen, Göttingen, Germany

**Introduction:** There is no consensus on the management of anticoagulation during extracorporeal membrane oxygenation (ECMO). ECMO is currently burdened by a high rate of hemostatic complications, possibly associated with inadequate monitoring of heparin anticoagulation. This study aims to assess the safety and feasibility of an anticoagulation protocol for patients undergoing ECMO based on Thromboelastography (TEG) as opposed to an activated partial thromboplastin time (aPTT)-based protocol.

**Methods:** We performed a multicenter, randomized, controlled trial in two academic tertiary care centers. Adult patients with acute respiratory failure treated with veno-venous ECMO were randomized to manage heparin anticoagulation using a TEG-based protocol (target 16-24 minutes of the R parameter, TEG group), or a standard of care aPTT-based protocol (target 1.5-2 of aPTT ratio, aPTT group). Primary outcomes were safety and feasibility of the study protocol.

**Results:** Forty-two patients were enrolled, 21 were randomized to the TEG group and 21 to the aPTT group. Duration of ECMO was similar in the two groups (9 (7-16) days in the TEG group and 11 (4-17) days in the aPTT group, p=0.74). Heparin dosing was lower in the TEG group compared to the aPTT group (11.7 (9.5-15.3) IU/kg/h versus 15.7 (10.9-21.3) IU/kg/h respectively, p=0.03). Safety parameters, assessed as number of hemorrhagic or thrombotic events and transfusions given, were not different between the two study groups. As for the feasibility, the TEG-based protocol triggered heparin infusion rate adjustments more frequently (p<0.01) and results were less frequently in the target range compared to the aPTT-based protocol (p<0.001). Number of prescribed TEG or aPTT controls (according to study groups) and protocol violations were not different between the study groups.

**Conclusions:** TEG can be safely used to guide anticoagulation management during ECMO. Its use was associated with the administration of lower heparin doses compared to a standard of care aPTT-based protocol.

## P253 Determinants and prognostic implications of the acute cor pulmonale clinical risk score in severe ARDS rescued with venovenous ECMO

### C Basílio, J Artur Paiva, R Roncon-Albuquerque

#### Intensive Care Medicine Department, Centro Hospitalar de S.João, Porto, Portugal

**Introduction:** The aim of the present study was to analyze the determinants and prognostic implications of the recently proposed acute cor pulmonale (ACP) clinical risk score(1) in patients with severe acute respiratory distress syndrome (ARDS) rescued with venovenous (VV) extracorporeal membrane oxygenation (ECMO).

**Methods:** Single-center retrospective study of patients (n=152; 45±11.8 years; 63% males) undergoing VV-ECMO for severe ARDS. The ACP-Score (0-4) was calculated immediately before ECMO initiation and at ECMO-Day1, -Day3 and -Day7, as follows: pneumonia as cause of ARDS - 1 point; driving pressure >=18cmH2O - 1 point; PaO2/FiO2 ratio <150mmHg - 1 point; PaCO2 >=48mmHg - 1 point.

**Results:** Longer duration of mechanical ventilation before VV-ECMO was associated with higher ACP-Scores. Patients with higher ACP-Scores before VV-ECMO also presented longer total duration of mechanical ventilation and hospital stay. After VV-ECMO initiation, ACP-Scores significantly decreased from 3.0±0.74 to 1.5±0.84, 1.5±0.96 and 1.6±0.99 at ECMO-Day1, -Day3 and -Day7, respectively. At ECMO-Day7, patients with higher ACP-Scores (3-4) presented increased hospital mortality when compared with patients with lower ACP-Scores (0-2): 47.6 vs. 24.7%, respectively (p=0.038). At ECMO-Day7, high driving pressures and low PaO2/FiO2 ratios were the ACP-Score determinants that significantly associated with increased hospital mortality.

**Conclusions:** In severe ARDS, VV-ECMO support allowed a significant and sustained ACP-Score reduction in most patients. This was achieved by artificial lung correction of low PaO2/FiO2, hypercapnia and elevated driving pressures. After an initial period of VV-ECMO support, patients with higher ACP-Scores present higher mortality rates. Our results suggest that on-going adjustment of ECMO and ventilation parameters is necessary to maximize outcome.

**Reference**

1. Mekontso Dessap A et al. Intensive Care Med 42:862-870, 2016

## P254 Mechanical power decreased in acute respiratory distress syndrome patients supported with extracorporeal membrane oxygenation

### T Vossler, S Nonas

#### Oregon Health & Science University, Portland, OR, USA

**Introduction:** We sought to use mechanical power to describe “lung rest” in patients with acute respiratory distress syndrome (ARDS) supported with extracorporeal membrane oxygenation (ECMO). Mechanical power describes work done by the ventilator on the patient’s respiratory system over time. This concept unifies tidal volume, rate, and total pressure delivered during the ventilatory cycle into a discrete value that may be useful to guide ventilatory support. We hypothesized that initiation of ECMO led to decreased mechanical power delivered to the patient.

**Methods:** We reviewed the charts of the three medical intensive care unit patients at our institution supported with ECMO for severe ARDS. We collected data on plateau pressure, driving pressure, and mechanical power before initiating ECMO, then at <6 hours, 24 hours, and 72 hours after. We calculated the mechanical power delivered by the ventilator to the patient in Joules per minute as 0.098 x respiratory rate x tidal volume x (peak pressure - ½ x driving pressure)[1].

**Results:** All patients were alive at discharge and at 90 days. Mean PaO_2_/FiO_2_ at ECMO initiation was 64±38, mean plateau pressure was 37±3 cm water. All patients received neuromuscular blockade at initiation of ECMO. Following ECMO initiation, mechanical power decreased by an average of 58%±14% initially, by 69%±4% at 24 hours, and by 66%±17% at 72 hours (Fig. 1). By comparison, driving pressure changed by an average value of -0.3±8.0, -0.3±5.5, and -2.0±4.6 cm water over those same intervals. Average plateau pressure changed by -3.3±5.7, -4.7±5.5, and -1.7±6.4 cm water during the same time period (Fig. 2).

**Conclusions:** In our limited case series, mechanical power decreased significantly following initiation of ECMO in patients with severe ARDS. We suggest mechanical power may be more useful than changes in driving pressure or plateau pressure when pursuing “lung rest” during ECMO.

**Reference**

1. Gattinoni L, et al. Intensive Care Med. 42(10):1567-1575, 2016

**Fig. 1 (abstract P254).**
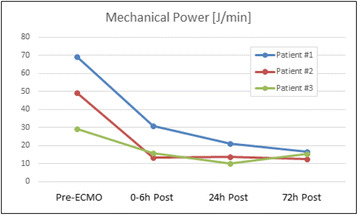
Change in mechanical power after ECMO initiation

**Fig. 2 (abstract P254).**
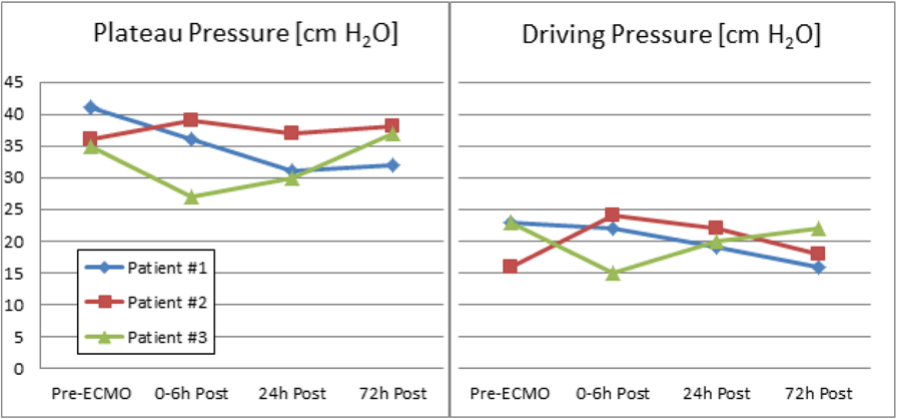
Change in driving pressure and plateau pressure after ECMO initiation

## P255 Attributable mortality of acute respiratory distress syndrome: a systematic review and meta-analysis

### LK Torres, JS Harrington, C Oromendia, AM Choi, II Siempos

#### Weill Cornell Medicine, New York City, New York, USA

**Introduction:** It is not clear whether acute respiratory distress syndrome (ARDS) is independently associated with mortality after controlling for underlying risk factor and baseline severity of illness. We attempted to assess the attributable mortality of ARDS by performing a systematic review and meta-analysis.

**Methods:** We systematically searched PubMed, EMBASE, Scopus and reference lists to identify observational studies reporting mortality rates of critically ill patients with and without ARDS. All included studies were matched for underlying risk factor. Primary outcomes were all-cause in hospital-mortality and short-term mortality (combined 28 day-mortality and intensive care unit-mortality). We calculated pooled risk ratios (RR) and 95% confidence intervals (CI) with a random-effects model. Our meta-analysis was registered with PROSPERO.

**Results:** Of the 3119 initially retrieved articles, 41 studies (44 cohorts) involving 58408 patients were included. The underlying risk factor was sepsis, trauma and other in 15, 18 and 11 cohorts, respectively. In-hospital mortality was higher in patients with versus without ARDS (31 cohorts; 54101 patients; RR 2.63, 95% CI 2.01-3.44; P<0.001). We saw a numerically stronger association between ARDS and in-hospital mortality in trauma (RR 3.15, 95% CI 2.17-4.57; P<0.001) than sepsis (RR 1.80, 95% CI 1.24-2.63; P=0.002). Short-term mortality was higher in patients with versus without ARDS (14 cohorts; 8040 patients; RR 1.88, 95% CI 1.27-2.78; P=0.002). ARDS was independently associated with mortality in approximately half of the 11 cohorts which controlled for baseline severity of illness using a multivariable analysis.

**Conclusions:** The accumulated evidence suggests that ARDS is independently associated with mortality after controlling for underlying risk factor; the association is stronger for trauma than septic patients. Evidence is mixed as to whether ARDS is independently associated with mortality after controlling for baseline severity of illness.

## P256 Attributable mortality of acute respiratory distress syndrome in critically ill septic patients

### LK Torres^1^, EJ Finkelsztein^2^, C Oromendia^1^, EJ Schenck^1^, A Higuera^3^, RM Baron^3^, LE Fredenburgh^3^, JW Huh^4^, AM Choi^1^, II Siempos^1^

#### ^1^Weill Cornell Medicine, New York, USA; ^2^New York-Presbyterian Brooklyn Methodist Hospital, Brooklyn, New York, USA; ^3^Brighman and Women’s Hospital, Harvard Medical School, Boston, MA, USA; ^4^Asan Medical Center, University of Ulsan College of Medicine, Seoul, South Korea

**Introduction:** Evidence is mixed as to whether acute respiratory distress syndrome (ARDS) is independently associated with mortality after controlling for baseline severity of illness, particularly in patients with sepsis.

**Methods:** This was an observational study comparing mortality rates of septic patients with and without ARDS. Subjects for the present study were enrolled in 3 ongoing prospective cohorts of critically ill patients hospitalized in medical intensive care unit (ICU) in the United States or South Korea. ARDS was defined using the Berlin definition for cases after 2012 and the American–European Consensus Conference definition for cases before 2012. Sepsis was defined using the Sepsis-3 definition. Baseline severity of illness was assessed using a modified sequential organ failure assessment (SOFA) after exclusion of the respiratory component. The primary outcome was in-hospital mortality.

**Results:** Of the 1024 critically ill patients enrolled in the 3 cohorts, 771 (75.3%) had sepsis and comprised the population of the present study. Of the 771 septic patients, 166 (21.5%) had ARDS. Patients with versus without ARDS had higher SOFA score; both total (median 14 vs 11; P<0.001) and modified (11 vs 10; P<0.001). The unadjusted mortality of septic patients with ARDS was higher than septic patients without ARDS (46.7% vs 22.4%; P<0.001). After controlling for baseline modified SOFA score, both moderate and severe ARDS remained significant predictors for in-hospital mortality [odds ratio (OR) 2.90; 95% confidence intervals (CI) 1.66-5.03; P<0.001 and OR 3.91; 95% CI 2.33-6.58; P<0.001, respectively]. In contrast, after controlling for baseline modified SOFA score, mild ARDS was not associated with in-hospital mortality (OR 1.04; 95% CI 0.40-2.39; P=0.94).

**Conclusions:** Among critically ill patients with sepsis, moderate and severe, but not mild, ARDS are associated with mortality after controlling for baseline severity of illness.

## P257 A multicenter study on the inter-rater reliability of HEART score among emergency physicians from three Italian emergency departments

### N Parenti^1^, ML Bacchi Reggiani^2^, S Morselli^1^, G Pezzuto^1^, F Agrusta^1^, N Amadori^1^, V Casolari^1^, M Volpe^1^, G Farina^2^, A Di Miccoli^2^, L Bonfanti^3^, F Numeroso^3^, A Vegetti^1^, F Pileri^1^, S Menetti^1^, M Nuzzetti^1^, G Tomassoli^1^, M Ravazzini^1^, M Buda^1^, F Mori^1^, M Cavazza^2^, G Cervellin^3^, A Pietrangelo^1^, L Brugioni^1^, A Luciani^1^

#### ^1^Via del Pozzo 71 Modena 41124, Bologna, Italy; ^2^Policlinico Sant’Orsola, Bologna, Italy; ^3^AOU Parma, Parma, Italy

**Introduction:** Previous studies suggested that the HEART (based on History, ECG, Age, Risk Factors, Troponin) score could be a valid tool to manage the patients with chest pain at the Emergency Department (Fig. 1). Our hypothesis was that there could be heterogeneity in the assignment, because of the History and ECG parameters. For this reason, our objective was to test the HEART reliability. There are no published studies on this topic.

**Methods:** This is a multicenter retrospective study conducted in 3 Italian EDs between March and October 2017 using clinical scenarios. Twenty emergency physicians were included, provided that they had undergone a course on HEART score. We used 53 scenarios from a medical database with each scenario including demographic and clinical characteristics. Each participant assigned scores to the scenarios using the HEART. We tested the measure of interrater agreement using the kappa-statistic, the confidence intervals are bias corrected. A p-value of <0.05 was used to define statistical significance.

**Results:** The participants’ assignment is shown in Fig. 2. The overall inter-rater reliability was good: Kappa = 0.63 (CI 95%; 0.57 – 0.72); with a good agreement between the low and high class of risk but a moderate reliability in the medium class: Kappa= 0.72, 0.70 and 0.51.

We have not found differences of inter-rater reliability among the senior (more than 5 yrs in ED) and junior physicians: Kappa= 0.65 (CI 95%; 0.57 – 0.73) and 0.60 (CI 95%; 0.51 - 0-72).The HEART score showed the worse value of inter-rater reliability in the History and ECG parameters : K inter = 0.37 (CI 95%; 0.33 – 0.44) and 0.42 (CI 95%; 0.29 – 0.50).

**Conclusions:** The HEART showed a good inter-rater reliability but a fair agreement in the History parameter. The clinical experience doesn’t influence the agreement in the assignment. The main limit of this study lies in using scenarios rather than real patients.

**Fig. 1 (abstract P257).**
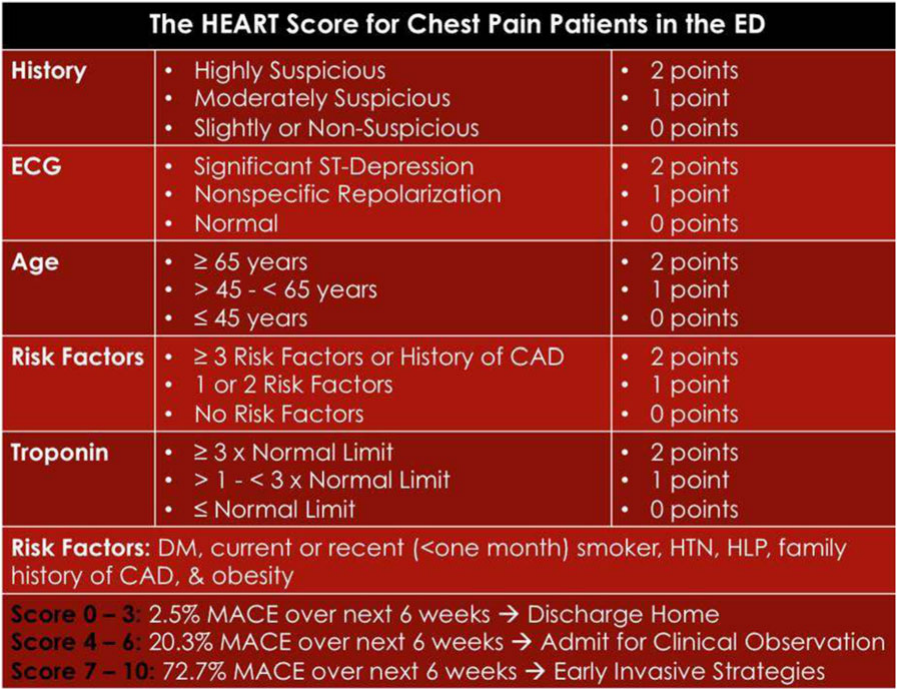
The HEART Score for chest pain patients in the ED

**Fig. 2 (abstract P257).**
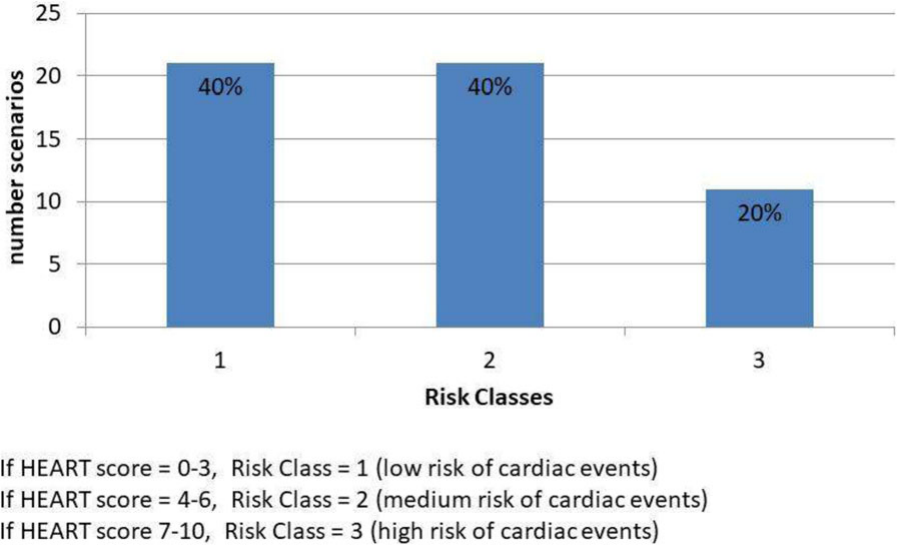
HEART score risk classes

## P258 Influence of hypoxic and hyperoxic preconditioning on endothelial function markers in a model of myocardial ischemia/reperfusion injury: experimental study

### I Mandel^1^, Y Podoksenov^2^, S Mikheev^3^, A Podoksenov^2^, J Svirko^2^, A Gusakova^2^, I Suhodolo^4^, V Shipulin^2^

#### ^1^Sechenov University, Moscow, Russia; ^2^Cardiology Research Institute, Tomsk, Russia; ^3^Treatment and Rehabilitation Center, Moscow, Russia; ^4^Siberian State Medical University, Tomsk, Russia

**Introduction:** The aim of the experiment was to study the efficacy of preconditioning, based on changes in inspiratory oxygen fraction on endothelial function in a model of myocardial ischemia/reperfusion injury in conditions of cardiopulmonary bypass (CPB).

**Methods:** The prospective study included 32 rabbits divided into four equal groups: hypoxic preconditioning; hyperoxic preconditioning (HyperP); hypoxic-hyperoxic preconditioning (HHP); control group. Animals were anesthetized and mechanically ventilated. We provided preconditioning, then started CPB, and then induced acute myocardial infarction by ligation of left anterior descending artery. After 45 minutes of ischemia we performed 120 minutes of reperfusion. We investigated endothelial function markers (endothelin-1 (ET-1), asimmetric dimethylarginine (ADMA), nitric oxide metabolites) at stages before ischemia (after preconditioning in study groups), after ischemia and after reperfusion.

**Results:** The level of ET-1 after the stage of ischemia increased in all groups, a significant difference was between HHP and control group (p=0.006), then ET-1 increased even more after the stage of reperfusion (p=0.003 HHP vs control group). The concentration of nitrite decreased after the stages of ischemia and reperfusion in comparison with the baseline in all groups. However, the level of nitrite after all types of preconditioning was higher than in the control group (p=0.016; 0.046; 0.009). The total concentration of nitric oxide metabolites in the study groups was higher than in the control group: before ischemia (after preconditioning) p=0.034; after ischemia p=0.014; after reperfusion, p=0.022. Concentration of ADMA was lower in the HHP comparing with the control group at the stages after ischemia (p=0.006) and after reperfusion (p=0.027).

**Conclusions:** HyperP and HHP maintain endothelial function: the balance of nitric oxide metabolites and the reduction of ET-1 hyperproduction in a model of myocardial ischemia/reperfusion injury in conditions of CPB.

## P259 Upscaling hemodynamic and brain monitoring during major cancer surgery: a before-after comparison study

### M Lima^1^, L Mondadori^1^, A Chibana^1^, D Gilio^1^, E Giroud^1^, F Michard^2^

#### ^1^A.C. Camargo Cancer Center, São Paulo, Brazil; ^2^MiCo, Denens, Switzerland

**Introduction:** Hemodynamic and brain monitoring are used in many high-risk surgical patients without well-defined indications and objectives. In order to rationalize both hemodynamic and anesthesia management, we implemented monitoring guidelines for patients undergoing major cancer surgery.

**Methods:** Early 2014, and for all eligible patients, we started to recommend (Standard Operating Procedure, SOP) cardiac output, central venous oxygen saturation, and depth of anesthesia monitoring with specific targets (MAP > 65 mmHg, SVV < 12%, CI > 2.5 l/min/m^2^, ScvO2 > 75%, 40 < BIS < 60). Eligibility criteria were pelvic or abdominal cancer surgery expected to last > 2 hours in adult patients. Pre-, intra-, and post-operative data were collected from our electronic medical record (EMR) database and compared before (from March to August 2013) and after (from March to August 2014) the SOP implementation.

**Results:** A total of 596 patients were studied, 313 before and 283 after the SOP implementation. The two groups were comparable in terms of age, ASA score, duration and type of surgery, The surgical POSSUM score was higher after than before (20 vs 18, p=0.045). The use of cardiac output, ScvO2 and BIS monitoring increased from 40 to 61%, 61 to 81%, and 60 to 88%, respectively (all p values < 0.05). Intraoperative fluid volumes decreased (16.9 vs 15.2 ml/kg/h, p=0.002), whereas the use of inotropes increased (6 vs 13%, p=0.022). The rate of postoperative delirium (16 vs 8%, p=0.005) and urinary track infection (6 vs 2%, p=0.012) decreased, as well as the median hospital length of stay (9.6 vs 8.8 days, p=0.032).

**Conclusions:** In patients undergoing major surgery for cancer, despite an increase in surgical risk, the implementation of guidelines with predefined targets for hemodynamic and brain monitoring was associated with a significant improvement in postoperative outcome.

## P260 Base deficit and severe maternal outcomes in critically ill patients with severe preeclampsia

### C Bello-Muñoz^1^, G Monsalve^2^, A Cardona^1^, A Paternina-Caicedo^3^, F Camargo^4^, C Lopez^5^, A Castro^6^, O Lavalle^7^, R Padron^2^, M Vasco^2^, J Rojas-Suarez^8^

#### ^1^Clinica del Prado, Medellin, Colombia; ^2^Grupo de investigacion en medicina critica y cuidados intensivos (Gricio), Cartagena, Colombia; ^3^Universidad de cartagena, Cartagena, Colombia; ^4^Clinica casa del niño, Monteria, Colombia; ^5^ESE la divina misericordia, Magangue, Colombia; ^6^Clinica de la mujer, Bogota, Colombia; ^7^Clinica Santa cruz de bocagrande, Cartagena, Colombia; ^8^Gestion salud IPS, Cartagena, Colombia

**Introduction:** Tissue perfusion and oxygen delivery is low in patients with severe preeclampsia, which would explain multiple organ failure and death in these patients. The aim of this study was to determine the relationship between the base deficit and the risk of adverse maternal and perinatal outcomes.

**Methods:** Retrospective multicenter cohort study included pregnant patients with severe preeclampsia admitted to six intensive care units at tertiary referral centers during a ten years period in Colombia. Clinical information was gathered from hospital medical records. The correlation of base deficit with adverse maternal outcomes was evaluated using logistic regression analysis. Outcomes were maternal death, acute kidney injury, HELLP syndrome, transfusion, eclampsia and extreme neonatal morbidity.

**Results:** 731 patients were included in the study, we found a total of 21 (2,8%) maternal deaths, the median calculated base deficit obtained was -5.5 meq/L. Patients with base deficit greater than -8.0meq/L had significantly higher mortality rates OR 3.02 (CI 1.26-7.2) P 0,013. This group of patients was also associated with a higher probability of developing a class 1 HELLP syndrome OR 1.7 (CI 1.02-2.82) P 0,03.

A more mild alteration in the base deficit (greater than -5.0meq/L) was related to the appearance of kidney injury OR 2.25 (CI 1.52-3.34) P 0.00 y complete HELLP OR 2.17 (CI 1.60-2.96) P 0.00.

**Conclusions:** Base deficit is related to worse outcomes in patients with severe preeclampsia. According to our results, a cut-off point greater than -8meq/L, there is a higher risk of death and worse outcomes such as class 1 HELLP syndrome.

## P261 Comparison of two different laser speckle contrast imaging devices to assess skin microcirculatory blood flow

### G Guven, Y Ince, OI Soliman, S Akin, C Ince

#### Erasmus MC, University Medical Center Rotterdam, Rotterdam, Netherlands

**Introduction:** Laser speckle contrast imaging (LSCI) is a common, non-contact and practical method used to assess blood flow of tissue surfaces. We have lack of knowledge about comparability of different LSCI devices due to the arbitrary units (AU) used to define blood flux. We sought to examine the linearity between skin blood flux, recorded using two different LSCI devices.

**Methods:** We performed post-occlusive reactive hyperemia test (PORH) on the arm and measured blood flux on the hand using two different LSCI devices (Moor Instruments, Devon, UK and Perimed AB, Järfälla, Sweden). All volunteers were measured at baseline, during occlusion and after release of occlusion during the hyperemia phase. The third finger and fourth finger nail were selected for recording blood flux and AU were used to express values.

**Results:** Fifteen healthy, non-smoker male volunteers participated in this study. An excellent correlation was found between the two LSCI devices (finger: R2:0.79, p<0.001 & finger nail: R2:0.68, p<0.001). Data were also assessed in terms of the variability at different stages of the PORH test (Fig. 1a-d). Correlation of devices was still high at baseline, first minute of occlusion and in the post-occlusion hyperemia phase. However, in the period between 1 minute after start of the occlusion and the beginning of the hyperemia, correlation was lower for the whole finger (R2:0.21, p=0.002) and correlation was lost for fingernail (R2:0.05, p=0.14) between the two devices.

**Conclusions:** Skin blood flux measured with two different LSCI devices are linearly correlated with each other. However care should be taken when assessing patients with low blood flux such as occurs during shock and ischemic organs.

**Fig. 1 (abstract P261).**
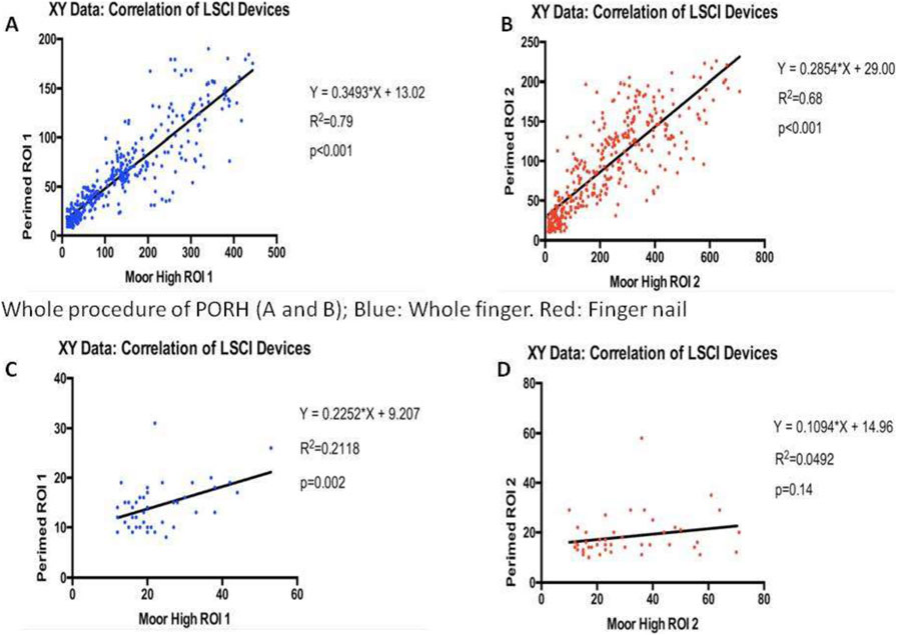
See text for description

## P262 Microvascular effects of hyperoxia and mild hypoxia in anesthetized rats

### E Damiani, C Scorcella, S Ciucani, S Bolognini, N Mininno, A Donati

#### Università Politecnica delle Marche, Ancona, Italy

**Introduction:** The aim of this study was to evaluate the effects of hyperoxia and mild hypoxia on microcirculatory perfusion in a rat model.

**Methods:** Spontaneously breathing anesthetized (isoflurane) male Wistar rats (n=12) were equipped with arterial (left carotid) and venous (right jugular) cannulae and tracheotomy. Rats were randomized in 3 groups: normoxia – inspired oxygen fraction (FiO_2_) of 0.21; hyperoxia – FiO_2_ 1; mild hypoxia – FiO_2_ 0.15. The following measurements were taken hourly for 4 hours: blood gases, mean arterial pressure (MAP), stroke volume index (SVI) and heart rate (echocardiography), skeletal muscle microvascular density (sidestream dark field videomicroscopy).

**Results:** At 1 hour, arterial O2 tension was 103±19 mmHg in normoxia, 296±60 mmHg in hyperoxia, 62±8 mmHg in mild hypoxia (p<0.001). Hyperoxia induced an increase in MAP (from 109±13 to 129±8 mmHg at 1h, p<0.05) and a decrease in SVI (from 0.67±0.1 to 0.59±0.1 ml/kg at 1h, p<0.05), while in mild hypoxia MAP tended to decrease and SVI tended to increase (p>0.05). Microvascular density decreased in hyperoxia and increased in mild hypoxia (Fig. 1).

**Conclusions:** In anesthetized rats, microvascular density decreased with hyperoxia and increased with mild hypoxia.

**Fig. 1 (abstract P262).**
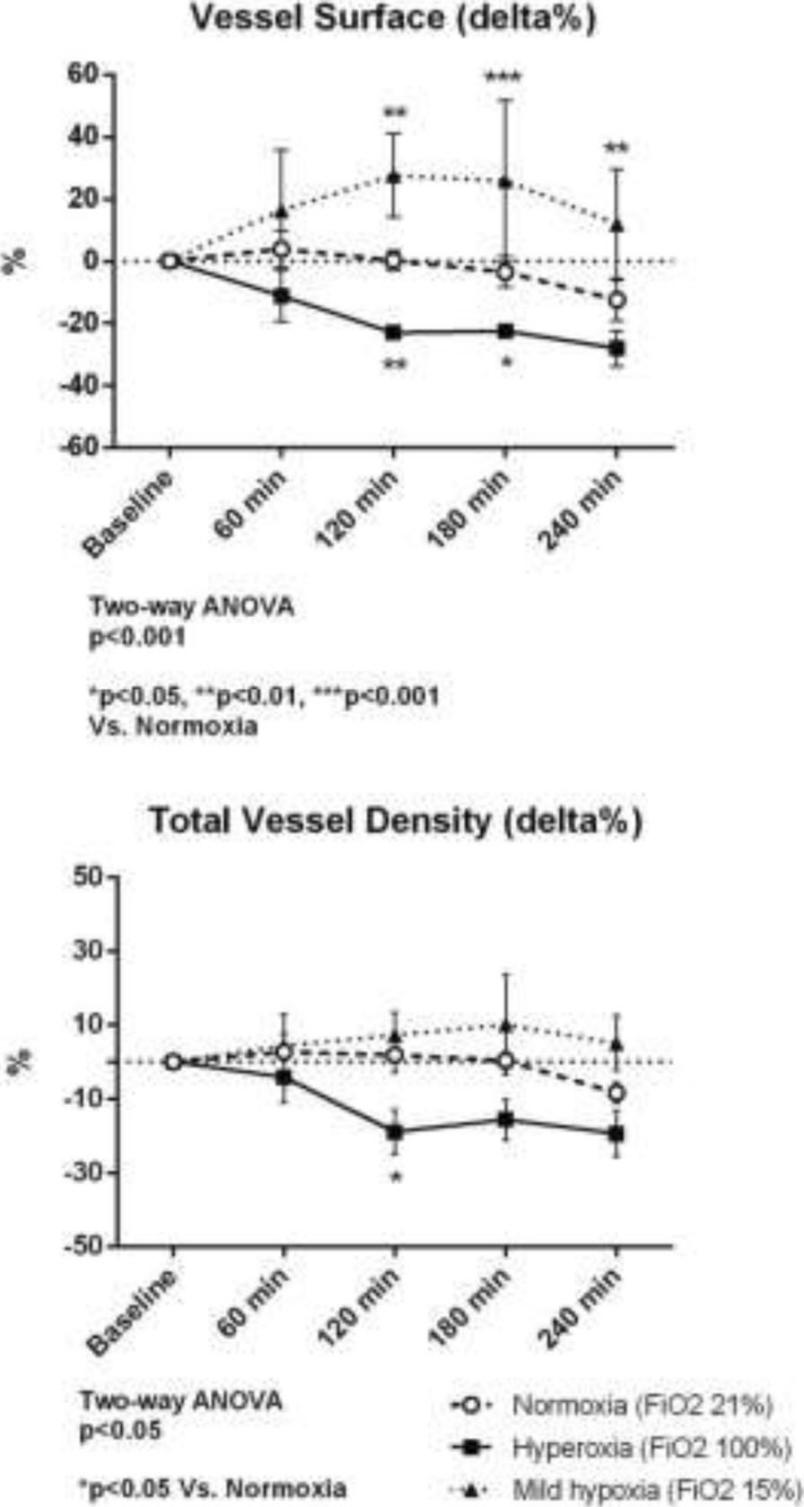
Changes in microvascular density

## P263 Relationship between high central venous oxygen saturation and mortality in patients with sepsis

### T Sricharoenchai, A Kanitsap, N Saiphoklang, P Rujiwit, P Pirompanich

#### Thammasat University, Pathum Thani, Thailand

**Introduction:** The imbalance between oxygen (O2) delivery and O2 requirement in patients with sepsis can be assessed by central venous oxygen saturation (ScvO2). The low or high ScvO2 may indicate cellular hypoxia or inability to utilize the O2. This study aims to determine the relationship between high ScvO2 and mortality in patients with sepsis.

**Methods:** A retrospective observational cohort study was done by collecting data (i.e., baseline characteristics, severity of infection and vasopressors) from medical records of >=15-year-old patients with sepsis and 1st ScvO2 measurement within 24 hours of sepsis, who were admitted in a university hospital between 2013 and 2014. The patients were stratified by 1st ScvO2 level (<70%, 70-80%, >80%) and APACHE-II score (<=25, >25). The primary outcome was in-hospital mortality.

**Results:** Among 376 patients, those with high ScvO2 (17.3%) and low ScvO2 (53.7%) were associated with adjusted hazard ratios for death of 0.79 (0.54-1.15, p=0.218) and 1.16 (0.86-1.56, p=0.325), respectively, while those with normal ScvO2 (29.0%) as control. When the patients were stratified by ScvO2 level and APACHE-II score, using patients with normal ScvO2 and low APACHE-II score as control, those with high ScvO2 and low APACHE-II score, and those with low ScvO2 and low APACHE-II score had adjusted hazard ratios of 0.54 (0.31-0.97, p=0.038) and 1.18 (0.79-1.76, p=0.432). For those with normal, high and low ScvO2, and high APACHE-II score had adjusted hazard ratios of 1.62 (1.02-2.57, p=0.041), 1.77 (1.05-2.96, p=0.031), and 1.88 (1.23-2.87, p=0.004), respectively.

**Conclusions:** The ScvO2 >80% with APACHE-II score >25, but not only ScvO2 >80%, is independently related to increased mortality in patients with sepsis.

## P264 Differences in the impact of lactate levels and ScvO2 on the prognosis of septic patients requiring mechanical ventilation

### Y Kishihara^1^, H Yasuda^2^, S Fujitani^3^, Y Taira^4^, K Morisawa^4^, Y Honma^3^, A Katsumi^1^, S Suzaki^1^

#### ^1^Red Cross Musashino Hospital, Tokyo, Japan; ^2^Kameda medical center, Chiba, Japan; ^3^Tokyo Bay Urayasu Ichikawa Medical Center, Chiba, Japan; ^4^St.marianna University School of Medical, Kanagawa, Japan

**Introduction:** Serum lactic acid levels and ScvO2 are useful predictive parameters for patients with sepsis. However, little is known the differences in the impact of lactate levels and ScvO2 on the prognosis of septic patients. In this study, we investigated these differences by analysing septic patients’ characteristics and prognosis.

**Methods:** This study is a post hoc analysis of data obtained from a multicentre, prospective, randomized controlled trial, which compared two fluid management strategies for septic patients requiring mechanical ventilation. We categorised patients into the following four groups: ScvO2 >= 70% and lactic acid levels < 2 mmol/L (HH group); ScvO2 >= 70% and lactic acid levels < 2 mmol/L (HL group); ScvO2 < 70% and lactic acid levels >= 2 mmol/L (LH group) and ScvO2 < 70% and lactic acid levels < 2 mmol/L (LL group). SOFA score, SAPS II score, lactic acid levels, ScvO2 and BNP were evaluated. Primary outcome was 28-day mortality, whereas secondary outcomes were the duration of mechanical ventilation, administration of CRRT, duration of catecholamine therapy and length of ICU stay.

**Results:** In total, 104 patients were included: HH group (n = 32), HL group (n = 31), LH group (n = 25) and LL group (n = 16). No significant differences were observed in terms of patient characteristics. Further, 28-day mortality was 32% in the LH group, 28.1% in the HH group, 25% in the LL group and 13% in the HL group, and there was no significant difference in terms of mortality among the groups. Furthermore, there were no significant differences in terms of secondary outcomes. On multivariate analysis using the HL group as reference, the odds ratios for 28-day mortality in the LH, HH and LL groups were 1.21 (95%CI, 0.5-5.8), 1.62 (95%CI, 0.36-7.2) and 2.0 (95%CI, 0.37-10.9), respectively.

**Conclusions:** Because 28-day mortality was higher in the HH group than in the LL group, serum lactic acid levels may have bigger impact on the prognosis of septic patients.

## P265 Skin oxygenation, biomarkers of endothelial injury and their association with severity of illness in patients with septic shock

### S Kazune^1^, A Grabovskis^2^, O Suba^3^

#### ^1^Hospital of Traumatology and Orthopaedics, Riga, Latvia; ^2^Institute of Atomic Physics and Spectroscopy, University of Latvia, Riga, Latvia; ^3^Riga East University Hospital, Riga, Latvia

**Introduction:** In septic shock endothelial damage can lead to failure of microcirculation and low microcirculatory oxygen saturation. In the skin this is seen as mottling and can be quantified using hyper spectral imaging. There is insufficient data about associations between skin oxygenation, severity of illness, biomarkers of endothelial damage and mortality in patients with septic shock.

**Methods:** This single centre observational study was performed in 24 consecutive intensive care patients with septic shock. Within 24 hours of admission hyper spectral imaging of knee area skin was performed and blood was sampled for assay of biomarkers of endothelial cell damage (plasminogen activator inhibitor -1 (PAI-1), soluble intercellular adhesion molecule (sICAM-1), soluble vascular cell adhesion molecule (sVCAM-1), thrombomodulin, angiopoetin-2). Nonlinear fitting of optical density spectra was used to calculate relative skin oxy/deoxy hemoglobin concentration and obtain oxygen saturation. The association between skin oxygen saturation, biomarkers, sepsis severity (APACHE II, SOFA) and 28-day mortality was analyzed.

**Results:** The median (IQR) age of patients was 71 years (62 to 76), and 60% were males. The median SOFA and APACHE II scores were 9 (7 to 12) and 24 (19 to 27) and 28-day mortality rate was 29%. 7 patients (37%) had mottling. There was a relationship between skin oxygenation, plasma biomarkers (thrombomodulin and sVCAM-1) and sepsis severity assessed by SOFA and APACHE II scores, P < 0.05. Using logistic regression analysis, skin oxygenation and biomarker concentrations were not associated with 28-day mortality rate.

**Conclusions:** In our cohort of patients with septic shock, skin oxygenation and biomarkers of endothelial injury were strongly associated with initial severity of sepsis but poorly predictive of 28-day mortality.

## P266 Comparison between ultrasound guided technique and digital palpation technique for radial artery cannulation in adult patients: a meta-analysis of randomized controlled trials

### S Maitra, S Bhattacharjee, D Baidya

#### All India Institute of Medical Sciences, New Delhi, New Delhi, India

**Introduction:** Possible advantages and risks associated with ultrasound guided radial artery cannulation in-comparison to digital palpation guided method in adult patients are not fully known. Previous meta-analyses included both adult and pediatric patients and long axis in-plane technique and short axis out of plane technique in the same analysis, which may have incurred biases [1,2].

**Methods:** PubMed and Cochrane Central Register of Controlled Trials (CENTRAL) were searched (from 1946 to 20th November 2017) to identify prospective randomized controlled trials in adult patients where 2-dimensional ultrasound guided radial artery catheterization has been compared with digital palpation guided technique. For continuous variables, a mean difference was computed at the study level, and a weighted standardized mean difference (SMD) was computed in order to pool the results across all studies. For binary outcomes, the pooled odds ratio (OR) with 95% confidence interval (95% CI) was calculated using the inverse variance method.

**Results:** Data of 1895 patients from 10 studies have been included in this meta-analysis. Overall cannulation success rate was similar between short axis out of plane technique and digital palpation [p=0.27; Fig. 1] and long axis in-plane technique with digital palpation. Ultrasound guided long axis in- plane approach and short axis out of plane approach provides better first attempt success rate of radial artery cannulation in comparison to digital palpation [p=0.01 and p=0.0002 respectively; Fig. 2]. No difference was seen in time to cannulate between long axis and short axis technique with palpation technique.

**Conclusions:** USG guided radial artery cannulation may increase the first attempt success rate but not the over all cannulation success when compared to digital palpation technique.

**References**

1. Gu WJ et al. Chest. 2016 ;149:166-79.

2. Tang L et al. PLoS One. 2014;9:e111527.

**Fig. 1 (abstract P266).**
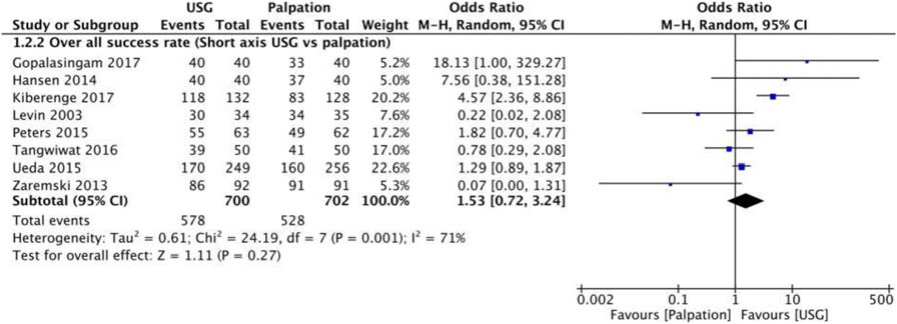
Forest plot showing odds ratio of overall cannulation success rate at study level and pooled analysis level (USG guided short axis out of plane versus digital palpation)

**Fig. 2 (abstract P266).**
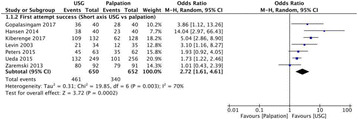
Forest plot showing odds ratio of first attempt cannulation success rate at study level and pooled analysis level (USG guided short axis out of plane versus digital palpation)

## P267 Comparison of real time ultrasound guidance versus palpation technique in radial artery catheterization in critically ill patients presenting with hypotension: a randomized controlled trial

### MS Khan, S Myatra, V Bhagat, S Siddiqui, A Narkhede, N Prabhu, AP Kulkarni, J Divatia

#### Tata Memorial Hospital, Mumbai, India

**Introduction:** Ultrasound guidance may improve the success rate of vascular cannulation. There is lack of data regarding the utility of USG guided arterial cannulation in critically ill patients in shock. We aim to compare the impact of using real time ultrasound guidance versus palpation method in achieving arterial catheterization in critically ill patients in hypotension.

**Methods:** A single center, prospective, randomized trial was performed among 100 critically ill patients aged >18 years, with hypotension (or requiring vasopressor infusion) and on not previous cannulated radial arteries. Patients were randomized in a ratio of 1:1 to the ultrasound group or palpation group. Under aseptic precautions, arterial puncture was performed using appropriate sized Leader Cath (Vygon, Ecquen, France), under real time USG guidance using short-axis out-of-plane view with bevel down. Data were recorded and compared between two groups. The unpaired Student’s t-test or Mann-Whitney U test were used for continuous variables, and the uncorrected Chi-squared or Fisher’s exact test were used for proportions.

**Results:** A total of 100 patients with hypotensive shock requiring radial artery catheterization were randomized into palpation (n = 51) and ultrasound (n = 49) groups. First pass success rate was significantly higher in ultrasound group as compared to palpation group (83% vs 41%, p<0.0001). Cannulation time was significantly shorter in ultrasound group (72.9 vs 88.7,p<0.05). Early complications were significantly higher in palpation group compared to ultrasound group (14.6% vs 5.2%, p<0.001).

**Conclusions:** In critically ill patients with hypotension (or requiring vasopressors), ultrasound guidance improved first pass success rate, shortened the cannulation time and reduced the rate of early complications in radial artery catheterizations.

## P268 Relationship between inferior vena cava diameter and variability with mean arterial pressure and respiratory effort

### B Kalin, K Inci, G Gursel

#### Gazi University School of Medicine, Ankara, Turkey

**Introduction:** There is no consensus on the use of vena cava inferior (IVC) diameter and variability in the assessment of fluid response (FR) in spontaneously breathing ICU patients. Influence from respiratory effort, experience requirement and measurement problems are reasons for not being preferred. The aim of the study is to investigate the relationship between IVC diameter, variability and spontaneous breathing effort and hypotension measured by ultrasonography in spontaneously breathing intensive care patients

**Methods:** The maximum and minimum diameters of the IVC were measured and the collapsibility index (CI) was calculated. Measurements were made in 2D mode on cineloop recordings. Diaphragm thickening ratio was used as a measure of respiratory effort. Correlations between respiratory effort criteria with IVC minimum diameter and CI were calculated by Pearson’s correlation coefficient. IVC measurement criterias, such as inspiratory diameter of < 1 cm, 25%, 40%, 45% of the CI were compared with Chi square test in hypotensive and non-hypotensive patients. We took two mean arterial pressure threshold for hypotension as 60 and 70 mmHg for this calculation.

**Results:** 62 patients were included in the study. For both hypotensive threshold values, there was no significant difference in the rates of hypotensive and non-hypotensive patients with and without a minimum IVC diameter of 1 cm below. Even there was no significant relationship between the CI higher than 25%, 40% and 50% and hypotension (p>0.05). In spontaneously breathing patients, a significant correlation was found between respiratory effort and IVC CI and IVC diameter < 1 cm

**Conclusions:** At the end of the study, there was a correlation between spontaneous breathing effort IVC diameter and CI in the intubed patients. Additionally the result that IVC CI is not different even between hypotensive and non-hypotensive patients suggests that this method should be used with caution in predicting FR.

## P269 Assessing the response to a fluid challenge with LiDCOplus: does it augment decision making?

### J Patterson, C McCue, A Puxty, M Hughes

#### Glasgow Royal Infirmary, Glasgow, UK

**Introduction:** Fluid responsiveness in ICU patients can be assessed using changes in pulse rate and blood pressure following administration of a fluid bolus, assisted if necessary by cardiac output (CO) monitors such as the LiDCOplus. This uses pulse contour analysis to estimate stroke volume (SV), with >10% change in SV following a fluid challenge (FC) signifying overall benefit. There is no evidence that the use of CO monitoring improves patient outcomes and it is unclear if it improves clinical decision making.

**Methods:** A LiDCOplus monitor was set up with the screen covered. A 250ml FC was administered over 2 minutes. The heart rate, systolic and mean arterial pressures were recorded before and after the FC. The clinician administering the FC was asked to decide if the patient was fluid responsive. Following this decision, the SV change was revealed and the clinician asked again to assess fluid responsiveness.

**Results:** Forty-five fluid challenges were studied. Use of the LiDCO changed the decision made on 7 occasions (Fig. 1). In three patients (7%), this change in decision was appropriate and either corrected a misinterpretation of the haemodynamic data or represented a patient whose only marker of fluid responsiveness was a SV change. In four patients (9%), the LiDCO changed the decision inappropriately from a correct interpretation of the haemodynamic data. In six patients (13%) the SV change was ignored when it should have changed the initial decision. In the remaining 32 patients (71%) the decision made with the haemodynamic data was in agreement with the SV change and unchanged by revealing the LiDCO data.

**Conclusions:** The use of LiDCO monitoring only appropriately changed the decision made with information from basic haemodynamic monitoring in 7% of patients. This augmentation of decision making was only seen in patients whose basic haemodynamic parameters did not respond to fluid. It changed a correct decision inappropriately in 9%. Overall, no improvement in the assessment of fluid responsiveness was seen.

**Fig. 1 (abstract P269).**
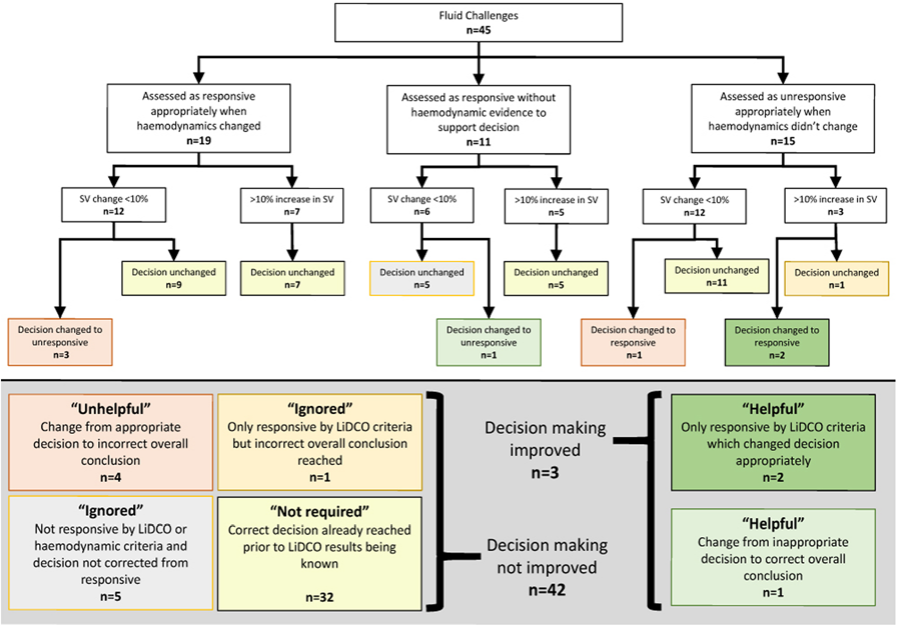
Summary of Fluid Responsiveness Decisions and Effect of LiDCOplus

## P270 Continuous monitoring of blood pressure surge during laryngeal microsurgery using non-invasive pulse transit time

### YS Park, SH Kim, SW Ku, GS Hwang

#### Asan Medical Center, Seoul, South Korea

**Introduction:** There are accumulating evidences suggesting that intraoperative blood pressure affects postoperative outcome including myocardial injury, acute kidney injury, stroke, and mortality. In a patient undergoing laryngeal microsurgery (LMS), blood pressure usually rises sharply due to the stimulation on the larynx. Since pulse transit time (PTT) has been reported to reflect arterial blood pressure fairly well, it has possibility to be a marker for blood pressure which reflects beat-to-beat changes in blood pressure and is less invasive than arterial catheterization.

**Methods:** Intraoperative noninvasive blood pressure (NIBP), electrocardiogram (ECG), and photoplethysmogram (PPG) of 26 patients undergoing LMS were recorded simultaneously. PTT was defined as a time interval between the R-wave peak on ECG and the point which the maximal rising slope appears on the PPG. The mean PTT values for one minute before and after the increase in blood pressure due to the stimulation on larynx were compared. Parameters of PPG such as width, height, maximal slope, minimal slope, and area were also compared. Then, correlation between blood pressure and each variable was calculated.

**Results:** As the larynx was stimulated by LMS, NIBPs have surged (systolic blood pressure, 113.6 ± 22.2 mmHg to 160.5 ± 24.4 mmHg, P < 0.001) and PTTs have decreased (444.8 [423.3;493.7] ms to 385.4 [357.4;419.7] ms, P<0.001) significantly in most of the patients. Systolic blood pressure and PTT were inversely correlated (r = -0.636, P < 0.001). Minimum slope of PPG also showed good negative correlation with systolic blood pressure (r = -0.537, P < 0.001).

**Conclusions:** PPT showed good correlation with systolic blood pressure and may have potential to be used as noninvasive continuous blood pressure monitor during a surgery in which blood pressure changes abruptly.

**Table 1 (abstract P270).** Correlation between systolic blood pressure and PPG related variables

	r	P
Pulse transit time	-0.636	<0.001
PPG amplitude	0.164	0.287
PPG maximum slope	0.249	0.103
PPG minimum slope	-0.537	<0.001
PPG width50	-0.065	0.675
PPG area	0.205	0.182

## P271 Restrictive inter operative volume coupled with lung ultrasound in prevention of post operative cardiogenic pulmonary edema development at cardiac patients who undergone non cardiac surgical procedure-pilot study

### M Karaman Ilic^1^, M Draguljic Svagusa^2^, I Leko^2^, K Slunjski^2^, I Kozul^2^, L Nikles^2^

#### ^1^Clinical Hospital Sveti Duh, Faculty of Medicine JJ Strossmayer University Osijek, Zagreb, Croatia,^2^ Clinical Hospital Sveti Duh, Anesthesiology and ICU, Zagreb, Croatia

**Introduction:** Aim of this prospective randomized pilot study was to investigate influence of intra operative restrictive volume approach and post operative lung ultrasound (LUS)on prevention and early detection of postoperative interstitial syndrome development

**Methods:** 42 cardiac patients who underwent non cardiac surgical procedure were randomly assigned for: group A-liberal volume approach or for group B-combination of restrictive intra operative volume approach and small dose of norepinephrine. All patients post operatively received <=1.5 ml/kg/h fluids, mostly crystalloids. LUS was performed before surgical procedure and 24 hours after their admission in ICU together with arterial blood gases measurements. The ultrasound characteristic of interstitial syndrome was development of B profile

**Results:** Before surgery all patients had A profile. Twenty for hours later in A group significantly higher number of patients 16/22 (72.7%) vs 3/22(13.6%) in B group,had B profile (p<0.05).At the same time there were no significant difference between the groups in amount of patients with PaO2/FiO2 ratio <= 270 (3 patients with positive B lines from A group vs 0 patients from group B).(p>0.05)

**Conclusions:** Intra operative fluid restriction is efficient in prevention of post operative cardiogenic pulmonary edema development. LUS is a simple non invasive method for early detection of interstitial syndrome even before development of signs of respiratory deterioration.

## P272 Performance comparison of ventricular and peripheral dP/dtmax for the evaluation of the left ventricular systolic function

### Monge Garcia^1^, Z Jian^2^, F Hatib^2^, C Hunley^3^, M Cecconi^4^, MR Pinsky^5^

#### ^1^Hospital SAS de Jerez, Jerez de la Frontera, Spain,^2^Edwards Lifesciences, Irvine, California, USA,^3^Orlando Health, Orlando, Florida, USA,^4^St. George’s Healthcare NHS Trust and St George’s University of London, London, UK,^5^University of Pittsburgh School of Medicine, Pittsburgh, Pennsylvania, USA

**Introduction:** The peak rate of left ventricular (LV) pressure (dP/dtmax) has been classically used as a marker of LV systolic function. Since measuring LV dP/dtmax requires LV catheterization, other surrogates have been proposed using the peripheral arterial waveform. The aim of this study was to test the performance of LV and arterial (aortic and femoral) dP/dtmax for assessing LV systolic function against the gold-standard (the slope of the end-systolic pressure-volume relationship, Emax) during different cardiac loading and contractile conditions.

**Methods:** Experimental study in 6 pigs. LV pressure-volume data was obtained with a conductance catheter and peripheral pressures were measured via a fluid-filled catheter into the aortic, femoral, and radial arteries. Emax was calculated during a transient occlusion of the inferior vena cava. The experimental protocol consisted in three consecutive stages with two opposite interventions each: changes in afterload (phenylephrine and nitroprusside), preload (bleeding and fluid bolus), and contractility (esmolol and dobutamine) (Fig. 1). Measurements were obtained before and after each hemodynamic intervention.

**Results:** Emax variations and LV, aortic, femoral and radial dP/dtmax changes throughout the study are shown in Fig. 2. All peripheral artery–derived dP/dtmax underestimated LV dP/dtmax. Percentage changes in LV and femoral ddP/dtmax were tightly correlated (r^2^=0.77; P<0.02). Both LV and femoral dP/dtmax were affected by preload changes during fluid infusion. All peripheral dP/dtmax estimations allow to detect LV systolic function changes according to Emax during isolated variations in contractility.

**Conclusions:** Femoral and LV dP/dtmax accurately reflected Emax changes, although both were affected by preload changes during fluid administration.

**Fig. 1 (abstract P272).**
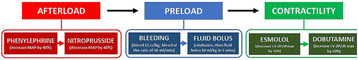
Experimental protocol.

**Fig. 2 (abstract P272).**
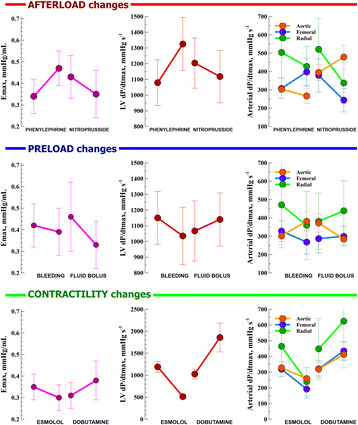
Emax, LV dP/dtmax and aortic, femoral and radial dP/dtmax changes.

## P273 Perioperative hemodynamic monitoring in cytoreductive surgery with hyperthermic intraperitoneal chemotherapy

### A Heijne, P Krijtenburg, LA Baggen, MA Swenne, C Slagt

#### Radboudumc, Nijmegen, Netherlands

**Introduction:** We conducted a study to validate three different cardiac output measurement devices in patients undergoing extensive abdominal surgery. In contrast to the pulmonary artery catheter, alternative measurement devices were introduced with different degrees of invasiveness.[1] The will evaluate the accuracy of the ClearSight (CS), the FloTrac (FT) and the ProAQT (PA) derived cardiac index (CI) compared to transpulmonary thermodilution (TPTD) derived CI (CITD) during the extensive abdominal surgery and hyperthermic intraperitoneal chemotherapy (HIPEC).

**Methods:** In this prospective observational study we included 25 adults between March 2016 and February 2017. On predefined moments during and after surgery we performed serial paired CI measurements in CS, FT, PA and TPTD. All devices were used according to the manual. CS, FT and PA were not calibrated against TPTD. Agreement was assessed with Bland-Altman plots. The repeatability coefficient (RC), corrected limits of agreement (LOA), bias and percentage errors (PE) were calculated [2]. Trending was assessed with polar plots and concordance analysis [3].

**Results:** Mean CI were 3.2, 3.1, 3.2 and 3.3 mL∙min^−1^∙m^−2^ (CS, FT, PA, TD). RC was comparable between CS, FT, PA and TD (RC of 0.5, 0.5, 0.6 and 0.1 mL∙min^−1^∙m^−2^). Bias was -0.1, -0.3, -0.2 mL∙min^−1^∙m^−2^ and LOA were ±1.6 (PE 50%), ±1.6 (PE 51%), ±1.7 (PE 53%) vs. ±1.1 (8%) mL∙min^−1^∙m^−2^ (CS, FT, PA, TD (ref vs. ref.)), which were only partially explained by the lack of repeatability (Table 1, Fig. 1). Concordance was <95% and radial LOA was ±<30° for all devices; mean polar bias was <5° for FT only (table 2, Fig. 2).

**Conclusions:** CS, FT and PA are not interchangeable with TPTD, because of inaccuracy [2]. When considering limitations they may be used for trending.

**References**

1 Slagt C et al. Crit Care 14:208, 2010

2 Cecconi M et al. Crit Care 13:201, 2009

3 Critchley LA et al. J Cardiothorac Vasc Anesth 25:536-46, 2011

**Table 1 (abstract P273).** Analysis of agreement using Bland-Altman statistics. Test devices vs. TD and TD vs. TD (reference vs. reference)

	CS	FT	PA	TD
Mean (mL min^−1^∙m^−2^)	3.2	3.1	3.2	3.3
Bias (95%-CI, mL∙min^−1^∙m^−2^)	-0.1 (-0.5 – 0.1)	-0.3 (-0.6 – -0.1)	-0.2 (-0.5 – -0.1)	0 (0 – 0)
LOA (95%-CI, mL∙min^−1^∙m^−2^)	±1.6 (1.5 – 1.8)	±1.6 (1.5 – 1.8)	±1.7 (1.6 – 1.9)	±1.1 (1.0 – 1.3)
PE (95%-CI, %)	50 (45 – 55)	51 (46 – 56)	53 (48 – 59)	8 (8 – 9)
RC (95%-CI, mL∙min^−1^∙m^−2^)	0.5 (0.5 – 0.6)	0.6 (0.5 – 0.6)	0.6 (0.6 – 0.7)	0.1 (0.1 – 0.2)

**Table 2 (abstract P273).** Analysis of trending. Concordance analysis suggests inability to track changes in CI, while polar plot analysis shows adequate ability considering systematic error in CS and PA

	CS	FT	PA
Angular bias	27°	2°	11°
Radial LOA	±24°	±28°	±19°
Concordance	90%	64%	82%

**Fig. 1 (abstract P273).**
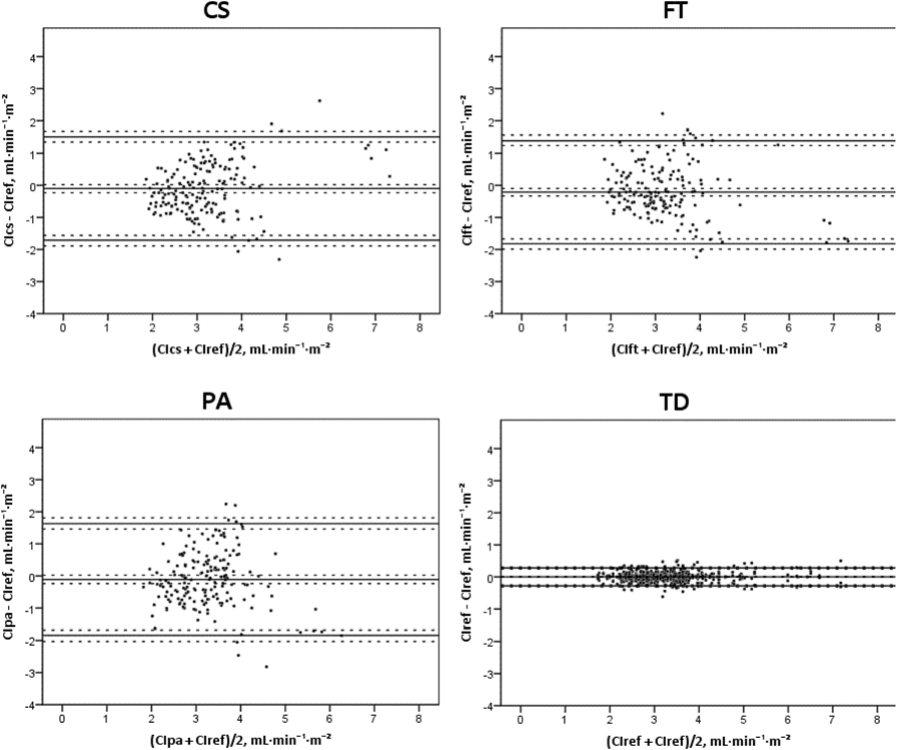
Bland-Altman plots of the three test devices and TD vs. TD. Data of 25 patients at up to nine points in time are plotted. Solid lines indicate bias and limits of agreement, dotted lines indicate 95%-CI of each solid line. These plots indicate similar performance of CS, FT and PA vs. TD

**Fig. 2 (abstract P273).**
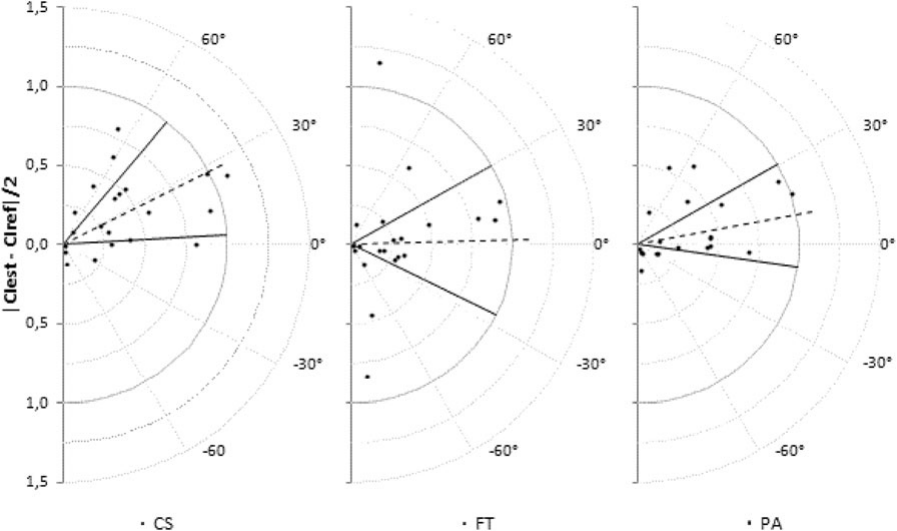
Polar plots showing trending capability of CS, FT, PA vs. TD between T4 (start HIPEC) and T6 (end of HIPEC). Exclusion zone is 14% (least significant change in CITD). All devices show satisfactory radial LOA (±<30°), but only FT shows acceptable radial bias (2°)

## P274 Comparison of cardiac index (CI) estimates by body surface temperatures combined with biometric data (CI_CIN), uncalibrated clearsight and flotrac vs. transpulmonary thermodilution derived CI_TD

### W Huber, J Mangold, T Lahmer, A Herner, U Mayr, R Schmid, M Heilmaier

#### Klinikum rechts der Isar, Munich, Germany

**Introduction:** Body surface temperature BST provide a rough estimate of cardiac index CI. Non-contact infrared thermometers (Thermofocus; Tecnimed) more accurately measure BST than clinical examination. We hypothesized that accurate measurement of BST combined with biometric data might provide a complete non-invasive estimate of CI (CI_CNI) with similar accuracy as the uncalibrated devices ClearSight (CI_CS) and FloTrac (CI_FT; both Edwards Lifesciences). Therefore, we compared CI_CNI to CI_CS, CI_FT and to the gold-standard CI_TD derived from transpulmonary thermodilution (TPTD; PiCCO).

**Methods:** In 22 patients (APACHE-II 28+-6) a total of 176 datasets were recorded (8 datasets per patient within 24h; study period 7/2017-11/2017). Immediately before TPTD we measured BST on the forehead, forearm (middle and distal), finger and great toe and recorded un-calibrated CI_CS and CI_FT. These data were compared to TPTD-derived CI-TD.

Statistics: IBM SPSS 24.

**Results:** Multiple regression analysis (R2=0.572) including BSTs and biometric data demonstrated independent association of CI_TD with BST_forehead (p<0.001), BST_middle_forearm (p=0.001), young age (p<0.001), male gender (p<0.001), height (p<0.001) and low weight p=0.021).This regression formula resulted in CI-CNI.

Bias and percentage error compared to CI_TD were -0.04 L/min/m2 and 49% for CI_CNI, -0.10 L/min/m2 and 65% for CI_FT and -0.85L/min/m2and 60% for CI_CS, respectively.

The ROC-AUCs regarding CI_TD>=5 L/min/m2and CI<=.5 were 0.92 and 0.79 for CI_CNI (both p<0.001), 0.79 and 0.63 (p<0.001 an p=0.6) for CI_FT, and 0.89 (p<0.001) and 0.68 (p=0.08) for CI_CS.

**Conclusions:** BSTs combined with biometrics provide an estimate of CI (CI_CIN) which is least comparable to CI_CS and CI_FT.

## P275 Features of oxygen extraction ratio and temperature homeostasis during early postoperative period after major abdominal surgery

### I Zabolotskikh, T Musaeva

#### Kuban State Medical University, Krasnodar, Russia

**Introduction:** About 100 years ago, the German physiologist Pflüger stated that the cardio-respiratory system fulfils its physiological task by guaranteeing cellular oxygen supply and removing waste products of cellular metabolism.

**Methods:** The study was performed in early postoperative period after major abdominal surgery in 160 patients. The physical condition of patients corresponded to 3 class of ASA. The median age was 46.0 (38.0- 62.0) years. Duration of the surgery was 6,4 (4,8‐9,5) hours. Surgery was performed under combined epidural anesthesia with mechanical ventilation. The study was conducted in the following stages: 1- admission from operating room; 2 - in 1-3 hours; 3 - 4-7 hours; 4 - 8-12 hours; 5 - after 13-24 hours after the surgery.

**Results:** Depend on rate of oxygen extraction index (ERO2) 4 groups were revealed: group 1 (n=44) ‐ low ERO2 (<21%) followed by recovery to normal levels to stage 2-3 (ERO2 = 22-32%), group 2 (n=42) ‐ normal level ERO2 (2232%) in all the stages, group 3 (n=40) ‐ high levels ERO2 (>33%) with recovery to normal levels to stage 2, group 4 (n=34) ‐ high ERO2 (>35%) in all the stages. Oxygen extraction index at admission to ICU after surgery can be normal (26.25% of patients), reduced (27.5% of patients) or high (46.25% of patients). When oxygen extraction ratio is reduced metabolic recovery occurs classically after 4-7 hours; when ERO2 is elevated - after 812 hours. Core temperature improvement is connected with the restoration of oxygen homeostasis. So, under normal and reduced ERO2 even mild central hypothermia after surgery were not observed, and at an elevated ERO2 moderate hypothermia after surgery was observed with only to 4-7 hours post-surgery restoration.

**Conclusions:** Maintaining an adequate tissue oxygenation is the cornerstone of metabolic response and postoperative recovery in patient after major abdominal surgery.

## P276 Relation between venous-arterial CO2 to arterial-venous O2 content difference ratio and renal resistive index in critically ill patients

### G Fotopoulou, I Poularas, S Kokkoris, I Broutzos, A Baladima, C Routsi

#### National and Kapodistrian University of Athens, Athens, Greece

**Introduction:** The ratio between venous-arterial PCO2 difference and arterial-venous O2 content difference (Pv-aCO2/Ca-vO2) has been recognized as an alternative marker of global anaerobic metabolism. Renal resistive index (RRI) has recently been proposed to detect renal tissue hypoxia and consequent acute kidney injury. The main objectives of our study were to evaluate whether the Pv-aCO2/Ca-vO2 ratio and RRI are associated and their implications to mortality in ICU patients.

**Methods:** Prospective observational study, including mechanically ventilated patients except those with pre-existing chronic kidney injury. Clinical and laboratory data, SOFA score Pv-aCO2/Ca-vO2 ratio and RRI measurements were obtained within the first 24 hours of ICU admission.

**Results:** A total of 168 patients (median age 62 years, 58% males, SOFA score 9±1) were included. Shock was present in 53.6% of them. Median (IQR) Pv-aCO2/Ca-vO2 ratio and RRI values were 1.54 (1-2.58) and 0.77 (0.70-0.83) respectively. There was a statistically significant correlation between Pv-aCO2/Ca-vO2 ratio and the presence of shock (p=0.017) as well as with the SOFA score (p=0.037). A statistically significant correlation was also observed between RRI, Pv-aCO2/Ca-vO2 ratio and ICU survival (p<0.001). High RRI (mean 0.80) and high Pv-aCO2/Ca-vO2 ratio (mean 1.97) were linked to an increased risk of ICU mortality. Stratified analysis by lactate level revealed statistically significant correlation between Pv-aCO2/Ca-vO2 ratio and RRI (p<0.01). Pv-aCO2/Ca-vO2 ratio and RRI were positively correlated for lactate value >=2 (rho=0.343).

**Conclusions:** Pv-aCO2/Ca-vO2 correlates positively with RRI, indicating tissue hypoxia. Consequently, their combination could serve as a complementary tool for clinical outcome assessment and for a better management of critically ill patients, especially those in shock states.

## P277 Cerebral oxygen saturation measurement in patients on venoarterial extracorporeal membrane oxygenation – a potential therapeutic target

### S Zoufaly, G Trummer, C Benk, T Wengenmayer, C Bode, DL Staudacher

#### Heart Center Freiburg University, Freiburg i. Br., Germany

**Introduction:** Monitoring of cardiac output and sufficient oxygen tissue supply in patients undergoing venoarterial extracorporeal membrane oxygenation (vaECMO) can be challenging. Cerebral oxygen saturation measurement (cSO2) via near infrared spectroscopy (NIRS) offers a promising tool, however has been validated only for on pump cardiac surgery. Goal of the present study was to assess the prognostic value of NIRS in patients undergoing vaECMO on the intensive care unit.

**Methods:** This retrospective registry analysis includes all patients (age > 18 years) with vaECMO and NIRS monitoring treated at a single center between 01/2015 and 10/2017 on two independent intensive care units. Two NIRS sensors were routinely placed on the forehead of each vaECMO patient. We evaluated average cSO2 over the whole course of therapy. The area under cSO2<50% was defined as the cSO2<50%time and was calculated by subtracting the actual cSO2 from the predefined threshold of 50% multiplied by the time [hours] of the desaturation.

**Results:** This study consists of 69 vaECMO patients (56 in cardiac shock and 13 after cardiac surgery). The patients were 58.8±13.6 years old and 26 (38.3%) survived with good neurology (Cerebral Performance Categories Scale 1 or 2) to hospital discharge. Average cSO2 was statistically similar in survivors and non-survivors (63±1% vs. 59±2%, p=0.09). The cSO2<50%time was significantly lower in survivors (20±1%h vs. 97±3%h p=0.04) (Fig. 1). Patients with cSO2<50%time above 50%h had an odds ratio of hospital survival of 0.19 (95%CI 0.38-0.91, p=0.037) (Fig. 2).

**Conclusions:** Cerebral oxygen desaturation below 50% was significantly associated with outcome in patients undergoing vaECMO. In patients with cSO2<50%time above 50h%, prognosis was especially poor. Prospective trials are needed to evaluate if cSO2 is a viable target for therapeutic interventions.

**Fig. 1 (abstract P277).**
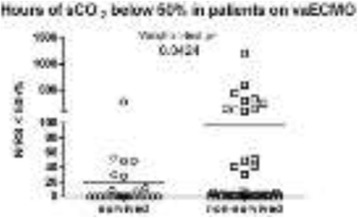
The area under cSO2<50% was significantly lower in survivors

**Fig. 2 (abstract P277).**
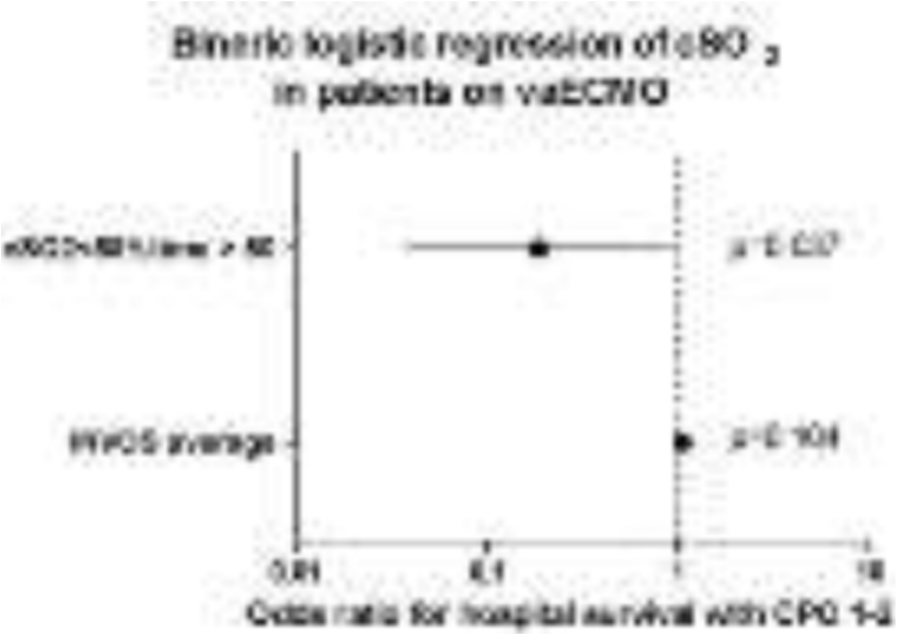
Prognosis of patients with cSO2<50%time above 50%h was poor

## P278 A software tool to quantify capillary hematocrit and microvascular hemodilution in sublingual incident dark field microscopy video clips

### S Arend^1^, C Ince^1^, M Van Assen^1^, T Fevzi^2^, MP Hilty^1^

#### ^1^Academic Medical Center, Amsterdam, Netherlands,^2^Acıbadem Mehmet Ali Aydınlar University School of Medicine, Istanbul, Turkey

**Introduction:** During the second consensus meeting on microcirculatory analysis the exploration of novel parameters related to physiological function of the microcirculation was proposed. Capillary hematocrit (cHct) is a direct measure of capillary hemodilution, a potential mechanism of microcirculatory dysfunction in states of shock. Our hypothesis was that by application of advanced computer vision (I) cHct can be reliably measured in given capillaries, and (II) change in cHct reflects capillary hemodilution induced by cardiopulmonary bypass (CPB).

**Methods:** In 11 patients undergoing coronary artery bypass surgery 3 sublingual capillary microscopy videos were recorded before and during CPB primed with HES 130/0.4. Per-capillary cHct was estimated as the product of the number of red blood cells (RBC) and an assumed volume of 90nl, divided by the capillary volume including plasma gaps. RBC number was assessed by manual counting in the first frame of a given video clip, as well as using a novel advanced computer vision algorithm employing blob detection to calculate the mean per-capillary RBC number in all frames of a given video clip (Fig. 1).

**Results:** 100 capillaries were analyzed, within a total of 100 and 322000 frames using manual and algorithmic analysis. A good correlation was found between both methods for cHct (r=0.79, p<0.01, Fig. 2). CPB initiation resulted in an decrease in cHct from (mean±SEM) 0.23±0.02 to 0.18±0.01, p<0.001 and 0.23±0.02 to 0.18±0.01, p=0.05 in manual and algorithm.

**Conclusions:** Accurate measurement of cHct is possible using advanced computer vision, and it reflects hemodilution induced by initiation of CPB. cHct further is a determinant of capillary delivery of oxygen. Combined with the assessment of functional capillary volume, blood flow velocity, and capillary hemoglobin saturation, cHct may enable direct optical quantification of capillary delivery of oxygen as an integrated functional parameter of the microcirculation.

**Fig. 1 (abstract P278).**
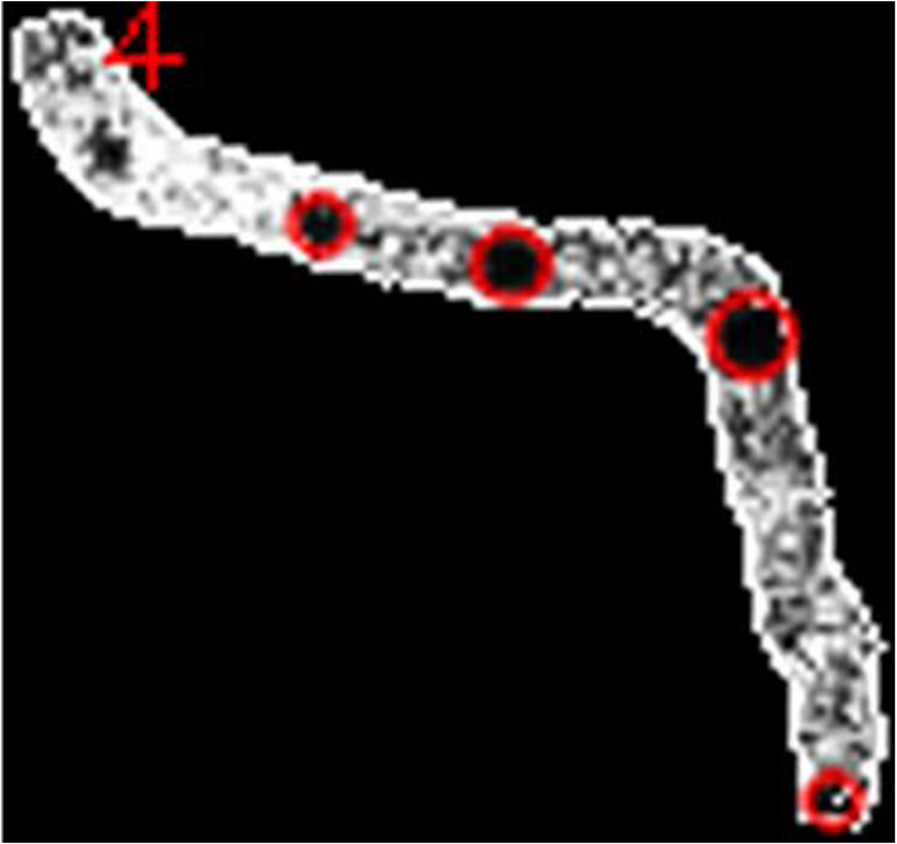
Detection of single erythrocytes using a novel advanced computer vision algorithm in a representative capillary ribbon extracted from a video frame of the sublingual microcirculation

**Fig. 2 (abstract P278).**
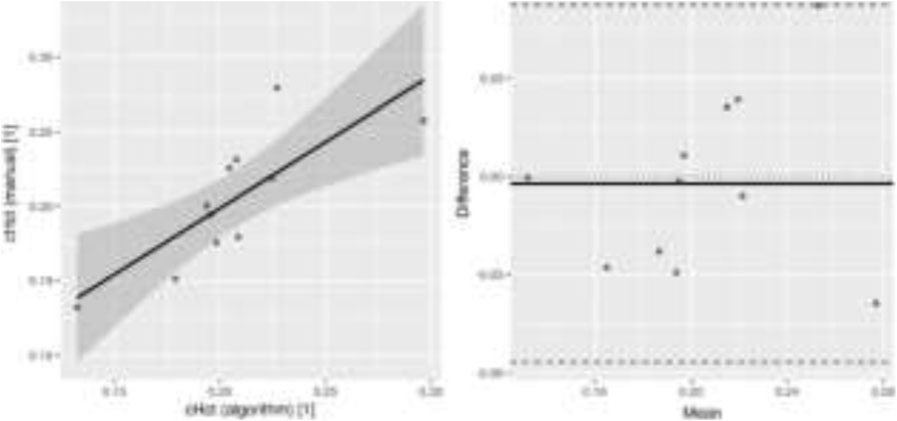
Correlation and Bland-Altman analysis of per patient capillary hematocrit (cHct) as measured manually and using a novel advanced computer vision algorithm

## P279 Cardiac preload expansion and cardiac output as related to patient outcome

### I Douglas^1^, P Alapat^2^, K Corl^3^, L Forni^4^, A Holder^5^, D Kaufman^6^, M Levy^3^, G Martin^5^, J Sahatjian^7^, E Seeley^8^, W Self^9^, NI Shapiro^10^, A Wolff^11^, D Hansell^12^

#### ^1^Denver Health Medical Center, Denver, Colorado, USA,^2^Ben Taub, Houston, Texas, USA,^3^Rhode Island Hospital, Providence, Rhode Island, USA,^4^Royal Surrey Hospital, Guilford, UK,^5^Grady Memorial Hospital, Atlanta, Georgia, USA,^6^New York Langone Medical Center, New York, New York, USA,^7^Cheetah Medical, Newton Center, Massachusetts, USA,^8^Univesity of California San Francisco, San Francisco, California, USA,^9^Vanderbilt, Nashville, Tennessee, USA,^10^Beth Israel Medical Center, Boston, Massachusetts, USA,^11^Bridgeport Hospital, Bridgeport, Connecticut, USA,^12^Massachusetts General Hospital, Boston, Massachusetts, USA

**Introduction:** Cardiac function is known to be impacted by sepsis. Passive Leg Raise (PLR) is an effective method to predict fluid responsiveness (FR) or cardiac response to preload expansion. Preload functional status and trending cardiac output may identify patient phenotypes with varying cardiac reserve, dysfunction and outcome.

**Methods:** Patient data were analyzed from a currently enrolling prospective randomized controlled study, evaluating the incidence of FR in critically ill patients with sepsis or septic shock (FRESH study, NCT02837731). Patients randomized to PLR guided resuscitation were classified as PLR+ (fluid responsive/preload dependent) if stroke volume (SV) increased >= 10% when measured with a non-invasive bioreactance device (Starling SV, Cheetah Medical). Patients were categorized into 5 different phenotypic cohorts based on changing physiology exhibited on PLR and trending cardiac output over the initial 72 hours of therapy.

**Results:** A total of 269 PLR assessments were performed in 31 patients. Overall, 36% (96/269) of assessments indicated a patient was PLR+ after receiving initial resuscitation fluid of ~ 3L. Most patients (71%) demonstrated a dynamic physiology with changing PLR Status occurring > 1 time over 72 hours. There were no differences among the 5 groups with respect to age, gender, or QSOFA score (Fig. 1). Patients in Group 1 exhibited a significantly decreased ICU stay (113.8 hours) compared to Group 3 (271.1 hours, p=0.024) (Fig. 2). Patients in Group 3 exhibited significantly increased ECHO evidence of LV/RV cardiac dysfunction (77%), compared to Group 1 (16%, p=0.02) (Table 1). Patients in Group 4 exhibited 100% evidence of ECHO based LV/RV cardiac dysfunction.

**Conclusions:** Physiological based resuscitation phenotypes identify significantly different patient groups. Patients who are initially not PLR+, but then become PLR+ with no improved CO are significantly more likely to have confirmed LV/RV dysfunction and a significantly longer ICU stay.

**Table 1 (abstract P279).** ECHO Documented LV/RV Dysfunction

SV	ECHO LV/RV Dysfunction	-	+
Grp 3	-	2	7
Grp 1	+	5	1
Chi Square	P	0.02	

**Fig. 1 (abstract P279).**
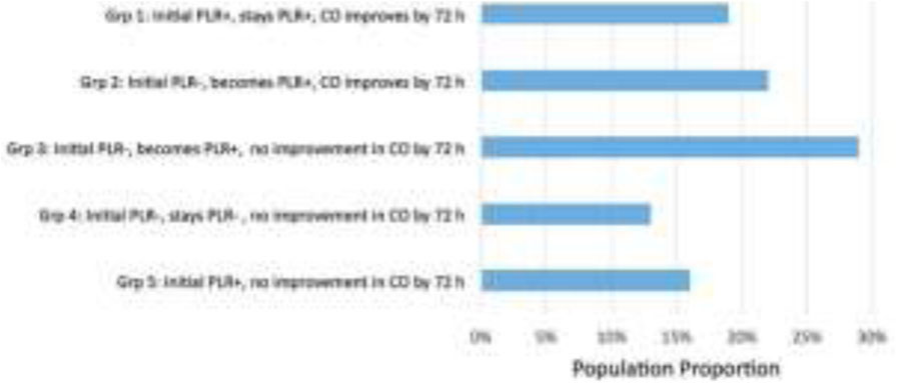
Physiologic Phenotypes Based on PLR and Trending Cardiac Output

**Fig. 2 (abstract P279).**
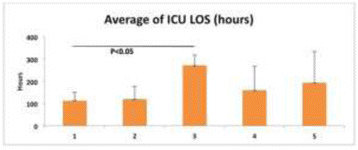
Average ICU LOS

## P280 Clinical measurement of plasma (PV) and blood volume (BV) at the bedside

### BA Molitoris^1^, DJ Meier^2^, ES Riley^2^, RM Sandoval^1^, AD Shaw^3^, DV Rizk^4^, JP Muldoon^2^, JS Strickland^2^

#### ^1^Indiana University School of Medicine, Indianapolis, Indiana, USA,^2^FAST Biomedical, Indianapolis, Indiana, USA,^3^Vanderbilt University Medical Center, Nashville, Tennessee, USA,^4^University of Alabama School of Medicine, Birmingham, Alabama, USA

**Introduction:** Accurate measurement of a patient’s intravascular volume status remains an unsolved clinical problem in the ICU setting. In particular, septic and cardio-renal patients often receive volume challenges or diuresis, respectively, with little appreciation of baseline BV or the resulting response. This can lead to volume overload and/or depletion and associated increases in morbidity, mortality and hospital length of stay.

**Methods:** We tested the performance of a novel, rapid, minimally invasive technique capable of measuring PV, BV and glomerular filtration rate (mGFR) in 32 human subjects. The method consists of a single IV injection of a large (150 kDa) carboxymethyl dextran conjugated to a rhodamine-derived dye and a small (5 kDa) carboxymethyl dextran conjugated to fluorescein. Plasma and blood volumes were quantified 15 minutes following the injection of the dye based on the indicator-dilution principle.

**Results:** This phase 2b study included 16 normal subjects, 8 chronic kidney disease (CKD) stage III and 8 CKD stage IV subjects. PV and BV varied according to weight and body surface area, with PV ranging from 2115 to 6234 mls, and both were stable for greater than six hours with repeated measurements. There was excellent agreement (Fig. 1) with Nadler’s formula for PV in normal subjects. A 24 hour repeat dose measurement in 8 healthy subjects showed PV variability of less than +/- 5%. Following an intravenous bolus of 350 ml 5% albumin solution the mean +/-(SD) measured increase in PV was 326.8 ml +/- 49.9 ml post infusion (Fig. 2).

**Conclusions:** This novel bedside approach allowed for rapid and accurate determination of PV, BV, mGFR (data not shown) and dynamic monitoring following clinical maneuvers such as fluid administration, with a high level of safety, accuracy and reproducibility. This approach should assist the Intensivist especially with volume administration and removal in septic and cardiorenal patients.

**Fig. 1 (abstract P280).**
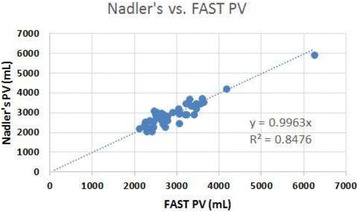
There was excellent agreement with Nadler’s formula for PV in normal subjects

**Fig. 2 (abstract P280).**
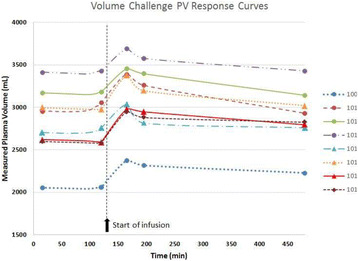
Following an intravenous bolus of 350 ml 5% albumin solution the mean +/-(SD) measured increase in PV was 326.8 ml +/- 49.9 ml post infusion

## P281 Age-dependent response to intravenous fluid restriction in a model of acute lung injury in rats

### SA Ingelse, J Juschten, MA Maas, NP Juffermans, JB Van Woensel, RA Bem

#### Academic Medical Center, Amsterdam, Netherlands

**Introduction:** Accumulating evidence shows that fluid overload is independently associated with adverse outcome in children and adults with acute lung injury. Fluid restriction initiated early in the disease process may prove beneficial, potentially by diminishing the formation of interstitial edema. The main goal of this study was to determine the short-term biophysical effects of intravenous (IV) fluid restriction during acute lung injury in relation to age.

**Methods:** Infant (2-3 weeks) and adult (3-4 months) Wistar rats were mechanically ventilated (MV) 24 hours after intratracheal inoculation with lipopolysaccharide to model acute lung injury. Both age groups were randomized to either a normal or restrictive IV fluid regimen during 6 hours of MV. Thereafter the rats were sacrificed and studied for markers of interstitial edema formation (wet-dry weight ratios), lung permeability (total protein and alpha-2 macroglobulin (A2M) in bronchoalveolar lavage; BAL) and local inflammation (cell counts and cytokines in BAL).

**Results:** Restrictive fluid therapy was not associated with worsening of hemodynamic indices during the period of MV in either infant or adult rats. However, as compared to the normal fluid regimen, restrictive fluid therapy led to lower wet-dry weight ratios of the lungs and kidneys in adult rats (p < 0.05), but not in infants (Figs. 1 and 2). No difference was found in total protein and A2M in BAL between the two fluid regimens in both age groups. Also, neutrophil influx in the lungs did not differ between fluid regimens in both age categories, nor did the influx of inflammatory cytokines IL-6 and MIP-2 in BAL fluid.

**Conclusions:** There is an age-dependent effect of early fluid restriction on the formation of interstitial edema in local and distant organs in the disease process of acute lung injury. Further investigation of the effects of fluid therapies in experimental models may help steering towards better treatment in critically ill patients.

**Fig. 1 (abstract P281).**
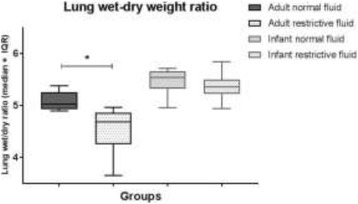
Lung wet-dry weight ratio. There is a significant difference between normal and restrictive fluid regimen in adult rats, however not in infants. Data are presented as median and interquartile range (IQR); whiskers present 1.5 IQR. * p<0.05

**Fig. 2 (abstract P281).**
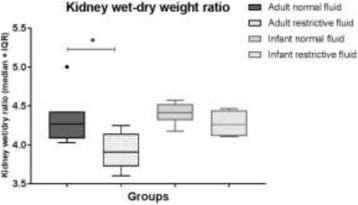
Kidney wet-dry weight ratio. There is a significant difference between normal and restrictive fluid regimen in adult rats, however not in infants. Data are presented as median and interquartile range (IQR); whiskers present 1.5 IQR. * p<0.05

## P282 The effect of a change in organizational measures on (para)medical behavior and adherence to fluid resuscitation protocol

### L Delmas Benito, J Haenen, M Koopmans, P Koetsier, E De Widt, EC Boerma

#### Medical Center Leeuwarden, Leeuwarden, Netherlands

**Introduction:** Fluid therapy remains the cornerstone of shock resuscitation, but recent studies have highlighted the potential dangers of fluid overload, and protocols have been established to apply responsible fluid resuscitation. However, studies show that adherence to protocols by healthcare providers remains a challenge. The present study researched the effect of a change in organizational measures on (para)medical behavior and adherence to fluid resuscitation protocols.

**Methods:** Fluid balances (FB) of post-cardiac surgical patients, 12 hours after ICU admission, were retrospectively evaluated after introduction of two different organizational measures, designed to (unconsciously) influence (para)medical behavior. Patients were divided into three groups: group A received 500ml fluid challenges, group B received 250ml fluid challenges and group C had a continuous FB registration throughout the entire hospitalization.

**Results:** 3 × 250 patients were included in the study. No significant differences were found across demographic features. The FB was significantly lower in group C in comparison to group A and B, (1.6 [0.7-2.6] L versus 2.8 [1.0-3.8] L and 2.8 [1.9-3.8] L respectively; (p<0.001)) (Fig. 1). In a multivariate analysis FB was independently associated with: group C (p<0.001), a history of diabetes (p=0.03), the Acute Physiology and Chronic Health Evaluation III score (<0.001) and the duration of aortic-cross clamp (p<0.001).

**Conclusions:** The main findings of this study substantiated the hypothesis that the introduction of continuous FB-tracking throughout the entire care process, is associated with a significant reduction in the administration of fluids in post-cardiac surgery patients, independent of differences in their baseline characteristics. Demonstrating that certain organizational changes can influence medical behavior beyond the scope of teaching and instruction, and therefore serves to provide awareness to the current issue known as ‘knowledge-to-care gap’.

**Fig. 1 (abstract P282).**
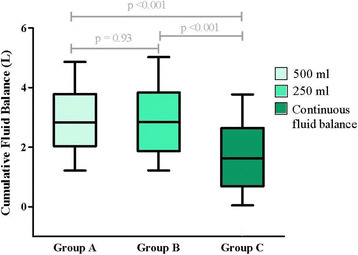
Cumulative fluid balance in the first 12 hours after start of cardiac surgery

## P283 Using a protocol for fluid resuscitation: how well is it followed?

### R Cowan, J Patterson, C McCue, M Hughes, A Puxty

#### Glasgow Royal Infirmary, Glasgow, UK

**Introduction:** Positive fluid balance in ICU patients has been correlated with worse outcomes [1]. Consequently, we developed a protocol to guide fluid resuscitation. The protocol was introduced in 2011 and mandates that fluid responsiveness is assessed when administering fluid boluses. Once a patient becomes fluid unresponsive, no further resuscitation fluid should be administered. To assess responsiveness, the protocol advises the use of haemodynamic data such as heart rate and blood pressure as well as the change in stroke volume (SV) measured by a LiDCOplus monitor. After years of use and a rolling education program this protocol was felt to be well ingrained in our unit culture. We then assessed how well it was being followed.

**Methods:** Staff performing fluid challenges were asked to fill out a form recording the haemodynamic and SV data measured before and after a fluid challenge. They were also asked to record their interpretation of just the haemodynamic data and then this data combined with the SV data.

**Results:** Forty five forms were completed. The protocol was not followed on 16 occasions (36%). Four patients who should have been assessed as responsive were deemed to be unresponsive. Six patients who should have been assessed as unresponsive were assessed as being responsive. The remaining deviations from the protocol represent misinterpretation of the haemodynamic data but correct use of the SV data to reach a correct final assessment.

**Conclusions:** Despite being a longstanding ingrained practice in our ICU, this review suggests that the protocol for fluid resuscitation is being followed incorrectly approximately a third of the time. This could result in inappropriate under or over administration of IV fluid. We plan to review the educational programme and raise awareness of the protocol to try and improve future compliance.

**Reference**

1. Vincent JL et al. Crit Care 19:251, 2015

## P284 A reappraisal of the effects of fluid administration on left ventricular loading conditions in critically ill patients

### M Jozwiak^1^, S Millasseau^2^, C Richard^1^, X Monnet^1^, P Mercado^1^, F Dépret^1^, JE Alphonsine^1^, JL Teboul^1^, D Chemla^3^

#### ^1^Hôpitaux universitaires Paris-Sud, Hôpital de Bicêtre, APHP, service de réanimation médicale; Inserm UMR S_999, Univ Paris-Sud, Le Kremlin-Bicêtre, France, ^2^Pulse Wave Consuting, Saint Leu La Foret, France, ^3^Hôpitaux universitaires Paris-Sud, Hôpital de Bicêtre, APHP, service de physiologie; Inserm UMR S_999, Univ Paris-Sud, Le Kremlin-Bicêtre, France

**Introduction:** Understanding the effects of therapeutics on the left ventricular (LV) loading conditions is of utmost importance in critically ill patients. The effective arterial elastance (Ea=ESP/SV, where ESP is aortic end-systolic pressure and SV stroke volume) is a lumped parameter of arterial load that has been proposed as an index of LV afterload. We aimed at comparing the effects of fluid administration on ESP (i.e., the LV afterload in the pressure-volume phase-plane according to the classic “cardiocentric” framework) and on Ea.

**Methods:** In 30 mechanically ventilated patients, we recorded Ea from the femoral peripheral systolic arterial pressure SAP (Ea=(0.9×femoral SAP)/SV) before and after the infusion of 500-mL of saline. Patients in whom fluid administration induced an increase in cardiac index (PICCO-2) >=15% were defined as “responders”.

**Results:** At baseline Ea (1.92±0.98 mmHg/mL) was positively related to total arterial stiffness TAS (r=0.95), to systemic vascular resistance SVR (r=0.89) and to heart rate HR (r=0.37) (each P<0.05). Fluid administration increased ESP (from 103±20 to 117±23 mmHg) and SV (from 64±26 to 73±24 mL) (each P<0.05). This resulted in a non-significant decrease in Ea (1.79±0.73 mmHg/mL). The changes in Ea were positively related to changes in TAS and SVR (each r=0.88; P<0.05) but not in HR. Most patients (90%) increased their ESP and the concordance rate between changes in ESP and Ea was 57%. In fluid responders, ESP increased and Ea decreased (each P<0.05). In fluid non responders, ESP increased (P<0.05) while Ea remained unchanged.

**Conclusions:** Fluid administration increased LV afterload (ESP) if one relies on the classic “cardiocentric” framework, and this challenged the opposite conclusions supported recently on the basis of the slight decreases in Ea (“arterial load” framework). Ea taken in isolation is not an index of LV afterload in critically ill patients.

## P285 Physiological determinants of the respiratory variations of the inferior vena cava diameter in ventilated critically ill patients

### M Jozwiak, P Mercado, JL Teboul, F Dépret, C Richard, X Monnet

#### Hôpitaux universitaires Paris-Sud, Hôpital de Bicêtre, APHP, service de réanimation médicale; Inserm UMR S_999, Univ Paris-Sud, Le Kremlin-Bicêtre, France

**Introduction:** The respiratory variations of the inferior vena cava (IVC) diameter in mechanically ventilated patients with preload responsiveness could be explain by a higher compliance of the IVC and/or higher respiratory variations of the IVC backward pressure, i.e., the central venous pressure (CVP).We aimed at determining the respective weight of these two phenomena.

**Methods:** In 25 mechanically ventilated patients, haemodynamic, respiratory and the intra-abdominal pressure (IAP) signals were continuously computerised. CVP, IAP and the IVC diameter (transthoracic echocardiography) were recorded during end-inspiratory and end-expiratory occlusions, before and after the infusion of 500-mL of saline. Patients in whom fluid administration induced an increase in cardiac index (PICCO-2) >=15% were defined as “responders”. The respiratory variations of the IVC diameter, CVP and IAP were calculated as (end-inspiratory - end-expiratory values)/mean value. The compliance of the IVC was estimated by the ratio between (end-expiratory - end-inspiratory) values of IVC diameter and CVP.

**Results:** Fluid administration increased cardiac index by more than 15% in 9 patients. The respiratory variations of the IVC diameter predicted fluid responsiveness (area under the ROC curve: 0.799 (95%CI: 0.591-0.931), p<0.05). Before fluid administration, the compliance of the IVC was not different between responders and non-responders (0.75±0.32 vs. 0.79±1.14 mm/mmHg, p=0.91), whereas the respiratory variations of the CVP were higher in responders than in non-responders (36±24 vs. 20±10 %, p=0.03). The respiratory variations of the IVC diameter were associated with the respiratory variations of CVP (r=0.49, p=0.01) but not of IAP (r=-0.12, p=0.56).

**Conclusions:** The respiratory variations of the IVC diameter rather depend on the respiratory variations of the CVP than on the IVC compliance. The IAP seems to not be involved in the respiratory variations of the IVC diameter.

## P286 Role of lung ultrasonography for assessment of endpoints of fluid therapy in patients with hypovolemic and septic shock

### A Moustafa, A Fahmy, M Soliman, G Hamid

#### Cairo University, Cairo, Egypt

**Introduction:** Assessment of hemodynamic status and lines of management of the acute circulatory shock state remains a challenging issue in Critical Care [1]. As the use of invasive hemodynamic monitoring declines, bedside-focused ultrasound has become a valuable tool in the evaluation and management of patients in shock.

**Methods:** This is a prospective cross sectional study included 25 pts admitted with septic or hypovolemic shock in the ICU, full clinical examinations & labs, ECG, chest x-ray (CXR), CVP measurement & lung U/S (2 patterns identified A profile denotes normal lung tissue & lung sliding while B profile indicates subpleural interstitial edema).Assessment of Fluid responsiveness in patients with A profile received IV 0.9% NS till the end point(B lines) denoting starting of alveolar edema or stabilization of his hemodynamics. US findings before & after resuscitation were correlated with hemodynamic changes (MAP, HR & CVP), CXR & Outcome (Mortality within first 24 hours after admission).

**Results:** Patients with A profile lung U/S pattern after resuscitation the CXR findings were free in 12 Patients & congested in 2 patients but patients with B profile lung U/S pattern after resuscitation the CXR findings were free in 2 patients & congested in 8 patients. Analysis showed Sensitivity 80%, Specificity 85.7%, & Accuracy 83.3% (p<0.001). CVP readings after resuscitation in patients with CVP <= 12 CmH2O pulmonary congestion was absent while patients with CVP > 12 CmH2O 10 patients developed lung congestion and 5 patients did not. Analysis showed Sensitivity 100%, Specificity 64.3% & Accuracy 79.2% (p<0.001). Mortality rate in the 1st 24 hours, 3 patients died. 1 died early during the fluid resuscitation and the other 2 died later after the fluid resuscitation representing 12% of the studied patients.

**Conclusions:** Bedside Lung U/S has a good sensitivity in detecting early signs of pulmonary congestion & can guides fluid therapy in shock management.

**Reference**

1. Vincent JL et al, Intensive Care Med (2008)

## P287 A study on the predictability of global end-diastolic volume index (GEDI) through brain natriuretic peptide (BNP) measurement in the initial infusion management of septic patients with mechanical ventilation

### H Yasuda

#### Kameda Medical Center, Chiba, Japan

**Introduction:** Among various measurement methods for prediction of the adequate infusion volume for septic patients requiring mechanical ventilation, the effectiveness of transpulmonary thermodilution technique (TPTD) and Global End-Diastolic Volume Index (GEDI) has become the focus of attention in recent years. However,, to insert the dedicated arterial pressure line and central vein line are required for the measurement of GEDI. BNP, on the other hand, has been shown to be useful for an index to rule-in/rule-out patients with acute heart failure. The aims of this study are to verify the usability of BNP as an index for infusion management in treating septic patients requiring mechanical ventilation.

**Methods:** This study is a post-hoc analysis of RCT (TPTD vs. EGDT) of an infusion management method for septic patients requiring mechanical ventilation conducted at 16 ICUs in Japan between October 2013 and March 2016. The correlation between BNP collected at 0, 24, 48, and 72 hours and GEDI measured at the same time was examined. Since the dataset used in this study consists of repeated measurement data, the analysis used the general linear mixed effect model (GLMM). The multivariate analysis adjusted with age, Cr, and cardiac index was also conducted.

**Results:** Of the 143 patients with the total BNP measurements conducted for 412 times and GEDI measurements for 171 times, the median of age and SAPS2 were 73 (IQR 62-80) and 53 (IQR 43-67), and the hospital mortality rate was 25%. The univariable analysis and the multivariable analysis using GLMM respectively found statistically significant differences, with regression coefficient at 0.03 95%CI 0.01-0.06 (p=0.02), and 0.06 95%CI 0.03-0.09 (p<0.001).

**Conclusions:** While a positive correlation between GEDI and BNP was statistically identified, its effect may be minor in clinical terms, and its significant clinical difference remains unclear.

## P288 Impact of nutritional support in the fluid balance

### R Marinho^1^, H Castro^1^, J Moura^2^, J Valente^3^, P Martins^4^, P Castelões ^5^, C Magalhaes^6^, S Cabral^7^, M Santos^8^, B Oliveira^8^, A Salgueiro^4^, F Coelho^7^, M Lopes^1^, B Xavier^9^, M Melo^9^, S Castro^6^, S Duarte^3^, N Santos^3^, E Lafuente^10^, A Marinho^1^

#### ^1^Centro Hospitalar do Porto, Porto, Portugal,^2^Unidade Local de Saude do Alto Minho, Viana do Castelo, Portugal,^3^Unidade de Saude Local de Castelo Branco, Castelo Branco, Portugal,^4^Centro Hospitalar e Universitario de Coimbra, Coimbra, Portugal,^5^Centro Hospitalar de Vila Nova de Gaia, Vila Nova de Gaia, Portugal,^6^Centro Hospitalar do Algarve, Algarve, Portugal,^7^Instituto Portugues de Oncologia do Porto, Porto, Portugal,^8^Faculdade de Ciencias da Nutricao e Alimentacao da Universidade do Porto, Porto, Portugal,^9^Centro Hospitalar Baixo Vouga, Aveiro, Portugal,^10^Centro Hospitalar Tamega e Sousa, Penafiel, Portugal

**Introduction:** Fluids are a cornerstone of the management of critically ill patients who are at risk of multiple organ dysfunction syndrome. However positive fluid balance (FB) is associated with worse morbidity and mortality in this population, so fluid administration needs to be carefully titrated and the nutritional support products must be taken in consideration.

Objective: Evaluate the impact of nutritional support in the fluid balance in a Intensive Care Unit

**Methods:** Observational prospective study, conducted in eleven Portuguese ICUs of nine general hospitals. Patients with 18 years of age or older were eligible if they were ventilated and had a length of stay (LOS) in ICU greater than 7 days. Demographic data, fluid balance along type of nutritional support used in the first 7 days and were collected from the selected patients.

**Results:** 130 patients were enrolled, 63.8% were male, the median age - 64±16 (19-91), ICU LOS - 15.4±6.1 days, mortality rate of 26.9% (35). 70 % of patients were admitted for medical reasons, 31.5% had normal weight, the remaining patients were either overweight or obese. The average daily FB in the eight days was 258 ± 464 ml, being the maximum at day 1 with +1152 ml, slowly trending down reaching a neutral balance at day 4 and reaching -224 ml at day 7. In the first days the majority of the intake is due to resuscitation driven fluids, however the nutritional support contribution rises as the days passes, reaching 25% at day 4 and 35% at day 7 (Fig. 1). Regarding the administration route, the enteral route was responsible to 28,9% of fluids at day 7 compared to 6,5% of parenteral route.

**Conclusions:** The nutritional support is an factor to take into account regarding fluid balance in Intensive Care Units. In this study after the 4th day the nutritional support, it was responsible for more than 25% of the total volume that was delivered to the patient and with an higher impact with the increase in LOS

**Fig. 1 (abstract P288).**
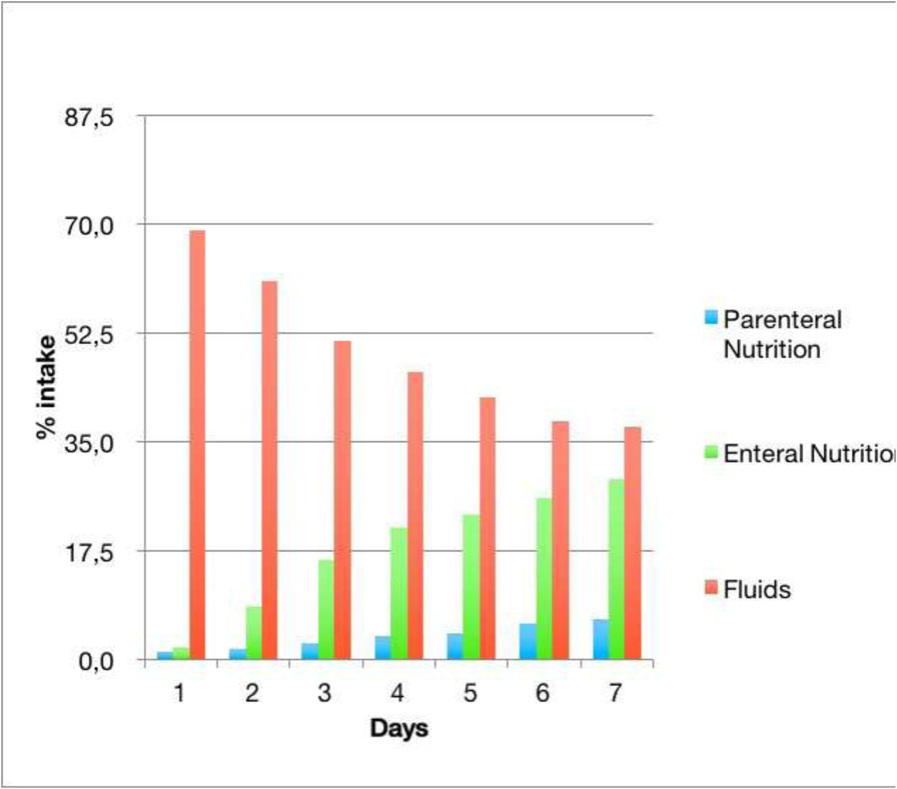
The contribution of nutritional support (enteral and parenteral) and fluids regarding the overall intake per day

## P289 The effect of fluid overload on length of stay ICU and duration of mechanical ventilation after cardiac surgery

### L Delmas Benito, J Haenen, M Koopmans, P Koetsier, E De Widt, EC Boerma

#### Medical Center Leeuwarden, Leeuwarden, Netherlands

**Introduction:** Although fluid therapy remains the foundation of shock resuscitation, fluid overload is associated with longer mechanical ventilation, renal failure and even mortality. Therefore, the benefit of fluid expansion associated with increased cardiac output and tissue perfusion should be balanced against the risk of pulmonary and tissue edema. The aim of this study is to investigate the effect of fluid overload on mechanical ventilation and length of stay in the Intensive Care Unit (LOS ICU) in post-cardiac surgery patients.

**Methods:** In this retrospective single-center observational study the fluid balance, after 12 hours of ICU admission of post-cardiac surgical patients, were evaluated. The LOS ICU and the duration of mechanical ventilation until the first extubation were recorded. Fluid balances (FB) were divided into quartiles and the 75th percentile was defined as high FB.

**Results:** 750 patients were included. The duration of mechanical ventilation and LOS ICU both increased noticeably in the fourth quartile compared to the first 3 quartiles (see Figs. 1 and 2). Moreover, for every liter increase in fluid balance there was an associated 1.5 risk increase of having prolonged mechanical ventilation, and a two-fold risk of extended LOS ICU (Tables 1 and 2).

**Conclusions:** Fluid overload in post-cardiac surgery patients is independently associated with prolonged mechanical ventilation and extended LOS ICU.

**Table 1 (abstract P289).** Binary regression analysis for prolongued mechanical ventilation

Outcome variables	Odds ratio	CI 95%	P-value
Cumulative fluid balance (L)	1.547	1.378-1.737	<0.001
APACHE III score	1.016	1.004-1.029	0.009
ECC (min)	1.004	1.001-1.007	0.015
Additive euroSCORE 1	1.070	1.006-1.137	0.032

**Table 2 (abstract P289).** Binary regression analysis for LOS ICU

Outcome variables	Odds ratio	CI 95%	P-value
Cumulative fluid balance (L)	2.015	1.737-2.337	<0.001
APACHE III score	1.028	1.014-1.042	<0.001
ECC (min)	1.007	1.004-1.011	<0.001
Additive euroSCORE 1	1.109	1.033-1.191	0.004
Post-operative LVEF	1.839	1.092-3.096	0.022

**Fig. 1 (abstract P289).**
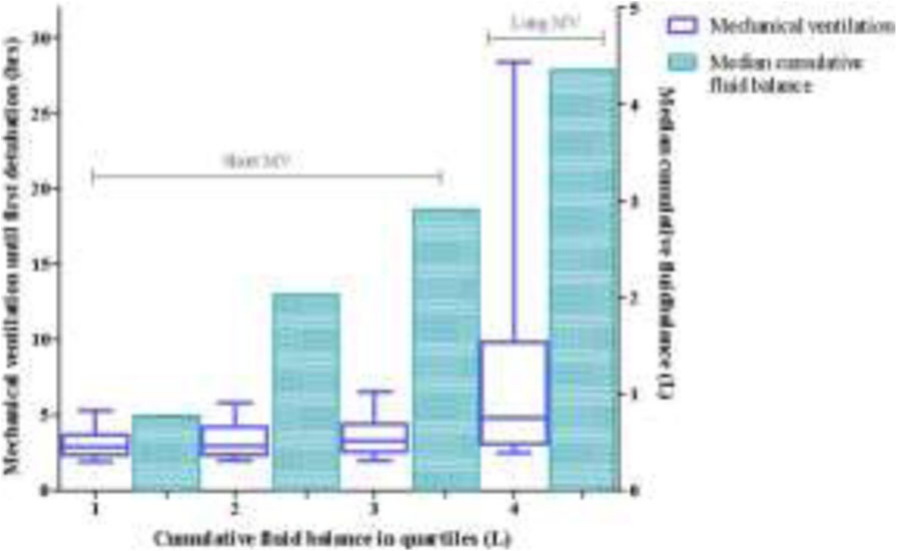
Quartiles of fluid balance in relation to mechanical ventilation

**Fig. 2 (abstract P289).**
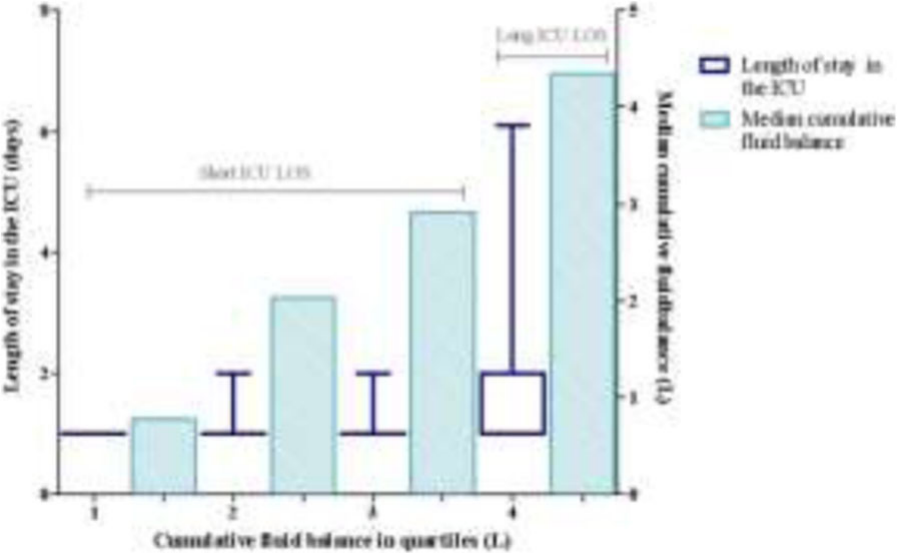
Quartiles of fluid balance in relation to LOS ICU

## P290 Impact of fluid accumulation on the survival of patients with septic shock

### C Villavicencio, J Leache, M Cartanya, R Carbonell, A Rodriguez, M Bodí

#### Juan XXIII University Hospital, Tarragona, Spain

**Introduction:** To evaluate the fluid accumulation during the first week of septic shock and its impact on mortality.

**Methods:** Prospective, observational study, conducted in a polyvalent ICU. 64 patients with diagnosis of septic shock were included in a period of 18 months. Demographic characteristics, comorbidities, APACHEII, SOFA, SAPSII, hemodynamic parameters, daily fluid balance (DFB), daily cumulative balance (DCB), percentage of fluid accumulation and the appearance of fluid overload were analysed during the first week of hospitalization. The impact in 28-day mortality was evaluated. Definitions: DFB: Differences between volume income and daily diuresis; DCB: Addition of the daily DFB; Percentage of fluid accumulation: DCB divided by weight at admission x 100; Fluid overload: Percentage of fluid accumulation more than 10%.

**Results:** We included 64 patients with mean age 65 years, 53% male, APACHE 28 ± 7, SAPS II 56 ± 20, SOFA in admission 8 ± 3, mechanical ventilation 76%, continuous renal replacement techniques 38%. The mean total volume administered during the first 7 days was 26 ± 8L with a mean DCB of 16 ± 8L and a mean fluid accumulation of 21% ± 13. Regarding Fluid accumulation: 17% have <10%, 35% between 10-20% and 47.5% > 20%. 28th-day mortality and ICU mortality were 17% i 28% respectively. During the first week, the percentage of fluid accumulation was significantly higher in non-survivors than in survivors (28.5 ± 10.7 L vs. 18.7 ± 13.1 L, p 0.046) (Fig. 1). Cumulative survival was significantly lower (logRank = 6.05, p=0.01) in patients with >20% of volume gain since the 6th day (Fig. 2). >20% volume gain in the 6th day is a independently associated variable to mortality after adjusting by age, APACHE and haemodialysis (OR = 7.3; CI 95% 1.2-43.9; p = 0.02) (Table 2).

**Conclusions:** In septic shock patients, Fluid overload more than 20% since 6-day of evolution is associated with a higher 28th-day mortality. Its early detection may influence the prognosis and survival.

**Table 1 (abstract P290).** General characteristics

General characteristics, ICU admission (n 64)	Survivors* (n 53)	Non-survivors* (n 11)	p
Age (mean, SD)	66 ± 14	67 ± 9	0.825
Sex male (%)	53	55	0.431
APACHE II (mean, SD)	28 ± 7	28 ± 11	0.938
SAPS II (mean, SD)	56 ± 18	53 ± 29	0.598
SOFA (mean, SD)	8 ± 2	9 ± 3	0.123
Abdominal sepsis (%)	38	6	0.361
		28-day mortality 17%. ICU mortality 28%	

**Table 2 (abstract P290).** Logistic regression

Logistic regression	Odds ratio CI 95%	p
Age	1.016 (0.946-1.091)	0.660
APACHE II	0.940 (0.838-1.054)	0.288
Hemodialysis	0.899 (0.156-5.184)	0.905
Fluid overload >20%	9.421 (1.342-66.139)	0.024

**Fig. 1 (abstract P290).**
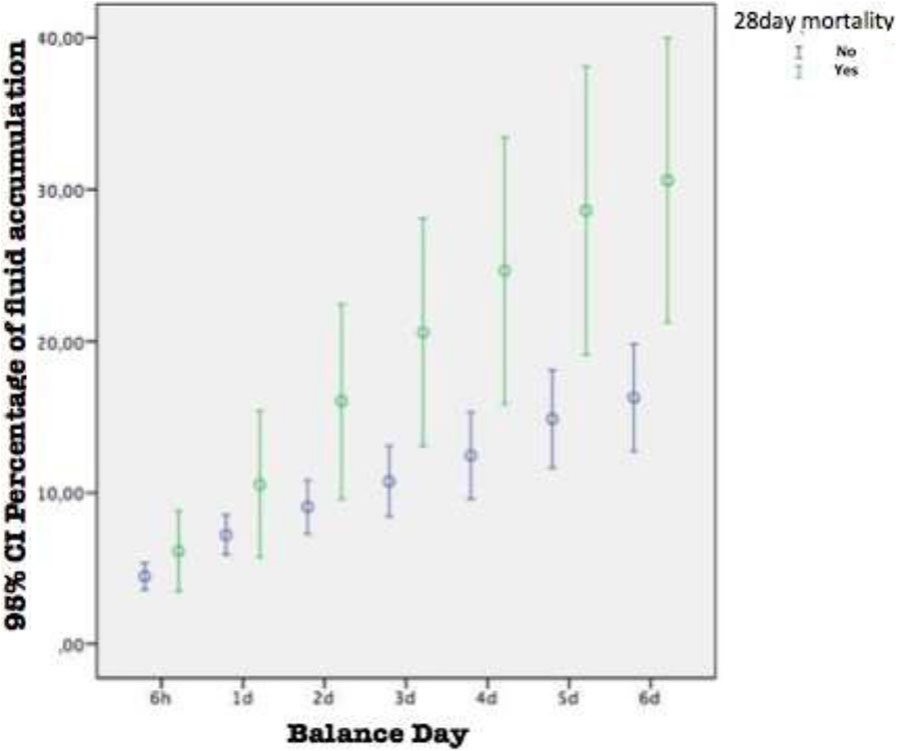
28th day mortality according to percentage of fluid accumulation

**Fig. 2 (abstract P290).**
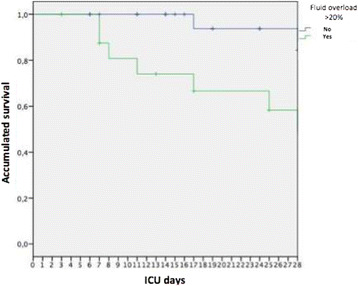
cumulative survival in patients with >20% of volume gain since the 6th day

## P291 Balance of fluids and its association with mortality in patients with ards

### N Canedo, J Baltazar, A Esquivel, A Cano

#### IMSS Centro Medico La Raza. Hospital de Especialidades., Mexico City, Mexico

**Introduction:** Acute pulmonary injury is a frequent income pathology to the intensive care unit (ICU) [1-3]. The patients with ALI/ARDS course with pulmonary edema because of alterations in the alveoli – capillary membrane. Frequently these kinds of patients require aggressive hydric management and positive hydric balance meanwhile the sickness.

**Methods:** to determine association between the magnitude of positive hydric balance and the mortality. Calculated APACHE II and SOFA, verify use and maximal doses of vasoactive drugs, hydric incomes and outcomes in 48 hours of length of stay, VM days and patient status at 28 days. Divided the sample in two groups, high/low hydric balance for the analysis.

**Results:** Included 90 patients, 61% were men, mean age 48.9 ± 16.56 years, APACHE II 17 (RIC 12-24), SOFA 8 (RIC 6-10) 61% of patient’s ARDS 48% die, 30 with ARDS and 12 with LPA. 82% length of MV was 14 (RIC 9-19) days, ICU LOS 13 (RIC 10-18 days). The most frequent cause of ICU income was Pneumonia 49% (44 patients), mean 48 hours’ hydric balance was 1,391.5 (572 - 2,438) ml, 1433 ml positive hydric balance was identified as cutoff by ROC. High hydric balance RR 3.126 (IC 95% 1.177 - 8.355, p= 0.022) and ARDS RR 3.37 (IC 95% 1.220 - 9.309, p= 0.019) were identified as independently mortality risk factors.

**Conclusions:** A >= 1433 ml hydric balance at 48 hours of ICU stay and ARDS, is an independently mortality risk factor.

**References**

1. Blank R, Napolitano L. Crit Care Clin 2011;27:439-58.

2. Ashbaugh DG, Bigelow DB, Petty TL, et al. Lancet 1967;2:319-23.

3. Murray J, Matthay M, Luce J, et al. Am Rev Respir Dis 1988;138:720-3.

## P292 Association between fluid overload and SOFA score kinetic from admission to day 5 during septic shock: results of EPIGOAL study

### X Chapalain^1^, V Vermeersch^1^, P Egreteau^2^, J Oilleau^1^, G Prat^1^, O Huet^1^

#### ^1^Brest University Hospital, Brest, France, ^2^Morlaix Hospital, Morlaix, France

**Introduction:** Sepsis is defined as a life-threatening organ dysfunction due to a deregulated host response to infection [1]. Fluid infusion is one of the cornerstones of sepsis resuscitation therapies. One of the major adverse effects reported is fluid overload (FO). The objective of this study was to assess influence of FO on SOFA score changes from day 0 to day 5.

**Methods:** This study is a retrospective, multicenter, epidemiologic data analysis. It was performed in three French ICUs. All adult patients admitted for septic shock, caused by peritonitis or pneumonia and mechanically ventilated, were enrolled. Delta SOFA score was defined as the SOFA score measured on admission minus SOFA score measured on day 5.

**Results:** 129 patients met the inclusion criteria of the study. FO occurs in about 40% of the patients. Cumulative fluid balance at day 5 was greater in the FO group (2.738 versus 8.715 ml, p < 0.001) (Table 1). Delta SOFA score was higher in the no FO group than in the FO group (4.52 versus 2.15, p = 0.001) (Fig. 1). There was a stepwise decrease of delta SOFA score when duration of fluid overload was greater (p = 0.001) (Fig. 2). In linear modelling, association between FO status and delta SOFA score was confirmed with an adjusted RR of 0.15 [0.03-0.63] (p = 0.009) (Table 2).

**Conclusions:** 1) FO patients had more prolonged multi-organ failure during septic shock; 2) The longer the FO is the longer the more multi-organ failure last.

**Reference**

1. Seymour CW et al. JAMA; 315(8):762–74, 2016

**Table 1 (abstract P292).** Baseline characteristics and outcomes according to fluid overload status

Characteristics	All patients, (n = 129) *	No fluid overload, (n = 73)	Fluid overload, (n = 47)	p-value
Age, mean (SD), years	65.69 (11.95)	66.88 (10.08)	63.20 (13.45)	0.091§
Male sex, n (%)	94 (73.4)	56 (76.7)	33 (70.2)	0.562
Site of infection, n (%)				
Pneumonia	94 (75.2)	55 (78.6)	32 (69.6)	0.381
Peritonitis	32 (25.6)	16 (22.9)	14 (30.4)	0.487
Daily fluid balance, mean (SD)				
Day 1	1,604.44 (2,806.21)	1,163.82 (1,848.98)	2,117.77 (3,915.37)	0.075§
Day 2	1,618.97 (1,547.52)	1,142.68 (1,431.51)	2,387.66 (1,443.90)	<0.001§
Day 3	1,263.71 (2,955.08)	608.88 (1,387.50)	2,475.89 (4,213.05)	0.001§
Day 4	459.16 (1,642.37)	88.68 (1,561.96)	1,041.81 (1,653.48)	0.002§
Day 5	91.20 (1,325.04)	-272.52 (1,184.24)	691.45 (1,410.06)	<0.001§
Cumulative fluid balance from day 1 to day 5	5,041.86 (6,789.93)	2,738.05 (4,217.44)	8,715.49 (8,619.00)	<0.001§
Delta SOFA score between day 1 to day 5	3.55 (4.01)	4.52 (3.74)	2.15 (3.50)	0.001§
Outcomes				
ICU length of stay	17.15 (19.06)	11.71 (10.61)	27.66 (25.35)	<0.001§
28-day mortality, n (%)	44 (34.1)	21 (28.8)	15 (31.9)	0.870
90-day mortality, n (%)	55 (42.6)	25 (34.2)	22 (46.8)	0.236
Ventilator free-days at day 28	10.81 (10.59)	13.84 (10.96)	7.19 (8.43)	0.001§
Duration of RRT	4.50 (3.17)	5.21 (3.18)	3.85 (2.83)	0.019§
Catecholamine free-days at day 10	1.77 (4.47)	1.47 (4.92)	2.45 (4.04)	0.257
§ represents p-value considered statistically sign				
* Fluid overload status is lacking for 9 patients				

**Table 2 (abstract P292).** Results of multivariate linear modeling with delta SOFA score as outcome and fluid overload as principal independent covariate

Variables	Unadjusted RR	CI 95%	p-value	Adjusted RR	CI 95%	p-value
Fluid overload status	0.09	[0.023 - 0.38]	0.001§	0.15	[0.03 - 0.63]	0.009§
Covariates						
Age	1.09	[1.02 - 1.16]	0.006§	1.03	[0.97 - 1.09]	0.4
Weight at baseline	1.00	[0.97 - 1.04]	0.8	0.98	[0.95 - 1.01]	0.18
Cardiovascular disease	6.6	[1.55 - 28.5]	0.011§	4.4	[1.10 - 17.6]	0.036§
Chronic renal insufficiency	0.81	[0.10 - 6.69]	0.84	0.16	[0.02 - 1.28]	0.084
Fluid intake at baseline	1.0	[0.99 - 1.00006]	0.122	1.0	[0.9997 - 1.0002]	0.68
SOFA score at baseline	1.8	[1.43 - 2.24]	<0.001§	1.72	[1.39 - 2.13]	<0.001§
Heart rate at baseline	1.0	[0.97 - 1.29]	0.83	1.0	[0.97 - 1.03]	0.998
Length of hydrocortisone infusion	0.89	[0.73 - 1.07]	0.22	0.93	[0.78 - 1.1]	0.38

§ p-value considered significant

**Fig. 1 (abstract P292).**
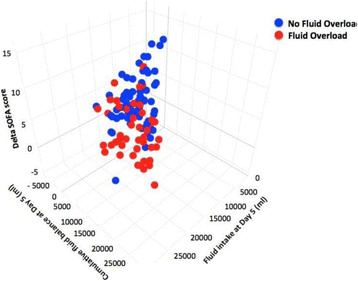
Distribution of delta SOFA score, cumulative fluid balance and fluid intake in patients with or without fluid overload status

**Fig. 2 (abstract P292).**
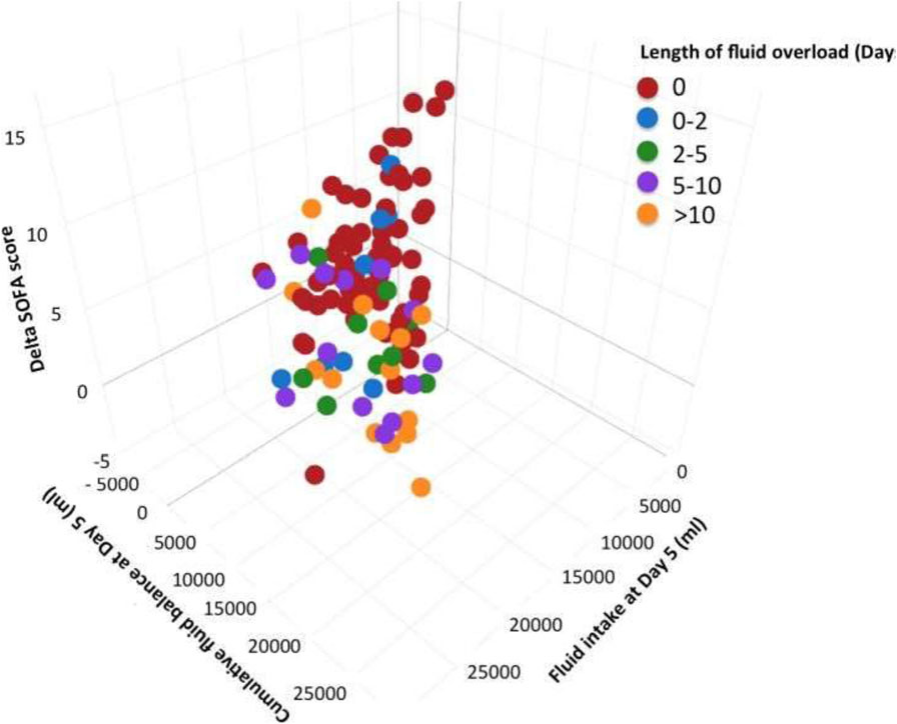
Distribution of delta SOFA score, cumulative fluid balance and fluid intake considering length of fluid overload

## P293 Large-volume crystalloid fluid is associated with increased hyaluronan shedding and inflammation in a hemorrhagic shock model

### L Smart^1^, C Boyd^2^, M Claus^2^, E Bosio^1^, G Hosgood^2^, A Raisis^2^

#### ^1^Harry Perkins Institute of Medical Research, Perth, Australia, ^2^Murdoch University, Murdoch, Australia

**Introduction:** Rapid administration of crystalloid or colloid fluids may cause endothelial glycocalyx shedding, thereby increasing inflammation.

**Methods:** Greyhounds under general anesthesia subject to hemorrhage for 60 minutes were given 20 mL kg-1 of either fresh whole blood (FWB), hydroxyethyl starch (HES) 130/0.4, 4% succinylated gelatin (GELO), or 80 mL kg-1 of isotonic crystalloid (CRYST) over 20 minutes (n=6 per group). Plasma biomarkers hyaluronan, interleukin (IL) 6, 8, 10, tumor necrosis factor-alpha, monocyte chemoattractant protein-1, keratinocyte chemokine-like (KC)) and atrial natriuretic peptide were measured at baseline, end of hemorrhage (Shock), end of fluid bolus (T20), and then 40 (T60), 100 (T120) and 160 (T180) minutes later. Cardiovascular parameters were also measured at above time points. Biomarker change from baseline (fold-change), indexed to hemoglobin, was compared between groups using mixed effects models (Bonferroni-Holm corrected P<0.05).

**Results:** Minor differences in measures of shock between groups after fluid administration resolved by T120. CRYST showed increased fold-change in hyaluronan compared to other groups at T20 (FWB P=0.019, HES P<0.001, GELO P<0.001), T60 (FWB P<0.001) and T120 (FWB P<0.001) (Fig. 1). GELO had increased fold-change in hyaluronan compared to other groups at T20 (HES P=0.009), T60 (FWB P<0.001) and T120 (FWB P<0.001, CRYST P=0.006), as did FWB at T20 (HES P=0.008).

CRYST showed increased fold-change in IL10 compared to other groups at T20 (HES P<0.001, GELO P=0.002), T60 (HES P=0.001, GELO P=0.005,), T120 (HES and GELO P<0.001) and T180 (HES and GELO P<0.001) (Fig. 2), of IL8 at T60 (GELO P=0.006), and of KC at Shock (FWB P=0.002, GELO P=0.007), T20 (FWB P=0.009, GELO P=0.007), and T120 (GELO P=0.002).

**Conclusions:** Rapid large-volume crystalloid given for hemorrhagic shock was associated with increased hyaluronan, a biomarker of endothelial glycocalyx damage, and inflammation, including increased IL10, IL8 and KC.

**Fig. 1 (abstract P293).**
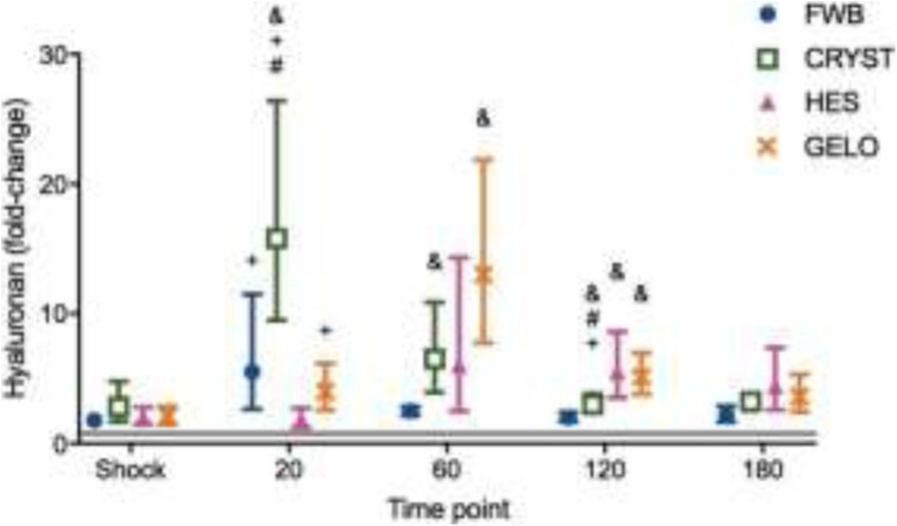
Plasma hyaluronan (geometric mean fold-change, 95% confidence interval) in dogs (n=6 per group) with hemorrhagic shock given 20 mL kg-1 of either fresh whole blood (FWB), hydroxyethyl starch 130/0.4 (HES), 4% succinylated gelatine (GELO) or 80 mL kg-1 of balanced isotonic crystalloid (CRYST). Data represents fold-change in biomarker concentration from baseline. Grey line (fold-change of 1.0) represents no change from baseline

**Fig. 2 (abstract P293).**
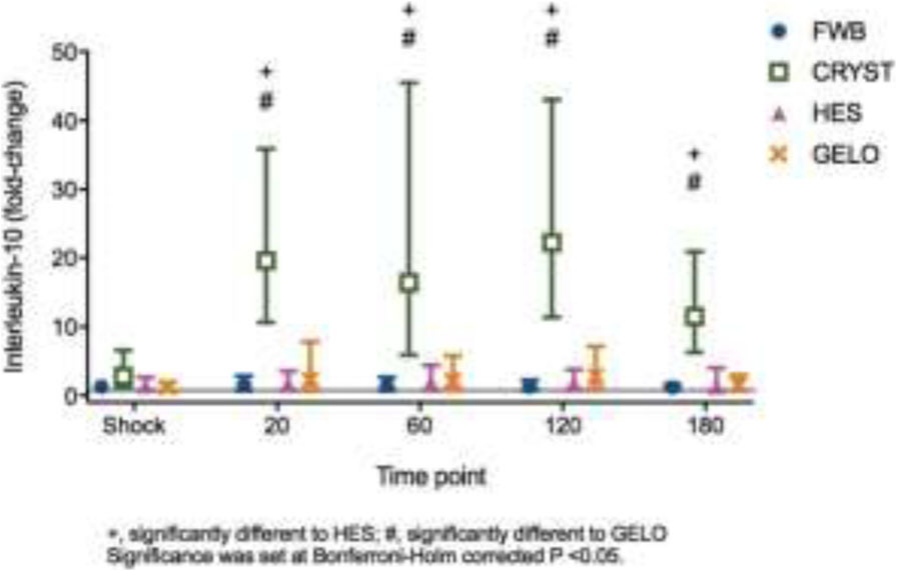
Plasma interleukin-10 (geometric mean fold-change, 95% confidence interval) in dogs (n=6 per group) with hemorrhagic shock given 20 mL kg-1 of either fresh whole blood (FWB), hydroxyethyl starch 130/0.4 (HES), 4% succinylated gelatine (GELO) or 80 mL kg-1 of balanced isotonic crystalloid (CRYST). Data represents fold-change in biomarker concentration from baseline. Grey line (fold-change of 1.0) represents no change from baseline

## P294 Immediate hemodynamic response to fluid challenge is independent of fluid type

### BR Hipszer^1^, A Joosten^2^, P Van der Linden^3^, F Hatib^1^

#### ^1^Edwards Lifesciences, Irvine, California, USA,^2^CUB Erasme, Université Libre de Bruxelles, Brussels, Belgium,^3^CHU Brugmann, Brussels, Belgium

**Introduction:** A bi-center randomized controlled trial has recently been published that investigates the impact of the type of fluid (crystalloid versus colloid) on patient outcome following major surgery [1]. The study used a closed-loop fluid delivery system to eliminate the clinician bias when determining when to deliver fluids. The goal of the current analysis is to compare the immediate hemodynamic response to 100 ml fluid boluses of either a crystalloid or a colloid solution.

**Methods:** Patient consent was obtained prior to transferring the data from [1] to Edwards Lifesciences for further post-hoc analysis. The percent change in stroke volume (DSV) following each 100mL bolus was tabulated and cross-referenced to the type of fluid. The responder rate and the DSV cumulative distribution function (CDF) were determined for each type of fluid administered. A responder was defined as a DSV >= 5% for a 100mL fluid challenge. The mean DSV was compared between the two groups using a student t-test.

**Results:** From the 160 datasets reported in [1], 119 were used in the analysis. Descriptive statistics are summarized in Table 1 and the CDFs are plotted in Fig. 1. More crystalloid boluses were administered. In both groups, the responder rate was around 50%. Mean DSV was not significantly different between groups (p = 0.57).

**Conclusions:** We observed similar responder rates and CDFs with the two fluid types, suggesting that the immediate hemodynamic response to 100 ml fluid boluses is independent from the fluid type. We therefore hypothesized that it is the longer intra-vascular persistence of the colloid that explain the lower number of boluses required to achieve the hemodynamic endpoints targeted in the clinical study [1].

**Reference**

[1] Joosten et al. Anesthesiology 128:55-66, 2018

**Table 1 (abstract P294).** Descriptive statistics of dataset

Fluid Type	Crystalloid	Colloid
Number of Cases	57	62
Number of Boluses	873	578
Responder Rate	49%	51%
Mean [SD] DSV	5.6 [13.2]	6.0 [12.0]

**Fig. 1 (abstract P294).**
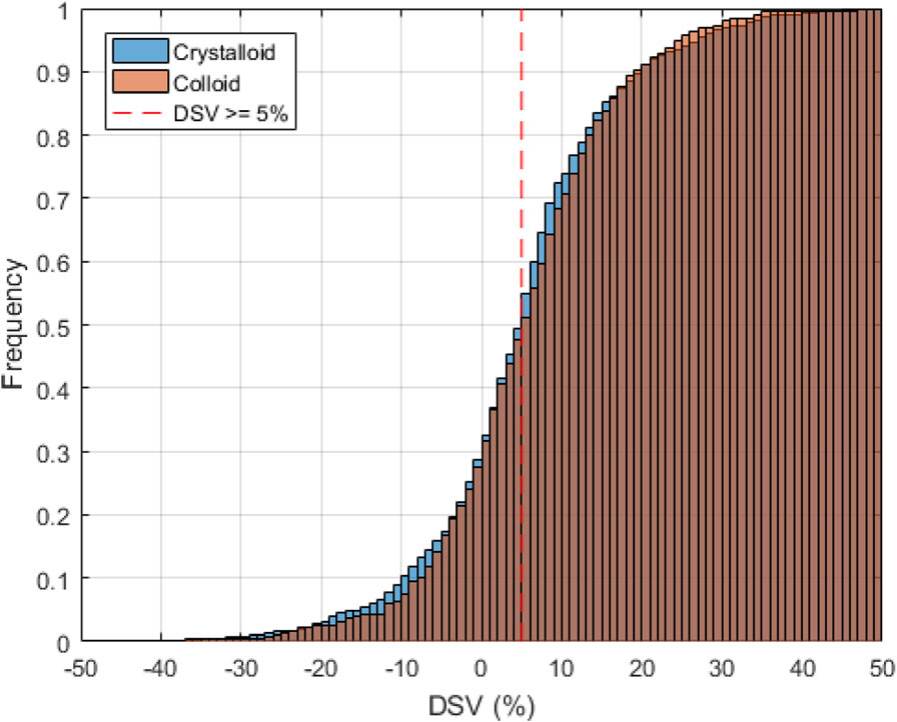
Cumulative distribution functions of delta stroke volume for crystalloid and colloid fluid boluses

## P295 A low serum albumin level in patients with burn shock is an independent risk factor for death.

### V Bagin, V Rudnov, M Astafeva, O Krasnoperova, I Korobko, V Vein

#### City Clinical hospital №40, Yekaterinburg, Russia

**Introduction:** Definitive large randomized controlled trial investigating the use of albumin for burn shock resuscitation have yet to be conducted. To date, we have a meta-analysis [R.J. Navickis et al, 2014] that suggests that albumin can improve outcomes of burn shock resuscitation. But many aspects of albumin transfusion remain controversially. The aim of this retrospective study was to determine the effect of transfusion of colloids, TBSA, age, sex and serum albumin level on the incidence of AKI and death.

**Methods:** Design: retrospective cohort controlled study. Inclusion criteria: males and females >18 years of age, TBSA burned 20%-80%, absence of AKI (KDIGO) at the admission. Patients were divided in two groups: albumin group – 31 patients received 10% albumin in addition to base fluid therapy with Ringer solution and control group – 32 patients received Ringer solution and other colloids, except albumin.

**Results:** Baseline characteristics were well balanced between groups except for the age. 28-days mortality was significantly different: 48.4 % and 81.2 % in albumin group and control group, respectively, p<0.01. Rate of AKI (KDIGO I-III) was 32.3 % in albumin group and 62.5 % in control group, p=0.02. There wasn’t significant difference between groups in total volume of fluids administered and diuresis in the first 3 days of treatment. To determine the independent risk factors for AKI and death, we performed a logistic and Cox-regression analysis involving factors such as: sex, age, TBSA, deep burns, transfusion of albumin, HES, gelatins and serum albumin level. Independent risk factor for AKI was age (OR 1.09 (95%CI 1.00-1.17), p=0,02). Independent risk factors for death were TBSA (OR 1.04(95%CI 1.01 – 1.08), p=0.03) and serum albumin level (OR 0.85(95%CI 0.74 – 0.98), p=0.03).

**Conclusions:** This study did not show influence of 10% albumin transfusion on mortality and incidence of AKI in patient with burn sock. Nevertheless low serum albumin level is independent risk factors for death.

## P296 Albumin use evaluation in a tertiary care hospital in United Arab Emirates

### SH Rashid, M Ur Rahman, MF Osseni, N Gebran, MF Abumandil

#### Tawam Hospital, Al Ain, Abudhabi, United Arab Emirates

**Introduction:** Impact of implementing albumin guidelines, staff education and interventions from physicians and clinical pharmacist on reducing albumin use in adult critical care unit. Albumin is widely used in the critical care units although its approved indications are limited. In 2016 Medication use evaluation (MUE) was conducted retrospectively to evaluate the appropriateness of albumin use at Tawam Hospital. The MUE prompted critical care albumin prescribing interventions due to high number of doses prescribed 660 (50%) in the hospital (Table 1/Fig. 1).

**Methods:** After the MUE analysis in Oct-Dec 2016, critical care departmental management initiated communication and discussions with physicians regarding high usage. Evidence-based lecture was given and albumin guidelines were implemented by clinical pharmacy with lead physician’s interventions. Intensive Care Unit (ICU) monthly data was pulled retrospectively from Jan 2016 to Sep 2017 using Hospital Information System (HIS).

**Results:** Post intervention data in Jan-Sep 2017 showed significant decrease of doses per month compared to 2016 (Table 2/Fig. 2). Critical care cost per month dropped from 29,029 $ to 20,673 $/month (Table 2). The initial MUE results showed ICU had 18% appropriate albumin doses, 82% controversial doses and no inappropriate dose as per the albumin guidelines.

**Conclusions:** A significant change of prescribing pattern was observed after albumin guidelines implementation, staff education and feedback from clinical pharmacist and lead physician causing significant drop of albumin prescribed doses and cost reduction by 29%. The reduction projected to an average annual saving of 100,272 USD (Table 2, Fig. 2).

**Table 1 (abstract P296).** Initial albumin MUE data (doses) collected (3 months)

Location/Doses	Oct-Dec 2016	Jun-Aug 2017
Intensive Care Unit (ICU)	576	213
High Dependence Unit (HDU)	84	23
Total doses	660	236

**Table 2 (abstract P296).** ICU monthly data collected from 2016 -Sep 2017

Cost	2016	Jan-Sep 2017
Total Cost (dhs)	1,254,077	669,814 (9 months)
Cost / month (dhs)	104,506/mth	74,424/mth
Cost / month (USD)	29,029 $/mth	20,673 $/mth
Average cost /Year (USD)	348,348 $	248,076 $

**Fig. 1 (abstract P296).**
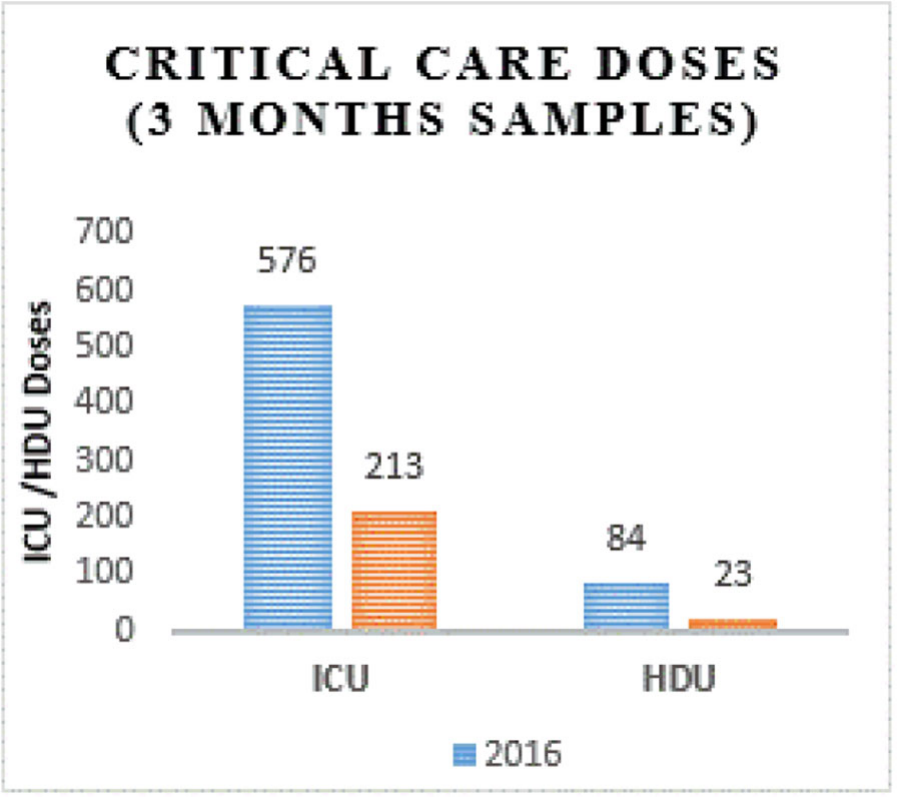
Albumin doses comparing usage in 3 months

**Fig. 2 (abstract P296).**
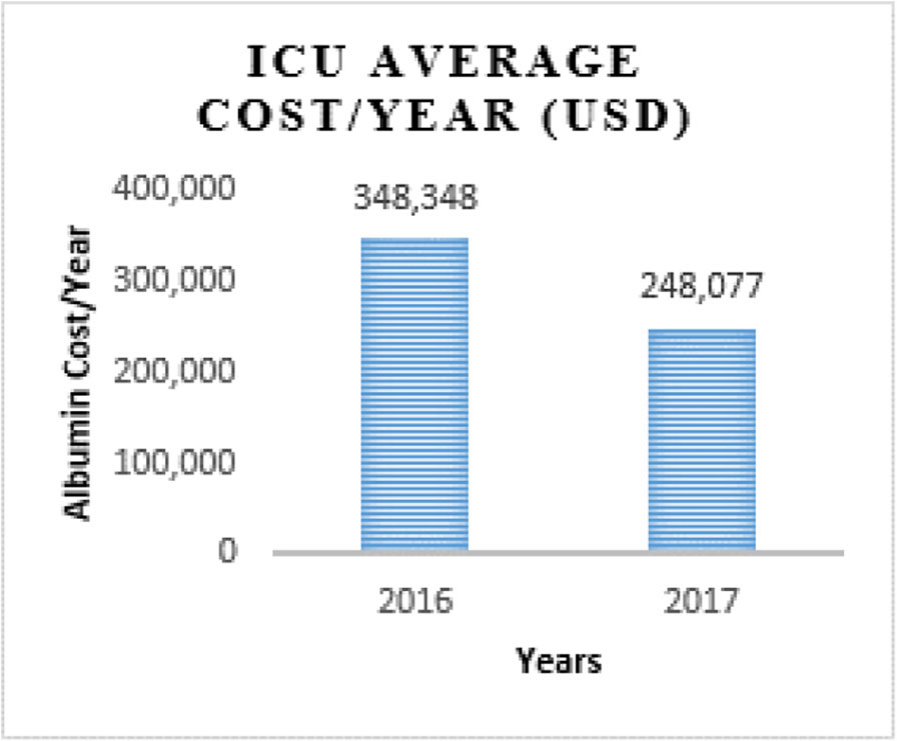
ICU Albumin cost pre & post interventions cost reduction (29%)

## P297 Lactated Ringer versus albumin in early sepsis therapy (RASP study): a randomized clinical trial

### C Park, F Galas, J Fukushima, R Nakamura, G Oliveira, J Almeida, S Rizk, L Hajjar

#### Cancer Institute of Sao Paulo, Sao Paulo, Brazil

**Introduction:** Previous studies have suggested that albumin might be superior when compared to crystalloids in septic shock. However, it is not known if albumin in the first hours of hemodynamic resuscitation improves outcomes in septic shock patients. The aim of this study was to evaluate whether fluid therapy with albumin 4% solution as compared to crystalloid solution (lactated’s Ringer) decreases 7-day mortality in cancer patients with septic shock.

**Methods:** The RASP study was a single-center, double-blind and randomized clinical trial. We randomly assigned 360 patients with cancer and septic shock to receive as resuscitation fluid in the first 12 hours of ICU admission bolus of 500ml of intravenous 4% albumin or lactated’s Ringer. The primary outcome was 7-day mortality. Secondary outcomes included ICU and hospital length of stay, 28-day mortality, daily SOFA score, rates of mechanical ventilation, renal replacement therapy and need of vasopressor drugs.

**Results:** From 1168 eligible patients, 360 were included in the study – 180 in albumin group and 180 in the Ringer group. There were no significant differences between groups in demographic and baseline characteristics. The total amount of administered fluid in the first 12 hours of resuscitation was 2402±642 ml in the albumin group and 2296±629 ml in the Ringer group, P=0.114. The 12-hour fluid balance was 980 ml (428-1397) in the albumin group and 970 ml (240-1358) in the Ringer group, P=0.252. We did not observe any difference between groups in 7-day mortality rates (28.9% in albumin group and 22.2% in Ringer group, P=0.147). There was no difference in 28-day mortality in the groups (55.6% in albumin and 45.6% in the Ringer group, P=0.058). No significant differences in secondary outcomes were observed in the groups.

**Conclusions:** In cancer patients with septic shock, resuscitation with albumin as compared to lactated Ringer did not improve the rate of survival at 7 days.

## P298 5% albumin vs 4% gelation as an early colloid resuscitation after 30 cc./kg of crystalloid resuscitation in surgical septic shock patients admitting to the general surgical ICU

### S Kongsayreepong, A Piriyapatsom, T Yuyen

#### Department of Anesthesiology, Siriraj Hospital, Mahidol University, Bangkok, Thailand

**Introduction:** Colloid has been suggested to be used in septic shock after 30 ml/kg crystalloid resuscitation. Aim of this study was to compare 5% albumin (A gr) & 4% gelatin (G gr) as an early colloid resuscitation in septic shock patients admission to the general SICU in term of efficacy (shock reversal time; stop vasopressor) & safety [incidence of AKI (KDIGO criteria)] in the first 3 days of ICU admission.

**Methods:** This prospective observational study was done in 125 consecutive septic shock patients, age>18 yrs admitting to the general SICU receiving 5% alb vs 4% gelatin 20 cc/Kg after initial 30 ml/Kg of crystalloid whom found to be fluid responsiveness. Patients undergoing cardiac, neurosurgical, traumatic, transplant, receiving FFP or other synthetic colloids were excluded. Demographic data, comorbidities, baseline Hb, Cr, albumin, type of surgery, site of infection, data in the first 3 ICU days (Hb, platelet, BUN, Cr, albumin, lactate & liver function, type & amount of fluid, blood/blood component, vasopressor, APACHE II & SOFA II score), ventilator & ICU days, 28 & 90 days mortality were recorded.

**Results:** Patients in G gr (62) were significant (p<0.05) younger, higher net fluid balanced, higher Hb, lower alb level & higher SOFA II score & had significant longer shock reversal time [60 (42-99) vs 48 (30-84) hrs]. In the patients with admission serum albumin<2.5 mg/d; patient in the G gr had significant higher incidence of AKI [AKI-I: 66.7% vs 39.1%; AKI-II: 53.3% vs 26.1% & AKI-III: 43.3% vs 17.3%) & higher RRT rate (21.0% vs 15.9%). There’re no significant different in ICU stay, 28 or 90 days mortality.

**Conclusions:** This study showed that 4% gelatin solution may not be safe to be used in surgical septic shock patient.

## P299 Hydroxylethyl starch 6% (130/0,4) on pump priming of cardiopulmonary bypass circuit during cardiac surgery: effects on intraoperative fluid balance, transfusion requirement and kidney function

### M Küllmar^1^, C Schmidt^1^, C Windhorst^1^, G Brodner^2^, S Martens^1^, M Meersch^1^, A Zarbock^1^

#### ^1^University Hospital Münster, Münster, Germany,^2^Hospital Hornheide, Münster, Germany

**Introduction:** Colloids are widely used for volume resuscitation. Among synthetic colloids, hydroxyethyl starch (HES) is commonly administered. In cardiac surgery, priming of the cardiopulmonary bypass (CPB) circuit with colloids minimizes resuscitation volume and results in less pulmonary fluid accumulation. However, the use of HES has been associated with a higher incidence of renal damage and a higher occurrence of coagulopathy. The aim of this study was to investigate the effect of low dose (5 - 10 ml/kg) HES 6% (130/0,4) in CPB pump priming on fluid balance, blood loss, transfusion requirement and occurrence of acute kidney injury.

**Methods:** In a pre-post design, data from 1120 patients undergoing cardiac surgery with CPB were analyzed. In 560 patients, priming solution consisted of 1250 ml balanced crystalloids, 250 ml mannitol 15%, tranexamic acid 2g and 500 I.E. heparin. For the other 560 patients, 500 ml of the crystalloids were replaced with HES 6% (130/0.4), the other components were the same. Patients were matched 1:1 with propensity score method. The primary endpoint was intraoperative fluid balance. Secondary endpoints were perioperative blood loss, transfusion requirement and the occurrence of acute kidney injury.

**Results:** In total, 866 patients were analyzed. The HES group showed less positive fluid balance than the crystalloid group (p< 0.001). There was no difference in intraoperative blood loss (p=0.426) and transfusion requirement (p=0.442). The occurrence of acute kidney injury was not significantly different between the two groups (p=0,147).

**Conclusions:** Low-dose administration of 5-10 ml/kg HES 6% (130/0.4) to CPB pump priming decreased intraoperative fluid accumulation without increasing perioperative blood loss and transfusion requirement. There was no effect on the incidence of acute kidney injury. Priming CPB pumps with a low-dose of HES 6% (130/0.4) is an important component for a restrictive volume strategy and might safely be used in patients with preexisting renal dysfunction.

## P300 A randomized trial comparing two balanced fluids in the critically ill

### S Omar, LR Mathivha

#### University of Witwatersrand, Johannesburg, South Africa

**Introduction:** Most crystalloid solutions used in critically ill patients have a greater chloride (Cl) concentration than plasma, which may be detrimental. Replacing some Cl with bicarbonate (HC03) reduces Cl, but may increase partial pressure of carbon dioxide (PC02) in blood. Such an increase in PC02 may be harmful [1]. The main objective was to determine if a HCO3 balanced fluid resulted in increased PaCO2 compared to a conventional balanced fluid.

**Methods:** Single center randomized controlled trial in an adult ICU, comparing balanced fluid (sodium,Na=142mmol/l, Chloride,Cl=99mmol/l, HCO3=49mmol/l) vs conventional fluid (Na=130mmol/l, Cl=110mmol/l, HC03<=27mmol/l). University ethics committee approval:M080932. We used the absolute difference between the PCO2 and 40mmHg as a comparison for the 2 fluid groups. Between-group comparisons of PC02 from D1-D7 was done by repeated measures ANOVA. A p value <0.05 was considered significant.

**Results:** 46 patients were allocated to the conventional group and 40 to the balanced group. At baseline the 2 groups were well matched (p>0.05) for age, weight, gender, severity of illness and organ support. There were no significant differences in PC02 between the two fluid groups, overall or at D1, D5 or D7. The balanced group showed a significant improvement in eGFR (sCr), between D0 and D5 (p=0.02) while the conventional group exhibited a significant decline (p=0.00). There were no significant differences between the 2 groups with respect to fluid requirements, number of positive blood cultures, ICU renal replacement utilization, ICU length of stay, ICU mortality and 28 day mortality.

**Conclusions:** The use of a balanced fluid did not result in an increase in PCO2 and appears to be safe. A beneficial effect on renal function was observed.

**Reference**

1. Wilson RF et al. J Trauma Acute Care Surg 74:45–50, 2013

## P301 Effects of crystalloid vs. colloid volume replacement on microcirculatory perfusion in free flap surgery

### I László^1^, Á Janovszky^1^, N Öveges^1^, Z Lóderer^2^, P József^1^, A Szabó^1^, V Vargán^1^, A Lovas^1^, T Tánczos^1^, A Mikor^1^, D Trásy^1^, Z Molnár^1^

#### ^1^University of Szeged, Szeged, Hungary,^2^Markusovszky Hospital, Szombathely, Hungary

**Introduction:** The effects of crystalloids and colloids on macro- and microcirculation is controversial. Our aim was to compare their effects on microcirculation during free flap surgery when management was guided by detailed hemodynamic assessment.

**Methods:** Patients undergoing maxillo-facial tumour resection and free flap reconstruction were randomized into a crystalloid (ringer-fundin, RF, n=15) and a colloid (6% hydroxyethyl starch, HES, n=15) groups. Cardiac index (CI), stroke volume (SVI) and pulse pressure variation (PPV) were continuously monitored by a non-calibrated device (PulsioFlex - PULSION, MAQUET). Central venous oxygen saturation (ScvO2), venous-to-arterial pCO2-gap (dCO2), lactate levels and hourly urine output was also measured, and a multimodal, individualized approach based algorhithm was applied [1]. Microcirculation was assessed by laser Doppler flowmetry (PeriFlux 5000 LDPM, Perimed Jarfalla, Sweden). Measurements were performed at baseline and from the start of reperfusion hourly for 12 hours. For statistical analysis, two-way RM ANOVA was used.

**Results:** There was no difference between the groups regarding age, sex, length of surgery (whole population: 348 ± 69 min). Patients in the RF-group required significantly more fluid in total (RF: 2581±986, HES: 1803±497 ml, p=0.011). Both groups remained hemodynamically stable (CI, SVI, PPV, ScvO2, dCO2, lactate and urine output) throughout the study. There was no difference between the RF-, and HES-groups in the laser Doppler measurements neither on the control site nor in the flap (Fig. 1).

**Conclusions:** We found that when hemodynamic management is guided by a multimodal assessment and stability is maintained, there was no difference between crystalloids and colloids in macrocirculation and microcirculatory perfusion.

**Reference**

1. Molnar Z et al. Curr Opin Anaesthesiol 171-177, 2017

**Fig. 1 (abstract P301).**
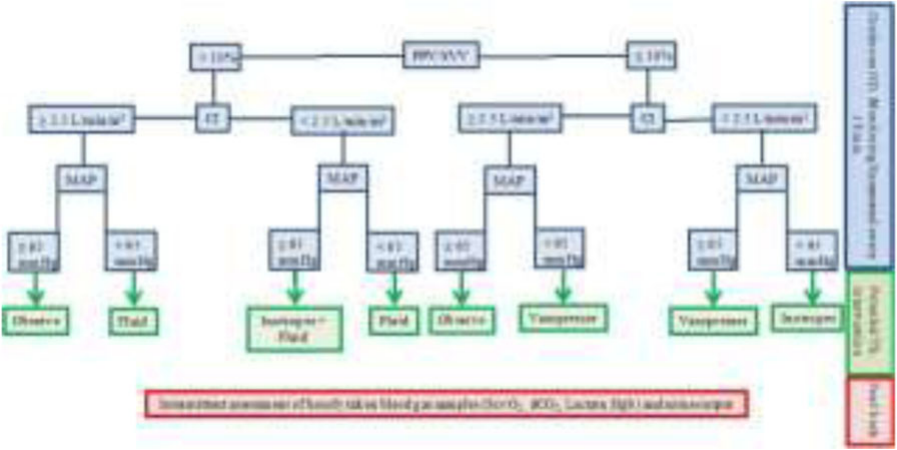
Protocol flowchart

## P302 Deciphering the immunomodulatory consequences of crystalloids

### F Brillant-Marquis, JA Sauvé, P Laplante, P Thebault, M Chassé, FM Carrier, R Lapointe, JF Cailhier

#### CRCHUM/CHUM/Université de Montréal, Montreal, Canada

**Introduction:** Our aim is to evaluate the impact of crystalloid fluids on immune cells. Intensive care unit (ICU) patients’ inflammatory status can switch from an early pro-inflammatory to a late anti-inflammatory phase, which favors infections. They can receive different crystalloids, either Normal Saline (NS), Ringer’s Lactate (RL) or Plasma-Lyte (PL). High chloride concentration present in NS has been associated with various complications [1], whereas high doses of NaCl have inflammatory effects on immune cells [2]. However, the immune consequences of crystalloids in humans are ill-defined.

**Methods:** Using our comprehensive immunemonitoring platform, we assessed the immunological phenotype of peripheral blood mononuclear cells (PBMC) in humans. 11 healthy subjects received a liter of NS, RL and PL. Blood samples were taken before and 6h later. PBMC phenotypes were assessed by flow cytometry and cytokine concentrations were measured by a multiplex assay. 9 off-pump cardiac surgery patients were also randomized to receive either NS, RL or PL during surgery and their stay in the ICU. Blood samples were drawn at various time-points. All leucocytes were analyzed in a similar fashion. We are still recruiting.

**Results:** Study of healthy subject’s PBMC suggested that RL reduced classical monocytes, whereas NS increased lymphocyte activation and IL-17 and MIP-1b levels. In cardiac surgery patients, our preliminary results suggested that RL and PL reduced classical monocytes and increased non-classical monocytes compared to NS. Neutrophils were also affected differently by crystalloids, where NS seemed to activate them more.

**Conclusions:** Our results suggest that crystalloids have different immune consequences. A better understanding of their immune modulation will lead to personalization of their use according to the inflammatory status of patients to restore their immune homeostasis.

**References**

1. Annane D et al. JAMA 310:1809-17, 2013

2. Ip WK et al. Nat Commun 6:6931, 2015

## P303 Hypertonic versus isotonic saline for initial fluid bolus in emergency department patients with suspected infection: a pilot RCT

### L Smart, E Bosio, S Macdonald, D Fatovich, C Neil, G Arendts

#### Harry Perkins Institute of Medical Research, Perth, Australia

**Introduction:** Hypertonic saline for fluid resuscitation may reduce glycocalyx shedding, endothelial activation and inflammation in patients with infection, and reduce the volume of fluid subsequently administered.

**Methods:** This randomised controlled open-label pilot study included 65 patients presenting to an emergency department with suspected infection requiring a fluid bolus. Patients received either a single bolus of 10mL/kg of 0.9% NaCl (isotonic group) or 5mL/kg of 3% NaCl (hypertonic group). Blood biomarker concentrations of glycocalyx shedding (syndecan-1, hyaluronan), endothelial activation (sICAM-1, sVCAM-1) and inflammation (interleukin-6, -8, -10, NGAL, resistin) were measured at T0 (before fluid) and 1 hour (T1), 3 hours (T3) and 12-24 hours (T24) later. Changes in biomarker concentrations were compared between study groups using mixed regression models, with fold-change from T0 reported. Differences in fluid volumes were compared using the Wilcoxon rank sum test. Significance was set at P<0.05.

**Results:** Syndecan-1 concentration in the isotonic group decreased from T0 to T1 (fold-change 0.8, 95% CI 0.7-0.9), which was significantly different to the hypertonic group (fold-change 1.0, 95% CI 0.9-1.1)(P=0.012)(Table 1). Interleukin-10 concentration decreased in the isotonic group from T0 to T24 (fold-change 0.1, 95% CI 0.0-0.3), which was significantly different to the hypertonic group (fold-change 0.8, 95% CI 0.3-2.6)(P=0.006). Otherwise, there were no significant differences in change over time between groups for measured biomarkers. Total fluid volume administered between T0 and T1 was significantly higher in the isotonic group (P<0.001) (Fig. 1) but not different for subsequent time periods.

**Conclusions:** Biomarkers of glycocalyx shedding, endothelial activation and inflammation were not different between patients receiving either 0.9% or 3% saline. Also, 3% NaCl did not reduce administration of additional fluids.

**Table 1 (abstract P303).** Serum concentrations of endothelial glycocalyx biomarker, syndecan-1, and inflammatory cytokine, interleukin-10, in patients with infection that received either 10mL/kg of 0.9% NaCl (isotonic group) or 5mL/kg of 3% NaCl (hypertonic group) in the first hour of hospitalisation (T0 to T1)

	T0	T1	T3	T24
Syndecan-1 (ng/mL)				
Isotonic	1.5 (1.2-1.9)	1.2 (1.0-1.6)	1.4 (1.0-2.0)	2.0 (1.6-2.5)
Hypertonic	1.4 (1.1-1.8)	1.4 (1.1-1.8)	1.5 (1.1-2.0)	2.0 (1.5-2.7)
Interleukin-10 (pg/mL)				
Isotonic	1.9 (0.6-6.3)	1.3 (0.4-4.2)	0.7 (0.2-2.2)	0.4 (0.2-0.9)
Hypertonic	0.5 (0.2-1.3)	0.5 (0.2-1.3)	0.8 (0.3-2.1)	0.5 (0.2-1.4)

**Fig. 1 (abstract P303).**
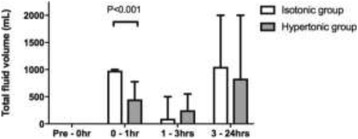
Median (Q1-Q3) total fluid volume, including all crystalloid and colloid fluids, received by patients during the study period

## P304 Acid-base variations of whole blood and isolated plasma induced by acute changes in partial pressure of carbon dioxide in critically ill patients and healthy controls: an in-vitro study.

### T Langer, S Brusatori, E Carlesso, F Zadek, V Castagna, R Limuti, G Giudici, T Mauri, A Zanella, G Grasselli, A Pesenti

#### University of Milan, Milan, Italy

**Introduction:** Acute changes in PCO2 are buffered by non-carbonic weak acids (ATOT), i.e., albumin, phosphates and hemoglobin. Aim of the study was to describe acid-base variations induced by in-vitro PCO2 changes in critically ill patients’ blood and isolated plasma, compare them with healthy controls and quantify the contribution of different buffers.

**Methods:** Blood samples were collected from patients admitted to the ICU and controls. Blood and isolated plasma were tonometered at 5 and 20% of CO2 in air. Electrolytes, pH, blood gases, albumin, hemoglobin and phosphates were measured. The Strong Ion Difference (SID) was calculated [1] and non-carbonic buffer power was defined as β=– ∆HCO3-/∆pH [2]. T-tests and linear regression were used for analysis.

**Results:** Seven patients and 10 controls were studied. Hemoglobin, hematocrit and albumin were lower in patients (p<0.001), while SID and phosphates were similar. PCO2 changed from 29±4 to 108±13 mmHg, causing different blood pH variations in patients and controls (0.43±0.06 vs. 0.36±0.02, p=0.03). Patients had lower blood and plasma β (20±5 vs. 30±4, p<0.001 and 2±2 vs. 4±1, p=0.03, respectively). Figure 1 shows changes in [HCO3-] and SID induced in blood by PCO2 variations. In both populations, 82±12% of [HCO3-] change was due to SID variations, while only 18±12% to changes in ATOT dissociation. A significant correlation between hematocrit and ∆SID was observed in the whole study population (Fig. 2).

**Conclusions:** The β of ICU patients was lower, likely due to reduced albumin and hemoglobin concentrations. Similar PCO2 increases caused therefore greater pH variations in this population. Electrolyte shifts, likely deriving from red blood cells [3], were the major buffer system in our in-vitro model of acute respiratory acidosis.

**References**

1. Staempfli HR et al. J Appl Physiol 95:620-630, 2003

2. Van Slyke DD J Biol Chem 52:525-570, 1922

3. Langer T et al. J Crit Care 30(1):2-6, 2015

**Fig. 1 (abstract P304).**
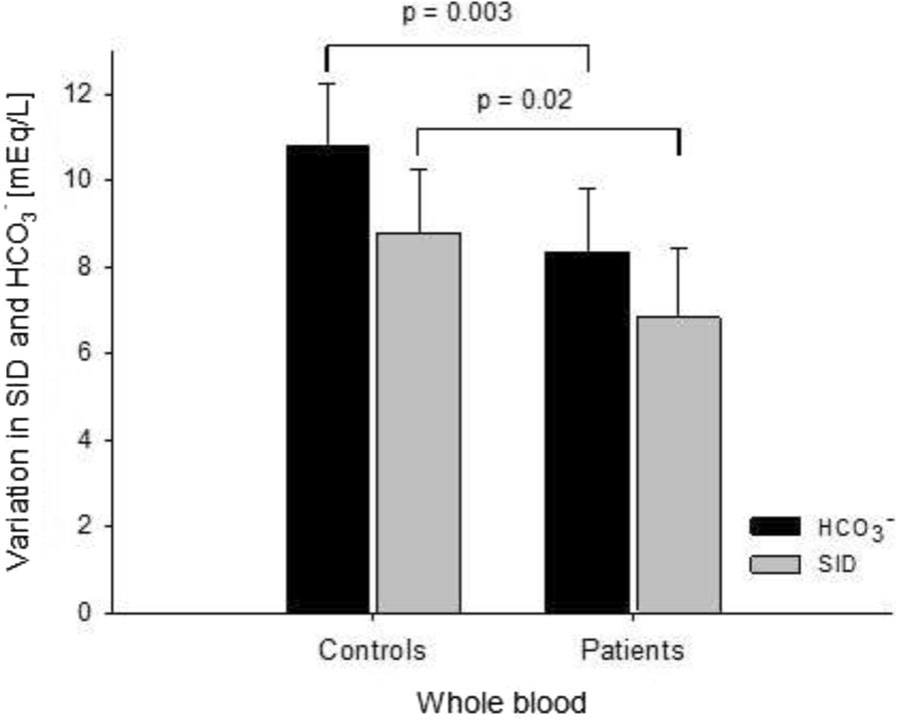
Changes in SID and bicarbonate concentration induced in whole blood by acute in vitro PCO2 variations

**Fig. 2 (abstract P304).**
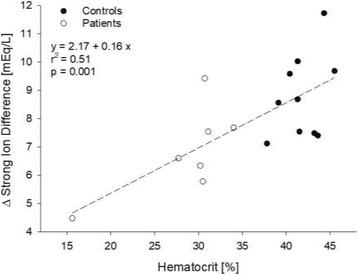
Regression between hematocrit and SID variation observed in the whole study population

## P305 Impact of electrolyte imbalance on prognosis in patients with aneurysmal subarachnoid haemorrhage: a retrospective study

### CWY Tam, HP Shum

#### Pamela Youde Nethersole Eastern Hospital, Hong Kong, China

**Introduction:** There is an increasing trend in the incidence of aneurysmal subarachnoid haemorrhage in Hong Kong and the disease carries high morbidity and mortality rate. Electrolyte disturbance is one of the known complications of SAH and the outcomes associated with this are not fully understood. The objective of this retrospective local study is to evaluate the pattern of electrolyte disturbances in patients with SAH and their impact on the prognostic functional outcome.

**Methods:** Patients with spontaneous aneurysmal SAH who were admitted to ICU at Pamela Youde Nethersole Eastern Hospital, Hong Kong between 1st January 2011 and 31st December 2016 were included into this retrospective local study. Collected data include demographic details, comorbidities, serum electrolyte levels (sodium and potassium) from day 1 to 11 of admission into ICU, radiographic intensity of haemorrhage using Fisher scale and the clinical grading of SAH using WFNS. Prognosis of these patients was estimated using the Glasgow Outcome Scale at 3 months after initial insult (Fig. 1).

**Results:** A total of 244 patients were included in this study. The mean age was 58, with the majority of patients being female (63.6%). The most common aneurysm location was in anterior communicating artery, though poor outcomes were shown significant in patients with posterior circulation aneurysms. Whilst early-onset hyponatremia was not correlated with poor outcome, late-onset hyponatremia was associated with better outcome. Logistic regression analysis identified 9 independent predictors of poor outcome (Table 1). Patients who underwent interventional radiological procedure treatment was shown to have better outcome.

**Conclusions:** Hypernatremia after SAH is associated with poor outcome. There does not appear to be significant evidence that hyponatremia has an effect on short-term mortality or certain outcome measures such as GOS, and its longer-term effects are not well characterized.

**Table 1 (abstract P305).** Logistic regression analysis to identify independent predictors for poor outcome (3 month GOS 1-3)

Parameters	Odds ratio	95% confidence interval	P-value
Age>55 years old	5.730	2.346-13.993	<0.001
APACHE IV score>50	4.314	1.752-10.622	0.001
WFNS grade>3	2.747	1.067-7.072	0.036
Fisher grade>2	2.878	1.125-7.361	0.027
Presence of ICH/IVH	4.055	1.603-10.259	0.003
Use of mannitol	4.318	1.027-18.159	0.046
Use of loop diuretic	6.022	1.994-18.189	0.001
Aneurysm involving posterior circulation	3.882	1.435-10.499	0.008
Day1-11 Sodium>146mmol/L	3.003	1.166-7.732	0.023
Received IR procedures	0.276	0.114-0.669	0.004

**Fig. 1 (abstract P305).**
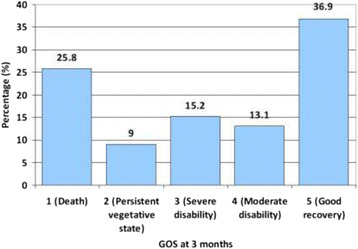
GOS at 3 months

## P306 Effect of hyponatraemia recorded within the first 24 hours on length of stay

### A McManus, P Morgan, A Myers, S Ali, T Samuels

#### Surrey and Sussex Healthcare NHS Trust, Redhill, UK

**Introduction:** We investigated the effect of hyponatraemia (defined as a sodium level < 135 mmol/l) recorded within the first 24 hours post admission on the overall length of stay (LOS; days) in our critical care unit.

**Methods:** We retrospectively analysed complete data for 1644 patients collected over a 3-year period. We performed an unpaired Wilcoxon rank sum test to compare the LOS between the two groups. Analyses were run using the open source statistical program R Version 3.4.2 (R Foundation for Statistical Computing, Vienna, Austria).

**Results:** The hyponatraemia group consisted of 544 patients with mean age 66.0 (SD 16.0) years, mean sodium 129.6 (SD 5.1) mmol/l and median LOS 4.1 [IQR 2.4 – 8.4] days. The normal sodium level (defined 135-145 mmol/l) group consisted of 1100 patients with mean age 64.3 (SD 18.5), mean sodium 139.1 (SD 2.3) mmol/l and median LOS 3.8 [IQR 1.9 – 7.0] days (Fig. 1 – note logarithmic transformation of LOS data). We found a statistically significant difference between the two groups when comparing the length of stay (p < 0.001).

**Conclusions:** Dean et al demonstrated no significant difference in the mean length of stay using the same definitions of hypo and eunatraemia as in this study [1]. Even though our data appears to contradict their findings, regarding the statistical significance seen, we feel that this is not significant clinically, given the very similar median times for LOS between the two groups; the unbalanced design may contribute to the statistical significance.

**Reference**

1. Dean P et al. Crit Care 17:443, 2013

**Fig. 1 (abstract P306).**
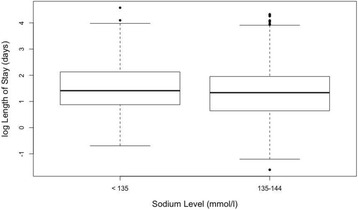
Length of stay between the two groups (note logarithmic scale for LOS)

## P307 Effect of hypernatraemia recorded within the first 24 hours on length of stay

### A McManus, T Samuels, A Myers, P Morgan

#### East Surrey Hospital, Redhill, UK

**Introduction:** We investigated the effect of hypernatraemia (defined as a sodium level > 145 mmol/l) recorded within the first 24 hours of admission on the overall length of stay (LOS; days) in our critical care unit.

**Methods:** We retrospectively analysed data for 1666 patients collected over a 3-year period. We used an unpaired Wilcoxon ranked sum test to compare the LOS between the two groups.

**Results:** The hypernatraemia group consisted of 189 patients with mean age 64.3 (SD 19.0) years, mean sodium 149.9 (SD 5.6) mmol/l and median LOS 4.0 (IQR 1.9 – 8.4) days. The eunatraemia (defined 135-145 mmol/l) group consisted of 1477 patients with mean age 64.3 (SD 17.8) years and mean sodium 139.8 (SD 2.8) mmol/l with a median LOS of 3.8 (IQR 1.9 – 7.1) days. We found no statistically significant difference (p = 0.0636) between the two groups when comparing the length of stay (Fig. 1).

**Conclusions:** Darmon et al demonstrated prognostic consequences of an admission sodium greater than 145, eliciting hypernatraemia as a factor independently associated with 30-day mortality [1]. In contrast, our study suggests that hypernatraemia (as defined) is not associated with the length of stay, however this result is limited by the unbalanced design of this small study.

**Reference**

1. Darmon M et al. Crit Care 17(1):R12, 2013

**Fig. 1 (abstract P307).**
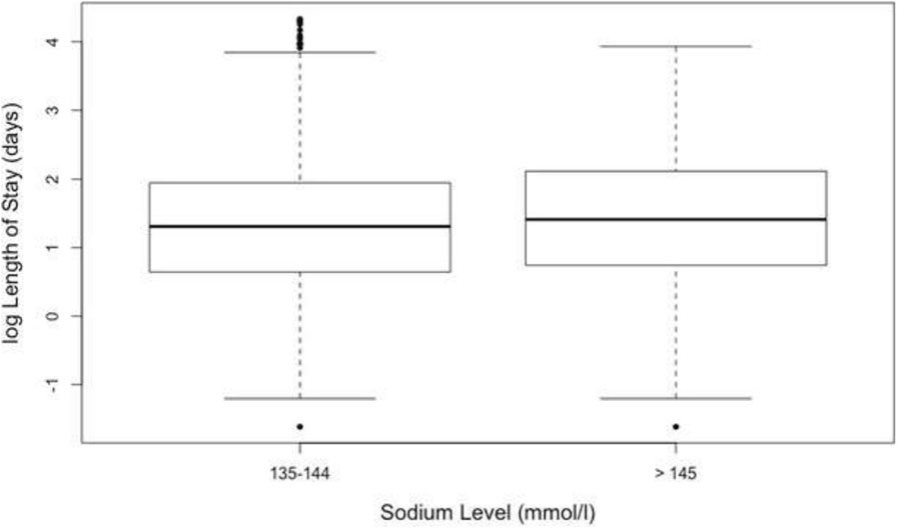
See text for description

## P308 Auscultation for bowel sounds in patients with ileus: an outdated practice in the ICU?

### SHW Van Bree^1^, NP Juffermans^2^

#### ^1^OLVG, Amsterdam, Netherlands,^2^AMC, Amsterdam, Netherlands

**Introduction:** Our aim is to determine whether auscultation for bowel sounds helps in clinical decision making in ICU patients with ileus.

Ileus can be the consequence of an operation, a side effect of drugs or the result of an obstruction requiring direct operative correction. Although auscultation for bowel sounds is routinely performed in the ICU and a well-established part of the physical examination in patients with suspected ileus, its clinical value remains largely unstudied.

**Methods:** A literature search of PubMed, Embase and Cochrane was performed to study the diagnostic value of auscultation for bowel sounds.

**Results:** Auditory characteristics (tinkling, high pitched and rushes) were highly variable in postoperative ileus, mechanical ileus and healthy volunteers. The inter-observer variability for the assessment of the quantity, volume and pitch of bowel sounds was high, with a moderate inter-observer agreement for discerning postoperative ileus, bowel obstruction and normal bowel sounds (kappa value 0.57). The intra-observer reliability of duplicated recordings for distinguishing between patients with normal bowels, obstructed bowels or postoperative ileus was 54% [1]. No clear relation between bowel sounds and intestinal transit was found (Table 1). Sensitivity and positive predictive value were low: respectively 32% and 23% in healthy volunteers, 22% and 28% in obstructive ileus, and 22% and 44% in postoperative ileus (Table 2).

**Conclusions:** Auscultation with the aim to differentiate normal from pathological bowel sounds is not useful in clinical practice. The low sensitivity and low positive predictive value together with a poor inter- and intra-observer agreement demonstrate the inaccuracy of utilizing bowel sounds for clinical decision-making. Given the lack of evidence and standardization of auscultation, the critically ill patient is more likely to benefit from abdominal imaging.

**Reference**

1. Felder S et al. J Surg Educ. 71(5):768-773, 2014.

**Table 1 (abstract P308).** Auscultation for the presence of audible peristalsis in relation with intestinal transit

Postoperative day 2	Auscultation of bowel sounds		n =
	No	Yes	
GC = 0 (paralytic ileus)	1	6	7
GC < 2 (no recovery of colonic transit)	0	41	41
GC <= 2 (recovery of colonic transit)	1	11	12

**Table 2 (abstract P308).** Accuracy and observer variability in bowel obstruction, ileus and healthy volunteers

Author	Design	Patients	Outcome	Results (%)	Limitations
Gu, Dig Surg, 2010	Prospective	A: healthy volunteers	Correct classification of bowel sounds	A: 78/54	Electronic recordings
		B: intestinal obstruction	Sensitivity/PPV	B: 42/72	Patient characteristics unknown
		C: postoperative ileus		C: 84/93	Small selection of bowel recordings
Felder, J Surg Education, 2014	Prospective	A: healthy volunteers	Correct classification of bowel sounds	A: 32/23/59	Electronic recordings
		B: intestinal obstruction	& reliability	B: 22/28/52	
		C: postoperative ileus	Sensitivity/PPV /intra-observer reliability	C: 22/44/53	
Breum, World J Gastroenterol., 2015	Prospective	Patient clinically suspected of bowel obstruction	Sensitivity, specificity, PPV and NPV	Sens.: 42; Spec.: 78	Shorter duration of electronic recordings::
		A: intestinal obstruction (n=37)	pathological bowel sounds	PPV: 48; NPV: 76	25 seconds
		B: without obstruction (n=61)	intestinal obstruction & inter-observer agreement	kappa-value: 0.29 (low)	

## P309 Stress ulcer prophylaxis in critically ill patients; when to stop?

### Y Kato, H Uchino, T Kaihara, T Fukuoka

#### Kurashiki Central Hospital, Kurashiki Okayama, Japan

**Introduction:** Stress ulcer prophylaxis has become a standard of care in Intensive Care Unit (ICU). However, it has been proposed that enteral nutrition (EN) could play preventive role for gastrointestinal bleeding and some studies revealed no added benefit of acid suppressive drugs to patients on EN. Based on these backgrounds, we use proton pump inhibitor (PPI) as stress ulcer prophylaxis during starvation period, and discontinue it within 24 hours after commencing meals or EN. The aim of this study is to evaluate the applicability of our protocol by reviewing the incidence of upper gastrointestinal bleeding (UGIB) in our ICU.

**Methods:** We conducted a retrospective observational study. All consecutive patients admitted to our ICU between April 2016 and March 2017 were reviewed. Patients who had UGIB within 24 hours after admission, had previous total gastrectomy, or underwent upper gastrointestinal surgery were excluded. The primary outcome was the incidence of overt or clinically important UGIB, and the secondary outcome was protocol adherence. We presented descriptive data as number (percentage) and median (interquartile range).

**Results:** A total of 521 patients were included. Of those, 315 (60.5%) were male, median age was 73 (57-81), and median SOFA score was 5 (2-8). Of all 521 patients, 16 (3.37%) had overt bleeding, and 2 (0.38%) had clinically important bleeding. Both 2 patients who developed clinically important bleeding had respiratory failure and coagulopathy which had been identified as risk factors for UGIB from previous studies. Three hundred sixty two patients had commenced meals or EN during their ICU stay, and median duration of starvation period was 25.5 (17-41) hours. Among these 362 patients, 264 patients discontinued PPI within 24 hours after initiation of feeding resulting in 73.4% for the protocol adherence.

**Conclusions:** Termination of PPI within 24 hours after commencing nutrition can be feasible management due to its low incidence of clinically important UGIB event.

## P310 Much ado about cutting: an audit of care pathway and outcomes in patients undergoing emergency laparotomy

### Z Whitman, M Charlton, J Parker, N Flint

#### Leicester Royal Infirmary, Leicester, UK

**Introduction:** Patients requiring operative procedures admitted under non-surgical specialties typically experience delays in treatment and fail to meet peri-operative standards with regards to the timing of operative intervention. Patients admitted from medicine requiring an emergency laparotomy have an increased mortality when compared to those patients admitted from surgery (20.4% v 13.6%) [1].

**Methods:** We undertook a retrospective case note review of patients requiring a non-elective laparotomy at our hospital during a six-month period in 2016. Patients were identified using the emergency theatre booking system. Data were gathered on admission details, peri-operative care and post-operative stay.

**Results:** Two main investigators reviewed 104 patients to standardise data extraction. Six patients presenting with inflammatory bowel disease were excluded from analysis. Most patients (59.1%) were admitted through the Emergency Department; 17 (29.3%) of whom were initially admitted under medicine, with only 37.5% of these reviewed by a senior clinician prior to admission (Table 1). There was no statistically significant difference in mortality between the medicine and surgery groups. There was a trend to increased length of stay in ICU and in hospital in the medical group (Table 2).

**Conclusions:** Lack of senior decision making may have a direct impact on patient care due to the inappropriate streaming of patients to medicine. The increased mean length of stay in those patients admitted to medicine may reflect a delay in surgical intervention and therefore a prolonged recovery period. We are introducing an Acute Abdominal Pain Screening and Immediate Action Tool to improve identification of these high-risk patients and early involvement of senior decision makers.

**Reference**

1. Saunders DI et al. Br J Anaesth 109(3):368-375, 2012

**Table 1 (abstract P310).** Presence of a senior review in ED for those patients admitted to medicine and surgery

	Senior review?		
	Yes	No	Total
Admitted to surgery	18 (50%)	18 (50%)	36
Admitted to medicine	6 (37.5%)	10 (62.5%)	16

**Table 2 (abstract P310).** Length of stay (LOS) data in days for patients initially admitted to medicine and surgery

Length of Stay		Mean	Median
	ICU	8.2	3
Medicine	Hospital	24.9	12
	Post-operative	18.5	8
	ICU	3.6	3
Surgery	Hospital	15.0	11
	Post-operative	13.8	8

## P311 Injury caused by colorectal surgery: the role of proteins S100A

### J Maca, P Ihnat, M Peteja, O Jor, P Reimer, P Sevcik

#### University Hospital od Ostrava, Ostrava, Czech Republic

**Introduction:** Biomarkers reflecting the extent of surgical tissue trauma should be investigated in an effort to predict and prevent postoperative complications. The aim of the present study was to investigate blood concentrations of selected alarmins in patients after colorectal surgery in comparison to healthy individuals. The secondary aim was to analyze the relationship between alarmins and inflammatory biomarkers during early postoperative period.

**Methods:** The prospective, single-center, observational study consisted of non-surgical (NS) group (n=35) and surgical (S) group (n=38) undergoing colorectal surgery. Serum levels of selected alarmins (S100A8 and S100A12) and inflammatory biomarkers (leukocytes; C-reactive protein, CRP; interleukin-6, IL-6) were analyzed.

**Results:** Proteins S100A8 an S100A12 had significantly higher serum values in the S-group during all three days after the surgery. The multidimensional model taking into account age, sex, weight, group and days revealed significant differences between study groups for both proteins S100A8 and S100A12 (p<0.001, p=0.001, respectively). Biomarkers (leukocytes, CRP, and IL-6) showed significant differences between study subgroups (p<0.001, p<0.001, and p<0.001, respectively). In S-group, moderate positive correlations were found between S100A8 and all biomarkers: leukocytes (r=0.6), CRP (r=0.5), and IL-6 (r=0.6). S100A12 had moderate positive correlation with leukocytes (r=0.5). Levels of S100A8 also positively correlated with intensive care unit and hospital length of stay (r=0.6, r=0.5, respectively)

**Conclusions:** Protein S100A8 might be considered as early biomarker of first wave of immune activation elicited by surgical injury after colorectal surgery. The increase of the alarmins is reflected by the elevation of routine inflammatory biomarkers.

## P312 Effect of institutional case-volume on in-hospital mortality after living donor liver transplantation: analysis of 7440 cases between 2007 and 2016 in Korea

### L Lim^1^, S Yoo^1^, E Jang^2^, G Kim^3^, H Ryu^1^

#### ^1^Seoul National University College of Medicine, Seoul, South Korea,^2^Department of Information Statistics, Andong National University, Andong, South Korea,^3^Department of Statistics, Kyungpook National University, Daegu, South Korea

**Introduction:** The positive effect of case volume on patient outcome seen in complex surgical procedures such as coronary artery bypass graft surgery has not been definitively shown in liver transplantation (LT) with conflicting reports.

**Methods:** The Health Insurance Review and Assessment Service (HIRA) data from 2007 to 2016 was analyzed for in-hospital mortality, ICU length of stay, and hospital length of stay in patients undergoing LT, depending on the case volume of the institution.

**Results:** The operative mortality of 10993 LTs was 6.9% (690/10993). The operative mortality in institutions performing more than 50 cases/year was 5.1% (444/8668) as compared to 8.9% (118/1322) in institutions performing 10-49 cases/year and 12.8% (128/1003) in institutions performing less than 10 cases/year. After adjusting for disease severity using the Elixhauser index, risk factors for in-hospital mortality after LT included female sex (OR 1.38, 95%CI [1.17-1.63], p=0.0001), age over 60 (OR 1.31, 95%CI [0.73-2.36], p=0.046), and institutions with less than 10 cases/year (OR 2.86, 95%CI [2.32-3.54], p<0.0001). The overall ICU length of stay was 9.5±13.0 days. The ICU length of stay in institutions performing more than 50 cases/year was 9.1±13.4 days as compared to 10.7±12.2 days in institutions performing 10-49 cases/year and 11.6±9.7 days in institutions performing less than 10 cases/year. The overall hospital length of stay was 48.3±33.3 days. The ICU length of stay in institutions performing more than 50 cases/year was 47.5±32.7 days as compared to 50.4±35.9 days in institutions performing 10-49 cases/year and 51.9±34.9 days in institutions performing less than 10 cases/year. The overall hospital cost covered by the national insurance was $73,263±39,177 per liver transplant.

**Conclusions:** Our study results showed that LTs performed at institutions with higher case volume were associated with lower mortality and shorter ICU and hospital length of stay.

## P313 Outcome after a liver transplantation surgery from infected deceased donors

### N Fioravanti Postalli, T Kawagoe Alvarisa, P Travassos, R Vale, V Cordeiro veiga, A Ibrahim David, J Padilla, A Bernal Filho, A Gustavo Pereira, G Peron Junior, C Gritti, S Soriano Ordinola Rojas

#### Hospital BP - A Beneficência Portuguesa de São Paulo, Sao Paulo, Brazil

**Introduction:** Liver transplantation is the definitive treatment in end-stage liver cirrhosis with increased survival in the early years. There are several complications, but infection is the major cause of morbidity and mortality after transplantation. The objective was evaluate the outcome of patients in the postoperative period of liver transplantation, in which the donors were in the presence of infection.

**Methods:** Were analyzed all liver transplants performed in a large hospital in the period from 2014 to the beginning of 2017, which presented donor infection and evaluated: identification of the microorganism, antibiotic therapy and outcome (survival) in 30 days.

**Results:** From 2014 to early 2017, 71 patients underwent liver transplantation. Of these, 07 (10%) received organs from donors in the presence of infection. The infectious agents identified in donors varied between Acinetobacter baumannii, Enterobacter cloacae, Klebsiella pneumoniae Carbapenemase, VDRL and toxoplasmosis (IGG and IGM) tests positive, and anti-HBC positive serology. Therefore, bloodstream infection was present in all donors. The protocol of the institution was followed being collected cultures of the recipient and maintained antibiotic in the postoperative period. No deaths were attributed to the infection.

**Conclusions:** Postoperative care, associated with the continuity of antibiotic therapy or the early initiation of treatment of infection, as well, as care in the prevention of new infections, are primary procedures for a significant reduction in mortality in liver transplantation.

## P314 Prevalence and prognostic value of abnormal liver test results in critically ill children and the impact of nutrition hereon

### R Haghedooren^1^, M Jenniskens^1^, M Jenniskens^1^, F Guïza ^1^, S Verbruggen^2^, G Guerra^3^, K Joosten^2^, L Langouche^1^, G Van den Berghe^1^

#### ^1^KU Leuven, Leuven, Belgium,^2^Erasmus-MC Sophia Childern’s Hospital, Rotterdam, Netherlands,^3^University of Alberta, Stollery Children’s Hospital, Edmonton, Canada

**Introduction:** Critical illness-induced liver test abnormalities are associated with complications and death in adult ICU patients, but remain poorly characterized in the pediatric ICU (PICU). In the PEPaNIC RCT, delaying initiation of parenteral nutrition to beyond day 7 (late PN) was clinically superior to providing PN within 24h (early PN), but resulted in a higher rise in bilirubin. We aimed to document prevalence and prognostic value of abnormal liver tests and the impact of withholding early PN in the PICU.

**Methods:** We performed a preplanned secondary analysis of 1231 of the 1440 PEPaNIC patients aged 28 days to 17 years, as neonatal jaundice was considered a confounder. Plasma concentrations of total bilirubin, ALT, AST, γ GT, ALP were measured systematically during PICU stay. Analyses were adjusted for baseline characteristics including severity of illness.

**Results:** During the first 7 PICU days, the prevalence of cholestasis (>2mg/dl bilirubin) ranged between 3.8%-4.9% and of hypoxic hepatitis (>=20-fold ULN for ALT and AST) between 0.8%-2.2%, both unaffected by the use of PN. Throughout the first week in PICU plasma bilirubin concentrations were higher in late PN patients (p<0.05), but became comparable to early PN patients as soon as PN was started on day 8. Plasma concentrations of γ GT, ALP, ALT and AST were unaffected by PN. High day 1 plasma concentrations of γ GT, ALT and AST (p<=0.01), but not ALP, were independent risk factors for PICU mortality. Day 1 plasma bilirubin concentrations displayed a U-shaped association with PICU mortality, with higher mortality associated with bilirubin concentrations <0.20mg/dl and >0.76mg/dl (p<=0.01).

**Conclusions:** In conclusion, overt cholestasis and hypoxic hepatitis were rare and unrelated to nutritional strategy. However, accepting a large macronutrient deficit during week 1 increased plasma bilirubin. A mild elevation of bilirubin on the first PICU-day was associated with lower risk of death and may represent an adaptive stress response rather than true cholestasis.

## P315 Positive fluid balance is an independent risk factor for intensive care unit mortality in patients with acute-on-chronic liver failure

### F Sousa Cardoso^1^, A Laranjo^1^, V Gamelas^1^, R Pereira^1^, L Bagulho^1^, P Fidalgo^1^, C Karvellas^2^

#### ^1^Curry Cabral Hospital, Lisbon, Portugal,^2^University of Alberta Hospital, Edmonton, Canada

**Introduction:** Patients with acute-on-chronic liver failure (ACLF) have high mortality rates. We sought to evaluate the impact of fluid balance in ICU mortality for these patients.

**Methods:** Retrospective analysis from a prospective database including 84 consecutive patients with ACLF admitted to the Curry Cabral Hospital (Lisbon, Portugal) ICU from 04/2013 to 03/2017. The association of fluid balance during ICU stay (until discharge, liver transplant (LT), or death) with ICU mortality was studied using logistic regression following bootstrapping (1000 samples).

**Results:** Median (IQR) age was 59 years and 71 (85%) patients were male. Most frequent precipitant events were infection and bleeding, in 43 (51%) and 23 (27%) patients, respectively. Ascites was present in 71 (85%) patients. On ICU admission, 64 (76%) patients had at least 2 organ failures (based on CLIF-SOFA score). Median (IQR) APACHEII and MELD scores were 21 (17:24) and 27 (20:33), respectively. Invasive mechanical ventilation, vasopressors, and renal replacement therapy (RRT) were required for 32 (38%), 45 (54%), and 17 (20%) patients, respectively. During ICU stay 42 (50%) patients died (5 following LT). Median (IQR) fluid balance per day in ICU was +0.5 (-0.2:+1.1) liters. This was significantly associated with ICU mortality (adjusted OR = 1.61 per each liter increment), following adjustment for INR and need for vasopressors and RRT (AUC = 0.78) (Fig. 1, Table 1).

**Conclusions:** For patients with ACLF, positive fluid balance was associated with worse ICU mortality. Frequent bedside hemodynamic assessment and a judicious use of intravenous fluids might help to improve these patients’ outcomes.

**Table 1 (abstract P315).** Unadjusted and adjusted analysis of the association of fluid balance per day in the intensive care unit (liter) with intensive care unit mortality

	OR	P	Adjusted OR	P
Fluid balance per day in ICU (liter)	1.52 (1.03-2.26)	0.037	1.61 (1.06-2.44)	0.030
INR			2.60 (1.20-5.62)	0.016
Vasopressors			0.97 (0.36-2.63)	0.96
Renal replacement therapy			5.16 (1.38-19.3)	0.008
Model: n = 84; AUC (95%CI) = 0.78 (0.67-0.88)				

**Fig. 1 (abstract P315).**
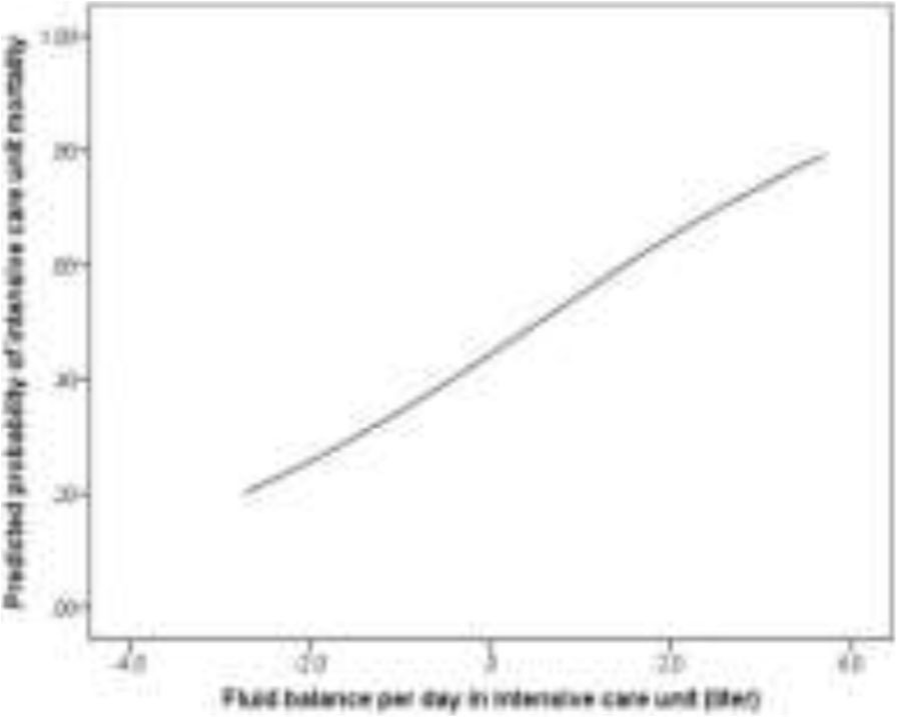
Unadjusted predicted probability of intensive care unit mortality based on fluid balance per day in the intensive care unit (liter)

## P316 The psoas muscle in critically ill patients with acute pancreatitis

### P Turton^1^, R Hay^1^, A Sud^2^, R Sutton^2^, I Welters^2^

#### ^1^Royal Liverpool University Hospital, Liverpool, UK,^2^University of Liverpool, Liverpool, UK

**Introduction:** Muscle wasting is a common consequence of disuse and inflammation during admission to intensive care with critical illness. Limb muscles are known to decrease in size during critical illness, but less is known about muscles of the trunk. In this study, we tracked how psoas muscle area changes at multiple levels, in a group of patients with acute severe pancreatitis.

**Methods:** Paired computed tomography (CT) scans were obtained from 21 patients admitted to the Royal Liverpool University Hospital’s ICU with acute severe pancreatitis. The first scan was within 3 days of admission, and the second took place between 8 to 16 days later. For each scan, three slices were identified: the top and bottom plates of L4, and the mid-point of L4 vertebral body. On each slice, the cross sectional area (CSA) of the left and right psoas muscle was calculated using ImageJ. The difference and percentage change in CSA between both scans was calculated. White cell counts and C-reactive protein results were obtained, with peak levels correlated against change in muscle size.

**Results:** Combined CSA of the left and right psoas muscle increased from top to bottom plates and was positively correlated with height (r=0.74, p<0.001 mid L4 level)) and weight (r=0.57, p=0.014, mid L4 level) at all three levels. At all three levels, there were significant losses of CSA between the two scans (see Table 1). CRP was moderately correlated with percentage change in CSA (r= -0.55, p=0.014). Increasing weight on admission was associated with greater percentage losses in CSA (r= -0.78, p<0.001). WCC did not correlate with change in size.

**Conclusions:** In critically ill patients with acute severe pancreatitis, there are significant losses in both psoas muscles throughout the L4 level. Further prospective studies are required to determine if inflammatory markers and cytokines have a role in these losses, and to determine the functional effects of these losses.

**Table 1 (abstract P316).** Percentage change in psoas muscle cross sectional area at three different levels

Level	Mean change in CSA (%) [SD]	95% CI	P-value
Upper plate of L4	-13.98 [11.83]	-19.36 to -8.60	<0.001
Mid L4	-10.14 [6.07]	-13.07 to -7.21	<0.001
Lower plate of L4	-11.21 [5.70]	-13.96 to -8.46	<0.001

## P317 Penta-therapy for severe acute hyperlipidemic pancreatitis: a retrospective chart review over a 10-year period

### J Lu, Y Xie, J Du, M Kang, W Jin, H Xie, R Cheng, R Tian, R Wang

#### Shanghai General Hospital, Shanghai, China

**Introduction:** The evidence for penta-therapy for hyperlipidemic severe acute pancreatitis (HL-SAP) is anecdotal. The purpose of our study is to evaluate the efficacy of penta-therapy for HL-SAP in a retrospective study.

**Methods:** Retrospective study between January 2007 and December 2016 in a hospital intensive care unit.HL-SAP patients were assigned to conventional treatment alone (the control group) or conventional treatment with the experimental protocol (the penta-therapy group) consists of blood purification, antihyperlipidemic agents, low-molecular-weight heparin, insulin, covering the whole abdomen with Pixiao (a traditional Chinese medicine).Serum triglyceride, serum calcium, APACHE II score, SOFA score, Ranson score, CT severity index, and other serum biomarkers were evaluated. The hospital length of stay, local complications, systematic complications, rate of recurrence, overall mortality, and operation rate were considered clinical outcomes.

**Results:** 63 HL-SAP patients received conventional treatment alone (the control group) and 25 patients underwent penta- therapy combined with conventional treatment (the penta-therapy group). Serum amylase, serum triglyceride, white blood cell count, C -reactive protein, and blood sugar were significantly reduced, while serum calcium was significantly increased with penta-therapy. The changes in serum amylase, serum calcium were significantly different between the penta-therapy and control group on 7th day after the initiation of treatment. The reduction in serum triglyceride in the penta-therapy group on the second day and 7th day were greater than the control group. Patients in the penta-therapy group had a significantly shorter length of hospital stay.

**Conclusions:** This study suggests that the addition of penta-therapy to conventional treatment for HL-SAP may be superior to conventional treatment alone for improvement of serum biomarkers and clinical outcomes.

## P318 Energy requirements in patients with acute severe pancreatitis: comparison between indirect calorimetry and validated equations

### E Salciute^1^, J Liucvaikyte^2^, A Klimasauskas^2^, G Kekstas^2^, J Sipylaite^2^

#### ^1^Vilnius University Hospital Santaros clinics, Vilnius, Lithuania,^2^Faculty of Medicine, Vilnius University, Vilnius, Lithuania

**Introduction:** To compare the energy requirements of patients with acute severe pancreatitis measured by the indirect calorimetry and calculated with validated equations.

**Methods:** Prospective observational study with acute severe pancreatitis patients was conducted after the approval of the Regional Committee of Bioethics. Demographic data was collected. Measurements of indirect calorimetry (IC) were obtained and compared to 11 equations: 1) Harris-Benedict; 2) Penn State; 3) Faisy; 4) Swinamer; 5) Ireton – Jones; 6) Mifflin – St. Joer; 7) Mifflin × 1.25; 8) Harris Benedict × 1.25; 9) Harris Benedict adjusted for obesity; 10) ASPEN recommendation of 25 kcal/kg; 11) ASPEN recommendation of 30 kcal/kg.

**Results:** 22 patients were included in the study and 371 IC measurements were compared to the results of the validated equations. The highest discrepancy of energy requirement was observed between IC and Harris-Benedict equation adj. for obesity, 2359 ± 469 and 1749 ± 176 kcal, respectively (median ± IQR, p < 0.01). ASPEN recommendation of 25 kcal/kg with 2375 ± 625 (median ± IQR) kcal was the closest to the measured energy expenditure, the difference being non-significant (p = 0.62) [Fig. 1]. However, the correlation coefficient between IC measurements and ASPEN 25 kcal/kg estimation was only 0.212 (p < 0.01) while Faisy equation with daily changing variables (such as temperature and minute ventilation) resulted in correlation coefficient of 0.538 (p < 0.01) [Fig. 2].

**Conclusions:** In critically ill patients with severe acute pancreatitis, ASPEN recommendation of 25 kcal/kg resulted in the best estimation of median energy requirement compared with the indirect calorimetry measurements. However, adjusting daily nutritional targets would be best achieved with the dynamic Faisy equation.

**Fig. 1 (abstract P318).**
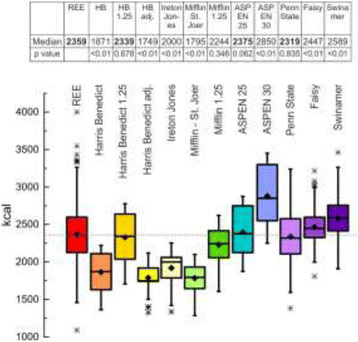
Comparison of median values and distribution of measured and calculated energy requirements

**Fig. 2 (abstract P318).**
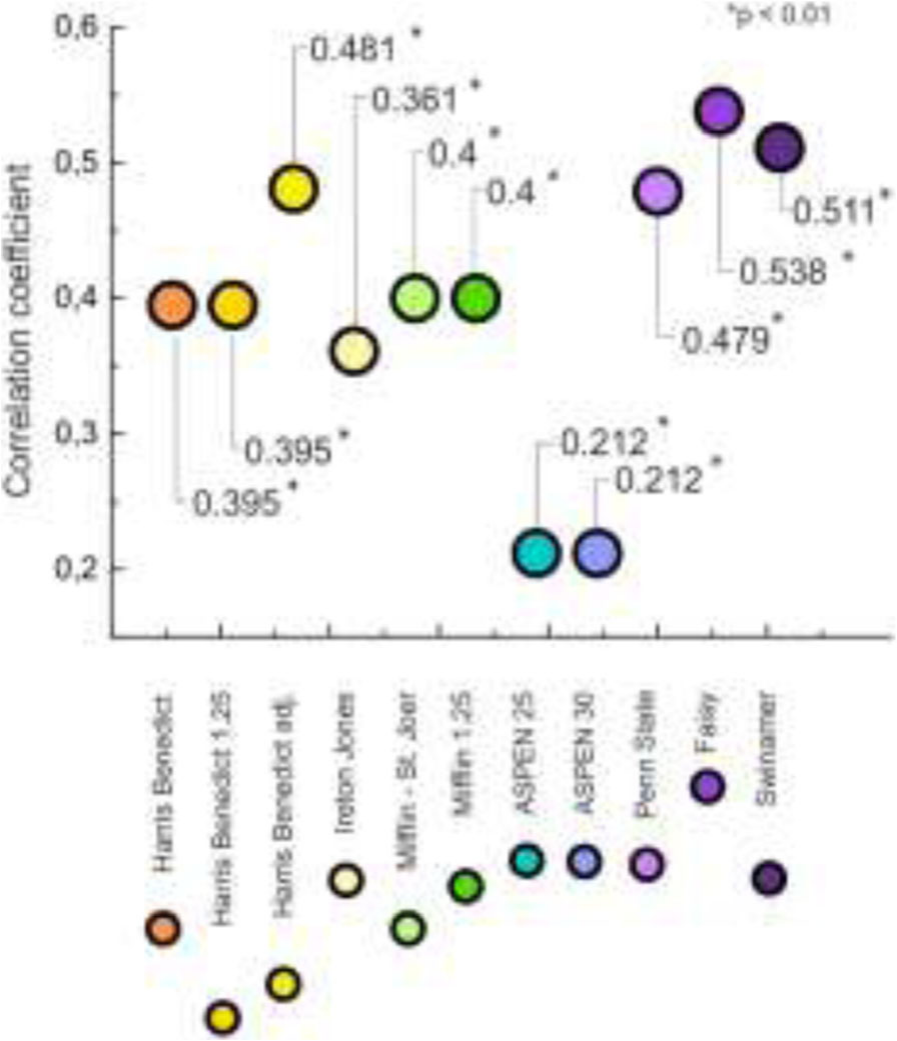
Correlation coefficients between measured and calculated energy requirements

## P319 Energy expenditure measured by indirect calorimetry and provision of energy and protein for patients with acute severe pancreatitis

### E Salciute^1^, J Liucvaikyte^2^, A Klimasauskas^2^, G Kekstas^2^, J Sipylaite^2^

#### ^1^Vilnius University Hospital Santaros clinics, Vilnius, Lithuania, ^2^Faculty of Medicine, Vilnius University, Vilnius, Lithuania

**Introduction:** To measure energy requirements of patients with acute severe pancreatitis using indirect calorimetry and investigate if the amount of received calories and protein are related to better outcomes.

**Methods:** Prospective observational study with acute severe pancreatitis patients was conducted after the approval of Regional Committee of Bioethics. Demographic, outcome data and clinical nutrition records were collected. Energy needs of patients were measured using indirect calorimetry (IC).

**Results:** 22 patients were enrolled in the study. 12 patients survived. Average energy expenditure (EE) for all patients was 26 ± 4 kcal/kg (mean ± SD). There was no difference in the average EE between the patients who survived and those who died: 27 ± 1 and 25 ± 1 kcal/kg (mean ± SD) respectively (p > 0.05). However, there was a negative correlation between EE and SAPS 3 score in the non-survivors group – correlation coefficient -0.679, p < 0.05. The energy deficit (computed by subtracting caloric intake from EE measurement) was similar among survivors and non-survivors, 5.5 ± 1 vs 6.5 ± 2 kcal/kg, respectively (mean ± SD) (p > 0.05). The patients who survived had received 21 ± 1 kcal/kg while those who died – 18 ± 1 kcal/kg (mean ± SD) (p > 0.05). The provision of protein was also similar for both groups: 0.9 ± 0.1 g/kg for survivors and 1 ± 0.04 g/kg for non-survivors (mean ± SD) (p > 0.05). There was no statistically significant correlation between provision of calories and protein and outcomes such as length of hospital and ICU stay or duration of mechanical ventilation.

**Conclusions:** Average energy expenditure in critically ill patients with acute severe pancreatitis roughly equals to ASPEN estimation of 25 kcal/kg and does not differ among survivors and non-survivors. Outcomes such as survival, length of hospital and ICU stay and duration of mechanical ventilation were unaffected by caloric nor protein provision in this sample.

## P320 Efficacy and safety of TAK-954 in critically ill patients with enteral feeding intolerance: a randomized phase 2a clinical trial

### M Chapman^1^, A Deane^1^, K Jones^1^, C Barnes^2^, D Nguyen^2^, C Almansa^3^

#### ^1^University of Adelaide, Adelaide, Australia,^2^Theravance Biopharma US, Inc., South San Francisco, California, USA,^3^Takeda Development Center Americas, Inc., Cambridge, Massachusetts, USA

**Introduction:** Disturbances in gastrointestinal motility are common in critically ill patients receiving enteral nutrition. Slow gastric emptying (GE) is the leading cause of enteral feeding intolerance (EFI), which compromises nutritional status and is associated with increased morbidity and mortality. This phase 2a study evaluated the efficacy, safety and tolerability of acute TAK-954 (previously TD-8954), a selective agonist of the 5 hydroxytryptamine receptor 4 (5HT4), compared with metoclopramide in critically ill patients with EFI.

**Methods:** This was a double-blinded, double-dummy study conducted in mechanically ventilated patients with EFI (>200 mL gastric residual volume) randomized to receive either intervention (TAK-954 0.5 mg over 1 hour and 0.9% saline 10 ml injection QID) or control (0.9% saline over 1 hour and metoclopramide 10 mg injection QID). Within 1 hour of the first dose, patients received a test meal of 100 mL Ensure® and GE was measured using scintigraphy. Primary objectives were to evaluate the safety and tolerability of TAK-954 and its effect on GE (% retention at 180 mins) vs control.

**Results:** A total of 13 patients (intervention, n = 7; control, n = 6) were studied. The median ages were 47 and 57 years in these groups, respectively. Post-treatment, a 2-fold greater number of patients had normal gastric retention (<13% at 180 mins) in the intervention group vs the control group (6 vs 3; Fig. 1). In the intervention and control groups, 5 and 4 patients experienced adverse events (AEs; 2 and 3 of which were serious), respectively (Table 1). No AEs led to treatment discontinuation.

**Conclusions:** A greater proportion of patients receiving TAK-954 had normal gastric retention after a single dose compared with those receiving metoclopramide. Treatment with TAK-954 was not associated with an increase in AEs. These results support further evaluation of TAK-954 in critically ill patients with EFI.

**Table 1 (abstract P320).** please see text description

	Intervention [TAK-954 0.5 mg; n = 7] n (%)	Control [n = 6; metoclopramide 10 mg] n (%)
Total number of AEs	8	5
Patients reporting at least 1 AEa	5 (71.4)	4 (66.7)
Any moderate or severe AEs	3 (42.9)b	3 (50.0)c
Any serious AEsd	2 (28.6)e	3 (50.0)f
AEs leading to discontinuation	0 (0)	0 (0)
Any AEs leading to drug interruption	0 (0)	0 (0)
Deaths	2 (28.6)	3 (50.0)

^a^Only one AE (diarrhea in the metoclopramide group) was considered to be potentially treatment-related

^b^One patient reported each of the following: cerebral hemorrhage, respiratory failure and decubitus ulcer

^c^One patient reported each of the following: hemmorhage intracranial, subarachnoid hemmorhage, disease progression, hypertension

^d^No serious AEs (including deaths) were considered to be treatment-related in either group

^e^One patient reported each of the following: cerebral hemorrhage and respiratory failure

^f^One patient reported each of the following: hemmorhage intracranial, subarachnoid haemorrhage and disease progression AE, adverse event

**Fig. 1 (abstract P320).**
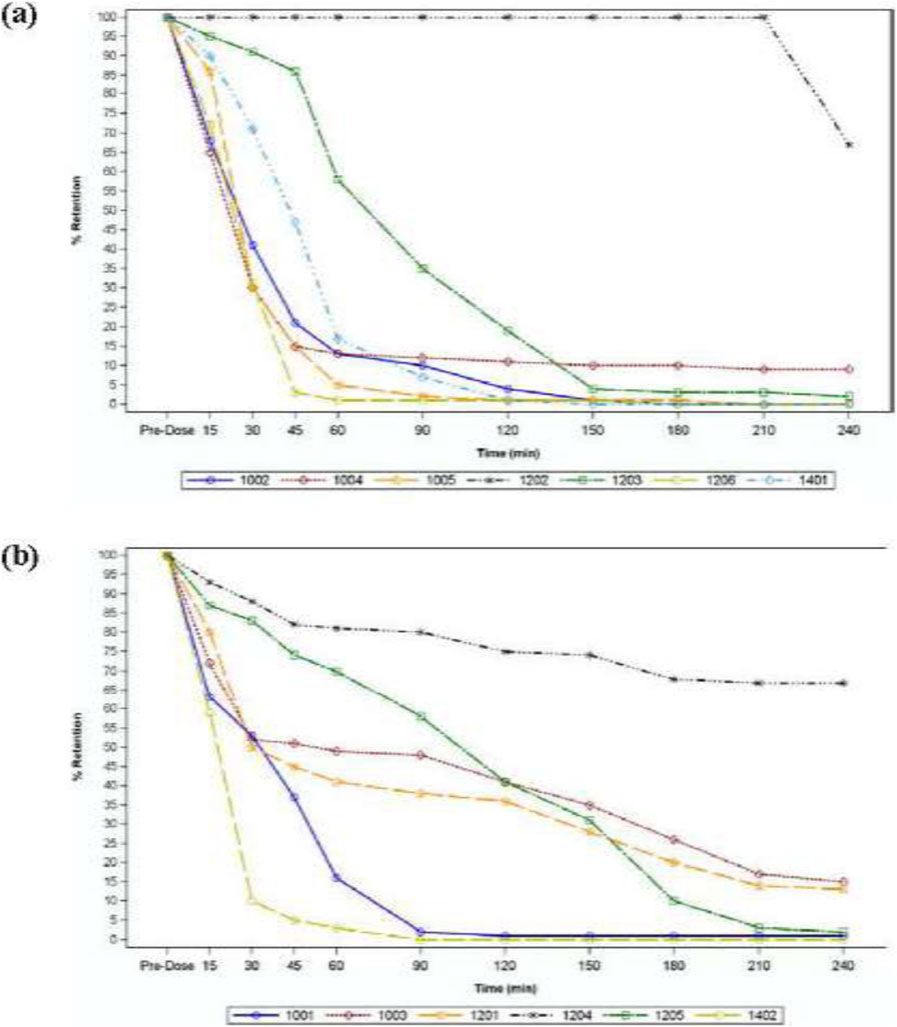
Grastric retention assessed by individual scintigraphy in the (a) intervention (TAK-954 0.5 mg; n=7) and (b) control (metoclopramide 10 mg; n = 6) treatment groups

## P321 Method to assess gastric emptying in the fed state in enterally tube fed patients: comparison of the paracetamol absorption test to scintigraphy

### J James^1^, W Doll^2^, S Harris^1^

#### ^1^Lyric Pharmaceuticals, Inc., South San Francisco, California, USA,^2^Scintipharma, Inc, Lexington, Kentucky, USA

**Introduction:** The paracetamol absorption test (PAT) is the most common and practical approach for assessing gastric emptying (GE) in critically ill patients. However, current methods require that paracetamol be administered to an empty stomach, removing gastric contents and depriving patients of feeding for several hours. The objective of this study was to develop methods to assess gastric emptying in these patients without interrupting feeding.

**Methods:** Gastric emptying was assessed in the fed state using PAT and scintigraphy in 12 healthy volunteers. Paracetamol 1g in 30mL was ingested immediately before consumption of a test meal of 250mL Ensure Plus containing 375kcal, 15.6g protein, and 12.3g fat plus 4mBq of 99mTc-DPTA as a scintigraphic agent. Comparisons were made between paracetamol absorption and the time to 25% and 50% gastric emptying by scintigraphy at baseline and after administration of ulimorelin 600μg/kg, a prokinetic agent known to enhance gastric emptying. Blood samples for paracetamol were collected for up to 4h post administration. Values for normal gastric emptying were based on the 95% confidence intervals for PK parameters. Sensitivity and specificity were assessed by receiver operating characteristic (ROC) analysis before and after treatment.

**Results:** The PAT correlated with scintigraphy and PK parameters for normal emptying were determined. Cmax and AUC2 were the most sensitive and specific parameters for assessing GE with lowest variability and areas under the ROC curve of 0.8981 and 0.8889, respectively. A 2h sampling period appeared sufficient to distinguish normal from abnormal emptying.

**Conclusions:** The PAT can be used to distinguish normal versus abnormal GE in the fed state. Under the conditions used, patients can receive up to 250mL enteral feeding over a 2h test period (125 mL/hr). This method can be used to distinguish normal from abnormal gastric emptying in enterally tube fed patients without interrupting feedings.

## P322 Association of energy adequacy with 28-day mortality in mechanically ventillated critically ill patients

### M Haliloglu^1^, B Bilgili^2^, I Sayan^2^, H Ozcelik^1^, A Kekecoglu^1^, I Cinel^2^

#### ^1^Yedikule Chest and Thoracic Surgery Training and Research Hospital, Istanbul, Turkey,^2^Marmara University, School of Medicine, Department of Anesthesiology and Reanimation, Istanbul, Turkey

**Introduction:** For mechanically ventillated critically ill patients, the effect of full feeding on mortality is stil controversial. We aimed to investigate the relationship of energy intakes with 28-day mortality, and nutritional risk status influenced this relationship.

**Methods:** This prospective observational study was conducted among adult patients admitted to ICU and required invasive mechanical ventilation (IMV) for more than 48 h. Data on baseline characteristics and the modified Nutritional Risk in Critically ill [mNUTRIC] score was collected on day 1. Energy intake and nutritional adequacy was recorded daily until death, discarge or until twelfth evaluable days. Patients were divided into 2 groups: a)received < 75% of prescribed energy b) received >= 75% of prescribed energy.

**Results:** 150 patients (65% male, mean age 51.0±15.3 years, mean body mass index 27.9±6.2 kg/m2, mean mNUTRICscore 5.8± 1.7) were included. In the univariate analysis, mNUTRİC score was associated with 28-day mortality. In the multivariable logistic regregression analysis, mNUTRIC score(Odds ratio, OR 1.65, CI 1.20-1.70, P < O.OO1) was associated with 28-day mortality. Nutritional adequacy was assessed, median nutritional adequacy was 0.40 (0.17-0.75). In patients with high mNUTRİC score (5-9), received >= 75% of prescribed energy was associated with a lower predicted 28-day mortality; this was not observed in patients with low mNUTRİC score (0-4).

**Conclusions:** Nearly 60 % of IMV required patients admitted to ICU were at nutritional risk, mNUTRİC score is associated with 28-day mortality. Energy adequacy of >= 75% of prescribed amounts were associated with decreased mortality in patients with a high mNUTRİC score.

## P323 Nutritional risk in elderly patients undergoing emergency surgery

### D Alampi, R Boninsegna

#### Sapienza University of Rome, Rome, Italy

**Introduction:** Malnutrition in elderly subjects can be an additional risk in emergency surgery.

**Methods:** We performed a study on patients in Intensive Care of Sant’Andrea Hospital, Rome, Italy. 56 patients (33M/23F, mean age 85.6 years, BMI 25.39) undergoing emergency surgery were recruited in the period from May 2016 to July 2017. ASA, SOFA, body mass index, preoperative albumin and lymphocyte count were recorded. All patients were subjected to enteral nutrition. We analyzed need for mechanical ventilation, surgical site infection, multiorgan failure (MOF), length of stay in ICU. Statistical data analysis studied correlation between predictors and outcome of patients.

**Results:** Patients included in the study were ASA IV. Four patients died in the first few days after surgery (2÷16 days). Mean length of stay in ICU was 5.2 ±3.4 days. Univariate analysis showed a correlation between hypoalbuminemia and the onset of MOF (p = 0.004); reduction of the lymphocyte count and risk of MOF (p = 0.008). SOFA score showed a significant correlation with occurrence of pneumonia (p = 0.035) and MOF (p = 0.04). Including the 30-day mortality among confounders, albumin and lymphocyte count were the strongest predictors of MOF. Length of stay in ICU and ventilation days did not have statistical significance. BMI showed no predictive value of any outcome.

**Conclusions:** Our sample was poor but results of our study seem to indicate malnutrition as an independent risk factor for elderly patients undergoing emergency surgery.

## P324 Early multidisciplinairy screening of dysphagia at admission to the emergency department – a pilot study

### D Melgaard, L Sørensen, D Sandager, A Christensen, A Jørgensen, M Ludwig, P Leutscher

#### North Denmark Regional Hospital, Hjørring, Denmark

**Introduction:** Dysphagia increase the risk of aspiration pneumonia, malnutrition, dehydration and death. This combined with the fact that patients with dysphagia have a longer stay in the hospital makes early prognosis and appropriate treatment important. Knowledge about effect of early dysphagia screening is limited. The aim of this study is to examine the prevalence of dysphagia in the Emergency Department (ED) population.

**Methods:** This study included consecutively hospitalized patients in 10 days from 2pm-10pm at the ED of North Denmark Regional Hospital. The screening took place within 2 hours of admission. Inclusion criteria were any of the following: age ≥65 years, neurological disorders, alcoholism, COPD, pneumonia, dyspnoea, diabetes or unexplained weight loss. A nurse screened patients with a water test and with signs of dysphagia tested by an occupational therapist with the V-VST and the MEOF-II.

**Results:** Of 140 eligible patients (56% male, median age 75 years) 95 (68%) were screened. It was impossible to screen 12 patients (9%) to limited time and 30 patients (21%) due to poor health condition and 5 patients (4%) declined participation. The prevalence of dysphagia in the study population was 16% (15 patients). Results from the water test were confirmed with V-VST and MEOF-II. In patients with lung related diseases or circulatory diseases was the prevalence respectively 25% and 24%. Patients, not screened due to poor health condition, were tested during hospitalisation and the prevalence of dysphagia was 75% in this group of patients.

**Conclusions:** The prevalence in ED patients was 16%. Patients transferred to other departments due to poor health condition had a prevalence of 75%. It is possible to screen patients in the ED. The water test is a useful screening tool in an acute setting.

## P325 Delivering nutrition targets in an intensive care unit – a quality improvement initiative

### L Shanahan

#### Mater Misericordiae University Hospital, Dublin, Ireland

**Introduction:** To improve protein and energy delivery in a nutrition delivery bundle was introduced to a Level 3 ICU. Greater protein and energy intake is associated with improved outcomes in the critically ill [1-4], but only 50% of prescribed protein and energy is delivered in ICUs worldwide [5,6].

**Methods:** Percentage of target protein and energy delivery was measured via participation in the International Nutrition Survey (INS) before and after a “nutrition delivery bundle” was introduced by the ICU dietitian. The nutrition delivery bundle involved all stakeholders in ICU nutrition care (Fig. 1) and included the following quality improvement measures: increased ICU dietetic staffing, update of ICU Enteral Feeding Protocol with staff education, use of higher protein formulations, earlier patient nutrition assessment, daily calculation of percentage nutrition delivery, increased nutrition communication through more regular discussion of patient care with medical team, expansion of choice of nasojejunal tube available, 6 monthly reporting of key nutrition performance indicators, improved resources for cover dietitian(s) when ICU dietitian on leave (Fig. 2).

**Results:** Prior to a nutrition delivery bundle being introduced the Mater Misericordiae University Hospital (MMUH) ICU achieved 59% of protein and 62% of energy targets over the first 12 admission days of 20 consecutive mechanically ventilated patients in ICU >72hrs enrolled in the International Nutrition Survey. This increased to 75% of protein and 79% of energy targets in 2014 (Table 1).

**Conclusions:** A 27% improvement in protein and energy delivery to critically ill patients was seen after the introduction of a dietitian-led nutrition delivery bundle.

**References**

1. Compher C et al. Crit Care Med 45:156-163, 2017

2. Yeh DD et al. JPEN 40:37-44, 2015

3. Nicolo M et al. JPEN 40:756-762, 2015

4. Ferrie S et al. JPEN 40:795-895, 2015

5. Heyland DK et al. JPEN 27:74-83, 2003

6. Cahill NE et al. Critical Care Medicine 38:395-401, 2010.

**Table 1 (abstract P325).** International Nutrition Survey Results 2011 and 2014. MMUH, Mater Misericordiae University Hospital

	MMUH 2014	All sites 2014	MMUH 2011	All sites 2011
% Energy target achieved	79	58	62	64
% Protein target achieved	75	54	59	60

**Fig. 1 (abstract P325).**
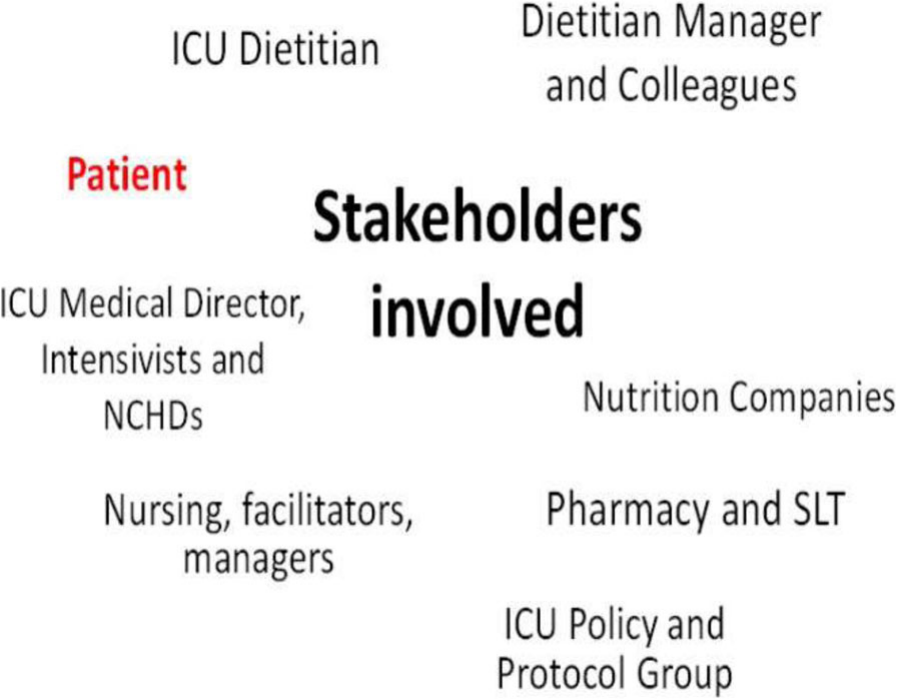
Stakeholders involved. ICU, intensive care unit; SLT, speech and language therapy; NCHDs, non-consultant hospital doctor

**Fig. 2 (abstract P325).**
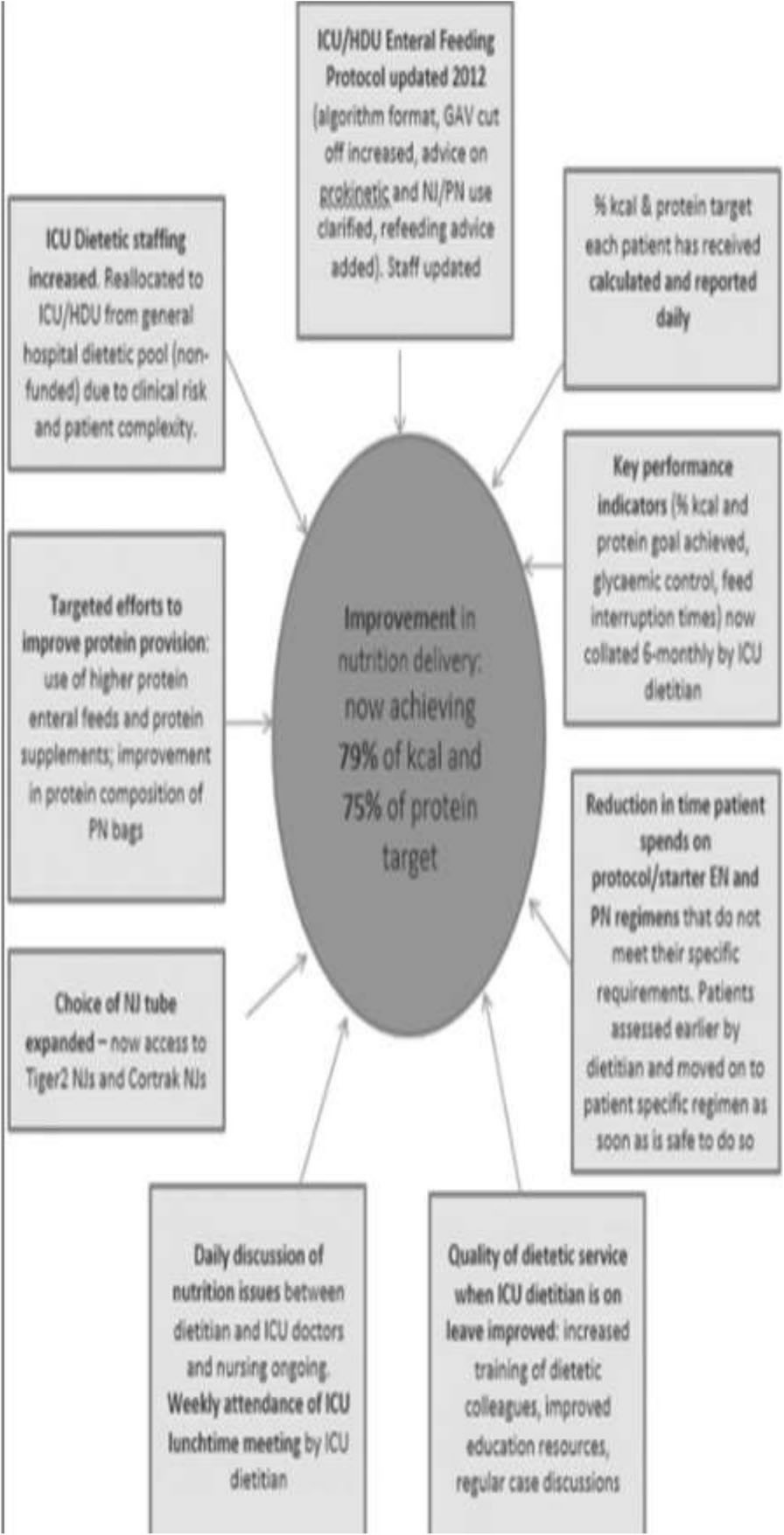
Nutrition Delivery Bundle. ICU, intensive care unit; HDU, high dependency unit; GAV, gastric aspirate volume; NJ, nasojejunal; PN, parenteral nutrition; EN, enteral nutrition

## P326 Monitoring energy demand in the multiple trauma critically ill patient with sepsis based on indirect calorimetry. a prospective observational monocentric study

### O Bedreag, A Rogobete, C Cradigati, M Sarandan, C Mihoc, S Popovici, D Sandesc

#### Victor Babes University of Medicine and Pharmacy, Timisoara, Romania

**Introduction:** The critically ill polytrauma patient with sepsis presents with variable energetic necessities characterized by a pro-inflammatory, pro-oxidative and hypermetabolic status. One of the challenges the ICU doctor faces is adapting the nutritional therapy based on the individual needs of each patient. Through this paper we wish to highlight the trend of energy needs in the case of critically ill polytrauma patients with sepsis by using non-invasive monitoring of respiratory gases based on indirect calorimetry (GE Healthcare, Helsinki, Finland).

**Methods:** This is a prospective observational study carried out in the Anesthesia and Intensive Care Unit “Casa Austria”, Emergency County Hospital “Pius Brinzeu”, Timisoara, Romania. We monitored VO2, VCO2, energy demand (ED), and specific clinical and paraclinical data. We measured energy demand values monitored by direct calorimetry with values calculated based on standard formulas.

**Results:** 21values have been recorded in the study. The mean VO2 was 3.3 ± 0.4 ml/min/kg, the mean VCO2 was 2.3 ± 0.3 ml/min/kg. In regard with energy demand, the mean ED obtained through direct calorimetry was 2393.2 ± 912.9 kcal/day, and those obtained by using mathematic formulas were 1988.6 ±1100 kcal/day (p < 0.05). Moreover, statistically significant differences have been observed regarding the mean difference between energy demand determined using indirect calorimetry and that determined mathematically, respectively between the enteral and parenteral administered ED.

**Conclusions:** Continuous monitoring of the energy demand in critically ill patients with sepsis can bring important benefits in regard with the clinical prognosis of these patients through the individualization and adaption of intensive therapy for each patient.

## P327 Measuring changes in muscle mass during critical illness: d3-creatine dilution

### W. Evans^1^, J. James^2^

#### ^1^University of California, Berkeley, Berkeley, California, USA,^2^Lyric Pharmaceuticals, Inc, South San Francisco, California, USA

**Introduction:** Cachexia is defined as a complex metabolic syndrome associated with underlying illness, characterized by loss of muscle with or without loss of fat. In cancer cachexia, reduction in muscle size has been demonstrated to be an independent risk factor for mortality. Loss of muscle in ICU patients is rapid and extensive and is also associated with mortality risk, but methods to measure muscle mass in these patients are lacking. Surrogate methods (DEXA, CT, ultrasound, total body water) do not measure muscle mass directly

**Methods:** The D3-creatine (D3-Cr) dilution method takes advantage of the fact that 98% of Cr is found in muscle and that muscle mass can be assessed by Cr pool size. Cr is transported into muscle against a concentration gradient and irreversibly converted to creatinine (Crn), which is excreted in urine. A single oral dose of D3-Cr is transported to skeletal muscle, and measurement of D3-Crn enrichment in a spot urine sample provides an accurate estimate of skeletal muscle mass.

**Results:** The method has been validated in preclinical and clinical studies; in a large longitudinal observation study in older men, D3-Cr muscle mass was strongly associated with habitual walking speed, risk of falls, and incident mobility limitation; DEXA failed to show these relationships. The D3-Cr method is being used in a NICU study to measure changes in muscle mass in neonates (Gates Foundation Grant). Further, this method has been incorporated into a trial assessing the treatment effects of a ghrelin agonist in ICU patients with enteral feeding intolerance (NCT02784392). In this trial, the D3-Cr dose is delivered intravenously and a spot urine sample is collected at baseline and postdose.

**Conclusions:** The D3-Cr method provides a non-invasive, accurate way to assess therapeutic agents that may mitigate the loss of skeletal muscle mass; it is of particular utility in clinical settings where changes in muscle mass are consequential, such as muscle loss during an ICU admission.

## P328 Enteral nutrition (EN) enriched with ω3 fatty acids modulates systemic inflammatory balance and cytokines after poor grade subarachnoid haemorrhage (SAH)

### M Casadio^1^, M Zanello^2^, V Chiarini^3^, E Mariani^4^

#### ^1^IRCCS Neurological Sciences Institute of Bologna, Bellaria Hospital, Bologna, Italy,^2^IRCCS Neurological Sciences Institute of Bologna, Bellaria Hospital, University of Bologna, Bologna, Italy,^3^University of Bologna, Bologna, Italy,^4^IRCCS Rizzoli Institute, Bologna, Italy

**Introduction:** Systemic inflammatory response syndrome(SIRS) SAH-related determines neurological and systemic dysfunctions [1]. Artificial nutritional support in acute phase can have a pharmaco-nutritional role [2].

**Methods:** In this randomized controlled trial we included during 8 months all consecutive patients admitted to the Intensive Care Unit after aneurysmal poor grade SAH. Patients were randomized to receive ω-3 fatty acids enriched EN (Peptamen AF®, 3.6-4.3g/day of ω -3 fatty acids intake) or a control isocaloric isonitrogenous EN (Nutrison Energy®-Isosource Protein®). EN administration started within 48 hours, caloric requirement was set at 25 kcal/kg/day and protein intake at 1.5g/kg/day, as whey proteins for the ω-3 modified EN and casein for the control EN.

**Results:** We enrolled 32 patients: 13 received the control EN and 19 the ω -3 modified one. No differences were found in demographics, clinical characteristics and cytokines balance (IL-1β, IL-6, IL-8, IL-17, INF-γ, TNF-α, MCP-1, RANTES, IL-4, IL-10, IL-13) at admission. After 8 days of EN administration we found in ω-3 group a higher level of haptoglobin (466.9 +/-157 vs 325.2 +/-186mg/dl, p<0.05), and prealbumine (39.7 +/- 18 vs. 24.2 +/-11 mg/dl), a reduction of inflammatory factor IL-6 (33.2 +/-6,5 vs. 58.5 +/-7.3 pg/mL, p<0.05), PCR (8.1 +/-5.5 vs. 11.2 +/-4.3 mg/dl, p< 0.05) and a shift of inflammatory balance towards anti-inflammation (p <0.05).

Ω -3 group showed a higher EN tolerance (caloric intake: 26 +/-3 vs 22 +/-3 kcal/kg, p=0.05; protein intake: 1.4 +/-0.5 vs 1.2 +/-0.5, p=0.05) with a better nitrogen balance (p<0.05), a decrease in SOFA score at day 8 (-2 vs -1.1, p<0.05), more days free from SIRS (3.3 vs 1 days, p<0.05) and 3 more days free of antibiotic therapy (p<0.05).

**Conclusions:** Early ω-3 fatty acids enriched EN with whey proteins reduces SIRS induced by SAH and ameliorates nutritional status.

**References**

[1] Yoshimoto Y et al. Stroke 32:1989, 2001

[2] Calder PC et al. Clin Nutr 30:1, 2017

## P329 The impact of supplemental parenteral nutrition early during critical illness on invasive fungal infections: a secondary analysis of the epanic randomised controlled trial

### G De Vlieger, C Ingels, PJ Wouters, Y Debaveye, I Vanhorebeek, J Wauters, A Wilmer, MP Casaer, G Van den Berghe

#### KULeuven, Leuven, Belgium

**Introduction:** Postponing initiation of supplemental parenteral nutrition (PN) to beyond one week in the intensive care unit (ICU) (late-PN) reduces ICU-acquired infections [1] and antimicrobial drug costs [2] as compared with initiation of PN within 24–48h of admission (early-PN). We hypothesize that late-PN also reduces the risk of ICU-acquired invasive fungal infections (IFI) and antifungal drug costs during hospital stay.

**Methods:** In a secondary analysis of the EPaNIC [1] we identified patients with an ICU-acquired IFI based upon the study database, the health economy analysis [2] and patients files. We assessed the impact of late-PN (N=2,328) versus early-PN (N=2,312) on acquired IFI and on the likelihood over time of acquiring an IFI in uni- and multivariable (adjusting for age, body mass index, nutritional risk, acute physiology and chronic health evaluation II score, diagnostic group, immunocompromised and sepsis upon admission, history of diabetes mellitus or malignancy) way and hospital antifungal drug costs in univariable way.

**Results:** Of 4,640 patients, 189 acquired an IFI in ICU, of whom 77 in late-PN (3.31%) and 112 in early-PN (4.84%) (p=.008) (Table 1). After multivariable adjustment, late-PN was signficantly associated with a reduced odds of acquiring an IFI and a reduced likelihood of acquiring an IFI at any time as compared with early-PN (Table 2). Antifungal drug costs per patient were lower in late-PN than in early-PN (Table 1).

**Conclusions:** Postponing the administration of supplemental PN to beyond one week reduced the risk and the likelihood at any time of acquiring an IFI in the ICU and lowered costs for antifungal drugs during hospital stay.

**References**

1. Casaer MP et al. N Eng J Med 365(6): 506–17, 2011

2. Vanderheyden S et al. Crit Care 16(3): R96, 2012

**Table 1 (abstract P329).** Univariable analysis using the Chi-square and student’s t test

	Early-PN (n=2,312)	Late-PN (n=2,328)	p-value
ICU-acquired IFI	112 (4.84%)	77 (3.31%)	0.008
Antifungal drug cost	440.1 € (SE 52.8)	260.4 € (SE 52.6)	0.02

PN: parenteral nutrition, ICU: intensive care unit, IFI: invasive fungal infections, SE: standard error

**Table 2 (abstract P329).** Multivariable logistic regression and Cox proportional hazard ratio analysis

	Late-PN versus Early-PN	95% confidence interval	p-value
ICU-acquired IFI	Adjusted OR 0.67	0.49-0.92	0.01
Likelihood for acquiring an IFI at any time	Adjusted HR 0.71	0.53-0.94	0.02

PN: parenteral nutrition, ICU: intensive care unit, IFI: invasive fungal infection, OR: odds ratio, HR: hazard ratio

## P330 Vitamin C supplementation in the critically ill: systematic review and meta-analysis

### P Laferriere-Langlois^1^, F Lamontagne^1^, W Manzanares^2^

#### ^1^CHUS, Sherbrooke, Canada,^2^Hospital de Clinicas, Montevideo, Uruguay

**Introduction:** Vitamin C, an enzyme cofactor and antioxidant, could hasten the resolution of inflammation, which affects most intensive care unit (ICU) patients. While many observational studies have demonstrated that critical illness is associated with low levels of vitamin C, randomized controlled trials (RCTs) of high-dose vitamin C, alone or in combination with other antioxidants, yielded contradicting results. The purpose of this systematic review and meta-analysis is to evaluate the clinical effects of vitamin C when administered to various populations of ICU patients.

**Methods:** Eligible trials: RCTs comparing vitamin C, by enteral or parenteral routes, to placebo in ICU patients. Data Collection and Analysis: We searched MEDLINE, EMBASE, and the Cochrane Central Register of Controlled Trials. After assessing eligibility, data was abstracted in duplicate by two independent reviewers. Overall mortality was the primary outcome; secondary outcomes were infections, ICU length of stay (LOS), hospital LOS, and ventilator days. Pre-specified subgroup analyses were conducted to identify more beneficial treatment effects.

**Results:** Pooling 9 RCTs (n=1322) reporting mortality, vitamin C was not associated with a lower risk of mortality (risk ratio [RR]: 0.84, 95 % confidence interval [CI]: 0.48-1.37, P=0.44, I2=59%). In a subgroup analysis, trials of lower quality (n= 5) were associated with a reduction in mortality (RR 0.50, 95% CI 0.32, 0.77, P= 0.002), whereas high quality trials (n= 4) were not. No statistical difference existed between subgroups (P= 0.22). In addition, no effect was found on infections, ICU or hospital length of stay, and ventilator days.

**Conclusions:** Current evidence does not support the hypothesis that vitamin C supplementation improves clinical outcomes of ICU patients.

## P331 Achieved adequacy for energy - what about protein?

### AM Young, T Vassalos

#### Golden Jubilee National Hospital, Clydebank, UK

**Introduction:** The protein intake for patients who met adequacy for energy was assessed within our cardiothoracic intensive care. Nutritional support should aim to provide at least 80% of calorie requirements to achieve nutritional adequacy with suggested protein requirements of 1.2-2 g/kg/day [1]. Guidelines highlight the difficulty achieving the correct protein:energy ratio from nutritional support to meet this target especially in the obese population.

**Methods:** The audit was registered with clinical governance. Data was collected prospectively from patients requiring tube feeding for three or more days from January 2016 – October 2017 (Table 1). Data included type and volume of feed and calories from other sources. Patients who met adequacy for energy (Fig. 1) had protein intake calculated (g/kg) based on actual (ABW) or ideal body weight (IBW) where Body Mass Index (BMI) was 30kg/m^2^ or higher. Data analysed using Student t-test.

**Results:** Nutritional support was initiated within 48 hours for 88% (n=128) of patients. Full feed was established by day three (IQR 2-4 [range 1-8]) providing 1710 (±257) kcal/day. The patient population did not achieve the protein target (Fig. 2) with 3% exceeding 1.2 g/kg body weight. Men classed as obese had a significantly lower protein intake compared to men with BMI less than 30kg/m^2^ (p=0.04) and obese women (p=0.03).

**Conclusions:** The audit of nutritional support monitors the percentage of patients who achieve nutritional adequacy within the unit. However the majority of these patients did not meet the minimum protein requirements despite the range of feeds available. Patients who are obese may be most at risk of protein depletion as recommendations suggest require protein intake at upper end of range. The audit demonstrates a need to review feeding protocols.

**References**

1. McClave S et al. Enteral Nutr 40:159-211, 2016

**Table 1 (abstract P331).** Summary of population

Patients	216
Received >80% requirements	68% (n=146)
Males	64% (n=93)
Age (yrs)	61.3±15.3
BMI (kg/m^2^)	27.3±5.5
BMI >=30kg/m^2^	23% (n=34)
Days fed	11 (3 -51)
Mortality	15.7% (n=23)

**Fig. 1 (abstract P331).**
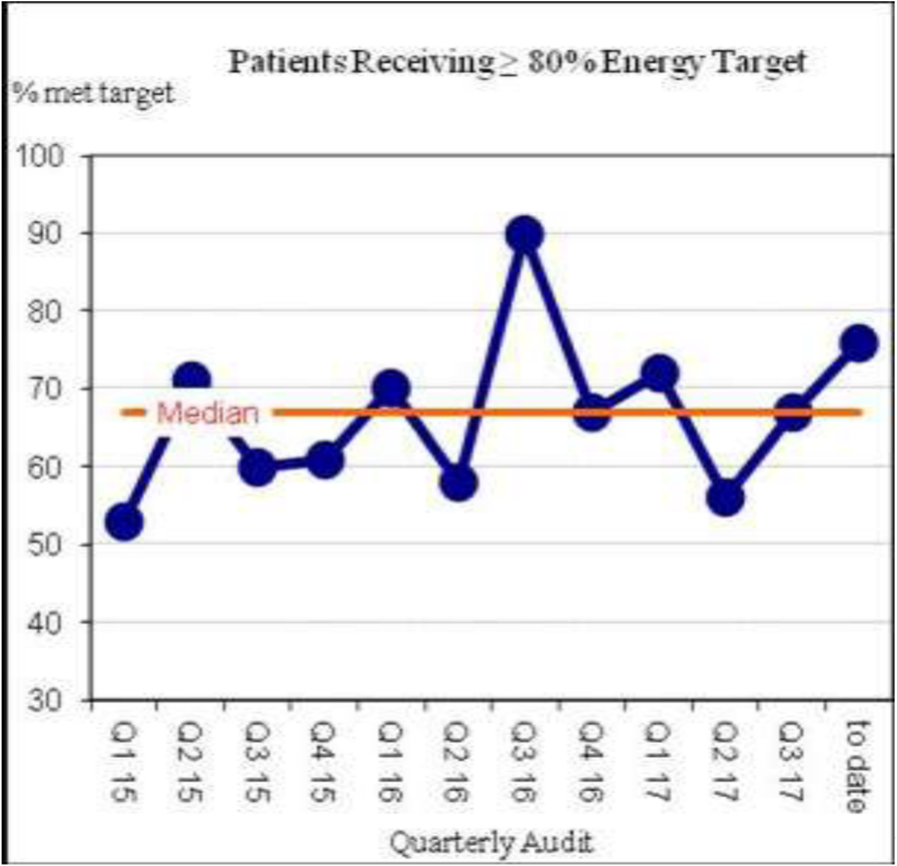
Monitoring Nutritional Adequacy

**Fig. 2 (abstract P331).**
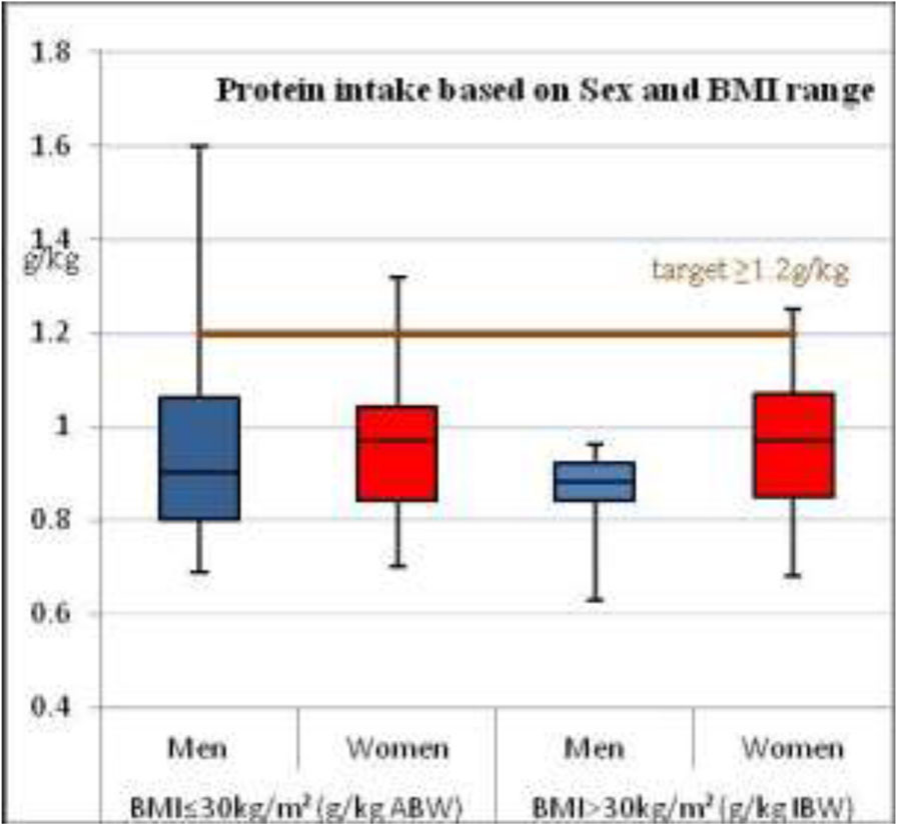
Protein intake based on sex and age

## P332 Energy requirements of the critically ill patient during the first week

### R Marinho^1^, R Sousa^2^, R Sousa^2^, M Lopes^1^, M Santos^3^, A Marinho^1^

#### ^1^Centro Hospitalar do Porto, Porto, Portugal,^2^Instituto de Ciencias Biomedicas Abel Salazar, Porto, Portugal,^3^Faculdade de Ciências da Nutrição e Alimentação da Universidade do Porto, Porto, Portugal

**Introduction:** The nutritional requirements of the critically ill patient have been a topic of intense discussion in recent years. If, in one hand, experts seek to optimize the patient’s nutritional support by following the international guidelines, which recommends giving the patients 25kcal/kg of body weight each day, some authors recommend an underfeeding protocol in the first week of hospital stay. This discrepancy revolves around the evolution of medical care, with better management of multiple organ dysfunction, hyperthermia, anxiety, pain and sedation, that could have reduced the nutritional needs of this highly consumptive and hypercatabolic patient.

Objective: To evaluate if the nutritional requirements of critically ill patients in their first week of hospital stay are in line with the international guidelines and the Harris- Benedict equation.

**Methods:** A transversal prospective study was developed to evaluate the energy requirements of the patients, using the indirect calorimetry method and the Harris- Benedict equation, over a period of four months. Stress variables like pathology group, hemodynamic support, sedation, body temperature, outcome and SOFA score were also evaluated.

**Results:** 46 patients were included in this study, with a mean energy expenditure of 19.22 ± 4.67 kcal/kg of body weight per day. 63% of these had energy expenditure values below 20kcal/kg per day assess by indirect calorimetry. Harris-Benedict and indirect calorimetry values for energy requirements were concordant (±10%) in only 33% of patients. Of the stress variables, only the SOFA score had any significant impact on the measured energy expenditure.

**Conclusions:** The nutritional requirements of the critically ill patient in the first week of intensive care stay are well below the values recommended by ESPEN guidelines. Indirect calorimetry is still the gold standard in the evaluation of energy requirements.

## P333 Association between modified nutric score and clinical outcome in Thai critically ill patients

### S Taesotikul^1^, N Thanintara-arj^2^, V Saifah^2^, V Tangsujaritvijit^3^, B Chindavijak^2^, P Dilokpattanamongkol^2^

#### ^1^Faculty of Pharmacy, Mahidol University, Department of Pharmacy, Bangkok, Thailand, ^2^Faculty of Pharmacy, Mahidol University, Bangkok, Thailand, ^3^Faculty of Medicine Ramathibodi Hospital, Mahidol University, Department of Medicine, Bangkok, Thailand

**Introduction:** Importance of nutrition risk assessment is to identify critically ill patients who might have benefit from early nutrition support for recovery of acute illness [1]. The purpose of this study is to determine the association of modified NUTrition Risk in the Critically ill (NUTRIC) score for categorising nutrition risk and clinical outcome in Thai population.

**Methods:** We conducted a single-centre retrospective cohort in medical intensive care unit (MICU), university hospital, Thailand. All adult patients admitted to MICU between June and November 2016 were eligible. Variables for assessment of modified NUTRIC score including age, Acute Physiology and Health Evaluation II (APACHE), Sequential Organ Failure Assessment score (SOFA), co-morbidity and days from hospital to ICU admission were collected. Patients with modified NUTRIC score >=5 were classified as high nutrition risk. Twenty-eight-day mortality and ICU-free days were analysed as primary and secondary outcome, respectively.

**Results:** Seventy patients with mean age of 60.8±18.5 and mean APACHE of 19.8±6.2 were recruited. High nutrition risk was detected in 43/70 (61%) patients. Twenty-eight-day mortality rate in high nutrition risk group was significantly higher than low nutrition risk group (48.8% vs 14.8%, respectively, p-value 0.004). ICU-free days in high nutrition risk group were also significantly shorter than low nutrition risk group (8.7±9.9 days vs 15.6±9.0 days, respectively, p-value 0.004). Modified NUTRIC score >=5 predicted 28-day mortality rate in Thai critically ill patients with sensitivity of 84.0%, specificity of 51.1% and positive predictive value of 48.8%.

**Conclusions:** More than half of MICU patients had high nutrition risk, and modified NUTRIC score was associated with 28-day mortality rate and ICU-free days. Modified NUTRIC score had high sensitivity, however it had poor specificity and positive predictive value in Thai population.

**Reference**

1. McClave SA et al. JPEN J Parenter Enteral Nutr. 40(2):159-211, 2016

## P334 Comparison of nutric score, nutritional risk screening (NRS) 2002 and subjective global assessment (SGA) in the ICU: a cohort study

### S Saseedharan^1^, EJ Pathrose^1^, DR Karnad^2^, AT Patil^1^

#### ^1^S L Raheja Hospital, Mumbai, India,^2^Jupiter Hospital, Thane, India

**Introduction:** Patients admitted to the intensive care unit (ICU) are usually at high risk of malnutrition [1, 2]. The purpose of our study was to compare the accuracy of Nutric score, NRS 2002 and SGA in predicting LOS-ICU, LOS-HOSP and in-hospital mortality.

**Methods:** A total of 348 consecutive patients admitted between March to June 2016 in a mixed (medical/surgical) ICU were assessed on day of admission using the three screening tools to classify them into high-risk and low-risk of malnutrition. Day 1 APACHE 2 scores and demographic data were recorded. LOS-ICU, LOS-HOSP in-hospital mortality and secondary outcomes studied were need for supplemental nutritional support, need for ventilation and need for dialysis in high-risk and low-risk patients by each nutrition assessment tool.

**Results:** Of the 348 patients studied, 221 (63.5%) were males and 127 (36.5%) were females. 67.87% males and 71.65% females were found to be at a high risk of malnutrition by at least one of the scores. The mean APACHE 2 score for patients at high risk (using any one screening tool) was 15.11 (SD 6.10) and 8.04 for the low risk group (SD 3.34; p <0.01). The NRS 2002 and SGA demonstrated statistically significant correlation(p=0.001) for length of ICU stay for both the high risk and low risk group whereas only the NRS 2002 correlated significantly for the length of hospital stay(p=0.002). Mortality was significantly higher in high risk patients identified using all 3 scores.

**Conclusions:** There was a wide difference in the percent of patients identified as high-risk using each of the 3 scores.

**References**

1. Correia MI, Campos AC, Study EC. Nutrition 19(10):823-5, 2003

2. Waitzberg DL, Caiaffa WT, Correia MI. Nutrition17(7):573-80, 2001

## P335 Nitrogen balance in postoperative cancer patients in ICU

### CR Lordani^1^, IR Porto^1^, RG Eckert^2^, NM Valerio^1^, T Lordani^1^, RC Schimidt^2^, AC Jorge^1^, P Duarte^1^

#### ^1^Hospital Universitario do Oeste do Parana, Cascavel, Brazil,^2^Hospital do Câncer/UOPECCAN, Cascavel, Brazil

**Introduction:** Nitrogen Balance (NB) may be an important tool in the nutritional management of critically ill patients. Cancer patients present a special challenge regarding nutrition, due to its peculiar characteristics related to neoplasia and adjuvant treatments. Objectives: To evaluate NB in patients with solid cancer in the postoperative period in the ICU, analyzing the correlation between NB and the mortality outcome in the ICU.

**Methods:** Retrospective cohort study. We evaluated adult patients (>18 years) admitted to the ICUs of two different hospitals, with diagnosis of current cancer in postoperative period (elective or emergency surgeries). Patients were excluded if the diagnosis of cancer was not confirmed. NB (measured through analysis of dietary protein intake subtracted from 24-hour urinary urea plus an estimate of non-urinary losses) was calculated on the 1st, 3rd and 5th ICU day. NB was measured only while the patient was in the ICU.

**Results:** During the study period, 125 patients were included (mean age 58.1, mean APACHE 17.4, 65.6% male). Admission APACHE II and abdominal-site surgery were predictors of mortality. The NB of all patients was negative on the 1st ICU day. In the patients who survived, NB of the 3rd and 5th day remained stable (negative), whereas in patients who died NB was more positive (Fig. 1). There was no difference in the amount of protein ingested on the 1st day between survivors and deceased patients.

**Conclusions:** Among adult patients with solid cancer in the postoperative period in the ICU, NB was persistently negative in the survivors between 1st and 5th ICU day, but among the patients who died NB tended to be more positive on the 3rd day. NB monitoring could allow a more adequate individualization of nutritional management in this group of patients.

**Fig. 1 (abstract P335).**
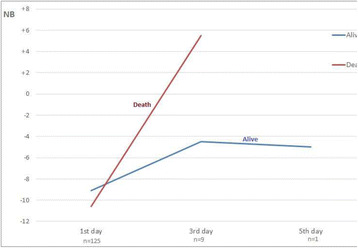
Nitrogen Balance in 1st, 3rd and 5th ICU day

## P336 After the ICU: the nutritional desert. Adequacy of nutritional therapy during and after ICU stay

### MCM Piérar, J Demol, C Verhelst, I Passia, HD Spapen, MLN Malbrain, E De Waele

#### Department of Intensive Care UZ Brussel, Brussels, Belgium

**Introduction:** Nutritional therapy plays an important role in the treatment of critically ill patients. Caloric and protein goals are defined, and artificial nutrition tailored to the targets which are related to outcome [1]. Questions rise about the mean caloric and protein needs of patients, once discharged from ICU, and the evolution of body weight, and nutritional adequacy. The aim is to know the ratios between caloric needs and intake of patients with a minimum stay at ICU of 5 days.

**Methods:** After evaluation of 146 critically ill patients, 12 patients were prospectively followed during their entire hospitalization. Data concerning nutritional needs, prescriptions and delivery were collected from the electronic medical file. Nutritional calculations of oral intake were done by Nubel. Ratios were made during the entire stay and body weight was followed up.

**Results:** In 5 female and 7 male patients, median age 63.5 years (range 26-84 year), estimated body weight of 74.8 ± 21 kg and actual body weight of 73.3± 17 kg, a mean caloric need of 1795 ± 479 kcal/day and an effective delivery of 1348 ± 508 kcal/day was observed. Body weight increased in two patients and decreased in 10 (83%). In ten out of twelve patients, underfeeding was present. One patient with a caloric need of 1125 kcal/day received a mean caloric load of 230 kcal/day (20.4%).

**Conclusions:** The overall observed evolution in body weight was negative in most of the patients. Nutritional adequacy was low after ICU discharge and never reached target.

**References**

1. Zusman O, Singer P. Crit Care 21(1):128, 2017

## P337 Both the immediate and delayed inflammatory responses in all major organs are reduced by single dose 17β-estradiol following severe burn injury

### JG Wigginton, PE Pepe, KR AbdelFattah, JW Gatson, AH Idris, V Warren, JP Minei, DL Maass

#### UT Southwestern Medical Center, Dallas, Texas, USA

**Introduction:** Severe burn injury can create a rapid-onset, sustained pro-inflammatory condition that can severely impair all major organs. This massive systemic response has been documented clinically by associated biomarker measurements including dramatic elevations in cytokines such as IL-6. The severity of multi-organ injury and subsequent development of other systemic complications in burn patients have been well-correlated with IL-6 levels, including the increased risk of sepsis/multi-organ failure and associated morbidity and mortality. Considering that estrogen is a powerful and easy to use anti-inflammatory agent, an experimental burn model was created to test the potential value of parenteral 17β-estradiol (E2) as a feasible and inexpensive early intervention to mitigate the the profound pro-inflammatory response associated with severe thermal injury.

**Methods:** Male rats (n = 28) were assigned randomly into three groups: 1) controls/no burn (n = 4); 2) burn/placebo (n = 12); and 3) burn/E2 (n = 12). Burned rats received a 40% 3° TBSA dorsal burn, fluid resuscitation and one dose of E2 or placebo (0.5 mg/kg intra-peritoneal) 15 minutes post-burn. Eight animals from each of the two burn groups (burn/placebo and burn/E2) were sacrificed at 30 minutes (sham group at 7 days only), with four each of the two burn groups sacrificed at 45 days. Tissue samples from 9 major organs and serum were obtained and analyzed by ELISA for IL-6 at each of these intervals.

**Results:** In the burned rats, 17β-estradiol decreased the organ levels of IL-6 significantly as measured at both early (30 min.) and late (45 day) phases post-burn (Figs. 1 & 2. Also, sham animal levels were comparable to the estradiol group,

**Conclusions:** Experimentally, a single, early post-burn dose of estrogen significantly mitigates the associated detrimental inflammatory response in all major organs up to 45 days. In turn, this may present a promising potential therapy to decrease the widespread multiple-organ dysfunction seen in severe burn injury patients.

**Fig. 1 (abstract P337).**
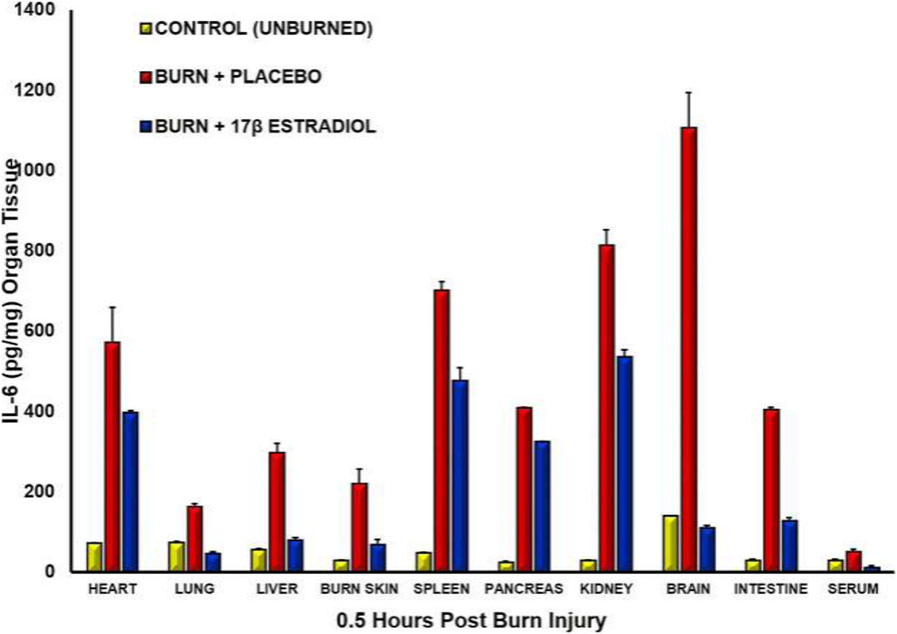
30 minute post-burn IL-6 levels in all major organs

**Fig. 2 (abstract P337).**
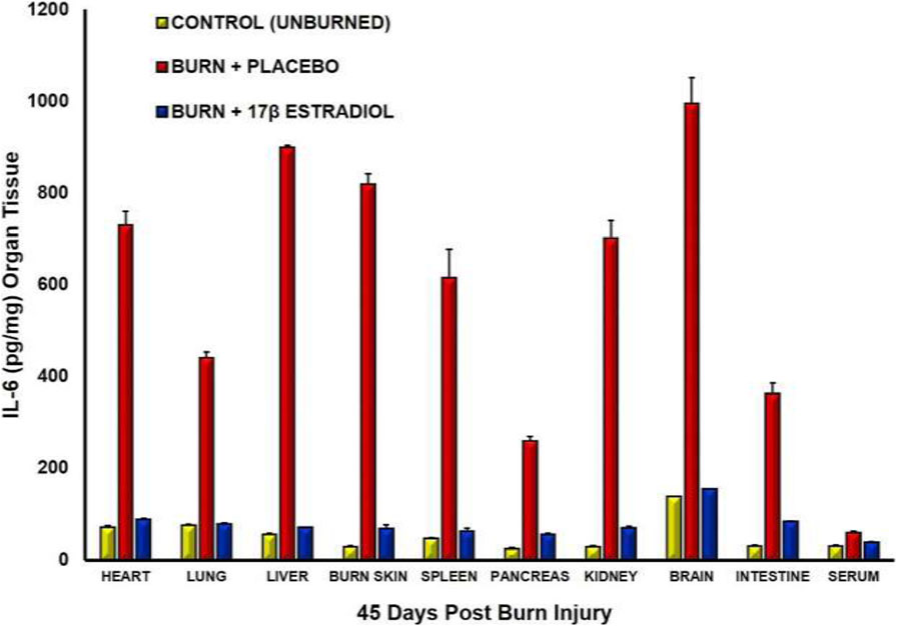
45 day post-burn IL-6 levels in all major organs

## P338 Early, single-dose estrogen increases levels of brain-derived neurotrophic factor (BDNF), a neurotrophin for neuronal survival and neurogenesis following indirect brain inflammation caused by severe torso burns

### JG Wigginton, PE Pepe, KR AbdelFattah, JW Gatson, AH Idris, V Warren, JP Minei, DL Maass

#### UT Southwestern Medical Center, Dallas, Texas, USA

**Introduction:** Prior studies have found that patients with severe burns may suffer significant neurocognitive changes. While frequently attributed to psycho-social issues, we have found a substantial, rapid and sustained (30 min - 45 day) increase in rat brain inflammatory markers (for example, IL-6) following remote torso burns that is blunted by a single post-burn dose of estrogen. Brain-derived neurotrophic factor (BDNF), one of the most active neurotrophins, protects existing neurons and encourages the growth and differentiation of new neurons and synapses. As estrogens not only blunt inflammation but also exert an influence on CNS growth factors, we hypothesized that 17β-estradiol (E2) might affect levels of BDNF in the post-burn rat brain.

**Methods:** Male rats (n = 44) were assigned randomly into three groups: controls/no burn (n = 4); burn/placebo (n = 20); and burn/E2 (n = 20). Burned rats received a 40% 3° TBSA dorsal burn, fluid resuscitation and one dose of E2 or placebo (0.5 mg/kg intraperitoneally) 15 minutes post-burn. Eight animals from each of the two burn groups (burn/placebo and burn/E2) were sacrificed at 24 hours and at 7 days, respectively (sham group at 7 days only), with four each of the two burn groups sacrificed at 45 days. Brain tissue samples were analyzed by ELISA for BDNF.

**Results:** Mean levels of BDNF were significantly elevated within 24 hours and continued to increase up to 45 days post-injury in burned animals receiving the 17β-estradiol (>300 pcg/mg) as compared with the placebo-treated burned animals (<160 pg/mg) and controls (<120. pcg/mg). See Fig. 1.

**Conclusions:** Early, single-dose estrogen administration following remote severe burn injury significantly elevated levels of BDNF in brain tissue. This finding may represent an extremely novel and important pathway to enhance both neuroprotection and neuroregeneration in burn patients.

**Fig. 1 (abstract P338).**
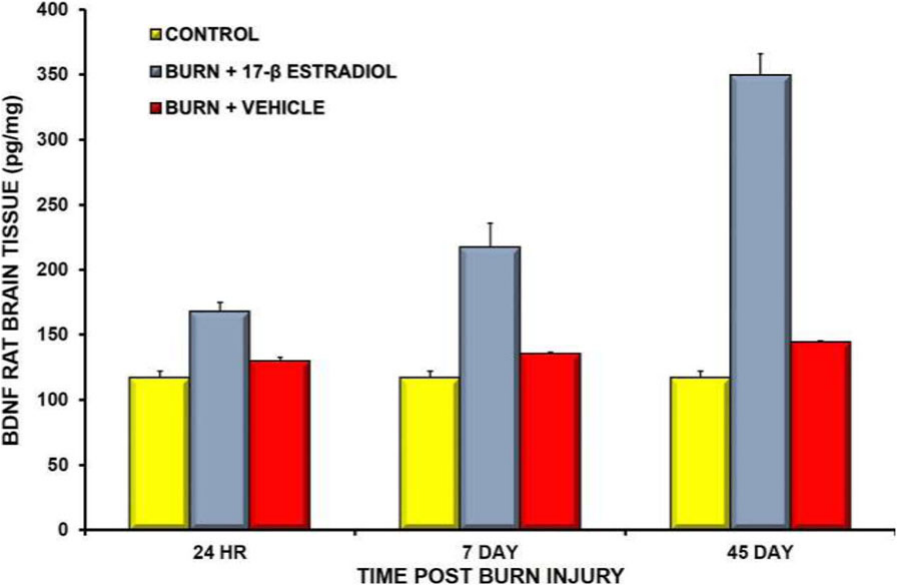
BDNF Levels (pg/mg) in the brain

## P339 The value of cortisol in patients with the infection and multiple organ dysfunction.

### S Tachyla, A Marochkov

#### Mogilev Regional Hospital, Mogilev, Belarus

**Introduction:** Hormones changes in patients with infection and multiple organ dysfunction is a topic that hasn’t been adequately studied. Goal of study: to establish the value of cortisol in patients with infection and multiple organ dysfunction.

**Methods:** After approval the ethics committee of the Mogilev Regional Hospital a prospective observational study was performed. The study included 181 patients aged 18 to 87 years. All patients were hospitalized in the Intensive Care Unit with the infection and multiple organ dysfunction. Patients with endocrine diseases and receiving glucocorticoids were excluded. Cortisol levels were measured on admission and during the course of treatment by radioimmunoassay. In Group L (n = 16) patients had a low levels of cortisol, in the M group (n = 96) - normal cortisol, in group H (n = 69) - high cortisol.

**Results:** Cortisol level was in L-group 91.9 (8.28, 131.7) nmol/L, in M-group 410.9 (292.8; 504.7) nmol/L, in H-group 934.2 (763, 6; 1495.5) nmol/L.

It is found that the mortality was higher in the groups L - 43.8% (p = 0.33) and H - 47.8% (p = 0.03), than in the M-group - 31.3%. The M-group odds ratio equals 2.02 at 95% confidence interval 1.06 - 3.82 when compared with the H-group. In the M-group in survivors patients (n = 36) showed a decrease cortisol with 1281 (1033.8, 1702.5) nmol/L to 912.3 (801.5, 1068.8) nmol/L (p = 0.01). While the no survivors patients (n = 33) showed increase cortisol with 732 (657.1, 749.2) nmol/L to 1491.2 (1000; 1600) nmol/L (p = 0.008).

Thus itself cortisol level is not a marker of mortality. Receiver operating curve analysis for cortisol was performed: area under the curve equals 0.56 at 95% confidence interval of 0.47 - 0.65 (p = 0.19), sensitivity 48.4%, specificity 70.6%.

**Conclusions:** In patients with infection and multiple organ dysfunction may be observed disorders in cortisol levels. These disorders require correction to prevent the increased mortality.

## P340 Cortisol profiles in the critically ill after cardiac surgery

### B Gibbison^1^, C Rogers^1^, J Evans^1^, K Stevenson^1^, G Angelini^1^, S Lightman^2^

#### ^1^University of Bristol/University Hospitals Bristol NHS Trust, Bristol, UK,^2^University of Bristol, Bristol, UK

**Introduction:** The hypothalamic-pituitary-adrenal (HPA) axis is a key regulator of critical illness. Cortisol and adreno-corticotrophic hormone (ACTH) are pulsatile, which emerges from the feed forward - feedback of the two hormones [1]. Different genes are activated by continuous or pulsatile activation of the glucocorticoid receptor, even when the total amount is the same [2]. We aimed to characterise the ACTH and cortisol profiles of patients who were critically ill after cardiac surgery and assess the impact of inflammatory mediators on serum cortisol concentrations.

**Methods:** 20 patients with >2 organ system failure, >2 days after cardiac surgery were recruited. Total cortisol was assayed every 10 min, ACTH every hour and IL1, IL2, IL4, IL6, IL8, TNF-α every 4 hours. Cortisol binding globulin (CBG) was assayed at 0 and 24hrs. The relationship between cortisol and the inflammatory mediators was quantified in individual patients using a mixed regression model.

**Results:** All profiles showed pulsatility of both cortisol and ACTH and there was concordance between the two hormones (See Fig. 1). One patient died after 23 hours (see Fig. 2). This patient lost pulsatility and concordance of cortisol and ACTH. Mean CBG was 26.89μ g/ml at the start of sampling and 28.13μ g/ml at the end. There was an association between IL6 (p=0.0002), IL10 (p<0.0001), IL4 (p=0.029) and serum cortisol levels. There was no association between the other mediators and cortisol.

**Conclusions:** Cortisol and ACTH are both pulsatile in critical illness. Because pulsatility emerges from the interaction between the two hormones[2] – the premise of a ‘disconnect’ between the pituitary and adrenal gland is refuted. IL6, IL10 and IL4 may have roles in the control of cortisol during critical illness.

**References**

1. Walker JJ et al. Proc Biol Sci. 277:1627-33, 2010

2. Conway-Campbell BL et al. J Neuroendocrinol. 22:1093-100, 2010

**Fig. 1 (abstract P340).**
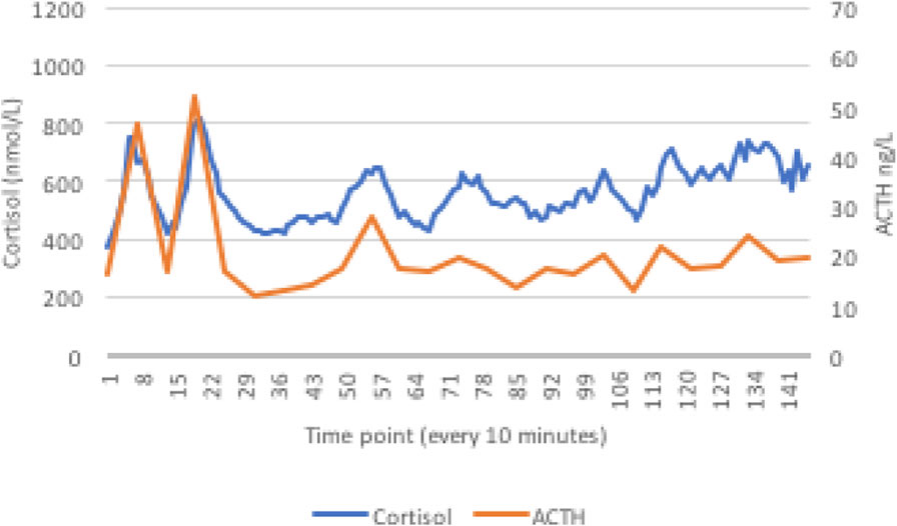
Example of one patient undergoing 24 hour sampling of cortisol and ACTH (Patient survives).

**Fig. 2 (abstract P340).**
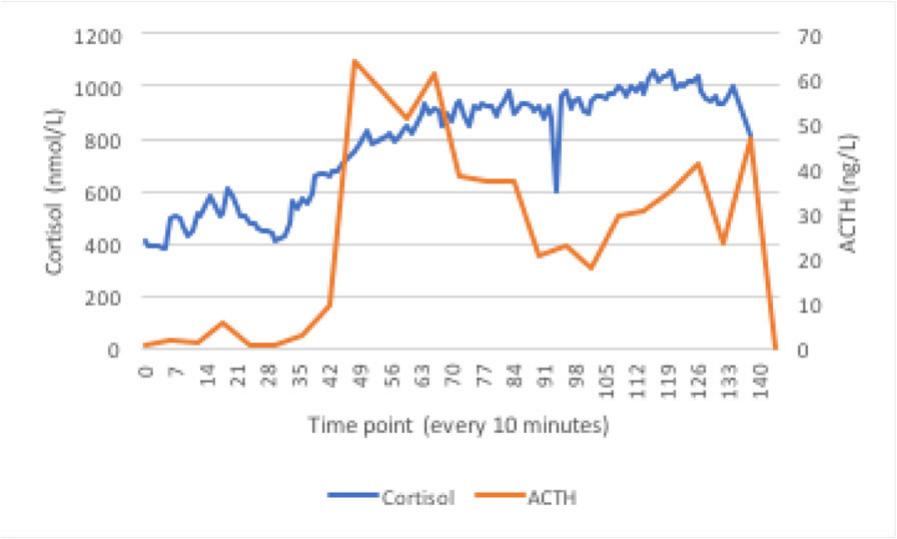
Example of one patient undergoing 24 hour sampling of ACTH and cortisol (Patient dies at hour 23).

## P341 The glucocorticoid receptor in the liver plays a key role in cortisol homeostasis and survival from sepsis

### M Jenniskens, R Weckx, T Dufour, S Vander Perre, L Pauwels, S Derde, F Güiza, G Van den Berghe, L Langouche

#### KU Leuven, Leuven, Belgium

**Introduction:** Elevation in plasma cortisol is a vital response to sepsis and partially brought about by reduced cortisol breakdown in which bile acids (BAs) may play a role. Vice versa, cortisol can also upregulate BAs. We hypothesized a central role for the hepatic glucocorticoid receptor (hGR) in cortisol and BA homeostasis and in survival from sepsis.

**Methods:** In a mouse model of sepsis, we documented hGR expression and investigated the impact of hepatocyte-specific shRNA-knockdown of GR on markers of corticosterone (CORT), BA and glucose homeostasis, inflammation and survival. We also compared hGR expression in human septic ICU and elective surgery patients.

**Results:** In mice, sepsis reduced hGR expression with 21% (p=0.04), elevated plasma CORT, BAs and glucose and suppressed A-ring-reductases. Also in human patients, sepsis reduced hGR expression (p<0.01), further suppressed by treatment with steroids (p=0.04). In septic mice, further and sustained hGR-inhibition increased mortality from 12% to 60% (p<0.01). At 30h, hGR-inhibition prevented the rise in total plasma CORT, but did not affect A-ring-reductases expression. However, it further reduced CORT binding proteins, resulting in elevated free CORT equal to septic mice without modified hGR. After 3 days of hGR-inhibition in sepsis, total and free CORT were comparable to septic mice without modified hGR, now explained by further reduced A-ring-reductase expression, possibly driven by higher hepatic BA content. HGR-inhibition blunted the hyperglycemic sepsis response without causing hypoglycemia, markedly increased hepatic and circulating inflammation markers and caused liver destruction (p<0.05), the severity of which explained increased mortality.

**Conclusions:** In conclusion, sepsis partially suppressed hGR expression, which appears to upregulate free CORT availability via lowered CORT binding proteins and A-ring-reductases. However, further sustained hGR suppression evoked lethal excessive liver and systemic inflammation, independent of CORT availability.

## P342 Upregulation of cortisol in sepsis and its association with markers of hemostatic dysregulation

### A Walborn, S Walborn, D Hoppensteadt, P Maia, R Green, G Wegryzn, M Mosier, J Fareed

#### Loyola Univeristy Medical Center, Maywood, Illinois, USA

**Introduction:** Cortisol levels have been found to be increased in sepsis patients, and high cortisol levels have been correlated with increased mortality. The purpose of this project is to assess the association of plasma cortisol levels with severity of coagulopathy in a population of patients with sepsis and clinically confirmed DIC.

**Methods:** Citrated, de-identified plasma samples were collected from 52 adults with sepsis and suspected DIC at the time of ICU admission. Platelet count was determined as part of standard clinical practice. PT/INR and fibrinogen were measured using standard techniques on the ACL-ELITE coagulation analyzer. Cortisol, D-dimer, PAI-1, CD40L, NLRP3, and microparticles were measured using commercially available ELISA kits and were performed. DIC score was calculated using ISTH scoring algorithm.

**Results:** Cortisol showed significant variation based on DIC status (Kruskal-Wallis ANOVA, p < 0.0001). Patients with non-overt DIC and overt DIC exhibited significantly elevated cortisol levels compared to healthy controls (p < 0.0001 for both groups). Cortisol levels showed DIC based variations. Patients with sepsis and overt DIC had elevated cortisol compared to patients with sepsis and no DIC (p = 0.0069) (Fig. 1). Correlations were evaluated between cortisol and hemostatic markers platelets, fibrinogen, INR, D-Dimer, and PAI-1 as well as with the inflammatory marker, NLRP3 and the platelet markers CD40L and microparticles. Cortisol showed significant correlations with D-Dimer, PAI-1, and INR. (D-Dimer Spearman r = 0.480, p = 0.001; PAI-1 Spearman r = 0.415, p = 0.002; INR Spearman r = 0.305, p = 0.037).

**Conclusions:** Cortisol showed a significant association with hemostatic status in a population of patients with sepsis and well-defined coagulopathy. Cortisol levels were significantly elevated in patients with overt or non-overt DIC compared to healthy individuals and in patients with overt DIC compared to those with sepsis without DIC.

**Fig. 1 (abstract P342).**
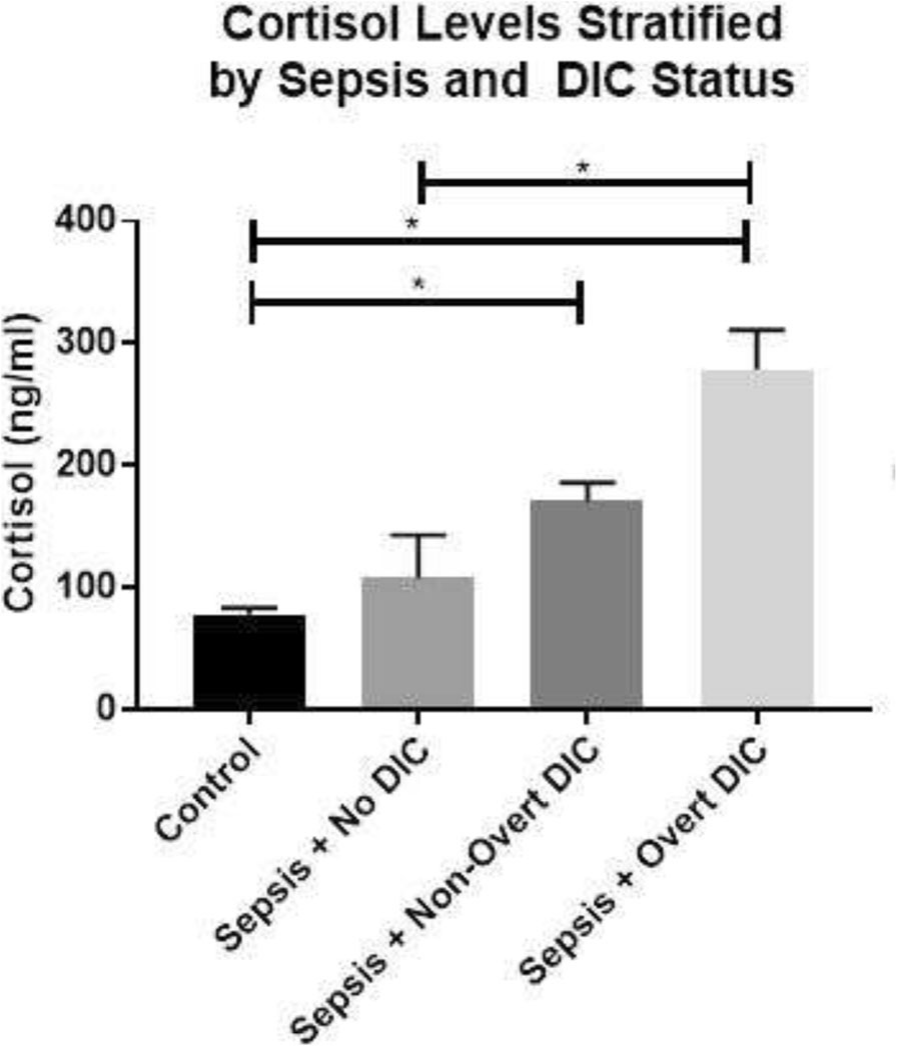
Cortisol Levels Stratified by Sepsis and DID Status

## P343 Clinical features and probability of adrenal insufficiency in patients who were transported to an emergency department with hypoglycemia

### T Kawahara, M Furuya, N Tominaga, M Toda, N Toyama, S Kondo

#### Shinkomonji Hospital, Kitakyushu, Japan

**Introduction:** In most cases presenting with hypoglycemia in emergency departments (EDs), the etiology of the hypoglycemia is almost identified. However, about 10% of cases, the etiology of hypoglycemia cannot be determined.

**Methods:** This is a 2-year prospective observational study. A total of 232 patients were transported to our ED with hypoglycemia. After the investigation, a rapid ACTH loading test (synthetic 1-24 ACTH 250 μg iv.) was performed on 21 patients with unexplained hypoglycemia; i.e., 250 μg ACTH was administered intravenously and blood specimens were collected before loading, at 30 min and 60 min after ACTH administration. We adopted a peak serum cortisol level < 18 μg/dl or a delta cortisol of < 9 μ g/dl for the diagnosis of adrenal insufficiency.

**Results:** Among the patients, 163 of 232 (70.3%) were using antidiabetic drugs, 15 (6.5%) were using hypoglycemia-relevant drugs, 12 (5.2%) suffered from digestive absorption failure including malnutrition, 10 (4.3%) had been consuming alcohol, 9 (3.9%) suffered from malignancy, and 2 (0.9%) suffered from insulin autoimmune syndrome. Initially, an etiology was unknown in 21 of 232 (9.1%) patients. Rapid ACTH test revealed the adrenal insufficiency in 19 (8.2%) among them. Administration of hydrocortisone in adrenal insufficiency patients promptly improved hypoglycemia. In those patients, serum sodium level was lower (Na; 134 vs. 139 mEq/l, P<0.001) and serum potassium level was higher (K; 4.7 vs. 3.9 mEq/l, P<0.001) than in the other hypoglycemic patients, respectively. There was no significant difference in baseline plasma glucose level on ED between the groups of patients (28 vs. 26 mg/dl, P=0.34).

**Conclusions:** The probability of adrenal insufficiency was much greater than that of the better-known insulinoma as a cause of hypoglycemia. When protracted hypoglycemia of unknown etiology is recognized, we recommend that the patient is checked for adrenal function using the rapid ACTH loading test.

## P344 Thyroid hormone levels and their prognosis in septic patients

### MV De La Torre-Prados, A García-de la Torre, P Nuevo-Ortega, E Cámara-Sola, T Tsvetanova-Spasova, A Fernández-Porcel, C Rueda-Molina, L Salido-Díaz, I Mateos-Rodríguez, I García-Gómez, AM Sánchez-García

#### Hospital Universitario Virgen de la Victoria, Málaga, Spain

**Introduction:** Sepsis caused have showed serious alternations of thyroid hormones releasing, causing a nonthyroidal illness syndrome. The aim of the study was to measure thyroid hormone levels in septic patients and analyse its relation with clinical state and outcome.

**Methods:** Prospective study in a cohort of 150 consecutive septic patients. We studied thyrotropin (TSH), free triiodothyronine fraction (fT3) and free thyroxin fraction (fT4) serum levels, APACHE II and SOFA score. Statistical analysis was performed using SPSS 15.0.

**Results:** We analysed 150 episodes of sepsis (16%) and septic shock (SSh) (84%), the median age of the patients was 64 (inter-quartile range, 48.7-71) years; the main sources of infection were: respiratory tract (39%) and intra-abdomen (30%); 70.7% had medical diseases. APACHE II score was 25 [21-30], SOFA score was 10 [7.75-11] and 28-day mortality was 22.7%. Our data shown 18.3% with low levels of TSH (<0.2uUI/mL), 20.3% had low levels of fT4 (<0.75 ng/dL) and 71.4% low levels of fT3 (<2 pg/mL). The TSH (0.89 vs. 1.46 uUI/mL) and fT3 (1.3 vs 1.8 pg/ml) concentration of SSh group were significantly lower than those of sepsis group, whereas FT4 (1.10 vs 1.18 ng/dL) it was not statistically significantly. Correlation of FT3 to APACHE II (r = −0.342, p = 0.035) and SOFA score (r = −0.409, p = 0.017). The profile of death patients were men (64.7%, n =22), with significantly older (63 vs. 57 years; p=0,049), as well as clinical severity scores, APACHE II (29.8 vs. 24.1; p<0.001) and SOFA (12.1 vs 8.9; p<0,001). Non-survivors had significantly lower TSH 0.85 vs. 1.4 uUI/mL; p=0.042, and fT3 1.2 vs. 1.39 pg/mL, p=0.031, however fT4 did not show statistical significance 0.42 vs. 0.58ng/dL, p=ns.

**Conclusions:** Conclusions: Most of our septic patients present an altered thyroid function. Our data suggest that TSH and specially fT3 may be used as a marker of disease severity and a mortality predictor.

## P345 Observational study to evaluate short and long-term bone metabolism alteration in critical patients.

### M Cozzolino^1^, G Marcucci^2^, M Andrisani^1^, A Cecchi^1^, A Franci^1^, ML Brandi^2^, A Peris^1^

#### ^1^A.O.U. Careggi, Florence, Italy,^2^University of Florence, Florence, Italy

**Introduction:** Reduction of bone mineral density and/or muscle mass can be short and long-term complications in critical patients admitted in Intensive Care Unit (ICU). The study aims to evaluate, during a 12-month period, the following parameters: 1) the alterations of bone metabolism and quantitative and qualitative parameters of bone tissue, 2) the proportion of subjects with bone fragility, and 3) the identification of specific risk factors.

**Methods:** An observational-longitudinal monocentric study is being conducted in adult patients hospitalized in ICU. The evaluations performed at baseline, 6 and 12 month visits include analysis of biochemical and instrumental exams.

**Results:** A specific clinical-care pathway was created between Bone Metabolic Diseases Unit and ICU, in order to perform specific anamnestic collection, biochemical analysis of bone metabolism, and instrumental exams. 31 patients were enrolled and evaluated at the baseline visit. Biochemical exams, performed within 72 hours of hospitalization, showed that 64% (N:20) of subjects had a deficit of 25OHvitaminD <20 ng/dl, associated with normal corrected serum calcium levels and of these 42% (N:13) had high PTH levels. Bone alkaline phosphatase was increased in 26% (N:8) of patients.

**Conclusions:** Critical patients are “fragile” subjects, which should be monitored with a short and long-term follow-up. The creation of a clinical pathway that includes specialists of bone metabolism may be a virtuous way to identify patients who report bone mass loss and increased fracture risk. This study will allow to implement the knowledge regarding specific risk factors of bone fragility and the most appropriate therapeutic choices as prevention and treatment.

## P346 A retrospective analysis of predictors for length of intensive care stay for patients admitted with diabetic ketoacidosis

### A Fung, TL Samuels, AE Myers, PG Morgan

#### East Surrey Hospital, Redhill, UK

**Introduction:** Diabetic ketoacidosis (DKA) is one of the most common metabolic causes of admission to the intensive care unit (ICU). The incidence of DKA is quoted as between 4.6-8 episodes per 1000 patients with diabetes mellitus (DM) [1]. We aim to establish the factors that affect length of stay (LOS) on ICU.

**Methods:** We undertook an analysis of patients admitted to ICU over the last 7 years with a primary diagnosis of DKA. We assessed whether there was an association between the following factors and an increased length of ICU stay: age, gender, body mass index (BMI), systolic blood pressure, heart rate, sodium, potassium, haemoglobin and pH. These factors were assessed using multiple linear backward stepwise regression.

**Results:** Overall, 94 admissions were identified over the time period from the ward watcher database. The median LOS was 2.4 days (IQR 1.3 – 4.7). Our analysis demonstrated that length of ICU stay (alpha level <0.05) was significantly associated with BMI, low systolic blood pressure, and the presence of hyponatraemia or hypernatraemia.

**Conclusions:** We found the variables that affect the LOS for patients presenting to our unit with DKA are BMI, elow systolic BP, low sodium and high sodium. We intend to extend this work to include survival analysis with the same subgroup of patients.

**References**

1. Savage M., et al., Diabetic Med 28(5):508-515, 2011

## P347 Maximal glycemic gap is the best glycemic variability index correlated to ICU mortality in medical critically ill patients

### T Issarawattna, R Bhurayanontachai

#### Prince of Songkla University, Songkla, Thailand

**Introduction:** Several evidences shown a correlation of glycemic variability (GV) and ICU mortality. However, there have been no report of the correlation between various parameters of GV and mortality in medical ICU patients. The aim was to determine the correlation between various parameters of GV and medical ICU mortality, as well as, to identify the best GV index to predict ICU mortality.

**Methods:** A retrospective chart review was then conducted in medical ICU at Songklanagarind hospital. The patient characteristics, causes of admission, APACHE II, blood glucose within the first 24 hours of ICU admission and ICU mortality were recorded. Glycemic variability parameters including maximal glycemic gap, standard deviation, coefficient of variation and J-index of blood glucose were calculated. The correlation of those GV index to ICU mortality was determined. The ROC and AUROC of each GV index were then compare to identify the best GV index to predict ICU mortality.

**Results:** Of 538 patients, 442 patients (82.2%) were survived (Table 1). All GV indexes were significantly higher in non-survival group (p < 0.05) (Table 2). Maximal glycemic gap was independently correlated to ICU mortality and give a highest AUROC compared to others GV. (Maximal glycemic gap AUROC 0.69 (95%CI 0.64-0.75 vs. coefficient of variation AUROC 0.68 (95%CI 0.62-0.74) vs standard deviation AUROC 0.67 (95%CI 0.61-0.73) vs J-index AUROC 0.63 (95%CI 0.57-0.7), (p< 0.001) (Fig. 1).

**Conclusions:** Maximal glycemic gap independently correlated to ICU mortality and was the best GV to predict ICU mortality in medical critically ill patients.

**Table 1 (abstract P347).** Baseline characteristics, statistic analysis with Chi-square test

Characteristics	Survivor	Non survivor	p-value
Number of patient (n (%))	442 (82.2)	96 (17.8)	
Age (Median, IQR)	65 (51,79)	66 (51,75)	0.77*
Male (n (%))	231 (52.3)	60 (62.5)	0.09
Diabetes mellitus (n (%))	101 (24.9)	20 (20.8)	0.48
APACHE II	18	23	<0.01*
Insulin administration (n (%))	78 (17.6)	26 (27.1)	0.05
Hypoglycemia (n (%))	31 (7.0)	12 (1.5)	0.11

*Mann-Whitney U test

**Table 2 (abstract P347).** Glycemic variability and ICU mortality, statistic analysis with Mann-Whitney U test

Glycemic variability (Median, IQR)	Survivor	Non survivor	p-value
Maximal glycemic gap	66.5 (39.0,113.8)	112 (70.5,177.0)	<0.01
Coefficient of variation	0.2 (0.1,0.3)	0.3 (0.2,0.4)	<0.01
Standard deviation	26.2 (15.8,40.3)	40.3 (25.8,61.5)	<0.01
J - index	27.1 (7.5,177.7)	40.0 (10.2-177.8)	<0.01

**Fig. 1 (abstract P347).**
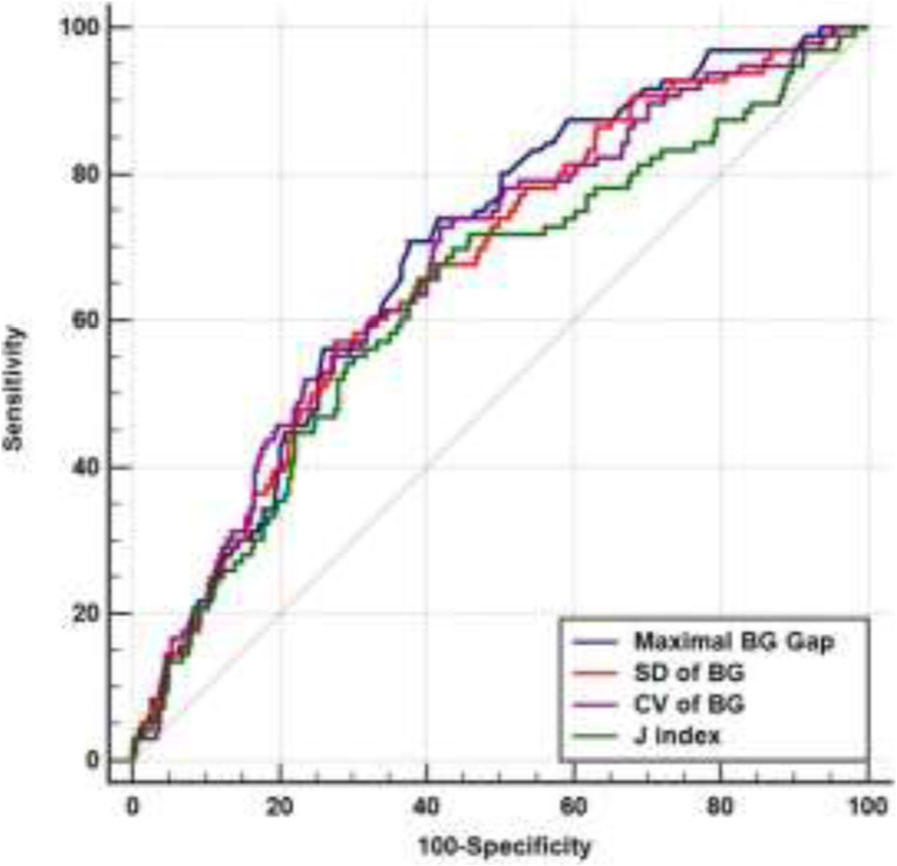
AUROC between glycemic variability and ICU mortality, Maximum BG gap 0.693(0.638-0.747), CV 0.677(0.619-0.736), SD 0.672(0.614-0.730), J-index 0.634(0.571-0.698), All p-value < 0.05

## P348 Reliability of capillary blood glucose measurement for diabetic patients in emergency department

### H Ben Turkia, S Souissi, A Souayeh, I Chermiti, F Riahi, R Jebri, B Chatbri, M Chkir

#### Regional Hospital of Ben Arous, Ben Arous, Tunisia

**Introduction:** Acute glycemic disorders should be early diagnosed and treated in Emergency Department (ED), especially hypoglycemia. Can capillary blood glucose (CG) replace plasmatic glucose (PG). The objective of this study was to compare capillary blood glucose with venous blood glucose

**Methods:** Patients with type 2 diabetes were included. We realize a capillary blood glucose with a glucose meter (acu-check active-Roche) and a concomitant determination of venous blood glucose with laboratory machine (synchrony CX3 delta system beckman coulter). A correlation study (Pearson correlation) between the two measurements was evaluated and linear fitting equation was established. The concordance was checked with Bland and Altman method.

**Results:** During the 4 months of the study, 258 patients were included. The average age was 55+/-19 years old, with a sex ratio =1. Majority of patients (70%,n=182) had type 2 diabetes and 58% was treated with insulin. We found an excellent correlation between the two techniques with a Pearson correlation coefficient r= 0.96.Topredict the PG from CG, we can use this equation: PG(g/l)=0.9979 CG(g/l)+ 0.08128 (R2=0.9207 ; p=0.0001). We noticed a good concordance between the two techniques especially in case of hypoglycemia and moderate hyperglycemia (Fig. 1). However, 11 releases were noted with a PG higher than 4g/l.

**Conclusions:** In ED, the measurement of capillary glucose can exempt from venous blood glucose especially in case of hypoglycemia and moderate hyperglycemia.

**Fig. 1 (abstract P348).**
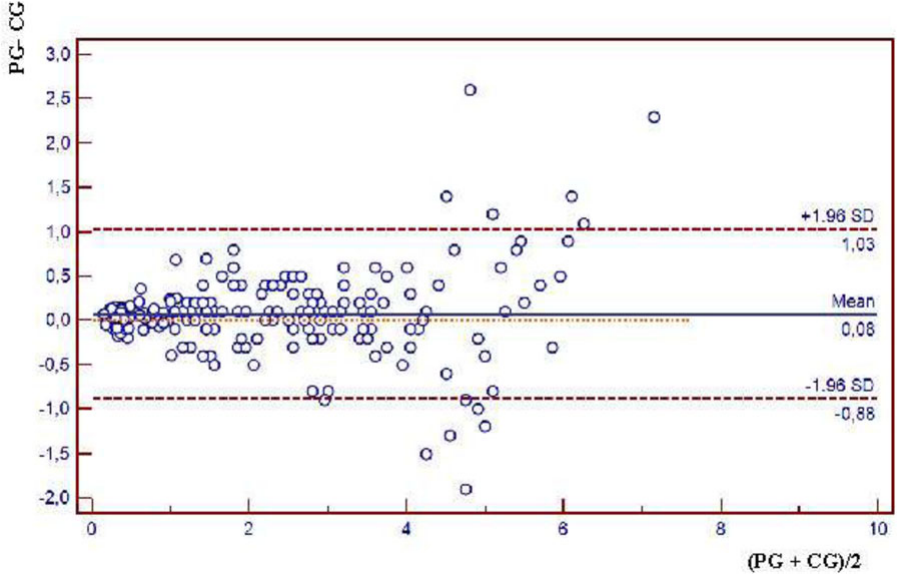
Concordance CG vs PG (Bland and Altman Method)

## P349 Comparison of length of stay and deep vein thrombosis incidents in critically ill patients

### E Hanindito, P Airlangga, S Sulistiawan, N Rehatta

#### Dr. Soetomo Hospital, Surabaya, Indonesia

**Introduction:** Vein thrombosis may occur both in deep and superficial vein of all extremities. Ninety percent of vein thrombosis may progress into pulmonary embolism which is lethal. Deep vein thrombosis (DVT) is frequently found in critically ill patients in ICU, especially patients who are treated for a long time. This study aims to analyse the comparison between length of stay and DVT incidents in critically ill patients.

**Methods:** A cross-sectional study was employed. We include all patients who were 18 years or older and were treated in ICU of Dr Soetomo public hospital for at least 7 days. Data were collected from June 2016 until June 2017. The patients were examined with Sonosite USG to look for any thrombosis in iliac, femoral, popliteal, and tibial veins and Well’s criteria were also taken.

**Results:** Thirty patients were included in this study. This study shows that length of stay is not the only risk factor for DVT in patients treated in ICU. In our data, we found out that the length of treatment did not significantly cause DVT. Other risk factors such as age and comorbidities in patients who are risk factors may support the incidence of DVT events. The diagnosis of DVT is enforced using an ultrasound performed by an expert in the use of ultrasound to locate thrombus in a vein.

**Conclusions:** Length of treatment is not a significant risk factor for DVT. Several other factors still need to be investigated in order for DVT events to be detected early and prevented.

## P350 Newly diagnosed onco-hematologic patients behaviour in the ICU

### M Dos Santos Couto, F Coelho, A Jeronimo, F Faria

#### Instituto Português de Oncologia do Porto, Francisco Gentil, E.P.E., Porto, Portugal

**Introduction:** Onco-hematologic (OH) patients behave differently from the oncologic in ICU, with a more unpredictable disease course impelled by the infectious risk caused by immunosuppression and disease relapse risk.

**Methods:** We reviewed and characterized the newly diagnosed and non-treated OH patients admitted in our ICU between 2011 and 2016 and followed up them until July 2017.

**Results:** A total of 52 patients were included (18% of 289 OH patients admitted, 2,5% of 2.114 patients admitted). The median age was 48 years-old ([10-74]) being 55.8% male. The majority had Non-Hodgkin Lymphoma (NHL, 48.1%), 28.8% Acute Myeloid Leukemia, 13.5% Acute Lymphoblastic Leukemia, 5.8% Hodgkin Lymphoma, 1.9% Chronic Myeloid Leukemia and 1.9% Aplastic Anemia. The median APACHE score was 24.5 and SAPS II 55. The median length of stay was 4 days. Non-invasive ventilation was required for 19.2% (median time of 19.2 hours) and 71.2% were mechanically ventilated (median time of 33.8 hours). Dialysis was necessary in 46.2% and vasopressors in 25%. The overall survival was 63.5% and the probability of discharge from the hospital alive 48%. From the 33 patients who survived, 97% did chemotherapy (completed in 62.5%). In 2017 26.9% of the 52 patients are alive (92.9% with complete response and one other had significant reduction of the BCR-ABL1 fusion gene; one NHL was discharge from consultation). The rest died in progressive disease (50%), septic shock (34.2%), tumor lysis syndrome (5.3%), vascular compressive syndrome (2.6%), pneumonia (2.6%), respiratory insufficiency (2.6%), subarachnoid hemorrhage (2.6%). The median survival after diagnosis was 58 days ([443-957] CI 95%) and the median survival after discharge from ICU was 1389 days ([1087-1690] CI 95%).

**Conclusions:** One-third of newly diagnosed OH patients survived ICU; almost all were treated with chemotherapy afterwards (most of them ending it with durable responses). There are many possible predictors of a better prognosis, which are clarified here.

## P351 Trends in patient characteristics and outcomes of cancer patients admitted to the ICU of a tertiary teaching hospital in the U.S.

### CM Sauer^1^, LA Celi^2^, D Ramazzotti^3^

#### ^1^Harvard School of Public Health, Boston, Massachusetts, USA,^2^Massachusetts Institute of Technology, Cambridge, Massachusetts, USA,^3^Stanford University, Palo Alto, California, USA

**Introduction:** Due to improved critical care and cancer treatments and thus increased cancer-specific survival [1], we hypothesized that both the number of cancer patients admitted to the ICU and overall survival have increased over the last decade.

**Methods:** MIMIC-III, a freely accessible critical care database of Beth Israel Deaconess Medical Center, Boston, USA [2] was used to retrospectively study trends and outcomes of cancer patients admitted to the ICU between 2002 and 2011. Logistic regression analysis was performed to assess predictors of 28-day and 1-year mortality.

**Results:** Out of 41,468 ICU admissions, 1,100 hemato-oncological, 3,953 oncological and 49 patients with both a hematologic and solid malignancy were analyzed. Hematologic patients had higher critical illness scores, while oncological patients had similar APACHE-III and SOFA-scores. In the univariate analysis, cancer was strongly associated with mortality (OR 2.5, Table 1). Over the 10-year study period, 28-day mortality of cancer patients decreased by 30% (Fig. 1). This trend persisted after adjustment for covariates, with cancer patients having significantly higher mortality (OR=2.49, 95%CI: 2.3, 2.7). Between 2002 and 2011, the adjusted 28-day mortality decreased by 8% every year. Over the decade, 1-year mortality decreased by 27%. Having cancer was the strongest individual predictor of 1-year mortality in the multivariate model (OR=4.40, 95%CI: 4.1, 4.8) (Fig. 2).

**Conclusions:** Between 2002 and 2011, the number of cancer patients admitted to the ICU increased steadily and significantly, while longitudinal clinical severity scores remained overall unchanged. Although hematological and oncological patients had higher mortality rates than patients without cancer, both 28-day and 1-year mortality decreased significantly over the study period.

**References**

[1] Jemal A et al., J Natl Cancer Inst, 109, 2017

[2] Johnson AEW et al., Sci Data, 160035, 2016

**Table 1 (abstract P351).** Overview of patient characteristics and outcomes. Patients with cancer had divergent clinical characteristics and higher 28-day and 1-year mortality

Variables	Level	Without Cancer	Hematological malignancy	Solid malignancy	Double malignancy	p-value
Case number		36,466	1,100	3,953	49	
Age at admission (mean (sd))		63.8(17.8)	65.3(16.4)	65.8(13.6)	69.9(12.7)	<0.001
Proportion with DNR (mean (sd))		0.06 (0.24)	0.08(0.27)	0.10(0.29	0.08(0.27)	<0.001
Race (%)	Non-White	10292 (28.3)	249(22.6)	974(24.6)	10(20.4)	<0.001
	White	26074(71.7)	851(77.4)	2979(75.4)	39(79.6)	
Length of stay ICU (mean (sd))		4.59(6.09)	5.38(7.15)	4.19(5.30)	4.00 (3.34)	<0.001
Length of stay Hospital (mean (sd))		9.28 (8.72)	14.71(15.19)	10.05(8.32)	10.98(10.01)	<0.001
Any duration of ventilation (%)	No	18185(50.0)	630(57.3)	2254(57.0)	29(59.2)	<0.001
	Yes	18181(50.0)	369(33.5)	990(25.0)	11(22.4)	
Any use of vasopressors (%)	No	23938(65.8)	731(66.5)	2963(75.0)	38(77.6)	<0.001
	Yes	12428(34.2)	369(33.5)	990(25.0)	11 (22.4)	
Elixhauser SID30 score (mean (sd))		8.76(11.09)	17.57(11.78)	19.41(13.02)	23.88(12.37)	<0.001
SOFA score (mean (sd))		3.27(2.50)	4.56(3.01)	3.32(2.66)	3.39(2.86)	<0.001
APACHE III score (mean (sd))		42.03(19.42)	50.39(22.09)	44.51(20.31)	46.73(23.27)	<0.001
OASIS score (mean (sd))		31.35(8.97)	32.51(8.96)	31.15(9.51)	31.18(9.89)	<0.001
28-day Mortality (%)	Alive	31726(87.2)	795(72.3)	2810(71.1)	46(73.5)	<0.001
	Dead	4640(12.8)	305(27.7)	1143(28.9)	13(26.5)	
1-year Mortality (%)	Alive	27988(77.0)	521(47.4)	1533(38.8)	20(40.8)	<0.001
	Dead	8378(23.0)	579(52.6)	2420(61.2)	29(59.2)	

**Fig. 1 (abstract P351).**
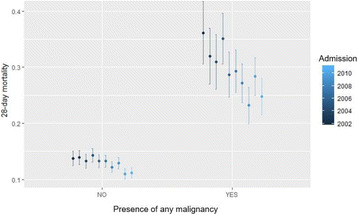
Longitudinal change in 28-day mortality for cancer patients (yes) compared with controls (no) over the 10-year study period. Mortality in the cancer group decreased from 36% to 25% (-30%), while mortality in the control group decreased from 14 to 12% (-21%).

**Fig. 2 (abstract P351).**
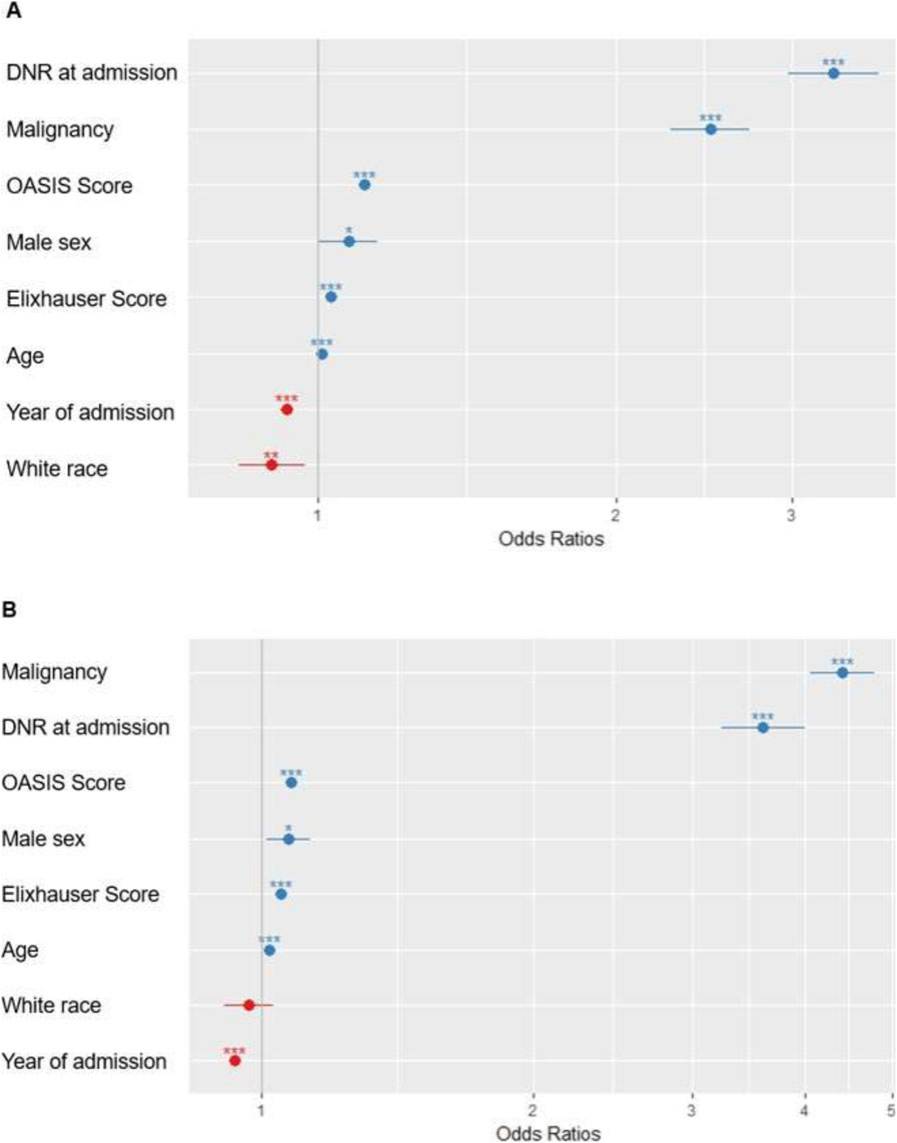
Results of the logistic regression analysis for (A) 28-day and (B) 1-year mortality. All covariates were statistically significant except for white race in the 1-year mortality model. ***p-value<10-16, **p-value<0.001, *p-value<0.01

## P352 Predictive accuracy of sepsis scores in hematological patients

### L Probst, M Kochanek, A Shimabukuro-Vornhagen, M Von Bergwelt, J Vehreschild, J Prinz, B Böll

#### University Hospital Cologne, Cologne, Germany

**Introduction:** Sepsis was redefined in 2016 with the introduction of an increase in Sequential Organ Failure Assessment ∆SOFA) score of >= 2 and the quickSOFA (qSOFA) as screening tools for sepsis-related mortality. However, the implementation of these criteria into clinical practice has been controversial and the applicability for hematological patients is unclear.

**Methods:** We therefore studied the diagnostic accuracy of different sepsis criteria for sepsis and mortality according to definition criteria in a retrospective analysis of hematological patients in an academic tertiary care hospital. Patient characteristics and variables were collected in ICU- and non-ICU patients to determine the Systemic Inflammatory Response Syndrome (SIRS), ∆SOFA and qSOFA. By applying the definition of sepsis as “life-threatening organ dysfunction caused by a dysregulated host response to infection” [1] as reference, the scores were evaluated.

**Results:** Of the analyzed 186 hematological patients, 48 (25.8%) were treated on ICU and 138 (74.2%) on a regular ward of which 24 patients (17.4%) were later transferred to ICU. The median age was 55 years, 60.8% were male and the median length of hospital stay was 14 days.

96/186 (51.6%) had sepsis, of which 88.5% were SIRS-positive, 58.3% SOFA-positive and 18.9% qSOFA-positive. The false positive rate was 78.6% for SIRS, 29.4% for ∆SOFA and 5.3% for qSOFA. The positive predictive value was 53.8% for SIRS, 71.0% for SOFA and 77.8% for qSOFA (Table 1).

In total, 41/186 patients (22.0%) died, of whom 26 (63.4%) had sepsis. In patients with sepsis who died, 5/22 were SIRS-negative, 4/24 ∆SOFA-negative and 14/20 qSOFA-negative (Fig. 1 and Table 2).

**Conclusions:** In conclusion, these findings suggest that criteria proposed in the Sepsis-3 definition might have limitations as screening tools for sepsis-associated mortality in hematological patients and should be studied in larger cohorts.

**References**

1. Singer et al. JAMA 315(8):801-810, 2016

**Table 1 (abstract P352).** Analysis of SIRS, ∆SOFA and qSOFA regarding sensitivity, specificity, positive predictive value (PPV), negative predictive value (NPV) and hazard ratio

Hematological patients (n=186)	SIRS	Δ SOFA	qSOFA
Sensitivity (%)	88.5	58.3	18.9
Specificity (%)	21.4	70.6	94.7
PPV (%)	53.8	71.0	77.8
NPV (%)	64.3	57.8	54.5
Hazard ratio (HR)	0.589	3.258	2.874

**Table 2 (abstract P352).** Predictive performance of SIRS, ∆SOFA and qSOFA for in-hospital mortality in patients with and without sepsis in the hematological cohort

Mortality, No. (%)	Septic patients (n=96)	Non-septic patients (n=90)
SIRS ≥ 2	17 (22.1), n=77	10 (15.2), n=66
SIRS < 2	5 (50.0), n=10	4 (22.2), n=18
∆ SOFA ≥ 2	20 (40.8), n=49	7 (35.0), n=20
∆ SOFA < 2	4 (11.4), n=35	6 (12.5), n=48
qSOFA ≥ 2	6 (42.9), n=14	3 (75.0), n=4
qSOFA < 2	14 (23.3), n=60	9 (12.5), n=72

**Fig. 1 (abstract P352).**
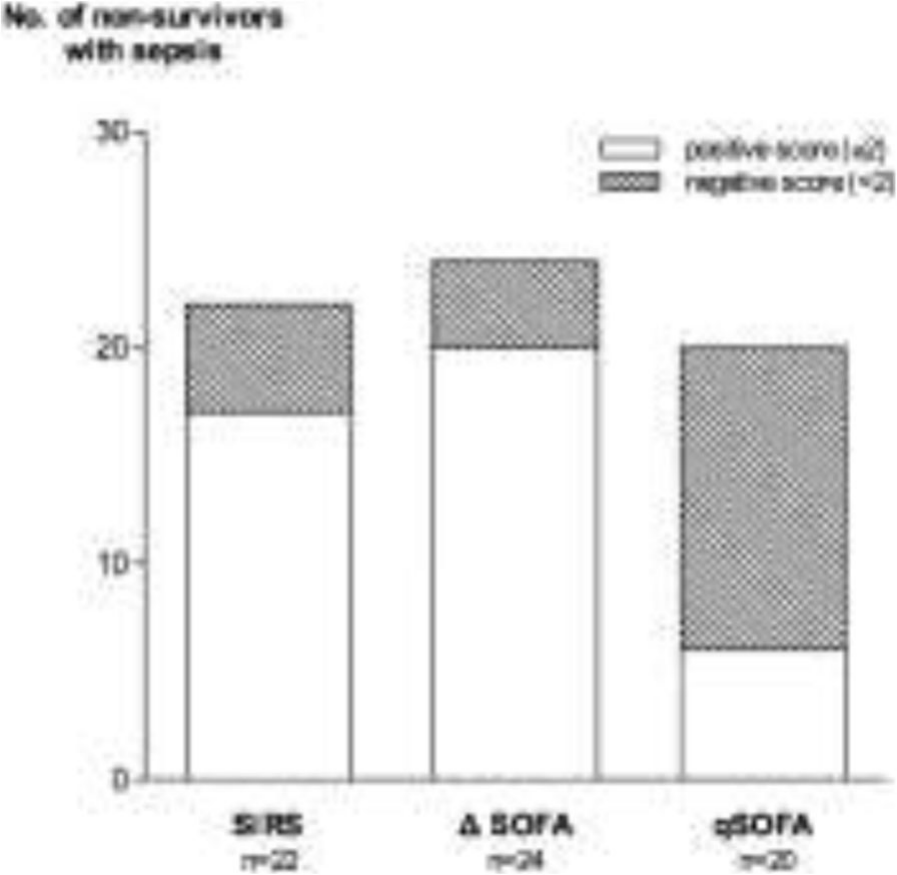
Number of non-survivors with sepsis that did not fulfill the sepsis criteria of SIRS, ∆SOFA or qSOFA

## P353 Enoxaparin pharmacokinetics in patients with augmented renal clearance, preliminary results of a single center study

### A Ramos^1^, A Dogliotti^2^, N Pires^1^, C Lovesio^1^, D Latasa^1^, M Perezlindo^1^, F Acharta^1^

#### ^1^Sanatorio Parque, Rosario, Argentina,^2^Grupo Oroño, Rosario, Argentina

**Introduction:** Augmented renal clearance (ARC) has being described in some groups of critically ill patients. The aim was to investigate the impact of ARC on the pharmacokinetics of enoxaparin.

**Methods:** This is a prospective study in a surgical and medical intensive care unit (ICU) carried out from August to November 2017. Patients <65 years old, under prophylactic treatment with enoxaparin and normal plasma creatinine, were included. Anti-Xa activity was measured at second day under treatment. Creatinine clearance was calculated from urine sample collected during 24-hours. ARC was defined by a creatinine clearance >=130 mL/min/1.73 m2.

**Results:** Thirteen patients aged 43 years old (±16.4) were included. Six patients developed ARC and 5 of them were in therapeutic range. Seven patients did not develop ARC and 6 of them were in therapeutic range. There was no differences between the two groups in achieving therapeutic range (Fisher test, p=0.5). We did not observe thromboembolic events.

**Conclusions:** We found no relationship between ARC and therapeutic failure in patients under prophylactic treatment with enoxaparin.

## P354 Retrospective review of argatroban use and dosing in critical care

### T Freeman, S Khorsid, A Retter

#### St Thomas’ Hospital, London, UK

**Introduction:** This study reviewed argatroban use in patients in a tertiary hospital critical care unit. Argatroban is a direct thrombin inhibitor approved for use in proven or suspected heparin-induced thrombocytopenia (HIT) in patients with renal dysfunction.

**Methods:** This was a retrospective cohort study in a medical and surgical ICU in a tertiary teaching hospital. Data was collected for adult patients treated with argatroban for proven or suspected HIT April-August 2016, excluding patients requiring ECMO. We scored patients using the 4T score and compared this to an ELISA immunoassay optical density score which quantifies the PF4/H antibody level. Also noted was use of continuous haemodialysis and organ failure using the Sequential Organ Failure Assessment (SOFA), scoring >=3 defines failure.

**Results:** 16 patients were treated with argatroban for proven or suspected HIT. 15/16 patients had a positive ELISA. There was no relationship between 4T score and ELISA optical density (Fig. 1). Infusions were commenced at either the manufacturer recommended dose of 2 μg/kg/min or a reduced dose of 0.5 μg/kg/min. Patients receiving the reduced dose had a median of 2 organs failing compared to 1 in the standard regimen. The time taken to the first APTR in range was longer with the reduced dose regimen, however, the time to a stable APTR was less (Table 1). In 2 patients the dose of argatroban never stabilised. 1 died and 1 was very sensitive to argatroban and required cessation of the infusion for interventions. In the reduced regimen group, there were 2 episodes of bleeding, 1 minor PR bleed in a patient with 3 organs failure and 1 upper GI bleed.

**Conclusions:** In this population of ICU patients the 4T score did not correlate with the ELISA optical density score, as found previously. Patients with multi-organ failure mostly received the reduced starting dose. However, the bleeding events were still confined to this group. This correlates with previous studies that organ dysfunction necessitates a dose reduction for argatroban.

**Table 1 (abstract P354).** Argatroban dosing and bleeding rate

	Standard Regimen (n=5)	Reduced dose regimen (n=11)
Argatroban dose at stabilisation (mcg/kg/min)	0.59	1.06
Median time to first APTR in range (hours)	2	7
Median Time to stable APTR (hours)	23	16
Episodes of bleeding	0	2

**Fig. 1 (abstract P354).**
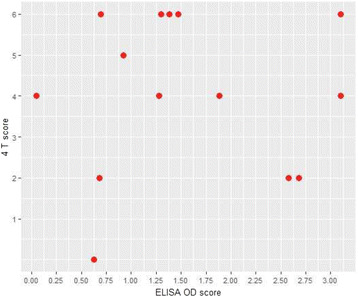
4T score plotted against ELISA optical density score

## P355 Haemostatic effects of therapeutic plasma-exchange in non-coagulopathic patients

### M Popescu, D Tomescu

#### Fundeni Clinical Institute, Bucharest, Romania

**Introduction:** Therapeutic plasma-exchange (TPE) represents the treatment of choice in many immune pathologies. The aim of this study was to assess the effect of TPE on coagulation in normal subjects.

**Methods:** We prospectively included 20 patients who underwent TPE with 65 ml/kg fresh frozen plasma for Myasthenia gravis. Patients with pre-existing coagulation disorders, pro-haemostatic or anti-haemostatic treatment were excluded. Standard coagulation tests (activated partial thromboplastin time – aPTT, prothrombin time – PT, International Normalized Ratio - INR), fibrinogen levels, platelet count and rotational thromboelastometry (ROTEM) were performed before and after each session. Three ROTEM tests were performed: ExTEM, InTEM and FibTEM. For each test the following parameters were recorded: clotting time (CT), clot formation time (CFT), maximum clot firmness (MCF), alpha angle, thrombin potential index (TPI), maximum velocity of clot formation (MaxV), time to MaxV (MaxVt) and area under the curve (AUC).

**Results:** The mean age in our study group was 54±22 years. The effects of TPE on standard coagulation were increased aPTT (24±2 to 36 ± 6 s, p=0.005) and decreased fibrinogen levels (286±76 to 242±48 mg/dL, p=0.008). A non-significant decrease in platelet count was observed (160333±23091 to 151133±22244/mm_3_, p=0.662). On ROTEM parameters TPE was associated with increased CT in ExTEM (57±8 to 73±12 s, p=0.030) and InTEM (156±15 to 194 ±52 s, p=0.003) and increased MaxVt on ExTEM (90± 27 to 128 ± 37 s, p=0.031) and InTEM (177±17 to 225±71 s, p=0.003). All other ROTEM parameters changed non-significantly. The decrease observed in fibrinogen levels was not associated with a decrease in FibTEM MCF (15±2 to 14±2 mm, p=0.414).

**Conclusions:** Our results demonstrate that TPE is associated with minimum changes in clot kinetics initiation that do not result in either pro- or anti- coagulant changes. Therefore, TPE with fresh frozen plasma can be safely used in normal subjects.

## P356 Anaemia in the ICU - impact of phlebotomy

### M Mackovic, N Maric, N Udiljak, M Bakula

#### Clinical Hospital Sveti Duh, Zagreb, Croatia

**Introduction:** Acutely ill patients are prone to critical illness anaemia, a multifactorial condition with potential contribution of iatrogenic anaemia defined as lowered Hb due to large/frequent venepunctions. Decline in Hb is most pronounced in the first 3 days of ICU stay. It correlates with the need for RBC transfusion, but the impact on patient outcome is uncertain. The aim of this study was to determine impact of phlebotomy on change in Hb (ΔHb), and correlation of ΔHb with need for transfusion, presence of central venous catheter (CVC) and patient outcome.

**Methods:** Single-center, prospective cohort study enrolling 202 patients during 3 months was performed in a medical ICU at Clinical Hospital Sveti Duh, Zagreb, Croatia. E, Hct and Hb values were obtained on day 1 and 7. Presence of CVC, need for RBC transfusion and outcome were recorded. Primary outcome was overall survival (OS).

**Results:** Although no significant association of the ΔHb with OS (OR=0.99; 95% CI 0.97-1.00; p=0.238) was observed, both baseline and day 7 Hb were significantly associated with OS (OR=1.01; 95% CI 1.00-1.02; p=0.049; OR=1.05; 95% CI 1.02-1.08; p‹0.001). CVC presence was associated with more pronounced ΔHb (11% compared to 6% without CVC; Mann-Whitney test; p=0.018). Total volume of extracted blood was significant predictor of transfusion need (OR=1.01; 95% CI 1.01-1.01; p‹0.001). Hb, E and Htc significantly decreased during 7 days. Median Hb lowered for 11% (p=0.001). Percentage of patients with Hb level below the anaemia threshold increased for 26 percentage points.

**Conclusions:** Critical illness anaemia is an unexplained phenomenon. Impact of phlebotomy is hard to unequivocally determine since there are many confounders. The change in Hb levels during ICU stay correlates with the need for transfusion that could cause immunomodulation and potentially adverse outcome. Every effort should be made to maintain adequate Hb levels and lower the risk of iatrogenic anemia.

## P357 Red blood cell transfusion in critically ill patients with traumatic brain injury: an international survey of physicians’ attitudes

### P Lessard Bonaventure, F Lauzier, A Boutin, M Schemilt, R Zarychanski, M Saxena, P Zolfaghari, D Griesdale, D Menon, S Stanworth, S English, M Chassé, D Fergusson, A Turgeon

#### CHU de Québec-Université Laval, Québec, Canada

**Introduction:** Anemia is prevalent in critically ill traumatic brain injury (TBI) patients and red blood cell (RBC) transfusions are often required. Over the years, restrictive transfusion strategies have been advocated in the general critically ill population. However, considerable uncertainty exists regarding optimal transfusion thresholds in critically ill TBI patients due to the susceptibility of the injured brain to hypoxemic damages.

**Methods:** We conducted an electronic self-administered survey targeting all intensivists and neurosurgeons from Canada, Australia and the United Kingdom working caring for TBI patients. The questionnaire was developed using a structured process of domains/items generation and reduction with a panel of experts. It was validated for clinical sensibility, reliability and content validity.

**Results:** The response rate was 28.6% (217/760). When presented with a scenario of a young patient with severe TBI, a wide range of transfusion practices was noted among respondents, with 47% favoring RBC transfusion at a hemoglobin level of 7g/dL or less in the acute phase of care, while 73% would use this trigger in the plateau phase. Multiple trauma, neuromonitoring data, hemorrhagic shock and planned surgeries were the most important factors thought to influence the need for transfusion. The level of evidence was the main reason mentioned to explain the uncertainty regarding RBC transfusion strategies.

**Conclusions:** In critically ill TBI patients, transfusion practices and hemoglobin thresholds for transfusion are said to be influenced by patients’ characteristics and the use of neuromonitoring in critical care physicians and neurosurgeons from Canada, Australia and the UK. Equipoise regarding optimal transfusion strategy is manifest, mainly attributed to lack of clear evidences and clinical guidelines

## P358 A retrospective audit of massive transfusion practices at a tertiary metropolitan hospital

### S Pawar, TC Chirakija, KD Deshpande

#### St George Hospital, Sydney, NSW, Australia

**Introduction:** The evidence suggests favourable effects of blood transfusion with high plasma and platelet to RBC ratios on the outcomes of patients receiving massive transfusion (MT).The adherence to the recommendation and its impact on the clinical outcome has not been described at our facility.

**Methods:** This was a retrospective cohort study involving patients who received MT prior to or during ICU stay from January 2009 to December 2012 at St George Hospital. Data were collected from Electronic Medical Records. Appropriate descriptive statistics were used to describe patients’ baseline characteristics. A multiple logistic regression analysis was performed to determine predictors of mortality.

**Results:** A total of 487 patients (mean age 57.6±17.4 years, 59% males, mean APACHE II score 19.3±8.2) were included in the analysis. The main sources of admission were the operating theatre (74.4%) and emergency department (12.1%). Of the 445 patients who underwent surgery 248 (55.7%) were elective and 197 (44.3%) were an emergency. Mean length stay for ICU and hospital were 2.7 and 23 days respectively. Median duration of ventilation was 21 (13-77) hours. No patient received transfusion with blood products using the RBC:FFP:Platelets ratio of 1:1:1 . Compliance with 1:1 ratio of FFP:RBC and Platelets:RBC was 57.3% and 19.6% respectively. The overall mortality rates were 16.2% (ICU), 18.2% (28-day), and 22.7% (1-year). No significant associations were found between FFP:RBC ratio and mortality rates. Patients with higher APACHE II score received more platelet transfusions and mortality rates were higher in those who received Platelets:RBC ratio >1. On multivariate analysis, higher APACHE II score was an independent predictor of increased mortality.

**Conclusions:** The compliance with the recommended 1:1:1 ratio of blood products was poor. There was no association between transfusion ratios and mortality after adjusting for APACHE II score.

## P359 Changing trends in transfusion practice during first-time coronary artery bypass grafting over 15 years (2002-2017)

### H Cauwenberghs, H Day, L Kuppurao

#### Royal Brompton and Harefield Hospital NHS Foundation Trust. Harefield Hospital London, Harefield, UK

**Introduction:** The lack of evidence-based medicine supporting the transfusion decision is illustrated by the wide range of blood product use during first-time coronary artery bypass grafting (CABG). Use of red blood cells (RBC) ranges from 3 to 83 percent, while the use of platelets range from 0 to 40 [1]. Approximately 20 percent of CABG patients suffer abnormal bleeding, with platelet dysfunction thought to be the most common culprit [2].

**Methods:** The objective of this study was to evaluate the use of allogeneic blood and blood products among patients undergoing first-time CABG over the past 15years. The first 50 patients who underwent CABG (on-pump and off-pump) from 1 st of March each year were included for analysis. The percentage of patients receiving RBC, fresh frozen plasma (FFP), platelet and cryoprecipitate during the first 48 hours intra- and postoperatively were analysed. Linear regression analysis was performed in each group.

**Results:** Our analysis shows that the use of RBC decreased over the last 15 years, in contrast to the use of the other 3 investigated products. (see Fig. 1) The increase of platelets was the most pronounced with a direction coefficient of 0.022 and had the least variability (r^2^=0.59). (see Fig. 2) The decrease in RBC was less obvious than the rise in platelet use (direction coefficient of 0.015) and had a higher variability (r^2^=0.32). The consumption of FFP and cryoprecipitate stayed constant (direction coefficient of 0.004 and 0.001 respectively).

**Conclusions:** The higher incidence of semi-urgent CABG in recent years, which involves continuation of anti-platelet therapy until the day before surgery, can be an explanation for our observed increased use of platelets. The observed decrease in RBC transfusion over the past 15 years might be due to rising awareness of complications associated with red cell transfusion.

**References**

1. Stover EP et al. Anesthesiology 88:327-33, 1998

2. Kytola L et al. Acta Anaesthesiol Scand 42:178-83, 1998

**Fig. 1 (abstract P359).**
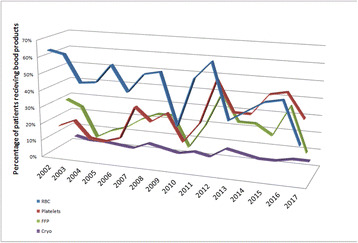
Blood and blood component usage in first time CABG

**Fig. 2 (abstract P359).**
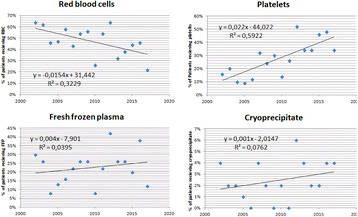
Regression analysis for each blood product over time

## P360 Predictors of red blood cell transfusion in cardiac surgery patients

### D Ringaitiene, L Puodziukaite, V Vicka, D Gineityte, J Sipylaite

#### Department of Anesthesiology and Intensive Care, Institute of Clinical Medicine, Faculty of Medicine, Vilnius University, Vilnius, Lithuania

**Introduction:** Red blood cells (RBC) transfusion is frequently required in cardiac surgery and is associated with increased morbidity and mortality rates. The aim of this study is to identify predictors of RBC transfusion for patients undergoing cardiac surgery, emphasizing the use of bioelectrical impedance analysis (BIA).

**Methods:** This was a retrospective study of patients who underwent elective cardiac surgery between years 2013 and 2014 in a tertiary reference center. Patients’ demographic and clinical variables, preoperative BIA measurements and postoperative data were analyzed. The univariate and multivariate logistic regression analyses were used to identify the predictors of postoperative RBC transfusion. All of the calculations were performed with IBM SPSS v. 24.

**Results:** Among 642 patients included (67.8% males, median age 66 [range, 59 - 73]), 210 (32.7%) of them received at least one unit of RBC postoperatively. Median number of units transfused was 2 [range, 2 - 4]. Hypertension, stroke, renal failure, preoperative hemoglobin and hematocrit values, BIA provided phase angle, aorta clamp time and cardiopulmonary bypass (CPB) time were associated with risk of RBC transfusion and were included in the final model of multivariate regression analysis. Preoperative stroke (OR=0.394, CI95%: 0.183-0.848, p=0.017), preoperative hemoglobin (OR=0.943, CI95%: 0.928-0.960, p<0.001), low phase angle (OR=0.430, CI95%: 0.250-0.740, p=0.002) and CPB time (OR=1.013, CI95%: 1.008-1.018, p<0.001) were identified as independent predictors.

**Conclusions:** Several factors were identified to be significantly associated with postoperative RBC transfusion in patients undergoing cardiac surgery. Among the conventional predictors a value of the BIA provided phase angle was indicated as a potent tool. Further analysis of clinical benefits of these findings is needed.

## P361 Influence of postoperative red blood cell transfusion on late mortality in cardiac surgery patients

### D Ringaitiene, L Puodziukaite, V Vicka, D Gineityte, J Sipylaite

#### Department of Anesthesiology and Intensive Care, Institute of Clinical Medicine, Faculty of Medicine, Vilnius University, Vilnius, Lithuania

**Introduction:** Red blood cells (RBC) transfusion is a common intervention in cardiac surgery and is associated with higher mortality rates and predisposes serious adverse events. The aim of this study was to determine whether red blood cells (RBC) transfusion is linked to long-term results after cardiac surgery.

**Methods:** This observational retrospective study included all of the patients who underwent any of the STS defined elective cardiac surgery types from 2013 to 2014. We evaluated 3-5 year all-cause mortality rates and secondary postoperative outcomes defined by the STS risk prediction model. Patients were categorized according to whether they received RBC transfusions postoperatively; long-term results were compared using Cox-regression analysis and Kaplan-Meier method.

**Results:** The overall rate of postoperative RBC transfusion for the study cohort of patients (67.8% males, median age 66 years [range, 59 - 73]) was 32.7% (n=210). Long term mortality rate was 12.3% (n=79), providing Kaplan-Meier mean survival estimates of 50.4 months in RBC group vs. 63.4 months in no RBC group (p=0.002). In a multivariate cox regression analysis adjusted for the preoperative EuroSCORE II value RBC transfusion remained as an independent predictor of mortality, OR=1.88 CI95%: 1.15-3.10, p=0.013. Secondary clinical outcomes analysis of STS outcomes revealed a higher rate of prolonged ventilation (22.8% vs. 3.1%, p<0.001), deep sternal wound infection (1.4% vs. 1.6%, p=1.000), renal failure (11.9% vs. 0.5%, p<0.001), stroke (5.2% vs. 1.2%, p=0.005) and surgical re-exploration (18.3% vs. 1.4%, p<0.001) in RBC group.

**Conclusions:** RBC transfusions are associated with higher mortality and morbidity rates after cardiac surgery. Further studies are needed to determine whether these assumptions are based by the postoperative course of the patients.

## P362 Blood transfusions in the intensive care setting: an audit of prescribing and administering practices

### N Kalyal, C Ward, A Ankuli, T Bolonenkova, A Molokhia

#### Lewisham and Greenwich NHS Trust, London, UK

**Introduction:** Transfusion of packed red cells (PRCs) is an important treatment option for patients requiring intensive care but, like all treatments, it is not without risk. These patients, although may be more sensitive to anaemia, are also at increased risk of transfusion-related complications. We conducted an audit of blood prescribing and administering practices in our intensive care unit.

**Methods:** Audit proformas were placed in blood prescribing forms for a 1-month period. All transfusions of PRCs were logged over this time, and transfusion triggers, post-transfusion Haemoglobin (Hb) and whether Hb was checked between units was recorded, in addition to other supplementary information.

**Results:** Over a 1-month period, 25 transfusion events were recorded, with an average age of the transfused patients of 60 years old (range 35 - 87 years). 76% of transfusion events were for low Hb, 8% for bleeding and in 16% of cases the indication was not documented. For patients transfused for a low Hb, the mean transfusion trigger was 75 g/L (range: 66 g/L – 86 g/L). Only 12% had a transfusion trigger of 70g/L or less, and a further 12% who were transfused for a low Hb had a Hb of 80g/L or more. 36% of transfusion events involved transfusing 2 or more units and, in only 22% of these cases the Hb was checked between units. Excluding the two bleeding patients, the mean increase in Hb following a single unit transfusion was 11.4 g/L (range 2 g/L – 18 g/L), whilst in patients transfused two units, the average increase in Hb was 10 g/L per unit transfused (range 7 g/L – 14.5 g/L), suggesting single unit transfusions may have greater Hb yields.

**Conclusions:** Our audit demonstrated variability in transfusion triggers and progress needed with administering practices when transfusing multiple units of blood in the non-bleeding patient. We have since implemented measures to meet guidelines in both prescribing PRCs with restrictive triggers and in the administration and assessment of Hb between units, and will be re-auditing.

## P363 Thromboelastometry versus standard coagulation tests versus restrictive protocol to guide blood transfusion prior to central venous catheterization in cirrhosis: a randomized controlled trial

### LL Rocha, A Serpa Neto, CM Pessoa, RF Chaves, T Crochemore, E Silva, LR Ferraz, TD Correa

#### Hospital Israelita Albert Einstein, Sao Paulo, Brazil

**Introduction:** There is a perceived increased risk of bleeding in cirrhosis patients undergoing invasive procedures. This lead to a high rate of empirical prophylactic transfusion, which has been associated to increased complications and cost. The best strategy to guide transfusion in these patients remains unclear. Our aim was to compare three strategies to guide blood component transfusion prior to central venous catheterization (CVC) in critically ill cirrhosis patients.

**Methods:** Single center, randomized, double-blinded, controlled clinical trial conducted in Brazil [1]. All cirrhosis patients admitted to the ICU with indication for a CVC were eligible. Participants were randomized 1:1:1 to three transfusion strategies based on: (1) standard coagulation tests (SCT), (2) rotational thromboelastometry (ROTEM) and (3) restrictive. The primary outcome was proportion of transfusion of any blood component prior to CVC. Secondary outcomes were incidence of major and minor bleeding, ICU length of stay (LOS), and 28-day mortality. Analysis was intention-to-treat.

**Results:** 57 participants (19 in each group) were enrolled between September 2014 and December 2016. Most were male (64.9%) and listed for liver transplantation. The study ended after reaching efficacy in first interim analysis. There was no significant difference in baseline characteristics among groups. Regarding primary endpoint, there was 14 (73.7%), 13 (68.4%), and 3 (15.8%) events in SCT, ROTEM and restrictive groups, respectively (p <0.001). There was no difference between SCT and ROTEM groups (p >0.99). Overall 28-day mortality was 33.3% and was similar between groups. ICU LOS did not differ between groups. There was no major bleeding. Overall minor bleeding occurred in 10.53% with no difference between groups.

**Conclusions:** A restrictive strategy is safe and effective in reducing the need of blood component transfusion prior to CVC in critically ill cirrhosis patients. A ROTEM-based strategy was no different from transfusion guided by SCT.

**References**

1. Rocha LL et al. Trials 18:85, 2017

## P364 Prophylactic fibrinogen concentrate reduces postoperative bleeding in pediatric cardiac surgery: a randomized clinical trial

### L Lima, L Hajjar, L Camara, F Ferreira, L Galas, M Sundin, J Jardim, F Galas

#### Heart Institution, Sao Paulo, Brazil

**Introduction:** Bleeding is a common complication during and after pediatric cardiac surgery, with acquired hypofibrinogenemia being the most associated disorder. This trial evaluated whether the use of prophylactic fibrinogen concentrate reduces bleeding and the requirement of allogeneic blood transfusion after pediatric cardiac surgery.

**Methods:** A clinical randomized study with children undergoing cardiac surgery. Inclusion criteria: Cardiac surgery with cardiopulmonary bypass, age under 28 days or RACHS 1 >= 3 or reoperation with age under 10 years and FIBTEM®-A10 less than 15 mm at thromboelastometry. Patients were randomized 1:1 to treatment group [fibrinogen concentrate according to the formula (15 - A10 (mm) x body weight(Kg)/140)] or control group (saline 0.9%). Outcomes were postoperative bleeding and the number of transfused units.

**Results:** In these preliminary results, 30 patients were analyzed; 14 (46.7%) patients were allocated in the fibrinogen concentrate group and 16 (53.3%) patients in the control group. Fibrinogen concentrate patients had lower total blood drainage volume compared to the control group (125 vs. 244 ml, p= 0.042). There was no difference between groups regarding intraoperative and postoperative blood transfusion. Fibrinogen levels analyzed by FIBTEM were similar between the groups at the end of CPB (6 vs. 7 mm, p= 0.292). Patients receiving fibrinogen concentrate had a significant increase in fibrinogen levels after infusion of the study solution (10 vs. 8 mm, p= 0.059, 204 vs. 160 mg/dL, p= 0.009).

**Conclusions:** These preliminary findings suggest that prophylactic use of fibrinogen concentrate reduced postoperative bleeding in children undergoing cardiac surgery. There was no significant difference in the need of blood transfusion between groups.

## P365 The effect of intravenous desmopressin in coagulation profile of patients undergoing valve surgery: a randomized clinical study

### G Santana, L Hajjar, L Camara, M Piccioni, L Galas, F Galas

#### Heart Institution, Sao Paulo, Brazil

**Introduction:** Desmopressin (DDAVP) is a vasopressin analogue which improves platelet function. Its general use as a haemostatic agent is still controversial. The aim of study was to evaluate the effect of prophylactic desmopressin in blood coagulation in patients undergoing heart valve surgery.

**Methods:** Prospective, randomized, double-blind clinical trial performed at the Heart Institute of the University of São Paulo. A total of 108 adult patients undergoing heart valve surgery were enrolled from February 2015 to November 2016. Immediately after cardiopulmonary bypass weaning and heparin reversal, patients were randomized in ratio 1:1 to intervention group: DDAVP (0.3 μg/kg) or control group. Blood samples were drawn at three different times, at baseline (T0), 2 hours (T1) and 24 hours (T2) after study medication. Blood coagulation and perioperative bleeding were analysed using laboratorial tests and thromboelastometry, chest tube drainage and requirement of allogenic transfusion within 48 hours.

**Results:** A total of 54 patients were allocated to intervention and 54 to control group. Blood levels of factor VIII at T2 (236.5 ± 62.9 vs. 232.3 ± 66.7, P=0.015) and prothrombin time [14.1 (12.9 - 15.2) vs. 13.4 (12.1 - 14.5), P=0.007] were significantly higher in desmopressin group. Standard coagulation tests, as well as viscoelastic and platelet aggregation tests, were similar between groups. There was no difference in postoperative drainage and blood transfusion [10% (21.3) vs. 15%, P=0.526] between desmopressin and control group.

**Conclusions:** Prophylactic use of desmopressin in heart valve surgery does not influence coagulation and thromboelastometric parameters.

## P366 Identifying the impact of hemostatic resuscitation on development of multiple organ failure using factor analysis: results from a randomized trial using first-line coagulation factor concentrates or fresh-frozen plasma in major trauma (RETIC study)

### P Innerhofer^1^, D Fries^1^, M Mittermayr^1^, N Innerhofer^1^, D Von Langen^1^, T Hell^2^, P Würtinger^3^, E Oswald^1^

#### ^1^Medical University Innsbruck, Innsbruck, Austria,^2^University of Innsbruck, Innsbruck, Austria,^3^University Hospital Innsbruck, Innsbruck, Austria

**Introduction:** to clarify how hemostatic resuscitation affects occurrence of multiple organ failure.

**Methods:** analysis of secondary endpoints of the RETIC study [1] (coagulation factors, activated protein C (APC), thrombin generation, ROTEM parameters, syndecan-1, thrombomodulin (TM) and D-Dimer) measured at randomization, and after patients had received FFP or coagulation factor concentrates (CFC) at admission to ICU, 24 and 48 hours thereafter. We used factor analysis to reduce the highly interrelated variables to a few main underlying factors and analysed their relation to MOF before and after hemostatic therapy.

**Results:** The factors Concentration, Clot and Hypoperfusion representing trauma-induced coagulopathy (Table 1) were comparable between groups at baseline (Fig. 1) and only high Hypoperfusion-score predicted MOF, while after therapy a low Clot-score also predicted MOF. Only the changes of the Clot-score independently affected occurrence of MOF (p=0.0002, adjusted OR 3.40, CI 2.46- 4.71), while changes of Concentration (p= 0.8979, adjusted OR 0.96, CI 0.68-1.34) and Hypoperfusion (p=0.8098, adjusted OR 1.06, CI 0.84-1.33) did not. A lower Clot-score occurred after FFP transfusion than use of CFC, mainly through persistent thrombocytopenia (platelet count R2-4 FFP vs CFC p<0.02) (Fig. 2). The higher Concentration-score after FFP did not affect MOF and FFP had no beneficial effect on fibrinolysis, syndecan-1, TM or APC.

**Conclusions:** Hemostatic resuscitation should augment the factor Clot, which is feasible with early fibrinogen administration but not with FFP. The found platelet-saving effect of early fibrinogen administration is important as platelets play a major role in inflammation and transfusion of platelets did not correct thrombocytopenia.

**References**

1. Innerhofer P et al. Lancet Haematol 4(6):e258-e271, 2017

**Table 1 (abstract P366).** The loading of a variable on one or more of the three factors corresponds to the correlation of that variable and the factor

	Concentration	Clot	Hypoperfusion
coagulation factors	0.56 to 0.91		
Fibrinogen	0.69	0.31	
Platelets	0.55	0.34	
ROTEM		-0.37 to 0.97	
APC			0.52
Syndecan-1			0.96
Thrombomodulin	0.39		0.31

Coagulation factors = concentrations of coagulation factors FII, FV, FVII, FVIII, FIX, FX, FXI, FXIII antithrombin, protein C, thrombin peak. ROTEM parameters = extrinsically activated coagulation time, alpha angle and and clot firmness at 10 min, fibrin polymerization at 10 min.

**Fig. 1 (abstract P366).**
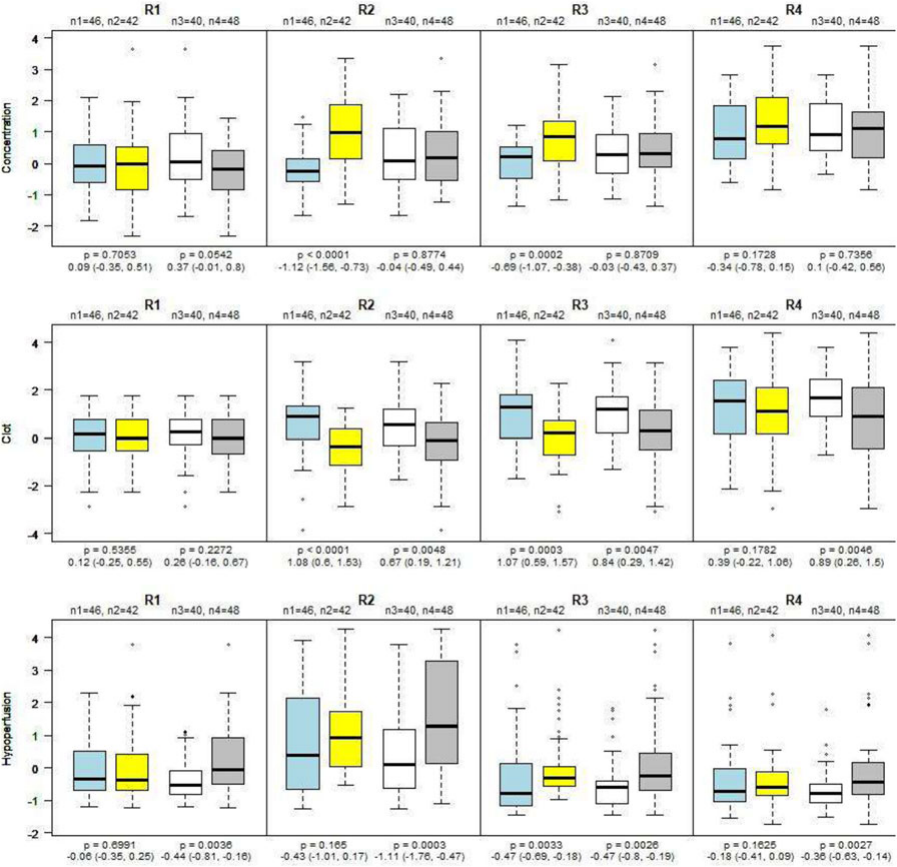
Boxplots show the factors Concentration, Clot and Hypoperfusion, extracted from the performed factor analysis (see Table 1) for the CFC (blue, n=46) and the FFP (yellow, n=42) group, as well as for patients without (white, n=40) and with (grey, n=48) multiple organ failure. Each factor is given at the measurement time point baseline (R1) and following haemostatic resuscitation at admission to ICU, 24 and 48 hours thereafter (R2 to R4).

**Fig. 2 (abstract P366).**
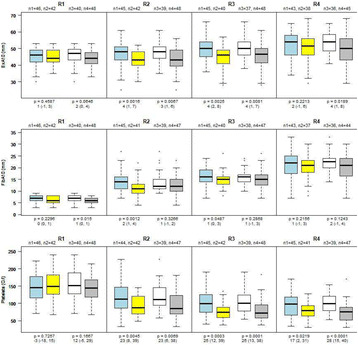
Boxplots show available measurements of extrinsically activated clot firmness at 10 min (ExA10), fibrin polymerization at 10 min (FibA10) and platelet count at baseline (R1) and after therapy at admission to ICU, 24 and 48 hours thereafter (R2 to R4) for the CFC (blue, n=46) and the FFP (yellow, n=42) group as well as for patients without (white, n=40) and with (grey, n=48) multiple organ failure.

## P367 Early prediction of the need for emergent blood product transfusion in trauma: can we do better ?

### F Swerts^1^, PY Mathonet^1^, A Ghuysen^1^, V D’Orio^1^, JM Minon^2^, M Tonglet^1^

#### ^1^CHU de Liège, Liège, Belgium,^2^CHR de la Citadelle, Liège, Belgium

**Introduction:** The Trauma Induced Coagulopathy Clinical Score (TICCS) was developed to be calculable on the site of injury with the objective to discriminate between trauma patients with or without the need for Damage Control Resuscitation (DCR) and thus transfusion [1]. This early alert could then be translated to in-hospital parameters at patient arrival. Base excess (BE) and ultrasound (FAST) are known to be predictive parameters for emergent transfusion. We emphasize that adding this two parameters to the TICCS could improve its predictability.

**Methods:** A retrospective study was conducted in the University Hospital of Liège. Based on the available data in the register (from January 1st 2015 to December 31st 2016), the TICCS was calculated for every patient. BE and FAST results were recorded and points were added to the TICCS according to the TICCS.BE definition (+3 points if BE < -5 and + 3 points in case of a positive FAST). Emergent transfusion was defined as the use of at least one blood product in the resuscitation room. The capacity of the TICCS, the TICCS.BE and the Trauma Associated Severe Hemorrhage (TASH) to predict emergent transfusion were assessed.

**Results:** A total of 328 patients were included in the analysis. 46 (14 %) needed emergent transfusion. The probability for emergent transfusion grows with the TICCS.BE value (Fig. 1). Positive Predictive Values (PPV) and Negative Predictive Values (NPV) of the three scores are displayed in Table 1.

**Conclusions:** Our results confirm that BE and FAST results are relevant parameters that can be added to the TICCS for better prediction of the need for emergent transfusion after trauma.

**References**

1. Tonglet ML et al. Crit Care 18(6):648, 2014

**Table 1 (abstract P367).** PPV and NPV of the three scores

	PPV	NPV
TICCS >= 10	66.6 %	88.0%
TICCS.BE >= 14	81.25%	89.1%
TASH >= 16	90.0%	87.1%

**Fig. 1 (abstract P367).**
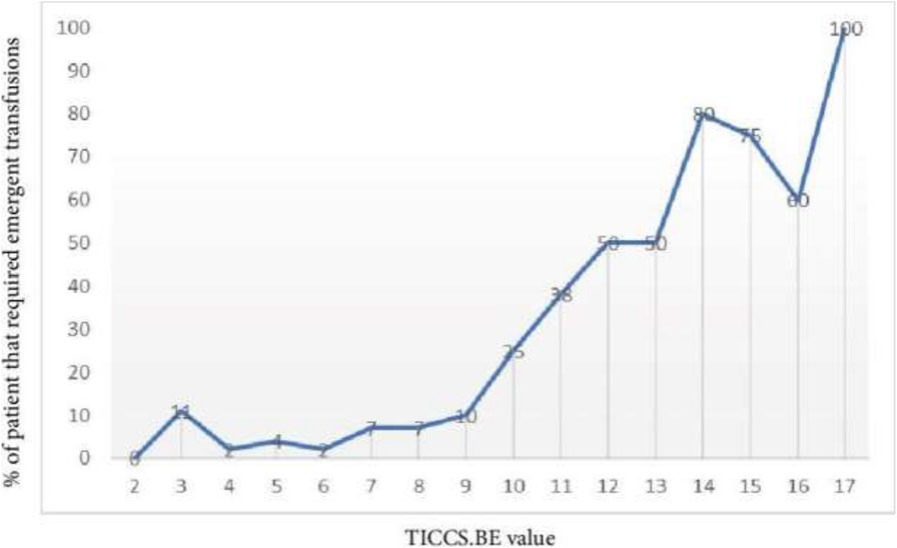
Probability for emergent transfusion with TICCS.BE values.

## P368 Influence of antioxidant therapy with high dose of vitamin c on mortality rates in critically ill polytrauma patients

### A Rogobete^1^, O Bedreag^1^, C Cradigati^2^, M Sarandan^2^, S Popovici^1^, D Sandesc^1^

#### ^1^Victor Babes University of Medicine and Pharmacy, Timisoara, Romania,^2^Emergency County Hospital "Pius Brinzeu" Timisoara, Timisoara, Romania

**Introduction:** The management of the critically ill polytrauma patient is complex and is often a challenge for the intensive care team. The objectives of this study is to analyze the oxidative stress expression in polytrauma cases as well as to evaluate the impact of antioxidant therapy on outcomes.

**Methods:** This prospective study was carried out in the Clinic for Anaesthesia and Intensive Care “Casa Austria”, form the “Pius Brînzeu” Emergency County Hospital, Timisoara, Romania, with the approval of the hospital’s Ethics Committee. ClinicalTrials.gov identifier NCT03095430. The patients’ selection criteria included an Injury Severity Score (ISS) of 16 or higher, and age of 18 or higher. 67 patients were eligible for the study. They were divided in two groups, group A (antioxidant free, control, N=32), and group B (antioxidant therapy, study group, N=35). The antioxidant therapy consisted in continuous IV administration of 7500 mg/24 h of vitamin C until discharge from ICU.

**Results:** The patients included in the study presented with similar characteristics, and no statistically significant differences were shown between group A and B regarding age (p > 0.05), sex (p > 0.05), ISS upon admission (p > 0.05), percentage of patients admitted in the ICU more than 24 hour post-trauma (p > 0.05), and associated trauma (p > 0.05). Among patients in group B statistically significant differences were identified regarding the incidence of sepsis (p < 0.05), multiple organ dysfunction syndrome (p < 0.05), mechanical ventilation time (p < 0.05), and mortality (p < 0.05). No statistically significant differences were shown regarding the time spent in the ICU (p > 0.05).

**Conclusions:** Following this study we can state that the administration of substances with a strong antioxidant character has positive influences on the outcome of critically ill patients, decreasing the incidence of secondary pathologies as well as mortality rates.

## P369 Diagnostics of coagulation disorders during different stages of traumatic disease

### I Basekno, O Tarabrin, D Sazhyn, Y Potapchuk, P Tarabrin

#### Odessa National Medical University, Odessa, Ukraine

**Introduction:** Polytrauma fully meets the criteria of the global pandemic. Throughout the world, as a result of traumatic injuries, about 16 thousand people die every day, and about 5.8 million people per year [1].

**Methods:** This study included 118 patients with traumatic injuries: concomitant skeletal trauma, fractures of pelvis, femur, humerus. On the 1st and the 2nd day all patients received tranexamic acid (15 mg/kg during 10 minutes followed by an infusion 1g during 8 hours), prothrombin complex concentrate (PCC) 1 ml/kg (25 IU/kg), and FFP in a dose 15 ml/kg. From the 3d till 14th day all patients received enoxaparin (3500 U every 12 hours). Method of low-frequency piezoelectric thromboelastography (LPTEG) used to study the functional state of the hemostasis system.

**Results:** Different periods of traumatic illness are characterized by various manifestations of blood coagulation disorders. The following LPTEG values were checked -Intensity of contact coagulation (ICC), Intensity of coagulation drive (ICD), clot maximum density (MA) and fibrinolytic activity - Index of retraction and clot lysis (IRCL). In the first period of traumatic injury ICC was decreased by 19.27%, ICD- decreased at 26.71%, MA was decreased by 24.92%, IRCL was 31.18% above the norm. The 3-14th days of post traumatic disease characterized next indicators – on the 5th day ICC were increased by 14.72%, compared to the norm; ICC increased by 22.62%, ICD increased by 17.57%, slightly increased MA, and IRCL was nearly in the normal range.

**Conclusions:** Rapid and accurate diagnosis of the coagulation system by LPTEG method at different stages of traumatic disease allows for more accurate selection and adjustment of the therapy, which allows improving the prognosis of the disease.

**References**

1. Pape HC et al. Damage control management in the polytrauma patient 2010, P.13-14.

## P370 Tranexamic acid in pediatric trauma

### JM Thomson, HM Drone, JL Jantzer, AK Tsai, JT Jancik

#### Hennepin County Medical Center, Minneapolis, Minnesota, USA

**Introduction:** Evidence for tranexamic acid (TXA) in the pharmacologic management of trauma is largely derived from data in adults [1]. Guidance on the use of TXA in pediatric patients comes from studies evaluating its use in cardiac and orthopedic surgery. There is minimal data describing TXA safety and efficacy in pediatric trauma. The purpose of this study is to describe the use of TXA in the management of pediatric trauma and evaluate efficacy and safety endpoints.

**Methods:** This retrospective, observational analysis of pediatric trauma admissions at Hennepin County Medical Center from August 2011 to November 2017 compares patients who did and did not receive TXA. The primary endpoint is survival to hospital discharge. Secondary endpoints include surgical intervention, transfusion requirements, length of stay, thrombosis, and TXA dose administered.

**Results:** There were 35 patients [<=] 16 years old identified for inclusion using a massive transfusion protocol order. Twenty patients (57%) received TXA. Baseline characteristics and results are presented as median (IQR) unless otherwise specified, with statistical significance defined as p < 0.05. Patients receiving TXA were more likely to be older, but there was no difference in injury type or injury severity score (ISS) at baseline (Table 1). There was no difference in survival to discharge, need for surgical intervention, or thrombosis (Table 2). Patients who did not receive TXA had numerically higher transfusion requirements and longer length of stay, but these did not reach significance.

**Conclusions:** TXA was utilized in 57% of pediatric trauma admissions at a single level I trauma center, more commonly in older patients. Though limited by observational design, we found patients receiving TXA had no difference in mortality or thrombosis.

**References**

1. CRASH-2 trial collaborators. Lancet 376:23-32, 2010.

**Table 1 (abstract P370).** Baseline characteristics

	Received TXA (N = 20)	No TXA (N = 15)	P-value
Age (years)	15 (10-16)	5 (4-13)	0.001
Gender, N (%) male	16 (80)	8 (53)	0.09
ISS	29 (24-46)	32 (21-43)	0.9
Injury type, N (%) blunt	15 (75)	12 (80)	0.73

**Table 2 (abstract P370).** Results

	Received TXA (N = 20)	No TXA (N = 15)	P-value
Survival to discharge, N (%)	13 (65)	11 (73)	0.6
Packed red blood cells (mL/kg)	18.7 (12.6-28.2)	27.2 (17.4-41.7)	0.4
Total blood products (mL/kg)	25.0 (15.9-50.1)	39.9 (23.2-68.1)	0.34
Surgical intervention, N (%)	15 (75)	13 (87)	0.39
Thrombosis, N (%)	3 (15)	3 (20)	0.7
Length of stay (days)	9 (2-17)	14 (4-32)	0.28

## P371 Withdrawn

## P372 Retrospective 5-years evaluation of vena cava inferior filter placement in patients with pelvic fractures at level 1 trauma center

### E Semenas, J Almström

#### Department of Surgical Sciences/Anaesthesiology and Intensive Care Medicine, Uppsala, Sweden

**Introduction:** The risk of venous thromboembolism (VTE) in trauma is greatly increased and one of the leading causes of morbidity and mortality after an accident [1]. Prophylactic measures to prevent VTE primarily consist of anticoagulants. In instances in which anticoagulation is contraindicated or inadequate, inferior vena cava (IVC) filters can be used [2]. However, insertion of IVC filter as a prophylactic measure is controversial as filter-related complications are well documented and increase with treatment time [3]. The objectives of our study were to evaluate IVC filter insertion indications and filter related complications in pelvic trauma patients.

**Methods:** 250 patients with pelvic fractures were operated during the study period 1/01/2011-31/12/2015. All patients who received IVC filter during the period were included into analysis. Relevant data was collected from electronic patient journal.

**Results:** Thirty four patients received retrievable filters during the study period (22 males and 12 females) (Table 1). Median age of patients was 59 years (range, 21-80). The predominant indication (79%) was prophylactic insertion. The median indwell time was 26 days (range 1-410 days). Despite IVC filter insertion one patient experienced lung embolism and another - DVT. In eleven cases IVC filters were tried to be removed at the treating hospital. In two cases filter extraction was unsuccessful and in another two cases filters were left in place due to IVC thrombosis.

**Conclusions:** Majority of IVC filters were inserted outside guidelines [4] and proportion of prophylactic indications is significantly higher (80% vs 24%) than seen in registry studies [5]. Filter related complications were observed in 18% of patients. More restrictive approach to prophylactic IVC insertion should be exercised.

**References**

1 Geerts et al. 1994

2. Falck-Ytter et al. 2012

3. Décousus et al. 1998

4. Falck-Ytter et al. 2012

5. Lee et al. 2015

**Table 1 (abstract P372).** Patient data

Age, median (min-max) years	59 (21-80)
Male/Female	22/12
Type of injury	
Isolated pelvic injury	13 (38%)
Head injury	10 (29%)
Thoracic injury	14 (42%)
Extremities injury	15 (44%)
Polytrauma	21 (62%)

**Table 2 (abstract P372).** Filter related complications

	Total number of patients (n=11)
Deep venous thrombosis	1
Lung embolism	1
Inferior v.cava thrombosis	2
Failed extraction of filter	2

## P373 The impact of preinjury antiplatelet and anticoagulant pharmacotherapy on outcomes in patients with major trauma admitted to intensive care unit (ICU)

### M Cozzolino, S Jaeger, S Batacchi, A Franci, F Socci, G Fulceri, M Bonizzoli, A Peris

#### A.O.U. Careggi, Florence, Italy

**Introduction:** Trauma is a leading cause of morbidity and mortality. The presence of comorbidity further complicates the management of trauma patients. Increasingly trauma patients are identified with preinjury use of antitrombotic agents. The purpose of the study was to determine whether preinjury antiplatelet and anticoagulant use affected outcomes after major trauma.

**Methods:** We conducted a retrospective review of patients >= 18 years admitted to our ICU with diagnosis of major trauma, defined as Injury Severity Score (ISS) >= 15. Patient data including age, Charlson Comorbidity Index (CCI), ISS, SAPS II and the number of patients with acute bleeding (transfusion with >= 3 red blood cells units) were collected. Patients were divided into 3 groups: patient without antitrombotic therapy (Group A), patient on antiplatelet agents (Group B) and patients on anticoagulation agents (Group C).

**Results:** From January 1, 2013 and July 1, 2017, 480 patients were identified. 386 patients (80.42%) in group A, 71 patients (14.79%) in group B and 22 patients (4.58%) in group C. Age, CCI, ISS and SAPS II are shown in Table 1. No differences between the groups were found for length of ICU stay and duration of mechanical ventilation. Incidence of acute bleeding was different between the groups: 26.16% in Group A, 40.84% in Group B and 31.81% in Group C (p=0.03). ICU and hospital mortality were significantly higher (p 0.00003) in Group B (35.21%; 40.85%) and Group C (27.27%; 31.82%) compared to Group A (13.73%; 15.81%).

**Conclusions:** Patients on preinjury anticoagulants and antiplatelet agents showed an increased mortality; this may be the result of the greater incidence of bleeding, the older age and more comorbidities in this groups.

**Table 1 (abstract P373).** Demographic data and scoring

	Group A	Group B	Group C	p
Age (years)	48.11 ± 19.48	77.57 ± 8.01	74.41 ± 12.32	p< 0.00001
CCI	1.3 ± 1.7	4.43 ± 1.75	4.23 ± 1.92	p<0.00001
ISS	31.26 ± 13.79	31.08 ± 11.26	33.63 ± 14.96	ns
SAPS II	37.49 ± 17.17	54.05 ± 17,74	48.90 ± 17.63	p<0.00001

## P374 Is enzymatic debridement better in critically burned patients?

### N Cáceres Giménez, C Gutierrez Mavarez, K Nanwani Nanwani, J Cantero Escribano, B Civantos Martin, J Martínez Méndez, A Garcia de Lorenzo

#### Hospital Universitario La Paz, Madrid, Spain

**Introduction:** Early debridement of burned tissue reduces infection rate, ICU stay and mortality. The use of proteolytic enzymes such as bromelain allows a faster, more effective and selective debridement of denatured tissue, preserving and exposing healthy tissues, reducing debridement times compared to standard of care.

**Methods:** Retrospective observational study performed in the Critical Burn Unit (March 2016 to September 2017) including 27 patients >18 years old with a total body surface area (TBSA) burned > 15% and < 75%, or > 65 years old with a TBSA burned > 10%, who underwent enzymatic debridement. Mean and standard deviation were used for normal quantitative variables and median and interquartile range in the opposite case. Qualitative variables were presented by absolute and relative frequencies.

**Results:** Mean age was 47.6 ± 17.8 years old, 74% males, APACHE II 11 (RI 5-18), ABSI 7 (RI 5-9). Median TBSA burned was 29% (RI 18-50%), 21% (RI 16-39) were deep dermal or full thickness. Time until debridement was 21 hours (RI 8-35). 7.4% (n=2) had incomplete debridement after first application, 33% (n=9) received regional anesthesia, 91% (n=25) didn’t need blood transfusion. 25% of patients who didn’t have vasopressors prior debridement, needed the use of it with a mean dose of 0,6mcg/Kg/min. 25% of patients with vasopressors prior treatment, required an increase of dose by a mean of 0.9 mcg/Kg/min. Median ICU stay was 19 days. Mortality was 22%.

**Conclusions:** Topical bromelain allows a fast start of tissue debridement with a low rate of failure. The need for fasciotomy and blood transfusion was very low. Topical treatment involved a fast and simultaneous debridement of the TBSA burned, generating an inflammatory response that in some cases required vasopressors.

## P375 Activity of the serum cholinesterase correlates with the length of the ICU stay following polytrauma

### AR Zivkovic^1^, K Schmidt^1^, T Stein^2^, M Münzberg^2^, T Brenner^1^, MA Weigand^1^, S Kleinschmidt^2^, S Hofer^3^

#### ^1^Heidelberg University Hospital, Heidelberg, Germany,^2^BG Trauma Center Ludwigshafen/Rhine, Ludwigshafen, Germany,^3^Westpfalz Hospital, Kaiserslautern, Germany

**Introduction:** Polytrauma with resulting systemic inflammatory response is associated with prolonged stay at the intensive care unit and delayed patient recovery. Early detection of the systemic inflammation might prove helpful in the treatment of the polytrauma patients. Cholinergic activity plays an important role in the inflammatory response. The activity of serum cholinesterase (butyrylcholinesterase; BChE) has been shown to correlate with extent of the acute inflammatory response. Here, we describe a correlation between the change in the BChE activity and the early systemic inflammation upon severe traumatic injury. Moreover, we assessed whether the BChE activity, when measured in patients at the hospital admission following polytrauma, could predict the patient outcome.

**Methods:** All patients (n = 47) or their legal designees gave written informed consent to the work (Trial-Code No. S-391/2015 and No. 837.539.15/10307). The BChE activity was measured by using point-of-care-test system (Securetec Detektions-Systeme AG, Neubiberg, Germany). Levels of the routine inflammation biomarkers, i.e. C-reactive protein (CRP) and the white blood cell count (WBCC), were measured during the initial treatment period. Measurements were performed at the admission, followed by 12, 24 and 48-hour time points. Injury Severity Score (ISS) was used to assess the trauma severity.

**Results:** The observed reduction in the BChE activity was in accordance with the change in the CRP concentration and the WBCC. The BChE activity measured at the hospital admission negatively correlated with the length of the ICU stay in patients with polytrauma (r = -0.5, Spearman’s rank correlation coefficient).

**Conclusions:** The BChE activity might be used as an early indicator for the magnitude of the systemic inflammation following polytrauma. Moreover, the BChE activity, measured at the hospital admission, might predict the patient outcome and therefore prove useful in early identification of the high-risk patients.

## P376 Pharmacological interventions for agitation in traumatic brain injury: a systematic review

### D Williamson ^1^, AJ Frenette^1^, M Perreault^2^, L Burry^3^, E Charbonney ^1^, F Lamontagne^4^, MJ Potvin^1^, JF Giguère^1^, S Mehta^3^, F Bernard^1^

#### ^1^Hôpital du Sacré-Coeur de Montréal, Montreal, Canada,^2^Université de Montréal, Montréal, Canada,^3^Mount Sinai Hospital, Toronto, Canada,^4^Centre Hospitalier Universitaire de Sherbrooke, Sherbrooke, Canada

**Introduction:** Among TBI complications, agitation is a frequent behavioural problem [1]. Agitation causes potential harm to patients and caregivers, interferes with treatments, leads to unnecessary chemical and physical restraints, increases hospital length of stay, delays rehabilitation, and impedes functional independence. Pharmacological treatments are often considered for agitation management following TBI. However, the benefit and safety of these agents in TBI patients as well as their differential effects and interactions are uncertain.

**Methods:** The major databases and the grey literature were searched. We included all randomized controlled, quasi-experimental, and observational studies with control groups. The population of interest was all patients, including children and adults, who have suffered a TBI. Studies in which agitation was the presenting symptom or one of the presenting symptoms, studies where agitation was not the presenting symptom but was measured as an outcome variable and studies assessing the safety of these pharmacological interventions in TBI patients were included.

**Results:** We identified 14 226 references with our search strategy. Two authors screened 12 899 after removal of duplicates. After searching the grey literature and secondary databases, a total of 170 potential articles were identified. Eleven studies in which agitation or an associated behavior was the presenting symptom, 11 studies where agitation was not the presenting symptom but was measured as an outcome variable, and 3 studies assessing the safety of these pharmacological interventions were identified. Overall, the quality of studies was weak. In studies directly addressing agitation, pindolol and propranolol may reduce assaults and agitation episodes. Amantadine and olanzapine may reduce aggression, whereas valproic acid may reduce agitated behavior.

**Conclusions:** There is weak evidence to support the use of pharmacological agents for the management of agitation in TBI.

**References**

1. Williamson DR et al. Syst Rev 5(1):193, 2016

## P377 Impact of decompressive craniectomy on neurological functional outcome in critically ill adult patients with severe traumatic brain injury: a systematic review and meta-analysis

### P Bonaventure, JA Jamous, F Lauzier, R Zarychanski, C Francoeur, A Turgeon

#### CHU de Québec – Université Laval, Québec, Canada

**Introduction:** Severe traumatic brain injury is associated with high mortality and functional disability. Several interventions are commonly used to control the intracranial pressure to prevent secondary cerebral injuries. Among them, decompressive craniectomy (DC) is widely performed; however, its impact on functional outcome is still under debate. Our objective was to assess the efficacy and safety of this procedure in adult patients with severe traumatic brain injury.

**Methods:** We systematically searched in MEDLINE, EMBASE, CENTRAL, Web of Science, conference proceedings and databases of ongoing trials for eligible trials. We included randomized controlled trials of adult patients with severe traumatic brain injury, comparing DC to any other intervention. Our primary outcome was the neurological function based on the Glasgow Outcome Scale. Secondary outcomes were mortality, intensive care unit (ICU) and hospital length of stay, intracranial pressure control, and complications. Two reviewers independently screened trials for inclusion and extracted data using a standardized form. We used random effect models to conduct our analyses and the I2 index to assess heterogeneity.

**Results:** We identified 5360 citations, from which we included 3 trials for a total of 573 patients. We observed no impact on the Glasgow Outcome Scale with the use of decompressive craniectomy (RR=1.03, 95%CI [0.74 ; 1.44]). We observed no effect on mortality (RR=0.66, 95%CI [0.40 ; 1.10]) or ICU length of stay (DM=-2.12, 95%CI [-7.80 ; 3.57]). However, we observed a decrease in the mean intracranial pressure (MD=-3.73, 95%CI [-5.78 ; -1.68]) and an increased incidence of patients with complications (RR=1.95, 95%CI [1.32 ; 2.89]).

**Conclusions:** We did not observe an association between DC and neurological outcome in adult patients with severe traumatic brain injury. Current evidence does not support the use of DC as a standard of care intervention in adult patients with severe traumatic brain injury.

## P378 Factors influencing the decision to monitor intracranial pressure in traumatic brain injury. A large monocentric study

### F Fossi^1^, C Robba^2^, M Rota^3^, A Vargiolu^3^, D Lagravinese^4^, PC Volpi^1^, G Citerio^3^

#### ^1^Università degli Studi Milano Bicocca, Monza, Italy,^2^Addenbrooke’s Hospital, Cambridge, UK,^3^San Gerardo Hospital, Monza, Italy,^4^IRCCS Ospedale San Raffaele, Milano, Italy

**Introduction:** The blurry indications on Intracranial pressure (ICP) monitoring in patients with Traumatic Brain Injury (TBI) causes differences in the clinical practice among different centers [1, 2]. Aim of this study is to assess the main factors that guided the decision making of monitoring ICP in TBI patients in our institution.

**Methods:** This is a retrospective, observational study including adult TBI patients admitted to the Neuro Intensive Care Unit (NICU) at S. Gerardo Hospital, Monza, from 1997 to 2016 [3].

Univariate logistic regression analyses were performed to identify predictors associated with the decision for ICP monitoring.

**Results:** A total of 857 adult patients were included (Tables 1 and 2). The risk of poor outcome estimated by the IMPACT model was associated to the decision to monitor ICP (Fig. 1). ICP was more often monitored in patients with severe TBI, with one dilated pupil at admission and positive CT findings (in particular, high Marshall scores).

**Conclusions:** According to our results, the clinician follows a multifactorial reasoning: the main determinants for the decision to monitor ICP are GCS, pupils’ abnormalities and, above all, CT findings. Future studies will be needed to clarify specific indications for the clinicians in the identification of patients who would benefit from invasive monitoring.

**References**

1. Maas AIR, Menon DK, Adelson PD, et al. Lancet Neurol. November 2017. doi:10.1016/S1474-4422(17)30371-X.

2. Carney N, Totten AM. Guidelines for the Management of Severe Traumatic Brain Injury. 4th ed. Brain Trauma Foundation

3. Citerio G et al. Acta Neurochir (Wien). 142(7):769-776, 2000

**Table 1 (abstract P378).** Relationship between trauma severity characteristics and outcome according to ICP monitoring in an adults TBI population admitted to San Gerardo Hospital NICU during a 20 years’ time horizon (January 1997 - December 2016)

	ICP monitoring	No ICP monitoring	OR (95% CI)	p-value
GCS Mild	42 (10%)	91 (22%)	1 (Reference)	<0.0001
GCS Moderate	83 (19%)	90 (22%)	2.00 (1.25-3.20)	
GCS Severe	317 (72%)	234 (56%)	2.94 (1.96-4.39)	
IMPACT < 25%	59 (13%)	124 (30%)	1 (Reference)	<0.0001
IMPACT >25% and <75%	257 (58%)	191 (46%)	2.83 (1.97-4.06)	
IMPACT > 75%	81 (18%)	58 (14%)	2.94 (1.86-4.64)	
Unfavourable outcome	235 (53%)	159 (38%)	2.09 (1.57-2.78)	<0.0001

**Table 2 (abstract P378).** Relationship between tomographic and TBI characteristics according to ICP monitoring in an adults TBI population admitted to San Gerardo Hospital NICU during a 20 years’ time horizon (January 1997 - December 2016)

	ICP monitoring	No ICP monitoring	OR (95% CI)	p-value
Marshall CT classification: I	5 (1%)	52 (13%)	1 (Reference)	<0.0001
Marshall CT classification: II	114 (26%)	209 (50%)	5.67 (2.20-14.60)	
Marshall CT classification: III	37 (8%)	28 (7%)	13.74 (4.85-38.91)	
Marshall CT classification: IV	8 (2%)	11 (3%)	7.56 (2.08-27.56)	
Marshall CT classification: V	251 (57%)	84 (20%)	31.08 (12.01-80.38)	
Marshall CT classification: VI	27 (6%)	31 (7%)	9.06 (3.16-25.96)	
SAH: no	157 (36%)	195 (47%)	1 (Reference)	0.0008
SAH: yes	285 (64%)	220 (53%)	1.60 (1.22-2.11)	

**Fig. 1 (abstract P378).**
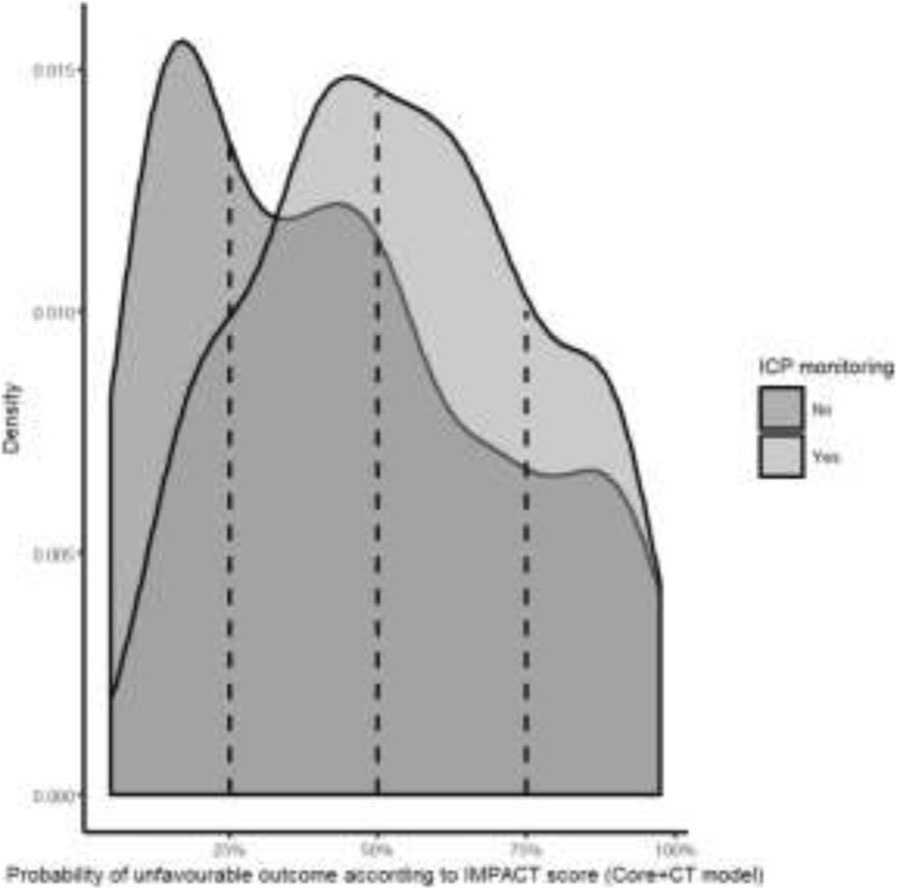
Histogram density plot of IMPACT score (Core + CT model) for predicting 6 months’ outcome according to ICP monitoring

## P379 Trajectories of early secondary insults after traumatic brain injury: a new approach to evaluate impact on outcome.

### PC Volpi^1^, C Robba^2^, M Rota^1^, A Vargiolu^1^, G Citerio^1^

#### ^1^Ospedale San Gerardo, Monza, Italy,^2^Addenbrooke’s Hospital, Cambridge, UK

**Introduction:** Secondary insults (SI) occur frequently after traumatic brain injury (TBI). Their presence is associated with a worse outcome. We examined the early trajectories of hypotension (SBP<90mmHg), hypoxia (SpO2<90%) and pupillary abnormalities from the prehospital settings to the Emergency Department (ED), and their relationship with 6-months outcome.

**Methods:** In this retrospective, observational study we included all TBI patients admitted to our Neuro Intensive Care Unit (NICU) from January 1997 to December 2016. We defined the trajectories of SI:

- "sustained" if present on the scene of accident and at hospital admission,
- "resolved" if present on the scene but resolved in ED,
- "new event" if absent on the scene and present in ED,
- "none" if no insults were recorded.

We investigated the association of SI trajectories with 6-months dichotomized outcome (Glasgow Outcome Scale (GOS); favorable=4-5; unfavorable=1-3).

**Results:** 967 patients were enrolled in the final analysis. Hypoxia and hypotension were related with unfavourable outcome when sustained (70.6% and 78.8%) and resolved (59.3% and 58.8%) while pupillary abnormalities were associated with unfavourable outcome when sustained and new events (65% and 66.7%). Results are summarized in the two figures below.

**Conclusions:** Trajectories could better define the dynamic and the burden of SI and their impact on outcome of TBI patients. Early treatments can influence evolution of SI and improve outcome.

**Fig. 1 (abstract P379).**
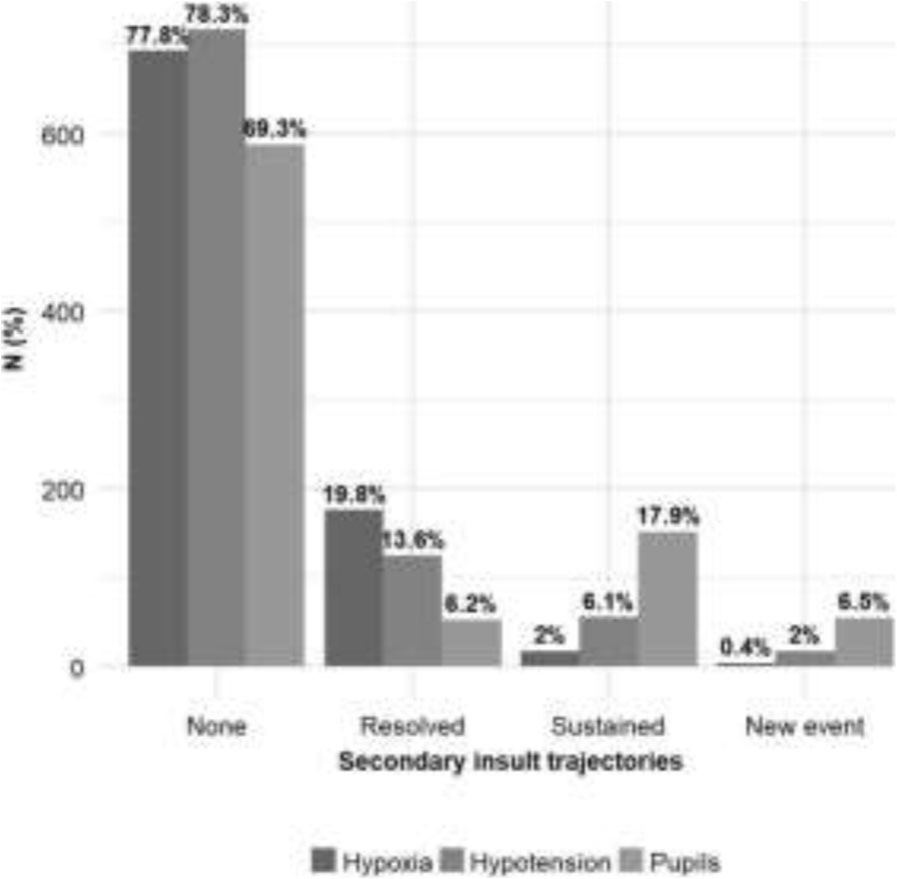
Trajectories of hypoxia, hypotension and pupillary abnormalities

**Fig. 2 (abstract P379).**
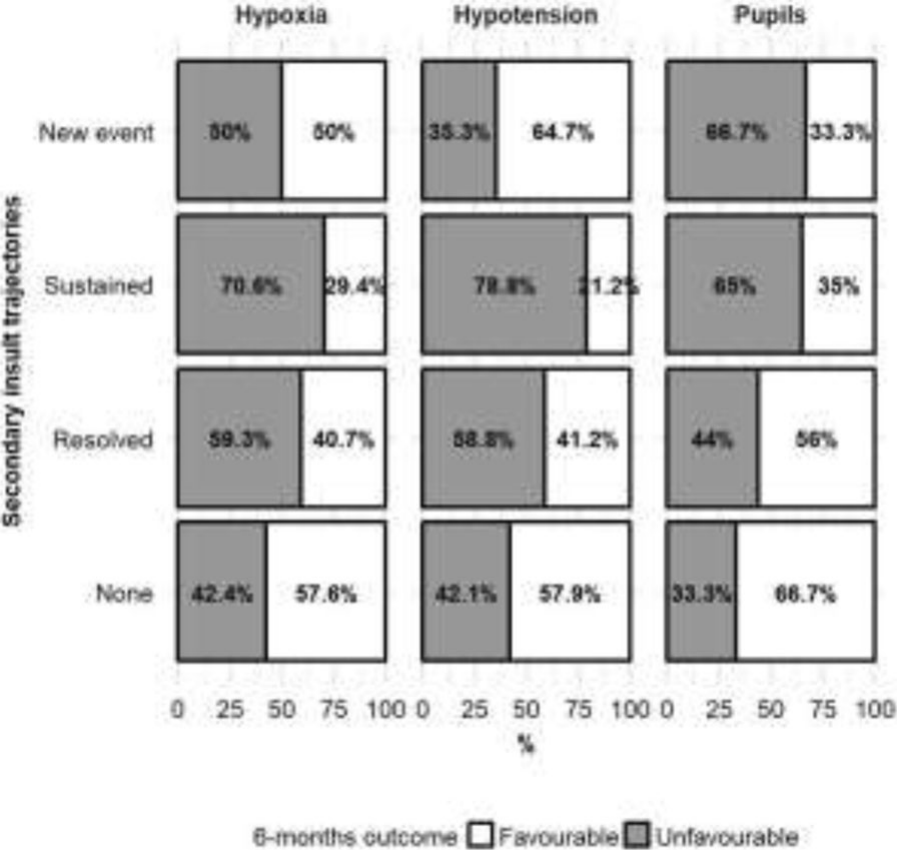
Trajectories of SI in relation to 6-months outcome as measured by GOS.

## P380 Experience with 23.4% sodium chloride for the management of intracranial hypertension in pediatric patients

### AR Birmingham, JL Jantzer, JT Jancik

#### Hennepin County Medical Center, Minneapolis, Minnesota, USA

**Introduction:** Guidelines for management of pediatric traumatic brain injury recommend maintaining intracranial pressure (ICP) <20 mmHg [1]. Use of 23.4% sodium chloride (NaCl) is considered safe and effective for management of ICP in adults, but evidence for concentrations >3% in pediatrics is limited. This study will describe the safety and efficacy of 23.4% NaCl in reducing ICP among pediatric patients.

**Methods:** This retrospective study evaluated patients <=18 years old who received 23.4% NaCl and had continuous ICP monitoring. Cerebral perfusion pressure (CPP), mean arterial pressure (MAP), ICP, and brain tissue oxygenation (PbtO2) were recorded hourly and were compared to baseline for 6 hours after each dose. Safety outcomes included peak serum sodium, peak serum chloride, and the incidence of stage 1 acute kidney injury (AKI) (serum creatinine elevation >=0.3 mg/dL or >=50%) [2].

**Results:** Between August 2007 and July 2017, 45 eligible pediatric patients received 235 doses of 23.4% NaCl; 215 doses were included in the analysis of perfusion parameters. Mean age was 11.6 +/- 6 years (2 months to 18 years), and the median initial Glasgow Coma Scale score was 4. Subjects received a median of four 23.4% NaCl boluses, with a mean dose of 0.5 +/- 0.18 mL/kg. Significantly lower ICP and higher CPP (p<0.001) were observed at all post-treatment time points (Fig. 1); PbtO2 was also significantly increased during 3 of the 6 hours recorded (p<0.05). There was no difference in MAP. Peak post-treatment serum sodium and chloride were 157 +/- 6 mEq/L and 122 +/- 7 mEq/L, respectively (Fig. 2). Stage 1 AKI was observed in 15.6% of patients, and in-hospital mortality was 24.4%.

**Conclusions:** Our data suggests that 23.4% NaCl is a safe and effective therapy for elevated ICP in pediatric patients.

**References**

1. Kochanek PM et al. Pediatric Crit Care Med 13:S1-S82, 2012

2. Sutherland SM et al. Clin J Am Soc Nephrol 10:554-561, 2015

**Fig. 1 (abstract P380).**
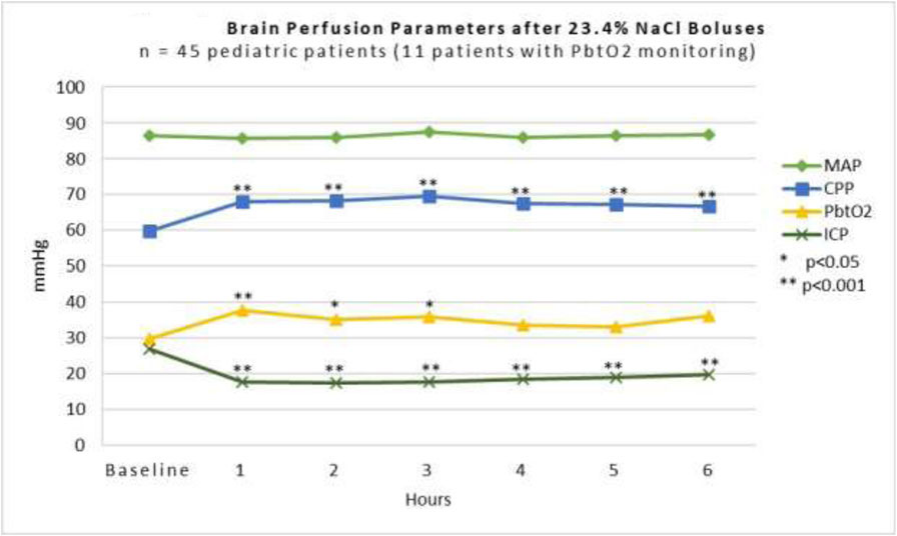
Brain Perfusion Parameters after 23.4% NaCl Boluses

**Fig. 2 (abstract P380).**
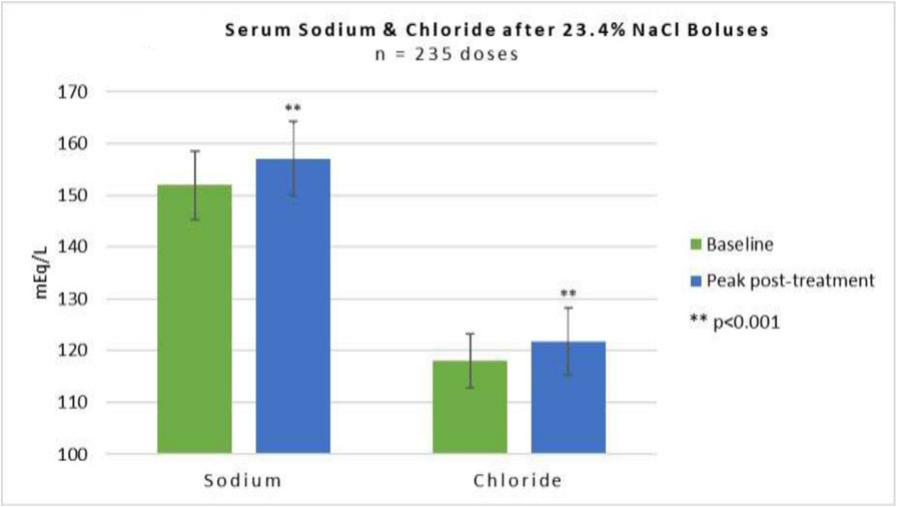
Serum Sodium & Chloride After 23.4% NaCl Boluses

## P381 The assistance of optic nerve sonography in evaluating patients with mild head trauma

### YT Lou^1^, C Chen^2^, Y Lin^2^

#### ^1^E-DA Hospital, I-Shou University, Kaohsiung, Taiwan,^2^Kaohsiung Medical University, Kaohsiung, Taiwan

**Introduction:** Focusing on patients with mild head trauma, we assessed the relationship between optic nerve sheath diameter (ONSD), clinical symptoms potentially related to intracranial lesions and confirmed intracranial hemorrhage (ICH) evidenced by head CT, and further investigated the effect of application of optic nerve sonography (ONS) on these patients’ clinical management and outcome.

**Methods:** We performed a prospective study in adult patients with mild head trauma (GCS 14 and 15) qualified for acquisition of urgent head CT scan. The clinical symptoms potentially related to intracranial lesion including abnormal vitals, vomiting, headache, persistent dizziness were recorded. ONS as well as head CT were then performed. All ONS examinations were executed by an experienced sonographer to eliminating interrater bias. Head CT findings were dichotomized as positive or negative finding for ICH based on formal radiology reports. The patients’ disposition including admission, surgery and safe discharge were followed.

**Results:** 78 patients were enrolled for the survey. 16 patients had at least one symptom related to potential intracranial lesion (20.5%). The mean ONSD was 44±9mm. 25 patients were found to have ICH and 6 underwent neurosurgery thereafter. No significant difference of ONSD was found between the groups with and without ICH, as well as the group receiving surgery or conservative treatment. With introducing a conventional 5mm threshold of ONSD, the sensitivity, specificity, PPV and NPV was 0.36, 0.83, 0.50 and 0.73, respectively. While incorporating occurrance of at least one positive clinical symptom with the ONSD measurement greater than 5mm as a composite threshold, the sensitivity, specificity, PPV and NPV was 0.32, 1.00, 1.00 and 0.76, respectively.

**Conclusions:** The diagnostic value of ONS in mild head trauma is defective. Nevertheless, with the supplemental aid of recognition of clinical symptoms relevant to potential intracranial lesion, the overall accuracy would improve.

## P382 A correlation between YKL-40 concentrations in cerebrospinal fluid and Marshall classification in traumatic brain injury – preliminary results

### G Pavlov^1^, M Kazakova^1^, P Timonov^1^, K Simitchiev^2^, C Stefanov^1^, V Sarafian^1^

#### ^1^Medical University - Plovdiv, Plovdiv, Bulgaria,^2^University of Plovdiv, Plovdiv, Bulgaria

**Introduction:** Establishment of prognostic models in traumatic brain injury (TBI) would improve the classification based on predictive risks and will better define treatment options [1]. In recent years, one of the most intensively studied glycoprotein is YKL-40. It is expressed as a consequence of broad spectrum of inflammatory and malignant diseases [2]. This is study aimed to investigate the level of YKL-40 in TBI patients and its relationship with several clinical models.

**Methods:** We determined plasma and cerebrospinal fluid (CSF) YKL-40 levels in six (6) patients – on the 24th and 96th hour after the TBI. Each patient was examined by physical and instrumental methods for somatic and neurological status, clinical assessment and prognostic scales (GCS, Marshall Classification, APACHE III). Routine haematological and biochemical tests were also performed. As control served the CSF of age-matched suddenly deceased healthy individuals (n = 11), which was examined post mortem for YKL-40 levels.

**Results:** We found no significant difference between plasma YKL-40 levels till 24th and 96th in all patients (mean difference ± SD: 57 ± 237 ng/ml). Significantly higher CSF YKL-40 levels till 24 h in TBIs compared to the control group was detected (p=0.014). A correlation between CSF YKL-40 concentrations till 24 h and Marshall Classification (p=0.042) was determined. No association of YKL-40 concentrations with GCS and APACHE III was observed neither for the plasma glycoprotein nor for the one in CSF.

**Conclusions:** Our preliminary results show that YKL-40 protein levels are related to the inflammation and to the prognostic model reflecting the pathophysiology of TBI. Acknowledgements: The financial support by the National Science Fund of Bulgaria (Contract DM 03/2 12.12.2016) is gratefully acknowledged.

**References**

1. Gomez PA et al. J Neurosurg 121:1314-1322, 2014

2. Johansen JS. Dan Med Bull 53:172-209, 2006

## P383 Cerebral autoregulation modifies electroencephalography power during intracranial hypertension events

### L Prisco^1^, A Vargiolu^2^, A Patruno^2^, G Citerio^3^

#### ^1^Oxford University Hospitals NHS Foundation Trust, Oxford, UK,^2^Ospedale San Gerardo, ASST, Monza, Italy,^3^University of Milan Bicocca, Milan, Italy

**Introduction:** Intracranial hypertension (IH) after acute traumatic brain injury (TBI) is a medical emergency which requires prompt treatment to prevent secondary brain injury. The mechanisms underlying IH are not yet fully understood. We explore the electroencephalography (EEG) signatures during IH.

**Methods:** In 5 TBI patients, we isolated 15 IH waves (Table 1). We analyse continuous simplified processed EEG power (Z-Ratio= {[(0-7Hz Power) – (7-30Hz Power)]/0-30Hz Power}) [1] time-synchronised to vital parameters (Intracranial Pressure, Mean Arterial Pressure and Cerebral Perfusion Pressure) (Fig. 1) and calculated Cerebral Autoregulation (AR) as correlation coefficients (Pearson) for each IH Wave. Z-Ratios were divided according to binary AR outcome and correlation calculated with intracranial pressure before, during and after the IH waves.

**Results:** Our preliminary analysis demonstrated a negative correlation between Intracranial pressure and Z-Ratio in the grouped 6 IH waves with preserved AR, but no correlation in the grouped 9 IH waves with impaired AR (Table 2 and Fig. 2). This indicates a decrease in power in the EEG low frequencies (0-7Hz) and/or an increase in the EEG high frequencies (7-30Hz) for increased values of intracranial pressure when AR is preserved.

**Conclusions:** Features of IH waves differ depending on the ability of the injured brain to autoregulate cerebral blood flow. These features might include different signature of EEG frequency changes. The causative links and clinical significance of the different EEG patterns remain unexplored and might represent a signature of neurovascular coupling.

**References**

1. Albertario CL et al. Sleep 18(10):836-43,1995

**Table 1 (abstract P383).** Patients Demographics

Subject Age (Years)	Marshall CT on admission	GOSE ICU, 6 & 12 months	N of IH events
70	5	2, 1, n/a	5
79	5	3, 1, n/a	2
36	5	2, 2, 2	3
61	5	4, 5, 5	0
59	2	3, 3, 4	5

**Table 2 (abstract P383).** Correlation (Pearson) between EEG Z-Ratio and ICP before, during and after episodes of intracranial hypertension. ICP epochs grouped based on preserved/impaired cerebral autoregulation

Autoregulation	Correlation coefficient R	P	95% CI
Preserved	-0.1967	0.001	-0.309 to -0.079
Impaired	0.0287	0.544	-0.064 to 0.121

**Fig. 1 (abstract P383).**
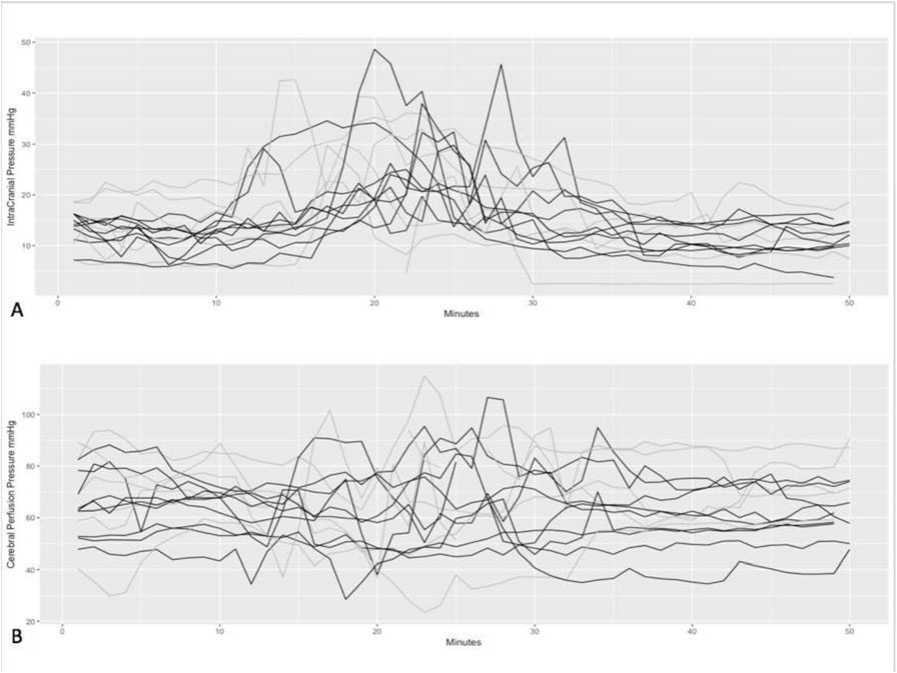
Plot of: A. Intracranial pressure during intracranial hypertensive events (Gray - Preserved Cerebral Autoregulation, Black - Impaired Cerebral Autoregulation). B. Cerebral Perfusion Pressure during intracranial hypertensive events (Gray - Preserved Cerebral Autoregulation, Black - Impaired Cerebral Autoregulation).

**Fig. 2 (abstract P383).**
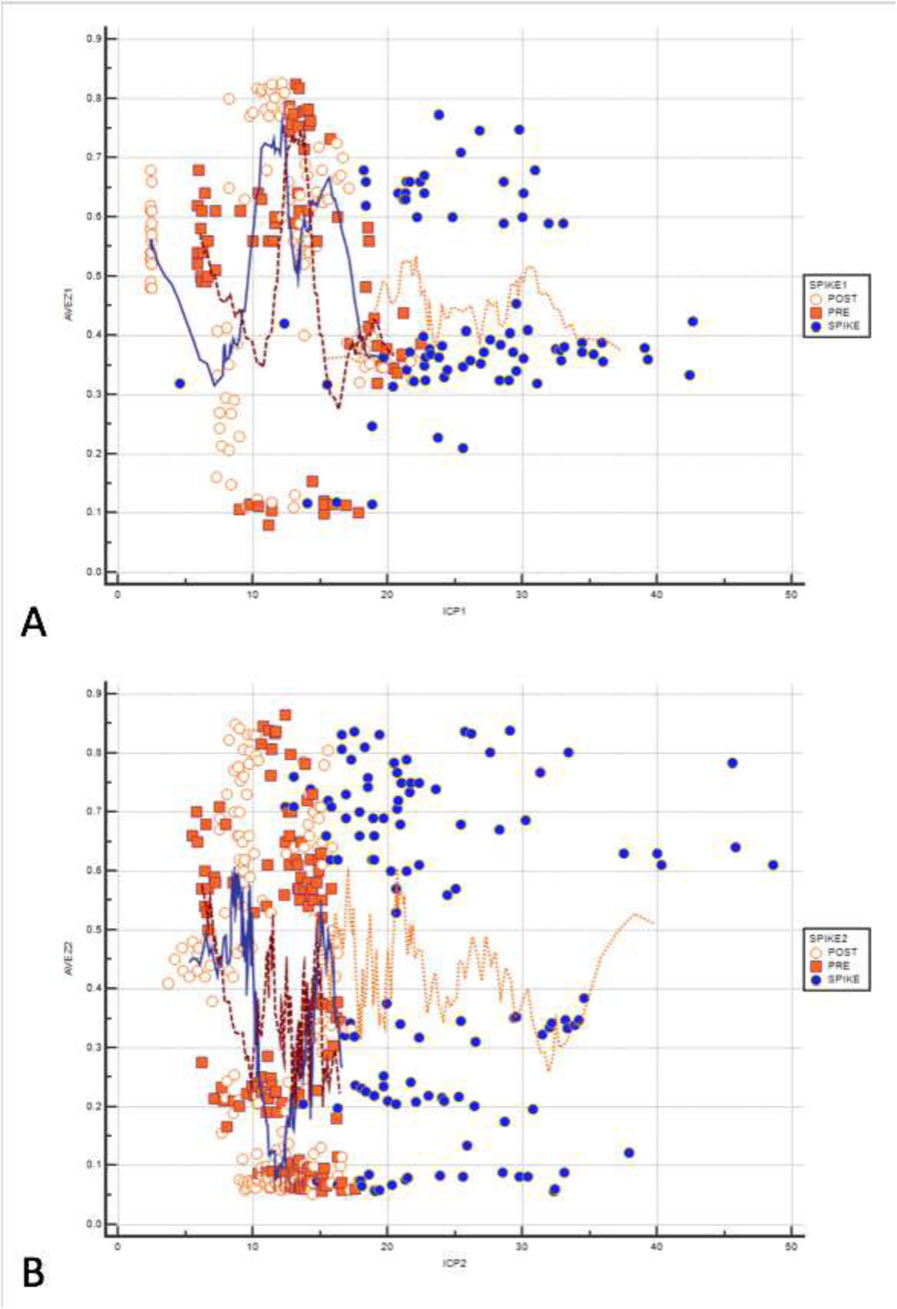
A. Correlation Scatter Plot and Moving Average (lines) between Average EEG Z-Ratio and Intracranial Pressure before (empty round orange marker), during (full orange square marker) and after (full blue round marker) intracranial hypertension events. A. Preserved cerebral Autoregulation. B. Impaired Cerebral Autoregulation.

## P384 Esophageal temperature management in patients suffering from traumatic brain injury

### F Bhatti ^1^, M Naiman^2^, AV Tsarev^3^, E Kulstad^4^

#### ^1^Bradford Teaching Hospitals NHS Foundation Trust, Bradford, UK,^2^University of Illinois, Chicago, Illinois, USA,^3^Dnipropetrovsk Medical Academy of the Health Ministry of the Ukraine, Dnipropetrovsk, Ukraine,^4^The University of Texas, Southwestern Medical Centre, Dallas, Texas, USA

**Introduction:** Targeted temperature management of patients who have suffered a traumatic brain injury is often used in the hope of preventing further insult to the brain; however, there is no uniform approach to managing temperature either locally, nationally or internationally, and maintenance of goal temperature in this patient population is often challenging due to hypothalamic injury. We sought to evaluate the feasibility and safety of an esophageal heat transfer device (EnsoETM, Attune Medical, Chicago, IL) to perform temperature management of patients suffering from traumatic brain injury.

**Methods:** This was an IRB-approved prospective study of patients undergoing temperature management after traumatic brain injury. Patients were treated with an esophageal heat transfer device connected to an external heater-cooler, and maintained at target temperature for at least 24 hours. Patient temperature obtained via Foley catheter was recorded hourly, and the deviation from goal temperature during treatment reported.

**Results:** A total of 12 patients were treated from August 2015 to May 2016. Temperature targets during treatment ranged from 34.0 to 36.8 degrees C. Maintenance of target temperature was successful, with 85% of readings within +/- 1 degrees C of target, and 75% of readings within +/- 0.5 degrees C of target. One patient developed a small hydrothorax, not attributed to device use. All patients survived to discharge from the ICU, with median CPC of 2 (range 1 to 4).

**Conclusions:** Targeted temperature management of patients with traumatic brain injury using an esophageal heat transfer device was feasible and safe, providing a tight maintenance of goal temperature in this challenging patient population.

## P385 Traumatic brain injury in Modena: 3 year experience of 6 months follow-up

### F Mosca^1^, S Rinaldi^2^, F Ragusa^3^, V Ferrari^4^, A Marudi^2^, E Bertellini^1^

#### ^1^Azienda ospedaliera universitaria Policlinico di Modena, Modena, Italy,^2^Ospedale Civile Sant’Agostino, Baggiovara (MO), Italy,^3^Ulss 9 Marca Ospedal Cà Foncello, Treviso, Italy,^4^Univeristà di Modena e Reggio emilia, Modena, Italy

**Introduction:** Traumatic brain injury (TBI) represents a serious problem in Europe. It still is the principal cause of death in US and Europe. Every year in Italy 250 people on 100,000 suffers of TBI and 17 on 100,000 dies. Disability and incapacity from TBI provides “strong ethicals, medicals, social and health economy imperative to motivate a concerted effort to improve treatment and preventions”

**Methods:** Our hospital is the hub for Modena’s county for TBI and we took part in the past 3 year on European project CREACTIVE (Collaborative ResearcE on ACute Traumatic brain Injury in intensiVe Care medicine in Europa) as branch of Italian group GIVITI (Gruppo Italiano per la Valutazione degli Interventi in Terapia Intensiva). Our study concerned about patients with TBI dismissed from ICU that “personally” or by the family will accepted the consensus to be included in our follow up conducted after 6 months from the dismissal. We collected clinical data from the admission to the dismissale and measured impact of TBI on all day life with GOS-E and QOLIBRI-OS using telephonical interview.

**Results:** We collected data about 63 patients, 33 answered to the telephonical follow-up and only 10 compilated the QOLIBRI-OS. We found out that patients admitted with lower GCS has worst outcome in terms of quality of life. It also appears that anisocoria during ICU staying represents an odds ratio for death and is connected with worst quality of life after 6 months from the dismissal (Tables 1 & 2). Inability to re-start a normal work-activity appeared to be the most important factor on the perception that our patient have of their new lives.

**Conclusions:** Anisocoria seems to be an indicator of severe brain damage. GCS, despite it’s simplicity, still represent the best and easiest way to score TBI. Work impairment appear to be the most important disability to determine subjective perception of quality of life after TBI, so efforts have to be made to improve work rehabilitation after the dismissal from hospital.

**Table 1 (abstract P385).** Correlation aniscoria - death

hospital outcome	no aniscoria	anisocoria	missing data anisocoria	n°
resigned	37	7	4	48
deceased	2	5	0	7
missing data outcome	6	0	2	8
n°	45	12	6	63

**Table 2 (abstract P385).** Correlation work impairment - QOLIBRI

QOLIBRI-OS	daily life activities impairment	no daily life activities impairment	work impairment	no work impairment
75	0	1	1	0
79.17	1	3	1	3
91.67	0	1	0	1
100	0	4	0	4
TOTAL	1	9	2	8

## P386 Moderate hyperventilation of short duration does not change cerebral metabolism in patients with severe traumatic brain injury

### G Brandi^1^, A Pagnamenta^2^, P Steiger^1^, S Klinzing^1^

#### ^1^University hospital of Zurich, Zurich, Switzerland,^2^Ente Ospedaliero Cantonale, Bellinzona, Switzerland

**Introduction:** Hyperventilation (HV) reduces elevated intracranial pressure (ICP) by changing autoregulatory functions connected to cerebrovascular CO2 reactivity. Criticism to HV is due to the possibility of developing cerebral ischemia and tissue hypoxia because of hypocapnia-induced vasoconstriction. We aimed to investigate the potential adverse effects of moderate HV of short duration in the acute phase in patients with severe traumatic brain injury (TBI), using concomitant monitoring of cerebral metabolism, continuous brain tissue oxygen tension (PbrO2), and cerebral hemodynamic with transcranial color-coded duplex sonography (TCCD).

**Methods:** A prospective trial was conducted between May 2014 and May 2017 at the University Hospital of Zurich. Adults (>18 years), with non-penetrating TBI, first GCS < 9, ICP-monitoring, PbrO2 and/or microdialysis (MD)-probes were included within 36 hours after injury. Data collection and TCCD measurements took place at baseline (A), at the begin of moderate HV (PaCO2 4-4.7 kPa) (C), after 50 minutes of moderate HV (PaCO2 4-4.7 kPa) (D), and after return to baseline (E) (Fig. 1). Repeated measures ANOVA was used to compare variables at the different time points followed by post hoc analysis with Bonferroni adjustment as appropriate. P-value < .05 was considered significant.

**Results:** Eleven patients were included (64% males, mean age 36 ± 14 years). First GCS was 7 (3-8: median and interquartile range). Data concerning PaCO2, ICP, PbrO2, mean flow velocity (MFV) in the middle cerebral artery, and MD values are presented in Table 1. During HV, ICP and MFV decreased significantly. PbrO2 presented a trend of reduction. Glucose, lactate and pyruvate did not change significantly (Table 2).

**Conclusions:** Short episodes of moderate HV have a potent effect on the cerebral blood flow, as assessed by TCCD, reduce ICP and PbrO2, and do not induce significant changes in cerebral metabolism.

**Table 1 (abstract P386).** Changes during the test. Data are expressed as mean with standard deviation

n= 11 A	C	D	E	Sign.
PaCO2 kPa 5.01 (0.23)	4.33 (0.24)	4.12 (0.35)	4.66 (0.37)	p<0.001
MAP mmHg 92 (10)	93 (12)	90 (10)	94 (13)	p=0.4
CPP mmHg 77(9.0)	85 (10)	81 (14)	80 (11)	p<0.05
ICP mmHg 16 (6)	8 (6)	10 (7)	14 (6)	p<0.001
PbrO2 mmHg 32 (10)	33 (10)	30 (8)	30 (8)	p<0.05
MFV right (cm/s) 81.0 (23)	68.6(21.2)	70.9 (20.5)	76.8 (28.2)	p<0.05
MFV left (cm/s) 80.1 (22)	65.6 (16.6)	63.4 (14.9)	75.3 (23.6)	p<0.001

PaCO2 AvsC p<0.0001 CvsD p<0.05 CvsE p<0005 DvsE p<0.0001 AvsE p<0.001 AvsD p<0.0001; CPP AvsC p<0.005 CvsE p<0.05; ICP AvsC p<0.0001 AvsD p<0.0001 CvsE p<0.0001 DvsE p<0.005; PbrO2 CvsD p<0.05; MFV right AvsC p<0.0001 AvsD p<0.005; MFV left AvsC p<0.05 AvsD p<0.001 CvsE p<0.05 DvsE p<0.05

**Table 2 (abstract P386).** Cerebral metabolism during the test. Data are expressed as mean ± standard deviation

	Baseline (1h –A)	D+1h	D +2h	
Brain glucose (mmol/L)	1.53 (0.99)n=8	1.42 (0.69)n=9	1.40 (0.67) n=9	p=0.48
Brain lactate (mmol/L)	3.34 (1.05)n=8	3.48 (1.29)n=9	3.45 (1.45) n=9	p=0.22
Brain pyruvate (umol/L)	101.44 (38.31)n=8	99.65 (39.49)n=9	97.26 (44.72) n=8	p=0.34
LP ratio	34.25 (8.21)n=8	39 (17.73)n=9	37.5 (11.44) n=8	p=0.5

PaCO2 arterial partial pressure of CO2, CPP cerebral perfusion pressure (mmHg), ICP intracranial pressure (mmHg), PbrO2 brain tissue oxygen tension (mmHg), MFV mean flow velocity in the middle cerebral artery

**Fig. 1 (abstract P386).**
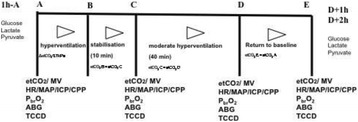
Study protocol. At different time points several variables were collected and TCCD measurements were performed. A baseline, B increasing minute ventilation, C begin moderate hyperventilation with target PaCO2 4-4.7 kPa, D after moderate hyperventilation for 50 minutes, E return to baseline. ABG arterial blood gas. Glucose, Lactate and Pyruvate concentrations in the extracellular fluid are sampled by microdialysis one hour before A (1h-A) and one (D+1h) and two hours after D (D+2).

## P387 Outcome of pediatric patients six months after moderate to severe TBI - results of CREACTKids study from three PICU in Israel

### I Lazar ^1^, O Duek^1^, V Knyazer^1^, Z Zonis^2^, Y Hoffmann^2^, J Ben-Ari^3^, A Hadash^3^, G Bertolini^4^, On Behalf of the CREACTIVE Consortium

#### ^1^Soroka Medical Center and the Faculty of Health Sciences, Ben Gurion University of the Negev, Beer Sheva, Israel,^2^Galilee Medical Center, Nahariya, Israel,^3^Ruth Rappaport Children’s Hospital, Rambam Health Campus, Haifa, Israel,^4^Mario Negri Institute for Pharmacological Research, Ranica, Italy

**Introduction:** CREACTIVE multinational project, follow patients with moderate-to-severe traumatic brain injury (TBI), as part of the global InTBIR initiative. Patients outcome are measured in terms of injury-related disability and quality of life at six months post injury. CREACTKids is the pediatric sub study. Here we present data collected from three pediatric intensive care units (PICU) from Israel.

**Methods:** Common Data Elements endorsed by InTBIR are collected, Clinical data is collected via computerized software. Children outcome are followed-up with separate Tool-box. The tool-box assesses impairment and disability in sleep, behavioral, emotional and neurocognitive domains using both questionnaires (GOSe-Peds, Peds-QL 4.0, Strengths & Difficulties, Temperament, UCLA-RI) and computer based neurocognitive tasks. Outcome is assessed during meeting 6 months post injury. We present two years descriptive results and TBI-patients full outcome assessment.

**Results:** 2428 patients were admitted to three PICUs during 2016-2017, 209(8.6%) due to TBI. 43 completed 6-month ambulatory follow-up. Follow-up shows most children GOSe-Peds score was upper moderate disability or higher, However, a marked cognitive impairment (mainly in memory and inhibition) was found: mean score of TBI patient’s Corsi task was 3.18±2 while normal controls 5.19±2.3. Patients correct answers in Go/No go task was 48.08±9.5 while normal control’s mean is 120.1±31.3. Results also reveals an increase in emotional and behavioral problems (compared to status before injury). More than 50% of patients show at least one significant symptoms of post traumatic stress disorder (PTSD).

**Conclusions:** Our results describe the heavy burden of TBI on Pediatric patients. Most patients showed reasonable physical recovery (as per GOSe-Peds) but with alarming Socio-Cognitive disability. We suggest better diagnosis and early intervention to improve these patients’ outcome.

## P388 Analysis of external ventricular drainage associated infection in the neurosurgery ICU of the single tertiary level hospital

### N Balciuniene, T Tamosuitis, U Dobrovolskyte

#### Lithuanian University of Health Sciences, Kaunas, Lithuania

**Introduction:** The external ventricular drainage (EVD) is one of the most frequent interventions in our Neurosurgery Intensive care Unit (NICU) and about 15% of these patients are diagnosed EVD related infection (meningitis or ventriculitis). The aim of our study was to evaluate the prevalence, risk factors and outcome of EVD associated infection in NICU patients.

**Methods:** We performed a single-center observational, retrospective cohort study of 212 who underwent EVD insertion operation during their stay in the NICU at our 2200 bed university teaching hospital Kaunas Clinics Hospital from January 2012 to December 2016. These patients (103 males) with median age 63 (19-80), were used for further analysis. There were 50 patients in EVD related infection group. Clinical variables such as age, sex, prior clinical diagnosis, duration of EVD, total numbers of EVD per person, and outcome were analyzed to verify the risk factors, causative agents and outcomes.

**Results:** An infection was documented in 23% of all patients. Extended duration of catheterization correlates with an increasing risk of neuroinfection during the first 10 days of catheterization (p=0.034). Also a significant risk factors were reinsertion of EVD (p=0.001), unfavorable outcome in Glasgow Outcome Scale (score< 9, p=0.029). Patients with infections had a longer intensive care unit stay (p = 0.0029) or hospital stay (p=0.004).

**Conclusions:** Longer duration of drainage, repeated insertion or unfavourable outcome in Glasgow Outcome Scale are the major risk factors for EVD associated infection.

## P389 Preliminary report : decreasing incidence of delirium in surgical intensive care unit by maintaining target cerebral oxygen using regional cerebral oxygen saturation (rso2) during intraoperative major surgery

### K Auksornchat^1^, K Kumwilaisak^2^

#### ^1^Thammasat University, Prathumthani, Thailand,^2^King Chulalongkorn Memorial University, Bangkok, Thailand

**Introduction:** Delirium is a major cause of complications in postoperative patient in ICU. Risk factors for delirium include poor cerebral hemodynamics and peri-operative cerebral desaturations. Intra-operative target cerebral oximetry monitoring may decrease the incidence of postoperative delirium in elective major abdominal surgery patients.

**Methods:** A single-blinded, randomised controlled trial in patients undergo elective major abdominal surgery who received postoperative care in surgical ICU with age more than 65 years were randomised into two groups. The intervention group was received intra-operative target cerebral oxygen monitoring using cerebral oximetry whereas the control group was not. Delirium was assessed in both group at 24, 48, 72 hour postoperatively. Other risk factors for delirium, mechanical ventilator day, length of ICU stay, length of hospital stay and post-operative complication were recorded.

**Results:** From August 2015-March 2016, 37 patients who met the criteria were randomised to 19 patients in intervention group and 18 patients in control group. Overall incidence of delirium was 27.03% (Intervention 21.05% VS Control 33.34%, p=0.401). Baseline cerebral oxygen in intervention group was 66.79 ± 3.11%. Desaturation below 10% from baseline was found in 8 from19 patients (42.1%) and was the only significant risk factor associated with delirium (p=.008, odd ratio 1.68). There was no significant different in mechanical ventilator day, ICU length of stay, hospital length of stay and postoperative complication between both groups. There was no complication associated with application of the cerebral oximetry probe in the intervention group.

**Conclusions:** From this preliminary report can not demonstrated the significant different of intra-operative target cerebral oxygen monitoring by using cerebral oximetry in prevention of delirium. However the reduction of cerebral oxygen more than 10% from baseline in intervention group showed significantly associated with delirium postoperatively.

## P390 The SET score as a predictor of ICU length of stay and the need for tracheotomy in stroke patients who need mechanical ventilation

### A Henein, N Suri, M Saad, E Attallah

#### Khoula Hospital, Muscat, Oman

**Introduction:** SET score was initially developed as an in- house screening tool based on tracheotomy predictors identified in several retrospective studies. It combined the categories of neurological function, neurological lesions, and general organ function/procedure, and weighed by allocation of certain point values [1]. In our study it was very interesting to us to find a tool to judge application of early tracheotomy, and as we have a good culprit number from stroke cases so we decided to try to apply this score in our ICU after discussion with the inventor of this score.

**Methods:** 164 stroke patients were prospectively included in the study as they were ventilated or were very little potential for ventilation and assessed by the stroke-related early tracheotomy score (SET score, Table 1) within the first 24 h of admission (Table 2). Endpoints were length of stay and ventilation time (VT) after doing early tracheotomy. We examined the correlation of these variables with the SET score using standard analytical methods.

**Results:** The SET score with a value cutoff point of 8 had a significant effect on decision of making tracheotomy and hence decreasing ventilation time and length of stay in ICU, which affected outcome (Figs. 1 & 2).

**Conclusions:** All efforts must be exhausted in neuro intensie care to decrease the secondary changes of brain injury after stroke,early tracheotomy is a good tool to decrease length of stay in ICU and ventilation time in these patients.Inventing a tool to judge these decisions of doing tracheotomy was a challenge. SET score proved to be valuable.Further multi center trials are needed.

**References**

1. Schӧnenberger S et al. Neurocrit Care 25:94-104, 2016

**Table 1 (abstract P390).** SET score

Areas of assessment	situation	points
neurological function	dysphagia	4
	observed aspiration	3
	GCSon admission <10	3
	brain stem	4
neurological lesion	space occupying cerebellar	3
	MCA infarction>2/3	4
	ICH>25 ml	4
	diffuse lesion	3
	hydrocephalus	4
general organ function/procedure	neurosurgical intervention	2
	additional respiratory disease	3
	Pao2/Fio2>150 mmhg	2
	APS of APACHEII>20	4
	LUNG IIJURY SCORE>1	2

**Table 2 (abstract P390).** Demographic data of the patients

MCA	Total 80	70 needed tracheotomy	10 did not do tracheotomy
spontaneous ICH	Total 30	16 needed tracheotomy	14 did not need tracheotomy
SAH	Total10	2 needed tracheotomy	8 did not need tracheotomy
Hydrocephalus	Total 6	4 needed tracheotomy	4 did not need tracheotomy
Brain stem hemorrhage	Total 8	8 needed tracheotomy	0 did not need tracheotomy
Diffuse lesion	Total 18	6 needed tracheotomy	12 did not need tracheotomy

**Fig. 1 (abstract P390).**
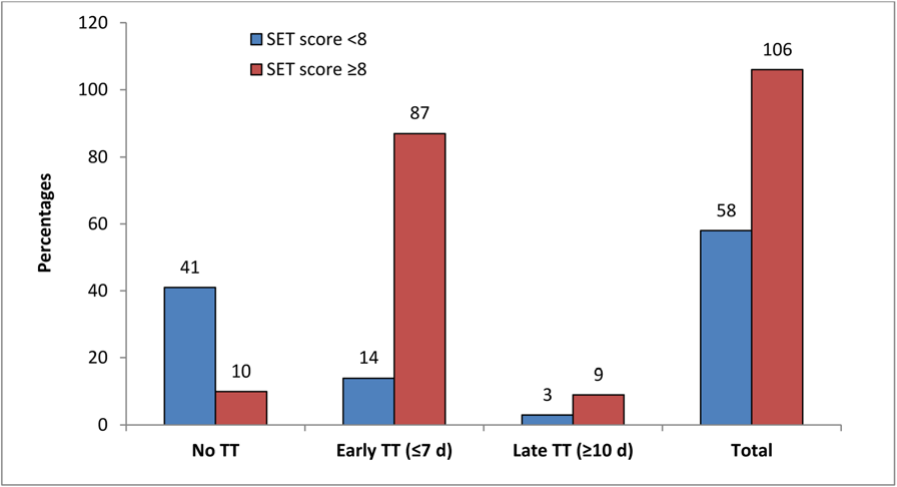
Distribution of tracheostomy by the SET score among the studied patients (N=164)

**Fig. 2 (abstract P390).**
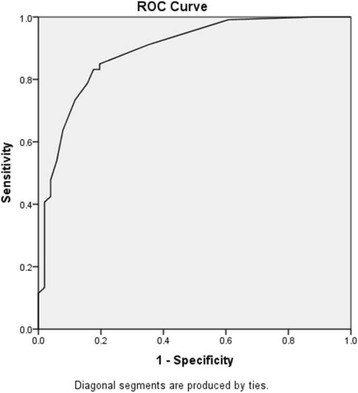
Specificity for the cutoff point of SET SCORE. Cut point of 9 is the best to predict tracheostomy with sensitivity of 85.0% and specificity of 80.4%. Cut point of 9 is the best to predict early tracheostomy with sensitivity of 86.1% and specificity of 81.5%. Since no patients had score 9 so the previous analysis that consider cut-point of 8 should remain the same but just change the number in the text to 9

## P391 Venous thromboembolism prophylaxis among neurocritical care patients: what is current practice?

### KM Sauro^1^, DJ Niven^1^, A Soo^1^, P Couillard^1^, A Kramer^1^, J Kromm^1^, D Zygun^2^, SM Bagshaw^2^, HT Stelfox^1^

#### ^1^University of Calgary, Calgary, Canada, ^2^University of Alberta, Edmonton, Canada

**Introduction:** The objective was to examine VTE prophylaxis practices among neurocritical care patients (NCC) and evaluate concordance with Neurocritical Care Society guidelines. Venous thromboembolism (VTE) is a leading cause of preventable, in-hospital deaths in high income countries, and patients admitted to ICUs are at increased risk. Effective and efficient strategies to prevent VTE exist; however, NCC patients present unique challenges due to competing risks of bleeding.

**Methods:** Population-based administrative data and electronic medical records were used to retrospectively audit VTE prophylaxis practices among NCC patients admitted to 10 adult medical-surgical/neurological ICUs in Alberta, Canada in 2014. NCC patients were identified using admission diagnosis. Data included: demographic characteristics, form of VTE prophylaxis, contraindication for pharmacological VTE prophylaxis (platelet count <50 x109/L, INR >=2, PTT >=55 sec, diagnosis with high-risk of bleeding, documented therapeutic anti-coagulation) and outcomes of care. Guideline concordance was evaluated.

**Results:** Of 7,669 admissions, 16.5% were NCC patients. Typically, NCC patients were 56 years-old, male, with no comorbidities (52.3%), and an APACHE II score of 17. Most NCC patients had a contraindication for pharmacological VTE prophylaxis (65.4%). Overall, NCC patients were more likely to receive mechanical (90.3% ICU days) than pharmacological VTE prophylaxis (74.1% ICU days), however pharmacologic was more likely among younger patients with lower APACHE II scores. Guideline concordant care varied by recommendation; lower for pharmacological and higher for mechanical VTE prophylaxis.

**Conclusions:** NCC patients uncommonly receive guideline concordant pharmacological VTE prophylaxis. Collectively, our findings suggest that current VTE prophylaxis prescribing practices may reflect uncertainty around risks associated with VTE prophylaxis among NCC patients.

## P392 Prophylaxis of postoperative venous thromboembolism for cerebral aneurysm correction

### V Cordeiro Veiga, LM Coscrato Junqueira, L Freitas, P Travassos, RT Vale, TK Alvarisa, J Caldas, S. Rojas

#### Hospital BP - A Beneficência Portuguesa de São Paulo, São Paulo, Brazil

**Introduction:** The neurocritical care patients are at particular risk to VTE, who immobile due to neurologic injury and these group of patient frequently have a prolonged hospital course since multiple neurologic and medical complications may arise. The objective is to evaluate the safety of the early introduction of prophylaxis of venous thromboembolism in the postoperative period of cerebral aneurysm repair.

**Methods:** Retrospective study. 157 patients submitted to surgical correction of the cerebral aneurysm (81) or endovascular (76). January 2014 through September 2015. Were evaluated: Prophylaxis type and complications and outcomes: venous thromboembolism and pulmonary thromboembolism during hospitalization and follow-up of 6 months and survival at 30 days and 6 months.

**Results:** 80.2% were females. Mean age of 54.5 ± 14.4 years. Mean time to hospital stay: 3.9 ± 5.5 days (Surgical group: 4.5 days versus 1.99 days in endovascular group, p < 0.001). 13.4% had subarachnoid hemorrhage at the time of admission, with length of stay of 5.87 days (p < 0.001). Prophylaxis during permanence UTI: 94.9% mechanical prophylaxis, 25.5% pharmacological prophylaxis and 24.4% combination therapy. Of the patients who received pharmacological prophylaxis, there were two cases of heparin-induced thrombocytopenia and one case of major bleeding, in both cases, pharmacological prophylaxis was discontinued. The prophylaxis of venous thromboembolism was started in 24 to 48 hours in 95.5% of the patients. Venous thromboembolism was found in 1.27% of patients and pulmonary thromboembolism in 1.91%, both in surgical patients Survival was 30 days in 98.7% and 6 months: 96.21% (no statistical difference with endovascular and surgery groups).

**Conclusions:** The introduction of prophylaxis of venous thromboembolism in postoperative patients of cerebral aneurysm is a safe practice and should be established routinely in this group of patients.

## P393 Early pharmacological thrombosis prophylaxis is not associated with hematoma expansion in patients with primary intracerebral hemorrhage

### B Ianosi^1^, M Gaasch^2^, W Hackl^1^, M Kofler^2^, A Schiefecker^2^, L Huber^1^, R Beer^2^, V Rass^2^, P Rhomberg^2^, B Pfausler^2^, C Thomé^2^, E Schmutzhard^2^, E Ammenwerth^1^, R Helbok^2^

#### ^1^UMIT - the health & life sciences university, Hall in Tirol, Austria,^2^Medical University of Innsbruck, Innsbruck, Austria

**Introduction:** European guideline recommendations for the start of pharmacological deep vein thrombosis (DVT) prophylaxis after intracerebral hemorrhage (ICH) are unprecise and result in different clinical approaches. We analysed the effect of early (<48h) and late (>48h) administration of subcutaneous enoxaparin as DVT prophylaxis on prolonged hematoma expansion (HE) (>24h) and outcome in patients with primary ICH.

**Methods:** We retrospectively analysed prospectively collected data from 134 consecutive ICH patients that received DVT prophylaxis in a tertiary hospital. HE was defined as an increase of >6mL measured using the ABC/2 method or the semiautomatic software based volumetric approach. Using multivariate analysis, we analysed risk factors including early DVT prophylaxis for HE>24h, hospital mortality and poor 3-month functional outcome (3m modified Rankin Score>3).

**Results:** Patients presented with a median GCS of 14 (IQR 10-15), hematoma volume of 11mL (IQR 5-24) and were 71y old (IQR 61-76). 56% received early DVT prophylaxis, 37% late DVT prophylaxis and 6% had unclear bleeding onset. Hematoma volume was smaller in the early DVT prophylaxis group with 9.5mL (IQR 4-18.5) vs 17.5mL (IQR 8-29) in the late prophylaxis group (p=0.038) without any other significant differences in disease severity. Delayed HE (N=5/134, 3.7%) was associated with higher initial hematoma volume (p=0.02) and lower thrombocyte count (p=0.03) but not with early DVT prophylaxis (p=0.36) in a multivariate analysis adjusted for known risk factors. Early DVT prophylaxis was not independently associated with 3m outcome.

**Conclusions:** Although limited by the retrospective design, our data suggest that early DVT prophylaxis (<48h) may be safe in patients presenting with primary ICH, which supports the recommendations given by the Neurocritical Care Society.

## P394 Lack of evidence of increased mortality in patients with intracerebral hemorrhage admitted during the weekend

### B Ianosi^1^, V Rass^2^, L Huber^1^, W Hackl^2^, M Kofler^2^, A Schiefecker^2^, M Gaasch^2^, R Beer^2^, P Rhomberg^2^, B Pfausler^2^, C Thomé^2^, E Schmutzhard^2^, E Ammenwerth^2^, R Helbok^2^

#### ^1^UMIT - the health & life sciences university, Hall in Tirol, Austria,^2^Medical University of Innsbruck, Innsbruck, Austria

**Introduction:** A recent study suggested that weekend admission is associated with higher risk of mortality in Austrian intensive care units (ICU) [1]. We investigated if this association holds true for emergency admissions of patients with intracerebral hemorrhage (ICH) in our neurological ICU (NICU).

**Methods:** Prospectively collected data from 256 consecutive ICH patients, admitted to our tertiary care hospital were retrospectively analyzed. Weekend admission was defined from Friday 5 PM to Monday 8 AM. We used COX Regression and multivariate analysis to identify risk factors including admission time for hospital mortality and 3-month outcome with poor outcome defined as modified Rankin Scale score (mRS) >3.

**Results:** 31% of patients (79/256) were admitted on the weekend. Patients presented with a median GCS of 13 (IQR 5-15), hematoma volume of 24 mL (IQR 9-45), SAPS Score of 29 (IQR 24-36) and were 72 years old (IQR 64-80). Median time from symptom onset until NICU admission was 3 hours (IQR 2-7). There was no difference between groups based on admission time concerning these factors and in addition, surgical treatment, decision to withdraw/withhold therapy, intubation, use of nasogastric tube or infectious complications were similar. Incidence of hydrocephalus requiring external ventricular drainage (p=0.027) was higher in the weekend group. Hospital- and 3m-mortality was 25% and 32% and did not differ based on admission time (p=0.35, p=0.7). In contrary, known contributors for poor outcome were significantly associated: increased age (p=0.037), increased ICH Volume (p=0.001) and lower GCS at admission (p<0.001) or infratentorial location of ICH (p=0.011).

**Conclusions:** Weekend admission did not result in a higher mortality rate in ICH patients admitted to our NICU with 24-7 neuro-intensivist staffing. Due to the different SAPS on admission (lower in our group) an age and severity matched comparison of neurological and non-neurological patients is needed

**References**

1. Zajic P et al. Crit Care. 21(1):223, 2017

## P395 Some characteristics of hormonal status in patients in the acute period after spontaneous intracerebral hemorrhage

### L Tsentsiper, N Dryagina, E Kondratyeva, A Kondratyev

#### Russian Polenov’s Neurosurgical Institute, Sankt-Petersburg, Russia

**Introduction:** The incidence of spontaneous intracranial hemorrhage (SICH) in the world according to different authors is from 1.5 to 2 million a year. SICH is associated with an increased risk of endocrine dysfunction.

**Methods:** This study included 58 patients aged 13 to 72 years. We studied the levels of ACTH and cortisol in the morning and the evening, TSH, FT3 and FT4, prolactin, GH, men determined LH, FSH, testosterone. GCS; rating on a scale of Hunt and Hess patients with arterial aneurysm rupture were consistent with points 2-4, 2-4 class Fischer scale. All patients were subjected to sedation and analgesia (further “sedation”) as therapeutic narcosis - opioid analgesic fentanyl 0.5-1 μg/kg/h, alpha 2-adrenergic agonist clonidine 0.2-0.5 μg/kg/h, sodium thiopental 2.4 μg/kg/h. for 3 to 9 days.

**Results:** The endocrine status of the patient was estimated depending on her functional state at the sedation and post sedation period. Our results indicate lower activity of the PAS, PTS for sedated patients. Our studies conducted within 28 days after a SICH revealed no hormonal deficiency requiring correction in the acute period. Therapeutic sedation limits the severity of the stress response indicating that patient in sedation period had ACTH and cortisol levels lower than in post- sedation period.

**Conclusions:** The hormone response to SICH is different in patients after aneurysmal SAH and ones of hypertensive genesis and caused by AVM rupture, the latter had more pronounced HP activation.

Patients were diagnosed with pituitary-adrenal system hyperfunction with daily secretion rhythm irregularities. Patients with negative outcome during post-sedation period had more evident pituitary-adrenal system hyperfunction than those with positive one. Patients with negative outcome had FT3 level reduction (34-45%).

Male patients developed hypogonadotropic hypogonadism.

## P396 Impact of clevidipine on intracranial pressure in adults with intracranial hemorrhage

### KA Elwood, TS Lam, HM Rhodes, JT Jancik

#### Hennepin County Medical Center, Minneapolis, Minnesota, USA

**Introduction:** There is a paucity of literature describing the relationship between clevidipine and its impact on intracranial pressure (ICP). The safety of clevidipine in patients with intracranial hemorrhage is often extrapolated from studies using nicardipine, which has demonstrated a neutral effect on ICP [1]. The objective of this study was to determine if there was a relationship between clevidipine initiation and changes to cerebral hemodynamic parameters.

**Methods:** This study was a retrospective analysis of adults admitted to Hennepin County Medical Center between July 2012 and July 2017. Individuals were included if they had intracranial bleeding and ICP data recorded prior to initiation of a clevidipine infusion. Baseline demographic data was collected, including age, gender, type of injury, and initial Glasgow Coma Score (GCS). Data was collected to evaluate ICP, cerebral perfusion pressure (CPP), systolic blood pressure (SBP), and clevidipine infusion parameters. Cerebral hemodynamic parameters and blood pressures were evaluated using a paired t-test. Results were considered statistically significant if P < 0.05.

**Results:** A total of 14 patients with 17 encounters qualified for inclusion. The median age was 48 years (range 18-77). Males comprised 78.6% of the population. The most common injury was intracranial hemorrhage, occurring in 69.2% of the population. The median initial GCS was 6 (IQR 3-14). Mean results for cerebral hemodynamic parameters and blood pressures are reported in Table 1. The mean clevidipine dose was 9.2 mg/hour (range 1-21 mg/hr). The average duration of data collection for the clevidipine infusion was 9.36 hours (range 3.97-11.95 hours).

**Conclusions:** These results suggest clevidipine is effective in reducing blood pressure and does not have a negative impact on cerebral hemodynamic parameters.

**References**

1. Nishiyama T, et al. Can J Anaesth 47(12):1196-201, 2000

**Table 1 (abstract P396).** Results

Perfusion Parameter (mm Hg)	Pre-Clevidipine	Post-Clevidipine	P-Value
Mean ICP	11.31	11.69	0.837
Mean CPP	77.53	69.88	0.004
Mean SBP	149.88	136.41	0.001

## P397 Intracranial hemorrhage in HELLP syndrome patients admitted to the ICU

### T Yuyen, S Kongsayreepong, A Piriyapatsom

#### Department of Anesthesiology, Siriraj Hospital, Mahidol University, Bangkok, Thailand

**Introduction:** Intracranial hemorrhage is a rare & serious complication with high mortality of HELLP syndrome (hemolysis, elevated liver enzymes & low platelet count). The aim of this study is to find the significant predictor of intracranial bleeding in the HELLP syndrome patient admission to the ICU.

**Methods:** This prospective observational study was done in all obstetric patients admission to the general SICU of a tertiary university hospital during Jan 2013-Oct 2017. Data recording include patient demographic data, comorbidities, parity, ASA status, perioperative antihypertensive medication & BP, intraoperative blood loss, type & amount of fluid/blood & blood component, perioperative data: Hb, platelet count, liver function, coagulogram (PT, INR, fibrinogen), perioperative adverse event, ventilator/ICU days & ICU mortality.

**Results:** There were 115 HELLP patients out of 422 obstetric patients admitted to the ICU. 19 HELLP patients (16%) developed intracranial hemorrhage during ICU admission with the severity started from small to massive intracranial hemorrhage who need craniotomy with clot removal. 17 patients (36%) die. One patient survived from massive intracranial hemorrhage after wide craniectomy & therapeutic hypothermia went home with mild hemiparesis. 3 patients developed intracranial hemorrhage on the day 3-4 after delivery. All patients still have elevated liver enzyme during hemorrhage. HELLP patients who had intracranial hemorrhage were significant (p<0.05) older, poor perioperative blood pressure control, lower platelet and fibrinogen, higher INR ratio & liver enzyme.

**Conclusions:** Intracranial hemorrhage in HELLP patient is a high morbidity & mortality. Tight BP control & correction of platelet, fibrinogen, INR are needed during the peripartum period until the liver dysfunction come back to normal.

## P398 Systemic inflammatory response syndrome as predictor of poor outcome in non-traumatic subarachnoid hemorrhage patients

### V Rass^1^, M Gaasch^1^, M Kofler^1^, A Schiefecker^1^, B Ianosi^2^, P Rhomberg^1^, R Beer^1^, B Pfausler^1^, E Gizewski^1^, C Thomé^1^, E Schmutzhard^1^, R Helbok^1^

#### ^1^Medical University Innsbruck, Innsbruck, Austria,^2^Institute of Medical Informatics, Hall, Austria

**Introduction:** Subarachnoid hemorrhage (SAH) is a serious life threatening disease associated with high mortality and morbidity. A substantial number of patients develop systemic inflammatory response syndrome (SIRS). Here we aimed to identify risk factors for SIRS development and to evaluate the role of SIRS-burden on patients’ outcome.

**Methods:** Prospectively collected data from 297 consecutive non-traumatic SAH patients admitted between 2010-2017 were analyzed. SIRS was diagnosed based on >=2 criteria (hypo/hyperthermia, tachypnea, leukopenia/leukocytosis, tachycardia) and defined as early (<=3d) and delayed SIRS (d6-d10). SIRS-burden was defined as the percentage of SIRS-positive days within the first 10d. Multivariate analysis was used to analyze risk factors for the development of early and delayed SIRS as well as the relationship of SIRS with poor functional outcome [3-month modified Rankin Scale (3m-mRS)>=3].

**Results:** 78% of SAH patients had early SIRS and 69% delayed SIRS. Median SIRS-burden within the observation period of 10d was 60% (IQR=10-90%). Risk factors for early SIRS were poor admission grade (OR=1.74, 95%-CI=1.12-2.72, p=0.013), aneurysm clipping (OR=6.45, 95%-CI=1.35-30.81, p=0.019), hydrocephalus requiring EVD placement (OR=3.04, 95%-CI=1.02-9.03, p=0.046) and a higher modified Fisher Scale score (OR=1.78, 95%-CI=1.19-2.64, p=0.005). Patients with early SIRS were at increased risk to develop delayed SIRS (OR=3.54, 95%-CI=1.32-9.51, p=0.012). With every 1 point increase of the SIRS score patients had a 1.7 higher odds for poor functional outcome (95%-CI=1.33-2.11, p<0.001). SIRS-burden had the highest predictive value for outcome compared to early and delayed SIRS (p=0.002).

**Conclusions:** SIRS is common after SAH and significantly contributes to poor functional outcome independently of established risk factors. SIRS is more frequent in poor admission grade SAH patients and patients undergoing aneurysm clipping. SIRS-burden may more accurately predict poor functional outcome than early SIRS alone.

## P399 Subarachnoid hemorrhage outcomes in a large Brazilian cohort – a multicentric study

### B Goncalves^1^, R Turon^1^, C Rynkowski^2^, N Lima^2^, F Miranda^1^, N Melo^1^, M Prazeres^1^, F Bozza^3^, P Kurtz^1^, C Righy^1^

#### ^1^Instituto Estadual do Cerebro Paulo Niemeyer, Rio de Janeiro, Brazil,^2^Hospital Cristo Redentor, Porto Alegre, Brazil,^3^Fundação Oswaldo Cruz, Rio de Janeiro, Brazil

**Introduction:** Aneurysmal subarachnoid hemorrhage (SAH) is an acute cerebrovascular event with high mortality and is an important cause of neurologic disability among survivors. Many complications in the course of SAH, such as hydrocephalus, also play a role in the poor outcome. The aim of the study was to describe the characteristics of patients with SAH admitted to the ICU to evaluate the factors associated with outcome.

**Methods:** This study was conducted in two reference centers – one in Rio de Janeiro and one in Porto Alegre. From July 2015 to September 2017, every adult patient admitted to the ICU with aneurysmal SAH was enrolled in the study. Data were collected prospectively during hospital stay. The primary endpoint was mortality and dichotomized functional outcome (poor outcome defined as Glasgow Outcome Scale 1 to 3) at hospital discharge and 12 months. Dichotomous variables were analyzed using two-tailed Fisher’s exact test.

**Results:** A total of 261 patients were included. Demographic characteristics are presented in Table 1. Frequency of clinical and neurological complications are presented in Table 2. In univariate analysis, factors most frequently seen in patients with unfavorable outcome were seizure (17% vs 3%, p=0.0003), hydrocephalus (51% vs 17%, p<0.0001), meningitis (30% vs 12%, p=0.0004), rebleeding (17% vs 4%, p= 0.0008), vasospasm (46% vs 26%, p=0.001), pneumonia (37% vs 7%, p<0.0001), sepsis/septic shock (21% vs 4%, p<0.0001), post-surgical neurological deterioration (37% vs 18%, p=0.001) and delayed cerebral ischemia (37% vs 12%, p<0.0001). At 12 months, out of 74 patients with follow-up, 40% had poor outcome.

**Conclusions:** SAH is associated with high morbidity. Both neurological complications as clinical complications were associated with unfavorable outcomes. Therapeutic interventions to prevent those may have an impact on clinical outcomes.

**Table 1 (abstract P399).** Demographic characteristics

	N = 261
Female gender	190 (73%)
Median age	55 (19-87)
Poor grade (WFNS 4-5)	70 (27%)
Modified Fisher scale 3-4	176 (67%)
Death	46 (18%)
Poor outcome (GOS 1-3)	131 (50%)

**Table 2 (abstract P399).** Frequency of main complications

Complication	Frequency
Seizure	26 (10%)
Hydrocephalus	89 (34%)
Meningitis	54 (21%)
Rebleeding	27 (10%)
Pneumonia	58 (22%)
Sepsis/septic shock	32 (12%)
Delayed cerebral ischemia	64 (25%)

## P400 Associated factors with brain tissue hypoxia in sah patients with protocolized brain oxygen optimization

### V Rass^1^, D Solari^2^, M Gaasch^1^, B Ianosi^3^, M Kofler^1^, A Schiefecker^1^, J Miroz^2^, P Morelli^2^, R Beer^1^, B Pfausler^1^, C Thomé^1^, M Oddo^2^, R Helbok^1^

#### ^1^Medical University Innsbruck, Innsbruck, Austria; ^2^Centre Hospitalier Universitaire Vaudois (CHUV), University of Lausanne, Lausanne, Switzerland; ^3^UMIT: University for Health Sciences, Medical Informatics and Technology, Hall in Tirol, Austria

**Introduction:** Brain tissue hypoxia (brain tissue oxygen tension, PbtO2<20mmHg) is common after subarachnoid hemorrhage (SAH) and associated with poor outcome. Recent data suggest that brain oxygen optimization is feasible and may reduce the time with brain tissue hypoxia to 15% in patients with severe traumatic brain injury [1]. Little is known about the effectiveness of protocolized treatment approaches in poor-grade SAH patients.

**Methods:** We present a retrospective analysis of prospectively collected data of 105 poor-grade SAH patients admitted to 2 tertiary care centers where PbtO2<20mmHg was treated using an institutional protocol. Treatment options were left to the discretion of the treating neuro-intensivists including augmentation of cerebral perfusion pressure (CPP) using vasopressors if necessary, treatment of anemia and targeting normocapnia, euvolemia and normothermia. The dataset used for analysis was based on routine blood gas analysis for hemoglobin data matched to 2 hourly averaged data of continuous CPP, PbtO2, temperature and cerebral microdialysis (CMD) samples over the first 10 days of admission.

**Results:** Patients were admitted with a GCS of 3 (IQR 3-4) and were 58 (IQR 48-66) years old. Overall incidence of brain tissue hypoxia was 25%. During this time we identified associated episodes of CPP<70mmHg (27%), hyperglycolysis (CMD-lactate>4mmol/L, CMD-pyruvate>120μmol/L; 26%), pCO2<35mmHg (19%), metabolic distress (CMD-lactate-to-pyruvate-ratio>40; 18%), PaO2<80mmHg (14%), Hb<9g/dL (10%), and temperature>38.3°C (4%) (Fig. 1). Of these variables only hyperglycolysis was significantly more common (37%) during episodes of normal PbtO2 (75% of episodes).

**Conclusions:** Our results present clinical data of protocolized PbtO2-targeted therapy and show that there is room for further optimization. A larger cohort with predefined interventions is needed to proof the effect on longterm outcome after SAH.

**References**

1. Okonkwo DO et al. Crit Care Med 45:1907-1914, 2017

**Fig. 1 (abstract P400).**
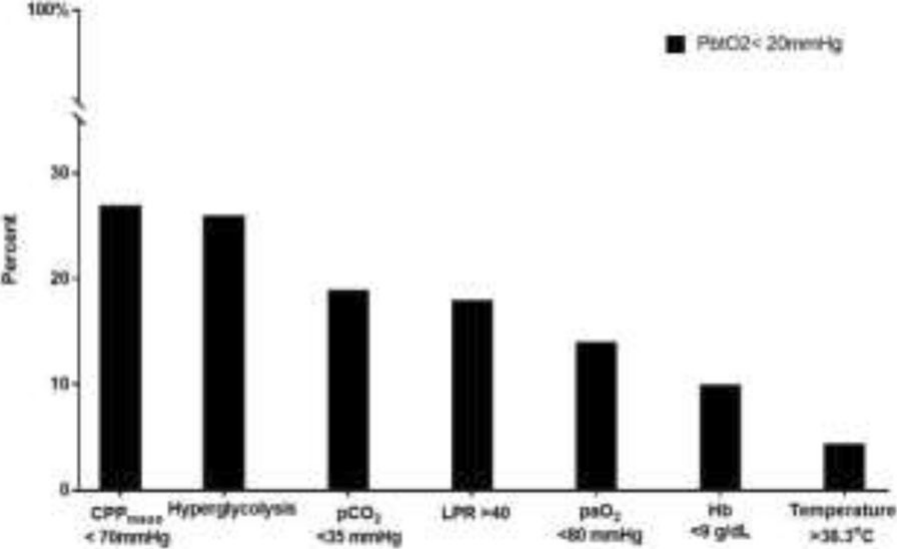
The black bars represent the percentage of abnormalities of factors shown in the x-axis during brain tissue hypoxia.

## P401 Intracranial hemorrhage after thrombolysis for ischemic stroke: life threatening complication or irrational fear?

### G Karlis^1^, N Magkas^2^

#### ^1^General Hospital of Rodos, Rodos, Greece; ^2^Ippokrateion General Hospital, Athens, Greece

**Introduction:** The NINDS study in 1995 established intravenous thrombolysis (IVT) with recombinant tissue plasminogen activator (rt-PA) within 3 hours of symptoms onset as the treatment of choice for acute ischemic stroke (AIS). The time-window for administering IVT was expanded to 4.5h after the results of the ECASS-3 trial in 2008. To date intravenous rt-PA remains the only approved systemic therapy for AIS with proven efficacy in resolving neurological deficits and improving functional outcomes. Nevertheless, only a very small proportion of AIS patients (2-3%) are treated with thrombolysis. The most feared complication of IVT is intracranial hemorrhage (ICH). ICH is a potentially fatal adverse event, thus an exhaustive list of contraindications has been implemented in stroke guidelines to minimize the risk and to assist the selection of patients that will gain the most of the benefit from IVT. On the other hand the application of such a stringent protocol can be discouraging and may reduce the number of patients that could possibly benefit from the treatment.

**Methods:** We performed a comprehensive review of the recent literature regarding the hemorrhagic complications of rt-PA in AIS with emphasis in ICH. The electronic database of PubMed was searched to retrieve publications of relevant original studies. We reviewed articles from January 1995 to October 2017.

**Results:** Symptomatic ICH ranged from 2.4% to 11% in randomized ?A3B2 show $132#?>trials with the usual rt-PA dose of 0.9mg/kg. In studies respecting the 4.5h window the rate of ICH did not exceed 7.3%. There is no study showing significantly worse mortality rate for patients treated with rt-PA.

**Conclusions:** Underutilization of IVT despite the overwhelming evidence that support the effectiveness of such therapy can be partly attributed to the fear of hemorrhagic complications. This fear is not justified by current data. The estrangement of the emergency medicine community regarding IVT and the domination of stroke experts in decision making is also a barrier.

## P402 Regional wall motion abnormalities and reduced global longitudinal strain is common in patients with subarachnoid hemorrhage and associated with markedly elevated troponin

### K Dalla

#### Sahlgrenska University Hospital, Gothenburg, Sweden

**Introduction:** Stress-induced cardiomyopathy after subarachnoid hemorrhage (SAH) is a life-threating condition associated with poor outcome. Regional wall motion abnormalities (RWMA) is a frequent finding, however, assessment of RWMA is known to be difficult. In the present study we hypothesized that global and regional longitudinal strain (GLS and RLS) assessed with speckle tracking echocardiography can detect myocardial dysfunction indicated by increased levels of the cardiac biomarker troponin (TnT).

**Methods:** This prospective study comprised 71 patients with SAH. The TnT was followed daily from the admission up to 3 days post-admission and elevated TnT was defined as > 80 ng/l. A transthoracic echocardiography examination was performed within 48 hours after the hospitalization. The peak GLS was determined using the three apical projections and presented as the mean of the 18 segments. Reduced GLS was defined as > -15% and reduced RLS was considered present when segmental strain was > -15% in > 2 adjacent segments.

**Results:** The TnT was increased in 17 (24%) patients. The TnT (ng/l) level in patients with RWMA (n=12) (median, (25% and 75% percentile)) was 648 (338-750), in patients with reduced GLS (n=12) 502 (107-750) and in patients with reduced RLS (n=42) 40 (10-216) respectively. Among patients with normal TnT had one patient RWMA (2%) and three patients reduced GLS (6%). The Table shows the diagnostic performance regarding detection of patients with increased TnT.

**Conclusions:** The presence of RWMA and reduced GLS is a relatively common finding in patients following SAH and indicate markedly increased levels of TnT. Normal TnT reduces the likelihood of LV systolic dysfunction. Assessment of RLS by speckle tracking is not helpful in the assessment of patients with SAH.

## P403 Off-label thrombolysis for ischemic strokes: the challenge to overcome guidelines

### P Papamichalis^1^, V Zisopoulou^1^, E Dardiotis^2^, S Karagiannis^1^, D Papadopoulos^1^, T Zafeiridis^1^, A Pappa^1^, D Babalis^1^, E Neou^1^, I Staikos^1^, I Staikos^1^, A Skoura^1^, G Hadjigeorgiou^2^, A Komnos^1^

#### ^1^General Hospital of Larissa, Larissa, Greece; ^2^University Hospital of Larissa, Larissa, Greece

**Introduction:** Deviations from strict eligibility criteria for intravenous thrombolysis (IVT) in ischemic strokes regarding either license contraindications to alteplase or relative contraindications to thrombolysis have been reported in international literature, with conflicting results on patients’ outcome.The aim of our study was to evaluate safety and efficacy for patients receiving IVT outside standard inclusion criteria.

**Methods:** Retrospective analysis of our department’s thrombolysis database.We compared 83 patients with strict protocol adherence (strict protocol group) [mean age 63 years and National Institutes of Health Stroke Scale (NIHSS) at admission 12/range 5–28] and 41 patients with protocol deviations (off-label group) [mean age 68 years and NIHSS at admission 10/range 2–24],in particular 10 patients >80 years old, 13 patients with mild stroke-NIHSS< 5,and 22 with symptom-to-needle time 3-4.5 hours (4 patients had 2 deviations).

**Results:** Patients in the off-label group were older but had no difference in baseline severity scores (SAPSII, NIHSS). They had no statistically significant difference on short-term (NIHSS at 7 days, need for critical care support, primary adverse event) and long-term (mortality,functional outcome at 3 months) outcome measures when compared to standard protocol patients.

**Conclusions:** In accordance with international literature,off-label thrombolysis is save and equally effective to standard protocol thrombolysis.Thrombolysis strict protocol needs expansion of inclusion criteria.

## P404 Neuron-specific-enolase as marker of acute brain injury and efficacy of therapy

### V Artemenko^1^, A Budnjuk^2^, A Kartashov^3^

#### ^1^MC INTO-SANA, Odessa, Ukraine; ^2^ONMedU, Odessa, Ukraine; ^3^Odessa Regional Hospital, Odessa, Ukraine

**Introduction:** Most scales (GCS,NIHSS) don’t consider the pathway of secondary acute brain failure (sABF). Neuron-specific-enolase (NSE) could be usefull in diagnostic and treatment pts. with sABF [1,2].

**Methods:** Prospective study incl. 35 pts. with ABF. Pts. were identical in condition, age and comorbidies. In main group, NSE examed and choline alfoscerate was used, pts. was divided into 2 subgroups Ia (n=12) with acute ischemic stroke(AIS) and Ib (n=10) pts. with posthypoxic encephalopathy. The control group (n=13) pts. with AIS treated by Loc.Protocol №602. Clinical, laboratory, and imaging variables were fully compared. Pts. examed by ABCDE algorithm, GCS and NIHSS. Brain CT, Carotid Doppler performed. Considering criteria:NSE(days 1,3,5), neurological status, length of stay in ICU (ICU LOS). "SS-6.0"was used.

**Results:** The baseline NSE was higher and correlated to NIHSS (16.3±2.2, ÷2=1.08) in all pts. In Ia, Ib sbgroups NSE decreased for 3-5 days vs. control group 10-12days (÷2=7.93) and correlated with regression neurological deficit. ICU LOS in main group was 3.8±0.9 days vs. control group 5.9±0.9 days. Sensitivity and specificity of NSE as a marker of brain injury in pts. with AIS were 65 and 83% and in posthypoxic pts. were 90 and 90%, respectively, which showed NSE as eligible diagnostic criterion of posthypoxic cerebral edema. In Ia (AIS) pts. and Ib (posthypoxic edema) were confirmed by increasing NSE in 4-fold and 9-fold respectively more vs. pts. who had only Brain CT at first day. NSE also correlated with regression neurological deficit and improving of the neurological status. Although, two pts. In IIb group died with NSE 150-220 ìg/ml

**Conclusions:** 1. NSE is an effective marker of the severity of damages even in the sABF, and shoved efficacy efficacy of treatment. 2. Negative outcome can be in pts. with sABF and more 3-fold increasing NSE and increasing up to 150-220 ìg/ml is a mortality predictor. 3. We included NSE in local protocols

## P405 N-terminal pro-brain natriuretic peptide as a bio-marker of the acute brain injury

### N Dryagina, L Tsentsiper, A Kondratyev

#### Russian Polenov’s Neurosurgical Institute, Sankt-Petersburg, Russia

**Introduction:** The detection of biomarkers levels facilitates an early diagnosis of brain tissues damage, allows assessing the prognosis of the disease and its outcome, and performs the monitoring of the patient treatment.

**Methods:** We studied 64 patients (36 m, 28 f.). 1st group comprised 12 patients with severe brain trauma: 1a – survivors with good outcome (on Glasgow outcome scale groups I-II) (n=8), 1b – dead or severely disabled (on Glasgow outcome scale groups III-V) (n=4). 2nd group comprises 37 patients with intracranial and sub-arachnoid hemorrhages: Assignment to groups 2a (n=14), 2b (n=22) was done using the same criteria as group 1. 3rd group comprises 16 patients operated in conjunction with brain tumor. Assignment to groups 3a (n=6) and 3b (n=10) was done using the same criteria as groups 1 and 2. We tested the level of N-terminal Pro-Brain natriuretic peptide in blood (0-125 pg/ml) between 1st and 3rd days after severe brain injury and then every 2-12 days for the total duration of 21 days.

**Results:** : Statistical analysis failed to demonstrate noticeable difference in the level of NTproBNP between groups 1,2,3. We detected the differences between subgroups (p<0.01). Patients from groups 1a,2a,3a (n=28) NTproBNP level stayed below 700 pg/ml in 20 cases (71%), in the 8 cases (29%) the level was above 700 pg/ml, but by 14-21th day decreased to the normal values. For patients in subgroups 1b,2b,3b (n=36) in 28 cases (78%) NTproBNP level was above 700 pg/ml at least once, in 8 cases (12%) level stayed below 700 pg/ml but remain high for the entire duration of the study without significant decrease.

**Conclusions:** All the patients with acute brain injury show the increased level of NTproBNP above normal values, irrespective of ethiology of injury. In case when NTproBNP level increases above 700 pg/ml and/or does not decrease to the normal values it is possible to predict a negative outcome.

## P406 Troponin level as a predictor of neurological outcome & mortality in acute ischemic strokes

### E El gammal, M Soliman, M Mohamed, D Ragab

#### Cairo university, Cairo, Egypt

**Introduction:** Cerebrovascular and coronary artery diseases share many of the same risk factors [1]. Cardiac mortality accounts for 20% of deaths and is the second commonest cause of death in the acute stroke population, second only to neurologic deaths as a direct result of the incident stroke.

**Methods:** This is a prospective observational study from July 2015 to April 2016 done on 80 adult patients (groupI: 50 pts acute ischemic strokes & group II:30 pts as control) in Kafr-Elsheikh general hospital ICU. Inclusion criteria: All patients with acute ischemic stroke while Exclusion criteria: Patients with heart or renal failure/sepsis&septic shock/Ischemic heart disease or Hemorrhagic stroke,full clinical examination&labs including admission quantitative serum cardiac Troponin I ELISA immunoassay,ECG,2D echocardiography&CT brain on day 0&3,Alberta stroke program (ASP) early CT (ASPECT) to predict neurological outcomes and mortality in patient with acute ischemic stroke within 28 days so survivors Vs non-survivors in group 1 were divided to G1A & G1B respectively.

**Results:** Dyslipidemia, hypertension, diabetes mellitus were significant comorbidities in all ischemic stroke pts.TLC, Urea, INR and Troponin were significantly higher in case group Vs control group.GCS was found to be lower in non-survivors at day 0&at 3rd day follow up while ASPECT was significantly lower only at 3rd day follow up.Troponin level was significantly higher in non-survivors G1B, it was also higher in patents who developed convulsion later during their ICU stay& it was significantly inversely correlated to GCS and ASP. troponin had sensitivity 53% and specificity 87% (ROC curve analysis)

**Conclusions:** Troponin level was predictor for mortality in patient with acute ischemic stroke.it is well correlated to GCS and ASP on admission.it was a predictor for occurence of convulsions later in ICU stay.

**References**

1. Prosser J et al. Stroke 38:2295–2302, 2007

## P407 Hyperkinetic disorder treatment using sevoflurane in patients with unresponsive wakefulness syndrome (UWS) and minimal conscience state (MCS)

### E Kondratieva, N Lesteva, A Kondratiev, S Kondratyev

#### Polenov Neurosurgical Institute, Saint Petersburg, Russia

**Introduction:** Based on examination and treatment of hyperkinetic disorder in patients with UWS and MCS, we supposed that hyperkinesis manifesting the formation of the generator of pathologically enhanced excitation in cerebral cortex, basal ganglia, which subsequently causes the formation of hyperkinesis. Halogen-containing anesthetic sevoflurane had a good clinical effect in patients with prolonged impairment of consciousness.

**Methods:** The study included 5 patients with UWS (4 - hypoxia, 1 -encephalitis) and 3 patients with MCS (2 - hypoxia, 1-encephalitis). Hyperkinetic disorder presenting as permanent myoclonus of arms and legs, face. All patients were performed head MRI and EEG (before, during and after anesthesia), CRS-R assessment, 3 patients - [18F]-FGD PET. Initial anesthesia: propofol 2-3 mg/kg, rocuronium bromide (Esmeron) 0, 6 mg/kg, fentanyl 3–5 mg/kg and clonidine (clophelin) 0.5-0.7 mg/kg. Maintenance of anesthesia is carried out due to the following scheme: inhalation anesthesia using Sevoflurane (2.0-3.0 vol%, MAC 0.8-0.9). Additionally, during the 2nd - 4th hours of medical anesthesia was prescribed the intravenous injection using Ketamine 1-2 mg/kg/hr. The anesthesia is used during 24 hours. The patients were nurtured by balanced mixtures through nasogastric tube. After 24 hours the patients were gradually transferred to the autonomous breathing. The control clinical and instrumental studies to evaluate the therapy effectiveness (EEG, CRS-R) were performed.

**Results:** In 5 patients (2 MCS, 3 UWS) was observed the hyperkinetic disorder regression as decrease of hyperkinesis manifestation, 3 patients didn’t have a significant dynamics.

**Conclusions:** The artificially formed “pharmacological dominant” (using sevoflurane and Ketamine) may decrease the activity of pathological system of the brain, which clinically presented as significant decrease of hyperkinesis manifestation in 5 out 8 patients.

## P408 15-year experience of using benzodiazepines in predicting outcomes and targeted treatment of patients in unresponsive wakefulness syndrome (UWS).

### E Kondratieva

#### Polenov Neurosurgical Institute, Saint Petersburg, Russia

**Introduction:** We accepted a hypothesis that in some patients UWS is a consequence of a pathologic system (PS), that limits the brain functional activity. Identification of a PS allow to predict consciousness recovery. EEG registration under benzodiazepines test (BT) has become the method of PS identifying in UWS patients.

**Methods:** We examined 145 UWS patients (74 - traumatic, 71 -non traumatic). CRS scales assessment, EEG with BT, MRI of brain were performed for all patients. The midazolamum was administered iv 0.04 mg/kg,.In 3-4 min after BZD was recorded EEG for 15 min. The test was considered to be positive if against the background of BZD EEG pattern restructuring was observed: the low-amplitude EEG activity was rebuilt with the advent of alpha- and beta- spectrum.In patients with slow-wave activity of theta- and delta- spectrum appeared stable fast forms, and in patients with baseline polymorphic EEG pattern was recorded prevalence of alpha activity and (or) the alpha rhythm. In order to confirm the correlation between the BZD effect and EEG pattern restructuring, Flumazenil was administrated at rate of 0.1 mg every 1 to 2 minutes until the original EEG pattern has been registered again.

**Results:** The BT was true positive (recovery consciousness in 3-12 month later) in 22 traumatic and 19 non traumatic patients. True negative (permanent UWS 12 month later) in 27 traumatic and 43 non traumatic patients. False positive - 11 traumatic, 4 non traumatic. False negative 14 traumatic, 5 non traumatic patients. Sensitivity BT to VS/UWS = 74.6% Sensitivity to MCS = 43.1%

**Conclusions:** Our data confirmed the correctness of hypothesis that a PS limits the activity of the brain in patients in a UWS. We proposed diagnostic method of a PS activity and suppression. Apparently, BZD are the drugs of first stage examination choice in the treatment of UWS patients.

## P409 Early identification of sepsis-associated encephalopathy with EEG is not associated with short-term cognitive dysfunction

### C Maenhout^1^, L Ferlini^1^, I Crippa^1^, FS Taccone^1^, J Creteur^1^, H Slama^1^, P Peigneux^2^, N Gaspard^1^

#### ^1^Erasme Hospital, 1000, Anderlecht, Belgium; ^2^Université Libre de Bruxelles - Neurosciences Institute, Bruxelles, Belgium

**Introduction:** Septic-associated encephalopathy (SAE) affects approximately 75% of septic patients. Recent studies showed SAE is associated with short-term mortality and long-term cognitive disability. However, diagnosis of SAE is one of exclusion and its association with short-term cognitive deficit is uncertain. The aim of this study is to evaluate the sensitivity of clinical examination in detecting SAE. The association between SAE and short-term cognitive impairment is also assessed.

**Methods:** Prospective observational study enrolling adult septic patients admitted to a mixed ICU. Exclusion criteria were: encephalopathy from another cause, history of psychiatric/neurologic disease, cardiac surgery. All patients received continuous EEG monitoring and were assessed for SAE for up to 7 days after inclusion. We performed a comprehensive consciousness assessment twice daily during the ICU (GCS; Full Outline of UnResponsiveness, FOUR; Coma Recovery Scale-Revised, CRS-R; Reaction Level Scale 85, RLS85; Confusion Assessment Method for the ICU, CAM-ICU). We defined altered brain function as GCS<15, FOUR<16, CRS-R<23, RLS85>1, or positive CAM-ICU. Modified Synek scale was applied to EEG interpretation. After discharge, we assessed cognitive functions with Montreal Cognitive Assessment and Frontal Assessment Battery.

**Results:** We performed 204 clinical evaluations on 38 patients (Jan 2016-Oct 2017). GCS, FOUR, CSR-R and RLS85 detected SAE in 147, 113, 140 and 139 cases respectively. CAM-ICU was positive in 57/111 cases. EEG was altered in all patients. EEG alteration correlated with clinical evaluation (GCS - r2 0.38, FOUR - r2 0.32, CRS-R - r2 0.43, RLS8 - r2 0.42 and CAM-ICU, p<.001). No correlation between cognitive function at hospital discharge and severity of EEG alteration was found.

**Conclusions:** EEG was more sensitive than clinical assessment in detecting SAE. Altered EEG was not associated with short-term cognitive function.

## P410 Analysis of the training needs in Italian centers that use brain ultrasound in their daily practices: a descriptive, multicenter study

### R Aspide^1^, M Pegoli^1^, G Morello^2^, G Varelli^3^, M Casartelli Liviero^4^, P Gritti^5^, F Zumbo^6^, F Bilotta^7^

#### ^1^IRCCS ISNB Bellaria hospital, Bologna, Italy; ^2^ARNAS Garibaldi, Catania, Italy; ^3^Nuovo S.Chiara hospital, Pisa, Italy; ^4^University Hospital Borgotrento, Verona, Italy; ^5^Papa Giovanni XXIII hospital, Bergamo, Italy; ^6^Bufalini hospital AUSL Romagna, Cesena, Italy; ^7^"Sapienza" University, Roma, Italy

**Introduction:** As mission of SIAARTI Neuroanesthesia and NeuroICU group of study, we are mapping out the Brain Ultrasound training needs in our Centers. Although Brain Ultrasound is widely used to study the intracranial vessels and other issues, it is still not clear the homogeneity of the skills required in both Neuro and General ICU in Italy. The aim of this study is to explore the use of US-TCD and validate a collection of criterea which would prove useful in any future national wide survey.

**Methods:** Starting from Sept. 2017 the seven Center involved (Bologna, Catania, Pisa, Verona, Bergamo, Cesena, Roma) collected clinical and sonographic data, basing on a CRF of twenty criteria such as: kind of hospital and ICU, number of beds and neuro-patients/year, the physicians specialization trained to perform US-TCD, the kind of US doppler device used and the kind of training course followed. As a second step, data were analyzed by coordination team, as third step, during annual SIAARTI conference, these Centers had a deep discussion on these selected items, further modifying and adapting the content of the items.

**Results:** The result is a ready list of 20 items, an available tool for all the participant Centers, that are going to start with an internal test survey for a final validation.

**Conclusions:** There is more than one path to train a physician on Brain US in Italy and there are new possible applications, even outside of the Neuro sub-speciality. From the preliminary discussion, it is clear that in Italy we have a inhomogeneous frame of training and use. This group of study believes that among the anesthesiologists/intensivists, it is possible to find a useful number of physicians interested in training on this topic. The main aim is the production of a validated criterea collection, available for eventually future national survey, useful to help map out the real national training needs in Italy on US Brain.

## P411 Perinatal neurosurgical admissions to intensive care

### C Nestor, R Hollingsworth, K Sweeney, R Dwyer

#### Beaumont Hospital, Dublin, Ireland

**Introduction:** Beaumont Hospital is the Neurosurgical centre for Ireland serving a population of 3.6 million. We present data on all perinatal patients who required ICU admission for Neurosurgical conditions over an 8 year period. Our data presents an insight into the incidence and outcome of Neurosurgical conditions during pregnancy

**Methods:** Searching our database identified 11 pregnant and 8 recently pregnant patients admitted to ICU with neurosurgical conditions. Patient data was collected retrospectively by review of charts and of an electronic database. A further 12 pregnant patients were admitted for Neurosurgical intervention but did not require critical care.

**Results:** Intracranial haemorrhage was the most common diagnosis (5 subarachnoid haemorrhage and 5 had intra-cerebral haemorrhage). 6 patients presented with intracranial tumours and 1 patient had a traumatic brain injury. 1 patient was admitted post spinal tumour resection. 1 patient was referred with an ischemic stroke after iatrogenic injury to the carotid and vertebral artery. The requirement for organ support in this cohort of patients was high; 64% required ventilation and 45% inotropes. 13 patients underwent neurosurgical intervention & 6 medical treatment. 2 maternal deaths occurred at 16 & 37 weeks gestation. The modified Rankin Score (mRS) on discharge from hospital was <= 2 for 9 of the 17 surviving patients (median=3). Of the 11 pregnancies (all singleton) there were 3 foetal deaths. 1 patient miscarried spontaneously at 4 weeks, 1 had a medical termination of pregnancy at 12 weeks to facilitate chemotherapy and 1 foetus died after maternal death at 16 weeks. The 8 remaining patients delivered normal babies.

**Conclusions:** Neurosurgical disease requiring ICU admission during pregnancy is rare; our data suggest an incidence of 1 case per 2 million population. Maternal outcomes were mixed with more than half having a mRS>2 on discharge. Foetal outcomes were good with only one miscarriage and good neurological outcome in all surviving infants.

## P412 Differential effects of bystander-performed chest compressions and prehospital advanced life support on out-of-hospital cardiac arrest outcomes

### H Inaba^1^, Y Takei^1^, H Kurosaki^1^, K Ohta^2^, Y Myojo^2^, Y Wato^3^

#### ^1^Kanazawa University Graduate SChool of Medicine, Kanazawa, Japan; ^2^Ishikawa Prefectural Central Hospital, Kanazawa, Japan; ^3^Kanazawa Medical University, Uchinada, Japan

**Introduction:** Quality of chest compressions (CCs) before emergency medical service (EMS) arrival in addition to prehospital advanced life support (ALS) after EMS arrival may influence the outcomes of out-of-hospital cardiac arrest (OHCA).

**Methods:** In this prospective study of 4,253 OHCAs that received bystander-performed CCs between October 2010 and December 2016, CC quality was determined in 3,759 cases by EMS personnel on their arrival at the scene. Stepwise multivariable analyses that included interaction between time of day and arrest location were performed in a stepwise manner.

**Results:** Prehospital ALS (adjusted OR, 1.63; 95%CI, 1.38-1.93) but not good-quality of bystander-performed CCs (1.02, 0.84-1.26) was associated with sustained return of circulation (ROSC). Neither provison of good-quality CCs nor prehospital ALS was a major factor associated with on-month survival. However, good-quality of bystander-performed CCs (2.44, 1.81-5.00) in addition to shockable rhythm (13.3; 8.70-20.4) and bystander-witnessed OHCA (4.79; 2.98-8.00) were associated with higher chances of neurologically favourable one-year survival, whereas prehospital ALS (0.21; 0.10-0.39) and elderly OHCA (0.47; 0.31-0.73) were associated with lower chances of the survival (Fig. 1). The impact of good quality CCs on survival were preserved in bystander-witnessed OHCAs with shockable initial rhythm. Non-central region (adjusted OR for good-quality, 0.46; 95%CI, 0.39-0.54), lack of BLS training experience (0.47; 0.36-0.62), elderly-only rescuers (0.53; 0.44-0.65), CC initiation following dispatcher-assisted cardiopulmonary resuscitation (0.71; 0.55-0.91), and female-only rescuer (0.77; 0.65–0.90) were associated with poor-quality CCs. CC quality in at-home OHCAs remained low throughout the day, whereas that in out-of-home OHCAs decreased during night-time.

**Conclusions:** Provision of good-quality CCs before EMS arrival but not prehospital ALS was essential for neurologically favourable survival.

**Fig. 1 (abstract P412).**
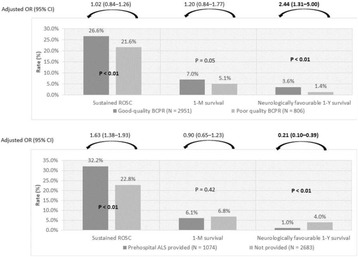
Major factors associated with good-quality bystander-performed chest compressions

## P413 New protocol for start of chest compressions before definitive cardiac arrest improved survival from out-of-hospital cardiac arrest witnessed by emergency medical service

### H Inaba^1^, H Kurosaki^1^, K Ohta^2^, Y Myojo^2^, Y Wato^3^

#### ^1^Kanazawa University Graduate SChool of Medicine, Kanazawa, Japan; ^2^Ishikawa Prefecture Central Hospital, Kanazawa, Japan; ^3^Kanazawa Medical University, Uchinada, Japan

**Introduction:** Healthcare providers including emergency medical service (EMS) personnel usually confirm absence of carotid pulse before starting chest compressions. At the end of 2011, Ishikawa Medical Control Council implemented new criteria for start of chest compressions encouraging EMS to start chest compressions when carotid pulse was week and/or <50/min in comatose adult patient with respiratory arrest or agonal breathing.

**Methods:** Data were prospectively collected for out-of-hospital cardiac and respiratory arrests during the period of 2008–2015. Definitive cardiac arrest was recorded when loss of carotid pulse was confirmed by pulse checks performed every 2 min after the early start of chest compressions. The effect of early chest compressions on the proportions of definitive cardiac arrest was analysed in 243 cases with respiratory arrest and circulatory depression in initial patient evaluation. Before/after comparison of neurologically favourable 1-Y survival was performed in 619 cases with EMS-witnessed OHCA.

**Results:** The early start of chest compressions did not significantly prevent definitive cardiac arrest that followed respiratory arrest with circulatory depression in the initial patient evaluation (Fig. 1). Time interval between start of chest compressions and definitive cardiac arrest confirmation (median; IQR) was 2; 1.5-3 min. The survival rate of all EMS-witnessed OHCAs after the implementation of new criteria was significantly higher than that before the implementation: adjusted OR; 95% CI, 1.86; 1.02-3.40 (Fig. 2). No complications related to early chest compressions were reported during the study period.

**Conclusions:** Start of chest compressions before definitive cardiac arrest improved survival from out-of-hospital cardiac arrest witnessed by emergency medical service. Healthcare providers including EMS personnel should be encouraged to provide chest compressions on cases with respiratory arrest and severe cardiovascular depression.

**Fig. 1 (abstract P413).**
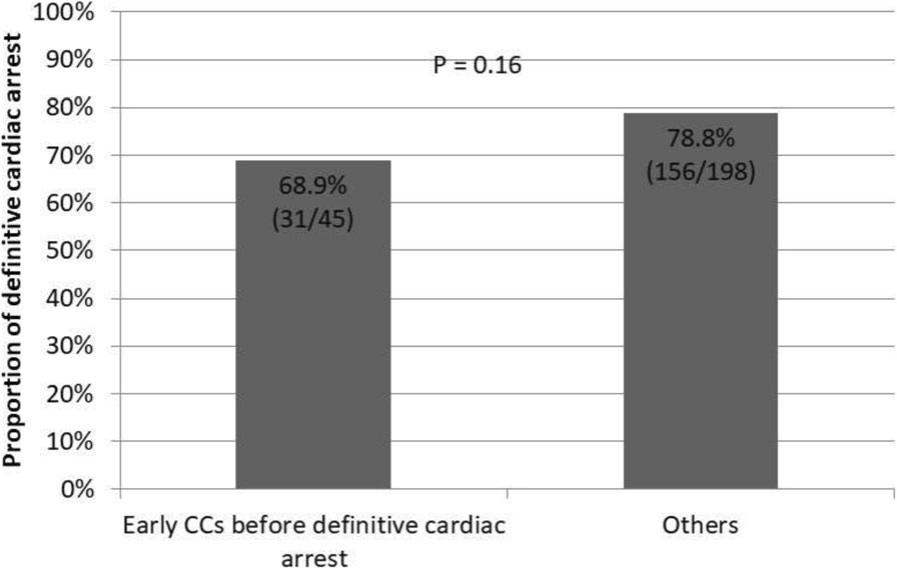
The effect of early CCs on the proportions of definitive cardiac arrest that followed respiratory arrest and circulatory depression in initial patient evaluation

**Fig. 2 (abstract P413).**
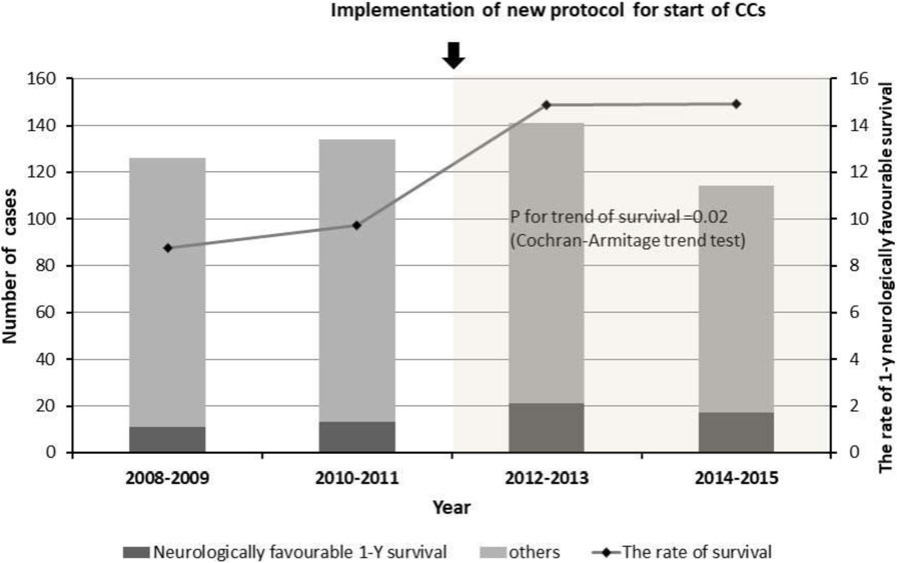
Trend in neurologically favourable 1-Y survival

## P414 Time to intubation during cardiac compressions, direct versus video laryngoscopy

### S Arcioni^1^, J Borchard^2^, A Binks^3^

#### ^1^Intensive Care, Liverpool Hospital, Liverpool, Sydney, Australia; ^2^Research Central, Wollongong Hospital, Wollongong, Australia; ^3^Anaesthesia, Wollongong Hospital, Wollongong, Australia

**Introduction:** Our study sought to determine if there is a difference in time to tracheal intubation between direct and video laryngoscopy during cardiac compressions. Guidelines suggest no more than 5 seconds should be taken to perform intubation to minimise any delay in compressions [1,2]. It is unclear if use of video laryngoscopes results in faster intubation times during cardiac arrest.

**Methods:** Observational trial involving Emergency, Anaesthesia and Intensive Care doctors. Participants’ baseline data obtained by questionnaire. Resusci-Anne™ manikin with Airway Trainer™ head [Laerdal] with grade 1 airway was utilised. Participants intubated the manikin 3 times, once with each of: MacIntosh size 3 blade, C-Mac video laryngoscope (Karl Storz, Germany) with size 3 blade and portable McGrath MAC enhanced video laryngoscope (Medtronic, USA) with size 3 blade. Order of laryngoscopes was randomised by computer generated sequence. Continuous cardiac compressions were performed throughout attempts.

**Results:** Total 54 participants. There was a statistically significant difference in time to intubation between the 3 devices using Friedman test (p<0.01). Wilcox signed-rank test demonstrated time to intubation with videolaryngoscopy was longer, C-Mac (p=0.032) and McGrath (p=0.011) compared with direct laryngoscopy. There was no significant difference between the two videolaryngoscopes (p = 0.401). When controlled for participants level of seniority and previous experience with device, direct laryngoscopy was still significantly faster than C-Mac (p = 0.009) and McGrath (p = 0.017)

**Conclusions:** Our study showed a disadvantage of video laryngoscopy during cardiac compressions. Faster intubation times with direct laryngoscopy could result in less pause in compressions and decrease periods without perfusion. Direct laryngoscopy is an appropriate first choice for tracheal intubation during cardiac arrest.

**References**

1. Soar et al; European Guidelines for Resuscitation; 100-147, 2015

2. Australian Resuscitation Council; Guideline 11.6; 2016

## P415 Impact of phone CPR on ROSC outcome

### A Giugni^1^, S Gherardi^2^, L Giuntoli^1^, A Monesi^1^, A Finelli^1^, G Gordini^1^

#### ^1^Maggiore Hospital, Bologna, Italy; ^2^Bologna University, Bologna, Italy

**Introduction:** Early cardiopulmonary resuscitation (CPR) improves survival in out-of-hospital cardiac arrest, and Phone-CPR instructions can increase the number of victims receiving CPR before Emergency Medical Service (EMS) arrival. Little is known about the impact of CPR phone instructions on the outcome of patients (pts) with return of spontaneous circulation (ROSC). The target of this study is to investigate the impact of phone instructions on mortality, and on neurological outcomes of patients who survived an out-of-hospital cardiac arrest.

**Methods:** We enrolled pts admitted to ICU after ROSC following out-of-hospital-cardiac-arrest, from 1/1/2008 to 30/06/2016; pts younger than 18, in-hospital cardiac-arrest-victims, pts who underwent cardiac arrest in health facilities, and missing data records were excluded. Written informed consensus was obtained for all pts during follow up. Data about comorbility, mortality, neurologic outcome, CPR timing according to Utstein Style, complications in ICU, metabolic state on ER admission, were collected. Study population was divided into two groups for statistical analysis: pts with immediate CPR guided by phone instructions (Phone-CPR group) and those who did not underwent immediate CPR by laic bystanders. Data were extracted from ICU, EMS databases and registered EMS phone calls.

**Results:** 172 pts met study criteria. Phone CPR were given in 25 cases, 15% of the whole study population. Results are summarized in Tables 1 and 2

**Conclusions:** Phone-CPR significantly reduced CPR-free interval. It correlates with a significative increase in shockable rhythms on EMS arrival. There is no significative reduction in mortality and in disability, even if a decrease trend can be observed. Phone-CPR seem to be a promising, effective and easy to use tool to improve survival and disability in ROSC, and should be widely applied.

**References**

1. Wnent, J. et al. Resuscitation 118: e11–e12, 2017

2. Tanaka, Y. et al. Resuscitation 83: 1235–1241, 2017

3. Viereck S. et al. Resuscitation 115: 141–147, 2017.

**Table 1 (abstract P415).** Demographic data, CPR intervals, Rhythm on EMS arrival, reperfusion

Variable	No Phone-CPR	Phone-CPR	p-Value
Age (years)	63.05 ± 16	60.85 ± 17	0.534
Male Sex (%)	64.38	80.77	0.102
Charlson Comorbility Index	2.9 ± 2.23	2.28 ±1.67	0.91
Cardiac Arrest-CPR interval (min)	12 ± 8.7	3 ± 2.2	<0.01*
Cardiac Arrest-ROSC interval (min)	27 ± 13	30 ± 17	0.52
Shockable Rhythm on ALS arrival (%)	60.27	84.62	0.017*
Coronary Reperfusion (%)	47.82	43.33	0.699

**Table 2 (abstract P415).** ICU stay, mortality and disability data

Variable	No Phone-CPR	Phone-CPR	p-Value
Length of stay in ICU (days)	7.4 ± 6.6	6.2 ± 4.6	0.41
Days of mechanical ventilation	5.7 ± 5.6	4.5 ± 3.6	0.33
ICU mortality (%)	32.87	23.08	0.953
Hospital mortality (%)	6.25	5.88	0.953
6 months after cardiac arrest mortality (%)	21.11	18.75	0.830
Overall mortality (%)	51.36	50	0.897
Good recovery after 1 year	67.31	75.00	0.607

## P416 Early hemodynamic complications in cardiac arrest patients- a substudy of the TTH-48 study

### J Hästbacka^1^, H Kirkegaard^2^, E Soreide^3^, FS Taccone^4^, I De Haas^5^, U Arus^6^, C Storm^7^, CA Sorensen^5^, CH Duez^2^, AN Jeppesen^2^, AM Grejs^2^, AI Larsen^3^, MB Skrifvars^1^

#### ^1^University of Helsinki and Helsinki University Hospital, HUS, Finland; ^2^Aarhus University Hospital and Aarhus University, Aarhus, Denmark; ^3^Stavanger university Hospital, Stavanger, Norway; ^4^Erasme Hospital, Université libre de Bruxelles, Brussels, Belgium; ^5^Aalborg University Hospital and Clinical Institute, Aalborg University, Aalborg, Denmark; ^6^North Estonia Medical Centre, Tallinn, Estonia; ^7^Charité-Universitätsmedizin Berlin, Berlin, Germany

**Introduction:** Our aim was to determine the incidence and severity of hemodynamic complications during therapeutic hypothermia and analyze whether these complications can be predicted from data available on admission.

**Methods:** This is a substudy of the TTH-48 study, where cardiac arrest (CA) patients were randomized to receive therapeutic hypothermia treatment for either 24 or 48 h [1]. Hypotension within four days from admission was recorded and defined as mild, moderate, severe or circulatory failure. Arrhythmias were recorded and classified as mild, moderate or severe. We calculated the incidence and distribution of severity of the events. We used multivariate logistic regression analysis to test association of admission data with any hypotension or any arrhythmia.

**Results:** Of all patients, 55.1% had hypotension which was mild in 58.2%, moderate in 27.3%, severe in 7.7% cases. 6.7% had circulatory failure. An arrhythmia was present in 44% of patients. Of these, 45.1% were mild, 29% moderate and 25.8 % severe. Bradycardia (N=3), new CA (N=1) and circulatory shock (N=1) were hemodynamic reasons for preterm rewarming. In multivariate analysis age (p=0.005, OR 1.033) and admission MAP (p=0.005, OR 1.020) were significantly associated with hypotensive complications. Only use of mechanical compressions was significantly associated with risk for arrhythmia (p=0.007, OR 0.380).

**Conclusions:** Hypotension and arrhythmias were frequent in cardiac arrest patients during days 1-4 from admission, but mostly mild or moderate in severity. Age and admission MAP were associated with hypotension. Only the use of mechanical compressions was independently (negatively) associated with arrhythmias.

**References**

1. Kirkegaard H et al. JAMA 318:341-50, 2017

## P417 Coronary angiographic findings after cardiac arrest in relation to ECG and comorbidity

### R Lagedal, L Elfwén, M Jonsson, E Lindgren, D Smekal, L Svensson, S James, P Nordberg, S Rubertsson

#### Institution for surgical sciences, anaesthesia and intensive care, Uppsala, Sweden

**Introduction:** The aim of this study was to describe the coronary angiographic findings in relation to specific ECG changes and comorbidity in survivors after cardiac arrest.

**Methods:** A retrospective cohort study of out-of-hospital cardiac arrest patients with data retrieved between 2008-2013 from national registries in Sweden. Unconscious patients with coronary angiography performed within 28 days after return of spontaneous circulation and available ECG were included (Fig. 1).

**Results:** After exclusion, 1133 patients were analyzed (Fig. 1), (Table 1). 249 (22%) were women and mean age were 64 years. Patients without ST-elevation were separated into groups with specified ECG changes and comorbidities. Differences were observed in the incidence of any significant stenosis, total occlusion and PCI performed, between the specified ECG changes, as well as between the comorbidity groups (Table 2). 27% of patients with ST-depression or pathologic T-waves had a complete vessel occlusion compared with only 16% in patients with normal ECG regardless of comorbidity (p=0.02). Patients with previous ischemic cardiac disease and/or diabetes had 34% incidence of total occlusion compared with only 20% in patients without these comorbidities, regardless of ECG pattern (ST elevation and left bundle branch block excluded) (p<0.01). Furthermore, no differences in incidence of total occlusion, PCI-attempts or significant stenosis between non-ST elevation pattern and left bundle branch block were found.

**Conclusions:** Our study suggests, that a specific evaluation of ECG and comorbidities can identify patients after cardiac arrest at high risk of coronary artery lesions that may benefit from immediate PCI.

**Table 1 (abstract P417).** Description comorbidity in relation to ECG changes after cardiac arrest

ST-depression (157)	23 (14,6)	4 (2,5)	6 (3,8)	14 (8,8)
Pathologic T-wave (38)	8 (21,1)	1 (2,6)	3 (7,9)	6 (15,8)
RBBB5 (59)	10 (16,9)	1 (1,7)	3 (5,1)	9 (15,3)
LBBB6 (108)	20 (18,5)	3 (2,0)	3 (2,8)	14 (13,0)
Other (155)	22 (14,2)	4 (2,6)	7 (4,5)	19 (12,3)

1.Ischemic heart disease. 2.Kidney disease, acute or chronic. 3.Chronic obstructive pulmonary disease. 4.Diabetes mellitus. 5.Right bundle branch block. 6.Left bundle branch block

**Table 2 (abstract P417).** Coronary angiographic findings in relation to ECG changes after cardiac arrest

ECG (n=1133)	Main stem^1^ n(%)	1 vessel^1^ n(%)	2-3 vessel^1^ n(%)	Total occlusion n(%)
Normal	13 (8.5)	35 (22.9)	44 (27.5)	25 (16.3)
ST elevation	32 (6.8)	198 (42.8)	184 (39.8)	242 (52.3)
LBBB2	6 (5.5)	12 (11.1)	42 (38.9)	20 (18.5)
ST depression	16 (10.2)	38 (24.2)	65 (41.4)	40 (25.5)
Pathologic T wave	4 (10.5)	8 (21.1)	14 (36.8)	13 (34.2)
RBBB^3^	5 (8.5)	17 (28.8)	19 (32.2)	14 (23.7)
Other^4^	6 (3.8)	40 (25.8)	55 (35.5)	42 (27.1)

1.Significant stenoses diagnosed during angiography in main stem or 1-3 of the coronary artery regions. Patients with stenoses in both main stem and other coronary artery regions are classified as main stem stenoses. 2.Left bundle branch block. 3.Right bundle branch block. 4.Other ECG changes, e.g.pacemakerrhytms

**Fig. 1 (abstract P417).**
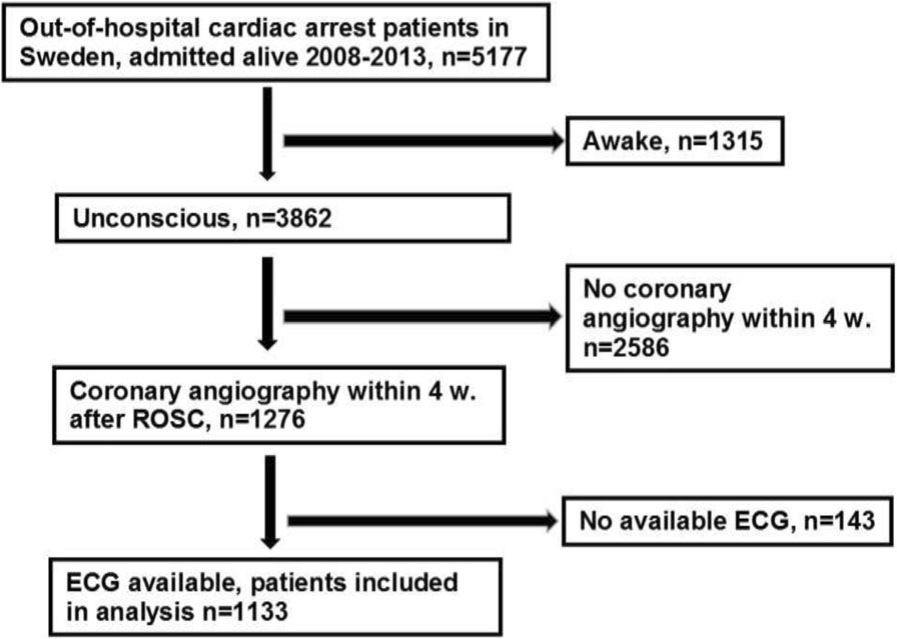
Participant flow

## P418 Gender differences in coronary angiography findings after out-of-hospital cardiac arrest

### E Lindgren^1^, R Lagedal^1^, L Elfwén^2^, M Jonsson^2^, D Smekal^1^, L Svensson^2^, S James^3^, P Nordberg^2^, S Rubertsson^1^

#### ^1^Anaesthesiology and Intensive Care Medicine, Uppsala, Sweden; ^2^Karolinska Institute, Stockholm, Sweden,^3^Uppsala University, Uppsala, Sweden

**Introduction:** Fewer women after return of spontaneous circulation from Out-of-Hospital Cardiac Arrest (OHCA) are undergoing coronary angiography (CAG) with possible Percutaneous Coronary Intervention (PCI). The aim was to investigate gender differences in comorbidity, CAG findings and outcome after OHCA in comatose patients with a shockable first ECG rhythm.

**Methods:** A retrospective cohort study of out-of-hospital cardiac arrest patients with data retrieved between 2008-2013 from national registries in Sweden (Fig. 1).

**Results:** There was no difference in age or comorbidity except for men having more ischemic heart disease, 21.5 vs 15.0% (p=0.006). Rates of previous myocardial infarction did not differ, 8.2 vs 6.3%. No difference was seen in rates of ECG indicating prompt CAG according to guidelines. Still, more men underwent CAG but no difference in numbers of CAG leading to PCI was seen (Table 1). Furthermore, in patients with ST elevation or LBBB, no gender difference in CAG and subsequent PCI was found. Men had lower rates of normal CAG findings but more triple vessel and left main coronary artery disease (Table 2). 1 year survival did not differ, 49.1 vs 45.0%.

**Conclusions:** Our study suggests, that despite no gender differences in rate of ECG findings indicating a prompt CAG, men seems to have a more severe coronary artery disease while women have more frequently normal angiograms. However, this did not influence 1 year survival.

**Table 1 (abstract P418).** Background characteristics and treatment

	Men N=1267 (76.9)	Women N=380 (23.1)	p value
Age, mean, range	66.5 (18-96)	66.6 (18-96)	0.867
ST-elev/LBBB*	466 (39.8)	127 (38.8)	0.754
CAG	543 (42.9)	134 (35.3)	0.009
-with PCI	373 (68.7)	85 (63.4)	0.244
CAG when ST-elev/LBBB	320 (68.7)	82 (64.6)	0.380
-with PCI	241 (75.3)	62 (75.6)	0.956
1-year surv.	622 (49.1)	171 (45.0)	0.577

Data is presented as No. (%) unless otherwise indicated. *96 men and 53 women had missing data

**Table 2 (abstract P418).** Early Angiography findings, patients with data

p=0.008	Men N=530	Women N=133
Normal	88 (16.6)	33 (24.8)
Occlusions, No of vessels:		
1	188 (35.5)	51 (38.3)
2	109 (20.6)	29 (21.8)
3	104 (19.6)	13 (9.8)
LMCA stenosis	41 (7.7)	6 (4.5)
Non conclusive	0	1 (0.8)

**Fig. 1 (abstract P418).**
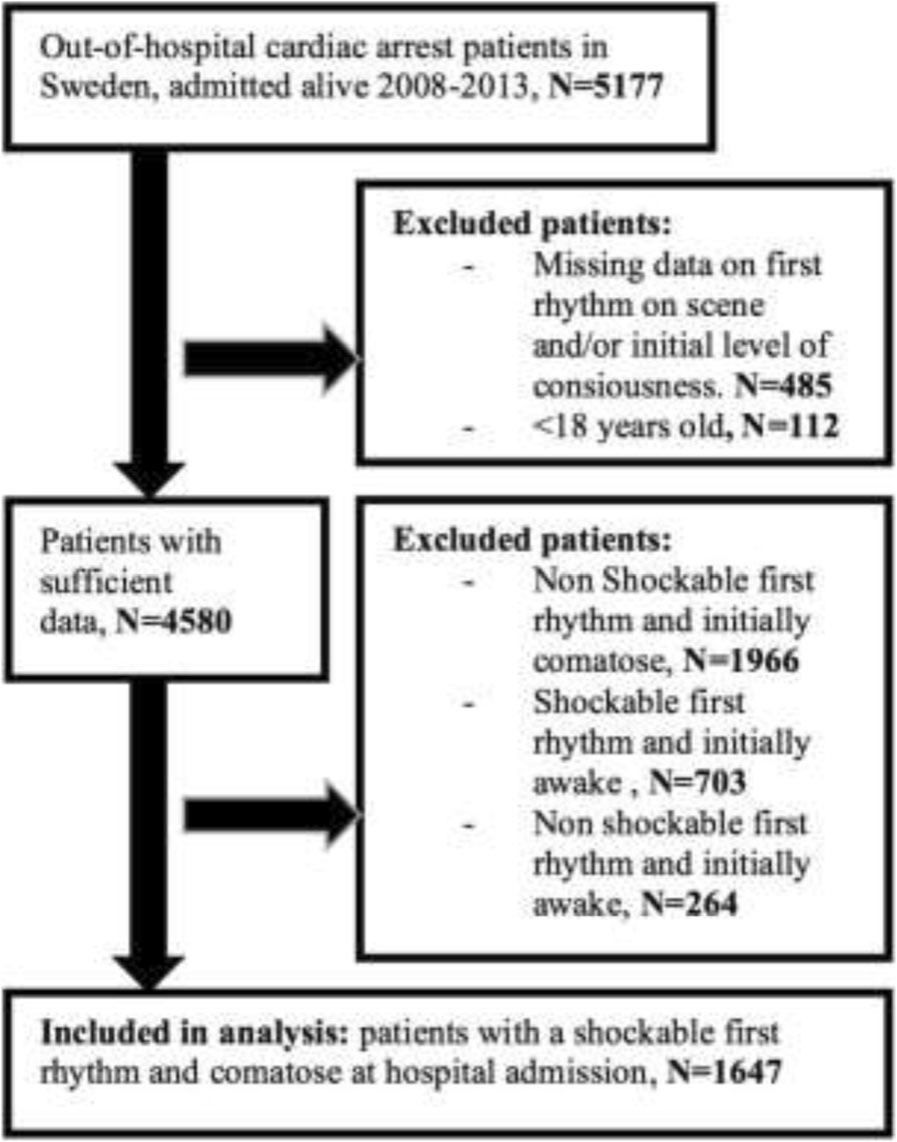
Participant flow

## P419 Circadian variation of outcomes in out-of-hospital cardiac arrest of cardiac etiology

### K Maekawa, M Hayakawa, T Yoshida, Y Itagaki, T Tsuchida, A Tomita, Y Honma, A Mizugaki, H Murakami, T Oyasu, T Saito, K Katabami, T Wada, H Sageshima, A Sawamura

#### Hokkaido University Hospital, Sapporo, Japan

**Introduction:** The circadian clock influences a number of cardiovascular physiological processes. A time-of-day variation in infarct size has recently been shown in patients with ST segment elevation myocardial infarction. However, there is no clinical evidence of circadian variation in patients with out-of-hospital cardiac arrest (OHCA) of cardiac etiology.

**Methods:** We performed retrospective analysis using data from Japan’s nationwide OHCA registry from January 2005 through December 2012, which includes all OHCA patients presented with ventricular fibrillation as first documented rhythm, and consequently confirmed cardiac etiology. In order to eliminate the night and weekend effects, we enrolled only patients suffered OHCA in the morning (6:00-11:59) or afternoon (12:00-17:59) on weekdays. We determined the impact of time-of-day onset of OHCA on clinical outcomes including return of spontaneous circulation (ROSC), survival and favorable functional status at 1 month after cardiac arrest.

**Results:** Of 13474 eligible patients, 7199 suffered OHCA in the morning and 6275 in the afternoon. The rate of ROSC was higher in the afternoon group than in the morning group (37.2% vs. 35.0%, adjusted odds ratio 1.08, 95%CI 1.01-1.17, p=0.036), however, the rate of survival and favorable functional status at 1 month were not significantly different among both groups (36.7% vs. 36.1%, 25.5% vs. 26.2%, respectively). In the propensity-matched cohort consisted of 6103 patients each from both groups, the rate of ROSC was higher in the afternoon group (37.1% vs. 35.3%, adjusted odds ratio 1.10, 95%CI 1.01-1.18, p=0.023) and there were no significant differences in the rate of survival and favorable functional status at 1 month (36.6% vs. 36.6%, 25.5% vs. 26.6%, respectively).

**Conclusions:** We found an independent correlation between the time of the day at which OHCA occurred and ROSC, however, this did not impact long-term outcomes.

## P420 An alarm bell ignored: impact of availability of intensive care unit on outcome of out-of-hospital cardiac arrest

### C Chen^1^, Y Yeh^2^, S Change^2^, P Lee^2^

#### ^1^E-DA Hospital, I-Shou University, Kaohsiung, Taiwan; ^2^Kaohsiung Medical University Hospital/Kaohsiung Medical University, Kaohsiung, Taiwan

**Introduction:** In the fast-paced pre-hospital settings, it’s difficult to estimate accurate bed availability of intensive care unit (ICU) of the receiving hospitals while approaching out-of-hospital cardiac arrest (OHCA) patients. It might be troublesome if a OHCA patient regaining signs of life is sent to a hospital without ICU bed. Previous work has not elucidate the problem. We aim to evaluate the influence of ICU bed capacity on outcome of OHCA patients.

**Methods:** We conduct a retrospective cohort study focusing on the association between OHCA outcome and ICU bed availability. The OHCA data was acquired from a regional emergency operation center, and the ICU bed information was obtained from a regional surveillance system among 22 hospitals. Demographic data of OHCA patients, timestamp of OHCA event, and features of the receiving hospitals were collected. Primary outcome was survival to discharge. Secondary outcome was favorable neurological outcome defined as cerebral performance category (CPC) 1 or 2.

**Results:** In a 6-year period, 5141 OHCA (12.2%, traumatic) patients were sent to the hospitals with available records of ICU bed availability. Overall survival (discharged alive) was 7.8%, and 1% achieved favorable neurological outcome. On hospital arrival, the survival rate of OHCA patient treated in the hospitals with available ICU capacity was 8.6%. 17 % of OHCA patients were sent to the hospital without ICU bed, and demonstrated a survival rate of 3.5% with a favorable neurological outcome of 1.2%. In the multivariate analysis, the first recorded cardiac rhythm, location of OHCA, and availability of ICU bed appeared to be significant risk factors of survival to discharge, but not observed in neurological outcome.

**Conclusions:** In addition to cardiac rhythm and location of OHCA, we disclosed the major indicator of ICU capacity for an increased chance of survival. With regard to achieving better precision public health, we recommend ICU bed availability as an adjuvant dispatch tool for OHCA management.

## P421 Noninvasive monitoring during transport of OHCA. a step further towards early precision medicine for ROSC patients?

### S Dziura, C Castroviejo, D De Longueville, M Bartiaux, S Malinverni

#### CHU Saint Pierre, Brussels, Belgium

**Introduction:** Oxygen delivery has a fundamental role in out-of-hospital cardiac arrest (OHCA) management and achieving recommended SatO2 levels in the prehospital phase is elusive. Often times, it exceeds physiological levels in order to avoid insufficient oxygenation [1]. Hyperoxia has been associated with increased in-hospital mortality, though uncertainty remains about this association. Multiwave pulse co-oximetry has safely been studied intraoperatively as a guide to monitor hyper- and hypoxia by calculating an oxygen reserve index (ORI) which could add information to pulse oximetry measures when SpO2 is >98% [2].

**Methods:** This is a monocentric prospective study including 12 patients with successful resuscitation following OHCA. The aim of our study is to evaluate the feasibility and assess the availability of novel non invasive oxygen and hemodynamic variables. Collected data principally concern blood oxygen and circulation such as ORI, SpO2, total Hb, perfusion index and pulse rates. Recording is ideally started at time of ROSC.

**Results:** We monitored 12 consecutive patients for a total time of 456.8 min during transport from OHCA place to the ER. SpO2 signal was present for 82.3% of transport time.Oxygen Reserve Index signal was present for 58.5% of the total transport time. Pleth variability index (PVI) signal was present 59.8% of the total transport time. SpHb signal was present 44.7% of total time from ROSC to hospital. The confidence interval for each variable is given in Fig. 1.

**Conclusions:** Our pilot study shows that noninvasive measurements of hyperoxia, fluid responsiveness and hemoglobin are readily available from the prehospital phase of post-ROSC care allowing for early tailored and goal directed interventions.

**References**

1 Young P et al. Resuscitation 85(12):1686-1691, 2014

2. Applegate R et al. Anesth Analg 123(3):626-633, 2016

**Fig. 1 (abstract P421).**
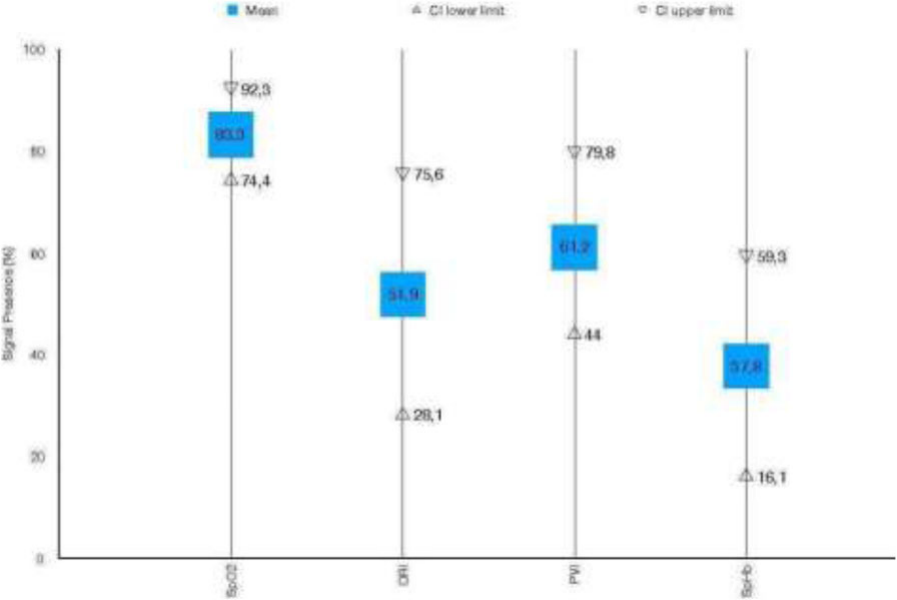
Mean ± SD time percentage of signal capture from ROSC to hospital admission during total time for each variable

## P422 Implementation of a rescue and organ perfusion protocol after ohca: clinical experience and perspective

### A Franci, G Di Tommaso, G Cianchi, ML Migliaccio, C Guetti, M Ciapetti, S Di Valvasone, M Bonizzoli, A Peris

#### Azienda Ospedaliero Universitaria Careggi, Firenze, Italy

**Introduction:** We analysed the effect of a new protocol for out-of-hospital cardiac arrest (OHCA) shared by the territorial emergency medical service and the in-hospital emergency team. We examined the logistical and operative changes prompted by the introduction on the ambulances (January 2016) of a Mechanical Chest Compression (MCC) device and by the start (June 2016) of a protocol of Non-Heart-Beating Donation (NHBD)

**Methods:** We enrolled 105 patients affected by OHCA brought to the Emergency Room from June 2016 to August 2017. Patients were enrolled regardless of their age, comorbidities, aetiology and first rhythm documented. We compared Period 1 (Jun-Dec 2016) in which the system was just started, with Period 2 (Jan-Aug 2017) where the system would had reached a good level of reliability. We compared hospital survival and neurological outcome through Cerebral Performance Categories (CPC). We also evaluated the use of a MCC device, the necessity of an Extracorporeal Life Support (ECLS), the rate of coronary angiography (PCA) and organ procurement with special attention to the NHBD

**Results:** OHCA increased from 39 in Period 1 to 66 in Period 2 (Table 1). The use of MCC increased by 8%. ECLS treatment in the Emergency Room increased by 8%, while PCA significantly raised of 20%. Both survival and neurological outcome showed no differences between groups. We detected a 4% increase in NHBD without any statistical significance

**Conclusions:** This new protocol has allowed a larger number of OHCA to reach the Emergency Room and it has made possible for 16 patients to start an ECLS. In 13 patients NHBD was able to satisfy patient’s wish of organ donation

**Table 1 (abstract P422).** Results P1 vs P2

	P1 (%)	P2 (%)
OHCA	39	66
MCC	20 (51)	39 (59)
ECLS	4 (10)	12 (18)
PCA	5 (13)	22 (33)* p 0.02
NHBD	4 (10)	9 (14)
Survival	10 (26)	14 (21)
CPC 1-2	6 (15)	11 (17)

## P423 Association of SOFA score with 12-month survival and healthcare costs after cardiac arrest.

### PT Pekkarinen^1^, I Efendijev^1^, R Raj^1^, D Folger^1^, E Litonius^1^, R Laitio^2^, S Bendel^3^, S Hoppu^4^, T Ala-Kokko^5^, M Reinikainen^6^, MB Skrifvars^1^

#### ^1^Helsinki University Hospital, Helsinki, Finland; ^2^Turku University Hospital, Turku, Finland; ^3^Kuopio University Hospital, Kuopio, Finland; ^4^Tampere University Hospital, Tampere, Finland; ^5^University of Oulu and Oulu University Hospital, Oulu, Finland; ^6^North Karelia Central Hospital, Joensuu, Finland

**Introduction:** We used nationwide registry data from the intensive care units (ICU) of the five Finnish university hospitals to evaluate the association of 24-hour sequential organ failure assessment (SOFA) score with 12-month survival and healthcare costs after cardiac arrest (CA).

**Methods:** We included adult CA patients treated in the ICUs between January 1st, 2003 and December 31st, 2013. We acquired the confirmed date of death from the Finnish Population Register Centre database and gross 12-month healthcare costs from the hospital billing records and the database of the Finnish Social Insurance Institution.

**Results:** A total of 5814 patients were included in the study and 2401 were alive at 12 months. Median (interquartile range, IQR) 24-hour SOFA score was 8 (6-10) in 12-month survivors and 10 (8-13) in non-survivors. The SOFA score had an area under receiver operating characteristic curve of 0.68 (95% CI 0.66-0.69) for predicting 12-month mortality. In multivariate regression model with age and gender, SOFA score had an odds ratio, OR (95% confidence interval, CI) of 1.21 (1.19-1.23) for predicting 12-month mortality. All except cardiovascular sub-score also had independent predictive value.

Median (IQR) healthcare costs in 12 months after CA were 47 000€ (28 000-75 000€) in 12-month survivors and 12 000€ (6 600-25 000€) in non-survivors. In univariate linear regression model one point increase in SOFA score was associated with 170€ (95% CI 150-180€) increase in the cost per day alive in the first 12 months after CA.

**Conclusions:** The SOFA score is a good indicator of disease severity but the overlap between outcome groups does not allow its use for early prognostication in CA patients. The association of SOFA and its sub-scores with 12-month outcome and healthcare costs highlights that in addition to neurologic damage the full spectrum of multiple organ failure affects the survival and morbidity of CA patients.

## P424 Public opinion on cardiopulmonary resuscitation decision and outcome in out-of-hospital cardiac arrest patients – questionnaire study

### TY Li^1^, HP Shum^2^, W Wong^2^, SF Wong^2^, GCK Wong^1^, KC Chan^3^, WW Yan^2^

#### ^1^Queen Elizabeth Hospital, Hong Kong, Hong Kong; ^2^Pamela Youde Nethersole Eastern Hospital, Hong Kong, Hong Kong; ^3^Tuen Mun Hospital, Hong Kong, Hong Kong

**Introduction:** Survival after out-of-hospital cardiac arrest (OHCA) varies widely between communities. The survival-to-hospital discharge rate ranges from 0% to 31.2%, in contrary to a mean estimate of survival rate of 54% from the general public. Studies showed that the knowledge of CPR was poor among the general public and their expectation on CPR outcome may not be realistic. Related information among local population is limited and therefore, this study was undertaken to determine the accuracy of knowledge in local population in Hong Kong, and establish opinions of the general public regarding CPR on OHCA patients.

**Methods:** This was a cross-sectional questionnaire study administered in community settings to the volunteer respondents over January to December 2016. Descriptive statistics including public opinion on survival-to-discharge rate and duration of resuscitation were reported. Logistic regression and factor analysis were performed to identify factors that public opined a physician should consider to decide continuation of resuscitation.

**Results:** Among 416 respondents, the mean estimate of predicted survival-to-discharge rate after cardiac arrest was 48.4% (median 50%; IQR 20-70%). Mean estimated duration of resuscitation to withdraw from CPR was 43 minutes (median 30 min; IQR 20-60 min). Physicians’ opinion and prediction of outcome, premorbid state and age of patient were the three factors that the survey respondents considered most important for decision making on resuscitation.

**Conclusions:** The public had inaccurate perceptions regarding resuscitation time, procedures, interventions and survival rate. Unrealistic high survival rate was expected on the basis of limited acceptance of different resuscitation interventions. Better public education was necessary.

## P425 Metabolomic profile in ventricular fibrillation arrest and return of spontaneous circulation

### E Locci^1^, G Karlis^2^, A Gulati^3^, A Noto^1^, F Rosa^1^, M Mura^1^, G Finco^1^, E D’Aloja^1^, P Scano^1^, T Xanthos^4^

#### ^1^University of Cagliari, Cagliari, Italy; ^2^General Hospital of Rodos, Rodos, Greece; ^3^University of Chicago, Chicago, USA; ^4^European University Cyprus, Nicosia, Cyprus

**Introduction:** Metabolomics is a novel approach that can characterize small molecules (metabolites) and has the potential to explore genotype-phenotype and genotype-environment interactions, delivering an accurate snapshot of the subject’s metabolic status. In this context, the aim of metabolomics is to improve early diagnosis, classification, and prediction over the development of a pathological condition. To this end, metabolomics have not been used in the characterisation of cardiac arrest (CA), cardiopulmonary resuscitation (CPR) and return of spontaneous resuscitation (ROSC). The aim of the present study was to explore whether metabolomics can characterize the CA versus ROSC in a swine model of ventricular fibrillation (VF).

**Methods:** Ten animals were intubated and instrumented and VF was induced with the use of a cadmium battery. VF was left untreated for 6min and the animals were then resuscitated according to the 2010 guidelines. Defibrillation was attempted in all animals. Venous blood was drown at baseline, 2 min, 4 min, 6 min during untreated CA and finally at 2min, 30min, 2h, 6h after ROSC in order to determine the metabolomic profile during CA and during the early post-resuscitation period. ROSC was defined as the presence of an organized cardiac rhythm with a mean arterial pressure of at least 50 mmHg for >5 min. Blood was centrifuged and serum was analysed by high resolution 1H-NMR spectroscopy. NMR spectral data were submitted to multivariate discriminant analysis.

**Results:** Eight animals survived the experiment and were included in the analysis. Metabolites upregulated in the immediate ROSC versus CA were succinate, hypoxanthine, choline and lactate. Metabolites upregulated in the 2 hour ROSC versus CA were ornithine and alanine. The 3 measured phases are shown in Fig. 1.

**Conclusions:** It appears that different metabolic pathways are being activated in the ROSC period. Further studies are needed to accurately define the metabolic pathways.

**Fig. 1 (abstract P425).**
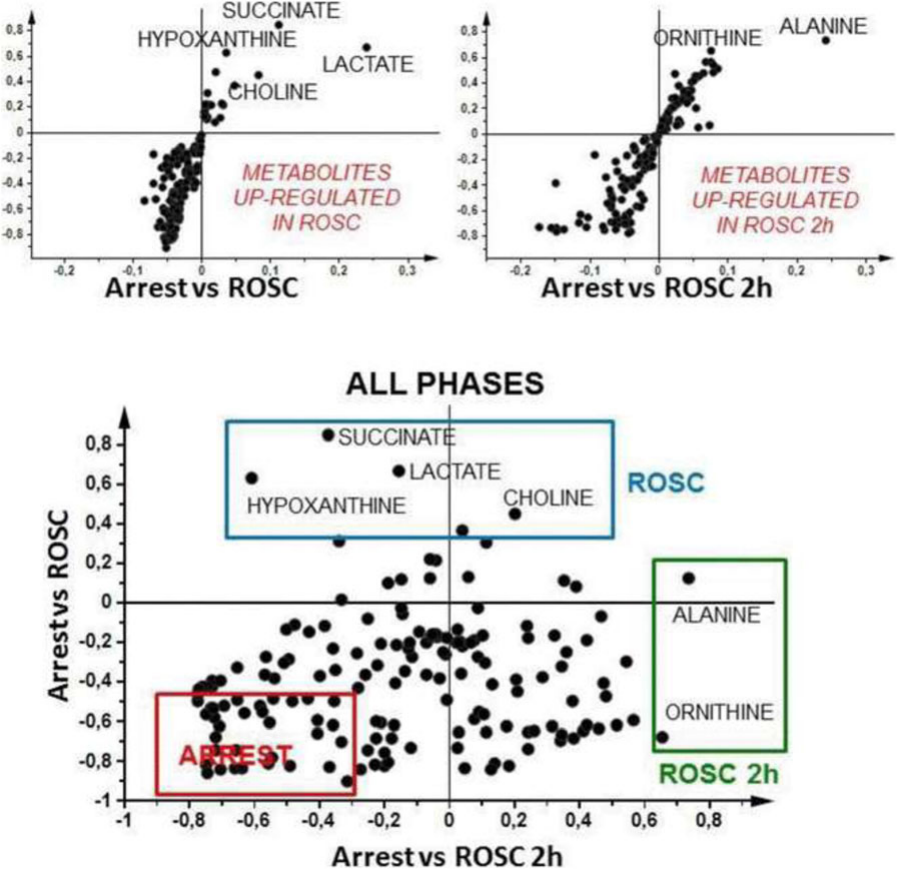
Characterizing metabolites in return of spontaneous circulation (ROSC) versus cardiac arrest (CA).

## P426 30-day survival and prediction of coronary disease after out-of-hospital cardiac arrest

### J Higny^1^, C De Meester de Ravenstein^2^, E Schroeder^1^, A Guédès^1^, L Gabriel^1^, C Hanet^1^

#### ^1^CHU UCL Namur, Yvoir, Belgium; ^2^Cliniques universitaires Saint-Luc, Brussels, Belgium

**Introduction:** Key predictors of survival after OHCA have been described in the literature. Current guidelines recommend emergency angiography in patients without an obvious extra-cardiac cause of arrest [1]. However, the value of this strategy is debated. Moreover, diagnosis of acute coronary ischaemia after OHCA remains challenging, especially in patients without ST-segment elevation.

**Methods:** Retrospective single-centre study including a consecutive series of 123 patients resuscitated from OHCA. Baseline characteristics and resuscitation settings were analysed. Our aim was to identify qualitative variables associated with an increased chance of 30-day survival after OHCA. Afterwards, we sought to identify parameters associated with a positive coronary angiography in patients with a cardiac aetiology and absence of ST-segment elevation.

**Results:** The independent predictors of the 30-day outcome after OHCA included haemodynamic instability (OR 5.39; 95% CI 1.60-18.13), witnessed cardiac arrest (OR 0.13; 95% CI 0.05-0.35) and coronary angiography (OR 0.15; 95% CI 0.05-0.43) (Table 1). Factors associated with a positive coronary angiogram in patients without ST-segment elevation included personal history of coronary artery disease (CAD) (P<0.001) and male gender (P=0.005). Diabetes and dyslipidaemia were the most related cardiovascular risk factors (P<0.05). Presence of at least two cardiovascular risk factors (CVRF) was statistically predictive of CAD in this population (P<0.001) (Table 2).

**Conclusions:** We investigated qualitative predictors of 30-day survival after OHCA. Our findings suggest that the identification of patients with a better prognosis might be improved. We also identified predictors for a positive angiography in patients without ST-segment elevation. Therefore, the identification of risk criteria may help to select the best candidates for emergency angiography.

**Reference**

1. Windecker S, Kolh P et al. Eur Heart J 35:2585, 2014

**Table 1 (abstract P426).** Univariate and multivariate analysis for the prediction of 30-day mortality after OHCA

Variables	Univariate analysis (OR 95% CI)	P-value	Multivariate analysis (OR 95% CI)	P-value
Male gender	0.53 (0.23-1.22)	0.14		
Age > 65 years	2.09 (1.00-4.36)	0.050		
Convertible rhythm	0.20 (0.09-0.45)	<0.001		
Witnessed CA	0.07 (0.03-0.17)	<0.001	0.13 (0.05-0.35)	<0.001
Shock	4.30 (1.83-10.10)	<0.001	5.39 (1.60-18.13)	<0.001
ACS	0.21 (0.09-0.45)	<0.001		
Coronary angiography	0.11 (0.05-0.26)	<0.001	0.15 (0.05-0.43)	<0.001

CA: cardiac arrest, ACS: acute coronary syndrome

**Table 2 (abstract P426).** Parameters associated with CAD in patients with cardiac aetiology and absence of ST-segment elevation

Variables	Patients with CAD	Patients without CAD	P-value
Male gender	34 (85)	14 (54)	0.005
Age > 65 years	28 (70)	12 (46)	0.054
Convertible rhythm	28 (70)	16 (61)	0.48
Personal history of CAD	26 (65)	2 (8)	<0.001
Diabetes	25 (63)	7 (27)	0.004
Dyslipidaemia	25 (63)	5 (19)	<0.001
≥ 2 CVRF	36 (90)	10 (38)	<0.001

CAD: coronary artery disease, CVRF: cardiovascular risk factor

## P427 A novel prognostic model for the prediction of good neurological outcome after cardiac arrest

### T Castermans, M Vander Laenen, M Vander Laenen, P Vanelderen, J Dens, F Jans, D Mesotten, C De Deyne, W Eertmans

#### Ziekenhuis Oost-Limburg, Genk, Belgium

**Introduction:** Early outcome prognostication in successfully resuscitated out-of-hospital cardiac arrest (OHCA) patients remains challenging. Prediction models supporting the early decision to continue with full supportive treatment could be of major interest following OHCA. We constructed prognostic models able to predict good neurologic outcome within 48 hours after ICU admission.

**Methods:** Upon ICU admission, targeted temperature management at 33°C, hemodynamic and neuromonitoring (cerebral oxygen saturation measured with near-infrared spectroscopy and bispectral index (BIS)) was initiated. Prediction models for good neurologic outcome at 180 days post-CA were constructed at hour 1, 12, 24 and 48 after admission using variables easily collectable and known to be predictive for outcome. After multiple imputation, variables were selected using the elastic-net method. Each imputed dataset was divided into training and validation sets (80% and 20% of patients, respectively). Cut-off probabilities yielding a sensitivity above 90% were determined and performance of all logistic regression models was assessed using misclassification rates.

**Results:** Overall, 107 successfully resuscitated OHCA patients were enrolled. The prediction model at hour 48 predicted good outcome with the lowest misclassification rate (26.2%) using a cut-off probability of 0.15 (sensitivity=94%; specificity=53%). This model contained sex, diabetes, initial rhythm, percutaneous coronary intervention, presence of BIS value of 0, lactate and neuron-specific enolase at hour 48 as predictive variables for good neurological outcome.

**Conclusions:** Based on a prognostic model containing variables readily available following ICU admission, good neurological outcome after OHCA was predicted best at hour 48. This model could target patients who could benefit the most from further treatment efforts.

## P428 Outcomes of patients receiving therapeutic hypothermia at 34 degree celsius for non-traumatic OHCA

### M Chia

#### Tan Tock Seng Hospital, Singapore, Singapore

**Introduction:** We aim to find out the outcomes of patients brought in by EMS who had ROSC following non-­traumatic OHCA and started on TH at 34°C.

**Methods:** This is retrospective case record review. Inclusion criteria were all patients who had non­-traumatic OHCA conveyed by emergency medical services to our Emergency Department, who had ROSC and started TH in ED for the period 1st Aug 2012 to 31st Aug 2014. Exclusion criteria were traumatic OHCA and all patients declared dead at scene.

Data collection followed Ustein template. EMS data were from National Cardiac Arrest registry. Data of patients admitted were from ED and inpatient electronic case records. Patient were followed up for 30 days.

All OHCA were conveyed by EMS to EDs for continued care. Paramedic are trained in advanced life support. All airway was managed with supraglottic airway device, placed on mechanical compression device and intravenous adrenaline were administered. Our targeted temperature for TH was 34°C and followed a 24hrs cooling protocol in the intensive care unit (ICU).

**Results:** There was 888 non­traumatic OHCA. 225 had ROSC in ED but only 23 received TH. Of 23 who received TH, 5 died in ED, 18 were admitted. 12 subsequently died in hospital while 4 were alive at 30 ­days. 2 had cerebral performance category (CPC) 4, 1 had CPC 3 and 1 had CPC 1. Among patients who received TH, 10 were witnessed arrest by family, 4 by EMS provider, 1 by healthcare provider and 8 were unwitnessed. 15 did not received bystander CPR. 2 had bystander AED applied. The initial rhythm by EMS were 13 asystole, 7 PEA, 2 VF and 1 unknown. 2 had field ROSC.

**Conclusions:** 10% of patients who had ROSC in ED had TH. Patients who received TH did not have good outcomes, although 65% had a witnessed arrest, most did not have VF as initial rhythm.

## P429 A strategy to effect neurologically-intact survival for children with out-of-hospital cardiac arrest

### PE Pepe ^1^, PR Banerjee^2^, RA Vittone^2^, A Singh^3^, L Ganti^3^

#### ^1^University of Texas Southwestern Medical Center, Dallas, USA; ^2^Polk County (Florida) Fire Rescue Department, Bartow, Florida, USA; ^3^University of Central Florida, Kissimmee, Florida, USA

**Introduction:** In many venues, EMS crews limit on-scene care for pediatric out-hospital cardiac arrest (POHCA), attempting treatment during transport. Hypothesizing that neuro-intact survival can be improved by prioritizing on-site care, strategies were effected to expedite on-scene drug delivery and intubation (with controlled ventilation).

**Methods:** From 1/1/2012 to 4/30/2017, data for POHCA cases were collected. In 2014, new training prioritized on-site resuscitation (Phase I) using expedited drug delivery and intubation with controlled ventilation (~6 breaths/min). Training included psychological and skills-enhancing tools to boost confidence in providing on-scene care. In 2016, drugs were prepared while responding (Phase II). 2010 American Heart Association guidelines were used throughout and no other modifications were made. Neuro-intact survival in 2012-13 was compared to Phase I & II outcomes.

**Results:** Over the 5.33-years, EMS faced 143 consecutive POHCA cases. The great majority presented in asystole throughout. In those resuscitated, mean time from on-scene arrival to the 1st epinephrine infusion fell from 16.5 min (2012-13) to 7.3 min (Phase I) and 5.0 min (Phase II). By 2017, it was 2 min. for resuscitated patients and 3.33 min. for all patients. Intubation and intraosseous insertion occurred more frequently in Phase I/II, but there were no significant differences in age, sex, etiology, response times, bystander CPR or drug sequencing. Neuro-intact survival improved significantly from 0/38 in 2012-13 to 23.2% (13/56) in Phase I and 34.7% (17/49) in Phase II (p < 0.0001; 2-tailed Fisher’s exact test) (Fig. 1).

**Conclusions:** Although historically-controlled, the sudden appearance of neuro-intact survivors following a renewed focus on rapid on-site care was profound, immediate and sustained. Beyond skills-enhancing strategies, physiologically-driven techniques and supportive encouragement from leadership, pre-arrival psychological and clinical tools were also likely contributors to the observed outcomes.

**Fig. 1 (abstract P429).**
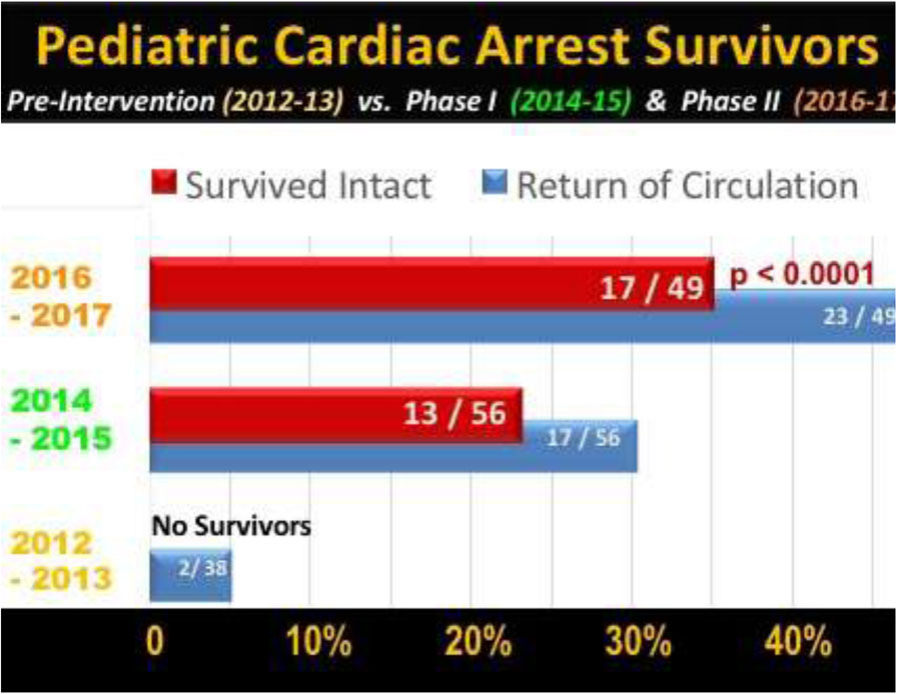
Effecting neurologically-intact survival for children with out-of-hospital cardiac arrest

## P430 Improved outcomes with a bundled resuscitation technique to enhance venous return out of the brain and into the heart during cardiopulmonary resuscitation

### PE Pepe ^1^, KA Scheppke^2^, PM Antevy^2^, D Millstone^2^, C Coyle ^2^, C Prusansky^2^, S Garay^2^, JC Moore^3^

#### ^1^University of Texas Southwestern Medical Center, Dallas, USA; ^2^Palm Beach County, Florida, USA; ^3^University of Minnesota and Hennepin County Medical Center, Minneapolis, Minnesota, USA

**Introduction:** Lowering intracranial pressure to improve brain perfusion during CPR has become a focus for our team. Combined with devices that enhance venous return out of the brain and into the thorax during CPR, outcomes have improved using head/chest elevation in the laboratory (Fig. 1). This study’s purpose was to confirm the safety/clinical feasibility of this new approach involving mechanical CPR at an angle.

**Methods:** 2,285 consecutive out-of-hospital cardiac arrest (OOHCA) cases (all rhythms) were studied for 3.5 years (1/1/14 to 30/6/17) in an expansive, socio-economically-diverse U.S. county (pop. 1.4 mill). In 2014, EMS crews used the Lucas© and impedance threshold (ITD) devices on such patients, but, after April 2015, they also: 1) applied O2 and deferred +-pressure ventilation several min; 2) raised the backboard ~20°; and 3) solidified a pit-crew approach to expedite Lucas© placement. Neuro-intact survival was not recorded until 2015, so resuscitation by EMS to hospital admission was used for consistency. Quarterly reports were run to identify any periodic variations or incremental effects during protocol transition (Quarter 2, 2015).

**Results:** No problems were observed with head/torso-up positioning (n=1,319), but rates of resuscitation rose steadily during the transition period with an ensuing sustained doubling (Fig. 2) over the ensuing 2 years when compared to those studied (n=806) prior to the head-up approach (mean 35.2%; range 30-40% vs. 17.9%, range 15-20%; p < 0.0001). Outcomes improved across subgroups. Response intervals, indications for attempting CPR and bystander CPR rates were unchanged. Resuscitation rates in 2015-17 remained proportional to neuro-intact survival.

**Conclusions:** The head/torso-up CPR bundle was not only feasible, but also associated with an immediate, steady rise in resuscitation rates during the transition phase with a sustained doubling of resuscitation rates, making a compelling case that this bundled technique may improve OOHCA outcomes in future clinical trials.

**Fig. 1 (abstract P430).**
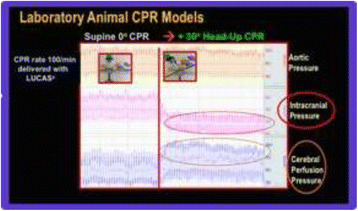
Laboratory studies leading to concept of improved cerebral perfusion pressures with head-up CPR

**Fig. 2 (abstract P430).**
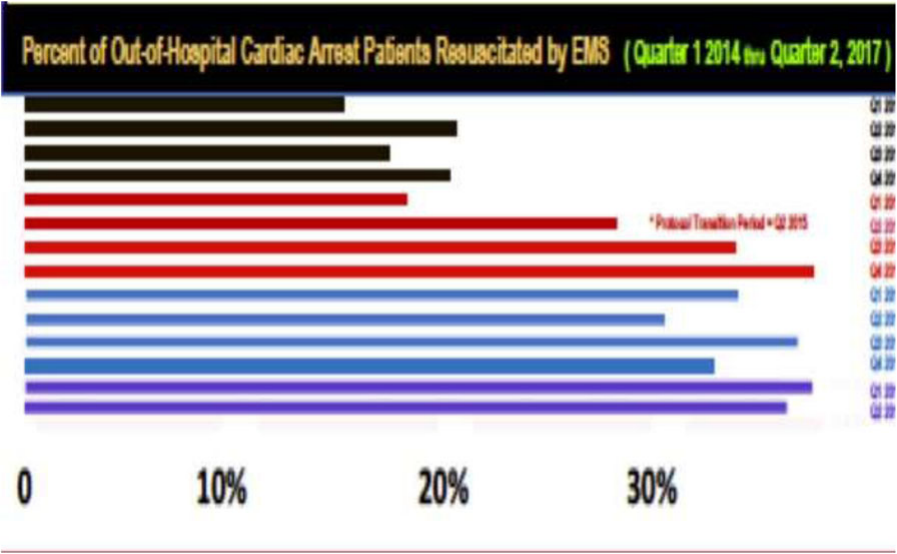
Immediate and Sustained Doubling of Resuscitation by EMS after Introducing the Novel Approach to CPR

## P431 Total one-year healthcare costs of cardiac arrest patients treated in the intensive care unit

### I Efendijev^1^, D Folger^1^, R Raj^1^, M Reinikainen^2^, PT Pekkarinen^1^, E Litonius^1^, MB Skrifvars^1^

#### ^1^University of Helsinki and Helsinki University Hospital, Helsinki, Finland; ^2^North Karelia Central Hospital, Joensuu, Finland

**Introduction:** Cardiac arrest (CA) often requires intensive care unit (ICU) treatment, which is costly. While there are plenty of data regarding post-CA outcomes, knowledge of cardiac arrest associated healthcare costs is virtually non-existent.

**Methods:** We performed a single-center registry-based study to determine expenditure data for ICU-treated CA patients between 2005 and 2013. Healthcare cost evaluation included costs from the initial hospital treatment, rehabilitation costs and social security costs up to one year post-CA. We calculated mean healthcare costs for one year survivors and for hospital survivors who died within the first year after cardiac arrest. We calculated effective costs per independent survivor (ECPIS) as an indicator of cost-effectiveness. We adjusted all costs according to consumer price index (CPI) in Finland as of 2013. All costs are presented as 2013 euros (€).

**Results:** We identified 1,314 CA patients eligible for the analyses. At one year after CA 52% of the patients were alive and 40% were alive and independent in daily activities. One year survival stratified by cardiac arrest location group was 59% for out-of-hospital CA patients, 47% for in-hospital CA patients and 27% for in-ICU CA patients. For the whole study population, mean healthcare costs were 50,211€ per patient. Healthcare costs for hospital survivors were 67,928€ per patient and for hospital non-survivors 22,100€ per patient. Healthcare costs for those who survived to hospital discharge but died within the first year were 56,490€ per patient, while for one year survivors they were 70,148€ per patient. Healthcare costs stratified by CA location are presented in Fig. 1. Mean ECPIS were 65,684€.

**Conclusions:** For ICU-treated cardiac arrest patients, the mean ECPIS were close to 65,000€. The best prognosis and the lowest costs were observed for out-of-hospital CA patients.

**Fig. 1 (abstract P431).**
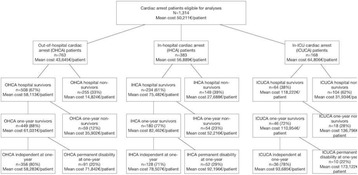
Healthcare costs stratified by cardiac arrest location

## P432 Epidemiology and outcomes from out-of-hospital cardiac arrests in Kaunas (Lithuania) in 2016

### A Krikscionaitien^1^, P Dobozinskas^1^, L Darginavicius^1^, N Jasinskas^1^, N Balciuniene^1^, J Vencloviene^2^, E Vaitkaitiene^1^, D Vaitkaitis^1^

#### ^1^Lithuanian University of Health Sciences, Kaunas, Lithuania; ^2^Vytautas Magnus University, Kaunas, Lithuania

**Introduction:** In Lithuania the incidence of out-of-hospital cardiac arrest (OHCA) is unknown, as there is no official coding for OHCA as a cause of death in the national death registry. Kaunas Emergency Medical Service (EMS) underwent major stepwise changes since 2011, including implementation of Medical Priority Dispatch System and dispatcher-assisted CPR instructions. We sought to describe the epidemiology and outcomes from OHCA in Kaunas, the second largest Lithuanian city.

**Methods:** The incidence, demographics and outcomes of patients who were treated for an OHCA between 1st January 2016 and 31st December 2016 in Kaunas EMS, serving a population of almost 0.3 million, were collected and are reported in accordance with 2014 Utstein recommendations.

**Results:** In total, 313 OHCA cases of EMS treated cardiac arrests were reported (105 per 100.000 of resident population). The mean age was 67.7 (SD=15.7) years and 64.9% were male. 70% OHCA cases occurred at home and 52.7% were witnessed by either EMS or a bystander. In non-EMS witnessed cases, 43.8% received bystander CPR, whilst public access defibrillation was not used. Medical dispatcher identified OHCA in 71.3% of all cases and provided over-the-phone CPR instructions in 60.2% of them. Average EMS response time (90% fractile) was 13 min. Cardiac aetiology was the leading cause of cardiac arrest (84.3%). The initial rhythm was shockable (VF or pVT) in 26% and non-shockable (asystole or EMD) in 70.5% of all cases. Return of spontaneous circulation (ROSC) at hospital transfer was evident in 24.9% and survival to hospital discharge was 8.6%.

**Conclusions:** ROSC and survival to hospital discharge in Kaunas were similar to those reported in United Kingdom in 2014 [1]. Routine OHCA data collection and analysis will allow us to track the efficiency of service improvements and should become a standard practice in all Lithuanian regions.

**References**

1. Hawkes C, et al. Resuscitation 110:133-140, 2017

## P433 Outcomes of patients admitted to intensive care following cardiac arrest

### J McLoughlin, E Landymore, P Morgan

#### East Surrey Hospital, Surrey, UK

**Introduction:** Patients who have return of spontaneous circulation following a cardiac arrest are haemodynamically unstable and require critical care input. Outcomes are often poor, with unadjusted survival to hospital discharge at 18.4%, following an in hospital cardiac arrest [1]. The aim of the study was to assess the survival of patients admitted to intensive care following a cardiac arrest, reviewing whether age and gender impacted on their outcome.

**Methods:** The INARC database for a general intensive care unit (ICU) at a district general hospital was reviewed. Since 1993, 519 patients were admitted following a cardiac arrest (both in and out of hospital). Their age, gender and survival to ICU discharge and overall hospital discharge were recorded.

**Results:** 210 female patients and 309 male patients of varying ages were admitted to our ICU following a cardiac arrest. The mortality for both genders increased with increasing age.

Overall survival to the time of ICU discharge following a cardiac arrest was similar for both females (44.3%) and males (48.5%). Figures 1 (female) and 2 (male) below show the number of patients who survived or died on ICU discharge, by age and gender. Mortality rates increased when reviewing hospital outcome, as some patients died following discharge to the ward.

**Conclusions:** Overall mortality in our ICU following a cardiac arrest at any age is at least 50%, with the general trend appearing to rise with increasing age. More male patients were admitted to ICU following a cardiac arrest than female, with similar survival rates for both male and female patients.

More research could be undertaken to assess whether survival rates following a cardiac arrest have improved since 1993 and also to compare outcomes following either an in or out of hospital arrest.

**References**

1. Nolan JP et al. Resuscitation 85:987-992. 2014

**Fig. 1 (abstract P433).**
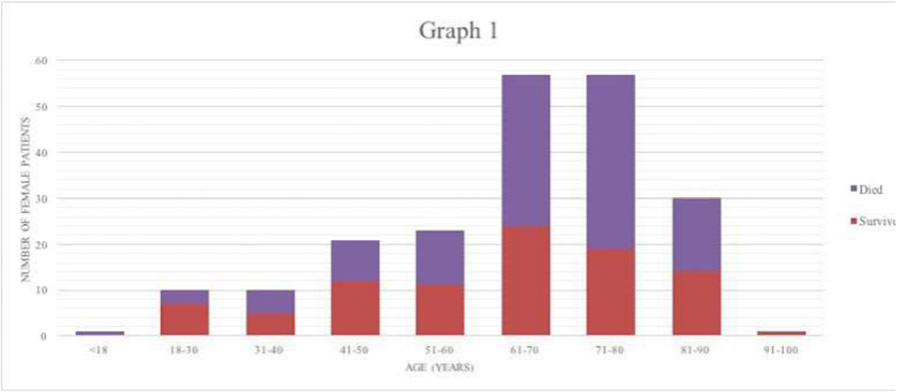
ᅟ

**Fig. 2 (abstract P433).**
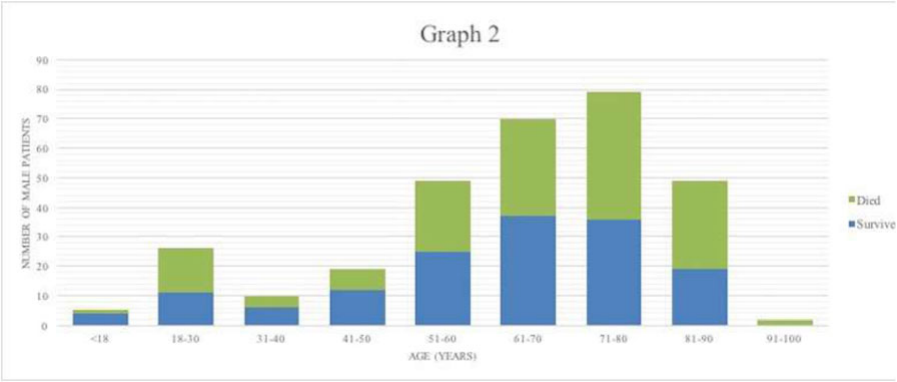
ᅟ

## P434 A retrospective analysis of the effects of opioids and ketamine on the survival and neurological status of patients in the postresuscitation period

### A Konkayev^1^, V Kuklin^2^, N Akhatov^3^, M Konkayeva^1^

#### ^1^Astana Medical University, Astana, Kazakhstan; ^2^Akerhus university hospital, Oslo, Norway; ^3^National medical center, Astana, Kazakhstan

**Introduction:** In a retrospective study from the Pittsburgh clinic, which analyzed survival data from 591 patients admitted to a hospital with a cardiac arrest outside the hospital, it was found that patients with opioid overdose showed significant improvements in neurological status when discharged from the hospital compared with patients who did not receive opioids [Elmer J. et al., 2015].

**Methods:** After local ethic committee approval 190 case-records of patients with cardiac arrest and subsequent resuscitation for the period 2006 - 2017 in the clinic of traumatology and orthopedics in Astana were analyzed. Criteria for inclusion in the study were hospital cardiac arrest, trauma to the musculoskeletal system.

**Results:** Out of 190 case-records, 17 (8.9%) patients with out-of-hospital cardiac arrest were excluded. Among all hospital stops of blood circulation, we found only 25 successful CPR (14.5%). Among the patients who were successfully resuscitated, 2 groups were identified: I - 16 patients (64%) who received ketamine or/and opioids before the blood circulation stopped (0-180 minutes); II - 9 patients (36%) who did not receive these medicines. The mean age in group I of patients was 39.1 ± 5.7 years, in group II - 43.2 ± 6.2 years (p> 0.05). Patients of the second group had an average life expectancy of 2.7 ± 0.9 days, with a maximum postresuscitation life of 4 days. Patients of the first group were in the hospital for 17.9 ± 2.1 days (p < 0.05), with a maximum period of 98 days. In the first group, the final neurologic evaluation according to the Glasgow scale was 11.4 ± 2.3 points, while in the second group it was 6.2 ± 1.2 points (p < 0.05).

**Conclusions:** A retrospective analysis revealed a better survival and neurological outcome in patients who received ketamine or/and opioids before circulatory arrest.

## P435 The prognostic value of simplified bispectral EEG monitoring in post-cardiac arrest patients

### V Vanstallen, W Eertmans, M Vander Laenen, M Vander Laenen, P Vanelderen, J Dens, F Jans, D Mesotten, C De Deyne

#### Ziekenhuis Oost-Limburg, Genk, Belgium

**Introduction:** Raw simplified EEG tracings obtained by a bispectral index (BIS) device significantly correlate with standard EEG [1]. This study aimed to investigate whether simplified BIS EEG tracings can predict poor neurologic outcome after cardiac arrest (CA).

**Methods:** Bilateral BIS monitoring (BIS VISTATM, Aspect Medical Systems, Inc. Norwood, USA) was started following ICU admission. Six, 12, 18, 24, 36 and 48hrs after targeted temperature management (TTM) at 33°C was started, raw simplified BIS EEG tracings were extracted and reviewed by two neurophysiologists for the presence of burst suppression, cerebral inactivity and epileptic activity. At 180 days post-CA, neurologic outcome was determined using the CPC scale, where a CPC1-2 and CPC3-5 corresponded to good and poor neurologic outcome, respectively.

**Results:** Of the 75 enrolled CA-patients enrolled, 40 had good and 35 poor neurologic outcome. With a positive predictive value (PPV) of 1.000 and a negative predictive value (NPV) of 0.606, epileptic activity within 6-12hrs predicted a CPC3-5 with the highest accuracy. Epileptic activity within time frames 18-24hrs and 36-48hrs showed a PPV for poor outcome of 0.917 and 0.938, respectively. Cerebral inactivity within 6-12hrs had a poor predictive power (PPV=0.545, NPV=0.566). In contrast, cerebral inactivity between 36-48hrs predicted a CPC3-5 with a PPV of 1.000 and a NPV of 0.597. The pattern with the worst predictive power at any time point was burst suppression with a PPV of 0.363, 0.529 and 0.500 at 6-12hrs, at 18-24hrs and at 36-48hrs, respectively.

**Conclusions:** Based on simplified EEG derived from a BIS device, both the presence of epileptic activity at any time as well as cerebral inactivity after the end of TTM can be used to assist with poor outcome prognostication in successfully resuscitated CA patients.

**References**

1. Haesen J et al. Crit Care 19(Suppl 1):434, 2015.

## P436 The helicopter as first response tool – Rio de Janeiro Fire Department experience.

### F Gonçalves^1^, R Vasconcellos^2^, C Karmiol^1^, J Bertelli^1^, V De Moraes^1^, R Bastos^1^, L Moura^1^, S Ramalho^3^

#### ^1^Corpo de Bombeiros do Estado do Rio de Janeiro, Rio de Janeiro, Brazil; ^2^Amil Resgate Saúde, Rio de Janeiro, Brazil; ^3^Harvard Medical School, Boston, USA

**Introduction:** The air medical rescue unit of the Rio de Janeiro Military Fire Department is a public funded service that covers the Rio de Janeiro State, Brazil since 1988. The Air Operations Division (GOA) manages a unit with 2 Eurocopter AS 350B2 air ambulances, 6 physicians and 6 nurses. Since 1988 more than 7,000 medical missions were performed, in 3 main categories: Air Medical Evacuation (EVAM), Inter-Hospital Transfers (TIH) and Neonatal Transfers (NEO). Knowledge of most frequent events and conditions allows better management of human and technical resources.

**Methods:** Descriptive analysis of GOA mission registries from October 2011 to October 2017.

**Results:** Flying 2023 hours, 1,606 missions were accomplished. EVAM accounted for 784 missions (49%), with a median mission time of 39 (interquartile range=28) min, followed by TIH with 552 flights (34%) and median time of 65 (IQR=70) min, and 270 (17%) were NEO missions with median time of 120 (IQR=92) min. Total time of aircraft usage was higher for TIH (39%), followed by NEO (32%). EVAM was the most frequent mission, however it accounted for 29% of aircraft utilization time, where most victims had traumatic brain injury (TBI) followed by other traumatic injuries (216 and 187 cases respectively). TBI victims were predominantly males (83%) with a median age of 30(IQR=23) years. Most commonly, TBI is a consequence of transportation accident (75%), where a motorcycle was involved in 31%, car collision in 24% and pedestrian run over 24% of the cases.

**Conclusions:** GOA utilizes the air ambulance helicopter as a first response tool in 49% of total missions, where respect for the Trauma Golden Hour is paramount. Traumatic brain injury is the most prevalent diagnosis at the scene of event. Therefore, GOA training and equipment must be tailored to meet this demand, which translates in stabilization of critical patients outside hospital environment with limited resources.

## P437 Safety profile during intra-hospital transportation of critically ill patients.

### V Veiga, T Alvarisa, N Postalli, R Vale, P Travasos, S Rojas

#### Hospital BP - A Beneficência Portuguêsa de São Paulo, São Paulo, Brazil

**Introduction:** The intra-hospital transport of critical patients is associated with adverse events and worse outcomes. The objective of this study was to evaluate the safety profile of intrahospital transport after the creation of a specific group for this purpose.

**Methods:** Evaluated all the transports of critical patients from October 2016 to September 2017, in a large hospital, after the creation of a group consisting of intensive care physician, nurse and physiotherapist. Clinical and non-clinical complications related to the transport and outcome of the patients were evaluated.

**Results:** A total of 1,559 transports were performed, 54.7% of the male patients and 60.9% of the patients being hospitalized. 10.6% were under mechanical ventilation and 19.8% under vasoactive drugs. At the time of transport, 78.8% were clinically stable. During transport, 7.5% presented clinical complications, being more frequent hemodynamic instability (43 patients) and respiratory failure (21 patients). Non-clinical complications occurred in 125 patients (8.0%), and communication failures were responsible for 79.2% of the occurrences. In 29 cases (1.9%) there was worsening of the clinical conditions during transportation, and in only one case this worsening resulted in an increase in the length of stay in the ICU and in the hospital, with no correlation with deaths.

**Conclusions:** The implantation of a group specialized in critical patients to carry out in-hospital transport made the process safer with complications rates lower than literature and guarantee better quality of care.

## P438 Clinical profile of patients admitted to ICU due to acute poisoning

### MP Benitez Moreno^1^, E Curiel Balsera^1^, MC Martínez González^1^, S Jimenez Jimenez^2^

#### 1Intensive Care Unit, Hospital Regional Universitario Carlos Haya, Malaga, Spain; ^2^Hospital Regional Universitario Carlos Haya, Málaga, Spain

**Introduction:** Patients suffering from acute intoxication, whether voluntarily for autolytic or accidental purposes, often require life support in intensive care units.

**Methods:** Retrospective observational study of all patients admitted for acute intoxication who required admission to the ICU of the Regional Hospital of Malaga between January 2012 and August 2016, older than 14 years with admission to the ICU for intoxication of any kind. We study patient characteristics in terms of age, sex and medical history, type of toxicity, severity and evolution in our unit.

**Results:** We found 70 cases of patients who required admission to the ICU due to acute intoxication, of which 55.6% were women. The average age was 47.36 (standard deviation 18.22). The average stay in ICU was 5.04 (standard deviation 8.09). 54.2% of patients had a psychiatric history. As other background highlights, 19.4% were addicted to illegal drugs and 25% were hypertensive. Most patients took more than one toxic 83.3% and intoxication was voluntary in 84.7% versus accidental in 12. 5% of cases. The toxic was known in 68%. The most used benzodiazepines in 26.4% of the total. The main cause of admission to the ICU was due to neurological deterioration in 49 of the cases registered and mechanical ventilation was necessary in 44 patients. The maximum time in mechanical ventilation was 34 days. The infection occurred in 24.3%, with the majority being respiratory infection. The 4.7% died in ICU. The hospital stay presented an average of 9.3 days.

**Conclusions:** The profile of a patient admitted to the ICU due to acute intoxication is that of a woman of middle age and psychiatric history, with voluntary intoxication of several toxic substances and requiring mechanical ventilation for a low level of consciousness for an average of 3 days. The survival is very high and it would be necessary to analyze the possible relapses of these patients.

## P438A Mushroom that break hearts: a case report

### E Karakoc, K Demirtas, S Ekemen, A Ayyildiz, B Yelken

#### Eskisehir Osmangazi University, Eskisehir, Turkey

**Introduction:** Because of the high mortality and morbidity mushroom poisoning is a significiant medical emergency [1]. Amanita phalloides (A. phalloides) is responsible for the 20% of the mortality in adults caused by mushroom poisoning. It causes damage in liver, kidneys and rarely pancreas, causing encephalopathic coma, disseminated intravascular coagulation, hemorrhage, hypovolemic shock and death but its effect on cardiac functions has not been established yet. There are three main groups of toxins;phallotoxins, virotoxins and amatoxins;amatoxin is the common responsible toxin from the fatality. We aimed to present a 44-year-old woman poisoned by mushroom complicated with hepatic,renal and cardiac toxicity

**Methods:** Patient with nausea and vomiting started 48 hours after mushroom eating,creatine kinase MB 2.73 ng/mL and cardiac troponin I 0.02 ng/mL Her blood urea nitrogen, creatinine levels and liver enzymes were higher than upper limits in lab tests (Table 1); she was admitted to ICU, treated for acute renal failure by hemodialysis.Plasmapheresis was applied against potent mushroom toxins. At 5.day in ICU, hypoxemia and severe swelling resistant to ultrafiltration was evaluated as a global left ventricular hypokinesia with ejection fraction(EF) 20%, end-diastolic diameter of 5.9 cm, and systolic pulmonary artery pressure (SPAP) of 40 mmHg. Oxygen was administrated to treatment.Urine output improved at 6.day, three more plasmapheresis sessions were performed. hypoxemia was recovered,liver enzymes and creatinin levels decreased

**Results:** At control EF measured was 44%, end-diastolic diameter of 4.9 cm, SPAP of 25 mmHg.than at the 15.day patient discharged from the ICU.After a year follow up assessment she has no complaints

**Conclusions:** One of the major problems for amanita poisoning is diagnosis. Patients who had mushroom poisoning should also be evaluated especially in terms of cardiac dysfunction with clinic signs, ECG, cardiac enzyme tests and ECO

**References**

1. Unverir et all, Human & Experimental Toxicology 26: 757–761, 2007

**Table 1 (abstract P438A).** Laboratory data

Variable	Normal range	Days		
		1	5	15
Blood urea nitrogen	(6.0 – 20 mg/dL)	24	56,3	10
Creatinine	(0.6 – 1.2 mg/dL)	4,64	5,57	0,88
Alanine aminotransferase	(1 – 41 U/L)	134	578	21
Aspartate aminotransferase	(1 – 38 U/L)	48	800	20

## P439 Predictive factors for secondary ICU admission within 48 hours after hospitalization in a medical wards from the emergency room

### M Cancella De Abreu^1^, S Herminger^1^, A Rousseau^2^, P Hausfater^1^

#### ^1^Hôpital Pitié Salpêtrière, Paris, France; ^2^Hôpital Saint Antoine, Paris, France

**Introduction:** The characterization of clinical and/or biological variables found in the emergency room predictive of a secondary admission in ICU would help to improve the identification of patients at risk of aggravation in order to avoid the associated consequences, such as, an increased mortality and increased hospital stay.

**Methods:** This is a retrospective monocentric study of 3 years with patients admitted secondarily to a medical ICU within 48 hours of admission to the general wards from the emergency department in the Pitié-Salpêtrière hospital in Paris. Each case was matched to 2 controls. 62 different variables were collected in the emergency room.

**Results:** 319 patients, of whom 107 were cases and 212 controls were studied. Pneumonia is the diagnosis the most frequent in cases followed by sepsis (in 23 and 16%, respectively). 6 predictive factors of a secondary transfer in resuscitation are found: smoking status (p = 0.0205) if active smoker - OR 0.390 (IC 0.11-1.35), if old smoker - OR 5.64 (IC 1.47-21.62); the emergency consulting motif, (p = 0.001), if dyspnea - OR 20.39 (IC 4.03- 103.19), if fever - OR 7.61 (IC 1.53-37.75); the MEDS score >= 7 (p = 0.037) - OR 0.31 (IC 0.10-0.93); the IGS2 score (p <0.0001) - OR 1.13 (IC 1.06-1.20); (P = 0.001), taking an advice to an ICU: if the answer is to continue the care in the ward - OR 8.13 (IC 2.41-27.38), if the response is to not resuscitation - OR 0.14 (CI 0.01-2.24); and demanding a blood gas (p <0.0001) - OR 7.60 (IC 2.78-20.77).

**Conclusions:** The risk of being admitted secondarily to intensive care is higher if patients consult for dyspnea or fever, if they are old smokers, if they have a high IGS2 score, if an arterial blood gas is requested and if an ICU medical advice is taken. The MEDS score under 7 and being an active smoker seems to be protects for the unexpected transfer.

## P440 Preventing chest drain guidewire retention using the wiresafe

### C Malcolm, S Sinha, M Petsios, J Barker, P Young, M Mariyaselvam

#### The Queen Elizabeth Hopsital NHS Foundation Trust, Kings Lynn, UK

**Introduction:** We conducted a forced error simulation study, to test efficacy of the WireSafe for Seldinger chest drain insertions. Guidewire retention accounts for 50% of never events in UK emergency medicine [1]. The WireSafe is an engineered solution which prevents this [2] and is currently being implemented by NHS England as an evidence-based, cost-effective preventative engineered solution for central venous catheter insertions [3].

**Methods:** With IRB approval and written consent, 20 chest drain competent doctors, but with no knowledge of the WireSafe, were randomised to standard or WireSafe groups. They were presented with a scenario to complete, where a colleague who had been urgently called away midway during a Seldinger drain insertion. The manikin had a 12G drain in-situ with a visible guidewire ‘accidentally’ left in the lumen. If asked, the assistant stated only that the WireSafe was a new procedure pack containing the sutures and dressings which could be used as a sharps repository after placement.

**Results:** The WireSafe prevented guidewire retention (100% WireSafe v 10% Standard, n=20, p<0.001, Fisher’s Exact test). In the WireSafe group participants underwent searches of trolley, floor and/or sharps bin before the realisation of the intra-luminal location of the wire and all were removed.

**Conclusions:** The WireSafe was 100% successful in preventing the never event of chest drain guidewire retention alongside facilitating fixation and sharps disposal.

**References**

1. www.rcem.ac.uk/safetyalerts. 2017

2. Mariyaselvam et al. Anesthesiology. 127: 658-65, 2017

3. https://nhsaccelerator.com/innovation/the-wiresafe/

## P441 Acquired neuromuscular weakness in eldery patients with femoral bone fracture, could we decrease the incidence?

### D Pavelescu, I Grintescu, L Mirea

#### Emergency Hospital Floreasca, Bucharest, Romania

**Introduction:** Acquired neuromuscular weakness(ANMW) is a serious problem in eldery, with high incidence, morbidity and mortality. It appear early after an acute stress, last for months and frequently has a difficult recovery

**Methods:** After Informed Consent, 127 patients 70-101 yo admitted in the Orthopedic Surgical Department with traumatic femoral bone fracture, were enrolled in an one year prospective, observational study. We assess the incidence of ANMW on admission, at discharge and at 3 months after using the Medical Research Council Sume Score, the time until surgery, the type of anesthesia, the severity of postoperative pain using VAS Score, the incidence of postoperative neurocognitive dysfunction, using MMSE score, the adecquate postoperative nutrition

**Results:** At admission, 13 patients already had neuromuscular weakness and were excluded from the study.At 3 postoperative day 16 patients developed ANMW. At discharge from the hospital, 49 patients were diagnosed with ANMW and were allocated in group B, the others without ANMW were allocated in group A.

The time until surgery was significantly higher in group B 67.3 vs 10.5 h, p<0.05: Concerning the type of anesthesia, in group A 84.6% had spinal/regional techniques of anesthesia vs 65.3% in group B, p<0.05. Concerning VAS score at 24 h, 51% of patients from group B experienced severe pain vs 6.15% in group A and 34.6% of group B experienced moderate pain vs 16.9% in group A, p<0.05 The incidence of postoperative neurocognitive dysfunction was 6.15% in group A vs 36.6% in group B, the caloric intake was 87.2% of the requirements in group A and 61.3% in group B

**Conclusions:** We could conclude that are many important factors implied in the development of ANMW and some of them we could influence by early surgical procedure, loco-regional techniques of anesthesia, adequate pain control and optimal nutrition.

## P442 Does Psoas Lumbar Vertebral Index (PLVI) have any prognostic value in the elderly population with major trauma?

### J Lowe, C Bigham

#### Derriford Hospital, Plymouth, UK

**Introduction:** Previous literature has suggested low Psoas Lumbar Vertebral Index (PLVI) scores are associated with worse morbidity following major trauma, possibly related to admission frailty [1].

**Methods:** Retrospectively analysis of Derriford Hospital’s Major Trauma Network Database from April 2014- April 2016 was performed. Inclusion criteria were patients aged 65 and over with an injury severity score (ISS) >=9 who had an admission pan CT trauma series. All data was collected prospectively and included initial Glasgow Coma Score (GCS), mechanism of injury, and a comprehensive list of injuries. Robust follow up included Intensive Care Unit (ICU) Length of Stay (LOS), Hospital LOS, 30 Day Mortality, and Glasgow Outcome Score (GOS) on discharge from hospital. PLVI was calculated as the mean psoas area at level of L4 pedicles indexed to the vertebral body area on the presentation trauma CT.

**Results:** 302 patients met the inclusion criteria. Dichotomizing PLVI into a high (n=143) and low values (n=159) either side of the mean did not demonstrate that PLVI is an independent predictor of a bad outcome: Death at 30 days (OR 1.061 0.982-1.146 p 0.133), GOS 1/2/3 on discharge from acute hospital (OR 0.996 (0.895-1.108) p=0.939).

**Conclusions:** Other factors are significant for worse prognosis on multivariate analysis: age, severity of injury and reduced GCS. More work is needed to identify sensitive and specific indicators of a poor outcome in elderly trauma patients to prevent inappropriate escalation of treatment and suffering.

**References**

1. Ebbeling L et al. Eur J Trauma Emerg Surg 40(1):57-65, 2014

## P443 Rapid ultrasonographic assessment of undifferentiated shock in hypotensive patients

### E Tesfaye, T Zewude

#### Tikur Anbessa Specialised Hospital, Addis Ababa, Ethiopia

**Introduction:** Rapid Ultrasound for Shock and Hypotension (RUSH) protocol involves pulmonary evaluation with cardiac, abdominal, and venous examination applied for all undifferentiated hypotensive patients in a focused manner to diagnose, monitor, and treat emergency medical conditions [1]. The objective of this study was to assess the importance of RUSH protocol in identifying specific type of shock (Obstructve, Cardiogenic, Distributive and Hypovolumic) leading to early intervention in Emergency Department.

**Methods:** The study design was prospective cross-sectional early bed-side ultrasound examination based on RUSH protocol on undifferentiated hypotensive patients in Tikur Anbessa Specialised Hospital. Patients received all needed standard therapeutic and diagnostic interventions without delay. They were followed until they received a final diagnosis after full evaluation.

**Results:** A total of 93 patients with undifferentiated shock were eligible for ultrasound evaluation, of which 85 were diagnosed with specific shock types based on RUSH protocol and 8 patients had normal evaluation. The overall kappa correlation of the RUSH exam compared with the final diagnosis was 0.88 which is an almost perfect agreement. The p value was P=0.00 95% CI [0.926, 1.003), so that there is a statistically significant positive relationship between initial Ultrasound impression and final diagnosis r (91) =.98, p=.000. Hypovolemic shock showed 95.6% sensitivity with PPV of 89.6% and 89.6 % specificity with NPV of 95.6%. Distributive shock was 62.5% sensitive with PPV of 90.9% and 88.2% specific with NPV of 98.7%. Both cardiogenic and obstructive shocks showed 100% specificity and sensitivity.

**Conclusions:** This study highlights the significant role of the RUSH protocol performed on initial evaluation of patients to diagnose shock etiology accurately and rapidly.

**References**

1. Volpicelli G et al. Intensive Care Med 39:1290-8, 2013.

## P444 Expected and observed medical events and emergencies during two mass events, gathering several hundred thousand participants: are prediction scores reliable?

### D Albiero^1^, M Migliari^1^, G Bellani^2^, A Andreassi^3^, R Stucchi^4^, E Albergoni^3^, M Caresani^1^, G Chiodini^3^, R Fumagalli^4^, G Foti^1^

#### ^1^ASST Monza, Monza, Italy; ^2^University of Milan Bicocca, Milan, Italy; ^3^Azienda Regionale Emergenza Urgenza, Milan, Italy; ^4^ASST Grande Ospedale Metropolitano Niguarda, Milan, Italy

**Introduction:** We aim to describe the observed medical events during two Papal visits (in 2012 and 2017) in the Milan area and compare them with those predicted by the commonly used Arbon score [1]. Published values in the literature are in the range of 0.5-2 for patient-presentation-rate to medical services (PPR) and around 0.03 (0.01-0.55) for transfer-to-hospital-rate (TTHR) both per 1.000 participants. To the best of our knowledge, most of the existing scores have been applied to the events within 200.000 people. Furthermore, existing works are limited to a descriptive analysis of single events in different locations.

**Methods:** This is a retrospective analysis of data collected during two events in the Milan Area: the papal visit in April 2017 was located in Monza, an outdoor vast location. A similar location was used for the papal visit in June 2012, the Bresso airport. The climate was mild (65%-69% humidity). Crowds was constituted manly by families, mood was relaxed and alcool was not available. It was estimated 400.000 participants in the 2017 and 450.000 in the 2012. Every medical event was recorded and PPR and TTHR calculated. Observed PPR and TTHR were compared with the expected calculated with Arbon regression model [1].

**Results:** The table summarizes medical PPR and TTHR observed and expected for the two events. The PPR was very similar for both events. In 2017 the TTHR was less than a half of that observed on 2012, likely due to different organization of the Medical Center. For both events, Arbon model underestimated patients presentations by 45% and 22%.

**Conclusions:** Current predictive models underestimate PPR in large mass religious events gathering 400.000 participants or more. Our data suggest that in these events a PPR of 0,6-0,8 can be expected and this estimate could be used to plan similar future events, while TTHR can vary widely.

**References**

1. Arbon P et al. Prehosp Disast Med 16(3):109-116, 2001.

**Table 1 (abstract P444).** Medical PPR and TTHR observed and expected for the two events

Event	2012	2017
Estimated Participants (approx)	450,000	400,000
Observed Patients presentations	367	241
Observed PPR (per 1000 participants)	0.8	0.6
Predicted patients presentations (Arbon score)	202	189
Observed hospital transfer	62	19
Observed TTHR (per 1000 participants)	0.138	0.05

## P445 Adasuve enables quicker dispositions of acute psychiatric patients in the emergency department

### K Hesse^1^, E Kulstad^2^, K Netti^1^, D Rochford^1^, R Shah^1^

#### ^1^Advocate Healthcare, Oak Lawn, USA; ^2^UT Southwestern, Dallas, USA

**Introduction:** Managing the special needs of patients who present with agitation or psychosis can pose a greater challenge to an already busy emergency department as their symptoms can escalate rapidly. Traditional antipsychotics used in the ED, such as haloperidol or ziprasidone often do not fully relieve patient’s symptoms and may require administration of repeat doses or additional medications such as benzodiazepines to achieve effective results. This can induce excess sedation which can lead to longer length of stay in the ED and requires additional time at the bedside by the ED physicians and staff to manage these patients. Adasuve® is an antipsychotic drug that works in a single-use device providing an aerosol form of Loxapine that is rapidly absorbed by the lungs which may offer faster symptom relief, allowing subsequent earlier psychiatric evaluation and disposition.

**Methods:** To test this hypothesis, data including time of physician assignment and time physician documented discharge disposition and number of hours physician was assigned to the patients was retrospectively collected from 407 patients who arrived to the emergency department presenting with agitation or psychosis that received Adasuve or other types of antipsychotic medication such as ziprasidone, haloperidol and benzodiazepines or a combination of the three.

**Results:** We found that physicians who administered Adasuve spent an average of 8.30 hours assigned to their patient compared to 11.42 hours when the physician administered any other type of antipsychotic medication. This resulted in a significant 3.12-hour difference (p < 0.002) between the two groups.

**Conclusions:** In conclusion, less time spent assigned to a patient that received Adasuve can be attributed to faster symptom relief which allowed the physicians to complete their psychological evaluations and develop dispositions more rapidly than with patients that received other antipsychotic agents.

## P446 Clinical work in language-discordant Emergency Department consultations

### A Cox^1^, D Cerf^2^, N Dauby^3^, P Humblé^4^, L Huygens^5^, S Li^6^, K Maryns^7^, P Mols^2^

#### ^1^Vrije Universiteit Brussel (VUB) and King’s College London (KCL), Brussels, Belgium; ^2^Saint-Pierre University Hospital (CHU), Emergency Department, Brussels, Belgium; ^3^Saint-Pierre University Hospital (CHU), Department of Infectious Diseases, Brussels, Belgium; ^4^Vrije Universiteit Brussel (VUB), Brussels Insitute of Applied Linguistics (BIAL), Faculty of Arts and Philosophy, Brussels, Belgium; ^5^Vrije Universiteit Brussel (VUB), Faculty of Medicine, Jette, Belgium; ^6^King’s College London (KCL),School of Medical Education, Shepherd’s House, Guys Campus, King’s College London, UK; ^7^Ghent University, Vakgroep Vertalen, Tolken en Communicatie Gent, Belgium

**Introduction:** Emergency Department (ED) clinicians are increasingly confronted with patients who do not speak their language. Emergency medicine is a predominantly oral activity in which medical errors result from poor communication. Outcome-based studies have shown that language barriers in the ED lead to prolonged consultations, diagnostic insecurity, extra test orderings, and have a negative impact on patient health. Less attention has gone out to unravelling how miscommunication arises in these consultations and what can be done to prevent or overcome it. To fill this gap, this paper describes the dynamics of communication during consultations across a language barrier in the ED.

**Methods:** Language-discordant ED consultations were audio-taped and multimodal contextual data were collected via ethnographic participant observation. The transcripts of the audio-recordings were analysed from a clinical and an interactional sociolinguistic perspective by an interdisciplinary research team.

**Results:** Communication across a language barrier generates a considerable amount of confusion. The causes, often invisible to the participants in the consultation, include misalignment of frames and goals, lack of mutual background information, lack of an adequate shared language repertoire and role conflicts. These causes are exacerbated by a context of stress, anxiety and tiredness. Language barriers are not absolute. Depending on the questions asked, the clinical tasks performed, and the communicative resources at the interactants’ disposal, the intensity of a language barrier can vary over the course of the consultation, generating a “communicative swing”. Interactants can use various strategies to prevent or repair miscommunication.

**Conclusions:** Our research generates new insights that are relevant for clinical practice in language discordant environments and can be embedded relatively easily into clinical skills training.

## P447 Prognosis of patients admitted in the emergency department with intermediate lactate blood level

### H Ghazali, H Ben Turkia, W Derouiche, I Chermiti, R Jebri, A Azzouzi, S Chiboub, S Souissi

#### Regional Hospital of Ben Arous, Ben Arous, Tunisia

**Introduction:** Objective: The measurement of lactate blood level is essential to evaluate the prognosis in critical phase.Many studies demonstrate that blood lactate level >= 4 mmol/l predict a high level of mortality in emergency department (ED). However, we don’t found enough studies for intermediate blood lactate level.

In this study we evaluate the prognosis of patients admitted in the ED with intermediate lactate blood level (2-3.9 mmol/l).

**Methods:** Prospective monocentric study over six months. Inclusion of patients older than 18 years old admitted in the intensive care unit (ICU) of ED with systematic measurement of blood lactate level at admission.The lactate assess was classified in 3 levels: low level (lower than 2 mmol/l), intermediate (2-3.9 mmol/L) and high level (>=4mmol/l). Patients weredivided in 2 subgroups: with or without hemodynamic instability. Prognosis was evaluated in hospital mortality at 7 day.

**Results:** Inclusion of 146 patients. Mean age =58±21, sex ratio=1.7. Level blood lactate n(%): : low (<2 mmol/L) 36 (25), intermediate (2-3,9 mmol/L) 66 (45) and high (>= 4 mmol/L) 43 (30). The mortality rate in the group of patient with intermediate lactate was 14 % at 7 day. In the subgroup of patient with normal blood pressure and intermediate blood lactate level, the mortality was estimated at 12.8% compared to 16% in the subgroup of patient with hemodynamic instability.

**Conclusions:** The intermediate lactate blood level in patients admitted in ED is associated to a significant risk of mortality. This population should be supervised closely and have an optimal management.

## P448 Respiratory emergencies at children at pre-hospital stage

### L Petcu, A Ghidirimschi, B Golovin, N Catanoi, N Scurtov

#### National Centre of Prehospital Emergency Medicine, Chisinau, Moldova

**Introduction:** The respiratory emergencies at pre-hospital stage are defined as clinical situations that endanger the child’s life and which require urgent transport to the Emergency Hospitalization Unit. In the structure of the incidence of respiratory emergencies in the Republic of Moldova at children, it records the highest percentage.

**Methods:** In the period of January 2016–June 2016 at the pre-hospital stage were registered per total 30957 requests of respiratory emergencies.

**Results:** From the total number of registered requests was determined a higher preponderance of respiratory emergencies at children in rural area of 61.02% and in urban area of 38.95%. The big respiratory emergencies was determined in 14.39% of cases of which respiratory insufficiency of II-III degree in 11.6% of cases affecting children with age of 0-12 months, respiratory distress 0.06% of cases at children of 0-6 months, acute laryngitis - tracheo-bronchitis 2.6% of cases of high incidence at children with age of 6 Months-2 years. The respiratory emergencies of II degree was of 81.4%, including acute pneumonia in 7.5% cases, acute bronchitis in 6.6%, acute respiratory tract infection associated with febrile syndrome in 67.5% cases. From the respiratory emergencies of III degree in the majority of cases are viral respiratory infections, representing 4.1% of cases. The first aid measures were carried out to all patients, as a result from the total number of patients, 86.8% were transported to the emergency hospitalization department with monitoring of hemodynamic indices.

**Conclusions:** As a result, from the total number of medical-surgical emergencies at children, the respiratory emergency records the highest incidence, most frequently affecting children with age of 0-12 months. In order to prevent major complications, it is necessary that the emergency nurse to identify the life-threatening signs and symptoms with their stabilization.

## P449 Fatigue-related risk in ED: the effect of reduction and proofing strategies on occupational performance and well-being

### P Bérastégui^1^, A Ghuysen^2^, A Nyssen^1^

#### ^1^Université de Liège, Liège, Belgium; ^2^Centre Hospitalier Universitaire du Sart Tilman, Liège, Belgium

**Introduction:** Emergency residents are particularly vulnerable to sleep deprivation due to persistent conflicts between work schedule and the biological clock. Recent approaches to address fatigue-related risk mainly focused on reducing work hours and ensuring sufficient recuperation time. Such approach has demonstrated its limits due to growing emergency rooms visits and emergency residents’ shortage. Dawson & McCulloch (2005) introduced the notion of proofing as a complementary approach to manage fatigue-related risk [1]. Fatigue proofing strategies (FPS) aim to reduce the likelihood a fatigued operator will make an error, in contrast of reduction strategies (FRS) aiming to reduce the likelihood a fatigued operator is working. Most formal risk control systems do not encompass the notion of proofing and FPS typically develop as informal practices. In this study, we aim to 1) identify informal reduction and proofing strategies used by residents and 2) to investigate how they relate to fatigue-related risk indicators.

**Methods:** First, we organized 4 focus-group with a total of 25 residents in order to identify informal strategies used to manage fatigue-related risk. Second, we designed a questionnaire assessing the frequency of use of each reported strategy. Third, we administered the questionnaire together with the Malash Burnout Inventory to a larger sample and conducted a prospective observational study. 32 residents participated in the study for a total of 181 shifts analyzed. We gathered sleep diaries, subjective sleepiness, reaction time, medical errors and performance ratings at different time points during day and night shifts.

**Results:** We conducted linear mixed-effect models to investigate the relationships between FRS, FPS, fatigue, performance and burnout.

**Conclusions:** Our results suggest that FRS and FPS allow residents to maintain acceptable occupational performance at the expense of individual factors.

**References**

1. Dawson et al. Sl Med Rev 365-80, 2005

## P450 Evaluation of the prescription of complementary examinations in the emergency department

### M Khaldi, H Sandid, E Missaoui, A Mahmoudi, M Methammem

#### CHU Farhat Hached, sousse, Tunisia

**Introduction:** Medicine of nowdays allows the clinician to use a large number of complementary examinations to facilitate and improve the management of the patient’s health, but unjustified prescriptions are frequently observed, leading to unnecessary effort for caregivers, laboratory and radiology technicians, not to mention the time lost for parents, the cost generated, the pain inflicted and the risks of error inherent in any investigation.

**Methods:** This is a prospective descriptive and analytical mono-centric study carried out in the Emergency Department of the Farhat Hached University Hospital of Sousse, Tunisia.

All patients were randomly recruited from during a three-month period from September 2016 to December 2016. Patients were selected for two hours randomly chosen during the day with exclusion criteria : Patients under 15 years of age consulting for non-traumatic pathology victims of cardio-respiratory arrest or early death (before the establishment of the examination), outgoing patients against medical advice, patients escaped, patients referred with a pre-established diagnosis.

**Results:** 179 patients including 42.4% men and 57.6% women were included in our study, additional examinations were performed in 144 patients (80.4% of the population), it means 728 exams in total. 494 examinations were of biological nature (67.85%), 105 radiological examinations (14.42%) and 129 other types (17.73%). 68, 7% of these examinations were prescribed by interns, 29.6% by residents and only 1.7% by seniors who were only informed in 48% of cases. Patients stayed on average 178 minutes (approximately 3 hours) with extremes of 10 minutes to 1540 minutes (25 hours and 35 minutes).

**Conclusions:** One of the main causes of delayed recovery of complementary examinations is the lack of coordination and management of different health personnel, doctors, nurses and stretcher-bearers.

## P451 Cardiogenic shock complicating st segment elevation myocardial infarction: prognosis in the emergency department.

### F Riahi, S Souissi, M Mabrouk, A Aloui, C Ben Slimane, W Derouiche, A Zoubli, N ElHeni

#### Regional Hospital of Ben Arous, Ben Arous, Tunisia

**Introduction:** Cardiogenic shock (CS) continues to be the most common cause of death in patients hospitalized with ST-segment elevation myocardial infarction (STEMI). Immediate diagnosis and early reperfusion were required to improve the prognosis.

objective: To identify prognostic factors related to mortality in CS complicating STEMI admitted in Emergency department (ED).

**Methods:** Prospective observational study (2009-2016). Inclusion: STEMI with CS. Criteria for CS: systolic blood pressure less than 90 mm Hg for longer than 30 minutes and signs of impaired organ perfusion. The prognostic factors related to mortality in ED were identified by a multivariate comparative study.

**Results:** Among the 902 patients presented with STEMI, the overall incidence of CS was 8% (n=75). Mean age was 64 +/- 10 years, sex ratio was 3. The median delay of consultation was 180 min. Fibrinolytic drug was administered in 42 patients with a success rate of 20%. Mortality in ED was 21%. The univariate comparative study between dead (N=16) and living patients (N=59) identified the following mortality-related factors: onset of chest pain - first medical contact (352+/-280 vs 295+/-290 min), respiratory rate (27+/-6 vs 20+/-8 cycles/min), killip score of III or IV (69% vs 34%, p<0.001), extensive anterior infraction (69% vs 37%, p=0.02), right ventricular infraction (44% vs 17%, p= 0.02). In Multivariate analysis, mortality-related factors were: anterior infraction, Odds ratio(OR): 2.83; 95% confidence interval CI (1.24-6.71), the right ventricle infarction OR: 3.80; 95%CI (1.49-9.70) and killip score III or IV OR: 2.61; 95% CI (1.22-5.56).

**Conclusions:** Among patients who had CS complicating STEMI, extensive anterior infarction, right ventricle infarction and killip score of III or IV at the admission are independent factors of poor prognosis that require early transfer to Percutaneous Coronary Intervention capable center without delay.

## P452 Drug-induced thrombocytopenia in the critically ill patients

### Y Panahi, N Sobhani, MA Safarpour, M Chahabi

#### Baqiyatallah University of Medical Sciences, Tehran, Iran

**Introduction:** Drug-induced thrombocytopenia are severe complications in intensive care unit (ICU) that can be associated with significant morbidity and mortality. The aim of this study is to investigate the etiology and risk factors of the drug-induced thrombocytopenia associated with various drugs in the ICU.

**Methods:** One hundred and forty-four critically ill patients during the 6 months entered into this study. Patients monitored for 14 days after ICU admission. During this period of time complete blood count, ALT, AST, Serum creatinine, Lactate, blood PH, Na, K, Mg and calcium measured before, 7, and 14 days’ post ICU admission. During this period of time SOFA score, APACHE II Score, RASS Score, concurrent disease, sepsis, drugs with exact dosage and time of administration investigated daily. The study was a descriptive and analytical that conducted in the general and surgical ICUs. Patients had at least 18 years old age who had been admitted to the ICU for more than 48 hours. Full medical and hematological, biochemical profile and history and all drug were recorded and analyzed.

**Results:** The total of 144 patients was included. The incidence of thrombocytopenia was 45.9% and the highest rate was recorded in the period of 3-5 days post ICU admission (30.1%). There was no correlation between age, sex, the cause of admission, type of ICU, mechanical ventilation and rate of thrombocytopenia. Underlying diseases such as diabetes mellitus, Serum creatinine level and mean atrial pressure had a correlation with thrombocytopenia. APACHE II and SOFA scores were a significant positive correlation with thrombocytopenia (P<0.001, P=0.005). Drugs include vancomycin, fluoroquinolones, digoxin, and colistin has a correlation with thrombocytopenia (P<0.001).

**Conclusions:** The incidence of thrombocytopenia in the patients admitted to the ICU was high. Thrombocytopenia could an indicator for severity of the diseases. Therefore, determination of thrombocytopenia and it’s etiology in the ICU is very important.

## P453 Effectiveness of simulation-based learning in intravenous medication safety: a randomized controlled study

### JC Servotte^1^, I Bragard^1^, AF Donneau^1^, N Dardenne^1^, M Guillaume^1^, R Tubes^1^, B Cardos^1^, B Pilote^2^, IL Simoneau^3^, IL Simoneau^3^, A Ghuysen^1^

#### ^1^Université de Liège, Liège, Belgium; ^2^Université Laval, Québec, Canada; ^3^Cégep de Sherbrooke, Sherbrooke, Canada

**Introduction:** This randomized controlled study assessed the impact of a 3-hour intravenous medication safety simulation-based learning (SBL) on self-efficacy, stress, knowledge and skills of nursing students. Medication administration error is a worldwide concern [1], that has been linked with a lack of knowledge and skills in safe medication administration among new graduate and student nurses [2-4]. Preventing medication errors could therefore involve training through simulation.

**Methods:** Participants (n=99) were randomly assigned either to the control group (CG, n=50) or the experimental group (EG, n=49). While CG and EG both had a traditional clinical internship, EG beneficiated in addition the 3-hour SBL, using standardized patients in the context of an intensive care unit. The two groups were assessed twice: at T0 and T1 (four weeks later), through an Objective Structured Clinical Examination (OSCE) and questionnaires. Two blinded experts rated the students OSCE with an evaluation grid.

**Results:** Mean participants age was 21,2. There were no statistically differences between groups at T0. Compared to the CG (0%), the EG increased its self-efficacy (+19.35%) with a significantly difference (p<0.001) at T1. The SBL conducted to a greater increase of knowledge and skills in the EG (respectively +150%, +128%) than in the CG (respectively +46% and +47%), with a statistically significant difference (p<0.0001).

**Conclusions:** Results reinforce the interest of a short SBL using standardized patients to improve medication administration. Clinical impact of these observations requires further evaluation to determine potential transfer in clinical settings and retention over time.

**References**

1. Donaldson LJ et al. The Lancet 389:1680-1682, 2017

2. Henneman EA et al. Applied Nursing Research 23:11-21, 2010

3. Whitehair L et al. Nurse Education Today 34:225-232, 2014

4. Mariani B et al. Clinical Simulation in Nursing 13(5):210-216, 2017

## P454 The effect of medication reconciliation in two ICUs in the netherlands

### L Bosma^1^, N Hunfeld^2^, R Quax^2^, E Meuwese^2^, M Van Kranenburg^1^, P Melief^1^, J Van Bommel^2^, P Van den Bemt^2^

#### ^1^Haga Hospital, The Hague, Netherlands; ^2^Erasmus University Medical Center, Rotterdam, Netherlands

**Introduction:** Medication errors occur frequently in the intensive care unit (ICU) and during care transitions.

Medication reconciliation by a pharmacist could be useful to prevent such errors. Therefore, the aim of this study was to determine the effect of medication reconciliation at the ICU.

**Methods:** A prospective 8-month intervention study with a pre- and post-phase was performed in Haga Teaching Hospital (2013) and Erasmus University Medical Center (2014). The intervention consisted of medication reconciliation by pharmacists at ICU admission and discharge.

The severity of potential harm of the medication transfer errors (MTE) (pADE=0; 0.01; 0.1; 0.4; 0.6) was scored. Primary outcome measures were the proportions of patients with >= 1 MTE at ICU admission and ICU discharge. Secondary outcome measures were the proportions of patients with a pADE score >= 0.01, the severity of the pADEs and a cost-benefit analysis. Odds ratio and 95% confidence intervals were calculated.

**Results:** Table 1 shows patient characteristics. Figure 1 shows the primary outcome measures (ORadj admission =0.18 [95% CI 0.11-0.30] and ORadj discharge = 0.24 [95% CI 0.15-0.37]). The proportion of patients with a pADE >=0.01 at ICU admission reduced from 34.8% to 8.0% and after ICU discharge from 69.5% to 36.2%. The pADE reduction resulted in a potential net cost benefit of € 103 per patient.

**Conclusions:** Medication reconciliation by pharmacists at ICU transfers is an effective safety intervention, leading to a significant decrease in the number of errors and a cost effective reduction of potential adverse drug events.

**Table 1 (abstract P454).** Patient characteristics

	Pre-intervention (n=266)	Intervention (n=212)	p value
Age (years), mean (SD)	61.3 (14.7)	61.8 (13.4)	p=0.73a
Sex, female (%)	98 (36.8%)	89 (42.0%)	p=0.25
Days on ICU, median (range)	3 (1-67)	3.5 (1-75)	p=0.56
APACHE IV, mean (SD)	79.2 (32.2)	73.22 (32.9)	p=0.06
Died in ICU, n (%)	60 (22.6%)	35 (16.5%)	p=0.10

**Fig. 1 (abstract P454).**
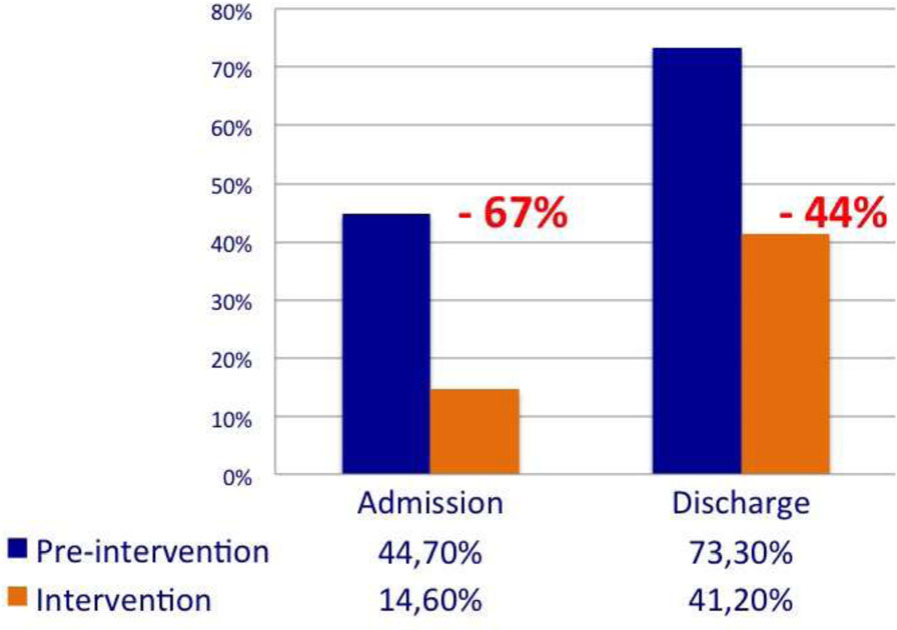
Proportion patients with ≥1 MTE

## P455 Evaluation of drug incompatibilities in ICU : development of an original method.

### M Benlabed, M Perez, R Gaudy, B Decaudin, G Lebuffe

#### Lille 2 University, Lille, France

**Introduction:** In intensive care unit, administration of numerous drugs in ICU patients via a central venous catheter provide a high risk of drugs incompatibilities. It has been reported in experimental studies [1] that particles issued of drug incompatibilities could induce thrombogenesis, microcirculation impairment and inflammatory response which could aggravate the occurrence of organ dysfunctions [2]. The objective of this study was to evaluate the occurrence of particles by reproducing in vitro the intravenous system and the drugs combination used in ICU for patients suffering either septic shock or Acute Respiratory distress Syndrome (ARDS).

**Methods:** First, we registered during a period of 6 months the most common central venous catheter system used in patients admitted for septic shock or ARDS in three University Hospital in Lille. The second part of the study was to reproduce in vitro the previous infusion system in order to quantify the amount of particles generated during a simulated period of 8 hours infusion. The egress of the IV line was connected to a dynamic particle counter QicPIC analyser (Sympatec Inc ; Clausthal Zellerfeld, Germany) (Fig. 1).

**Results:** The most common intravenous system observed was a three lumen central catheter. The proximal lumen was dedicated for vasoactive agents, the medial lumen for sedation and the distal lumen for the other drugs infused continuously and discontinuously..Among the drugs infused via the distal lumen of the central venous catheter, the most common combinations observed were heparin, piperacillin/tazobactam, esomeprazole and fluconazole administered simultaneously with glucose and parenteral nutrition. These drugs are known to be the cause of incompatibilities. The preliminary in vitro results showed high particulate contamination.

**Conclusions:** it is possible to determine a model for the study of drug incompatibilities in ICU.

**References**

1. Walpot H et al.: Anaesthesist, 1989

2. Jack T et al Intensive Care Med, 2012

**Fig. 1 (abstract P455).**
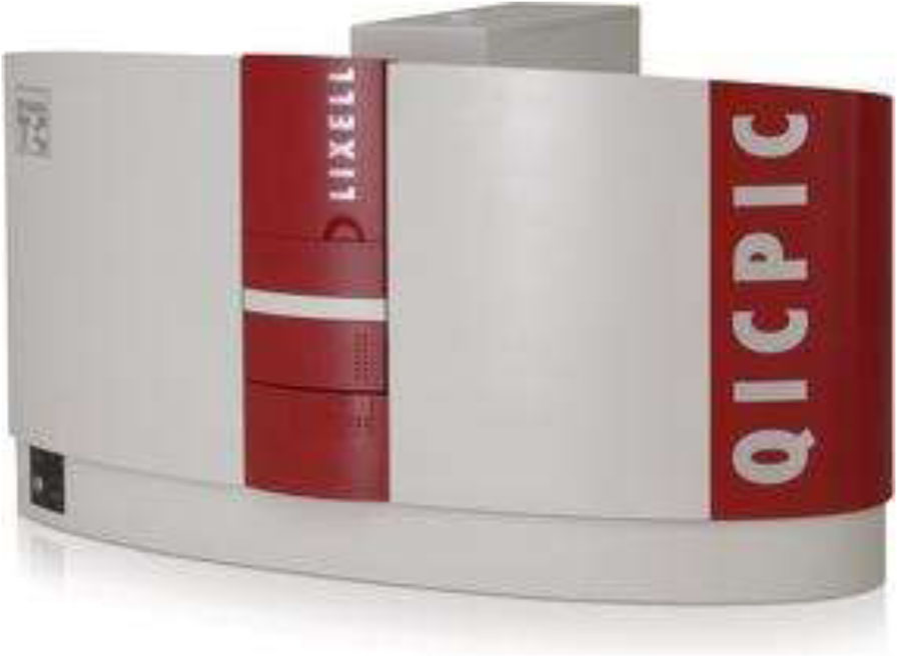
Qic pic particles analyser

## P456 Drug incompatibilities: a new challenge for the intensivist

### M Benlabed, M Perez, R Gaudy, B Decaudin, G Lebuffe

#### Lille 2 University, Lille, France

**Introduction:** The aim of this review is to evaluate and analyse the clinical consequences of drug incompatibilities in critically-ill patients, especially the incidence of organ dysfunctions and mortality.

**Methods:** A review of literature was conducted according to the PRISMA statement in June 2017, using Medline, ISI Web of Science and Clinicaltrials.gov. Data extraction: Eligible studies were case reports and randomised controlled trials (RCTs) that evaluated the effects of drug incompatibilities in critically-ill patients on morbidity or mortality as primary or secondary outcomes, or adverse events. Two investigators independently reviewed the eligibility of the study from abstracts or manuscript data. Data synthesis: Twelve articles met the selection criteria (Fig. 2). The six articles reporting RCTs concern only four RCTs. RCTs were single-centre studies comparing infusion with or without filter. Two of them included adult patients. The others included pediatric and neonatal intensive care unit patients. Primary endpoints were Systemic Inflammatory Response Syndrome (SIRS), organ failure, overall complication rate, bacteremia, sepsis, phlebitis and length of stay.

**Results:** The results are mixed with one RCT reporting a reduction in SIRS, organ failure and overall complication rate, two studies in disagreement over the occurrence of sepsis and one study reporting no impact on length of stay. The six articles on case reports show different drug incompatibility situations. They report pulmonary toxicity.

**Conclusions:** Little data is available on this topic. Infused particles may induce organ failure, in particular pulmonary toxicity and SIRS. Further studies are needed to establish a link between the level of exposure to drug incompatibilities and clinical implications.

**Fig. 1 (abstract P456).**
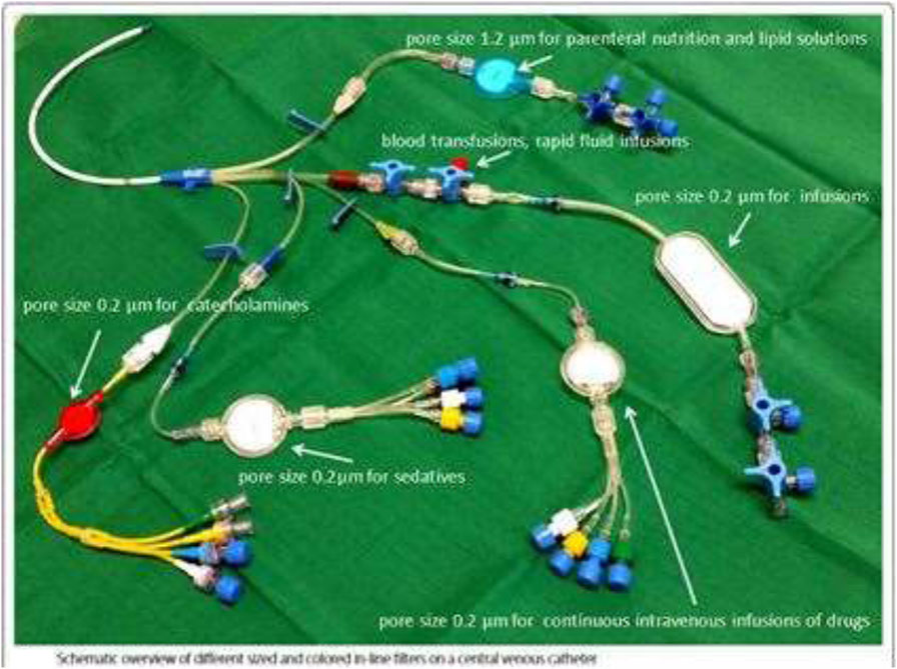
In line filtration

**Fig. 2 (abstract P456).**
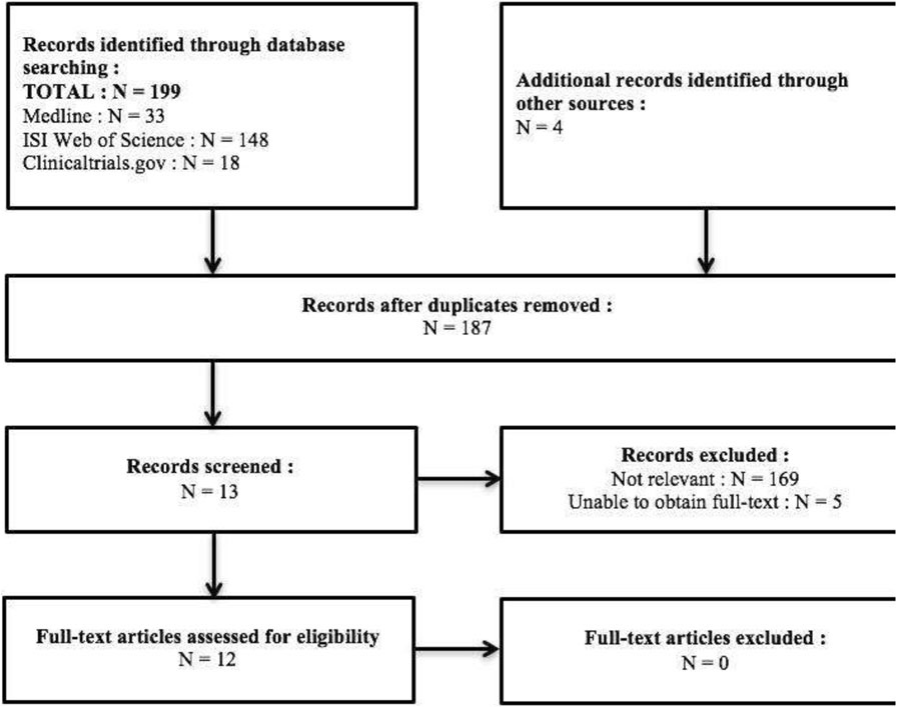
Flow chart

## P457 Worldwide management of brain dead organ donor: a systematic review of guidelines

### AJ Frenette ^1^, D Williamson^1^, B Rochwerg^2^, MJ Weiss^3^, I Ball^4^, K Serri^1^, F D’Aragon^5^, M Meade^2^, E Charbonney^1^

#### ^1^Hôpital du Sacré-Coeur de Montréal, Montreal, Canada; ^2^McMaster University Centre, McMaster, Canada; ^3^CHU de Québec, Québec, Canada; ^4^London Health Sciences Center, London, Canada; ^5^CHUS de Sherbrooke, Sherbrooke, Canada

**Introduction:** The clinical management of potential organ donors constitutes a challenge. Since targets and interventions are multiple and pertain to all physiological systems, caregivers may consider clinical practice guidelines as a precious tool. The objective of this systematic review was to identify clinical practice guidelines in order to describe practices around the world. High variability in practices among countries was expected. Findings will permit to identify the needs for future research.

**Methods:** We conducted a systematic review of clinical practice guidelines on the management of adult and paediatric brain dead organ donors. An electronic search strategy was conducted, using MeSh terms and appropriate key words, in 11 databases, from the earliest accessible date. A manual search was also conducted in Google in 9 different languages. Specific national transplantation organizations were directly contacted according to IRODAT data. Titles and abstracts were screened in duplicated and 2 independent reviewers abstracted data using a pre-tested data collection form. Quality of publications was assessed using the AGREE-II instrument.

**Results:** The search strategy using combined databases yielded 18 485 entries from which 8 guidelines were included. Combined with the results of the manual search, 25 clinical practice guidelines from 19 countries were finally included. We collected data on 7 predetermined clinical domains. Important heterogeneity in recommendations was found, particularly in the hemodynamic management, targets and monitoring as well as hormone replacement therapy domains. The majority of recommendations concerned adult donors only. The publications were in majority judged of low quality.

**Conclusions:** This review on the management of brain dead organ donor management highlights the high heterogeneity of recommendations and low methodological quality in guidelines around the world. Improvement in recommendations depends largely on future evidence-based studies

## P458 Implementing of active brain dead donor identification strategy does not affect potential donor pool: one year experience in a single donor center

### T Tamosuitis, I Maraulaite, A Trilikauskiene, D Damanskyte, D Lukminaite, N Balciuniene

#### Hospital of Lithuanian University of Health Sciences Kauno klinikos, Kaunas, Lithuania

**Introduction:** Insufficient identification of possible organ donors in the ICU is one of the main factors contributing to the loss of donors after brain death [1]. Up to 50% of potential donors might not be identified [2]. The aim of this study was to evaluate how active search of possible brain dead donors affect the potential deceased donor pool.

**Methods:** The strategy implemented at university hospital with 5 specialized ICUs from December 2016 to October 2017 and data compared to the matching period of the previous year. Donor coordinator visited all ICUs every day and selected patients who met possible brain dead donor criteria: 1) GCS <= 5; 2) severe brain injury. All data registered in original color coded follow-up system according to the patient status.

**Results:** A total of 237 patients were identified as possible donors. There was no significant difference of potential donor numbers in study period comparing to previous year (32 vs 31). Main causes of brain death remain intracranial hemorrhage and subarachnoid hemorrhage. The length of hospital stay of potential donors was significantly longer in study period comparing to previous year (4±4.86 vs 2.29±2.2, P=0.004). There was no significant difference of donor’s demographic data, conversion rates to actual donor or frequency of family refusals and medical contraindications.

**Conclusions:** Active search of brain dead donors neither increased total number of potential donors nor increased conversion rates and did not change a donor profile in our donor center. Longer observational period and more sophisticated follow-up system might be required.

**References**

1. European Directorate for the Quality of Medicines & HealthCare of the Council of Europe. Guide to the quality and safety of organs for transplantation. 6th edition. 39-46, 2016.

2. Richard V et all. Transplant coordination manual. 3rd edition. 37-43, 2014.

## P459 Current status and problems of organ transplantation before and after the enactment of the revised organ transplant law in Japan

### S Kamada, T Ikeda, S Ono, S Suda, T Nagura

#### Tokyo Medical University, Hachioji Medical Center, Tokyo, Japan

**Introduction:** The revised Organ Transplant Law was enacted in Japan in 2010. Under the revised law, it is now possible to donate organs with the consent of the family even if the intention of the potential donor is unknown. Organs from brain-dead children under the age of 15 can also be donated.

**Methods:** The aim of this study was to assess how to provide prompt transplant medical care and improve the donor’s condition. This was achieved by clarifying the problems encountered in the process leading to brain-dead organ transplantation at our institute before and after the enactment of the revised Organ Transplant Law. There were 79 cases of organ donation at our institute from January 2003 to June 2015. Among them, the background factors of 42 cases leading to organ donation were examined.

**Results:** The causes of the brain-dead condition were cerebrovascular disease (n = 15; 11 subarachnoid hemorrhage, 4 intracerebral hemorrhage), trauma (n = 8), suffocation (n = 5), cardiopulmonary arrest on arrival (n = 5), suicide by hanging (n = 3), cardiomyopathy (n = 1), and lethal arrhythmia (n = 1). The organs donated for transplantation were 54 kidneys, 32 eyes, 12 lungs, 8 livers, 7 hearts, and 6 tissues (i.e., heart valve, bone, and skin). The time lapses were as follows. The number of days from informed consent to family acceptance was 0.6 days before the enactment of the revised Organ Transplant Law and 1.5 days after the revision. The number of days from informed consent to organ removal was 2.5 days before the revision and 3.1 days after the revision. Even after the enactment of the revised Organ Transplant Law in Japan, it still takes about 3 days from informed consent to organ removal, with no current initiatives to shorten the time to organ removal.

**Conclusions:** Although 7 years have passed since the enactment of the revised Organ Transplant Law in Japan, there are still administrative and management problems that need to be addressed to achieve optimal organ transplantation.

## P460 The financial impact of proximity to a major airport on one critical care unit

### FJ Lamb, J McLoughlin, A Myers, TL Samuels

#### Surrey and Sussex Hospitals NHS Trust, Surrey, UK

**Introduction:** Workload resulting from in-flight emergencies has not been quantitatively analysed in the literature. For hospitals local to major airports, this may have significant financial implications.

**Methods:** Review was carried out of all cases admitted to East Surrey Hospital from Gatwick Airport over a 23 year period beginning in 1993. Data were collected by interrogating the ICNARC database. Demographics, presenting pathology and length of stay for each patient were recorded. In addition, the cost of care for patients admitted during 2016 was calculated using recent median figures for intensive care admission (local CCG rates).

**Results:** Since 1993, 196 patients were admitted from Gatwick Airport. This was approximately 2% of our critical care admissions. The mean (SD) age was 58.3 (14.5) years, and the median [IQR] length of stay 3 [1.1 – 6.7] days. Around 24% of these patients were non-UK or EU nationals and therefore not entitled to NHS care. Reasons for admission included cardiac (37.2%), respiratory (23.6%), central nervous system (12.6%), and gastrointestinal issues (12%). During 2016, 11 patients were admitted resulting in a total of 48.4 patient days in critical care. The total cost attributable to this group of patients was calculated to be £60,500.

**Conclusions:** There is a substantial additional financial burden on hospitals that regularly receive admissions from major airports simply due to their geographical location. There is no additional funding available for providing this service. The pattern of presenting conditions in our population is similar to that seen in previous reports describing in-flight emergencies [1]. Given the increasing accessibility of air travel and the economic pressures on healthcare providers, further analysis of the financial impact of this patient group on certain hospitals would be welcome.

**References**

1. Drew C. et al NEJM 368:2075-2083, 2013

## P461 A fast hug bid a day keeps the patient ok!

### E Sousa, T Leonor, R Pinho

#### Centro Hospitalar de Entre Douro e Vouga, Santa Maria da Feira, Portugal

**Introduction:** Regardless the underlying diagnose, providing meticulous supportive care is essential to critically ill patients management. In 2005, Vincent JL introduced the FAST HUG (Feeding, Analgesia, Sedation, Thromboembolic prophylaxis, Head of bed elevation, Ulcer prevention, Glucose control) mnemonic for recalling what he considered the key issues to review in daily clinical practice. Our Intensive Care Unit (ICU) decided to add BID (Bowel regimen; Indwelling catheter removal; De-escalation of antibiotics) indicators following some published data. Since 2013, the adequate use of this mnemonic became an instrument for quality of care evaluation. Objectives for each variable were designed; regular annual audits done. The present study aims to audit the use of this mnemonic in a portuguese tertiary hospital ICU, in 2017.

**Methods:** A prospective observational study was performed. Admissions in ICU staying at least one 00h00min and 23h59min period, during the first six months of 2017 were included. All mnemonic variables were recovered from ICU medical record database, as well as demographics, severity scores and clinical information. Data was analyzed with Microsoft Office Excel software.

**Results:** We included 119 admissions. The predictable global FAST HUG BID assessment was 1086 entries [one per each full day (00h00-23h59) in the unit, per patient]. The mnemonic was used in about 65% of the opportunities. The target thresholds were considered as achieved in 95% of entries (concordance equal or superior to 80%). Looking to individual variables, the best performance was achieved in H and U; worse performance was seen in S.

**Conclusions:** The daily use of this mnemonic aims to revisit important intervention sectors in critical patient. Applying the “Plan-Do-Check-Act” policy, this study allowed us to identify growth opportunities, reviewing or creating protocols, adopting more frequent training measures and seeking to take this model to other hospital areas.

## P462 Impact of incidents and adverse events in intensive care unit and its characteristics on outcomes

### E Siqueira, L Taniguchi, J Vieira Junior

#### Hospital Sírio LIbanês, Sao Paulo, Brazil

**Introduction:** Critically ill patients are usually exposed to adverse events (AE) due to acuity and complexity of care. AE might potentially result in disability or death, and increase in length of stay. Our aim was to assess the incidents and AE in a general intensive care unit (ICU).

**Methods:** This is a prospective cohort study conducted in a private tertiary hospital (Hospital Sírio-Libanês) in São Paulo, Brazil. All consecutive patients who were admitted to the ICU and all incidents and AE reported in the study period were evaluated. Univariate and multivariate analysis were used to identify risk factors associated with hospital mortality.

**Results:** Between May to November 2016 we studied 890 patients and 533 reported incidents and AE. Overall, 267 patients (30%) experienced some incident or AE during ICU stay. We found higher severity of illness (SAPS3 of 48 versus 44; p<0.001), mechanical ventilation (MV), use of vascular lines, drains and catheters, physical restraints, delirium and also an increased length of ICU (4 vs 2 days; p<0.001) and hospital stay (20 vs 11 days; p<0.001) and hospital mortality (24% vs 11%; p<0.001) among patients who experienced any incident or AE. Independent risk factors for hospital mortality in our logistic model were: higher SAPS3, MV and at least one adverse event during the ICU stay. Mortality was higher among patients who experienced late AE (>48 hours after ICU admission) compared to patients who experienced early AE (37% vs 19%; p<0.003). SAPS3, SOFA and MV were predictors of moderate and/or severe AE and a negative correlation between these events and ICU occupancy rate was found.

**Conclusions:** Patients who experienced incident or adverse event during ICU stay had poorer outcome. AE, mainly moderate or severe, MV and severity of illness were independent risk factors to mortality. There was a negative correlation between moderate or severe adverse event and ICU occupancy rate.

## P463 Monte Carlo modelling of patient flow can aid complex intensive care bed and workforce capacity planning.

### G Hadjipavlou, H Madder, J Titchell

#### Neurosciences Intensive Care, Oxford, UK

**Introduction:** Models for ICU populations based on the Queuing model use arrival rate, length of stay, and bed number [1,2]. These models lack the complexity of specialised ICUs with different admission types, and patient subpopulations.

**Methods:** We developed a Monte-Carlo simulation [3] with separate referral rates for emergency, elective, and ventilated patients. Bed occupancy is classified according to admission type with a conversion to prolonged ventilated stays at a rate of 4% [4]. We used data from our Neurointensive care unit to complete the parameters required for the model e.g. 13 beds and 1,725 referrals/day. Outcome measures were bed occupancy, and failed admissions. We tested two scenarios: increased referral rate (4.5/day), and increasing to 20 beds.

**Results:** The model simulated our intensive care where we have a high occupancy rate. Increasing referral rate led to a consumed ICU and an increase in failed admissions (Fig. 1). Lastly, increasing bed numbers eased pressures with fewer failed admissions.

**Conclusions:** We recommend a personalised ICU Monte-Carlo population model for specialist units for a more accurate representation of ICU bed occupancy. These ICU specific models should be more useful for predicting staff, bed and financial requirements in specialist units where healthcare resources are changing e.g. increasing geographical referral radius.

**References**

1. Williams, J. et al. Anaesthesia 70, 32–40 (2015).

2. Mathews, K. S. et al. Ann. Am. Thorac. Soc. 12, 886–894 (2015).

3. Kroese, D. P. et al. Wiley Interdiscip. Rev. Comput. Stat. 6, 386–392 (2014).

4. Lone, N. I. et al. Crit. Care 15, R102 (2011).

**Fig. 1 (abstract P463).**
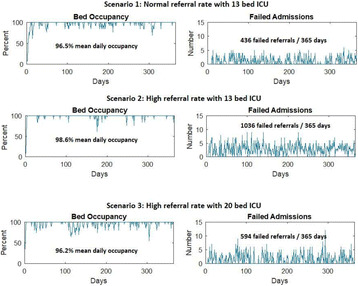
Monte Carlo simulations of bed occupancy under 1) normal, 2) increased referral, 3) increased referral and increased capacity conditions

## P464 Improving patient flow to enhance efficiency and quality of care

### I Goodhart, A Elrefaey, G Shepherd, N North, G Northfield, N Howard, A Puttappa, P Valdes

#### Addenbrooke’s Hospital,Cambridge University Hospitals NHS Foundation Trust, Cambridge, UK

**Introduction:** Overnight Intensive Recovery (OIR) is a 6-bed unit, providing care for high risk elective surgical patients in the first 24 post-operative hours. The Royal College of Anaesthetists report ‘The Pathway to Better Surgical Care’ [1] highlighted the importance of enhanced perioperative care for high risk patients. OIR is not a High Dependency Unit but patients receive invasive monitoring, vasopressors and enhanced respiratory support under the supervision of recovery staff and anaesthetists. Limited critical care capacity, greater complexity and an aging population are increasing demand. The Faculty of Intensive Care Medicine’s report ‘Critical Futures’ recommends establishing standards for level 1+ care. We aimed to establish scope of practice, measure efficacy and quality and improve standards.

**Methods:** Projects were registered and patient feedback was collected between January 2016 and January 2017. A Staff survey was completed by nursing, surgical and anaesthetic staff. Baseline efficiency was measured between July 2016 to January 2017. Changes were implemented and measures repeated in September 2017 and October 2017.

**Results:** >98% of patients reported satisfaction on all areas except noise, patient facilities for hand hygiene and being informed about timing of operations. Staff survey results revealed confusion regarding the interventions that are provided. Baseline capacity for new patients was 53%, bed occupancy varied between 1 and 12 per day (overflow to recovery) with overall capacity at 93.5% and mean length of stay (LOS) was 1.3 days (SD=0.7, n=481, =range 1-5). Following intervention, the LOS was reduced to 1.18 days (SD=0.4, n=112, range 1-3). New patient capacity was increased to 62% with a bed occupancy range 1-8.

**Conclusions:** Better patient flow increased occupancy and standards. Staff education and clear protocols are needed to improve patient booking and efficiency.

**References**

1. Preoperative Medicine the Pathway to Better Surgical Care. London: Royal College of Anaesthetists; 2015.

## P465 Morale: introducing the anaesthetic trainee confession session

### J Cuddihy, G Sivasubramaniam, D Mahtani, V Ponnaiah, V Ponnaiah, E Aziz

#### Guys and St Thomas’ Hospital, London, UK

**Introduction:** In clinical practice, when harm or potential harm occurs to patients, this can adversely impact upon the morale of staff involved and thereby affect clinical care delivered to subsequent patients. The personal narratives behind clinical incidents contain learning opportunities and individuals involved may reflect on the course of events and make changes to their practice to avoid recurrence. The aim of this study was to evaluate whether sessions enabling trainees to discuss their mistakes in a confidential environment improved trainee morale and safe clinical practice in an anaesthetic trainee cohort.

**Methods:** We conducted a survey amongst anaesthetic trainees in a London teaching hospital before and after a monthly, hour long, confidential, semi-structured, trainee lead “confession session” was introduced.

**Results:** Initial results demonstrated that 68% of respondents (N=30) had made a mistake resulting in patient harm with 84% of these individuals describing negative feelings about themselves as a consequence. Additionally, 97% of respondents had made a mistake causing a near miss, with 96% of these describing negative feelings as a result. Of note, only 55% of respondents felt comfortable discussing errors with more senior colleagues, whilst 78% felt comfortable discussing errors with their peers. A follow-up survey identified that 100% of respondents (N=13) agreed that the session had the potential to improve clinical practice and trainee morale with 77% agreeing that their own clinical practice had improved from attending the sessions.

**Conclusions:** Clinical mistakes leading to harm and “near misses” are common and provide opportunities to improve care. This trainee lead “confession session” appears to improve trainee morale and may improve patient care by encouraging trainees to engage in a process that seeks to understand error through sharing stories in a non-judgmental setting.

## P466 Funnel plots for quality control of the Swiss ICU - minimal data set

### A Perren ^1^, B Cerutti^2^, M Kaufmann^3^, HU Rothen^4^

#### ^1^Ente Ospedaliero Cantonale, Ospedale San Giovanni, Bellinzona, Switzerland; ^2^Faculty of Medicine, University of Geneva, Geneva, Switzerland; ^3^University Hospital, Basel, Switzerland,^4^Bern University Hospital - Inselspital, Bern, Switzerland

**Introduction:** A clinical database should be representative of the labelled population and guarantee completeness and accuracy of collected data. Without explicit permission of the patients, Swiss laws regarding data protection do not allow external audits based on periodic checks of random samples, supposed to give a general pattern of accuracy. To test alternative methods for quality control we introduced the principles of statistical process control to derive funnel plots from the Swiss ICU – Minimal Data Set (MDSi).

**Methods:** The MDSi from all certified adult Swiss ICUs (2014 and 2015) was subjected to quality assessment (completeness and accuracy). For the analysis of accuracy, a list of logical rules and cross-checks was developed as e.g. range of SAPS II according to age. Errors were classified in coding errors (e.g. NEMS score > 56 points) or implausible data (NEMS without basic monitoring). We also checked for ICUs producing significantly more errors - outliers - (> mean ± 3 standard deviations [SD] or > 99.8% confidence interval [CI] of an adapted version of the funnel plots, which allows the presence of trends depending of the ICU’s size.

**Results:** A total of 164’415 patient MDSi (31 items/patient; 32 items for trauma patients) from the 77 certified ICUs.were investigated. We detected 15’572 patients (9.5%) with an overall sum of 3121 coding errors and 31’265 implausible situations. Implausible situations related to supposedly inaccurate definitions (diagnostic and patient’s provenance prior to ICU admission) and discrepancies in the logical rules between diagnostics and treatments. Figure 1 is an example for imprecise coding of the diagnostic: 11 ICUs declared having treated 14-61% of their patients without a defined diagnosis.

**Conclusions:** Accuracy of data in MDSi needs further improvement. Funnel plots may be useful for meaningful interpretation of data quality and permit to identify ICUs disproportionately generating inaccurate and/or implausible data.

**Fig. 1 (abstract P466).**
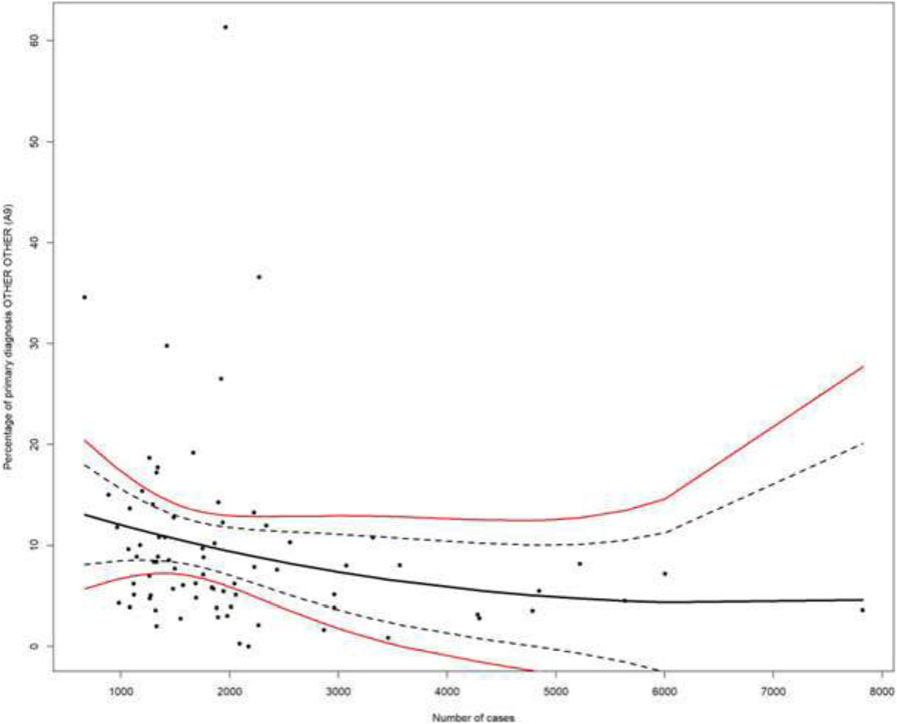
Funnel plot depicting for each ICU its relation between the number of treated patients (x-axis) and the percentage of these patients with imprecise coding of the diagnosis (y-axis). The red (dotted) lines denote the 99.8% (95%) Confidence Intervall. The central line represents “local medians” estimated by the Locally Weighted Scatterplot Smoother – method.

## P467 Effect of intensivist on the outcomes of advanced lung cancer who admitted to intensive care unit

### Y Cho^1^, J Song^2^, H Yang^1^, S Hong^1^, K Kim^1^, S Shin^1^, S Choi^3^, Y Lee^1^

#### ^1^Seoul National University Bundang Hospital, Seongnam, South Korea; ^2^Seoul National University Hospital, Seoul, South Korea; ^3^Semyung University, Jecheon, South Korea

**Introduction:** Lung cancer is the leading cause of intensive care unit (ICU) admission in patients with the advanced solid tumors. This study was aimed to elucidate the clinical factors associated with ICU mortality of advanced lung cancer patients and the effect of intensivist’s contribution on their clinical outcomes.

**Methods:** We included patients with advanced lung cancer including non-small cell lung cancer (NSCLC) with stage IIIB or IV and small cell lung cancer (SCLC) with extensive stage who admitted to ICU from 2005 to 2016. Multivariate logistic regression analysis was performed to find the variables associated with ICU mortality and in-hospital mortality. We applied autoregressive integrated moving average (ARIMA) for time-series analysis of the intenvention of intensivists.

**Results:** Among total 264 patients with advanced lung cancer, 85 patients (32.2%) were admitted ICU before introduction of organized intensive care at 2011, and 179 (67.8%) were admitted after 2011 (Fig. 1). The leading cause of admission was the respiratory failure (77.7%) and cancer-related event (34.5%) in terms of intensivist’s and oncologist’s perspective. Before and after 2011, the 30-day ICU mortality rate was 43.5% and 40.2% (p = 0.610), and the hospital mortality rate changed from 82.4% to 65.9% (p = 0.006) (Fig. 2). The length of stay (LOS) ICU and hospital were also statistically significantly decreased after 2011 (14.5 ± 16.5 vs. 8.3 ±8.6, p < 0.001; 36.6 ± 37.2 vs. 22.0 ± 19.6, p < 0.001) (Table 1). In multivariate analysis, admission after 2011 was independently associated with decreased hospital mortality (Odds ratio 0.42 95% confidence interval (CI) 0.21-0.77, p = 0.006). In autoregressive integrated moving average (ARIMA) models, the intervention of intensivist was statistically associated with reduced hospital mortality. (Coefficient -16.06, 95% CI -26.33 - -5.78, p = 0.002)

**Conclusions:** In patients with advanced lung cancer who admitted to ICU, the intervention of intensivist could be contributable to improve their clinical outcomes.

**Table 1 (abstract P467).** Clinical outcomes according to intensivist’s intervention

	All patients (N = 264)	Pre-2011 (N = 85)	Post-2011 (N = 179)	P value
30-day ICU mortality	109 (41.3%)	37 (43.5%)	72 (40.2%)	0.610
Hospital mortality	188 (71.2%)	70 (82.4%)	118 (65.9%)	0.006
Length of stay in ICU, days	10.3 ± 12.0	14.5 ± 16.5	8.3 ±8.6	<0.001
Length of stay in hospital, days	26.7 ± 27.3	36.6 ±37.2	22.0 ±19.6	<0.001

**Fig. 1 (abstract P467).**
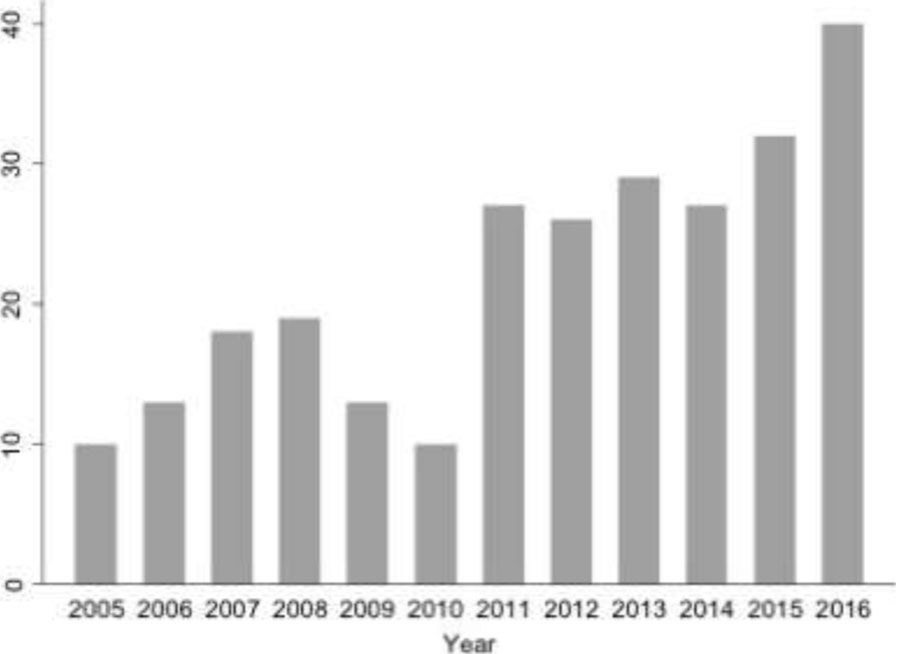
Trends of advanced lung cancer patients admitted to intensive care unit

**Fig. 2 (abstract P467).**
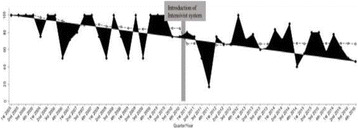
Run chart of hospital mortality of advanced lung cancer patients admitted to intensive care unit

## P468 Transfer time from the intensive care unit (ICU) and patient outcome

### S Chatterjee, S Sinha, M Bhattacharya, S Todi

#### AMRI Hospitals, Kolkata, India

**Introduction:** Patient outcome after ICU transfer reflect a hospital’s post-ICU continuity of care. This study assessed impact of after-hour ICU transfer on patient outcome.

**Methods:** Single-center, retrospective analysis of prospectively collected data between March 2016 to April 2017 at a tertiary care hospital in India.Patient data collected on all consecutive ICU admissions during study period. Patients were categorized according to ICU transfer time into daytime (08:00–19:59 hours) and after-hour (20:00–07:59 hours). Patients transferred to other ICUs or hospitals, died in ICU, or discharged home were excluded. Only first ICU admission was considered for outcome analysis. Primary outcome was hospital mortality; secondary outcomes included ICU re-admission and hospital length of stay (LOS). All analysis were adjusted for illness severity.

**Results:** Of total 1857 patients admitted during study period, 1356 were eligible for study; 53.9% were males and 383(28%) patients were transferred during after-hour. Mean age of two groups (daytime vs. after-hour 65.7±15.2vs.66.3±16.2 years) was similar(p=0.7). Mean APACHE IV score was comparable between those transferred during daytime vs. after-hour(45.6±20.4vs 46.8±22,p=0.05). Unadjusted hospital mortality rate of after-hour transfers was significantly higher compared to daytime transfers(7.1%vs.4.1%; p=0.02). After adjustment with illness severity, after-hour discharges were associated with a significantly higher hospital mortality compared to daytime transfers (aOR1.7, 95%CI 1.1,2.8; p=0.04). The median duration of hospital stay though higher for after-hour discharges, was not statistically significant in adjusted analysis (aOR1.1, 95% CI 0.8,1.4; p=0.5). ICU readmission rate was also similar in two groups (aOR 1.6, 95% CI 0.9,2.7; p=0.06).

**Conclusions:** After-hour transfers from ICU is associated with significantly higher hospital mortality. Hospital LOS and readmission rates are similar for daytime and after-hour transfers.

## P469 Payer status and intensive care unit (ICU) length of stay (LOS)

### S Chatterjee ^1^, M Bhattacharya^1^, S Sinha^1^, P Sen^2^, S Todi^1^

#### ^1^AMRI Hospitals, Kolkata, India; ^2^Jadavpore University, Kolkata, India

**Introduction:** This study compared ICU LOS among patients admitted under different payer status.

**Methods:** Retrospective analysis of prospectively collected data between October2016 to February2017 of a tertiary care ICU in India. Patient data collected on all consecutive ICU admissions. Primary and secondary outcomes were ICU LOS and hospital mortality respectively. ICU patients payer status were categorized as self-paid, corporate (paid-fully or partially-by- employer), and insurance (paid-fully or partially-by-third-party-payer). All analyses were adjusted for illness severity and ICU support (vasopressor use, mechanical or non-invasive ventilation, blood transfusion).

**Results:** Of 580 patients admitted during study period, 463 were eligible; 55.8% were males; 52.3% were self-paid, 35.9% insured, and 11.5% corporate-paid. Mean age differed significantly between groups (63.2±17.7 years - self-paid; 65.8±13.8 years - insured; 71.7±12.3 years - corporate; p=0.004). Overall mean APACHE IV score of 52.5±25 was similar across groups (p=0.08). No significant difference was noted in distribution of co-morbidities across self-paid, insured; and corporate groups (75.6%, 82.5% and 77.4%; p=0.3). Significantly higher number of patients received ICU support in self-paid and corporate groups compared to insured group (53.7%and 52.8% vs. 39.8%; p=0.02). Mean ICU LOS did not differ significantly among insured, self-paid and corporate groups (4.2±3.2 days vs. 3.7±2.9 and 3.9±2.2 days; p=0.4). Compared to self-paid group, adjusted ICU LOS did not significantly differ among corporate and insured patients (Insured aOR:1.99 95% CI 0.6,1.3, p=0.6; Corporate aOR:0.94 95% CI 0.5,1.8, p=0.8). Risk-adjusted hospital mortality was similar (Self vs Insured aOR: 0.8 95% CI 0.5,1.5, p=0.5; Self vs Corporate aOR 1.2 95% CI 0.6,2.5, p=0.7).

**Conclusions:** ICU LOS did not differ among patients under differing payer status in the study ICU.

## P470 Braden scale is predictive of mortality in critically ill patients, independent of its efficiency as a predictive tool of pressure ulcer risk

### D Becker^1^, TC Tozo^2^, IR Porto^3^, TT Chung^1^, P Duarte^3^

#### ^1^Hospital do Câncer/UOPECCAN, Cascavel, Brazil; ^2^Hospital São Lucas-FAG, Cascavel, Brazil; ^3^Hospital Universitario do Oeste do Parana, Cascavel, Brazil

**Introduction:** Braden Scale (BS) has been used as an important tool to predict risk of development of Pressure Ulcer (PU) in ICU patients. In its turn, the presence of PU correlates with higher illness severity and risk of death. Objectives: OBJECTIVES: To evaluate whether the values of the Braden scale in ICU patients correlate with a higher risk of death.

**Methods:** Prospective cohort study in 11 ICUs during a 45-day period. Adult (>18y) patients were followed until ICU discharge (death or discharged alive). BS was assessed (by the ICU nurse) on admission (1st day), 3rd, and 9th ICU day (only in the ICU), regardless of the presence of PU.

**Results:** During the study period, 332 patients were admitted (mean age 63.2 y, APACHE II 14.9, 52.1% male, admission causes: 50.3% medical, 12.0% trauma, 37.7% postoperative). The incidence of PU was 13.5%, and the BS was strongly correlated with the incidence of PU, either when mensured on the 1st day, on the 3rd or on the 9th day (p <0.001 PU x non-PU for each evaluation). In addition, BS values were strongly correlated with ICU mortality, regardless of the period evaluated (Fig. 1).

**Conclusions:** In addition to its role as a predictor of the incidence of PU in critically ill patients, BS was strongly correlated to ICU mortality.

**Fig. 1 (abstract P470).**
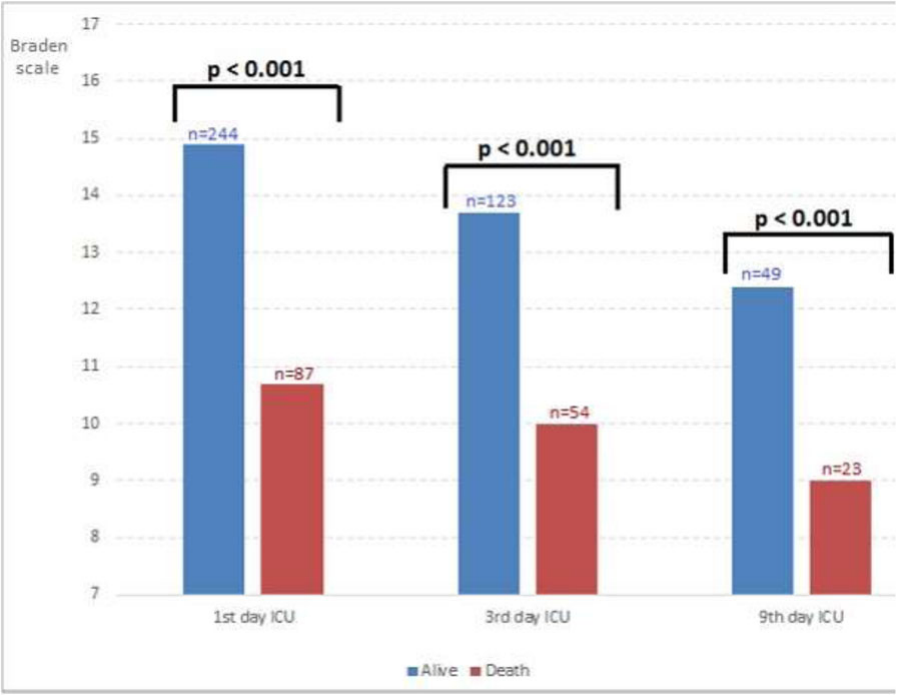
Braden scale on 1st, 3rd and 9th ICU day.

## P471 Triage tool for ICU referral: a pilot study

### L Griseto, R Ffrench-O’Carroll, S Campbell, O Tujjar

#### Sligo University Hospital, Sligo, Ireland

**Introduction:** Decisions when to refer and to admit patients to the intensive care unit (ICU) care are very challenging. Demand typically exceeds supply in ICU beds, which results in a constant need for evaluation of the processes involved in ICU referral and admission with a view to optimising resource allocation and patient outcomes. The aim of this study was to evaluate the theoretical impact of a newly designed triage tool for ICU referrals on a cohort of patients referred to ICU (Fig. 1).

**Methods:** We reviewed all patients consecutively referred to our ICU, whether admitted or not, in February 2017. Demographics, referring speciality, role of the referrer, comorbidities, the presence of advanced disease or terminal illness, the presence of acute organ failure, DNR status, reason for not admitting, and ICU mortality were recorded. A retrospective analysis of ICU referrals using a pilot triage tool was carried out independently by three authors.

**Results:** Forty-six patients were referred to our ICU over the study period. Of these, 34 (74%) were admitted. Patients were declined ICU if their admission was deemed unnecessary (50%), futile (33%), or were transferred due to bed shortage (16%). Of the patients referred, 25 (54%) had an advanced disease or a terminal illness. Of those, 18 (72%) were admitted, DNR status was unclear in 22 (88%), family was involved in 12 (48%) and their ICU mortality was 48%. By analysing retrospectively these referrals with the aid of a triage tool, we propose that the overall referrals could have decreased from 46 to 30 (42% percentage difference). DNR status and family involvement would have been clarified in all patients with advanced disease or terminal illness before ICU referral. Kappa score for inter-rater agreement was 0.78.

**Conclusions:** Adopting a triage tool for ICU referrals could reduce the overall proportion of inappropriate referrals and admissions. End-of-life discussion would also be proactively clarified prior to ICU admission.

**Fig. 1 (abstract P471).**
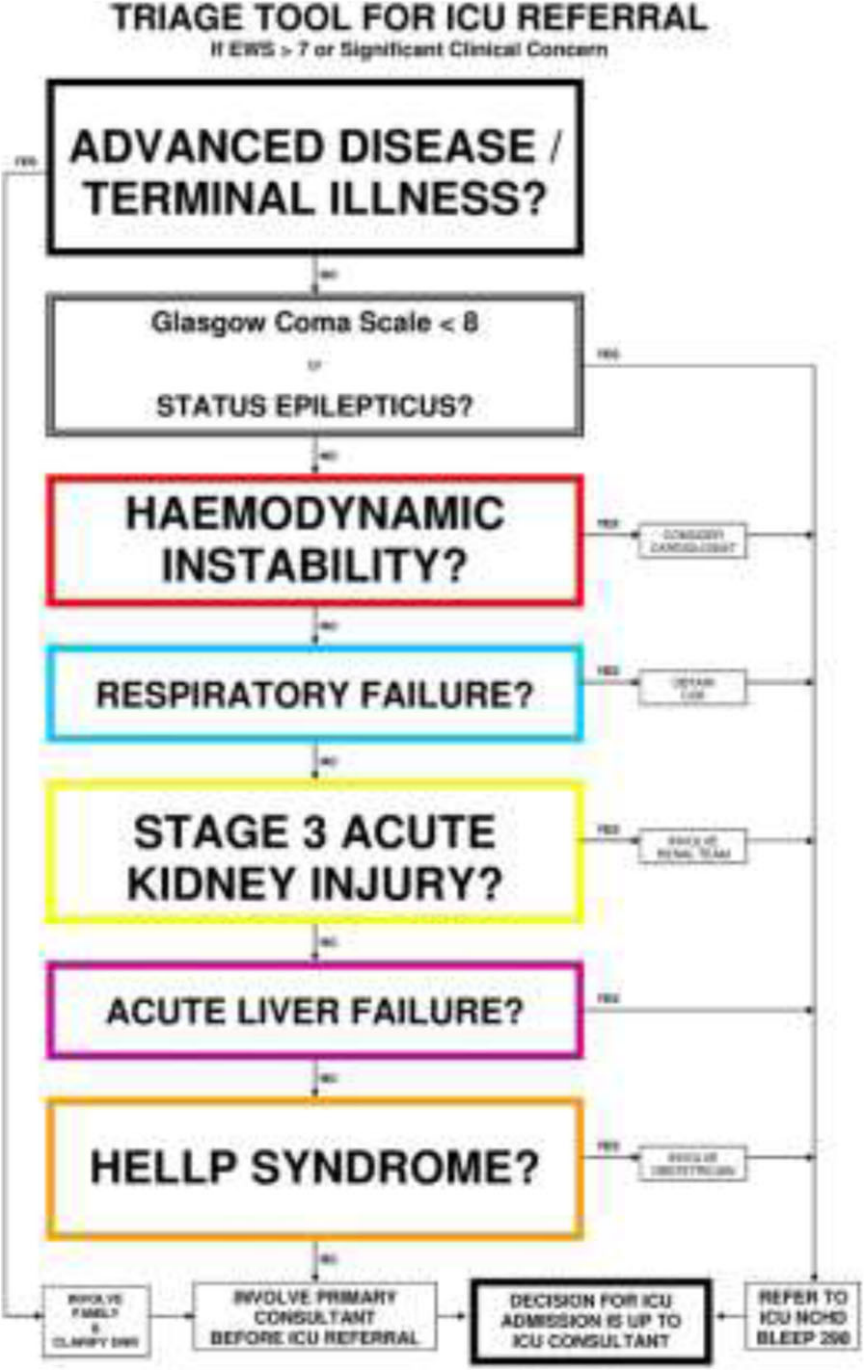
Triage tool for ICU referral

## P472 A decision-aid tool for ICU admission triage is associated with a reduction of potentially inappropriate intensive care unit admissions: an implementation study

### J Ramos^1^, O Ranzani^2^, B Perondi^2^, R Dias^2^, D Jones^3^, C Carvalho^2^, I Velasco^2^, D Forte^2^

#### ^1^Hospital Sao Rafael, Salvador, Brazil; ^2^Hospital das Clinicas, Sao Paulo, Brazil; ^3^Monash University, Melbourne, Australia

**Introduction:** Intensive care unit (ICU) admission triage occurs frequently worldwide and often involves decisions with high subjectivity, possibly leading to potentially inappropriate ICU admissions. In this study, we evaluated the effect of implementing a decision-aid tool for ICU triage on ICU admission decisions.

**Methods:** Urgent ICU referrals before (May, 2014 to November, 2014, phase 1) and after (November, 2014 to May, 2015, phase 2) the implementation of a decision-aid tool were prospectively evaluated. Our primary outcome was the proportion of potentially inappropriate ICU referrals (defined as priority 4B or 5 patients, as described by the 1999 or 2016 Society of Critical Care Medicine [SCCM] guidelines) that were admitted to the ICU in 48 hours following referral. We conducted multivariate analyses to adjust for potential confounders, and evaluated the interaction between phase and triage priorities to assess for differential effects in each priority strata.

**Results:** Of 2374 urgent ICU referrals, 110 (5%), 161 (7%), 284 (13%), 726 (33%) and 928 (42%) were categorized as priorities 4B, 4A, 3, 2 and 1 (SCCM 1999) or 110 (4.6%), 115 (4.8%), 887 (37%), 169 (7%) and 928 (39%) were categorized as priorities 5, 4, 3, 2 and 1 (SCCM 2016), respectively. Overall, 1184 (54%) patients were admitted to the ICU in 48 hours following referral. The implementation of the decision-aid tool was associated with a reduction of admission of potentially inappropriate ICU referrals [adjOR (95% CI) = 0.36 (0.13-0.97), p = 0.043] (Fig. 1). There was no difference on hospital mortality for the overall cohort between phase 1 and phase 2.

**Conclusions:** The implementation of a decision-aid tool for ICU triage was associated with a reduction of potentially inappropriate ICU admissions.

**Fig. 1 (abstract P472).**
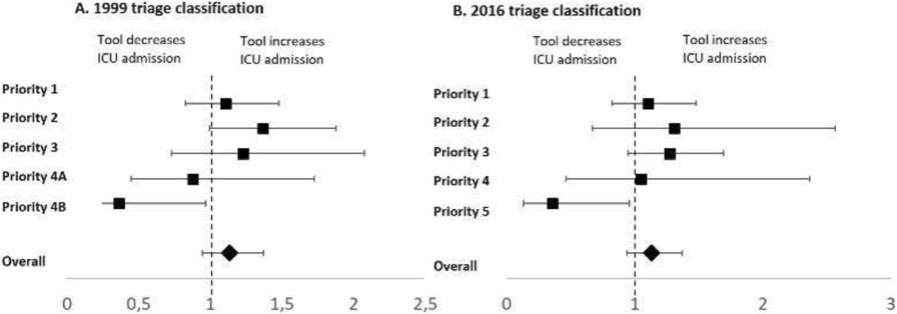
Association of the implementation of the decision-aid tool with ICU admission overall and in each priority strata, according to (A) 1999 SCCM’s triage guidelines and (B) 2016 SCCM’s triage guidelines

## P473 Scenario of an ICU after implementation of management software in a middle income country

### M Gomes da Silva Serra, A Catharina Azevedo Silva, G Silva Rocha, E Silva Oliveira, F Ferreira Ribeiro de Souza, S Pereira de Souza, L Couto de Oliveira Junior

#### Hospital Geral Cleriston Andrade, Feira de Santana Bahia, Brazil

**Introduction:** The aim was analyze the ICU bed rotation pattern, the epidemiological characteristics of patients and to correlate them with prognostic score after software implementation

**Methods:** This is an epidemiological and retrospective study. Data were collected between June 2016 and November 2017, using EPIMED® monitor software, applied in an adult ICU of a public hospital in Bahia/Brazil. Authorization for collection and use of data was granted by the institution. All patients hospitalized in the period were included regardless of other exclusion criteria.

**Results:** During the period evaluated, there were 1.011 new hospitalizations, 649 men (64.19%) and 362 women (35.81%). 46.38% (469) were in the age group of 18 to 44 years, followed by 28.28% of the patients (286), who were between 45 and 64. The mean duration of hospitalization in our unit was approximately 8,45 days. During the period covered, 1.009 exits occurred: 701 patients (69.47%) were discharged and 308 died (30.53%). The turnover rate of the ICU was 59.35. The occupancy rate calculated during the period was 101.39%. There were only 5 readmissions (0.49%) within 24 hours of admission. Regarding the hospital evolution of these patients we had 837 exits in this period, 429 (51.25%) were discharge and 408 (48.74%) were deaths, of these, 100 (11.95%) were after discharge from the ICU. The mean SAPS score was 51.11 (ranging from 17 to 99). The probability of death, according to the standard equation was 24.60% and the adjusted for Latin America of 32.10%.

**Conclusions:** The ICU has a high occupancy rate and rotation turnover, as well as a higher mortality than predicted by the score. These indicators show the great population demand that we have and alert to the impact on the sustainability of the unit and patient safety. These data allow a more informed management.

## P474 The implementation science of a critical care informatics system deployment in a central london teaching hospital

### H Rook, R West, F Master, C Bell, R Sloss, G Lakanpal, F Ahmad, S Shah, L McCaulsky, T Wheeler, K Child, T Best, R Mehta, PA Hopkins

#### King’s Critical Care, London, UK

**Introduction:** Although informatics are forming an increasingly important part of critical care delivery, the implementation science behind the introduction of such systems has not been explored [1]. Here, we describe a model for improvement through the implementation of a clinical information system (ICCA, Philips) in a large central London Critical care service (121 ICU beds). We also describe quality improvement measurement of the clinical/operational effectiveness and lessons learnt from deploying this model.

**Methods:** Research/ethics approvals were obtained. Surveys, interviews, round tables, targeted Delphi exercises and non-participant observation were conducted across four adult critical care units, involving 860 professionals. These methods were used to describe the baseline ‘paper-based’ workflow/inter-professional communication systems; and semi-quantitative quality improvement measures. Secondly, 10 critical care services worldwide were visited to generate a database of experience, lessons and models of optimised informatics delivery.

**Results:** Key challenges at baseline in relation to workflow/communication information transfer between different healthcare professionals; the adverse impact of operational strain (occupancy, high patient turnover, fluctuating patient casemix, high staff turnover); transfer of care (key meta data; current medications and medicines reconciliation); and heterogeneity of practice. Site visits revealed the importance of human resources; lead time technology advances; the prioritisation of nursing workflow and pharmacy medicines/prescribing database creation/testing and the importance of the hardware interface and ergonomics. Improvements included patient safety/experience, increased staff time and improved quality of communication/information transfer.

**Conclusions:** We have described an effective model for improvement using informatics in one of the largest critical care services in the world.

**References**

1. Varon J et al. Curr Opin Crit Care 8(6):616-24, 2002

## P475 Work-related stress amongst doctors and nurses in intensive care, A&E, acute medicine, anaesthetics and surgery

### I Lever^1^*, H Nawimana^1^*, N G^1^*, M Haider^1^,* A Molokhia ^2^

#### ^1^King’s College London, London, UK; ^2^University Hospital Lewisham, London, UK.

**Introduction:** Work-related stress is associated with anxiety, depression, days off-work, errors and ‘near misses’ [1]. Our objective was to assess stress levels and causes of stress among doctors and nurses at University Hospital Lewisham and Queen Elizabeth Hospital Woolwich. We surveyed staff using UK Health and Safety Executive’s Management Standards (HSEMS), a 35-question validated tool which identifies stressful work conditions requiring intervention.

**Methods:** We conducted an anonymous survey of doctors and nurses working in Intensive Care, Accident and Emergency (A&E), Acute Medicine, Anaesthetics and Surgery over six weeks. Results were analysed using the HSEMS Analysis Tool and broken down into seven areas: job demands, managers’ support, peer support, relationships, role, level of control and possibility of change. Each area was scored from 1-5 (5 represents lowest stress). We compared the Trust’s results against national standards.

**Results:** 283 healthcare professionals completed the survey. Intensive Care had the lowest stress levels and scored above average in all areas (n=55, mean 3.80, S.D. 0.39). This was followed by A&E (n=90, mean 3.63, S.D. 0.45), Anaesthetics (n=57, mean 3.58, S.D. 0.54), Surgery (n=42, mean 3.33, S.D. 0.47) and Acute Medicine (n=39, mean 3.25, S.D. 0.53) which had the highest stress levels. When compared to HSEMS targets peer support exceeded national standards. However, there is a clear need for improvement in staff’s ability to control and change their working environment.

**Conclusions:** Stress Levels on Intensive Care were reassuringly low when compared to other departments as well as national standards. We identified areas that need improvement and with the support of hospital management we will initiate HSEMS-validated measures to reduce stress.

**References**

1. Kerr et al, Occup Med 59:574-579, 2009

## P476 Introduction of a structured debrief on intensive care: a quality improvement initiative to optimise learning and improve practice

### L Zucco, H Damirji

#### University Hospital Lewisham, London, UK

**Introduction:** Early debriefing after stressful events holds great value in reflection on both an individual and team-based level. Our objective was to implement routine structured debriefing sessions for doctors working in intensive care in order to optimise learning and develop strategies to improve practice.

**Methods:** 100% of junior doctors (n=10, pre-implementation questionnaire) on the intensive care unit expressed a need for regular debriefing sessions to discuss challenging and complex cases. Weekly sessions were implemented and structured using the SHARP performance tool [1]. Key learning points were collected and added to a debrief list to track progress and assimilate learning. Informal feedback was obtained on a weekly basis with formal feedback assessed following one month of implementation.

**Results:** 30min sessions occurred on a weekly basis supported by a consultant intensivist. Desired outcomes included assessment of team performance, identification of key learning points and psychological support. Following one month, 100% doctors involved felt that debriefing sessions were important and should continue. 75% felt that they left every session with a key learning point applicable to future clinical practice. Common themes in perceived benefits included improved team communication and creation of an open environment to address concerns.

**Conclusions:** Working in intensive care exposes doctors to challenging and stressful situations. Implementation of a regular structured debrief session provides an opportunity for clinicians to address concerns, consolidate learning and develop strategies to improve clinical practice.

**References**

1. Ahmed M, et al. Ann Surg 258(6):958-63, 2013

## P477 Nurse staffing patterns, outcomes and efficiency in resource use in the context of icus with a “low-intensity” nurse staffing: a multicenter study in brazilian icus

### M Soares ^1^, FG Zampieri^2^, WN Viana^3^, JA Carvalho Jr^4^, R Costa^5^, TD Correa^6^, FA Bozza^1^, JI Salluh^1^

#### ^1^DOr Institute for Research and Education, Rio de Janeiro, Brazil; ^2^HCor, São Paulo, Brazil; ^3^Hospital Copa DOr, Rio de Janeiro, Brazil; ^4^Rede DOr São Luiz Itaim, São Paulo, Brazil; ^5^Hospital Quinta DOr, Rio de Janeiro, Brazil; ^6^Hospital Israelita Albert Einstein, São Paulo, Brazil

**Introduction:** Studies investigating nurse staffing and outcomes were often conducted in high-income countries with low bed/nurse ratios. Our objective was to investigate the association between nurse staffing patterns, outcomes and resource use in Brazilian ICUs.

**Methods:** Retrospective cohort study in 129,680 (68% medical) patients admitted to 93 medical-surgical ICUs during 2014-15. We retrieved patients’ data from an ICU registry (Epimed Monitor System) and surveyed participating ICUs about characteristics related to ICU organization. We used multilevel logistic regression analysis to identify factors associated with hospital mortality. We evaluated efficiency in resource use using standardized mortality rates (SMR) and resource use (SRU) based on SAPS 3.

**Results:** SAPS 3 score was 44 (34-54) points and hospital mortality was 18.2%. Intensivists were present 24/7 in 83% ICUs. Median bed/nurse ratio was 5.8 (4.2-7.3) and at least the chief nurse was board-certified in critical care (BCCC) in 47% ICUs. Bed/nurse technicians ratio was 1.9 (1.8-2.1). Adjusting for relevant characteristics at patient-level (age, admission type, SOFA, performance status, comorbidities, hospital days before ICU) and ICU-level (hospital type, checklist use, 24/7 intensivist, protocols), bed/nurse ratio was not associated with mortality [OR=0.99 (95% CI, 0.95-1.03)]. However, mortality was lower in ICUs with at least the chief nurse BCCC [OR=0.78 (0.65-0.74)]. In multivariate analysis, bed/nurse ratios <=6 [OR=3.53 (1.19-10.53)] and having the chief nurse BCCC [OR=6.36 (2.13-19.02)] were associated with higher efficiency.

**Conclusions:** In a “low intensity” nurse staffing scenario, bed/nurse ratios were not associated with mortality. However, having at least the nurse chief BCCC was associated with higher survival. Moreover, bed/nurse ratios <=6 and presence of chief nurse BCCC were associated with higher efficiency in resource use.

**Funding:**

Funded by IDOR, CNPq and FAPERJ. Endorsed by BRICNet.

## P478 Impact of nurse practitioners and physician assistants in the intensive care unit: a systematic review and meta-analysis.

### H Kreeftenberg^1^, S Pouwels^1^, A De Bie Dekker^1^, A Bindels^1^, P Van der Voort^2^

#### ^1^Catharina Hospital, Eindhoven, Netherlands; ^2^Tilburg University, Tilburg, Netherlands

**Introduction:** To gain insight in the place and additional value of the acute non-physician provider on the Intensive Care Unit (ICU).

**Methods:** A systematic search on the value of acute non-physician provider on the ICU was conducted. The methodological quality of the included studies was rated using the Newcastle Ottawa scale (NOS). The agreement between the reviewers was assessed with Cohen’s kappa.

**Results:** In total 145 studies were identified. Twenty comparative cohort studies were identified which compared non-physicians with either residents or fellows. All studies comprised adult intensive care. Most of the included studies were moderate to good quality. A random effects meta-analysis from all studies regarding length of stay and mortality showed no differences between non-physicians and physicians, although there was a trend to better survival when implementing acute non-physician providers in the ICU (Figs. 1 & 2). Mean difference for length of stay on the ICU was 0.36 (95% CI -0.07 – 0.79; I2=88%) and for in hospital -0.15 (95% CI = -0.90 – 0.61; I2=83%); while the odds ratio for ICU mortality was 0.94 (95% CI = 0.73 – 1.20; I2=60%) and for hospital mortality 0.94 (95% CI 0.89 – 1.00; I2=0).

**Conclusions:** The acute care non-physician provider in the ICU seems a promising clinician on the ICU with regard to quality and continuity of care. Whether they also can reduce mortality remains to be determined by designing studies, which adequately measure the contribution of the non-physician providers in ICU care overall and per task. Their role in Europe remains to be elucidated.

**Fig. 1 (abstract P478).**
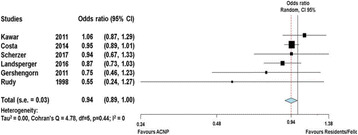
Forest plot demonstrating in hospital mortality between ICUs with NP/PA and residents

**Fig. 2 (abstract P478).**
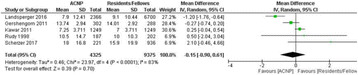
Forest plot demonstrating comparison in hospital length of stay between ICUs with NP/PA and with residents

## P479 Burnout and depression in ICU staff members

### N Bahgat

#### Menoufia University hospital, Shibin Elkom, Egypt

**Introduction:** Family and success in work are the most important sources of person satisfaction in life, Chronic prolonged exposure to stressful high workload in intensive care units (ICU), create a bad psychological state named burnout syndrome in which person is depressed, exhausted and thinks to leave job. In this study we made a survey on ICUs staff members in Egypt Menoufia university hospital to explore and find risk factors increase depression and burnout among nurses and doctor.

**Methods:** Questionnaires were given to all intensive care staff for estimating the prevalence and associated risk factors of burnout using Maslach Burnout Inventory (MBI) with its three subscales emotional exhaustion (EE), lack of accomplishment (LA), and depersonalization (DP). Depressive symptoms using the Beck Depression Inventory Scale. Blood sample was taken for assessing depression biomarkers including IL-6, tumor necrosis factor (TNF)-alpha, and coenzyme Q10 (CoQ10), which appears to be one of the most reliable peripheral biomarkers.

**Results:** 100 participants were respond in our survey from 127 ICU members the response rate was 78.7%, The depression symptoms found increased in nurses more than physicians in ICU with more desire to leave the job. There was strong correlation between the degree of depression symptoms and decrease percent of personal accomplishment. Impaired personal relationships at work and increased night shifts were major risk factors of burnout syndrome.

Levels of the proinflammatory cytokine (IL6 and TNF alpha) were elevated in members who recorded sever degree of depression score with decrease in concentration of Co-enzyme Q10.

**Conclusions:** The health workers in ICU had high liability for depression and burnout syndrome. The risk factors differ between nurses and doctors. IL6, co-enzyme Q10 and TNF alpha concentrations had god correlation with degree of severity of symptoms.

## P480 Impact of a tailored multicomponent program to reduce discomfort in the ICU on post-traumatic stress disorder: a case-control study

### P Kalfon ^1^, M Alessandrini^2^, M Boucekine^2^, M Geantot^3^, S Renoult^4^, S Deparis-Dusautois^5^, O Mimoz^6^, J Amour^7^, E Azoulay^8^, C Martin^9^, T Sharshar^10^, M Garrouste-Orgeas^11^, K Baumstarck^2^, P Auquier^2^

#### ^1^Centre Hospitalier de Chartres, Chartres, France; ^2^Aix Marseille Université, Marseille, France; ^3^CHU Dijon Bourgogne, Dijon, France; ^4^Clinique Ambroise Paré, Neuilly/Seine, France; ^5^Centre Hospitalier de Troyes, Troyes, France; ^6^CHU La Milétrie, Poitiers, France; ^7^CHU Pitié Salpêtrière, Assistance Publique - Hôpitaux de Paris, Paris, France; ^8^CHU Saint-Louis, Assistance publique - Hôpitaux de Paris, Paris, France; ^9^CHU H Nord, Assistance publique - Hôpitaux de Marseille, Marseille, France; ^10^CHU Raymond Poincaré, Assistance publique - Hôpitaux de Paris, Garches, France; ^11^Groupe Hospitalier Paris Saint-Joseph, Paris, France

**Introduction:** Reducing discomfort during the ICU stay should be beneficial on long-term outcomes. The aim of this study was to assess the impact of the implementation of a tailored multicomponent program to reduce discomfort in the ICU [1] on the occurrence of posttraumatic stress disorder (PTSD) 12 months after discharge from the ICU.

**Methods:** Design: case-control study; the cases were patients hospitalized in the ICUs which implemented the tailored multicomponent program; the controls were patients hospitalized in the ICUs which did not implement the program. Exposition: the tailored multicomponent program consisted of assessment of ICU-related self-perceived discomforts by using the IPREA questionnaire, immediate and monthly feedback to healthcare teams, and tailored site-targeted measures under control of a duo of local champions. General procedure: eligible patients were recalled 12 months after the ICU stay. Data collection: sociodemographics, clinical data related to the ICU stay, discomfort’s levels assessed the day of discharge from the ICU, life situation (home/care center), PSTD (IES-R) and anxiety-depression symptoms (HADS) 12 months after the ICU discharge.

**Results:** From the 617 eligible cases and 847 eligible controls, 344 cases and 475 controls were included (reason for exclusion: deaths after discharge from the ICU, lost to follow-up, patient refusal, cognitive incapacity). A total of 6.1% of the cases and 12.2% of the controls presented certain symptoms of PTSD at12 months (p=0.004). After adjustment for age, gender, IPREA score, McCabe score, presence of invasive devices during the ICU stay and considering anxiety-depression symptoms at 12 months, cases are less likely to have PTSD symptoms than controls.

**Conclusions:** Our tailored multicomponent program for discomfort reduction in the ICU can reduce long-term outcomes as PTSD. Diffusion of such a program should be enhanced in the ICUs paving the way for a new strategy in care management.

**References**

1. Kalfon P et al, Intensive Care Med 43(12):1829-1840, 2017

## P481 Pre-existing cognitive dysfunction in critically ill patients and the incidence of delirium during ICU treatment.

### ST Könning, D Ramnarain

#### Elisabeth Tweesteden Hospital Tilburg (ETZ), Tilburg, Netherlands

**Introduction:** Cognitive dysfunction is a major factor leading to disability and poor quality of life in ICU survivors. In order to identify patients at risk for developing cognitive dysfunction due to critical illness or ICU treatment, one has to discriminate between patients with pre-existing cognitive dysfunction and those developing new cognitive dysfunction or worsening of cognitive function during ICU treatment. We investigated the incidence of pre-existing cognitive dysfunction in ICU patients using the Informant Questionnaire on Cognitive Decline in the Elderly (IQCODE) and its relation with delirium during ICU treatment.

**Methods:** Patients relatives were asked to fill in the IQCODE on admission. An overall score on cognitive dysfunction was calculated by the average of the score on each item of the questionnaire. The incidence of delirium was based on the CAM-ICU score. Statistical analysis was performed using the Fisher’s exact test. P-values of less then 0.05 were deemed significant.

**Results:** In total 452 consecutive patients admitted to our ICU were analyzed, of whom 47.8% (n=216) showed decline in cognitive function prior to ICU admission. Cognitive function was divided in four groups; no change 52.2% (n=236), slight decline 34.1% (n=154), moderate decline 9.7% (n=44) and severe decline 4.0% (n=18) (Fig. 1). Incidence of delirium is shown in Fig. 2. Patients with moderate to severe cognitive dysfunction showed significant more delirium during ICU treatment than patients with no change in cognition (44.2% and 21.1% respectively, (p=0.023)).

**Conclusions:** Almost half of the patients admitted to the ICU have cognitive dysfunction prior to ICU admission. To assess ones cognitive function after ICU treatment one has to take in to account the patients pre-existing cognitive functioning. Patients with a moderate to severe pre-existing cognitive dysfunction develop significantly more delirium during ICU treatment.

**Fig. 1 (abstract P481).**
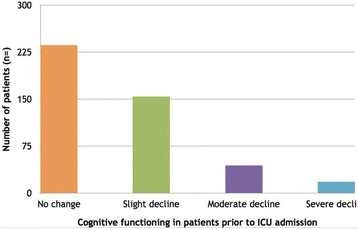
Cognitive functioning in patients prior to ICU admission.

**Fig. 2 (abstract P481).**
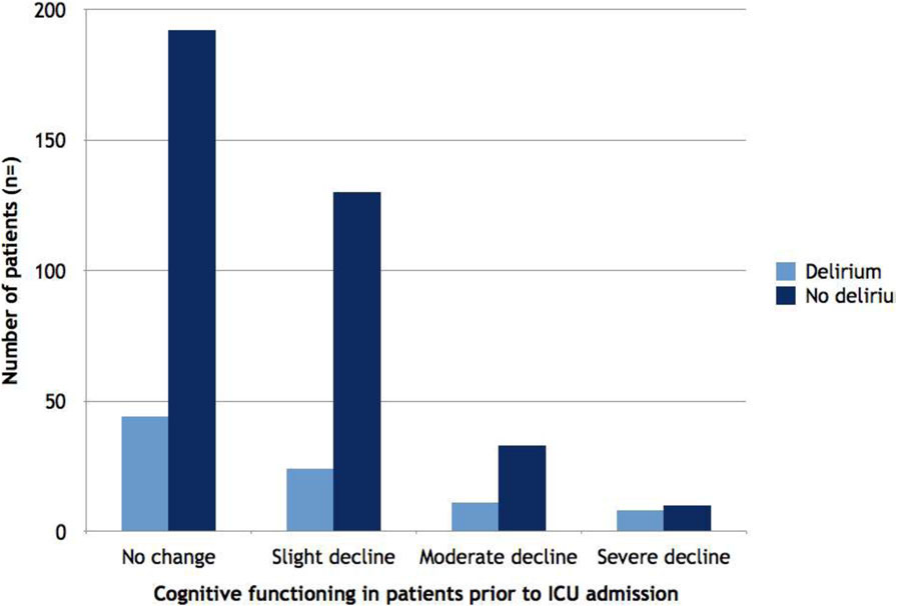
Patients with delirium vs no delirium in different groups of cognitive functioning.

## P482 Postoperative cognitive dysfunction (pocd) in high risk surgical patients. a retrospective study.

### A Hunter, M Wells, G Gunaratnam, I Ibrahim, D Walker, C Gore

#### Department of Critical Care, University College London Hospital, London, UK

**Introduction:** Our aim was to identify and analyse patients treated for POCD admitted to a thoracics/urology intensive care unit at University College London, UK. POCD is rising in the ageing high-risk surgical patient. Early identification of those at risk and timely intervention could help reduce associated morbidity and mortality [1].

**Methods:** We identified patients treated with haloperidol, midazolam, lorazepam, olanzapine, clonidine or chlordiazepoxide from our electronic data system. These pharmacological interventions were used as surrogate markers of primarily hyperactive POCD, acknowledging other forms of delirium may be unaccounted for. 111 of 808 admissions (13.7%) were shortlisted from August 2016 to July 2017. Patients were excluded if the drugs had been used for other indications. Prevalence of known POCD risk factors were then detailed. On these data we performed a cluster analysis using R.

**Results:** Of the 58 patients (7.17%) suitable for analysis, the mean age was 72. 41 patients underwent elective procedures. 39 were male and 19 were female. 75% patients had thoracic surgery. The mean pain score in the first 24 hours post-op was 1.6 (SD=1.1), (with 0= no pain, 4= very severe pain). 62% had evidence of poor sleep and 14% evidence of anxiety. In the 24 hours prior to evidence of POCD, the mean pain score remained 1.6 (SD=0.99), 76% had evidence of poor sleep and 22% had evidence of anxiety. 66% of our population was septic during their ITU admission.

**Conclusions:** Our analysis demonstrates POCD is highly prevalent in male patients over 70 undergoing thoracic procedures. We will now develop a POCD pathway targeting improved postoperative management of pain, sleep, anxiety and infection in this patient population.

**References**

1. Rudolph JL, Marcantonio ER. Anesth Analg 12:1202-1211, 2001

## P483 Validation of the SOS-PD scale for assessment of pediatric delirium: a multicenter study

### E Ista^1^, B Van Beusekom^1^, J Van Rosmalen^1^, MCJ Kneyber^2^, J Lemson^3^, A Brouwer^4^, G Dieleman^1^, B Dierckx^1^, M De Hoog^1^, D Tibboel^1^, M Van Dijk^1^

#### ^1^Erasmus MC - Sophia Children’s Hospital, Rotterdam, Netherlands; ^2^UMC Groningen - Beatrix Children’s Hospital, Groningen, Netherlands; ^3^Radboud University Medical Center, Nijmegen, Netherlands; ^4^Maastricht University Medical Centre, Maastricht, Netherlands

**Introduction:** Delirium in critically ill children has gained attention in the last few years and the incidence seems higher than anticipated before. The Sophia Observation withdrawal Symptoms-Pediatric Delirium (SOS-PD) was developed to combine assessment of delirium with iatrogenic withdrawal syndrome, two conditions with overlapping symptoms. The current study evaluates the measurement properties of the PD component (PD-scale) of the SOS-PD scale.

**Methods:** In a multicenter prospective observational study in four Dutch PICUs, patients aged 3 months to 17 years and admitted for more than 48 hours were included. These patients were assessed with the PD-scale three times a day. Criterion validity was established: if the PD total score was 4 or higher the child psychiatrist was consulted to confirm the diagnosis of PD using the Diagnostic and Statistical Manual-IV criteria as the “gold standard”. The child psychiatrist was blinded to outcomes of the PD-scale. In addition, the child psychiatrist assessed a randomly selected group of patients to establish false-negatives. The interrater reliability of the PD-scale between the care-giving nurse and a researcher was calculated with the intraclass correlation coefficient (ICC).

**Results:** Four hundred eighty-five patients with a median age of 27.0 months (IQR 8-102) were included. The pediatric delirium scale had an overall sensitivity of 92.2% and a specificity of 96.9% for a cut off score of 4 points. The positive predictive and the negative predictive value were respectively, 76.3% and 99.1%. The ICC of 75 paired nurse-researcher observations was 0.99 (95% CI 0.98-0.99). In total 48 patients were diagnosed with delirium during the PICU stay.

**Conclusions:** The PD scale shows a good validity for early screening of PD. So, the PD scale is a valid and reliable tool for nurses to assess delirium in critically ill children.

## P484 Feasibility of employing family-administered delirium detection tools in the intensive care unit (ICU)

### K Fiest^1^, K Krewulak^1^, J Davidson^2^, EW Ely^3^, HT Stelfox^1^

#### ^1^University of Calgary, Calgary, Canada; ^2^University of California, San Diego, San Diego, USA; ^3^Vanderbilt University Medical Center, Nashville, USA

**Introduction:** Our objective was to determine the feasibility of employing family-administered tools to detect delirium in the critically ill. The use of family-administered delirium detection tools has not been assessed in the ICU where patients are critically ill and frequently intubated. Family members may be able to detect changes in patient cognition and behavior from pre-illness levels earlier than unfamiliar providers. These tools may be a valuable diagnostic adjunct in the ICU.

**Methods:** Consecutive patients and family members (dyads) in the largest adult ICU in Calgary, Canada were recruited (Aug. 9-Sept. 11, 2017). Inclusion criteria were: patients with a Richmond Agitation Sedation Scale (RASS) >=-3; no primary brain injury and Glasgow Coma Scale score of <9; ability to provide informed consent (patient/surrogate); and remain in ICU for 24 hours. Data were collected for up to 5 days. Family-administered delirium assessments were completed once daily (Family Confusion Assessment Method & Sour Seven). To assess feasibility, we assessed proportion of eligible patients and percent family member enrollment. Barriers to enrollment were categorized.

**Results:** Of 99 admitted patients with family, 37 (37%) met inclusion criteria and 17 (46%) dyads consented. 20% of admitted patients did not have family and were thus ineligible. 73% of enrolled dyads assessed delirium at least once, with a median of 5 (of 10 total) assessments. The most common reason for non-enrollment was refusal by the family, who commonly reported feeling overwhelmed by the ICU environment. Barriers with nursing staff were encountered, including not providing access to patients and patient exclusion.

**Conclusions:** These data suggest that employing family-administered delirium detection tools in the ICU is feasible for a subset of the population. Future studies will validate the use of these tools in the ICU, decrease modifiable barriers to enrollment, and test strategies to overcome attitudinal barriers towards employing these tools.

## P485 Acute psychological distress in awake ICU patients: an observational study

### A Franci, L Cecci, L Tadini Buoninsegni, M Bonizzoli, J Parodo, A Ottaviano, A Peris

#### Azienda Ospedaliero Universitaria Careggi, Firenze, Italy

**Introduction:** Psychological impact of critical illness and ICU stay on patients can be severe and frequently results in acute distress as well as psychological morbidity after discharge [1]. However, the stressful experience in ICU and its influence on patient recovery, remain relatively understudied. We assessed patients in ICU for acute distress and psychological symptoms with validated tools.

**Methods:** We conducted an observational study in a group of awake ICU adult patients admitted in a tertiary centre for at least 48 hours, from January 2017 until October 2017, with mixed diagnosis on admission. We collected demographic factors, SAPS II at admission, mechanical ventilation, day of sedation, history of psychopathological disorder. Un-sedated and alert, critical care patients were assessed with tools such as Intensive Care Delirium Screening Checklist (ICDSC), Hospital Anxiety and Depression Scale (HADS) and Intensive Care Psychological Assessment Tool (IPAT).

**Results:** 68 patients were recruited, (mean age 51.2±17.9 years, 66.2% males). SAPS II at admission was 32.2±16.7, 60.3% was mechanically ventilated (mean duration 6.1±14), mean duration of sedation was 2.8±3.9 days and a rate of 22.05% had an history of psychopatological disorder. 10.3% of the sample had clinical delirium (ICSDC>3) and was not assessed with others tools, 20.6% had subclinical delirium (ICSDC <=3). Regarding psychological outcomes, 26.2 % (mean score 6.1±2.5) reported a score (>=8) on HADS that indicates a possible diagnosis of anxiety and 54.1% (mean score 7.9 ± 3.7) of depression. A rate of 24.6% reported a score >= 7 on IPAT suggesting an acute distress.

**Conclusions:** The study’s key finding was that acute psychological distress was high in awake ICU patients. Further work is needed to determine the efficacy of early psychological interventions to reduce the incidence of acute distress and psychological outcomes after ICU stay.

**References**

1. Wade et al. Crit Care 16:R192, 2012

## P486 Withdrawn

## P487 Withdrawn

## P488 Effect of acetaminophen on sublingual microcirculation in febrile septic patients: preliminary analysis

### U Falanga, R Domizi, E Casarotta, A Carsetti, S Tondi, A Donati

#### Università Politecnica delle Marche, Ancona, Italy

**Introduction:** In septic patients, increased plasma levels of cell-free hemoglobin (free-Hb) are associated with a reduction of perfused vessel density (PVD) of sublingual microcirculation and to adverse outcomes caused by hemoprotein-mediated lipid peroxidation. Recent studies show that acetaminophen protects from damage due to lipid peroxidation in sepsis [1]. The aim of this study is to detect changes in sublingual microcirculation after the infusion of a standard dose of acetaminophen in febrile septic patients.

**Methods:** Prospective observational study on 50 adult septic patients admitted to our Intensive Care Unit. Pre-infusion (t0), 30 minutes (t1) and 2 hours (t2) after the end of the infusion of acetaminophen, sublingual microcirculation was assessed with Incident Dark Field Illumination imaging; vital signs, plasma levels of acetaminophen and free-Hb were assessed.

**Results:** Preliminary descriptive analysis on the first 7 patients shows a median Sequential Organ Failure Assessment (SOFA) score of 8 (interquartile range IQR 7-11) and baseline temperature of 38,6 C° (IQR 38.1-39°C). An increase of the proportion of perfused vessels (PPV) was evident both at t1 and t2 (95.6% [91.13%-97.43%] at t0, 98.6% [96.84%-100%] at t2) (Fig. 1). No substantial increase of PVD was evident, but a trend towards improvement in median values at t2 (20.58 [18.00-33.44] at t0, 23.97 [16.97-30.87] at t2).

**Conclusions:** Definitive data are needed to understand the relation between acetaminophen infusion, free-Hb lipid peroxidation and microcirculatory dysfunction in sepsis, but preliminary results are encouraging.

**References**

1. Janz DR et al. Crit Care Med 43, 534–541, 2015

**Fig. 1 (abstract P488).**
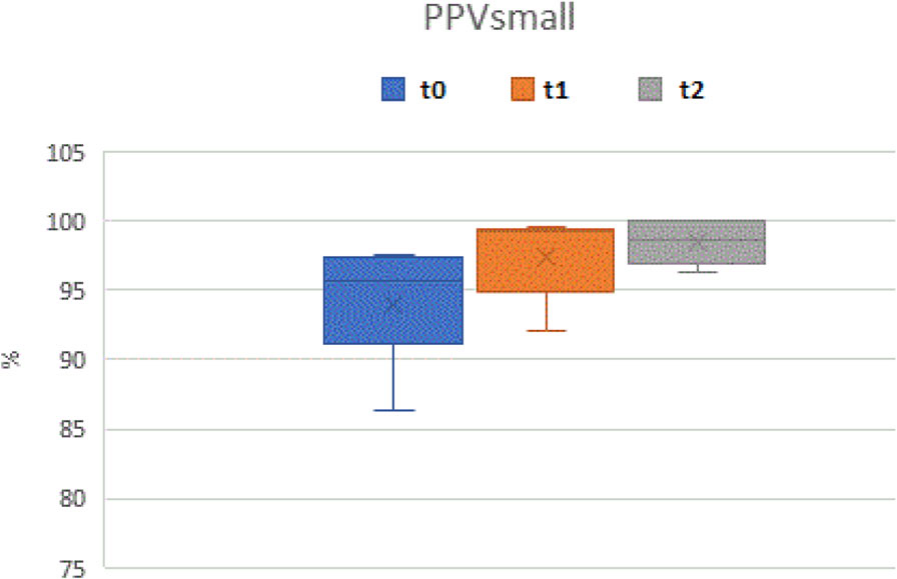
Proportion of Perfused small Vessels at to, t1, t2

## P489 Frequency, risk factors and symptomatology of iatrogenic withdrawal from opioids and benzodiazepines in critically ill neonates, children and adults: a systematic review of clinical trials

### MA Duceppe^1^, M Perreault^1^, AJ Frenette^2^, L Burry^3^, P Rico^2^, A Lavoie^4^, C Gélinas^5^, S Mehta^3^, M Dagenais^1^, D Williamson ^2^

#### ^1^McGill University Health Centre, Montreal, Canada; ^2^Hôpital du Sacré-Coeur de Montréal, Montreal, Canada; ^3^Mount Sinai Hospital, Toronto, Canada; ^4^CHU Ste-Justine, Montreal, Canada; ^5^Jewish General Hospital, Montreal, Canada

**Introduction:** Limited recommendations exist regarding evaluation, prevention and treatment of withdrawal syndromes in both pediatric and adult ICU populations. Given the potential for complications and its iatrogenic nature, defining the syndrome and identifying patients at risk is important. We conducted a systematic review to examine the frequency of withdrawal, risk factors and symptoms of iatrogenic withdrawal in critically ill pediatric and adult patients who received benzodiazepines and/or opioids during their ICU stay.

**Methods:** The literature search was conducted in Pubmed, Medline, EMBASE, Cochrane Central Register of Controlled Trials, Cochrane register of systematic reviews, DARE, CINAHL, Trip database, CMA infobase and NICE evidence from inception to October 2017. We also examined the grey literature. We included studies reporting frequency, risk factors or symptomatology of iatrogenic withdrawal of opioids, benzodiazepines (or both) in critically ill patients. We considered all study designs except case reports and case series. Pairs of reviewers independently abstracted data and evaluated methodological quality using the Cochrane collaboration tool, Newcastle-Ottawa or QUADAS-2. PROSPERO (registration number: CRD42016042746).

**Results:** We identified 21210 unique citations through database search and 146 full-texts were assessed for eligibility. Thirty-three studies were included; the majority were observational and only a few included adults. In prospective studies, mixed withdrawal was observed in 16.7% of adults and ranged from 7.5% to 100% in pediatric studies. Symptoms of withdrawal were not well described. Risk factors included higher cumulative dose and prolonged administration of opioids and benzodiazepines.

**Conclusions:** Iatrogenic withdrawal appears to be a frequent syndrome in critical care patients who received infusions of opioids and/or benzodiazepines. Larger studies are required, especially in critically ill adults, to better define the syndrome and its symptomatology.

## P490 Entropy–guided depth of hypnosis on general anesthesia in critically ill polytrauma patients

### O Bedreag, A Rogobete, C Cradigati, M Sarandan, S Popovici, D Sandesc

#### Victor Babes University of Medicine and Pharmacy, Timisoara, Romania

**Introduction:** A high percentage of polytrauma patients require surgery within the first 24 hours to stabilize primary traumatic injuries. One of the main intraoperative complications in this type of patients is due to hemodynamic instability [1]. Thus, it is necessary to implement multimodal monitoring involving both hemodynamic monitoring and monitoring of general anesthesia. The objectives of this study were to identify the possible implications of Entropy monitoring on hemodynamic stability in critically ill polytrauma patients.

**Methods:** Prospective Observational Study, Deployed in the Clinic of Anesthesia and Intensive Care, Emergency County Hospital "Pius Brinzeu" Timisoara, Romania. ClinicalTrials.Gov Identifier. There were two groups, Group A (N = 37), in which the depth of hypnosis was monitored through Entropy (GE Healthcare, Helsinki, Finland) and Group B (N = 35).

**Results:** The incidence of hypotension and tachycardia episodes was statistically significantly lower in Group A, unlike the control group (p <0.05). Moreover, a statistically significant (p <0.05) consumption of inhaled anesthetic agent was recorded in Group A compared with Group B. Consumption of vasopressor was also lower in Group A (p <0.0001, difference between means 0.960 ± 0.063, 95% confidence interval 0.8334 - 1.0866)

**Conclusions:** Deploying monitoring for the depth of hypnosis in general anesthesia using Entropy can significantly increase the hemodynamic stability of critically ill polytrauma patients.

**References**

1. Vincent JL et al. Crit Care 15:196, 2011

## P491 Use of methadone in critically ill patients

### R Vale, P Travasos, N Postalli, T Alvarisa, M Pedroso, V Veiga, S Rojas

#### Hospital BP - A Beneficência Portuguêsa de São Paulo, São Paulo, Brazil

**Introduction:** The use of methadone as a potent analgesic has been gaining ground in the intensive care setting, such as where it is possible to properly select the group of patients who will benefit from the drug, as well as monitoring of possible complications. The objective of this study is to evaluate the safety of the use of methadone in critically ill patients in a large hospital.

**Methods:** A retrospective analysis of all patients who used methadone in a neurological intensive care unit for a period of four months and the results were evaluated.

**Results:** In the four-month period, 52 patients used methadone during intensive care. 65 % of the patients were male, with a medical age of 59.7 ± 17.4 years. The main indication for the use of the medication was for analgesia in patients who were weaned from mechanical ventilation. The mean time of use was 6.1 days. In all cases evaluated, analgesia was effective, with methadone being used alone or in combination with other drugs, according to an institutional protocol. Among the complications found, 20 patients presented hypotension (38 %); 20 presented bradycardia (38 %); 15 presented constipation (29%); 4 had excessive sedation (8 %) and 7 had other complications. All complications were reversible. 10 patients of the studied population died, however, without correlation with the use of methadone.

**Conclusions:** The use of methadone, in the studied group, was effective in the control of analgesia, with no impact on patient safety when used in a monitored way.

## P492 Comparison the efficacy of intravenous ketorolac versus morphine in the treatment of renal colic

### R Mosaddegh, M Rezai, F Jalili, H Hadizadeh, M Salehi Namin

#### Emergency Medicine Management Research Center, Iran University of Medical Sciences, Tehran, Iran

**Introduction:** Renal colic is a common disorder which presents with dramatic acute pain. Providing rapid relief, using effective pain control medications is the clinical priority to treat the patients. This study aims to compare the effect of IV Ketorolac versus Morphine in releasing renal colic pain by measuring pain severity and duration and also the need for additional doses.

**Methods:** We performed a clinical pilot cohort study from during 2014 on patients with the clinical diagnosis of renal colic who recruited from the emergency department (ED) of Rasool-e-Akram Hospital and Firoozgar Hospital. Participants who were candidate to receive either Morphine or Ketorolac were divided into two groups who received either 30 mg Ketorolac IV or 5 mg Morphine. The pain was evaluated using the visual analog scale (VAS) at four time points: before drug injection (VAS-1), 20 minutes (VAS-2), 40 minutes (VAS-3), and 60 minutes (VAS-4) after injection. In cases when the pain was not controlled with the first injection of drug beyond 60 minutes; additional doses (rescue) were injected. Statistical analyses were performed using SPSS 21.

**Results:** One-hundred-fifty patients treated with Morphine and 150 ones with Ketorolac were studiedThe group treated with Morphine scored on average 9.91 before the injection, which was roughly 2.4 points higher than Ketorolac. Morphine reduced patients’ VAS scores more intensely (median: 10, IQR: 0 versus median: 6, IQR: 1; p value<0.001). In general, patients treated with Morphine were more likely to need a second (rescue) dose, when compared to Ketorolac group (38.6% vs 20%, p value= 0.001).

**Conclusions:** Morphine is a better option for pain release in cases of renal colic. Ketorolac released the pain to an acceptable level; but, because of its slower action time, we recommend it in cases with moderate than severe pains.

## P493 Effect of analgesics on cardiovascular and hormonal response to operative trauma

### D Loncar Stojiljkovic, MP Stojiljkovic

#### SGH, 11000, Serbia

**Introduction:** Objective of this study was to compare the effects of two analgesic regimens, one opioid and one non-opioid, on cardiovascular and hormonal reaction of patients undergoing elective surgery under general endotracheal anaesthesia.

**Methods:** A total of 40 elderly patients, ASA 2, scheduled for elective abdominal surgery were assigned to receive on induction a single dose of either fentanyl (0.2 mg, +0.1mg) or a fix combination of etodolac and carbamazepine (Novocomb, dose 100mg+100mg iv bolus). Haemodynamic parameters and concentrations of prolactin cortisol and growth hormone (GH) [1] were determined at critical points and 24 h after operation.

**Results:** Both fentanyl and Novocomb blocked the hypertensive-tachycardic response to surgical trauma. Cortisol was a more appropriate endocrine marker of stress than prolactin or GH since fentanyl as an opioid analgesic increased secretion of prolactin [2], while carbamazepine from Novocomb did the same with GH [3] (Figs. 1 & 2).

**Conclusions:** Cortisol plasma concentration correlates positively with cardiovascular parameters in patients undergoing elective abdominal surgery who received fentanyl or Novocomb as intraoperative analgesic. Its suppression is better marker of analgesia than prolactin and GH.

**References**

1. Vuong C et al. Endocr Rev 31(1): 98–132, 2010.

2. Lafuente A et al. Vet Hum Toxicol 36(6):524-8, 1994.

3. Syvälahti E, Pynnönen S. Acta Pharmacol Toxicol (Copenh) 40(2):285-8, 1977.

**Table 1 (abstract P493).** Fentanyl

	Prolactin (μ g/L)	Cortisol (nmol/L)	Growth hormone (pmol/L)
Baseline	7±1	425±64	143±75
Intubation	28±11*	420±88	152±81
First incision	143±31*	421±95	145±64
Surg. manip.	158±27*	555±21	76±32
24 h postop.	167±25*	655±21	68±28

**Table 2 (abstract P493).** Novocomb

	Prolactin (ìg/L)	Cortisol (nmol/L)	Growth hormone (pmol/L)
Baseline	23±10	429±52	6±1
Intubation	152±28*	428±41	10±2*
First incision	193±30*	396±34	21±14*
Surg. manip.	193±30* 160±28*	606±68*	38±30*

## P494 Volatile anaesthetic consumption and recovery times after long term inhalative sedation using the mirus system - an automated delivery system for isoflurane, sevoflurane and desflurane

### AI Georgevici, L Procopiuc, D Drees, J Herzog-Niescery, P Gude, H Vogelsang, TP Weber, M Bellgardt

#### St Josefs Hospital University Clinic Bochum, Bochum, Germany

**Introduction:** The new MIRUS system as well as the AnaConDa uses a reflector to conserve volatile anaesthetics (VA) [1,2]. Both systems can be paired with ICU ventilators, but MIRUS features an automated control of end-tidal VA concentrations (etVA). We compare feasibility and recovery times for inhalational long term sedation with isoflurane (ISO), sevoflurane (SEVO) or desflurane (DES).

**Methods:** 30 ASA II-IV patients undergoing elective or emergency surgery under general anaesthesia were included. Patients were randomized into three equal groups ISO, SEVO and DES. The MIRUS system was started with a targeted etVA of 0.5 MAC. We used the Puritan Bennett 840 ICU ventilator and performed a spontaneous breathing trial. If successful, the target concentration was set to 0 MAC and recovery times measured.

**Results:** Patients were comparable in demographics, tidal volume, respiratory rate and sedation time (total 696h: ISO 19±9h; SEVO 22±19h; DES 29±29h; p=0.55). In all patients, a MAC of 0.5 was reached. ISO 4.7±1.5 ml/h, SEVO 11.7±7.3 ml/h or DES 28.9±5.5 ml/h (p<0.001) were used. Recovery times (p>0.05): Open eyes ([mm:ss]; ISO 15:48±18:05, SEVO 06:11±09:09, DES 04:48±06:36); squeeze hands ([mm:ss]; ISO 20:22±21:20, SEVO 08:22±09:42, DES 03:37±02:47); extubation ([mm:ss]; ISO 26:40±39:12, SEVO 13:27±16:20, DES 04:00±02:00); tell birthday ([mm:ss]; ISO 34:42±39:27, SEVO 14:28±18:02, DES 05:37±02:17). The first extubation attempt was successful in 7, 8 and 5 patients sedated with ISO, SEVO and DES, respectively.

**Conclusions:** MIRUS could automatically control end-tidal VA concentrations in ventilated and spontaneously breathing patients. The recovery times are only prolonged in the ISO group and could be shortened by removing the reflector. The higher etVA required for a 0.5 MAC using DES and SEVO were associated with an increased VA consumption.

**References**

1. Bomberg H et al.: Anaesthesia 69:1241–50, 2014.

2. Romagnoli S et al: Crit Care Med. 45:e925-e931, 2017.

## P495 The use of intranasal fentanyl versus parenteral opioid for acute pain relief in adults: systematic review and meta-analysis

### F Fortier-Dumais, C Chau, JM Chauny, A Cournoyer, V Huard, J Lessard

#### Hôpital Sacré-Coeur de Montréal, Montréal, Canada

**Introduction:** Intranasal analgesia is increasingly used in order to relieve pain in the emergency department. This non-invasive approach avoids discomfort, stress and risks related to the parenteral route of administration. The objective is to compare intranasal (IN) fentanyl versus any parenteral opioid (intravenous, subcutaneous, intramuscular) for the effectiveness of acute pain relief in an adult population.

**Methods:** The systematic review was registered in Prospero (CRD42016052976). The research of articles was conducted through Embase, Central, and Medline databases. Randomized clinical trials comparing the effectiveness of IN fentanyl to any parenteral opioid for acute pain relief (<= 7 days) in an adult population (>= 18 years old) were considered for inclusion. Studies on breakthrough cancer pain were excluded. Two different reviewers extracted data and analyzed the quality of the selected articles. The main outcome was the difference between pain levels before and after analgesia. The effect size was approximated using the inverse of variance of standardized mean differences, based on a random-effect model. Heterogeneity was quantified using a test of I2. Results are presented with 95% confidence interval.

**Results:** Eight randomized clinical trials with 11 cohorts and a total of 613 patients were selected (320 IN fentanyl vs 293 control group). Selected articles contained a low risk of bias. There is no significant difference between the average levels of pain before and after analgesia comparing the two groups (SMD 0.12 [IC 95% -0.04 à 0.28], p=0.14; I2 = 0%) (Fig. 1).

**Conclusions:** IN fentanyl is as effective as other parenteral opioid to relieve pain during the first hour of treatment.

**Fig. 1 (abstract P495).**
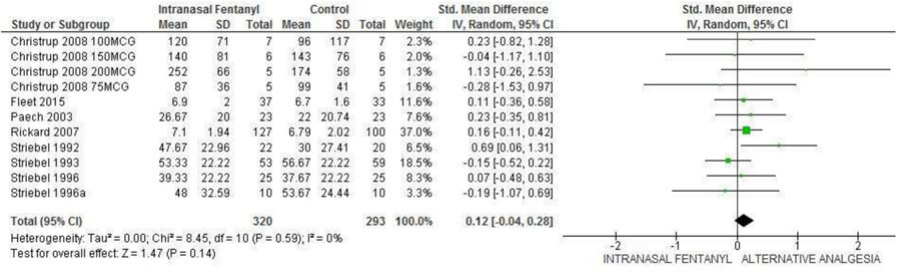
Intranasal fentanyl compared to parenteral opioid for acute pain relief

## P496 Anesthesiological management for percutaneous endoscopic gastro-jejunostomy (PEGJ) procedure in parkinson’s disease patients

### M Della Valle, L Marullo, A Iuorio, P Fusco, A Tessitore, F Ferraro

#### A.O.U "Luigi Vanvitelli", Napoli, Italy

**Introduction:** The aim of this study is to underline the importance of sedation protocol when performing the PEGJ procedure in advanced Parkinson’s Disease (PD) patients. Research about the use of sedation in endoscopy is getting more and more widespread as to answer to the increasing grade of complexity and duration of endoscopic procedures as to offer comfort to the patient in terms of analgesia, tolerability, and amnesia. Sedation is also a way to assure quality and safety examination and to improve its outcome [1].

**Methods:** This observational retrospective study includes 40 PD patients scheduled for PEGJ procedure (Fig. 1) in order to start therapy with Duodopa gel. We propose an anesthetic technique (Table 1) to support PEGJ with both local anesthesia and moderate sedation so as to provide analgesia and patient’s comfort. This technique ensures maintenance of spontaneous ventilation and a satisfactory cardiovascular function improving the examination’s outcome.

**Results:** 40 patients scheduled for PEGJ procedure for continuous levodopa infusion were included in this analysis. Mean patient age was 60±10,4.Patient demographics were: 2% male and 47% female. Mean duration of PEGJ procedure was 35’±5’. Mean stay time in recovery room 12’±2’. Compared to our old experience, we collected lack of patient’s discomfort, anxiety, and memory, high procedure compliance and improvement of the quality of procedure without use of opioids.

**Conclusions:** Based on our experience, we consider this sedation protocol effective for different reasons: to relieve or abolish patient’s discomfort, anxiety, and memory, to ensure compliance with the procedure, to ensure patient’s analgesia and patient’s safety and, finally to assure procedure’s quality and rapid discharge. Anyways, a multicentric study should be done to test our protocol.

**References**

1. Triantafillidis JK et al. World J Gastroenterol 19(4):463-81, 2013

**Table 1 (abstract P496).** Endoscopy protocol sedation

Premedication	Atropine	0.01 mg/Kg i.v.
	Midazolam	0.015-0.03 mg/kg i.v.
Induction	Propofol	0.5 - 1mg/kg i.v. (bolus)
Sedation	Propofol	2-5 mg/Kg/h continuous i.v.

**Fig. 1 (abstract P496).**
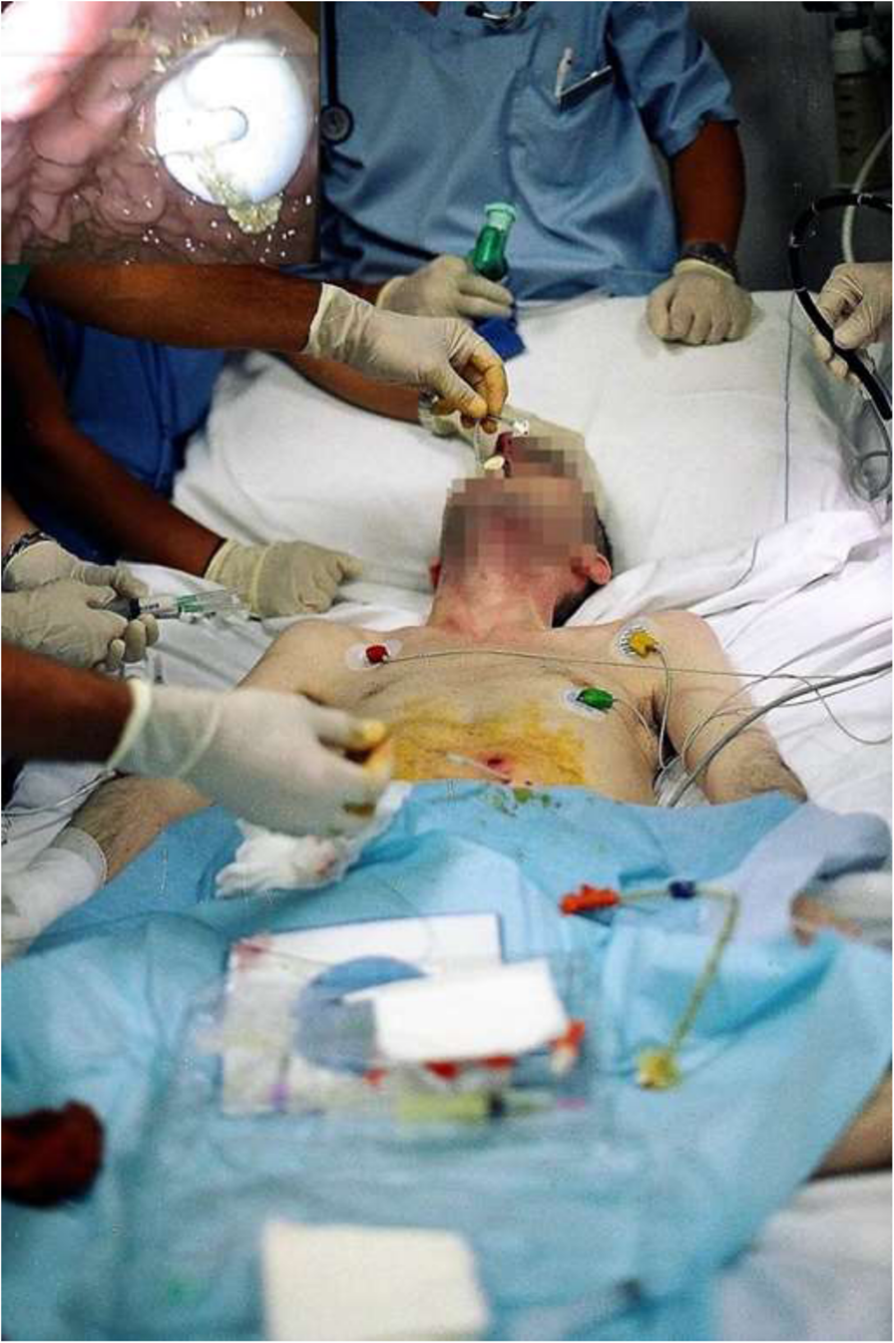
PEGJ procedure in Parkinson’s Disease patient; Overlapped Figure: Endoscopy view of bumper placement

## P497 Sleep disorders in ICU survivors

### C Alexopoulou, A Proklou, M Fanaridis, S Soundoulounaki, M Bolaki, EM Antonogiannaki, E Kondili

#### University Hospital of Heraklion, Crete, Heraklion, Greece

**Introduction:** Studies have shown that ICU survivors exhibit long-term neurocognitive impairment and perceived reduction in quality of life after ICU discharge, but studies examining sleep architecture and sleep disordered breathing (SDB) in ICU survivors after ICU discharge are scanty. The aim of our study was to assess sleep architecture and SBD in ICU survivors.

**Methods:** ICU survivors were screened for eligibility. Inclusion criteria were: age 18 - 80 yrs, mechanical ventilation >= 48 hours, GCS of 15 at the time of hospital discharge. Patients with a history of SBD, chronic neuromuscular disorders, chronic restrictive lung disease, congestive heart failure and respiratory failure at hospital discharge were excluded. Patients were evaluated within one week after hospital discharge and 6 months later. At both visits patients completed health related quality of life questionnaires (SF36 and Epworth Sleepiness Scale), underwent a physical examination, lung function tests including maximum inspiratory and expiratory mouth pressures, and an overnight full polysomnography (PSG).

**Results:** Sleep quality at 7 days of hospital discharge is poor, characterized by severe disruption of sleep architecture and excessive SDB, mainly of obstructive type which in 76% of patients was classified as moderate or severe. Although at six months after hospital discharge sleep quality remained relatively poor, significant improvement in N3 stage and AHI was observed, with more patients to be classified as normal or mild SDB. Both at hospital discharge and 6 months later quality of life was reduced but there was no relationship between the health related quality of life and sleep disturbances.

**Conclusions:** ICU survivors experience significant deterioration in their quality of life status with minor improvement 6 months later and a variety of sleep disturbances that seems to start getting better 6 months later.

## P498 Sleep in critically ill patients and outcome

### Y Boyko^1^, P Toft^1^, H Oerding^2^, JT Lauridsen^3^, M Nikolic^4^, PJ Jennum^5^

#### ^1^Odense University Hospital, Odense C, Denmark; ^2^Vejle Hospital, University of Southern Denmark, Vejle, Denmark; ^3^University of Southern Denmark, Odense M, Denmark; ^4^Rigshospitalet, Glostrup, Denmark; ^5^Rigshospitalet, University of Copenhagen, Glostrup, Copenhagen, Denmark

**Introduction:** Disrupted sleep in critically ill patients may be associated with delirium, prolonged stay in ICU and increased mortality. Polysomnography (PSG), the criterion standard method of sleep monitoring, is challenging in ICU due to interpretation difficulties, as the patterns defined by the standard classification for scoring sleep are absent in many critically ill patients. The aim of this study was to investigate if the presence of atypical patterns in critically ill patients’ PSG is associated with poor outcome measured by 90-days mortality in conscious critically ill patients on mechanical ventilation.

**Methods:** 70 PSGs (median duration 20 hours) recorded in conscious critically ill mechanically ventilated patients were scored by an expert in sleep medicine blinded to patient characteristics. Standard sleep scoring classification was used if possible. Otherwise, modified classification for scoring sleep in critically ill patients proposed by Watson et al. was applied [1]. The association of sleep patterns (normal or atypical) and micro-sleep phenomena (sleep spindles and K-complexes) with 90 days mortality was assessed using Weibull model by calculation of Hazard Ratios (HR).

**Results:** HR analysis showed twice as high mortality risk in case of atypical sleep compared to normal sleep; this was however not significant (HR 2.5; 95% CI 0.95-4.44; p=0.08). The presence of sleep spindles in PSG significantly reduced mortality risk to 1/3 (HR 0.33; 95% CI 0.13-0.86; p=0.02). The presence of K-complexes in PSG reduced mortality risk to ½, though not significantly (HR 0.52; 95% CI 0.24-1.12; p=0.1).

**Conclusions:** The absence of normal sleep characteristics in PSG in conscious critically ill patients on mechanical ventilation is associated with poor short-term outcome.

**References**

1. Watson et al. Crit Care Med 41:1958-1967, 2013.

## P499 Antipsychotics (APs) prescribing in critically ill delirious patients, the reported versus the perceived practice

### E Almehairi^1^, G Davies^1^, D Taylor^1^, S Yassin^2^, A Peers^3^, C McKenzie^1^

#### ^1^King’s College London, London, UK,^2^Guy’s and St Thomas Trust, London, UK,^3^South London and Maudsley NHS Foundation Trust, London, UK

**Introduction:** APs are the most commonly prescribed drugs in hyperactive/mixed delirium and agitation in critical care (CC) [1]. Yet evidence in CC is scant, there are known adverse effects (ADE) and prescription is out with the European license. Meticulous observation of AP selection, prescribing and safety, alongside delirium assessment/plan is essential to gain new knowledge and patients. When accompanied by prescribing clinicians perspective of delirium AP treatment results are more interpretable. We conducted a two-part single centre cohort study that aimed to describe/compare real to perceived delirium assessment/plan, APs prescribing and safety in CC adult patients at GSTT.

**Methods:** Part 1: a prospective survey, of CC prescribing clinician’s beliefs and attitudes to delirium assessment/plan, APs prescribing and safety over previous 12 months. Part 2: a meticulous audit of APs prescribing and safety and delirium/agitation assessment and plan, over period of 4 months.

**Results:** Part 1 Survey. 43 of 82 prescribers (53.6%) completed survey. 88% of reported using APs to treat delirium, with 83% selecting atypical APs as first option. Part 2 Audit. There were 2400 admissions to CC. APs were prescribed in 6.4% (188 prescription), 3.8% (113 prescription) were in delirium/agitation patients (Table 1). Survey (vs.) audit: In the survey 67% reported daily delirium screening whereas only 12.3% undertook daily screening in audit (Fig. 1). Higher quetiapine and lower IV haloperidol maximum daily dose were prescribed in audit in comparison with survey reported doses (Table 2). 12 Lead ECG was used to monitor AP ADE. In survey 34% reported assessing ECG once or more daily. Audit revealed only 19% actually did so (Fig. 2).

**Conclusions:** Authors believe perceived vs actual can identify key areas for Quality Improvement (QI). Major differences were in delirium assessment/plan and safety monitoring

**References**

[1] Thiboutot et al. Can J Hosp Pharm. 69(2):107-13, 2016

**Table 1 (abstract P499).** Characteristics of patients prescribed APs for treatment of delirium in critical care

	Haloperidol N=30	Quetiapine N=38	Olanzapine N=21	P value
Age	74 (66-81)	52 (42-72)	59(45-73)	0.0002*
Gender	22 Male, 8 Female	30 Male, 8 Female	15 Male, 6 Female	0.77
MV	18	33	15	0.040*
ECMO	1	5	0	0.10
CC LOS	12 (5-21)	24 (15-40)	25 (18-42)	0.003*
CC Unit	16 ICU, 4 HDU, 10 OIR	32 ICU, 5 HDU, 1 OIR	18 ICU, 2 HDU, 1 OIR	0.004*

**Table 2 (abstract P499).** Comparison of actual and perceived APs prescribed doses when treating critical care delirium

AP	Starting dose mg (a) Median IQ	Maximum daily dose mg/day (b) Median IQ	Audit vs. Survey comparison P value
Oral quetiapine-survey (63.8%)	25 (12.5-25)	200 (100-300)	(a) 0.205
Oral-quetiapine audit (42.6%)	25 (13-31)	100 (50-200)	(b) 0.014*
IV haloperidol-survey (26%)	1 (1-2.5)	2.5 (1.2-17)	(a) 0.33
IV haloperidol audit (22%)	1 (0.6-1)	16 (10-20)	(b) 0.003*
Oral-olanzapine-survey (11%)	2.5 (2.5-5)	5 (2.5-5)	(a) 0.6
Oral-olanzapine audit (22%)	20 (10-20)	1- (5-19)	(b) 0.2

**Fig. 1 (abstract P499).**
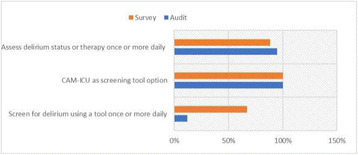
Delirium screening and assessment audit vs. survey

**Fig. 2 (abstract P499).**
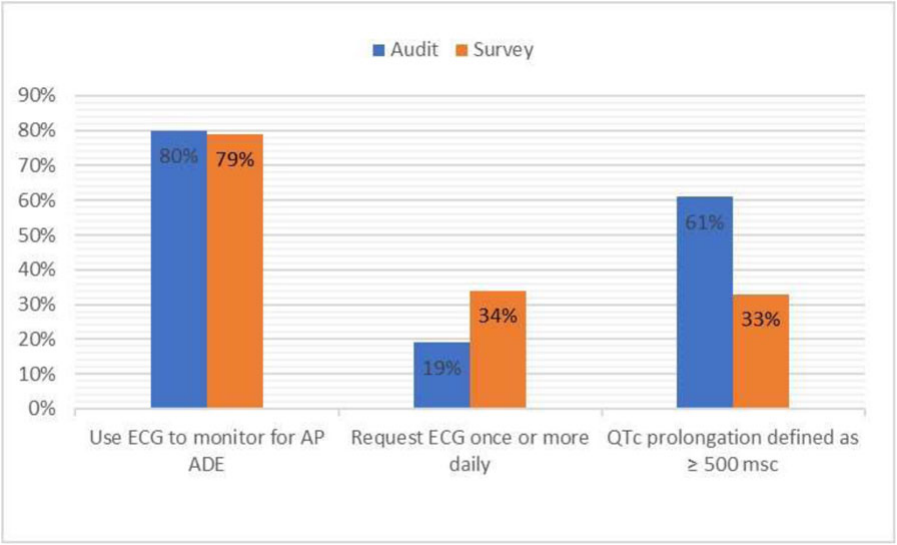
12 Lead ECG as APs ADE monitoring tool, audit vs. survey

## P500 Sedation practices and preferences of Turkish intensive care physicians: national survey

### S Urkmez^1^, E Erdogan^1^, T Utku^1^, Y Dikmen^1^

#### ^1^Istanbul University Cerrahpasa Medical Faculty, Istanbul, Turkey

**Introduction:** Sedation is one of the most common practices applied in the intensive care units. In literature there has been no data on sedation practices in Turkish ICUs, the aim was to provide knowledge on this matter.

**Methods:** An electronic survey form was generated with google forms. First part of the form included questions about demographics, and choices and routines of sedation administration. This part mostly contained multiple choice questions, which more than one choice could be indicated. Second part was comprised of some statements to investigate the attitudes of physicians, which were indicated on a five-point Likert scale. The link for the survey was posted to all email addresses registered in the Turkish Society of Intensive Care member database.

**Results:** Of 1700 members, 429 (25%) completed the survey form. Demographics are given in Table 1. Sedation was generally applied by the physicians (96%). The indications were mechanical ventilation (94%), agitation (87%), seizures (77%), anxiety (61%), delirium (59%). Drug choices of the respondents are shown in Fig. 1. Sedation level was evaluated daily by 73% of respondents, mostly using Ramsay Scale (57%). Daily established sedation level was indicated in 63.8%, and daily interruption of sedation was indicated in 71.4% answers. Sedation protocol was not used in 62.7% of the answers. Analgesics applied commonly, while 63% routinely evaluated pain and visual analogue scale (VAS) was the preferred method in 69% of the answers. 77.2% of physicians indicated routine use of neuromuscular blockers. In 50.5% answers routine evaluation for delirium was indicated, mostly using CAM-ICU.When the knowledge of 2013 SCCM guideline pain, agitation and delirium management, 38% indicated a positive answer.The respondents indicated their opinion for some comments on sedation, the answers are shown in the Table 2.

**Conclusions:** It may be concluded sedation practices may need to be improved by increasing awareness on novel concepts in this area.

**Table 1 (abstract P500).** Demographics of respondents

Median Age (min – max)	40 (29 – 63)
Sex % women	60.8
Median years spent working in the ICU (min – max)	7 (1 – 30)
Median size of the ICU (min – max)	16 (4 – 104)
Level of the ICU (%)	
Level 3	85.2
Level 2	11.3
Level 1	3.5
ICU type (%)	
Closed ICU	65.5
Open ICU	13.4
Mixt ICU	21.1
Hospital type (%)	
State University Hospital	34.5
State Teaching and Research Hospital	31.0
State Hospital	15.7
Private University Hospital	4.5
Private Hospital	14.3

**Table 2 (abstract P500).** Attitudes on Sedation

	Completely agree (%)	Agree (%)	No idea (%)	Disagree (%)	Completely disagree (%)
Sedation is a common adjunct in ICU treatment	32.6	52.8	1.2	9.9	3.5
Treating pain is more important than sedation	31.0	55.4	1.4	9.2	3.1
Sedation delays discharge from ICU	7.1	32.1	3.8	47.2	9.9
Sedation increases patient-ventilator asynchrony	1.2	3.8	2.4	58.3	34.4
Delirium is a frequent complication in ICU	29.2	61.4	2.6	5.9	0.9
Delirium has a negative effect on ICU outcome	43.3	51.3	1.6	2.4	1.4
Early mobilization has an important place in ICU	55.7	41.0	2.1	0.7	0.5
Early mobilization causes development of delirium	1.6	1.2	9.4	49.9	37.9
Post intensive care syndrome is a problem	33.8	50.5	13.6	1.6	0.5

**Fig. 1 (abstract P500).**
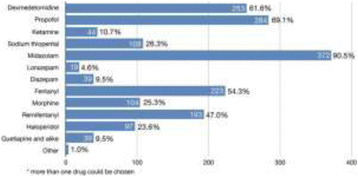
Drug Choices for Sedation

**Fig. 2 (abstract P500).**
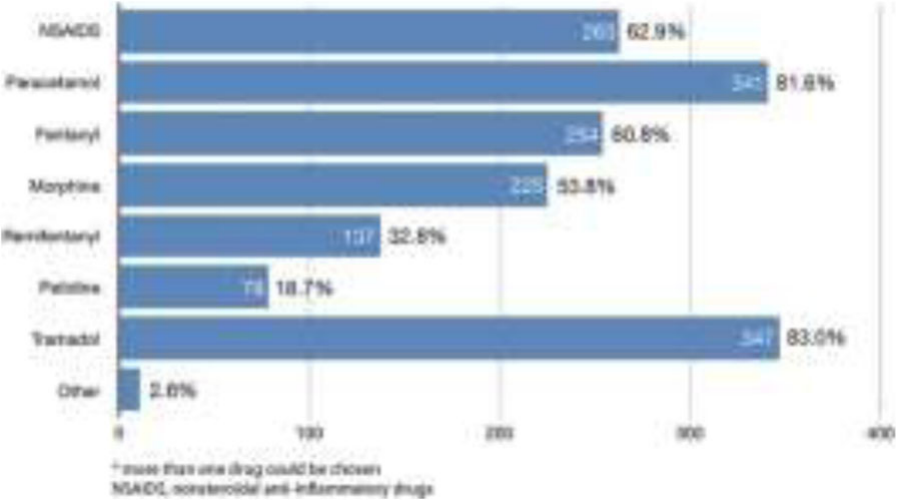
Drug Choices for Analgesia

## P501 Sedation during MRI: what medication to choose?

### I Kuchyn, K Bielka

#### Postgraduate Institute of Bogomolets National Medical University, Kiev, Ukraine

**Introduction:** In common sedation is required during MRI for adult uncommunicative patients or those with different psychiatric disorders [1]. Although it can be challenging to obtain the deep sedation level required to prevent the patient’s movement while maintaining respiratory and hemodynamic stability. Limited access to the patient may pose a safety risk during MRI.

Objectives: to compare efficacy and safety of dexmedetomidine sedation versus propofol during MRI in adults.

**Methods:** This prospective randomized study was conducted at department of anesthesiology and intensive care at Postgraduate Institute of Bogomolets national medical university (Kyiv, Ukraine) during 2015-2017. Uncommunicative conscious patients with acute ischemic stroke were included in the study and randomly allocated to 2 groups – dexmedetomidine (D) and propofol (P). The sedation goal was the same in the both group (RASS 0 to -2). Patients in group D receive dexmedetomidine infusion in dose 0.2-1.4 mcg/kg/h, in group P – propofol 1-4 mg/kg/h. Data are presented as median and 25-75 quartiles.

**Results:** 104 patients (52 in each group) with median age 62 [55-76] years were included in the study. The goal level of sedation was achieved during 84 [76-91]% of total sedation time in group D, and in 64 [51-76]% of time in group P (p<0.05). The incidence of complications varied between groups: arterial hypotension occurred in 8/52 (15%) patients in group D and in 5/52 (10%) patients in group P (p>0.05), bradycardia in 7/52 (13%) and 2/52 (4%) (p>0.05), desaturation in 2/52 (4%) and 14/52 (27%) (p<0.05), bradypnoe in 0/52 and 4/52 (7%) (p>0.05).

**Conclusions:** In this prospective randomized study dexmedetomidine comparing to propofol was associated with higher sedation quality and lower incidence of complication during acute ischemic stroke patients sedation for MRI.

**References**

1. Reddy et al Continuing Education in Anaesthesia Critical Care & Pain, 12:140–144, 2012

## P502 The usefulness of dexmedetomidine after lung transplantation in intensive care unit.

### M Ben-Rehouma, C Moulin, P Montravers

#### Hôpital Bichat, Paris, France

**Introduction:** Dexmedetomidine (DEX) showed some advantages in the sedation of patients in intensive care unit (ICU) [1]. Other studies described efficacity of DEX in ICU delirium [2]. The aim of this study was to evaluate the efficacity and safety of DEX after lung transplantation in ICU.

**Methods:** We conducted a prospective monocentric study in our surgical ICU between november 2015 at november 2017. In the first part of the study (november 2015 at november 2016), lung recipients did not received DEX; in the second part of the study DEX was used for the sedation in mechanically ventilated patients after lung transplantation. We compared the duration of mechanical ventilation in the two groups and the occurence of adverse effects.

**Results:** In total 59 lung recipients were enrolled. There was no difference between the two groups in demographic data, one or double-lung transplants, the cause of lung transplantation and the use of epidural infusion. In the DEX group, mechanical ventilation support was 118 hours versus 98.5 hours in the other group (p=0.55). There was no difference between delirium in the two groups (3/5, p=0.7). The occurence of adverse events like hypotension and bradycardia was significantly higher in the DEX group (4/0 for hypotension, p=0.013; 6/0 for bradycardia, p=0.0012).

**Conclusions:** The use of DEX after lung transplantation in ICU was not more efficience for the mechanical ventilator weaning. Lung recipients delirium was significantly the same in the two groups. The most notable effect was the occurence of bradycardia and hypotension in the DEX group.

**References**

1. SM. Jakob at al, JAMA. 2012

2. MC. Reade at al, JAMA. 2016

## P503 Impact of dexmedetomidine on the duration of invasive mechanical ventilation in pediatric intensive care patients - DEXPED trial

### M Genest^1^, V King^1^, Y El-Ghaddaf^1^, A Longpré^2^, MM Perreault ^1^, M Schnitzer^3^, S Ferreira-Guerra^3^, P Fontela^1^, T Di Genova^1^, M Dagenais^1^, N Ruo^1^

#### ^1^McGill University Health Centre, Montreal, Canada; ^2^Centre hospitalier universitaire de Sherbrooke, Sherbrooke, Canada; ^3^University of Montreal, Montreal, Canada

**Introduction:** DEXPED evaluated the impact of a prolonged exposure (>= 24 hours) to dexmedetomidine on the duration of invasive mechanical ventilation (IMV), length of PICU and hospital stay and use of other sedative agents.

**Methods:** DEXPED is a retrospective cohort study that included patients aged 0 to 18 years, admitted to the PICU of the Montreal Children’s Hospital between November 1st 2011 and April 25th 2015, requiring IMV and sedative agents for >= 48 hours. Patients exposed to dexmedetomidine during IMV (n=53) were compared to non exposed patients (n=159) using a propensity score analysis (1:3 ratio).

**Results:** Dexmedetomidine was administered at doses ranging from 0.2 to 2 mcg/kg/hour for a median duration of 67 hours (IQR [40.5;98]). The median duration of IMV was 161 hours (IQR [110-257]) in the exposed group and 116 hours (IQR [89.3-206]) in the non exposed group. The use of dexmedetomidine was associated with a smaller short-term probability of extubation (HR 0.67, 95%CI [0.47-0.96]; p=0.04). Patients who received dexmedetomidine were more likely to remain in the PICU (HR 0.62, 95%CI [0.42-0.92]; p=0.03) in any short time interval, as well as in the hospital (HR 0.56, 95%CI [0.35-0.88]; p=0.02), and received more opioids and benzodiazepines. However, a secondary analysis redefining exposure as initiation of dexmedetomidine within the first 48 hours from intubation suggested that exposure was associated with a greater short-term probability of extubation, although this study was not powered to perform this analysis.

**Conclusions:** Dexmedetomidine was associated with a longer duration of IMV. However, the association was inversed when patients received dexmedetomidine as a primary sedative agent. It is uncertain whether this difference of associations is due to immortal time bias or clinical features. Timing of initiation of dexmedetomidine in relationship to other sedatives may impact patient outcomes and should be considered in the planning of future trials.

## P504 Pharmacokinetics of dexmedetomidine during analgosedation in ICU patients

### A Bienert^1^, P Smuszkiewicz^1^, P Wiczling^2^, J Ber^1^, J Warzybok^1^, T Makiewicz^1^, J Matysiak^1^, A Klupczyñska^1^, I Trojanowska^1^, ZJ Kokot^1^, E Grzekowiak^1^, W Krzyañski^3(*)^

#### ^1^Poznan Univeristy of Medical Sciences, Poznan, Poland; ^2^Medical University of Gdañsk, Gdansk, Poland; ^3^University at Buffalo, Buffalo, NY, USA

^*^This project was supported by the grant 2015/17/B/NZ7/03032 founded by Polish National Science Centre and Kosciuszko Foundation, Exchange Program to the United States

**Introduction:** Dexmedetomidine (DEX) is an α2-agonist which has been increasingly used for analgosedation. Despite of many papers published, there are still only a few concerning the PK of the drug given as long-term infusion in ICU patients. The aim of this study was to characterize the population pharmacokinetics of DEX and to investigate the potential benefits of individualization of drug dosing based on patient characteristics in the heterogeneous group of medical and surgical patients staying in ICU.

**Methods:** All the subjects were sedated according to modified Ramsay Sedation Score of 3-4. Blood samples for DEX assay were collected on every day of the infusion and at the selected time points after its termination. The DEX concentrations in the plasma were measured using LC-MS/MS method. The following covariates were examined to influence DEX PK: age, sex, body weight, patients’ organ function (SOFA score), catecholamines and infusion duration. Non-linear mixed-effects modelling in NONMEM (Version 7.3.0, Icon Development Solutions, Ellicott City, MD, USA) was used to analyse the observed data.

**Results:** Concentration-time profiles of DEX were obtained from 27 adult patients (Table 1). The DEX PK was best described by a two-compartment model (Fig. 1). The typical values of PK parameters were estimated as 27 L for the volume of the central compartment, 87.6 L for the volume of the peripheral compartment, 38.5 L/h (9.2 ml/min/kg for a 70 kg patient) for systemic clearance and 46.4 L/h for the distribution clearance. Those values are consistent with literature findings. We were unable to show any significant relationship between collected covariates and DEX PK.

**Conclusions:** This study does not provide sufficient evidence to support the individualization of DEX dosing based on age, sex, body weight, SOFA, and infusion duration.

**Table 1 (abstract P504).** Demographic characterization of patients. Results are expressed as median and range

Parameter, unit	Median [Range or Number]
Age, years	59.5 [19-84]
Weight, kg	75 [45-100]
Male/Female	17/10
Infusion Time, h	42.8 [23.7-102]
Total dose of dex, mg	1.55 [0.29-6.67]
Infusion rate, ìg/kg/h	0.51 [0.1-1.5]
SOFA, score	12 [5-16]

**Fig. 1 (abstract P504).**
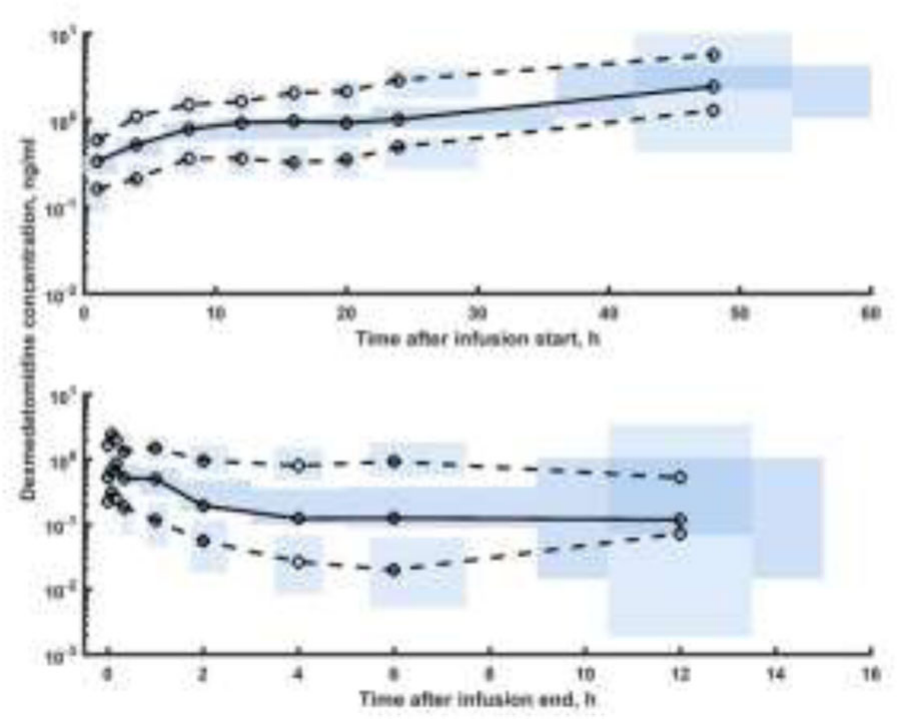
The prediction-corrected VPC plots for dexmedetomidine PK. The VPC plots show the simulation-based 95% confidence intervals around the 10th, 50th, and 90th percentiles of the PK data in the form of blue (50th) and gray (10th and 90th) areas. The corresponding percentiles from the prediction corrected observed data are plotted in black color

## P505 Peri-operative dexmedetomidine in high risk cardiac surgery - multicentre randomized double blind placebo controlled pilot trial

### Y Shehabi^1^, J Smith^1^, D Mcilroy^2^, M Green ^1^, L Wienberg^3^, S Kadiman^4^, M Bailey^1^, Z Endre^5^

#### ^1^School of Clinical Sciences - Monash University, Melbourne, Australia,^2^The Alfred Hospital, Melbourne, Australia,^3^The Austin Hospital, Melbourne, Australia,^4^National Heart Institute, Kuala Lumpur, Malaysia,^5^Prince of Wales Hospital, Sydney, Australia

**Introduction:** We hypothesized that perioperative dexmedetomidine (Dex) is safe and effective in patients undergoing high risk cardiac surgery. The primary purpose was to assess the feasibility, safety and efficacy of Dex compared to placebo. We compared vasopressors, inotropes, pacing and cardiac complications for safety and severe acute kidney injury (AKI), dialysis and death (Major Adverse Kidney Events MAKE) for efficacy.

**Methods:** Adults patients undergoing cardiac surgery [combined (valve + Coronary bypass) or complex] or with preoperative glomerular filtration rate (eGFR) < 60 mls/min/1.73m2 were included. Salvage or transplant surgery, dialysis, eGFR < 15 mls/min/1.73m2 and those on extracorporeal support were excluded. Dex (0.7 ug/kg/hr) was started at induction of anaesthesia and continued up to 24 hours after surgery. Equivalent volume of saline was given to control group. Standard intra and post-operative care was provided.

**Results:** We randomized 44 patients in the Dex group and 44 in the placebo (Pgp). The mean(SD) age 70.1(11.3) and eGFR 59.6(20.4) in all patients. No significant differences at baseline. in the Dex, 38.7% underwent complex surgery vs 19.7% pts in the Pgp. The mean(SD) bypass time and aortic clamp was comparable 140(62) and 106(49) min. The vasopressor requirements, pacing or cardiac complications at the end of bypass or in the first 24 hours after surgery were comparable. More patients in the Dex received inodilators 22.8% vs 13.6% in Pgp. MAKE occurred in 15.9% of Dex vs 11.4% in the Pgp. Dialysis requirements and 28-day mortality in the Dex group was 6.8% vs 11.4% and of 2.3% vs 9.1% in the Pgp respectively. Severe AKI occurred in 15.9% of Dex vs 11.4% Pgp. The ICU and hospital length of stay and postoperative ventilation time were comparable.

**Conclusions:** The use of perioperative Dex in high risk cardiac surgery is safe and well tolerated in the context of a double blind multicentre study. A Definitive trial is needed to investigate the role of Dex in high risk cardiac surgery.

## P506 Effects of propofol and dexmedetomidine on sleep architecture in ICU patients

### A Musica^1^, D Liberti^1^, C Pellegrini^1^, O Albanese^2^, C Cafora^1^, E De Blasio^1^

#### ^1^Rummo hospital, Benevento, Italy; ^2^Free worker, Benevento, Italy

**Introduction:** Sleep disturbances in ICU patients are frequent and associated with complications [1-3]. Goal of the study is to evaluate qualitative and quantitative alterations of sleep architecture under different regimes of sedation.

**Methods:** Nine tracheostomized COPD patients ready to be weaned from ventilation were enrolled.For each patient, the sleep architecture was studied by polisomnography (Sleep Profiler-Advanced Brain Monitoring) performing 3 recordings:basal registration, continuos infusion of propofol or dexmetomidine from 22 pm to 6 am. Rass target was -1/-2.

**Results:** The mean dose was 0.8mg/kg/h for propofol and 0.7 mcg/kg/h for dexmedetomidine.Quantitative analysis showed, a statistically significant longer Total Sleep Time (TST) and less sleep fragmentation (awakenings/hour) using dexmedetomidine. Qualitative analysis showed non statistical differences between the two regimens: longer N1 and N3 stage with propofol and a longer N2 and REM phase with dexmetedomidine. Furthermore, a reduced number of dosage adjustment was needed during dexmedetomidine sedation (Table 1).

**Conclusions:** Our study confirms the presence of disturbance of sleep archiecture in ICU patients. The use of sedatives could help to reduce sleep qualitative and quantitative disorder. Dexmedetomidine seems to reduce the wakefulness time and the sleep fragmentation but, while we haven’t found differences in sleep architecture using dexmedetomidine or propofol.

**References**

1. Delisle et al. Ann Intensive Care 1:42, 2011

2. Pasin et al. PLoS ONE 8(12):e82913.

3. Narisawa et al J Intensive Care 3:26, 2015

**Table 1 (abstract P506).** Please see text description

	propofol	dexmetedomidina	P value
Total Sleep Time (mean)	55%	73%	<0,05
Sleep fragmentation (awakenings/hour)	5,3	4,8	<0,05
N1(%)	15,2	11,3	NS
N2(%)	64,7	69	NS
N3(%)	7,6	5,8	NS
REM(%)	12,5	13,7	NS

## P507 Development of a multivariable prediction instrument for psychological morbidity in ICU survivors

### AS Milton, AR Schandl, PV Sackey

#### Karolinska Institutet, Stockholm, Sweden

**Introduction:** As many as 30% of intensive care unit (ICU) survivors suffer from psychological problems months to years after their hospital stay. Patients suffer predominantly from post-traumatic stress (PTS), depression and anxiety. Screening methods are lacking for individual prediction of psychological problems post-ICU.

**Methods:** A prospective multinational cohort study was performed in 10 ICUs in Sweden, Denmark and The Netherlands. All adult patients with an ICU stay >= 12 hours were screened for inclusion. Primary outcome was psychological problems three months after discharge from the ICU, assessed with the questionnaires Hospital Anxiety and Depression Scale (HADS) and Post-Traumatic Stress Symptoms Checklist-14 (PTSS-14). A subscale score >10 in the HADS and a score >45 in the PTSS-14 part B indicate clinically significant symptoms of depression, anxiety and PTS and was considered an adverse outcome. We collected data on 21 known risk factors for psychological problems post-ICU. Univariable and multivariable logistic regression modelling of risk factors was performed in order to create an instrument to be used bedside, predicting individual risk for adverse psychological outcome.

**Results:** 573 patients were included and 404 (71%) returned follow-up questionnaires. 14% of patients scored above the predefined cut-offs having symptoms of depression, anxiety or PTS. Age, lack of social support, depressive symptoms and traumatic memories at discharge remained significant after multivariable modelling and constituted the screening instrument (Table 1). The predictive value of the instrument was fairly good with an area under the receiver operating characteristics curve (AUROC) of 75% (Fig. 1).

**Conclusions:** We developed an instrument to be used at ICU discharge, predicting individual patients’ risk for psychological problems three months post-ICU. The instrument can be used as a screening tool for ICU follow-up and enable early rehabilitation.

**Table 1 (abstract P507).** Regression coefficients and odds ratios for predictors of psychological morbidity

Risk factor	Regression coefficient	Odds ratio	p-value	95% CI
Lack of social support	15.46	3.28	<0.01	1.47 7.32
Depressive symptoms (PHQ-2)	3.28	1.29	<0.01	1.10 1.50
Traumatic memories (PTSS-14A)	4.72	1.44	<0.01	1.13 1.82
Age		Data reported separately		

**Fig. 1 (abstract P507).**
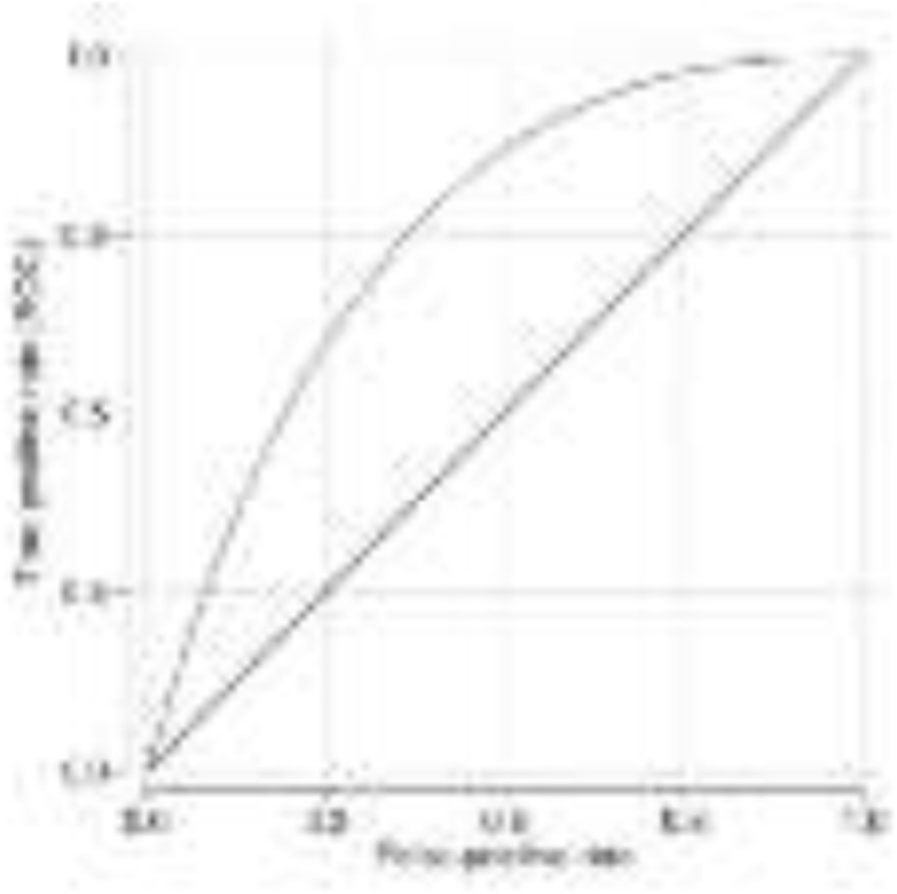
Receiver Operating Characteristics (ROC) curve for the predictive model

## P508 Improving the patients hospitalization experience in an intensive care unit by contact with nature

### W Yacov ^1^, Y Polishuk^2^, A Geal-dor^2^, G Yosef- hay^2^

#### ^1^Kaplan Medical Center, Rehovot, Israel; ^2^Kaplan medical center, Rehovot, Israel

**Introduction:** The intensive care unit is characterized by a noisy and threatening work environment using multi tecnologic equipment.the staff works very intensively caring for very complicated and unstable patients.whilst caring for the patients physical needs one must not forget the patients mentally needs.the improvement of the patients hospitalization experience by changing the environment improves the mood and responsiveness to treatment gives hope for healing to the patient and family.

**Methods:** a quality questionare with open questions relating to the subjective sensory experience of the patients and their families. the patients were transferred to the "sun balcony" for a period of 30-60 minutes having their families alongside. music was transmitted and the patients were offered food and drinks if their condition allowed.

**Results:** the patients reported a significant improvement of hospilizaton experience following their exposure to the "nature environment". patients described the sensory experience as a positive, pleasant, quiet and relaxing experience. the contact with the sun, wind, sky and grass and being outside on the "sun balcony" allows a disconnection from the threatening ICU environment.

**Conclusions:** The "sun balcony" gave the patients a sense of hope and wish for healing. mobilizing complicated patients to the "sun balcony" is a big challenge which requires planning and preparation by the staff. yet by the proactive and creative thinking of the staff the patients are tranferred to the "sun balcony" to give them encouragement, a feeling of well being and hope for recovering. This intervention is costless and a routine procedure in the intensive care unit.

## P509 Psychopathology and the association with ICU memories in ARDS patients post ICU follow-up

### L Tadini Buoninsegni, L Cecci, M Cozzolino, M Ferraro, M Bonizzoli, A Peris

#### A.O.U. Careggi, Florence, Italy

**Introduction:** Long-term psychological outcomes of patients(pts) discharged from ICU represent an emergent relevant matter of concern.Systematic reviews refer prevalence of 23%-48% for anxiety,17%-43% for depression and 8%-35% for posttraumatic symptoms in ARDS patients.The onset of psychiatric symptoms after discharge, might be associated with patient’s competence to process memories related with hospitalization and with memories.

**Methods:** We selected 35 ARDS pts in ICU of a tertiary centre (Jan 2014-Dec 2016) at least 72 hour, for 6 months follow-up and 26 pts for 12 months follow-up after discharge. The psychopathological assessment was performed using scale as: Impact Event Scale-Revised (IES-R), Hospital Anxiety and Depression Scale (HADS), ICU Memory Tool (ICU-MT).

**Results:** Mean age was 53.11±14.32 at 6 months follow-up and 51,19±14,83 at 12 months. PTSD symptoms was fund respectively in 24% and 34.6% pts at 6 and 12 months; anxiety symptoms 24% and 23.1% of pts;depression symptoms in 24% and 30.8%. Significant correlations were fund between psychopathology at 6 months and memories of ICU: HADS anxiety with delusion memories (r 0.45,p<0.01); HADS depression with factual (r 0.46,p<0.05), feeling (r 0.49,p<0.01) and delusion memories (r= 0.59,p<0.01); feeling (r 0.45,p<0.05). At 12 months significant correlations was fund between HADS anxiety and feeling memories (r 0.48,p<0.05); IES-R and factual (r 0.45,p<0.01), feeling (r 0.68,p<0.01) and delusion memories (r 0.64,p<0.01).

**Conclusions:** The results of the study confirmed the importance of assessing psychopathology after discharge from ICU. The onset of these symptoms appeared to be mediated by specific traumatic memories related with ICU hospitalization. The main clinical recommendation emerging from this study is to investigate psychiatric history and develop psychological strategies to manage frightening or delusional experiences during ICU stay.

## P510 Outcome and quality of life in patients with a prolonged intensive care unit stay

### K Steenhaut, S Oeyen, D Benoit, J Decruyenaere, E Hoste

#### Ghent University Hospital, Gent, Belgium

**Introduction:** Data on long-term (LT) quality of life (QOL) in ICU survivors are limited, especially after a prolonged ICU length of stay (LOS). We assessed QOL longitudinally for up to 7 years in patients with a prolonged ICU-LOS.

**Methods:** A 1-year prospective observational cohort analysis was performed. All patients consecutively admitted to the medical or surgical ICU or burn unit of a university hospital with an ICU-LOS of >= 8 days were included. QOL was assessed at baseline (BL) and at 3 months (m), 1 year (y) and LT (median 7.2 years (IQR 6.8-8.3)) after ICU discharge with EQ-5D and SF-36 surveys. At LT, questions about daily life were added. In subanalysis, we compared 2 groups (G1 and G2) based on median ICU-LOS.

**Results:** 374 patients (66% men) with a median age of 59, an APACHE II score of 22 and a SOFA score of 7 at ICU admission were included. 5 patients (1.3%) were lost to follow-up. Median ICU-LOS in the cohort, G1 and G2 was 16 (IQR 10-26), 10 (IQR 9-12) and 26 days (IQR 20-37) respectively. During ICU stay, G2 had significantly more and longer need for any type of organ supportive therapy (p<0.001) and had higher maximum SOFA scores (p<0.001). ICU, hospital, 3m, 1y and LT-mortality rates in the cohort were 16, 26, 29, 39 and 62% respectively. These rates were similar in G1 and G2. In the cohort, QOL decreased at 3m (p<0.001), improved at 1y (p<0.001) and stabilized at LT (p=0.297) but remained under BL (p=0.003), except for the mental scores (p=0.158). G1 and G2 showed the same evolution in QOL at 3m and 1y, but the drop in QOL at 3m was greater in G2 vs G1 (p=0.044). At LT, G1 improved to a better QOL than BL (p=0.035), while G2 remained under BL (p=0.041). Also at LT, G2 vs G1 experienced more sexual dysfunction (48% vs 33%), sleep disorders (42% vs 37%) and financial problems (20% vs 12%) (all p>0.05).

**Conclusions:** At LT, QOL was decreased in ICU survivors with a prolonged ICU-LOS. This was driven by the subgroup that had a LOS >= 16 d. These patients may benefit from a better post-ICU follow-up.

## P511 Early rehabilitation in the intensive care unit: reality!

### L Alonso, E Sady, R Gregório, L Junqueira, V Veiga, T Alvarisa, N Postalli, P Travassos, R Vale, S Ordinola

#### Hospital BP - A Beneficência Portuguêsa de São Paulo, São Paulo, Brazil

**Introduction:** The early mobilization program during intensive care hospitalization presents numerous benefits related to the outcome of the patient. The objective of this study is to evaluate the safety of the implementation of an early mobilization protocol within the first 24 hours of admission and its impact on high functional status of the ICU.

**Methods:** Retrospective study, from March 2013 to May 2017, evaluating patients admitted to the Neurological ICU, assessing the hemodynamic, respiratory and neurological variables in patients submitted to the early mobilization program, consisting of progressive therapeutic activities, including sedestation and orthostatism assisted on the board and evaluated the impact on the functional status/degree of high muscle strength of the ICU.

**Results:** From March 2013 to May 2017, 11,219 patients were admitted to a neurological intensive care unit, of whom 9,873 were included in the early mobilization program. The mean age of the patients was 66.5 years, with SAPS 3 of 43.98 points (estimated mortality risk of 22.3%) and real mortality of 11.2%. During the program, 3% presented clinical instability, which was promptly reversed in all situations. Ninety-one percent of the patients presented maintenance or gain of muscle strength/functional status.

**Conclusions:** The application of an early mobilization program within 24 hours of patient admission was shown to be safe, positively influencing the rehabilitation of neurological patients.

## P512 Short term outcomes of elderly survivors of intensive care

### I Ben Saida, E Ennouri, W Zarrougui, J Ayachi, M Limam, N Fraj, N Sma, A Khedher, A Azouzi, M Boussarsar

#### Farhat Hached Teaching hospital, Sousse, Tunisia

**Introduction:** Given the worldwide rapidly aging of the population, the demand of critical care for elderly is increasing. Data on short -term outcomes of elderly patients after ICU discharge are sparse. The objective of our study was to assess short term outcomes of elderly after ICU discharge and their potential risk factors.

**Methods:** An observational prospective cohort study performed in a medical adult ICU. The study included all elderly survivors (>=65 years) after ICU admission. Data were collected between January 2014 and December 2015 and the outcomes were assessed by telephone interviews at 1 month after discharge. Factors associated with readmission and post ICU mortality are presented as odds ratios.

**Results:** During the study period, 102 elderly patients were discharged alive. The follow up was possible for 80 (78.43%) patients. Predictors of one-month readmission in univariate analysis were coronary disease (p=0.027), SAPSII (p=0.019) and decline in functional status (p=0.00). In multivariate analysis, SAPSII (OR, 1.066 ; 95%CI, [1.001- 1.135] ; p=0.047) and decline in functional status (OR, 15.17 ; 95%CI, [3.7- 61.6] ; p=0.000) were the independent predictors of early readmission. Mortality rate at 1 month was 22.5%. Risk factors of one-month mortality in univariate analysis were SAPSII (p=0.002), heart rate at discharge (p=0.031), World health organization(WHO) performance status at discharge (p=0.000) and decline in functional status (p=0.000). In multivariate analysis, independent predictors of 30-day mortality were SAPII (OR, 1.11 ; 95%CI, [1.03- 1.19] ; p=0.005), decline in functional status (OR, 7.7 ; 95%CI, [1.75- 34.03] ; p=0.007) and WHO performance status (OR, 2.43 ; 95%CI, [1.33-4.43] ; p=0.004).

**Conclusions:** The present study supports previous findings of good early survival of elderly after ICU stay. Comorbidities don’t have an important impact on short term outcome after critical illness, which is most strongly predicted by severity of illness and physiological reserve at discharge.

## P513 Characteristics and outcome of elderly patients in intensive care unit

### I Coelho Santos, R Assis, A Real, T Pereira, N Lopes, A Araújo, L Pessoa, N Catorze

#### Centro Hospitalar Médio Tejo, Abrantes, Portugal

**Introduction:** Patients aged 80 years or older presently account for approximately 10-15% [1] of all Intensive care unit (ICU) admissions in Europe. The major challenge nowadays is to admit those elderly patients who will benefit from ICU treatment. The objective of this study is to describe the characteristics and outcomes of patients >=80years old admitted to the ICU.

**Methods:** Retrospective observational study of all patients aged >= 80 years admitted for >24h in 2016. Demographic data, admission diagnosis, APACHE II and SAPS II scores, use of ICU resources and mortality were collected.

**Results:** 152 patients (25%) were included, with a mean age of 85,06. Female gender was more prevalent (51.3%). Mean length of stay was 4,48 days with mean SAPS II and APACHE II scores of 40,23 and 38,82 respectively. The most prevalent type of admission was medical, 70,4% (n=107) and from these the main reasons for admission were respiratory disease (n=45; 29,6%) and sepsis (n=32; 21%). ICU Mortality rate was 29,6% (n=45), whereas 6-month mortality was 31,5% (n=48).Survival rate was often related with cardiovascular (23 [15,1%], P<.001) and respiratory diseases (32 [21%], P=.01), whereas non-survivors were admitted due to sepsis and neurologic causes. Mortality rate was independent from the mean length of stay, noninvasive ventilation and renal replacement therapy, but dependent for previously comorbidities. Mechanical ventilation was an independent predictive factor of ICU mortality (P<.001) and 6-month mortality (P=.008).

**Conclusions:** Nearly 70% of patients aged >= 80 years were discharged alive from ICU, and less than 50% survived 6 months after ICU admission.Our study revealed a better prognosis for admissions due cardiovascular and respiratory diseases. Efforts should be done to identify earlier septic and neurological patients that benefit ICU treatment, and reevaluate the critical patient pathway, in this special population.

**References**

1. Boumendil A et al. J Am Geriatric Soc 53:88-93, 2005

## P514 Voolcano I: very old and old people with critical disease I

### E Sousa, T Leonor, M Fernandes, R Pinho

#### Centro Hospitalar de Entre Douro e Vouga, Santa Maria da Feira, Portugal

**Introduction:** The aging of the population is a fact. The subgroup of very old (>= 80 years (ys)) is the one that increases the most rapidly. Intensive Care Unit (ICU) admission of these patients is an ongoing discussion worldwide.

Our ICU has designed the VOOLCAno aiming its characterization and reviewing outcomes, to find some predictive indicators. The purpose of this first analysis is to evaluate specifically the group of very old patients (VOLDs) admitted to a tertiary Portuguese hospital ICU.

**Methods:** Retrospective observational study was preformed, included all VOLDs admitted in ICU during 15 years (2002-2016). Demographic data, admission diagnosis, severity scores, Charlson comorbidity index, length of stay and outcomes were considered. Data analysis used SPSS software.

**Results:** We found a total of 460 admissions. The median age was 83.0 ys with IQR 4; Mostly male with medical admission diagnosis (sepsis and respiratory failure due to infection). There was a median Acute Physiology and Chronic Health Evaluation II of 18 (IQR 8) and Simplified Acute Physiology Score II of 49 (IQR 16). Median Charlson comorbidity index was 6.0 (IQR 2). Median length of stay was 3.9 days (IQR 8.2). Concerning outcomes, we found intra-ICU mortality of 36%; intra-hospital after ICU discharge mortality of 12% and mortality after hospital discharge of 41%. Identified as predictors of intra-hospital mortality the use of mechanical ventilation (p < 0.001), urgent surgical admission or medical admission versus schedule surgical admission (p < 0.001) and the absence of oncologic disease (p = 0.024). On multivariate analysis, only mechanical ventilation (p = 0.002, HR 0.35, 95% C.I. 0.18-0.68) and urgent surgical admission versus schedule surgical admission (p = 0.002, HR 0.29, 95% C.I. 0.14-0.63) remain significant.

**Conclusions:** Recognizing the need to understand what is the biologic|funcional age (opposed to chronologic age) would be beneficial in the selection of VOLDs to ICU admission.

## P515 Organ failure and return to work after intensive care

### S Riddersholm^1^, S Christensen^2^, K Kragholm^1^, CF Christiansen^2^, BS Rasmussen^1^

#### ^1^Aalborg university hospital, Aalborg, Denmark; ^2^Aarhus Univeristy hsopital, Aarhus, Denmark

**Introduction:** Organ failure is associated with an unfavorable prognosis. Nevertheless, the association with capability to return to work remains unclear. Therefore, we investigated the association between organ support therapy as a proxy for organ failure and return to work in a nationwide cohort of ICU survivors.

**Methods:** We linked Danish registry-data on ICU- and hospital-survivors working prior to hospital admission during 2005-2014, 18-65 years of age, with an ICU length of stay > 24 hours and not previously treated with dialysis, to data on return to work. We reported cumulative incidences (chance) of return to work with death as competing risk, and compared rate of return to work in adjusted Cox regression-models by number of organ support therapies including renal replacement therapy, cardiovascular support and mechanical ventilation and stratified on primary hospital-admission diagnosis.

**Results:** Of 24,795 patients 18-65 years of age, 35% (8,660) survived to hospital discharge (Tables 1 and 2). Among these, the chance of return to work was 72.2% (95% CI [71.3-73.2]) within two years (Fig. 1). Compared to patients with no organ support therapy, adjusted Cox regression showed that patients with support of one, two, or three organs had reduced chance of return to work (HR 0.76 [95% CI [0.71-0.81]), HR 0.74 [95% CI [0.70-0.78]) and HR 0.69 [95% CI [0.62-0.78], respectively) (Fig. 2). When stratified an increasing number of organ support was associated with a decreased chance of return to work among patients with infection, respiratory failure or trauma but not among patients with neoplasms or endocrine, gastrointestinal and cardiovascular diagnoses.

**Conclusions:** More than 70% of ICU-survivors returned to work. Overall, the chance of return to work within two years was independent of the number of organ support therapies in patients with at least one organ support therapy. However, in subgroups, the chance of return to work decreased with increasing number of organ-support therapies.

**Table 1 (abstract P515).** Baseline and in-hospital characteristics by primary admission diagnosis group

	Infectious (n=995)	Cardiovascular (n=2675)	Respiratory (n=622)	Gastrointestinal (n=445)
Age,median [25%-75%]	51.7 [39.8, 58.4]	52.6 [45.1, 58.7]	50.9 [38.7,58.3]	51.7 [41.5, 57.8]
Male sex(sd)	602 (60.5)	1673 (62.5)	392 (63.0)	267 (60.0)
Support of 0 organs	326 (32.8)	850 (31.8)	121 (19.5)	198 (44.5)
Support of 1 organ	276 (27.7)	728 (27.2)	207 (33.3)	92 (20.7)
Support of 2 organs	292 (29.3)	982 (36.7)	238 (38.3)	120 (27.0)
Support of 3 organs	101 (10.2)	115 (4.3)	56 (9.0)	35 (7.9)
Hospital-LOS	18.0 [10.0, 34.0]	21.0 [12.0, 54.0]	19.0 [9.2, 41.0]	22.0 [13.0, 43.0]

**Table 2 (abstract P515).** Baseline and in-hospital characteristics by primary admission diagnosis group

	Endocrine (n=157)	Neoplasms (n=573)	Trauma (n=1819)	Other (n=1374)
Age, median [25%-75%]	42.7 [27.0, 54.1]	52.3 [42.5, 59.1]	39.2 [25.0, 51.9]	42.1 [30.6, 54.3]
Male (sd)	78 (49.7)	324 (56.5)	1404 (77.2)	672 (48.9)
Support of 0 organs	104 (66.2)	334 (58.3)	794 (43.7)	606 (44.1)
Support of 1 organ	20 (12.7)	118 (20.6)	555 (30.5)	353 (25.7)
Support of 2 organs	21 (13.4)	102 (17.8)	444 (24.4)	325 (23.7)
Support of 3 organs	12 (7.6)	19 (3.3)	26 (1.4)	90 (6.6)
Hospital-LOS	8.0 [5.0, 15.0]	17.0 [10.0, 37.0]	23.0 [10.0, 62.0]	17.0 [8.0, 40.0]

**Fig. 1 (abstract P515).**
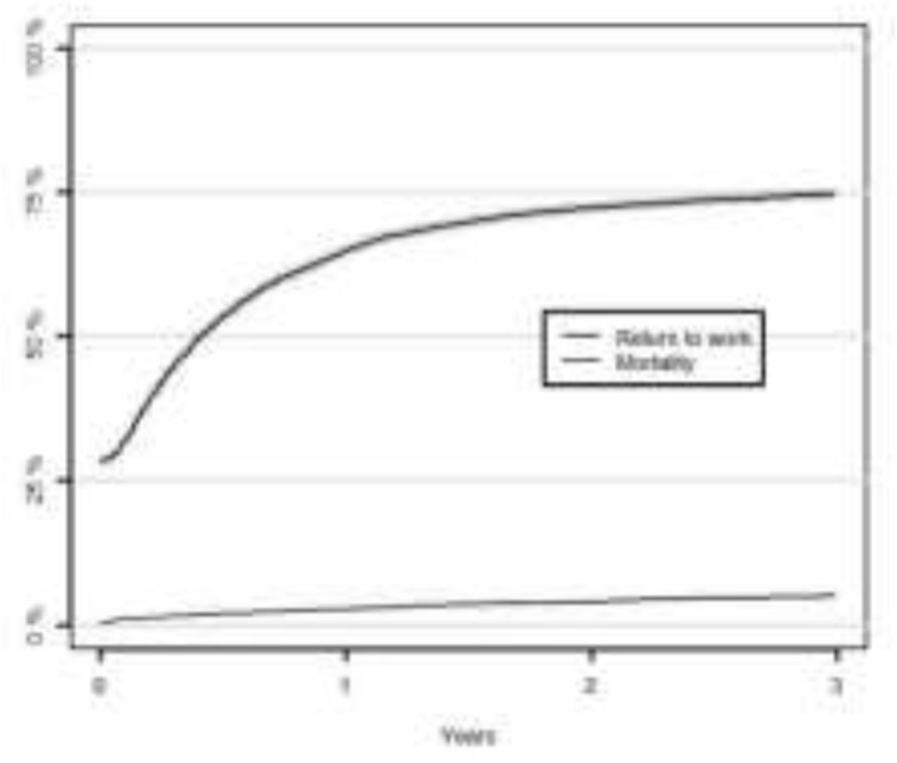
Cumulative incidence of return to work with death as competing risk

**Fig. 2 (abstract P515).**
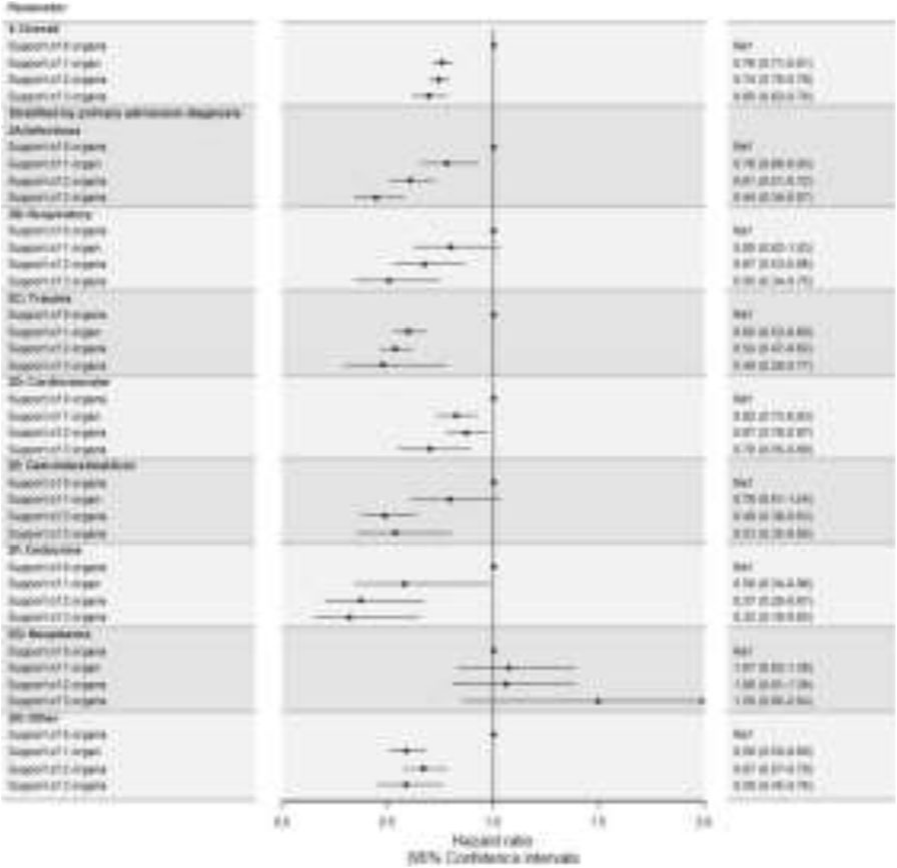
Multivariable cox regressions of return to work by: 1. Number of organ support in all 8660 ICU-survivors. 2 A-H: Analysis of organ support stratified on hospital-admission-diagnosis group Adjusted for age, sex, educational level, status of living alone. Data show return to work for survivors to hospital discharge. The hazard ratio (HR) denotes the relative hazard rate of return to work for the parameter compared to its reference after adjustment for confounders

## P516 Factors associated with non-return to work among general ICU survivors: a multicenter prospective cohort study

### R Rosa^1^, C Robinson^1^, M Falavigna^1^, L Biason^2^, P Cardoso^3^, P Berto^3^, M Mattioni^1^, L Tagliari^1^, P De Leon^1^, J Maccari^1^, R Oliveira^1^, J Andrade^4^, G Medeiros^1^, V Machado^1^, F Gomes^1^, C Dietrich^1^, F Dutra^1^, L Anzolin^1^, M Oliveira^1^, E Sanchez^1^, S Da Silva^1^, S Menezes^1^, D De Souza^1^, R Jeffman^1^, D Schneider^1^, D Sganzerla^1^, D De Oliveira^1^, C De Oliveira^1^, R Kochhann^1^, C Teixeira^1^

#### ^1^Hospital Moinhos de Vento, Porto alegre, Brazil; ^2^Hospital Independência, Porto Alegre, Brazil; ^3^Hospital de Clínicas de Porto Alegre, Porto Alegre, Brazil; ^4^Hospital Ernesto Dornelles, Porto Alegre, Brazil

**Introduction:** Critical care patients may develop long-term health problems associated to their illness or ICU treatments, which may affect their work capacity. Unfortunately, studies evaluating the impact of critical illness on work-related outcomes are scarce.Therefore, we aimed to investigate factors associated with non-return to work among ICU survivors.

**Methods:** A prospective cohort study involving ICU survivors of 6 Brazilian tertiary hospitals was conducted from May 2014 to August 2017. Patients with a ICU stay >72 h (for medical and emergency surgical ICU admissions) or >120 h (for elective surgical ICU admissions) who were discharged alive from the hospital were evaluated through a structured telephone interview 3 months after discharge from the ICU. A stepwise multivariate Poisson regression analysis adjusted by age, gender and years of education was used to evaluate the association of sociodemographic- and ICU-related variables with non-return to work.

**Results:** In total 986 ICU survivors completed the 3-month follow-up. Of these, 285 (29%) were working before ICU admission. Only 113 of 285 patients (39%) returned to work within the first 3 months after discharge from the ICU. Percentage of risk of death at ICU admission (relative risk [RR], 1.85; 95% confidence interval [CI], 1.04-3.29), decrease in physical functional status in relation to the pre-ICU period measured by Barthel Index (RR, 1.86; 95% CI, 1.48-2.35), not having a private healthcare insurance (RR, 1.57; 95%CI, 1.20-2.04), and a formal work status before ICU admission (RR, 1.44; 95%CI, 1.11-1.87) were independently associated with non-return to work within the first 3 months after discharge from the ICU.

**Conclusions:** The present study findings suggest that ICU factors, such as the severity of critical illness and ICU-related physical disability, and social factors, such as not having a private healthcare insurance and previous formal work status, may play an important role in the risk of non-return to work among ICU survivors.

## P517 A 23 year review of adult patients admitted to critical care with learning disabilities (LD)

### C Mearns, B Bray

#### Surrey & Sussex Healthcare NHS Trust, Redhill, UK

**Introduction:** Mortality rates among people with moderate to severe learning disabilities (LD) are 3 times higher than in the general population [1-3]. This study was designed to examine Critical Care admissions with learning disabilities in terms of mortality, demographics and reason for admission.

**Methods:** Data was retrieved for adult patients (>16 years old) between Sept 1993 and 2016. The Ward Watcher database for ICUs within Surrey and Sussex Healthcare NHS Trust was interrogated using search words including, learning disability, cerebral palsy, Down’s syndrome and autism.

**Results:** There were 154 episodes (1.4% of all admissions) of patients admitted with LD. 10% of the LD patients had more than 1 admission. Respiratory is the most common system affected (46%). Logistic regression suggests survival is highest in those with a neurological reason for admission (p=0.007). Proportionally LD patients were young compared to the total population (Fig. 1). We found that mortality appears to increase rapidly in those over 60 years of age and overall mortality is greater in those with LD (Fig. 2).

**Conclusions:** From April 2018 all UK Trusts will be required to complete a detailed review for patients with LD who die whilst in hospital care. This follows MENCAP’s report ‘Death by Indifference’ which exposed deficiencies in the care of 6 people with LDs who died whilst in NHS care and the subsequent Confidential Inquiry into premature deaths of people with learning disabilities. In our population, LD patients have an earlier death than the general population and the overall mortality from critical illness is greater.

**References**

1. Health Inequalities & People with Learning Disabilities in the UK: 2010 Emerson & Baines

2. Death by Indifference. MENCAP 2004

3. Confidential Inquiry into premature deaths of people with learning disabilities. CIPOLD 2013

**Fig. 1 (abstract P517).**
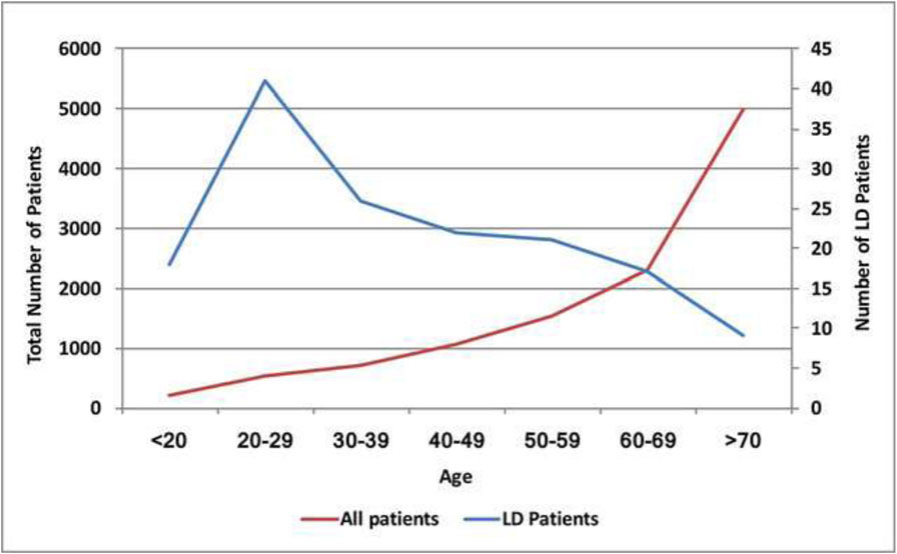
Graph of admission age in LD vs all patients

**Fig. 2 (abstract P517).**
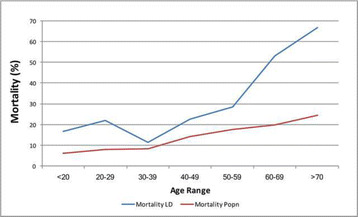
Graph of total mortality in LD vs all patients

## P518 Comparison of home and clinic follow-up visits after hospital discharge for post-ICU patients: a cross-sectional study

### R Rosa^1^, C Robinson^1^, P Berto^2^, P Cardoso^2^, L Biason^3^, J Maccari^1^, D Sganzerla^1^, D Schneider^1^, P De Leon^1^, M Mattioni^1^, L Tagliari^1^, M Falavigna^1^, M Oliveira^1^, L Anzolin^1^, S Da Silva^1^, F Dutra^1^, S De Menezes^1^, D De Souza^1^, E Sanchez^1^, R Kochhann^1^, F Gomes^1^, G Medeiros^1^, V Machado^1^, J Andrade^1^, C Dietrich^1^, C Teixeira^1^

#### ^1^Hospital Moinhos de Vento, Porto alegre, Brazil; ^2^Hospital de Clínicas de Porto Alegre, Porto Alegre, Brazil; ^3^Hospital Independência, Porto Alegre, Brazil

**Introduction:** Post-ICU patients may face barriers to access clinic-based rehabilitation programs due to the severity of their disabilities. We aimed to compare the burden of disability between post-ICU survivors who attended to a post-ICU follow-up clinic appointment and those who demanded home visits.

**Methods:** A cross-sectional study was conducted between February and November 2017 in a post-ICU follow-up service which is reference for 4 tertiary hospitals in Southern Brazil. Post-ICU patients with a ICU stay >72 h (for medical and emergency surgical ICU admissions) or >120 h (for elective surgical ICU admissions) who were discharged alive from the hospital were invited by telephone to participate in a clinic-based multidisciplinary appointment 4 months after ICU discharge. Home visits were offered to patients who claimed impossibility to attend the clinic appointment due to the severity of their disabilities. Frailty was evaluated through the Modified Frailty Index (MFI), physical functional status through Barthel Index (BI), cognitive function through the Mini-Mental Status Examination (MMSE), and anxiety and depression through the Hospital Anxiety and Depression Scale (HADS).

**Results:** In total, 120 patients were invited to attend to the post-ICU follow-up, 65% (n=78) accepted to participate in the study (clinic [n=61], home visit [n=17]). In comparison to clinic patients, home visit patients had higher median MFI scores (4 [IQR, 2-5] vs. 1 [IQR, 0-3], p<0.001), higher median decrease in their BI scores in relation to the pre-ICU period (45 [IQR, 20-70] vs 5 [0-15], p<0.001), lower median MMSE scores (21 [16-25] vs 26 [23-29], p=0.02), and more unplanned rehospitalizations (41.2% vs 11.7%, p=0.01). The scores of HADS for anxiety and depression did not differ between the two study groups.

**Conclusions:** Post-ICU follow-up policies should incorporate home visits as part of care, given that sicker patients may have difficulties to access clinic-based rehabilitation programs.

## P519 A multidisciplinary approach at the emergency department to admit potential organ donors for end-of-life care to the intensive care unit

### M Witjes^1^, A Kotsopoulos^2^, L Otterspoor^3^, I Herold^3^, K Simons^4^, K Woittiez^5^, J Eijkenboom^6^, H Van der Hoeven^1^, F Abdo^1^

#### ^1^Radboud university medical center, Nijmegen, Netherlands; ^2^Elisabeth Tweesteden hospital, Tilburg, Netherlands; ^3^Catharina hospital, Eindhoven, Netherlands; ^4^Jeroen Bosch hospital, ‘s-Hertogenbosch, Netherlands; ^5^VieCuri hospital, Venlo, Netherlands; ^6^Maxima Medical Center, Veldhoven, Netherlands

**Introduction:** The aim of the present study is to improve the recognition of potential organ donors by implementing a multidisciplinary approach for organ donation at the emergency department (ED) [1].

**Methods:** In a prospective intervention study, we implemented this approach in six hospitals in the Netherlands. When the decision to withdraw life sustaining treatment was made at the ED in patients with a devastating brain injury without contra indications for organ donation, an Intensive Care Unit (ICU) admission for end-of-life care was considered. Every ICU admission for end-of-life care was evaluated. Interviews were conducted with emergency physicians, neurologists and ICU physicians according to a standardized questionnaire. This interview focused on medical decisions that were made and difficulties arising during hospitalization.

**Results:** From 1 January 2016 to November 2017 data were collected on the number of patients admitted to the ED with acute brain injury. In total, 50 potential organ donors were admitted to the ICU for end-of-life care. Donation was either requested in the ED (12%), ICU (78%), neurology department (4%), or donation was not requested (6%). Out of 48 donation requests, 26 families (51%) consented to donation. This led to 21 successful organ transplantations. In four of these 21 patients family consent was obtained to intubate them solely for the purpose of organ donation. The most important points raised during the interviews were: explaining the non-therapeutic ICU admission to the family, the location where donation should be requested (ED/ICU) and utility of ICU resources.

**Conclusions:** A close collaboration between the ED, neurology department and ICU is necessary and achievable in order not to miss potential organ donors in patients with acute brain injury with a futile prognosis in the ED.

**References**

1. Witjes M et al. Am J Transplant 17(7):1922-7.

## P520 Physicians’ ability to predict hospital mortality in acutely deteriorating patients: a cohort study

### J Ramos^1^, R Dias^2^, D Forte^2^

#### ^1^Hospital Sao Rafael, Salvador, Brazil; ^2^Hospital das Clinicas, Sao Paulo, Brazil

**Introduction:** The aim of this study was to assess the accuracy of physician’s prediction of hospital mortality in critically ill patients in an intensive care unit (ICU) scarcity setting.

**Methods:** Prospective cohort of acutely ill patients referred for ICU admission in an academic, tertiary hospital in Brazil. Physicians’ prognosis and other variables were recorded at the moment of ICU referral.

**Results:** There were 2374 analyzed referrals. Physician’s prognosis was associated to hospital mortality. There were 593 (34.4%), 215 (66.4%) and 51 (94.4%) deaths in the groups ascribed a prognosis of survival without disabilities, survival with severe disabilities or no survival, respectively (p<0.001) (Fig. 1). Sensitivity was 31%, specificity was 91% and the area under the ROC curve was 0.61 for prediction of mortality. After multivariable analysis, severity of illness, performance status and ICU admission were associated to an increased likelihood of incorrect classification, while worse predicted prognosis was associated to a lower chance of incorrect classification. Physician’s level of expertise had no effect on predictive ability.

**Conclusions:** Physician’s prediction was associated to hospital mortality, but overall accuracy was poor, mainly due to low sensitivity to detect mortality risk. ICU admission was associated to increased incorrect classification, but there was no effect of physician’s expertise on predictive ability.

**Fig. 1 (abstract P520).**
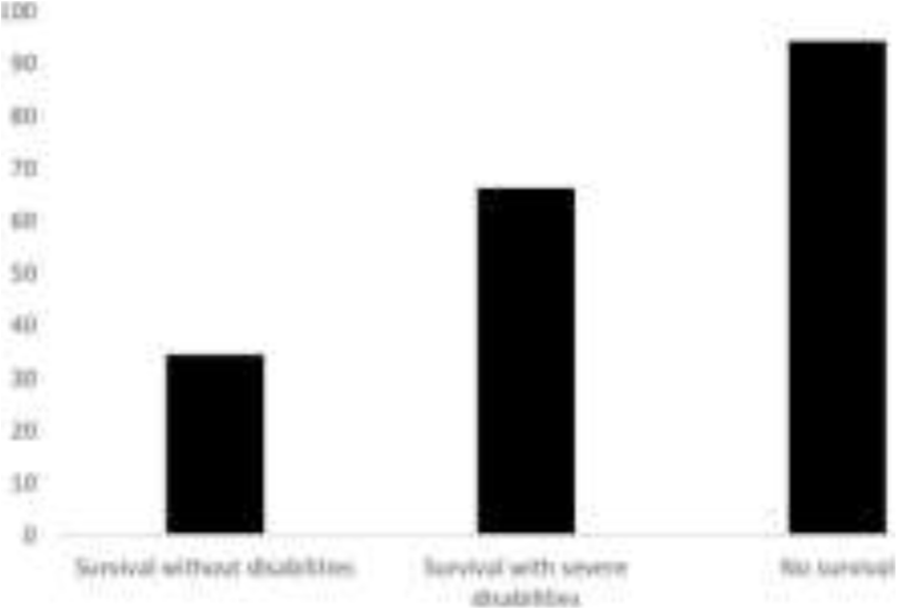
Association of physician’s prognosis with hospital mortality (p<0.001).

## P521 What are physicians in doubt about? An interview study in a neuro-intensive care unit.

### A Robertsen^1^, E Helseth^1^, R Førde^2^

#### ^1^Oslo University Hospital, Oslo, Norway; ^2^University of Oslo, Oslo, Norway

**Introduction:** Inescapable prognostic uncertainty, lack of decision-making capacity, risk of death or disability and long recovery trajectories complicate decision-making after traumatic brain injury.

**Methods:** To elicit experienced physicians’ perspective we interviewed 18 neurosurgeons, intensive care- and rehabilitation physicians from Oslo University Hospital about being in doubt about whether to offer, continue, limit or withdraw life-sustaining treatment and on how such cases were approached. Interviews were audiotaped and transcribed verbatim, coded and analysed using systematic text condensation by a clinician (AR) and a medical ethicist (RF).

**Results:** The difficulty of decision-making when there is prognostic uncertainty was acknowledged, leading to adaptive approaches; willingness to change and adjust plans along the way. To have access to different opinions within the physician group was seen as constructive. Time-critical decisions were based on team discussions and physician’s discretion. None-time critical decisions were reached through a process of creating common ground between the medical team and family. Themes physicians where in doubt about or expressed different opinions towards: 1) Appropriate aggressiveness of treatment in a given situation. 2) If and when to initiate discussions on appropriateness of treatment. Some believed that even addressing the issue in young patients or if small improvements were seen was inappropriate due to the possibility of late recovery. Physicians questioned the value of previously expressed patient’s wishes in this context. 3) Optimal timing and type of decisions. The need for nuanced individualized plans was recognized. To have a plan as opposed to just “wait and see” was seen as especially important in medical unstable patients.

**Conclusions:** Physicians expressed different views on appropriateness and optimal timing of level of care discussions and decisions in traumatic brain injury. A need for a more structured approach was exposed.

## P522 Improving effective communication in the ICU

### C Zucak, N Smith

#### Nepean Hospital, Kingswood, NSW, Australia

**Introduction:** There is a relationships between Intensive care patients losing the ability to speak and negative emotions [1]. Nursing care is challenging when patients are unable to verbalise and factors like pain and comfort are misjudged.. Our Intensive Care Unit has introduced a communication tool Intelligaze grid 3 which enables patients with primary motoric disorders to communicate their needs. A quality improvement study reviewed the methods of communication and interactions that our nurses use for patients who are ventilated. The objective of the study was to promote areas of improvement with communication in the ICU.

**Methods:** We used a mixed-methods qualitative and quantitative study to evaluate the communication tools used by our nursing staff to interact with ventilated patients. A convenient data sample for all nurses working on particular dates was collected which is 66% of the nursing workforce. The study has been approved as a Quality Assurance project by the Human Research Ethics Committee of Nepean Hospital.

**Results:** Sixty registered nurses (66%) participated in the study. The most common communication tool used with patients was closed YES/NO questions(27%), followed by hand gestures(21%), magnetic writing board(19.8%), lip reading(14.4%) and alphabet board(7.2%). The descriptive analysis identified challenges were levels of sedation, weakness, non-English speaking patients and delirium. A significant finding was that only 6% of nurses identified the patients message being understood and 5% acknowledged listening as effective communication.

**Conclusions:** Communication is a vital aspect of ICU nursing and is achieved through dialogue and specialised skills. The study concluded that ICU nurses find it difficult to communicate effectively with ventilated patients. The introduction of Intelligaze Grid 3 has improved patient communication and promotes holistic nursing care.

**References**

1. Hoorn.S, et al. Crit Care:20, 2016.

## P523 Evaluation of family satisfaction instrument in multicultural Middle Eastern critical care units

### A Ali, K Krishnareddy, H Hon

#### Sheikh Khalifa Medical City, Abu Dhabi, United Arab Emirates

**Introduction:** This cross sectional study was designed to investigate the level of family satisfaction in 3 intensive care units in a tertiary hospital in the United Arab Emirates (UAE), which is a multicultural society

**Methods:** Family members of patients who were admitted to intensive care unit for more than 48 hours or over were included in the study. Families were approached with a validated FS24- ICU family satisfaction survey questionnaire [1]. One hundred questionnaires were collected over a period of 3 months from January 2016 to March 2016 in our pediatric medical surgical and cardiac, adult cardiac and adult medical/surgical intensive care units.

**Results:** The overall level of satisfaction rate was comparable to other high-income and developed countries with total satisfaction score, medical care score and decision making score of 75.1 ± 14.2, 80.1 ± 18.6, and 68.1 ± 11.5 respectively (Table 1).

**Conclusions:** This is the very first study from the UAE demonstrating a high level of patient family satisfaction in both adult and pediatric intensive care units. This study also highlighted areas where further improvement needed to occur.

**References**

1. Wall RJ, Engelberg RA, Downey L, Heyland DK, Curtis JR Crit Care Med 35:271–279, 2007

**Table 1 (abstract P523).** Comparison of the total score and sub scores across different studies

	UAE study	Canadian study	Swiss study	American Study
Tool used	FS-24	Modified FS-ICU	FS-34	Modified FS-ICU and QOD-1
Total score	75.1 ± 14.2	84.3 ± 15.7	78 ± 14.7	76.6 ± 20.6
Care score	80.1 ± 18.6	83.5 ± 15.4	79 ± 14	77.7 ± 20.6
Decision making score	68.1 ± 11.5	75.9 ± 26.4	77 ± 15	75.2 ± 22.6

## P524 Breaking bad news in the emergency department: a randomized controlled study of a training using role-play simulation

### I Bragard^1^, JC Servotte^1^, I Van Cauwenberghe^2^, A Donneau^1^, N Dardenne^1^, R Tubes^1^, B Cardos^1^, M Guillaume^1^, A Ghuysen^1^

#### ^1^Université de Liège, Liège, Belgium; ^2^University Hospital Centre of Liege, Liège, Belgium

**Introduction:** This is a randomized controlled study aiming to assess the impact of an e-learning and a 2-hour role-play training in breaking bad news (BBN). BBN is an extremely stressful task for medical students [1-2]. Many do not feel sufficiently trained [3], while some widespread guidelines such as the SPIKES protocol exist [4]. The benefits of these protocols have been demonstrated in the oncology field [5], but little studied in the emergency field.

**Methods:** Medical trainees and residents were randomly assigned to the control group (CG) or to the experimental group (EG). Only EG received the e-learning and 2-hour role-play training. Both groups were assessed twice: at T0 and T1 (three weeks later). Each assessment included a video-recorded role-play with two actors playing the role of relatives, and questionnaires. Two blinded experts rated the videos.

**Results:** Out of 80 participants, 80% were trainees and 20% were anaesthesia residents. EG (n=43) and CG (n=37) were not different at baseline on the several variables. There were significant group and time interaction effects. Only EG increased their self-efficacy (from 2.6 to 85.4%, p < 0.001). EG improved more than CG their skills (respectively +86.5% and +13.8%, p<0.001) and EG decreased more than CG their inadequate communication skills (p < 0.0001).

**Conclusions:** A 2-hour role-play simulation blended with an e-learning on BBN in the emergency field appears to offer interesting perspectives. It enables a feasible approach as regards the acute care settings. Impact on real patients requires further evaluation in clinical settings.

**References**

1. Fallowfield L et al. The Lancet 363:312–319, 2004

2. Bragard et al. Journal of Health Psychology 15(7):1075-1081, 2010

3. Dosanjh et al. Medical Education 35:197–205, 2001

4. Baile et al. The Oncologist 5:302–311, 2000

5. Merckaert et al. British Journal of Cancer 109: 2507-2514, 2013

## P525

**Withdrawn**

## P526 Deficits of end-of-life care (EOLC) perceptions among physicians in intensive care units managed by anesthesiologists in Germany

### M Weiss^1^, A Michalsen^2^, A Toenjes^1^, F Porzsolt^1^, T Bein^3^, M Theisen^4^, A Brinkmann^5^, H Groesdonk^6^, C Putensen^7^, F Bach^8^, D Henzler^9^

#### ^1^University Hospital Medical School, Ulm, Germany; ^2^Tettnang Hospital, Tettnang, Germany; ^3^University, Regensburg, Germany; ^4^Raphaelsklinik GmbH, Akademisches Lehrkrankenhaus der Westfälischen Wilhelms-Universität Münster, Münster, Germany; ^5^Klinikum Heidenheim, Heidenheim, Germany; ^6^Saarland University Medical Center, Homburg/Saar, Germany; ^7^University Hospital Bonn, Bonn, Germany; ^8^Ev. Krankenhaus Bielefeld, Akad. Lehrkrankenhaus der WWU Münster, Bielefeld, Germany; ^9^Universitätsklinik der Ruhr-Universität Bochum, Klinikum Herford, Herford, Germany

**Introduction:** In order to apprehend the structural aspects and current practice of end-of-life care (EOLC) in German intensive care units (ICUs) managed by anesthesiologists, a survey was conducted to explore implementation and relevance of these items.

**Methods:** In November 2015, all members of the German Society of Anesthesiology and Intensive Care Medicine (DGAI) and the Association of German Anesthesiologists (BDA) were asked to participate in an online survey to rate 50 items. Answers were grouped into three categories: Category 1 reflecting high implementation rate and high relevance, category 2 low implementation and minor relevance, and category 3 low implementation and high relevance.

**Results:** Five-hundred and forty-one anesthesiologists responded, representing just over 1/3 of anesthesiology departments running ICU’s. The survey revealed new insights into current practice, barriers, perceived importance, relevance, and deficits of EOLC decisions. Only four items reached >= 90% agreement as being frequently performed, and 29 items were rated “very” or “more important”. 28 items attributed to category 1, 6 to category 2, and 16 to category 3, representing a profound discrepancy between current practice and attributed importance. Items characterizing the most urgent need for improvement (category 3) referred to desirable quality of life, patient outcome data, preparation of health care directives and interdisciplinary discussion, advanced care planning, distinct aspects of changing goals of care, standard operating procedures, implementation of practical instructions, continuing EOLC education, and inclusion of nursing staff and families in the process.

**Conclusions:** The survey generated awareness about deficits in EOLC matters in critical care. Consequently, already available EOLC tools have been made available through the website of the German Society of Anesthesiology and Intensive Care Medicine (DGAI): http://www.ak-intensivmedizin.de/arbeitsforen.html.

## P527 Changes in end-of-life practices (EOLP) in European intensive care units (ICUs)- the Ethicus II study

### L Busse^1^, C Hartog^2^, B Ricou^3^, A Michalsen^4^, C Feldman^5^, P Levin^6^, M Baras^7^, M Weiss^8^, HH Bulow^9^, A Avidan for the Ethicus II investigators.^7^

#### ^1^Emory St Joseph's Hospital, Atlanta, USA; ^2^University Hospital Jena, Jena, Germany; ^3^Hôpital Cantonal Universitaire de Geneve, Geneve, Switzerland; ^4^Tettnang Hospital, Tettnang, Germany; ^5^University of the Witwatersrand, Johannesburg, South Africa; ^6^Shaare Zedek Medical Center, Jerusalem, Israel; ^7^Hadassah Hebrew University Medical Center, Jerusalem, Israel; ^8^University Hospital Ulm, Ulm, Germany; ^9^Holbæk Hospital, Holbæk, Denmark

**Introduction:** This study evaluated differences in EOLP after 15 years in European ICUs that also participated in the Ethicus I study [1].

**Methods:** All previous Ethicus I centers were invited to participate in the Ethicus II study. Consecutive admitted ICU patients who died or had treatment limitations during a 6 month period from 1.9.2015 to 30.9.16 were prospectively studied. Previous EOLP and region definitions were used [1]. EOLP in the different regions of the Ethicus I study [1] were compared to the same ICUs in the Ethicus II study.

**Results:** 22 of the original 37 ICUs participated again in this study. Figure 1 shows the differences in EOLP by region. Figure 2 notes differences in patient mental competency at the time of decision, information about patient’s wishes and patient discussions in both Ethicus studies.

**Conclusions:** Changes included less CPR (especially in the South) with more withholding and withdrawing therapies. There was a greater number of competent patients with discussions and knowledge of their wishes.

**References**

1. Sprung CL et al. JAMA 2003; 290: 790-797

**Fig. 1 (abstract P527).**
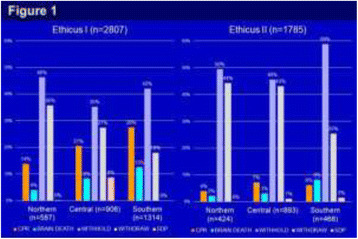
Differences in EOLP by region

**Fig. 2 (abstract P527).**
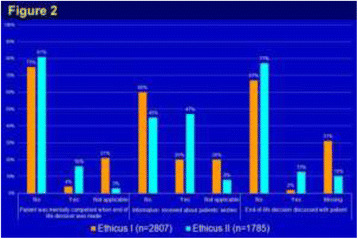
Differences in competency, patient wishes and patient discussions

## P528 Ethicus end-of-life practices (EOLP) in worldwide intensive care units (ICUs)- the ethicus II study

### A Avidan^1^, CL Sprung ^1^, B Ricou^2^, A Michalsen^3^, C Feldman^4^, M Anstey^5^, M Baras^1^, M Weiss^6^, H Bulow^7^, C Hartog^8^

#### ^1^Hadassah Hebrew University Medical Center, Jerusalem, Israel; ^2^Hôpital Cantonal Universitaire de Geneve, Geneve, Switzerland; ^3^Tettnang Hospital, Tettnang, Germany; ^4^University of the Witwatersrand, Johannesburg, South Africa; ^5^Sir Charles Gairdner Hospital, Perth, Australia; ^6^University Hospital Ulm, Ulm, Germany; ^7^Holbæk Hospital, Holbæk, Denmark; ^8^University Hospital Jena, Jena, Germany

**Introduction:** Substantial variability in EOLP occurs around the world [1]. Differences in EOLP were previously reported in Europe in the Ethicus I study [2].

**Methods:** ICUs worldwide were invited to participate through their country societies. Consecutive admitted ICU patients who died or had treatments limitations during a 6 month period from 1.9.2015 to 30.9.16 were prospectively studied. Regions included North, Central and Southern Europe (NE, CE, SE), North and Latin America (NA, LA), Asia (As), Australia (Au) and Africa (Af). Previous EOLP definitions were used [2].

**Results:** 199 ICUs in 36 countries participated enrolling 12,857 patients. Figure 1 shows differences in EOLP by region and Figure 2 in patient competency by region.

**Conclusions:** Worldwide differences included more CPR in Af, LA, and SE and less CPR in NE, Au and NA. There was more withdrawing (WD) in NE and Au and less WD in LA and Af. More patients were competent in Au and NE and less were competent in Af, SE and LA.

**References**

1. Mark, NM et al. ICM 2015;41:1572–1585

2. Sprung CL et al. JAMA 2003; 290:790-797

**Fig. 1 (abstract P528).**
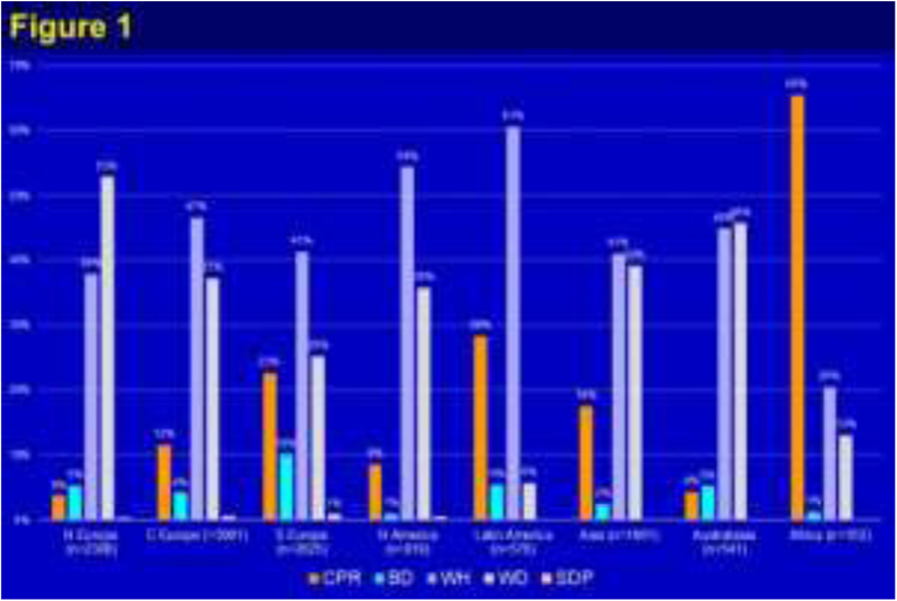
Differences in EOLP by region

**Fig. 2 (abstract P528).**
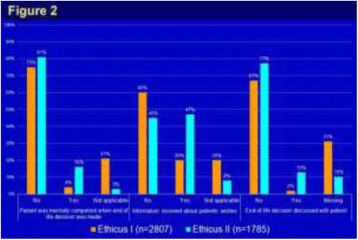
Patient mental compentency by region

## P529 Multidisciplinary team perceptions about terminal extubation in a teaching hospital in Brazil

### S Ribeiro, M Fukuda, J Alencar, P Almeida, R Brandao-Neto

#### Clinics Hospital, University of Sao Paulo Medical School, Sao Paulo, Brazil

**Introduction:** Palliative extubation is performed in patients with terminal ilnesses in which mechanical ventilation might prolong suffering. Even though the procedure involves nurses, respiratory therapists and doctors, some professionals feel unconfortable performing a palliative extubation. The concept of withdrawing life support can be easily confounded with euthanasia, specially in low income countries, where there is usually less education on palliative care.

**Methods:** A questionary containing 6 open ended questions concerning a hypotetical case of intracerebral hemorrhage and prolonged coma, with potential indication for palliative extubation was applied to 13 members of an emergency department intensive care unit staff (6 doctors, 4 nurses, 3 respiratory therapists (RT).

**Results:** More than half of the professionals (61%) had never participated in a palliative extubation. Four professionals (30%) believed palliative extubation is euthanasia. When asked about their own preferences in such a situation, only two (15%) would like to be tracheostomized. Symptoms anticipated by most professionals were dyspnea and respiratory secretions. Four (30%) would feel very uncomfortable performing palliative extubation because they either felt to be killing the patient or unable to manage symptoms

**Conclusions:** Most professionals in this tertiary emergency intensive care unit never witnessed a palliative extubation. However, most of believe this procedure is beneficial. Some still cannot understand the difference between palliative extubation and euthanasia. Education in palliative care and withdrawal of life support can be helpful to clear concepts and relieve moral distress in the team.

**References**

1. Chotirmall SH, et al J Crit Care. 25(2):360. e361–e368, 2010

## P530 Changing thoughts about end-of life care in the ICU; results of a survey

### M Kikuchi, A Yaguchi, M Kang, M Saito, M Tsunoda, T Oshiro, A Kim, K Shibahara, K Mochiduki, N Saito

#### Tokyo Women’s Medical University, Tokyo, Japan

**Introduction:** The decision of end-of-life care in the ICU is very tough issue because the law, ethics, traditions and futility should be concerned involving family's will. Especially, stop or withdraw therapy is a quite difficult operation in Japan because of our traditions. Recently there are few legal issues due to some guidelines. Our hypothesis is some difference over time exists in thoughts about end-of-life care in the ICU. The purpose of this study is to know changing

**Methods:** A questionnaire survey, which consists of 11 questions with 5 optional answers related to the thoughts of participants about end-of-life care of hopeless or brain death patients, was performed to nurses and doctors in our ICU. The questions were; whether accept to withdraw therapy or not and with family's will, whether positive or not to donate of organs from brain death patient, necessary of ICU care for brain death patient, feel guilty and stress for doing stop or withdraw therapy. The optional answer has 5 gradations from 'Yes' to 'No' for all questions. It was guaranteed to be anonymous for them in the data analysis. We conducted entirely same survey in 2012. The answers between in 2012 and in 2017 were compared. Mann-Whitney U test was used for statistical analysis. A p<0.05 was considered statistically significant.

**Results:** There were total 51 participants (32 nurses and 19 doctors) in 2012 and 42 participants (23 nurses and 19 doctors) in 2017. The acceptances of withdraw therapy in nurses were significantly decreased in 2017 than in 2012 (40% vs. 83%, 53% vs. 83%, p<0.05, respectively), while no changed in doctors. The positive of organ transplantation from brain death was also decreased in nurse in 2017 than in 2012 (43% vs. 80%, p<0.05).The feel guilty for withdraw therapy in nurses was also significantly decreased in 5 years (10% vs. 30%, p<0.05).

**Conclusions:** Some of end-of-life thoughts in the ICU were shown differences in nurses compared with 5 years ago.

